# Neuronal activity regulates alternative exon usage

**DOI:** 10.1186/s13041-020-00685-3

**Published:** 2020-11-10

**Authors:** Johanna Denkena, Andrea Zaisser, Barbara Merz, Bertram Klinger, Dietmar Kuhl, Nils Blüthgen, Guido Hermey

**Affiliations:** 1 Institute for Theoretical Biology and Institute of Pathology, Charité-Universitätsmedizin Berlin, Berlin, 10117 Germany; 2 Integrative Research Institute Life Sciences, Humboldt Universität Berlin, 10115 Berlin, Germany; 3 Institute for Molecular and Cellular Cognition, Center for Molecular Neurobiology Hamburg, University Medical Center Hamburg-Eppendorf, Falkenried 94, 20251 Hamburg, Germany

**Keywords:** Neuronal activity, Synaptic plasticity, Alternative splicing, Hippocampus, Gene expression, Transcriptome, Microarray, RNA sequencing

## Abstract

Neuronal activity-regulated gene transcription underlies plasticity-dependent changes in the molecular composition and structure of neurons. A large number of genes regulated by different neuronal plasticity inducing pathways have been identified, but altered gene expression levels represent only part of the complexity of the activity-regulated transcriptional program. Alternative splicing, the differential inclusion and exclusion of exonic sequence in mRNA, is an additional mechanism that is thought to define the activity-dependent transcriptome. Here, we present a genome wide microarray-based survey to identify exons with increased expression levels at 1, 4 or 8 h following neuronal activity in the murine hippocampus provoked by generalized seizures. We used two different bioinformatics approaches to identify alternative activity-induced exon usage and to predict alternative splicing, ANOSVA (ANalysis Of Splicing VAriation) which we here adjusted to accommodate data from different time points and FIRMA (Finding Isoforms using Robust Multichip Analysis). RNA sequencing, in situ hybridization and reverse transcription PCR validate selected activity-dependent splicing events of previously described and so far undescribed activity-regulated transcripts, including Homer1a, Homer1d, Ania3, Errfi1, Inhba, Dclk1, Rcan1, Cda, Tpm1 and Krt75. Taken together, our survey significantly adds to the comprehensive understanding of the complex activity-dependent neuronal transcriptomic signature. In addition, we provide data sets that will serve as rich resources for future comparative expression analyses.

## Introduction

Neurons go through activity-dependent alterations in their molecular composition and structure in order to fine-tune their synaptic strength. Such neuronal plasticity plays a vital role during a critical period in the development of the nervous system [1]. In the mature brain, neuronal plasticity contributes to sensory adaptation, learning and memory formation, as well as to a variety of pathological processes such as response to injury or epileptogenesis and neuropsychiatric or neurodegenerative disorders [2–5]. Posttranslational modifications of pre-existing proteins are thought to convey short-term activity-dependent synaptic changes, whereas long-term maintenance of synaptic adaptations relies on gene induction [6]. Signaling from the synapse to the nucleus induces gene expression followed by protein synthesis [7]. This leads to modifications in the composition of synaptic protein networks and provides a mechanism for translating synaptic activity into persistent changes of synaptic strength [7]. In accordance, a large number of genes whose expression level is altered by different neuronal plasticity inducing pathways were identified (reviewed in [8]). Indeed, several of these genes encode proteins that modulate synaptic function and play a role in learning and memory [9]. However, changes in gene expression levels represent only one aspect of the complexity of the transcriptional signature of neurons. Alternative splicing, the differential inclusion and exclusion of exonic sequence, allows a single gene to encode multiple protein isoforms with modified or even antagonistic properties. Alternative splicing of transcripts together with the use of alternative transcriptional initiation sites and polyadenylation sites expands the transcriptomic and proteomic diversity required for the regulation and diversification of specific functions [10–13]. Importantly, alternative splicing is not only controlled by intrinsic cell fate specific mechanisms but also dynamically adjusted by external stimuli. For instance, neuronal activity is thought to regulate splicing of a number of induced transcripts [10, 11]. However, so far most surveys focused only on gene expression levels and the description of neuronal activity-regulated splicing events is incomplete.

Different experimental paradigms have been employed to identify neuronal activity-regulated transcriptional programs [8, 9]. Initial in vivo approaches to identify transcriptional changes in excitatory neurons suffered from the fact that these changes were likely to be obscured or diluted by surrounding cell types not involved in the specific activity-dependent circuitry. Therefore, in vivo strategies aimed at intense global stimulation of an entire brain area which can be achieved by chemically induced or electroconvulsive seizures resulting in a robust, strong and synchronized induction of neuronal activity in the hippocampus [9]. In fact, screens for activity-dependent transcripts using seizure protocols as models for synaptic plasticity identified a large number of genes known to be necessary for memory consolidation [8, 9, 14–18].

In the present survey, we performed a genome wide analysis of alternative splicing events in the hippocampus of mice at different time points after chemically induced seizures. We used exon-specific microarrays and analyzed the obtained data by two different bioinformatics methods to identify alternatively used exons. We employed FIRMA (Finding Isoforms using Robust Multichip Analysis) which uses normalized scores without measure of significance and predicts splicing on exon level [19] and ANOSVA (ANalysis Of Splicing VAriation) which uses a statistical test approach and predicts splicing on gene level for single time points [20]. Here, we extended ANOSVA to accommodate data from different time points. By applying these methods, we identified alternative exon usage induced by neuronal-activity in previously described activity-regulated genes, but also of genes previously undescribed in this context. In independent experiments, we validated the differential expression of exons and splicing junctions by RNA-sequencing, RT-PCR and in situ hybridizations on hippocampal transcripts at corresponding time points after seizure induction.

## Results

### Analysis of neuronal activity-regulated genes

**Fig. 1:**
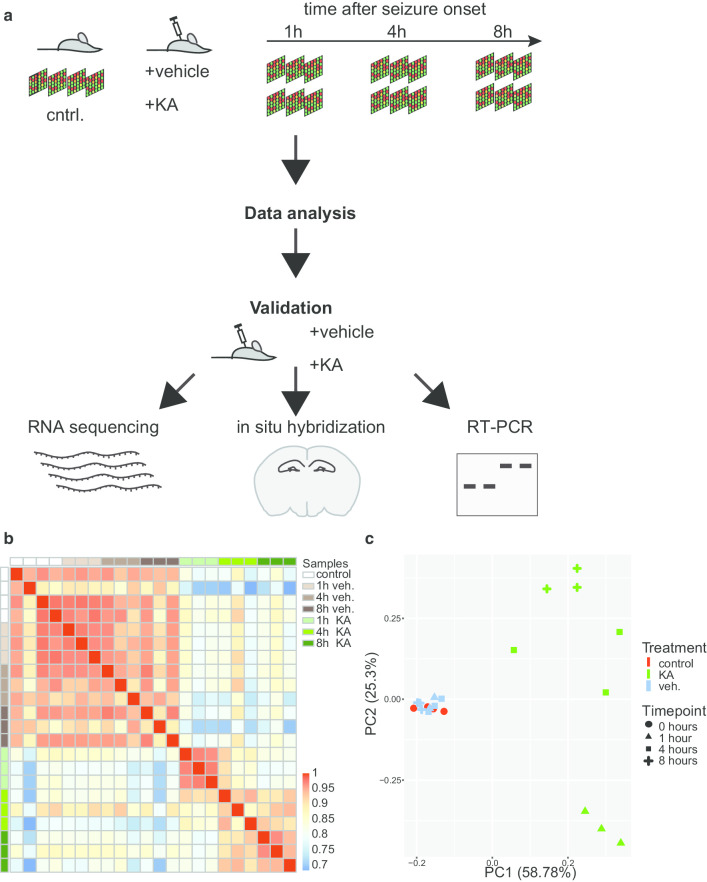
Strategy of the survey and validation of experimental groups. **a** Experimental setup. Differential expression of hippocampal RNA from untreated controls (cntrl, n = 4 replicates), from mice sacrificed at indicated time points after seizure onset (n = 3 replicates) and vehicle-treated time-matched controls (n = 3 replicates) was analyzed using exon microrarrays and exon usage was validated by RNA sequencing, in situ hybridization and RT-PCR. **b** Sample-wise correlation matrix of the 1000 genes with the highest standard deviation over all samples were extracted and used to construct a pairwise correlation matrix using Pearson correlation. **c** Principal component analysis of individual factor maps of significantly regulated genes. The two principal components PC1 and PC2 represent treatment and time after seizure onset, respectively. *KA* samples at indicated timepoints after kainic-acid induced seizure onset, *veh.* vehicle-treated time-matched controls

We triggered seizures in mice to induce strong synchronous neuronal activity in the hippocampus for a comprehensive analysis of alternatively spliced gene transcripts induced by neuronal activity. To identify temporally distinct patterns of gene expression, we treated mice with kainic acid or vehicle. We obtained hippocampal tissue for microarray analysis from animals sacrificed 1, 4 or 8 h after seizure onset and from time-matched vehicle-treated controls. In addition, we included in our analysis untreated control mice (Fig. 1a). RNA extracted from one hippocampus was hybridized to one microarray. We measured replicates of four animals for the untreated controls and three time-matched replicates for vehicle and kainic acid treatments respectively. We used exon microarrays, covering almost all exons of the murine transcriptome and containing approximately four probes per exon.

To estimate the variability between samples of the same and between different conditions, the 1000 genes with the highest inter-sample variability were used for unsupervised clustering on the correlation of transcriptomes (Fig. 1b). We observed that untreated and vehicle-treated control samples group together and the samples collected after seizures form a second cluster with the highest similarity among the samples obtained 1 h after seizure onset. Principal component (PC) analysis corroborated that expression data of the untreated and vehicle-treated samples group together (Fig. 1c). Hippocampal gene expression from mice after seizure was significantly different from the controls (PC1, treatment). Although the seizure samples differ more between and within the groups than the controls, these samples clearly group together and the three kainic acid-treated groups separated by time in the second component (PC2, time). Taken together, these analyses confirm that expression profiles of parallel treated animals were mostly similar, and that the data separates effects of seizure induction.

At this first analyzing step, we identified on the whole gene level 719 transcripts significantly induced by neuronal activity at least at one time point after seizure onset (Additional file 1). These were differentially up- or downregulated and categorized due to their expression kinetics in immediate early genes, delayed upregulated genes, delayed downregulated genes and immediate downregulated genes (Additional files 1 and 2).

### Alternative splicing of neuronal activity-regulated genes

We applied two different methods to identify exons with differential activity-dependent expression. The ANOSVA method identifies genes with splice variation in two different experimental conditions, e.g. control and a single time point after induction [20]. In order to monitor splicing changes over time, we here adjusted the ANOSVA method to incorporate data from multiple time-points. We name this modified analysis time-dependent ANOSVA. We identified 22 genes with significant splicing variations when allowing for 5% false positives (Table 1, Additional file 3). 14 of these identified genes with splice variation were also identified on the whole gene level as induced by neuronal activity in our initial analysis (Table 1, Additional file 1).

**Table 1:** Genes with significant activity-dependent exon usage identified through time-dependent ANOSVA

Gene name	Min. adj. p value *	DE^a^
Krt75	1.62168 × e^−12^	−
Homer1	2.175754 × e^−07^	+
Coq10b	3.153267 × e^−06^	+
Rcan1	0.000386	−
Errfi1	0.000652	+
Bdnf	0.000654	+
Atf3	0.000654	+
Tpm1	0.000693	−
Vmp1	0.0015166	−
Cyr61	0.0021082	−
Cda	0.0034645	+
Sgk1	0.0100454	+
Nr4a1	0.0120587	+
Per1	0.0129348	+
Fos	0.0136829	+
Lif	0.0136829	−
Inhba	0.0195556	+
Sox11	0.0374124	+
Pim1	0.0374124	+
Cyp2d26	0.0391230	−
C330019L16Rik	0.0407994	−
Ankrd33b	0.0489575	+

*min. adj. p-value, minimal adjusted p value derived by Bonferoni and Holm method

^a^DE, differentially expressed, indicates whether the whole gene was identified as induced by neuronal activity in our initial analysis (Table S1)

**Fig. 2:**
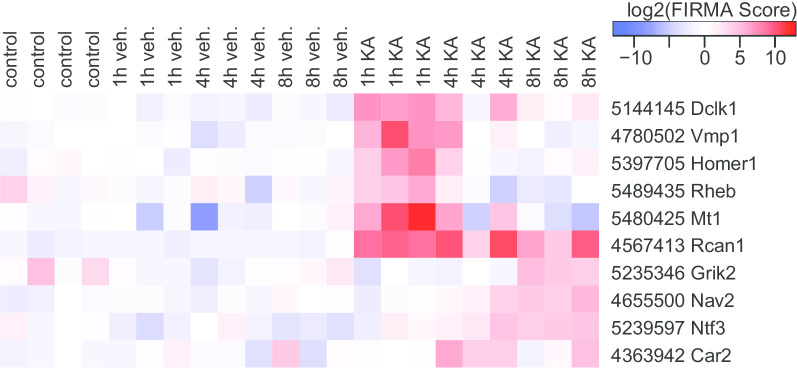
Heatmap of significant FIRMA scores. F scores were transformed to a log2 scale prior to compiling this heatmap and are the basis for the color key on the right. Affymetrix probeset IDs and corresponding gene names are shown on the right. Identity of experimental groups is indicated as controls (n = 4 replicates) and time points after kainic acid induced seizure onset (KA) and vehicle-treated time matched controls (veh.) (n = 3 replicates)

Next, we used FIRMA as a second independent method to identify activity-dependent splice variations. The FIRMA method operates under the assumption that exon skipping or retention is the most prevalent form of exon splicing. Therefore, an outlier detection is utilized to identify genes with alternatively used exons or probesets [19]. An F score, representing the discrepancy of one probeset from the other probesets of the same gene, is calculated for every probeset and sample. The F scores were combined into an all-sample score by choosing the maximum of the minimal F scores of one condition group and were used for false discovery rate (FDR) calculation. In Fig. 2 the F scores of all samples from the probesets with an FDR below 10% were visualized in a heatmap. The top scoring probesets display elevated scores in all samples of at least one condition group. Therefore, the method of choosing an all-sample score is adequate to identify probesets consistently deviating from the estimated expression level instead of simply detecting single random outliers. The 10 probesets fulfilling the demand for a FDR below 10% are shown in Table 2. Five of these 10 genes were detected as significantly activity-regulated on a whole gene level, and three were identified as highly significant by ANOSVA (Table 1, Additional file 1). A notable feature of the FIRMA method is the scoring by probesets instead of whole genes which already points at the differentially used exons (Fig. 2 and Table 2, Additional file 4). In contrast, only one minimal p-value is specified by the ANOSVA method and probesets have to be identified individually.

**Table 2:** Genes with significant activity-dependent exon usage identified through FIRMA

Gene name	Probeset ID	Probeset number	Exon number	F^s^ score^a^	F^0^ score^b^	FDR^c^	DE^d^
*Rcan1*	*4567413*	*4*	*3*	*444.6*	*0.345*	*0.0*	*−*
Dclk1	5144145	10	9	96.8	0.525	0.0	−
Mt1	5480425	1	2	70.9	0.621	0.0	+
*Vmp1*	*4780502*	*11*	*13*	*50.1*	*0.542*	*0.0*	*−*
Ntf3	5239597	7	3	14.8	0.371	0.067	+
Rheb	5489435	8	8	15.1	0.404	0.071	+
Grik2	5235346	9	8	15.5	121.96	0.077	+
Car2	4363942	22	13	16.1	0.347	0.083	−
*Homer1*	*5397705*	*6*	*2*	*161.6*	*0.581*	*0.091*	* + *
Nav2	4655500	3	9	169.6	0.841	0.1	−

FIRMA (F) scores represent the discrepancy of one probeset from the other probesets in the same gene and were calculated for every probeset and sample

Italics indicates genes also found highly significantly spliced using time-dependent ANOSVA (Table 1)

^a^F^s^ score, corresponds to samples collected after seizure compared to controls and samples

^b^F^0^ score, corresponds to samples collected after vehicle treatment compared to controls

^c^FDR, False discovery rate calculated from F scores for the alternative and the null model

^d^DE, differentially expressed, indicates whether the whole gene was identified as induced by neuronal activity in our initial analysis (Table S1)

Using two different methods, we identified alternative exon usage induced by neuronal-activity. Among the identified alternative spliced transcripts are already described, canonical activity-regulated genes, but also genes previously undescribed in this context.

### Validation of activity-induced alternative exon usage

To validate the observed alternative exon usage, we first visualized the data obtained from the exon arrays for selected genes. We depicted probe-wise and probeset-wise expression of transcripts as well as the log2 ratios for all analyzed time points, as shown in Additional file 5. Moreover, we aligned all probesets of a gene to the corresponding exons to compare these to known transcripts (Additional file 5). Besides, we generated and surveyed an independent RNA-sequencing data set (see overview in Fig. 1a). Selected exon spanning RNA sequencing results were verified by RT-PCR using exon spanning primer pairs (Additional files 6 and 7) and hippocampal RNA from independent experiments in which control mice and mice sacrificed 1 or 4 h after seizure onset were used. Finally, two alternative splicing events were confirmed by in situ hybridization.

For validation experiments, we choose the most significant genes identified by ANOSVA and FIRMA, Krt75 and Rcan1. For supplementary validation experiments, we aimed at a balance between genes identified as induced by neuronal activity in our initial analysis (Additional file 1) and genes that were not identified on the whole gene level. Moreover, we selected genes with an expected rather simple splicing pattern such as alternative 5′-exon usage, as observed for Cda, Erffi1 and Inhba, and genes with a rather complex splicing pattern and multiple activity-regulated splice variants, such as Homer1, or at least one activity-regulated splice variant, such as Tpm1 and Dclk1.

**Fig. 3:**
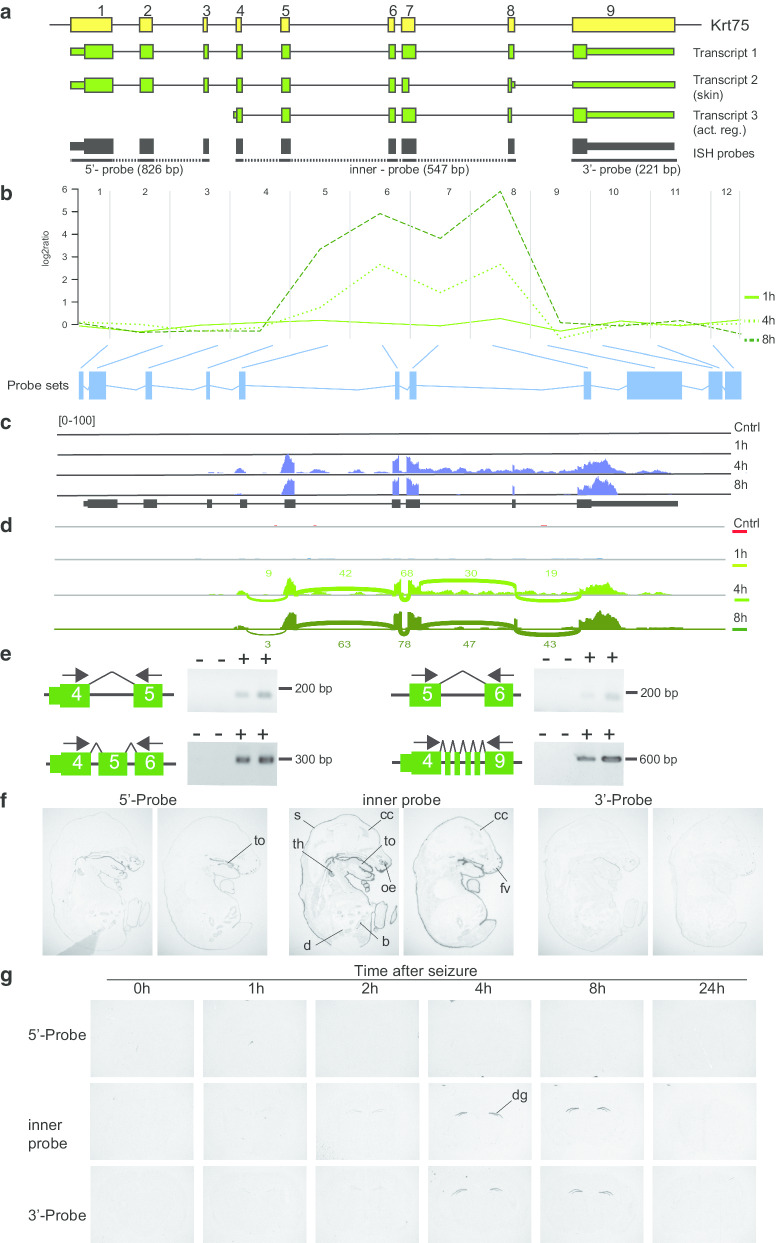
Exon usage of neuronal activity regulated Krt75. **a** Krt75 is encoded by 9 exons (yellow boxes, not to scale). Transcript 1 represents the canonical variant. Transcript 2 and 3 were identified in this study. Larger boxes represent coding sequence, smaller boxes untranslated regions. Probes used for in situ hybridization (ISH) indicated in black. **b** Visualization of the microarray data. The plot shows the log2 ratio calculated from the mean normalized intensities of transformed expression values over the course of probesets per exon. Green lines represent indicated expression kinetics. Blue boxes represent probesets per exon present on the microarrays. **c** Validation of exon expression in hippocampus of control mice or 1, 4 or 8 h after seizure onset by RNA sequencing. Shown are coverage plots (blue) from paired-end reads. The reference gene track is depicted below (black). **d** Sashimi plot of RNA sequencing data. Numbers indicate the counts of RNA sequencing reads that span the respective exon junctions. **e** Validation of splicing events by RT-PCR. Numbers indicate exons. Exon-spanning primers (arrows) were used to assess expression of exons in total RNA of hippocampi of control mice (−) and of mice sacrificed 4 h after seizure onset (+). The observed transcript size in the lower left panel indicates inclusion of exon 5 and in the lower right panel inclusion of exons 4–9 which was confirmed also by sequencing of the PCR product. **f** Autoradiograms of ISH of parallel sagittal sections of E16 embryonic mice with the three specific probes. **g** Autoradiograms of ISH of parallel coronal brain sections of mice sacrificed at indicated time points after seizure onset. ISH of one specific antisense probe was conducted in parallel on one glass slide. *b* bladder, *cc* cerebral cortex, *d* duodenum, *dg* dentate gyrus, *fv* follicles of vibrissae, *oe* olfactory epithelium, *s* skin, *th* thymus, *to* tongue

According to the time-dependent ANOSVA, the most significant alternatively spliced gene after neuronal activity is Krt75 (Table 1). The type-II cytokeratin Krt75 is highly expressed in hair follicle and in epithelia of the nail bed, but significant neuronal expression has not been reported before [21]. Gene specific analysis of our microarray data revealed an induction of probesets corresponding to exons 4, 6, 7 and the 5′ area of the terminal exon 9 (Fig. 3a, b and Additional file 5). The plot in Fig. 3b shows the log2 ratio calculated from the mean intensities at the three time points after seizure onset over the course of probesets per exon. The expression of Krt75 was below the median log2 expression value of all genes considered. This explains why Krt75 was not identified as significantly activity-regulated in our initial analysis on the whole gene level (Additional file 1). We analyzed our independent RNA sequencing data set and found an induction of Krt75 transcripts harboring exons 4–9 at 4 and 8 h after seizure onset (Fig. 3c, d). The increase in the relative expression level of the transcript is illustrated by the coverage plots from paired-end RNA-sequencing reads (Fig. 3c) and by the numbers in the Sashimi plots which indicate the counts of RNA sequencing reads that span the respective exon junctions (Fig. 3d). Notably, the microarray probesets did not represent Exon 5 and 8 (Fig. 3a, Additional file 5). We performed RT-PCR to demonstrate splicing of Krt75 exons. Using exon spanning primer pairs, we verified activity-regulated expression of exon 4 and 5, 5 and 6, and amplified a fragment corresponding to exon 4 to 6 including exon 5 by using primer specific for exon 4 and 6 (Fig. 3e). Finally, we used primer corresponding to exon 4 and 9 to amplify the complete transcript. DNA sequencing of the amplified product demonstrated usage of all exons 4–9 in the activity-regulated transcript 4 h after seizure onset. Thus, neuronal activity induces expression of a transcript that is truncated at its 5′-end when compared with the canonical transcript 1 (Fig. 3a, Additional file 8). The intron sequence upstream of exon 4 presents no alternative in-frame start codon. In exon 4, the sequence surrounding the first start codon corresponds to the Kozak sequence. It begins with the translated amino acid sequence MNKVE which is highly conserved in humans, chimpanzee, rat and chicken. The terminal exon nine harbors two canonical polyadenylation motifs (AATAAA and ATTAAA) and both are present in the activity-regulated transcript.

To inspect tissue specific expression of Krt75 in the brain in more detail, we performed in situ hybridizations using three different probes. The first corresponds to the 5′-region of the transcript that was not induced after seizures, the second corresponds to the activity-regulated exons 4–8, and the third to the 3′-terminal exon 9 (Fig. 3a). To obtain cDNA to generate the first probe, we cloned Krt75 from skin, amplified the canonical transcript and as well a previously undescribed splice variant that encodes a C-terminal truncated protein (Additional file 8). Next, we demonstrated functionality of the three different in situ probes on parallel sagittal sections of E16 mouse embryos (Fig. 3f). We detected with all three probes specific expression of Krt75, as expected, in the skin, but as well in epithelia of tongue, duodenum and bladder. Moderate expression in the developing cerebral cortex was observed only with the inner-probe and on a lower level with the 3′-probe, which is considerably shorter than the other probes and yielded in lower signal intensity. We hybridized parallel coronal sections of brains from adult mice sacrificed at different time points after onset of seizures (Fig. 3g). Under control conditions none of the three probes revealed expression in brain. We observed low expression 1 and 2 h after seizure onset only with the inner- and the 3′-probe. This expression was restricted to the dentate gyrus and more pronounced 4 and 8 h after seizure onset (Fig. 3g). The results are in accordance with our initial finding that Krt75 truncated at the 5′-end is expressed after neuronal-activity in the hippocampus.

**Fig. 4:**
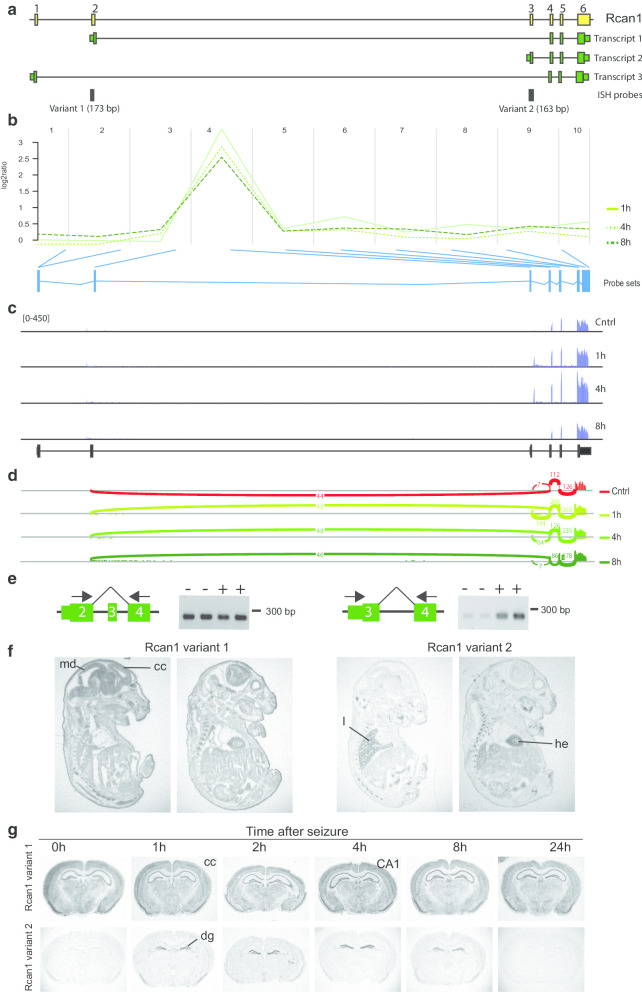
Activity dependent alternative splicing of Rcan1. **a** Rcan1 is encoded by 6 exons (yellow boxes, not to scale). The first three exons are mutually exclusive and their alternative usage results in transcript 1, 2 and 3 (green). The location of two alternative probes used for in situ hybridization (ISH) are shown in black. **b** Visualization of the microarray data. The plot shows the log2 ratio calculated from the mean normalized intensities of transformed expression values over the course of probesets per exon. Green lines represent indicated expression kinetics. Blue boxes represent the probesets per exon present on the microarrays. **c** Validation of Rcan1 variant expression in hippocampus of control mice or 1, 4 or 8 h after seizure onset by RNA sequencing. Shown are coverage plots (blue) from paired-end reads. The reference gene track is depicted below (black). **d** Sashimi plot of RNA sequencing data. Numbers indicate the counts of RNA sequencing reads that span the respective exon junctions. **e** Validation of splicing events by RT-PCR. Numbers indicate exons. Exon-spanning primer pairs (arrows) were used to assess expression of exons in total RNA of hippocampi of control mice (−) and of mice sacrificed 4 h after onset of seizures (+). Note that the observed transcript size in the left panel indicates exclusion of exon 3 and that the forward primer in the right panel corresponds to the 5′-UTR of exon 3. **f** Autoradiograms of ISH of parallel sagittal sections of E16 embryonic mice with the variant specific Rcan1 probes on parallel sections. **g** Autoradiograms of ISH of parallel coronal brain sections of mice sacrificed at indicated time points after seizure onset. ISH of one specific antisense probe was conducted in parallel on one glass slide. *CA1* field CA1 of the hippocampus, *cc* cerebral cortex, *dg* dentate gyrus, *he* heart, *l* lung, *md* midbrain

The most significantly altered expression observed by FIRMA corresponds to a probeset that represents exon 3 of RCAN1 (regulator of calcineurin-1) (Table 2). Time-dependent ANOSVA identified as well Rcan1 as an activity-regulated spliced gene (Table 1). Notably, Rcan1 was not identified as activity-regulated on the whole gene level in our initial analysis (Additional file 1). This is likely due to only one probeset (4567413) showing a strong upregulation of log2 ratios by 2.5 to 3.5 whereas the levels of all other probesets are almost unchanged (Additional file 9). The Rcan1 gene comprises 6 exons and the first three are mutually exclusively used as the initial exon in three alternative splice variants (Fig. 4a). The induced probeset corresponds to exon 3, which is present only in transcript variant 2 (Fig. 4a, b). RNA sequencing confirms induction of exon 3 and most likely of variant 2 which comprises exon 3–6 at the 1 and 4 h time point (Fig. 4c, d). Moreover, the sequencing results suggest that variant 1 which comprises exon 2 and exons 4–6 is constitutively expressed in the hippocampus. Using exon spanning primer pairs, we demonstrate that a transcript comprising exon 2 and 4, but not exon 3 is expressed under control conditions and 4 h after seizure onset, whereas a transcript expressing exon 3 and 4 is nearly undetectable under control conditions and clearly detectable 4 h after seizure onset (Fig. 4e). In addition, we used probes specific for exon 2 or exon 3 for in situ hybridizations (Fig. 4a). Exon 2 is widely expressed in embryonic tissue whereas expression of exon 3 is restricted to specific organs such as lung and heart and almost undetectable in embryonic brain (Fig. 4f). In adult murine brain, variant 1 is constitutively and widely expressed and the expression level is nearly unchanged after seizures (Fig. 4g). In contrast, variant 2 is almost undetectable under control conditions and markedly induced in the dentate gyrus of the hippocampus already 1 h after seizure onset (Fig. 4g). These experiments demonstrate induction of an alternative splice variant of Rcan1 after neuronal activity provoked by seizures. To assess if these observations are limited to seizure induced neuronal activity, we analyzed an additional RNA sequencing data set derived from primary cultured hippocampal neurons in which controls were compared with neurons 3 h after potassium-induced depolarization [22]. For activity-dependent expression of Rcan1, we observed a high constitutive expression of variant 1 and a significant induction of Rcan1 variants 1 and 2 (Additional file 10).

**Fig. 5:**
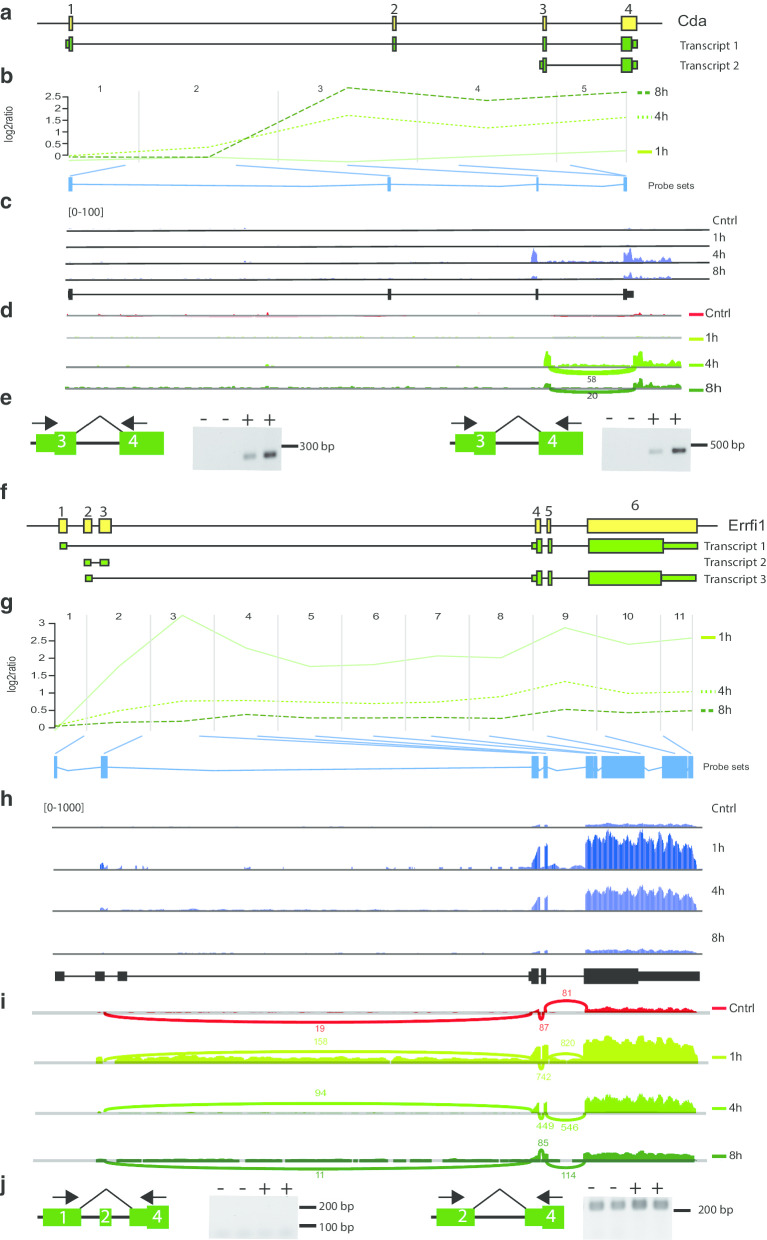
Activity dependent alternative splicing of Cda and Errfi1. **a** Cda is encoded by 4 exons (yellow boxes, not to scale). Transcript 1 comprises all 4 exons, transcript 2 only exon 3 and 4 and exon 3 has a 5′ extension. **b** Visualization of the microarray data. The plot shows the log2 ratio calculated from the mean normalized intensities of transformed expression values over the course of probesets per exon. Green lines represent expression kinetics of one time point after seizure onset. Blue boxes represent probesets per exon present on the microarrays. **c** Validation of Cda variant expression in hippocampus of control mice or 1, 4 or 8 h after seizure onset by RNA sequencing. Shown are coverage plots (blue) from paired-end reads for the four sample groups. The reference gene track is depicted below (black). **d** Sashimi plot of RNA sequencing data. Numbers indicate the counts of RNA sequencing reads that span the respective exon junctions. **e** Validation of splicing events by RT-PCR. Numbers indicate exons. Exon-spanning primers (arrows) were used to assess expression of exons in total RNA of hippocampi of control mice (−) and of mice sacrificed 4 h after seizure onset (+). Note that the forward primer corresponds to the 5′-UTR of exon 3 that is specific for transcript 2. The reverse primers correspond to 5′- or 3′-terminal sequence in exon 4. **f** Errfi1 is encoded by 6 exons. The first two exons are mutually exclusive and their alternative usage results in transcript 1 or 3. Transcript 2 comprises no open reading frame. **g** Visualization of the microarray data. **h** Validation of Errfi1 variant expression by RNA sequencing. **i** Sashimi plot of RNA sequencing data for Errfi1. **j** Validation of splicing events by RT-PCR. Note that no product was amplified by using Exon 1 and 4 specific primers

In addition to Rcan1 other genes with alternative 5′-exon usage were found in our analysis. Examples are Cda, Errfi1 and Inhba identified by time-dependent ANOSVA (Table 1). All three genes were as well identified as activity-regulated on the whole gene level. Cda is encoded by 4 exons and two transcripts harboring all 4 exons or only exon 3 and 4 are known (Fig. 5a). Probesets corresponding to exon 3 and 4 indicate activity-regulated expression 4 and 8 h after seizure onset (Fig. 5b, Additional file 11). RNA sequencing confirms induction of exon 3 and 4 (Fig. 5c, d). Using exon spanning primer pairs, we demonstrate that a transcript comprising the 5′-UTR of exon 3 and coding sequence of exon 4 are expressed 4 h after seizure onset, but not under control conditions (Fig. 5e). Attempts to amplify a full-length transcript 1 failed under both conditions. These data strongly suggest activity-regulated expression of only the shorter splice variant 2.

Six exons correspond to the Errfi1 gene (Fig. 5f). Two protein coding transcripts are known, exon 1 and 2 are mutually exclusive initial exons and spliced to exon 4–6. In addition, a non-coding transcript comprising exon 2 and 3 has been described (Fig. 5f). Analysis of our microarray data revealed that exon 1 expression levels are not altered, whereas exons 2 and 4–6 are induced 1 and 4 h after seizure onset (Fig. 5g, Additional file 11). RNA sequencing confirmed induction of exons 2 and 4–6 (Fig. 5h, i). Using exon spanning primer pairs, we could not detect expression of a transcript comprising exons 1 and 4 in murine hippocampus, but a transcript comprising the complete exons 2 and 4 appeared constitutively expressed and induced 4 h after seizure onset (Fig. 5j). RNA sequencing data of dissociated primary neuronal cultures in which neuronal activity was evoked by potassium-induced depolarization is in line with our observations and show specific induction of activity-regulated exons 2 and 4–6 (Additional file 12).

**Fig. 6:**
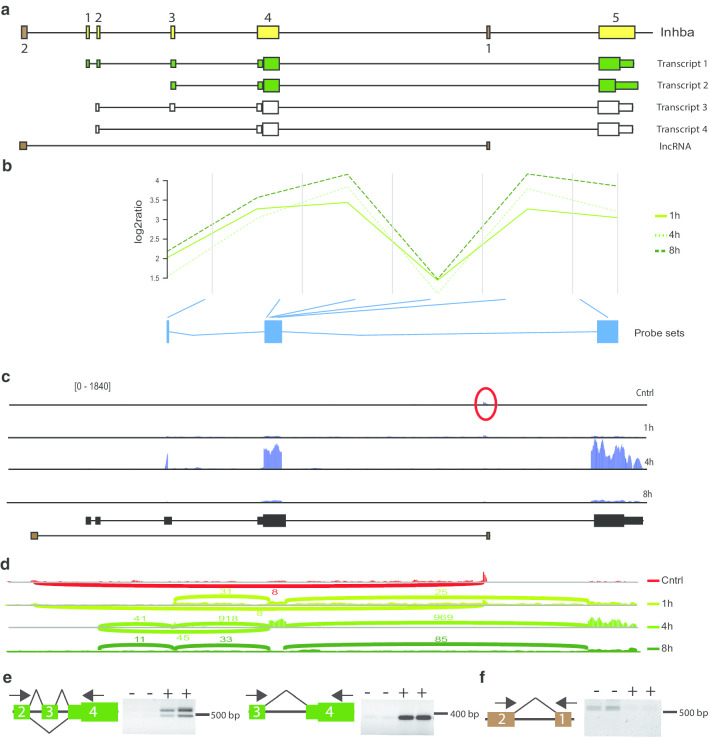
Activity dependent alternative splicing of Inhba. **a** Inhba is encoded by 5 exons (numbered, yellow boxes, not to scale). Transcript 1 and 2 (green) were described before. Transcript 3 and 4 (white) were identified in this study. Two additional transcribed exons encoding an lncRNA (brown), one upstream and one between exon 4 and 5 of Inhba are shown. **b** Visualization of the microarray data obtained for Inhba. The plot shows the log2 ratio calculated from the mean normalized intensities of transformed expression values over the course of probesets per exon. Each green line represents expression kinetics of one time point after seizure onset. The blue boxes represent the probesets per exon present on the microarrays. **c** Validation of Inhba variant expression in hippocampus of control mice or 1, 4 or 8 h after seizure onset by RNA sequencing. Shown are coverage plots (blue) from paired-end reads for the four sample groups. The reference gene tracks are depicted below (black, Inhba and brown, lncRNA). The red circle highlights expression of one exon of the lncRNA. **d** Sashimi plot of RNA sequencing data for cda. Numbers indicate the counts of RNA sequencing reads that span the respective exon junctions. **e**, **f** Validation of splicing events by RT-PCR using indicated exon-spanning primer pairs (arrows). The respective exons are indicated by numbers and in green for Inhba (**e**) and in brown for the lncRNA (**f**). Primers were used to assess expression of exons in total RNA of hippocampi of control mice (−) and of mice sacrificed 4 h after onset of seizures (+). Note that in the left panel two products were amplified, one corresponding in size to amplification of exon 2–4 and one to amplification of exon 2 and 4 and excluding exon 3

The gene Inhba comprises 5 exons and two transcripts have been described. One comprising all exons and the other exon 3–5 (Fig. 6a). Analysis of our microarray data revealed that exons 3–5 are induced after seizures (Fig. 6b, Additional file 13). However, the microarrays presented no probesets corresponding to exon 1 and 2. RNA sequencing confirmed induction of exons 3–5 one hour after seizure onset (Fig. 6c). In addition, RNA sequencing revealed an upregulation of exon 2 combined with exon 3 or alternatively with exon 4 at the 4 h time point, whereas at the 8 h time point exon 2 is combined only with exon 3 (Fig. 6d). Using exon spanning primer pairs, we detected expression of transcripts comprising exons 2–4 and alternatively 2 and 4 excluding exon 3 in murine hippocampus 4 h after seizure onset (Fig. 6e). Independently, we detected activity-regulated expression of exon 3 and 4 (Fig. 6e). These data demonstrate activity-dependent expression of transcript variant 2 and in addition induction of previously undescribed transcripts comprising exons 2–5 and a variant excluding exon 3.

In addition, the RNA sequencing data revealed expression of an uncharacterized reversed long non-coding RNA (lncRNA) B230303A05Rik originating from the intronic region between exon 4 and 5 of Inhba as well as from intergenic regions upstream of Inhba under control conditions, but not 4 or 8 h after seizure onset (Fig. 6a, c, d). RT-PCR using exon spanning primer pairs confirmed expression of this lncRNA under control condition but not 4 h after seizure onset (Fig. 6f) and corroborates the inverse correlation of Inhba expression and of its intronic lncRNA.

**Fig. 7:**
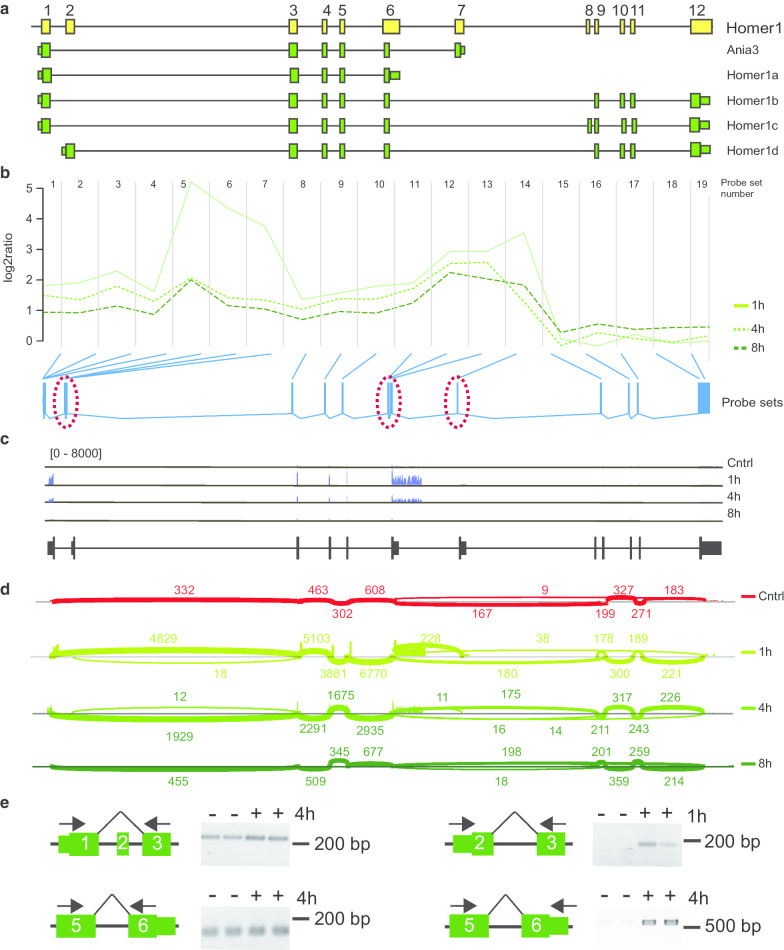
Activity dependent alternative splicing of Homer1. **a** Homer1 is encoded by 12 exons (yellow boxes, not to scale). Differential exon usage results in at least 5 splice variants (green). Larger boxes represent coding sequence and smaller boxes untranslated regions. **b** Visualization of the microarray data. The plot shows the log2 ratio calculated from mean normalized intensities of transformed expression values over the course of probesets per exon. Green lines represent expression kinetics of one time point. Blue boxes represent probesets per exon present on the microarrays. Red dotted circles highlight probesets corresponding to the second alternative 5′ exon which is featured in Homer1d, as well as probesets corresponding to the prolonged alternative 3′-end of exon 6, featured in Homer1a and probesets corresponding to the prolonged alternative 3′-end of exon 7, featured in Ania-3. **c** Validation of Homer1 variant expression in hippocampus of control mice or 1, 4 or 8 h after seizure onset by RNA sequencing. Shown are coverage plots (blue) from paired-end. The reference gene track is depicted below (black). **d** Sashimi plot of RNA sequencing data. Numbers indicate counts of RNA sequencing reads that span the respective exon junctions. **e** Validation of splicing events by RT-PCR. Numbers indicate exons. Exon-spanning primers (arrows) were used to assess expression in total RNA of hippocampi of control mice (−) and of mice sacrificed 4 h (+, 4 h) or 1 h after seizure onset (+, 1 h). Note that the product size in the upper left panel demonstrates exclusion of exon 2. The forward primer corresponding to exon 2 in the upper right panel is specific for the 5′-UTR. The reverse primer in the lower panels correspond to exon 6 and is specific for the coding region present in all transcripts (left) or specific for the 3′UTR of exon 6 present in Homer1a only (right)

Homer1 was identified by both applied methods, time-dependent ANOSVA and FIRMA (Tables 1, 2). The Homer1 gene comprises 12 exons and at least 5 splice variants are expressed suggesting a rather complex splicing pattern (Fig. 7a). Homer1a and Ania3 are previously described activity-regulated splice variants of Homer1 [23–25]. Notably, these do not include exon 2. Here, the FIRMA method identified exon 2 as activity-regulated (Table 2). Analysis of our microarray data shows increased expression of the first seven exons of Homer 1 after neuronal activity, whereas expression of exon 8–12 is unchanged (Fig. 7b, Additional file 14). Strongest induction of transcript levels was observed one hour after seizure onset with probesets corresponding to exon 2. This strong induction does not persist over time and is reduced again 4 and 8 h after seizure onset. In addition, probesets corresponding to exon 6 and 7 demonstrate an induction but both exons differ in their induction kinetics (Fig. 7b, Additional file 14). These three highly induced exons are specific for single splice variants. Exon 2 is specific for Homer 1d, exon 6 elongated at its 3′-end is present only in Homer1a as a terminal exon and exon 7 elongated at its 3′-end is used only in Ania3 as a terminal exon (Fig. 7a). RNA sequencing data are in accordance with these observations and demonstrate highest and prolonged induction of exons 1, 3, 4, 5, and the elongated exon 6 (Fig. 7c). A shorter induction of the elongated exon 7 and only a moderate elevation of exon 2 levels were observed 1 and 4 h after seizure onset and are clearly visible in the corresponding Sashimi plot (Fig. 7d). We performed RT-PCR analysis to demonstrate that exons 1 and 3 are constitutively expressed on common transcripts and these expression levels appear slightly elevated 4 h after seizures (Fig. 7e). A combination of exon 2 and 3, which is specific for Homer 1d, is not expressed under control conditions in the hippocampus, but detectable 1 h after seizure onset (Fig. 7e). Exon 5 and 6 are expressed under control conditions. In contrast, the expression of a transcript harboring exon 5 and the 3′ elongated version of exon 6 that serves as a unique terminal exon for Homer1a is strongly induced 4 h after seizure onset (Fig. 7e). To assess if these observations are limited to seizure provoked neuronal activity, we analyzed the additional RNA sequencing data set derived from primary cultured hippocampal neurons in which controls were compared with neurons 3 h after potassium-induced depolarization [22]. We observed a similar induction of Homer1 transcripts with highest induction of the elongated exon 6 and moderate induction of the elongated exon 7 and exon 2 levels (Additional file 15). Taken together, these data demonstrate the neuronal activity-regulated differential induction of exons specific for Homer1a and Ania3, which has been described before, and to our knowledge for the first time an activity-regulated induction of Homer1d.

**Fig. 8:**
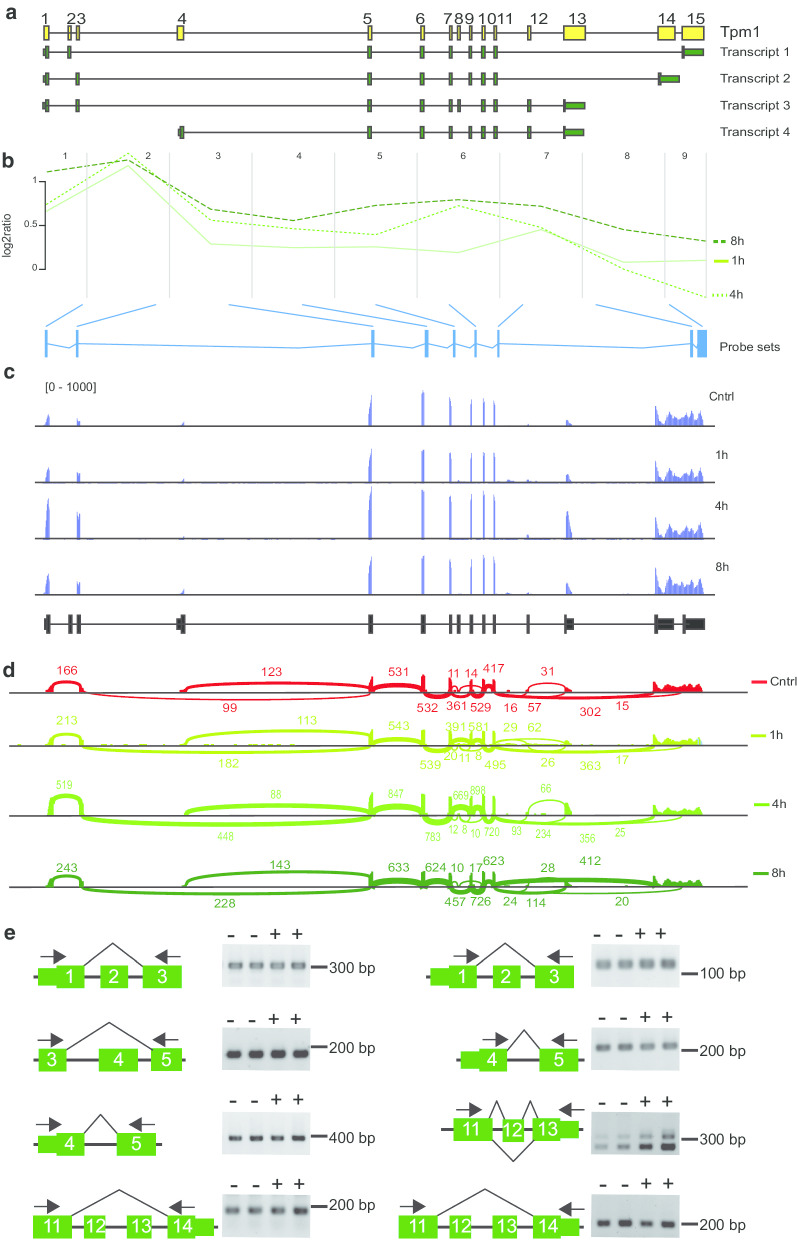
Activity dependent alternative splicing of Tpm1. **a** Tpm1 is encoded by 15 exons (yellow boxes, not to scale). Differential exon usage results in at least 21 splice variants. Of these, 4 are shown in green. Larger boxes represent coding sequence and smaller boxes untranslated sequence. **b** Visualization of the microarray data obtained for Tpm1. The plot shows the log2 ratio calculated from the mean normalized intensities of transformed expression values over the course of probesets per exon. Each green line represents expression kinetics of one time point after seizure onset. The blue boxes represent the probesets per exon present on the microarrays. **c** Validation of Tpm1 variant expression in hippocampus of control mice or 1, 4 or 8 h after seizure onset by RNA sequencing. Shown are coverage plots (blue) from paired-end reads for the four sample groups. The reference gene track is depicted below (black). **d** Sashimi plot of RNA sequencing data for Tpm1. Numbers indicate the counts of RNA sequencing reads that span the respective exon junctions. **e** Validation of splicing events by RT-PCR using indicated exon-spanning primer pairs (arrows). The respective exons are indicated by numbers. Primers were used to assess expression of exons in total RNA of hippocampi of control mice (−) and of mice sacrificed 4 h after onset of seizures (+). Note, forward primers corresponding to exon 1 and 4 match either to the 5′-untranslated sequence or to the coding sequence of the exon. The reverse primer matching exon 13 corresponds to the 3′ untranslated region and the revers primers corresponding to exon 14 match to the coding or the 3′ untranslated regions. The double bands produced by using primers corresponding to exon 11 and 13 indicate amplification of transcripts including (upper band) or excluding (lower band) exon 12

Other examples for activity-regulated exon selection in genes with complex splicing patterns are Tpm1 and Dclk1. We identified Tpm1 through time-dependent ANOSVA and on the whole gene level as an activity-regulated transcript (Table 1, Additional file 1). Alternative splicing of the Tpm1 gene is complex, as it comprises 15 exons and 21 different transcripts have been identified. Figure 8a depicts the 4 major transcripts. Unfortunately, not all exons were presented by probesets in our microarray analysis, but our analysis revealed that exons 1 and 3 are highly induced after seizures (Fig. 8a, b, Additional file 16). RNA sequencing confirmed induction of exon 1 and 3 (Fig. 8c, d). These analyses strongly suggest that exon 2 is not expressed in the hippocampus. RT-PCR using exon spanning primer pairs confirmed that transcripts spanning exon 1 and 3 are expressed in the hippocampus and these do not comprise exon 2 (Fig. 8e). Expression of transcripts comprising exon 3 and 5 but omitting exon 4 was verified by RT-PCR (Fig. 8e). However, parallel experiments demonstrated expression of transcripts comprising exon 4 (including its 5′-UTR) and exon 5 (Fig. 8d). Taken together, these data strongly suggest activity-regulated expression of transcripts comprising exon 1, 3 and 5, such as transcript 2 and 3 (Fig. 8a). RNA sequencing analysis further suggests that the terminal exon 13 is induced 4 h after seizure onset (Fig. 8c, d). RT-PCR using exon spanning primer revealed that transcripts with exon 13 as terminal exon comprise both either exon 11–13 or exon 11 and 13 omitting exon12 and both are expressed and most likely activity-induced in the hippocampus (Fig. 8e). In contrast, transcripts using exon 14 as terminal exon use as well exon 11, but omit exon 12 and 13 (Fig. 8e) and the RNA sequencing data suggest only moderate induction of such transcripts (Fig. 8c, d). In summary, these data suggest that expression of transcripts starting with the initial exons 1 and 3 and end with exon 13 as a terminal, such as transcript 3, are induced by neuronal-activity. Data derived from primary cultured hippocampal neurons in which controls were compared with neurons 3 h after potassium-induced depolarization corroborate our observations and show induction of transcripts starting with exons1 and 3 and terminating with exon 13 (Additional file 17).

FIRMA identified exon 9 of Dclk1 (doublecortin-like kinase 1) as specifically induced by neuronal activity (Table 2). The large number of Dclk1 splice variants suggests another example of complex activity-regulated alternative splicing. Our microarray analysis identified activity-regulated induction of exon 9 (Additional file 18a). Only two known transcripts comprise exon 9 suggesting that these transcripts are activity-regulated. Analyzes of RNA sequencing data sets of murine hippocampi after seizures and of dissociated primary neuronal cultures in which neuronal activity was provoked by potassium-induced depolarization demonstrate also activity-regulated induction of exon 9 and of exon 7 and 8 (Additional file 18b–d). This strongly suggests activity-regulated expression of the short transcript harboring exon 7–9. In addition, Sashimi plots suggest activity-regulated splicing of additional exons.

Taken together, our validation experiments confirm the exon usage induced by neuronal activity identified by two bioinformatics methods and predict a number of activity-regulated alternatively spliced transcripts.

## Discussion

Here, we identified increased expression levels of exons after chemically induced seizures in the murine hippocampus. To this end, we utilized exon-specific microarrays, observed low variance between biological replicates and analyzed the obtained data by different bioinformatics methods. To validate these findings we reproduced our results in additional cohorts of mice using RNA sequencing, RT-PCR and in situ hybridization. By applying these methods, we identified several activity-regulated splice variants that were otherwise not detected on a global gene level.

We used two different bioinformatics methods, ANOSVA and FIRMA, to identify exons induced by neuronal-activity. ANOSVA, which uses only treatment and probesets as factors at a single time point, was here adjusted to incorporate data from multiple time points and named time-dependent ANOSVA. The ANOSVA method supplies one minimal p value on the gene level and individual analyses have to determine which probesets caused a significant interaction term. In contrast, the FIRMA method already identifies differentially used exons by scoring probesets instead of whole genes. Both methods identified different sets of genes and of the 10 most significant genes identified by FIRMA 3 were also identified as highly significant by ANOSVA.

All genes tested in our validation experiments demonstrated activity-regulated exon usage. Analysis of an additional RNA sequencing data set derived from primary cultured hippocampal neurons under control conditions and 3 h after potassium-induced depolarization [22] gave comparable results for the genes induced 1 h after seizure onset. This suggests that the observed activity-regulated splicing events can be induced by different experimental paradigms of neuronal activity. Moreover, it underscores the high quality of the presented exon array and RNA sequencing data sets, which provide a rich resource for future comparative expression analysis, for the dissection of enhancers and promoters that drive activity-regulated expression and for selecting activity-regulated splice isoforms to be used in functional studies.

Some of the identified activity-regulated, alternatively spliced transcripts, such as Homer1, BDNF, Erffi1 and Inhba, are canonical activity-regulated transcripts [23–31]. For these and the here recognized Rcan1, Tpm1 and Dclk1 translation of the encoded proteins has been demonstrated [32–40]. Differences in exon usage after neuronal activity has been described before for BDNF and Homer1. BDNF is an example of activity-dependent differential usage of 5′-exons harboring untranslated sequence. Such alternatively used 5′-exons suggest differential promotor usage. We demonstrate for Rcan1 and Errfi1 activity-dependent splicing of mutual exclusive 5′-exons and for Inhba differential usage of two 5′-exons. The different splice variants of Errfi1 and Inhba each respectively comprise identical open reading frames. This suggests a pure regulatory function on the transcript level. Neuronal activity-regulated induction of Errfi1, a negative regulator of the EGFR-MAPK signaling pathway, depends on the transcription factor serum response factor (SRF), which is itself a neuronal activity-regulated gene known to stimulate immediate early gene expression (Additional file 1) [34, 41, 42]. This indicates that SRF specifically induces expression of an Errfi1 transcript starting with exon 2.

In contrast to Errfi1 and Inhba, the Rcan1 variants use alternative transcriptional start sites and encode alternative N-termini. Rcan1 is an inhibitor of calcineurin-dependent transcriptional response and critical for synaptic plasticity and formation of memory [37]. In Rcan1, exon 1–3 are mutually exclusively used 5′-exons. Exon 3 corresponds to the most significantly activity-dependent probeset identified by FIRMA and we demonstrate that variant 2 which includes exon 3 is induced by neuronal activity. It has been reported that the Rcan1 variant 1, which includes the second exon, is more abundant and higher expressed in the brain than the variant 2 [36]. This is in agreement with our study. Accordingly, it has been suggested that alternative promoters control expression of both variants [36]. Moreover, overexpression of variant 1 in mice promotes Alzheimer’s disease related pathology in the brain and Down syndrome-like hippocampal deficits [43, 44] and only variant 1 is found chronically overexpressed in brains of Down syndrome and Alzheimer’s disease patients [36]. Therefore, it has been hypothesized that increased expression levels of variant 1 are part of a maladaptive response resulting in disease conditions whereas this is not the case for variant 2 [36].

Activity-regulated expression of the cytokeratin Krt75 and the cytidine deaminase Cda are characterized by the exclusion of two or more 5′-exons and predicted alternative transcriptional start site usage. Krt75 was the most significantly spliced gene identified by time-dependent ANOSVA. Neuronal expression of Krt75 was not reported before. We observed moderate expression in embryonic forebrain, but no expression in the adult brain. The activity-regulated variant is highly specific expressed in the dentate gyrus 4 and 8 h after seizure onset. In agreement with a previous report [21], we observed Krt75 expression in different embryonic epithelia and skin. On the cellular level, Krt75 has been associated with the stabilization of cytoskeletal architecture [21]. The identification of an activity-regulated gene encoding a cytoskeletal protein mainly expressed in epithelia such as skin was unexpected. Interestingly, the activity-regulated gene Arg3.1/Arc which was first detected only in the nervous system and testis [16, 17] has been recently reported to be expressed in skin, specifically in skin-migratory dendritic cells and similarities between the cytoskeletal organization of neurons and skin-migratory cells have been suggested [45].

In contrast to the examples of alternative exon usage at the 5′-end, the complex activity-regulated splicing events of Tpm1, Dclk1 and Homer1 are characterized by the differential usage of internal exons. Tpm1 was identified by time-dependent ANOSVA and specific exons are up- or downregulated after neuronal activity. For Tpm1 a high number of alternative splice variants are known and differently expressed exons are not specific for only one variant. Therefore, it is difficult to predict from exon array data and short RNA sequencing reads which splice variant is differentially expressed after neuronal activity. However, we present evidence that transcript 3 is upregulated after neuronal activity.

Using FIRMA we identified exon 9 of Dclk1 as activity-regulated. This exon is used as an internal exon, but in the activity-regulated transcript as a terminal exon. A large number of Dclk1 splice variants regulated by alternative promotor usage have been described [38, 46]. Only two transcripts comprise exon 9, and transcription of both is induced in the hippocampus by BDNF treatment [39] and by seizures [47, 48] supporting the notion that different plasticity inducing stimuli trigger expression of the transcripts identified in this study. In agreement, Dclk1 has been suggested to enhance dendritic remodeling [49].

The activity-dependent switch of an internal exon to a terminal exon is reminiscent of Homer1. We identify exon 6 and 7 of Homer1 as activity-regulated and both exons present different induction kinetics. Moreover, our data demonstrate that both exons are used as terminal exons in the respective transcripts. The usage of these exons as terminal exons is specific for Homer1a and Ania1, respectively, and their activity-regulated expression has been extensively described before [23–25, 27, 29]. Our study reveals in addition and to our knowledge for the first time, a brief induction of exon 2 of Homer1, which is specific for Homer1d. Exon 2 is used in this variant as the initial 5′-exon suggesting alternative promoter usage. Of note, we observed an induction of Homer1d after seizures and after potassium depolarization in primary cultured neurons. The usage of exon 2 as 5′-exon results in an alternative N-terminus that probably modulates Homer1 action. So far, the specific function of the Homer1d protein has been poorly studied, but one report relates Homer1d to the trafficking of metabolic glutamate receptors [32]. Hence, Homer1d could transiently modify postsynaptic metabolic glutamate receptor localization or anchorage.

Homer1a is thought to modulate postsynapses in response to neuronal activity by giving rise to a dominant-negative isoform that functionally blocks or interferes with the preexisting longer and constitutively expressed variant [33]. Interestingly, several activity-induced splice variants present themselves shorter than constitutively expressed variants. Therefore, it is tempting to speculate that an activity-dependent functional inhibition of preexisting proteins by the activity-induced expression of shorter dominant-negative variants is a general mechanism to alter activity-dependent synaptic structures and functions.

We observed an inverse correlation of Inhba upregulation and the downregulation of a lncRNA. This reverse transcribed lncRNA with an intergenic and intronic part, but no overlap with the Inhba transcript was downregulated by neuronal activity. LncRNAs have been suggested as nuclear factors that organize nuclear sub-structures, modulate chromatin state, and regulate gene expression [50]. The complementary expression of Inhba and of its intronic lncRNA leads to the speculation that the lncRNA is part of a transcriptional repressor complex targeting Inhba expression.

In summary, we have identified a number of differentially expressed exons induced by neuronal activity. This induction corresponds to activity-dependent alternative splicing events that cause a transient shift in splice variants. These are likely to contribute to neuronal plasticity during development and learning processes. However, functional experiments will be needed to unveil the relevance of activity-dependent alternative splicing events. Although the physiological significance of most activity-dependent alternative splicing events is still enigmatic, dysregulation of this process may have a critical impact on neuronal plasticity-related functions and neuronal diseases.

## Methods

### Tissue preparation

3 months old male C57/bl6 mice were housed with a 12 h dark–light-schedule and experiments were performed during the dark cycle always at identical time points. Kainic acid (Ascent scientific) (20 mg/kg, dissolved in PBS) or similar amounts of PBS were administered by intraperitoneal injection. Within approximately 30 min kainic acid administered mice developed seizures. Mice were monitored throughout the experiment and seizures were scored and documented according to a modified Racine scale, a behavioral scaling (Additional file 19). Only tissue from animals reaching stage 5 (continuous rearing and falling) were used in subsequent experiments. The first rearing was defined as the starting point of the seizure. Animals were sacrificed at given time points after onset of the seizure as described before [29].

### Microarray hybridization

For microarray experiments, we sacrificed four mice under control conditions and three time-matched replicates for vehicle and kainic acid treatments, respectively, as these numbers resulted in our previous studies [29, 30] in sufficient statistical power. For RNA isolation hippocampi were dissected from fresh brains, flash frozen and stored at − 80 °C. Total RNA was isolated using TRItidy-reagent (Applichem), followed by an additional purification step using RNeasy columns (Qiagen), quantified by UV-spectroscopy and its quality verified using a LabChip BioAnalyzer (AGILENT Technologies). The amplification and labeling of RNA samples were conducted according to the manufacturer's instructions (Affymetrix). One µg from each sample was transcribed to cDNA using an oligo(dT)24 primer containing a T7 RNA polymerase promoter. After RNAse H-mediated second strand cDNA synthesis, the product was purified and served as a template in the subsequent in vitro transcription reaction. Biotin-labelled cRNA was prepared from double-stranded cDNA by in vitro transcription using the GeneChip RNA transcript labelling kit (Affymetrix). After clean-up, biotin-labelled cRNA was fragmented by alkaline treatment [40 mmol/l Tris–acetate (pH 8.2), 100 mmol/l potassium acetate, and 50 mmol/l magnesium acetate] at 94 °C for 35 min. 15 µg of each cRNA sample was hybridized for 16 h at 45 °C to an Affymetrix Mouse Exon Array 1.0 ST GeneChip covering the complete transcribed mouse genome. Chips were washed and stained with streptavidin–phycoerythrin using a fluidics station according to the protocols recommended by the manufacturer. Finally, arrays were scanned at 1.56-µm resolution using the Affymetrix GeneChip System confocal scanner 3000. RNA extracted from one hippocampus was hybridized to one microarray, and we measured four replicate animals for the untreated controls and three time matched replicates for vehicle and kainic acid treatments (Fig. 1a).

### Microarray data analysis

Data from GeneChip microarrays has been deposited in the NCBI Gene Expression Omnibus (GEO) and is accessible through the GEO Series accession number GSE88723. Raw data analysis was performed in R version 3.6 (https://www.bioconductor.org) using the bioconductor packages “oligo” [51] and “aroma.affymetrix” [52]. The bulk of the preprocessing was performed by using Robust Multichip Average procedure, which includes background correction, normalization and summarization to both transcript cluster level and probe set level for the investigation of alternative splicing. Affymetrix databases (Release 32) were used for annotation and probe sets without annotation were removed by choosing the annotation level "core", which is supported by consolidated evidence such as RefSeq transcripts and full-length mRNAs [53].

For the transcript level analysis only transcripts with normalized expression values above the median were retained for further analysis. To investigate sample-wise variability between different conditions as well as between samples of the same condition, pairwise correlation between all samples were calculated using the Pearson distance metric. For this, the 1000 genes with the highest standard deviation over all samples were extracted from the set of present gene expression values. The resulting correlation matrix was plotted using the pheatmap function from the ggplot2 package in R (https://ggplot2.org) [54].

We used the R package limma to assign significance to the regulation of genes over time, and identified differentially expressed genes for each time point (1, 4 and 8 h) individually. For the samples treated with KA or vehicle corresponding to each time point, we fit a linear model to each transcript and computed moderated t-statistics and corresponding p values by empirical Bayes moderation using the functions lmFit and eBayes [55]. The p values were adjusted after the Benjamini and Hochberg method. A principal component analysis (PCA) was conducted using the “prcomp” function in R specifying the different samples as individual factors.

### Identification of alternative splicing events

The ANalysis Of Splice VAriation, ANOSVA, uses a two-way ANOVA, where each observation is modeled by the combination of the effect of the probesets and the effect of the treatment and the significance of each interaction term is assessed using a t test [20]. To identify genes with alternative splicing events, the significance level for every interaction term is calculated and the minimal p value interpreted as a measure of confidence that the gene exhibits differential splicing across different conditions [53]. In order to better monitor splicing changes over time, we adjusted this method to include a time factor δ to represent the hour *h*, at which the mice were sacrificed, such that *y*_*peth*_, representing the observed log intensity measure per probe *p* in probeset *e* under treatment *t* at time point *h* is modeled as:

1 $$y_{{{\text{peth}}}} = \mu + \alpha_{e} + \beta_{t} + \delta_{h} + \gamma_{{{\text{eth}}}} + \varepsilon_{{{\text{peth}}}} .$$

The interaction term γ_eth_ now represents the effect of every combination of the 3 factors probe set α_e_, treatment β_t_ and time point δ_h_. This time-dependent ANOSVA was implemented by adjusting the workflow proposed by Rodrigo-Domingo et al. [53], which uses the package “aroma.affymetrix”, as it allows for preprocessing of the probe intensities without summarizing them to probeset or transcript level. Before performing the time-dependent ANOSVA, the probe intensity data was filtered to include only probe sets that were not cross-hybridizing and present in at least half of the samples of one or more treatment groups. Only probe intensities corresponding to transcripts with half or more probe sets present in at least half of the samples of a treatment group were considered for further analysis. Furthermore, transcripts with less than 3 probesets were removed. The probe intensity data was divided into data frames per transcript/gene and organized in a list, which was then used to apply the time-dependent ANOSVA procedure to each transcript/data frame. The minimal p value for the interaction term γ_eth_ was reported for every transcript and adjusted using the Benjamini—Hochberg, to account for multiple testing. The adjusted p values were used as a measure for significant splicing and a false discovery rate of 0.05 was set as cut-off. Because of the high inter-sample similarity of the control samples (untreated and treated with vehicle) detected during quality control, the expression values of these samples were all handled as the same treatment at time point 0, instead of splitting them into 4 different groups, as this resulted in over-fitted data and significant identification of noisy genes.

In addition to the time-dependent ANOSVA, the original ANOSVA method was applied to all time points separately, but as all genes identified as spliced using the simple ANOSVA were also identified using time-dependent ANOSVA, we focus on the time-dependent method instead.

The FIRMA method was introduced by Purdom et al. [19] and operates under the assumption that skipping or inclusion of single exons is the most common type of alternative splicing events. Therefore, FIRMA provides a score for each exon reflecting whether its probes systematically deviate from the expected gene expression level. The estimation of the expression levels of each gene was performed using the Robust Multichip Analysis, which itself involves background correction, normalization and summarization of the probe-level data. This method for detection of alternative splicing was implemented in the “aroma.affymetrix” package [52]. Only probe sets from transcripts with log expression values above the median in at least half of the samples were considered for analysis. This all-sample method, which seeks to pinpoint exons with alternative splicing without determining in which samples, scores each probe set by finding the minimum F score in each of the condition groups and then chooses the maximum out of these. This procedure ensures that a highly scored probe set has uniformly high F scores in all of the samples of at least one of the condition groups.

To establish a cut-off and a level of significance for the F score, all the scores from samples treated with vehicle and the control samples without treatment, were assigned to the background distribution *F*^*0*^ representing no activity induced alternative splicing. The scores corresponding to samples treated with kainic acid and the control samples were assigned to the sample distribution *F*^*S*^, which represents the alternative hypothesis that differences in splicing between the treatments and time points after seizure onset exist. For each distribution, the minima per condition group (control, 1 h, 4 h and 8 h) were determined and the maximum chosen for every probe set. This resulted in two sets of overall scores, one for the background distribution and one for the sample distribution, which were both arranged by decreasing order of the sample distributions all-sample scores. First the cumulative probability *p*^*S*^, defined as the frequency by which the scores *F*^*S*^ are larger than or equal to the observed value *F*^*S*^_*e*_ corresponding to probe set *e* in the sample distribution and divided by the number of total data points, was calculated for every score of the sample distribution.

2 $$p^{S}_{e} = \, P\left( {F^{s} \ge \, F^{s}_{e} } \right)$$

Then, for every probe set *e* the cumulative probability *p*^*0*^_*e*_ of the null distribution was calculated as the number of scores from the null distribution, which are larger than or equal to the score *F*^*s*^_*e*_ from the sample distribution corresponding to probe set *e* of the observed value *F*^*0*^_*e*_, divided by the total number of observations.

3 $$p^{0}_{e} = \, P\left( {F^{0} \ge \, F^{0}_{e} } \right)$$

The false discovery rate (FDR) for every probe set was further defined as

4 $${\text{FDR}}_{{\text{e}}} = p^{0}_{e} /_{{}} p^{S}_{e} .$$

A cut-off of 10% FDR was chosen as a threshold for highly significant differential splicing.

### RNA sequencing and data analysis

For RNA isolation hippocampi were dissected from fresh brains, flash frozen and stored at − 80 °C. Total RNA was isolated using TRItidy-reagent (Applichem), followed by an additional purification step using RNeasy columns (Qiagen), quantified by UV-spectroscopy and its quality verified using a LabChip BioAnalyzer (AGILENT Technologies). Sequencing libraries were prepared from total RNA with TruSeq stranded total RNA library preparation kit (Illumina). 125-bp paired end reads were generated with a HiSeq 2500 sequencer (Illumina). Readmapping was performed using STAR aligner [56], with the following options: –outFilterType BySJout, –outFilterMultimapNmax 20, –alignSJoverhangMin 8, –alignSJDBoverhangMin 1, –alignIntronMin 20, –alignIntronMax 1000000, –alignMatesGapMax 1000000, –outSAMtype BAM SortedByCoordinate. Resulting BAM files were visualized and Sashimi plots generated through Integrative Genomics Viewer (IGV) [57, 58].

RNA sequencing data has been deposited in the NCBI Gene Expression Omnibus (GEO) and is accessible through the GEO Series accession number: GSE148028.

In addition to our own RNA-sequencing data, we analyzed RNA-sequencing data derived from primary cultured hippocampal neurons under control conditions and 3 h after potassium-induced depolarization [22] deposited in the GEO database under: GSE89984.

Neuronal activity induced alternative splicing was analyzed in both datasets and all genes tested in our validation experiments demonstrated activity-regulated exon usage after kainic induced seizures. Only genes induced already 1 h after seizure onset, namely Rcan1, Errfi1, Homer1, Tpm1 and Dclk1, demonstrated a comparable induction 3 h after potassium-induced depolarization.

### PCR analyses

Total RNA was isolated using TRItidy-reagent (Applichem), followed by an additional purification step using RNeasy columns (Qiagen). One µg from each sample was transcribed to cDNA using a random-hexa primer and SuperScriptII Reverse Transcriptase (Thermofisher). The relative abundance of alternative spliced variants was then analysed by PCR using exon spanning primers and products were visualized by ethidium bromide staining after agarose gel-electrophoresis. Indicated PCR products were sequenced. The table in Additional file 6 lists all used exon spanning primer pairs. Additional file 7 summarizes all RT-PCR products, their sizes and indicates which figures show respective gels.

### In situ hybridization

For in situ hybridization, animals were sacrificed by cervical luxation at indicated time points after onset of the first seizure (each time point, n = 3), control animals were sacrificed 30 min after vehicle injection plus indicated time points (n = 3). Whole brains were flash frozen using liquid nitrogen and stored at − 80 °C until cryosectioning. In situ hybridization was essentially performed as described before [59]. In brief, antisense RNA probes labelled with [α-^35^S]-UTP were generated according to the manufacturer’s instructions (Promega). 20 µm cryosections of embryos or brains were fixed in 4% paraformaldehyde-PBS, acetylated, dehydrated and hybridized at 55 °C for 18 h. Ribonuclease A treatment was performed for 30 min at 37 °C. Following a high stringency wash in 0.1 × saline sodium citrate buffer at 55 °C, slides were exposed to X-ray films (Kodak Biomax MR; Amersham Bioscience) for 72 h. Specificity of signals was verified by comparing antisense with sense controls. Each gene was analysed at least on sections of three different animals of one experimental group. To generate Krt75 probes for in situ hybridization, we cloned Krt75 from mouse skin cDNA (a generous gift from Dr. Johanna Brandner). We cloned the canonical Krt75 cDNA (corresponding to GenBank accession number NM_133357) and with the identical primers a so far undescribed shorter splice variant (GenBank accession number MN037882). Moreover, we cloned from murine hippocampus 4 h after seizure onset a shorter variant (GenBank accession number MN124092). Krt75 expression was detected with 3 different probes (Fig. 3a). The 5′ probe was cloned from the full length plasmid using a NotI site of the multiple cloning site of the vector and an internal SalI site, the fragment corresponds to nucl. 71-897 of NM_133357. The internal probe and the 3′ probe were cloned from full length cDNA by PCR using sequence specific primers (5′-AAAGGATCCAGGTGAGTGAC and 5′-AAAGAATTCTCCCTCTCCGC for the internal probe and 5′-AAACTGCAGTCACT TCTACGG and 5′-AAACTCGAGTGCTTGTAACTCTTTC for the 3′ probe). The resulting products of 508 bp (corresponding to nucl. 969–1476 of NM_133357) and 237 bp (corresponding to nucl. 1495–1731 of NM_133357) respectively were cloned into pBSK and linearized for antisense transcription. RCAN1 variant 1 and variant 2 specific probes correspond to exon 1 and exon 2 respectively and were cloned from full length cDNA clones (Source Bioscience, UK) by PCR using sequence specific primers (5′-AAAGGATCCGTAGGGTGACTCTGCGGC and 5′-AAACTCGAGCAGGCCGTCCACGAACAC for variant 1 and 5′-AAAGGATCCTGCAAAGGAACCTCCAGCTTG and 5′-AAACTCGAGCCTGGTCTCACTTTCGCTGA for variant 2). The resulting products of 173 bp (corresponding to nucl. 159–331 of reference sequence NM_001081549) and 163 bp (corresponding to nucl. 21–184 of reference sequence NM_019466) respectively were cloned into pBSK and linearized with BamHI for antisense transcription. All templates were validated by sequencing.

## Supplementary information

**Additional file 1: **Significantly activity regulated genes identified on the whole gene level. pdf. Transcript Cluster ID according to the Affymetrix annotation system (NetAffx) and the corresponding gene symbol of identified activity regulated genes are listed. Log2 ratios at time point 1, 4, and 8 h are given as well as the cluster classification, visualized in Additional file 2.

**Additional file 2: **Temporal cluster analysis of activity-regulated genes. pdf. Activity-regulated genes identified on the whole gene level were assigned to clusters based on their differential regulation over four time points (0, 1, 4, 8 h after seizure onset). Similar expression kinetics were grouped and the thick black line represents mean expression profiles.

**Additional file 3: **Genes with activity-dependent splice variation identified through time-dependent ANOSVA. pdf. pValueBH, minimal adjusted p-value derived by Bonferoni and Holm method. DE, differentially expressed, indicates whether the whole gene was identified as induced by neuronal activity in our initial analysis (Additional file 1).

**Additional file 4: **Genes with activity-dependent exon usage identified through the FIRMA method. pdf. FIRMA (F) scores represent the discrepancy of one probeset from the other probesets in the same gene and were calculated for every probeset and sample. fSscore, corresponds to samples collected after seizure compared to controls and samples. f0 score, corresponds to samples collected after vehicle treatment compared to controls. FDR, False discovery Rate calculated from F scores for the alternative and the null model. DE, differentially expressed, indicates whether the whole gene was identified as induced by neuronal activity in our initial analysis (Additional file 1).

**Additional file 5: **Gene expression profile of Krt75. pdf. Analysis and visualization of the microarray data obtained for Krt75. The upper 3 plots show the log2 transformed expression values of Krt75 over the course of its probes and probesets per exon. While the red lines show the intensity values of the control samples (vehicle and untreated), the 3 shades of green represent the expression intensities in the samples at the three different time points after seizures. The upper plot shows raw probe level intensities and grey bins stand for the individual probes. The middle plot shows the normalized intensities summarized to probeset level (probes divided by grey bins) for every sample. The lower plot shows the log2 ratio calculated from the mean of the intensities seen in the second plot, each line represents one of the 3 time points after seizure. At the bottom, 3 types of exon models are depicted. The dark blue boxes represent the probesets per exons, which were included in the microarrays. The yellow boxes and track correspond to genomic sequence and each box indicates an exon. The light blue tracks and boxes represent the only so far annotated transcript present in the Ensembl database. Note that exons 5 and 8 are not represented by probesets.

**Additional file 6: **Exon spanning primers. pdf

**Additional file 7: **Products of RT-PCR. pdf

**Additional file 8: **Alternative splice variants of Krt75. pdf. **a** Exons of murine Krt75 according to the Ensembl database. Boxes represent exons (not to scale), black boxes correspond to coding sequence, white boxes to untranslated regions. Numbers indicate exon size (upper row) and intron size (lower row). A corresponding cDNA was cloned from skin in this study (Genebank accession number NM_133357). **b** An alternatively spliced and previously undescribed cDNA was cloned in this study from skin (Genebank accession number MN037882). The internal alternative splice site (arrowhead) in exon 7 is indicated. The usage of this alternative splice site results in a frameshift and premature stop in the sequence corresponding to exon 8. **c** A truncated splice variant was cloned in this study from hippocampus of mice 4 h after seizure onset (Genebank accession number MN124092). It starts with exon 4. **d** Schematic of the protein domain structure of Krt75 corresponding to the canonical variant depicted in **a**. 1A, 1B, 2A and 2B are coiled-coil subdomains and L1, L12 and L2 are intervening linkers. The orange box indicates 10–20 amino acid segments that are highly conserved among keratin and other interfilament proteins. **e** Schematic of the protein domain structure of Krt75 corresponding to the new splice variant from skin depicted in **b**. **f** Schematic of the protein domain structure of Krt75 corresponding to the activity-regulated splice variant expressed in the hippocampus depicted in **c**.

**Additional file 9: **Gene expression profile of Rcan1. pdf. Analysis and visualization of the microarray data obtained for Rcan1. The upper 3 plots show the log2 transformed expression values of Rcan1 over the course of its probes and probesets per exon. While the red lines show the intensity values of the control samples (vehicle and untreated), the 3 shades of green represent the expression intensities in the samples at the three different time points after seizures. The upper plot shows raw probe level intensities and grey bins stand for the individual probes. The middle plot shows the normalized intensities summarized to probeset level (probes divided by grey bins) for every sample. The lower plot shows the log2 ratio calculated from the mean of the intensities seen in the second plot, each line represents one of the 3 time points after seizure. At the bottom 3 types of exon models are depicted. The dark blue boxes represent the probesets per exons, which were included in the microarray analysis. The yellow boxes and track correspond to genomic sequence and each box indicates an exon. The light blue tracks and boxes represent the annotated transcripts present in the Ensembl database.

**Additional file 10: **Activity-dependent splicing of Rcan1 in primary cultured hippocampal neurons. pdf. **a** Coverage plots (blue) from paired-end reads of RNA from untreated primary hippocampal neurons (upper panel) and 3 h after KCl induced depolarization (lower panel). The reference gene track is depicted below (green). **b** Sashimi plot of RNA sequencing data from untreated primary neurons (upper panel) or 3 h after KCl treatment (lower panel). Numbers indicate the counts of RNA sequencing reads that span the respective exon junctions. RNA-seq data was generated by Quesnel et al. and deposited in the GEO database under ID code GEO: GSE89984.

**Additional file 11: **Gene expression profile of Cda and Errfi1. pdf. Analysis and visualization of the microarray data obtained for **a** Cda and **b** Errfi1. The upper 3 plots show the log2 transformed expression values over the course of its probes and probesets per exon. While the red lines show the intensity values of the control samples (vehicle and untreated), the 3 shades of green represent the expression intensities in the samples at the three different time points after seizures. The upper plot shows raw probe level intensities and grey bins stand for the individual probes. The middle plot shows the normalized intensities summarized to probeset level (probes divided by grey bins) for every sample. The lower plot shows the log2 ratio calculated from the mean of the intensities seen in the second plot, each line represents one of the 3 time points after seizure. At the bottom 3 types of exon models are depicted. The dark blue boxes represent the probesets per exons, which were included in the microarray analysis. The yellow boxes and track correspond to genomic sequence and each box indicates an exon. The light blue tracks and boxes represent the annotated transcripts present in the Ensembl database.

**Additional file 12: **Activity-dependent splicing of Errfi1 in primary cultured hippocampal neurons. pdf. **a** Coverage plots (blue) from paired-end reads of RNA from untreated primary hippocampal neurons (upper panel) and 3 h after KCl induced depolarization (lower panel). The reference gene track of Errfi1 is depicted below (green). **c** Sashimi plot of RNA sequencing data from the untreated primary neurons (upper panel) or 3 h after KCl treatment (lower panel). Numbers indicate the counts of RNA sequencing reads that span the respective exon junctions. RNA-seq data was generated by Quesnel et al. and deposited in the GEO database under ID code GEO: GSE89984.

**Additional file 13: **Gene expression profile of Inhba. pdf. Analysis and visualization of the microarray data obtained for Inhba. The upper 3 plots show the log2 transformed expression values of Inhba over the course of its probes and probesets per exons. While the red lines show the intensity values of the control samples (vehicle and untreated), the 3 shades of green represent the expression intensities in the samples at the three different time points after seizures. The upper plot shows raw probe level intensities and grey bins stand for the individual probes. The middle plot shows the normalized intensities summarized to probeset level (probes divided by grey bins) for every sample. The lower plot shows the log2 ratio calculated from the mean of the intensities seen in the second plot, each line represents one of the 3 time points after seizure. At the bottom 3 types of exon models are depicted. The dark blue boxes represent the probesets per exons, which were included in the microarray analysis. The yellow boxes and track correspond to genomic sequence and each box indicates an exon. The light blue tracks and boxes represent the only so far annotated transcript present in the Ensembl database.

**Additional file 14: **Gene expression profile of Homer1. pdf. Analysis and visualization of the microarray data obtained for Homer1. The upper 3 plots show the log2 transformed expression values of Homer1 over the course of its probes and probesets per exon. While the red lines show the intensity values of the control samples (vehicle and untreated), the 3 shades of green represent the expression intensities in the samples at the three different time points after seizures (compare color code at the bottom). The upper plot shows raw probe level intensities and grey bins stand for the individual probes. The middle plot shows the normalized intensities summarized to probeset level (probes divided by grey bins) for every sample. The lower plot shows the log2 ratio calculated from the mean of the intensities seen in the second plot, each line represents one of the 3 time points after seizure. At the bottom 3 types of exon models are depicted. The blue boxes represent the probesets per exons, which were included in the microarray experiments. The yellow boxes and track correspond to genomic sequence and each box indicates an exon. The light blue tracks and boxes represent transcripts as annotated in the Ensembl database. The blue lines below the first and third intensity plot show which probesets map to which exon.

**Additional file 15: **Activity-dependent splicing of Homer1 in primary cultured hippocampal neurons. pdf. **a** Homer1 expression in primary cultured hippocampal neurons of control mice or 3 h after KCl treatment detected by RNA sequencing. Shown are coverage plots (blue) from paired-end reads for the two sample groups. The reference gene track is depicted below (green). **b** Sashimi plot of RNA sequencing data for Homer1. Numbers indicate the counts of RNA sequencing reads that span the respective exon junctions. RNA-seq data was generated by Quesnel et al. and deposited in the GEO database under ID code GEO: GSE89984.

**Additional file 16: **Gene expression profile of Tpm1. pdf. Analysis and visualization of the microarray data obtained for Tpm1. The upper 3 plots show the log2 transformed expression values of Tpm1 over the course of its probes and probesets per exon. While the red lines show the intensity values of the control samples (vehicle and untreated), the 3 shades of green represent the expression intensities in the samples at the three different time points after seizures. The upper plot shows raw probe level intensities and grey bins stand for the individual probes. The middle plot shows the normalized intensities summarized to probeset level (probes divided by grey bins) for every sample. The lower plot shows the log2 ratio calculated from the mean of the intensities seen in the second plot, each line represents one of the 3 time points after seizure. At the bottom 3 types of exon models are depicted. The dark blue boxes represent the probesets per exons, which were included in the microarray analysis. The yellow boxes and track correspond to genomic sequence and each box indicates an exon. The light blue tracks and boxes represent the only so far annotated transcript present in the Ensembl database.

**Additional file 17: **Activity-dependent splicing of Tpm1 in primary cultured hippocampal neurons. pdf. **a** Coverage plots (blue) from paired-end reads of RNA from untreated primary hippocampal neurons (upper panel) and 3 h after KCl induced depolarization (lower panel). The reference gene track is depicted below (green). **b** Sashimi plot of RNA sequencing data from untreated primary neurons (upper panel) or 3 h after KCl treatment (lower panel). Numbers indicate the counts of RNA sequencing reads that span the respective exon junctions. RNA-seq data was generated by Quesnel et al. and deposited in the GEO database under ID code GEO: GSE89984.

**Additional file 18: **Gene expression profile of Dclk1. pdf. **a** Analysis and visualization of the microarray data obtained for Dclk1. The plots and color code are in correspondence to previous figures. The red dotted circle marks the activity-regulated exon 9. Black dotted lines indicate area between exon 6 and 19 that is magnified in B-D. **b** Validation of Dclk1 variant expression in hippocampus of control mice or 1, 4 or 8 h after seizure onset by RNA sequencing. Shown are coverage plots (blue) from paired-end reads for the four sample groups. The reference gene track of Dclk1 is depicted below (black). **c** Sashimi plot of RNA sequencing data for Dclk1. Numbers indicate the counts of RNA sequencing reads that span the respective exon junctions. **d** Coverage plots (blue) from paired-end reads of RNA from untreated primary hippocampal neurons (upper panel) and 3 h after KCl induced depolarization (lower panel). The reference gene track is depicted below (black). **e** Sashimi plot of RNA sequencing data from the untreated primary neurons (upper panel) or 3 h after KCl treatment (lower panel). Numbers indicate the counts of RNA sequencing reads that span the respective exon junctions. RNA-seq data was generated by Quesnel et al. and deposited in the GEO database under ID code GEO: GSE89984.

**Additional file 19: **Modified Racine scale (behavioral scoring in response to kainic acid). pdf

## Data Availability

GeneChip micorarray and RNA sequencing data has been deposited in the GEO database under GSE88723 and GSE148028. The nucleotide sequences reported here for Krt75 have been submitted to GeneBank with accession number MN037882 and MN124092. Additional data supporting the conclusions of this article is included within the article and its additional files.
