# Supplementary material for: Neuronal activity regulates alternative exon usage
Source: Mol Brain. 2020 Nov 10;13:148. doi: 10.1186/s13041-020-00685-3 (PMC7656758; doi:10.1186/s13041-020-00685-3)
Supplement: Supplementary file 1 — Additional file 1: Significantly activity regulated genes identified on the whole gene level. pdf. Transcript Cluster ID according to the Affymetrix annotation system (NetAffx) and the corresponding gene symbol of identified activity regulated genes are listed. Log2 ratios at time point 1, 4, and 8 h are given as well as the cluster classification, visualized in Additional file 2. [file 13041_2020_685_MOESM1_ESM.pdf]

Significantly activity regulated genes identified on the whole gene level

TranscriptClusterID	geneSymbol	logFC.1h	logFC.4h	logFC.8h	Cluster
6749557	Coq10b	1.42169837809623	0.493482916886033	0.495607556717703	UpUpUp
6753917	Ptgs2	3.74228577640743	2.35806775980061	1.35961210106644	UpUpUp
6759997	Scg2	1.06804391409659	1.14696604980176	1.04266948831374	UpUpUp
6760680	Per2	0.482300150168029	0.823698629335045	0.555308286515178	UpUpUp
6762784	Rgs2	2.20535376392748	1.37233073613769	1.29163579973172	UpUpUp
6763207	Ier5	0.854278654993525	0.786277075381337	0.986960511641014	UpUpUp
6763991	Rgs4	1.88201896668918	1.28589139500837	1.15291628256798	UpUpUp
6764040	Fcgr3	0.350383082206325	0.602909186599103	0.476491287672506	UpUpUp
6791451	Stat3	0.507943048183926	0.737937093805082	0.718805743479826	UpUpUp
6796121	Rhoj	0.577144325167499	1.47506228561493	1.10846101957003	UpUpUp
6808997	Homer1	1.7294219511319	1.29946898042309	0.988784464114076	UpUpUp
6812375	Nrn1	0.599329996748417	0.958681243205121	0.772580605375727	UpUpUp
6819907	Stmn4	0.603963293211096	0.663946340719347	0.655910417913513	UpUpUp
6830770	Trib1	1.80878890995383	0.907780856578623	0.893185447383116	UpUpUp
6831994	Atf4	0.581605005047555	0.499286425668115	0.701681649317768	UpUpUp
6833308	Grasp	1.01264189569757	1.26596359169327	1.15749014800945	UpUpUp
6834728	Ankrd33b	0.932126289706684	1.19397779310975	0.711990819478795	UpUpUp
6836973	Myh9	0.36085858260556	0.550003625765077	0.599944532260812	UpUpUp
6845569	Arhgap31	0.251455934838516	0.610727351555083	0.343716085443273	UpUpUp
6849595	Cdkn1a	2.17182603595475	2.21404464984676	1.86219997950169	UpUpUp
6857512	Hnrnpll	0.727818034677116	0.517760528141318	0.436756149275574	UpUpUp
6872878	Ppp1r3c	0.52655948036106	0.740563434690562	0.622220374641635	UpUpUp
6873066	Pdlim1	0.515825628421489	0.676403961312133	0.941230755503913	UpUpUp
6893558	Pmepa1	0.357083586123384	0.90719276466811	1.7241706484431	UpUpUp
6896519	Skil	0.443992600295827	0.853557439610566	1.24843595232238	UpUpUp
6896584	4930429B21Rik	1.06173475488774	0.744947697022431	0.541153923466912	UpUpUp
6902440	Eltd1	0.470328382328572	0.874759057369723	0.74317816402568	UpUpUp
6908919	Usp53	0.497832993088527	0.852404971985453	0.426042515715195	UpUpUp
6909153	Larp7	0.251567831836794	0.581590452082303	0.41933955274197	UpUpUp
6917129	Stk40	1.28801192034713	1.52530349831465	1.60374907546727	UpUpUp
6919320	Penk	0.555637875655527	0.533445142737332	0.660640433132151	UpUpUp
6935451	Nptx2	2.96446924714572	2.49164194312423	2.66245020246823	UpUpUp
6936759	Rheb	0.786834876150751	0.802105825177796	0.788010339700024	UpUpUp
6946119	Npy	0.2408770548215	0.649712217480553	1.11648644187272	UpUpUp
6950413	Emp1	0.987036441530774	1.09987586278476	1.10419162193321	UpUpUp
6958974	Pglyrp1	0.497991990968815	0.902206916194595	1.01074829302168	UpUpUp
6964365	Stx4a	0.415417038143212	0.504159040936233	0.270278789799585	UpUpUp
6964394	Tgfb1i1	0.585076260394712	0.577331592747491	0.631606736802552	UpUpUp
6974831	Prosc	0.828805920671696	0.79367617063422	0.553563087790844	UpUpUp
6977139	Tpm4	0.585506348053934	0.742268173168058	0.590485855888126	UpUpUp
6990526	Arpp19	0.868685909096877	1.00731653103515	0.923127185445681	UpUpUp
6996448	Tpm1	0.367069424369704	0.606384651759922	0.710315563719772	UpUpUp
6996935	Mapk6	0.350004678058262	0.877375355697279	0.953779001667346	UpUpUp
7005797	Hmgcs1	0.51398514723946	0.609165882931209	0.26527480734027	UpUpUp
7019519	Gla	1.0511634930072	0.522838607798437	0.275550778187764	UpUpUp
6766455	Sgk1	1.25464767698923	1.09376910621715	0.220452091391502	UpUpNon
6806435	Gcnt2	1.08514596687437	0.595824361286433	0.137282415117533	UpUpNon
6830761	Sqle	0.584570486241831	0.736844716034895	0.042558085883663	UpUpNon
6915138	Acer2	0.399252772062913	0.725576185426681	-0.0933686639152434	UpUpNon
6925149	Mfsd2a	0.521209903027831	0.599419352048282	0.290994763666161	UpUpNon
6935927	Cyp51	1.01086665895921	0.633180335841413	0.25431106748876	UpUpNon
6966610	Plekhf1	1.13255045038447	0.907133176979458	0.129581589979083	UpUpNon
6978291	Mt1	0.383520173728724	0.534203898599953	0.308465435795879	UpUpNon
6982921	Msmo1	0.922078953917961	0.789406645207111	-0.0845770867674952	UpUpNon
6752884	R3hdm1	0.35062924071	0.519633379674984	0.456826314991221	UpNonUp
6753179	Ppp1r15b	0.811122387108086	0.398179685812138	0.279117517649928	UpNonUp
6754014	Ivns1abp	0.590419474946065	0.428920500662453	0.27328209021732	UpNonUp
6755896	Dusp10	0.506980696073874	-0.0518917092568279	0.43872462899147	UpNonUp
6760006	Wdfy1	0.588667052407463	0.348476839714113	0.272160817469134	UpNonUp
6762132	Mapkapk2	0.356124709619944	0.67700684614554	0.818321007767504	UpNonUp
6762345	Btg2	3.62598076970745	0.608388975074205	0.4307003172754	UpNonUp
6764342	Grem2	0.426923427899614	0.447028639857501	0.527092181116005	UpNonUp
6768094	Ranbp2	1.35251300585187	0.897390670985784	0.399597316727098	UpNonUp
6768450	Jmjd1c	0.604422177646877	0.799119211218455	0.206632121811771	UpNonUp
6769080	Cstb	0.527103592280498	0.422718488482577	0.435363619630266	UpNonUp
6769150	Ptbp1	0.940528723098704	0.361054372106115	0.533145061152793	UpNonUp

6769193	Midn	1.47162566981203	0.675879619464125	0.776550030335268	UpNonUp
6769244	Dot1l	0.765763736523053	0.918107023881027	0.946375401040331	UpNonUp
6769255	Gadd45b	2.79333454916744	2.01385647994827	2.10826007699594	UpNonUp
6775389	Mknk2	0.833814158034124	0.432522231145532	0.286022410976989	UpNonUp
6779185	Spred2	0.359111445890137	0.636628277252158	0.881276629229854	UpNonUp
6784290	Arl4d	2.95413089553577	1.50145468627577	1.11697403623341	UpNonUp
6784412	Fmnl1	0.24206570642645	0.830907583381475	1.15354545673816	UpNonUp
6789229	Ndel1	0.541448563700748	0.273315426962921	0.324333383957264	UpNonUp
6789325	Cd68	0.587541123266272	0.261195340620835	0.57681911320401	UpNonUp
6790317	Dusp14	0.801620467890797	0.315743389167338	0.944320711543378	UpNonUp
6792486	H3f3b	0.740573449188949	0.473512691226615	0.256310275007025	UpNonUp
6795881	Frmd6	1.95456362674778	0.380793583108926	0.353717141583894	UpNonUp
6796691	Fos	4.35552584213965	1.83378629711308	1.57310215499532	UpNonUp
6799239	Kcnf1	1.03935852494267	0.254313827889259	0.420671967783726	UpNonUp
6800233	Ifrd1	2.42777656316412	1.54707049214089	0.800036830623274	UpNonUp
6802727	Dio2	0.631759827331871	0.708715516267224	0.358843952704262	UpNonUp
6804582	Klf6	1.07368767174763	0.115947457804957	0.54317261687998	UpNonUp
6804996	Inhba	2.97907699147859	3.07868050378605	3.62469477439668	UpNonUp
6807041	Gadd45g	3.22337957176186	1.20256838514694	0.71418727278519	UpNonUp
6808279	Pcsk1	1.88650387616958	1.2416349642872	1.14587015756142	UpNonUp
6810166	Plk2	1.58066905159317	1.04048021903786	0.615176506582269	UpNonUp
6812652	Nedd9	0.915865471128231	1.05520953289491	1.18928031687204	UpNonUp
6813327	Cltb	0.411525797154539	0.18685038814222	0.736923321405329	UpNonUp
6819156	Rem2	0.605698034587268	0.589304174833894	0.799672547836014	UpNonUp
6820055	Egr3	2.14854083949641	1.099760163279	1.08154514254664	UpNonUp
6826292	Pcdh8	2.56130240008902	1.82553227402639	1.80023178737453	UpNonUp
6827284	Spry2	1.5293484533209	1.12233612487898	0.813872792278889	UpNonUp
6831511	Bai1	0.289426562337256	0.470413289524476	0.577535909021178	UpNonUp
6833311	Nr4a1	2.84128139596274	1.04782664826532	0.52530910659043	UpNonUp
6834729	Ankrd33b	0.922889592879451	0.781509094887889	0.473068840213978	UpNonUp
6836691	Arc	2.39525198959531	1.59403145722176	1.66906466089845	UpNonUp
6843433	Ets2	0.385554395440538	0.490880390947808	0.615937741666979	UpNonUp
6847556	Adamts1	2.15586941863395	0.384827866140845	0.833095948576637	UpNonUp
6849626	Pim1	1.9465645179292	0.948894039943192	0.861397853238215	UpNonUp
6852144	Lbh	1.02648991707036	0.674922807941531	0.394782701698723	UpNonUp
6854487	Dusp1	2.90585456137702	0.705265292198502	0.720683685192771	UpNonUp
6854844	Sik1	2.1478879102922	1.1202688923685	0.983714226172486	UpNonUp
6855659	Vegfa	0.227019464228212	0.53034048684077	0.715054797124717	UpNonUp
6859972	Egr1	1.32007773532245	0.657651987692718	0.666068597779741	UpNonUp
6860778	Dmxl1	0.500080711734607	0.510258383941068	0.315097222521513	UpNonUp
6862133	Smad7	1.32057216774261	0.755190496000195	0.483037470311345	UpNonUp
6867776	Rela	0.576801457864089	0.105822100534495	0.321792450363856	UpNonUp
6868650	Tmem2	0.518775976084981	1.08848488291903	0.931717050436411	UpNonUp
6869436	Hectd2	0.621237780722674	0.984620175495505	0.573259459452071	UpNonUp
6870125	Ina	0.652517173445687	0.489438894918888	0.490532325620005	UpNonUp
6875197	Arl5b	2.18839029893283	0.384927692177992	0.305661372423067	UpNonUp
6876342	Hspa5	0.456968338061359	0.184082241614298	0.747357732482284	UpNonUp
6879925	Bdnf	1.73834489202467	1.36409877797807	1.34569226099382	UpNonUp
6881123	Slc20a1	0.37437937895672	0.387766391168491	0.685338891098576	UpNonUp
6882264	Srxn1	0.549184485179576	1.04370794485686	1.2269282791848	UpNonUp
6885879	Slc25a25	0.84173905245425	0.500677787041957	0.265490839420825	UpNonUp
6888937	Hsd17b12	0.347398186643549	0.529349548178513	0.390025820525303	UpNonUp
6894278	Gmeb2	0.608474780002999	0.664633281548066	0.574763793200384	UpNonUp
6897337	Ccrn4l	0.692715052779827	0.600363941103322	0.422776689407094	UpNonUp
6898076	Tiparp	2.46256222021875	0.838359866951004	0.466613370210529	UpNonUp
6898873	Fbxw7	0.561576031066016	0.610450542628212	0.326610542235147	UpNonUp
6899014	Etv3	0.884983166829991	1.22628587955113	0.835380486599013	UpNonUp
6899252	Pmvk	0.757433030369657	0.519412187271261	0.488494738886521	UpNonUp
6899694	Mcl1	0.751407838512303	0.605061063097965	0.627864556028137	UpNonUp
6899838	Prkab2	0.7084508192993	0.389456129713206	0.495736679098624	UpNonUp
6909555	Tet2	0.460349410992817	0.663908550047323	0.221877467631481	UpNonUp
6912989	Dnajb5	1.08581094982857	1.16454792108541	0.964022584042125	UpNonUp
6916775	Hivep3	0.263510021376117	0.27546385773555	0.533706493636611	UpNonUp
6917055	Epha10	0.605189240162275	0.510313658618023	0.381567757428032	UpNonUp
6918892	Errfi1	2.10260831684748	0.869703505900581	0.341355980846353	UpNonUp
6919163	Cdk11b	1.00085311344036	0.43831511658092	0.213896498291941	UpNonUp
6919932	Osgin2	0.423392815285326	0.821204235591734	0.703054876985366	UpNonUp
6923411	Jun	1.53216031980266	0.816134047978578	0.501562602703581	UpNonUp

6924832	Plk3	1.34870316266345	0.293057727256878	0.533437566621575	UpNonUp
6925872	Gpr3	1.78054756993363	1.71295521012943	1.53605421269208	UpNonUp
6926498	Efhd2	0.520248575956331	0.580758353773015	0.791701520481404	UpNonUp
6926987	Spsb1	0.432310717198897	1.20468961325121	0.522813042784065	UpNonUp
6929644	Dpysl5	0.402089791020334	0.745921286921238	0.358270219962823	UpNonUp
6929719	Fosl2	2.17629033222639	0.964912405860251	0.595493948002425	UpNonUp
6933028	Aff1	0.765144346299941	0.589730728198371	0.393602686356538	UpNonUp
6935502	Zfp655	0.424346389700372	0.523179161956068	0.324816362490406	UpNonUp
6936564	Ptpn12	0.802379985502713	0.27948810706673	0.4555064316121	UpNonUp
6937051	Cgref1	0.429696216296026	0.898619724395972	1.47436904058574	UpNonUp
6946055	Gpnmb	0.316945107209334	0.440257341866557	0.713784793849019	UpNonUp
6955032	Tet3	0.474398702762784	1.22876880383096	0.642495091277908	UpNonUp
6957051	Slc2a3	0.717309835132657	0.380639156590657	0.385561075929289	UpNonUp
6959292	Numbl	0.321810928717894	0.344883573858288	0.649599808214031	UpNonUp
6962876	Gdpd5	0.317426117486759	0.665500597694714	0.985734803926605	UpNonUp
6964424	Bag3	1.02025735727622	1.77648197410598	1.48670912520013	UpNonUp
6967006	Sult2b1	0.540076989179983	1.1034590223033	1.49248422623405	UpNonUp
6968781	Furin	0.210856771945297	0.656627760440596	0.798920353702452	UpNonUp
6969837	P2ry6	0.420786415719492	0.319955884755411	0.508729726076353	UpNonUp
6973247	Nup98	0.588961854717811	0.575905758719789	0.281322570362949	UpNonUp
6974639	Plat	0.686038548937986	0.421775042155199	0.486723635964197	UpNonUp
6975335	Dusp4	1.35448804004311	1.14818715645923	1.2423896390294	UpNonUp
6977027	Jund	1.08528963341104	0.212191403745383	0.293464564890382	UpNonUp
6977676	Dnajb1	1.21413656259451	0.114603557295387	0.364680391492298	UpNonUp
6983894	Junb	2.45540306627708	0.761934931259806	0.385101398686797	UpNonUp
6989534	Adpgk	0.336999545840067	0.61960367504859	0.507909477355246	UpNonUp
6989672	Tle3	0.298666815101474	0.424441140748819	0.532808706579792	UpNonUp
6990131	C2cd4b	1.25271092001144	1.12818369845857	1.18971361265448	UpNonUp
6991027	Tpbg	0.724156204281847	0.618699221181937	1.32148120869936	UpNonUp
6992367	Prkar2a	0.321318525754631	0.490834688648744	0.508260371140374	UpNonUp
7011050	Rab33a	0.261918662508596	0.677978759453919	0.694965970584352	UpNonUp
7014815	Rps6ka3	0.399227508655474	0.562018182885447	0.83831149448983	UpNonUp
7014929	Rbbp7	0.624267571128281	0.727227036553798	0.694343926410364	UpNonUp
7019867	Acsl4	0.48993622642025	0.575711137220426	0.47593054091324	UpNonUp
6748620	Arid5a	1.00501864196555	0.343363735102909	0.252378778118419	UpNonNon
6753439	Zfp281	0.582843197885419	0.109782014106006	-0.118578077910601	UpNonNon
6756403	Slc30a1	0.599087565804291	0.237577025718776	-0.108907821240472	UpNonNon
6764007	Hsd17b7	0.534951857178817	0.390991032938587	0.0814903667053686	UpNonNon
6766368	Map3k5	0.475836681205057	0.661912063323378	0.1701932170378	UpNonNon
6766586	Taar7b	0.777568855098205	0.328090490306169	-0.270223872364082	UpNonNon
6768693	Ipmk	0.403049531307659	0.52449909893824	0.143007756306111	UpNonNon
6769448	Rfx4	0.56737618951562	0.533386256448562	0.00439067538534775	UpNonNon
6769928	Tmcc3	0.752675164740962	0.262977575688154	-0.129619384853277	UpNonNon
6770030	Btg1	0.63896444560618	0.184353670486439	0.0696259067497923	UpNonNon
6770160	Dusp6	1.75367340678577	0.164754830641548	0.278169369642767	UpNonNon
6770201	Kitl	1.51147980121051	0.047035458186678	-0.0558065651614057	UpNonNon
6774719	Arid5b	0.605335530344712	0.732192380523946	0.0158554018176322	UpNonNon
6775391	Mob3a	0.627133969436605	0.109697178736581	0.0406663303435478	UpNonNon
6779264	Peli1	0.929727167985792	0.249546095421329	-0.030277128102303	UpNonNon
6779787	Psme4	0.564967968040652	0.457813332286437	0.172860979478933	UpNonNon
6780544	Med7	0.697494921474215	0.590513047591888	0.212262407204278	UpNonNon
6781612	Pmp22	0.679205019291271	0.239870484751023	-0.0904021079325874	UpNonNon
6781984	Per1	1.66798331957098	0.340498002156515	0.0539993258271624	UpNonNon
6783255	Rnft1	1.11958815809176	0.60432869660743	0.2296740350943	UpNonNon
6783642	Tob1	0.715587379438882	-0.398339997224778	-0.272019654437972	UpNonNon
6784485	Arf2	0.500608812288603	0.622045851632186	0.244151274050343	UpNonNon
6784845	Kcnj2	2.96002360067375	0.483871252341312	0.113267645136828	UpNonNon
6791965	Ddx5	0.620312427038819	0.194966271293203	-0.153932084185216	UpNonNon
6792129	Abca5	0.553253765699094	0.371285819808647	0.0617737626450418	UpNonNon
6796696	Jdp2	0.544537269957077	0.24647196335568	-0.115525276601214	UpNonNon
6797880	Ccnk	0.582557082065049	0.201745722647756	0.194049955221498	UpNonNon
6802088	Zfp36l1	0.676563658373826	-0.252168704593401	0.087453518175139	UpNonNon
6808308	Ell2	0.721114896213004	0.467282912913857	0.280674118975223	UpNonNon
6812250	Pxdc1	0.972995539254229	0.108708391996622	-0.197100602717061	UpNonNon
6815305	Hmgcr	1.27566691060271	0.597011874021302	0.12390717226811	UpNonNon
6815853	Zswim6	0.932596752485986	0.461250177373708	0.192610579818521	UpNonNon
6817053	Atxn7	0.643976892039051	0.0137802717966311	-0.0689424270392752	UpNonNon
6817393	Vcl	1.11852174375131	0.379805768419984	-0.108479046676625	UpNonNon

6817900	Selk	0.696737886347955	0.350627740948089	0.054747999248067	UpNonNon
6822729	Fezf2	0.58690172084573	0.427294640023259	0.250317309056692	UpNonNon
6824835	Tinf2	1.41932483515228	0.612064693091176	0.128671071857413	UpNonNon
6827123	Kctd12	0.564280544087105	-0.0309349292592716	0.0578723824082609	UpNonNon
6832532	Pim3	0.897483831843902	0.423611424375686	0.216950560054126	UpNonNon
6842552	Cxadr	0.501115843751175	0.0420175235450689	0.15406739667131	UpNonNon
6843928	Txndc11	0.86102352306359	0.116416097869738	0.194310837069721	UpNonNon
6846375	Nfkbiz	0.828245832107666	0.209804464482599	0.0388581184984012	UpNonNon
6849098	Zfp948	1.59503135176016	0.789388261144822	0.243423054136242	UpNonNon
6852750	Plekhh2	0.739959817331766	0.108821718560428	-0.116145116925031	UpNonNon
6855067	Hspa1a	0.530618607374022	0.191236893465668	0.377936389860846	UpNonNon
6855706	Srf	1.03429419382628	0.0379961196477192	0.0686265293008755	UpNonNon
6857037	Myl12a	0.69838632783332	0.517838117556846	0.228283776812592	UpNonNon
6857523	Srsf7	0.567927594528678	0.089493462340731	0.0546620465079134	UpNonNon
6857797	Zfp36l2	0.697150669688059	-0.0464675108616727	0.0496571523599857	UpNonNon
6864564	Etf1	0.618445380699091	0.407850358869876	0.194056314667512	UpNonNon
6868618	Zfand5	0.702040717322061	0.21099704968116	-0.0319467660888329	UpNonNon
6871062	Npas4	5.10828686142042	1.6175764538556	0.659656959648124	UpNonNon
6872888	Cpeb3	0.759056716521239	-0.0579670204320746	-0.029892239188	UpNonNon
6879087	Chrm4	0.788156988745035	-0.074267584428967	-0.0267808521988194	UpNonNon
6880393	Thbs1	2.36635202797114	0.556297719932486	0.0265736933178762	UpNonNon
6884352	Hspa14	1.03856883626083	0.163370936060749	0.0419330077604947	UpNonNon
6886678	Rnd3	0.872545556430897	0.515971443819899	0.345451059379114	UpNonNon
6886908	Nr4a2	1.76656359337333	0.599126903966101	-0.0903655203585823	UpNonNon
6888307	Ctnnd1	0.66398748380535	0.163421951920753	0.0622626454099952	UpNonNon
6893263	Dpm1	0.537774353216419	0.374055240248283	0.033596436568357	UpNonNon
6900180	Slc16a1	1.56084675950046	0.541976591658743	0.209033754365675	UpNonNon
6905366	Siah2	1.18441566183098	0.320439572799626	0.155007096939543	UpNonNon
6905657	Ccnl1	1.0350724636194	0.316724385065498	0.0939869777915724	UpNonNon
6910611	Dnajb4	0.579136991399068	0.100687130765089	-0.0840030250418933	UpNonNon
6916748	Slc2a1	1.13167501287049	0.448822963687453	-0.128647484233575	UpNonNon
6917577	Map3k6	0.369671512544013	0.590241635368457	0.177628483288491	UpNonNon
6931682	Rasl11b	0.868309700703332	0.426628243790007	0.391807062411112	UpNonNon
6941856	Vps37b	0.59013036068903	-0.187568189797928	0.116407900239324	UpNonNon
6952470	Lincpint	1.31611536652502	0.538036637817956	0.107336809922654	UpNonNon
6957654	Gpr19	1.3025201082255	0.311718370706892	0.140144257878874	UpNonNon
6966991	Ppp1r15a	1.08530747901503	0.174937696605673	0.166458725653712	UpNonNon
6983698	Usp38	1.03196140845432	0.334824602038988	0.15342235711022	UpNonNon
6984484	Ciapin1	0.500145166146515	0.314363378116291	0.155718173667185	UpNonNon
6985900	Slc7a5	0.345334360744235	0.528378577552743	0.334969648814257	UpNonNon
6988604	Usp2	0.534651536978437	0.284945934651638	-0.136050919198797	UpNonNon
6994935	Sc5d	0.571115180317539	0.551125845266586	0.100860988625787	UpNonNon
6995534	Sik2	0.783890614391469	0.68219103105171	0.158157110343197	UpNonNon
6997288	Htr1b	0.740826382095755	-0.0373180734374242	-0.0964879957222573	UpNonNon
6998708	Arih2	0.718161589305474	0.413024117368206	0.184864262690702	UpNonNon
7011928	Zfp275	0.625954408297171	-0.00604732014801198	-0.0493941616334914	UpNonNon
7011996	Emd	1.44080013960652	0.654378529249093	0.212416489527134	UpNonNon
7017784	Prkx	0.69206664390086	0.167486436106581	0.0599619391339862	UpNonNon
7023079	Ddx3y	0.706947752792577	0.124537086153698	0.178792559042294	UpNonNon
6788723	Rasd1	0.96094094844244	0.376768430402836	-0.550544749637185	UpNonDown
6817700	Arhgef3	0.616866472438489	0.223466563257121	-0.308941774671058	UpNonDown
6861260	Isoc1	0.280012365689023	-0.324512947267095	-0.645842308506868	UpNonDown
6913315	Nr4a3	2.11063628739927	0.433889923400391	-0.467063615060424	UpNonDown
6929510	Insig1	0.720473855520623	0.146982584137301	-0.265740667342871	UpNonDown
6773020	Rspo3	0.293249141942515	-0.562634330357525	-0.891921515423023	UpDownDown
6878702	Ypel4	0.286992013434755	-0.710666063397765	-0.590025450340653	UpDownDown
6758958	Fam126b	0.104496159370671	0.628653120593721	0.890919163438907	NonUpUp
6784056	Rara	0.0729277768291664	0.771345878133637	1.11023592996552	NonUpUp
6803780	Cinp	0.117981535061507	0.467235058678363	0.803632787801817	NonUpUp
6815259	Crhbp	0.110752076178937	0.890518955033906	1.07427821030219	NonUpUp
6833185	Gpd1	0.100002383462089	0.513668703162241	0.69810122804955	NonUpUp
6869885	Entpd7	0.124863378123653	0.83493154584045	0.747872071932625	NonUpUp
6883533	Dok5	0.123636310871741	1.13030476165099	1.35881826661682	NonUpUp
6899520	S100a10	0.275890421323111	0.984748096021257	1.12960704805082	NonUpUp
6901780	Tspan5	0.0132082820387018	0.384526219256861	0.520412805142221	NonUpUp
6925654	Serinc2	0.168675561268027	0.712095692745332	1.41364240285942	NonUpUp
6961201	Snrpa1	-0.188505765057554	0.48883786600665	0.508492889119358	NonUpUp
6963456	Ampd3	0.0360731206524277	0.431551312886306	0.5841086912172	NonUpUp

6966498	Dpy19l3	0.171879462495491	0.76283896298508	0.695345116076798	NonUpUp
6969207	Prss23	0.119487849407014	1.00046780413799	0.914544383043268	NonUpUp
6974220	Dlgap2	-0.0625142115865721	0.604814455988252	0.823281417188539	NonUpUp
7011944	Slc6a8	0.246856851002545	0.893985807164599	0.912409560845584	NonUpUp
6752409	Nifk	-0.328025395556704	0.758615424134987	0.391805470900815	NonNonUp
6754137	Rgs8	-0.0594709607444995	0.304727157160814	0.676100256758213	NonNonUp
6754437	Rfwd2	0.123071956429857	0.615252555940273	0.567575425990701	NonNonUp
6756637	Rgs20	0.250369302286487	0.270175865056997	0.5698959889528	NonNonUp
6757745	Zfp451	-0.190298971974881	0.530243844242458	0.52442113669722	NonNonUp
6762197	Cdk18	0.184291040783044	-0.00664537001088092	1.01109040038151	NonNonUp
6764349	Rgs7	0.0702719468761173	0.398644810630711	1.02293358373449	NonNonUp
6765504	Rgs17	-0.0203211145137509	0.352827830048036	0.750612790768164	NonNonUp
6769366	Chst11	0.119085245828709	0.566073581783559	0.732068673703489	NonNonUp
6771334	Ppm1h	-0.106493869819309	0.752193238065512	1.00412397283807	NonNonUp
6772875	L3mbtl3	-0.075354973815169	-0.0435713785474971	0.697599642304842	NonNonUp
6775559	Slc41a2	0.0754400249042903	0.161639992799442	0.656644103015195	NonNonUp
6775674	Cry1	0.161257902110965	0.440772576910939	0.650155898826976	NonNonUp
6779162	Spred2	0.292940707882061	0.722238627616233	0.802386678506994	NonNonUp
6779843	Sh3pxd2b	0.0636489927471287	0.507128730780716	0.906091164589686	NonNonUp
6779845	Ubtd2	0.0892634546157517	0.450012368229347	0.542514818327215	NonNonUp
6780945	Fstl4	-0.0727859641678375	0.32856964570365	0.883187746345458	NonNonUp
6782277	Camkk1	-0.232045321147055	0.358390283669681	0.57577836267476	NonNonUp
6782422	Rtn4rl1	0.13186228252288	0.520182074369393	0.817089173516345	NonNonUp
6783784	Phospho1	0.030409370857565	0.404708384604254	0.601919615350879	NonNonUp
6787100	Rhbdf1	0.118060013507187	0.355357540742222	0.801688891069256	NonNonUp
6788314	Pdlim4	0.156498672685752	0.368547421316887	0.613883074224025	NonNonUp
6790947	Cacna1g	0.106901556794143	0.154079049655322	0.501225260534024	NonNonUp
6791641	Gfap	-0.0423583432755222	1.01032561089813	1.09605688992652	NonNonUp
6792314	Sdk2	0.23653703735708	0.444933108966963	0.539945285885433	NonNonUp
6792458	Grb2	0.132812633202777	0.337976590622264	0.565685584346445	NonNonUp
6792614	Socs3	0.072592062586303	0.564375050146604	0.952689591816309	NonNonUp
6796485	Pcnx	-0.100397924622478	0.537406819833794	0.524252238976744	NonNonUp
6796500	Rgs6	0.213501632514939	0.287946291780995	0.927248766645037	NonNonUp
6797476	Slc24a4	-0.123675108306092	0.506321891428298	0.953867110493255	NonNonUp
6797496	Chga	0.00156722115724504	0.434133043763393	0.933273985477144	NonNonUp
6797551	Ppp4r4	0.0404484780916539	0.870171870708761	0.840714737087926	NonNonUp
6797579	Serpina3n	-0.0797737203455612	1.16409019511459	1.19418233103048	NonNonUp
6799462	Id2	0.258207747557038	0.399435025155315	0.51255508322496	NonNonUp
6799578	Sox11	0.251910198626812	0.851834806734944	1.33902916561049	NonNonUp
6801507	Trim9	-0.0582062971377721	0.441480604115125	0.563999194714346	NonNonUp
6803113	Gpr68	-0.173108554868081	0.275210966162292	0.681203044899602	NonNonUp
6805158	Elmo1	0.208358523941643	0.877331723766509	1.01619704415454	NonNonUp
6817611	Zmiz1	0.0148789103795587	0.765961457015762	1.35539093857822	NonNonUp
6818667	Samd4	0.0487133838203922	0.721344559100792	0.778779478748915	NonNonUp
6819596	Sacs	-0.206269345734961	0.567283493326924	0.545777494215827	NonNonUp
6819910	Adra1a	0.224136049268944	0.646506345438055	0.961120642006864	NonNonUp
6825853	Fndc3a	-0.20014577433471	0.236156909134436	0.585175832722325	NonNonUp
6832092	Mchr1	0.39379235514697	0.499106601085456	0.837142445618439	NonNonUp
6835089	Klf10	0.0647244785073345	0.892188693253708	1.44862227888147	NonNonUp
6836560	Fam135b	-0.110532874620995	0.703329714035787	0.631572219146215	NonNonUp
6836596	Ago2	0.0708017179056905	0.350339703248639	0.618055598288849	NonNonUp
6837144	Pdgfb	0.0778079837727513	0.536446864023854	0.574126684950701	NonNonUp
6837455	Scube1	0.0773771532408379	0.469918389288704	0.872914047656053	NonNonUp
6838257	Slc38a2	0.200376897617225	0.0436933692603989	0.624711673523774	NonNonUp
6838823	Ppp1r1a	0.14503225725026	0.123616348513062	0.545377249422995	NonNonUp
6841097	Naa50	-0.0737972965575441	0.347869771408824	0.508182382816088	NonNonUp
6842940	Bach1	0.123873274558711	0.434770412021485	0.508815679951173	NonNonUp
6844291	Med15	0.082692238214967	0.146623410802594	0.520713383376651	NonNonUp
6848718	Airn	0.351562830093064	1.15893735508185	1.47869255508965	NonNonUp
6850204	Ppp1r10	-0.0264675420304571	0.348228560298501	0.543896765706899	NonNonUp
6851320	Trip10	0.0957000768890239	0.516526084535637	0.706025539477977	NonNonUp
6852034	Lpin2	-0.113473370799488	0.101892041779676	0.655259297663673	NonNonUp
6857167	Galnt14	-0.0408209908675534	0.680807946026727	1.20548875297382	NonNonUp
6864680	Hbegf	0.282777258420788	0.554615555063962	1.11936292800419	NonNonUp
6866653	Mapk4	0.222857534878858	1.1298229387547	1.46308864618354	NonNonUp
6866852	Slc14a1	0.0943674755170115	0.500914563455172	0.996794826637252	NonNonUp
6869766	Ubtd1	-0.000672105860843046	0.0938935911250207	0.728040256032794	NonNonUp
6870193	Sorcs3	-0.047719033053457	0.810972057698464	1.74706694915198	NonNonUp

6871004	Syt12	-0.0959858994656948	0.374447871592996	0.732136554129742	NonNonUp
6873187	Crtac1	0.0717900335264968	0.141838849904584	0.513642148800073	NonNonUp
6874057	Gfra1	-0.0871072613188358	0.228629301981858	0.636190167730267	NonNonUp
6875132	Vim	0.142337820572051	0.717082401981225	0.956331408830326	NonNonUp
6876209	St6galnac4	-0.0341677369384944	-0.025843095134489	0.847485390925088	NonNonUp
6876226	Fam129b	0.124774424464812	0.518265038589536	0.875445920179655	NonNonUp
6878655	Itgav	0.107007134861987	0.323920284923141	0.701587677279128	NonNonUp
6879833	Kcna4	0.132029951335421	0.682684360683736	0.562860034200622	NonNonUp
6880544	Tyro3	0.20057288964921	0.333772355994079	0.717813345180601	NonNonUp
6884709	Pfkfb3	0.195964391195816	0.605395191436876	0.837888620697289	NonNonUp
6885039	Dnajc1	0.182064738437745	0.612659027299617	0.519071392605123	NonNonUp
6886957	Acvr1	0.133883224397375	0.699984433166364	1.00075843574892	NonNonUp
6888334	Rtn4rl2	0.172410874721928	0.604473352824025	0.767051766269222	NonNonUp
6890638	Gabpb1	0.0763924431242463	0.200958130638091	0.625934410128541	NonNonUp
6890699	Kcnip3	0.144526170727204	0.278643587838533	0.828719697019331	NonNonUp
6891295	Pak7	-0.0144781717152845	-0.047945319259802	0.561779196854353	NonNonUp
6896850	Fgf2	0.0596663675439418	0.438482191801601	0.767844984753468	NonNonUp
6897486	Lhfp	0.154404789436059	0.242605951428146	0.949631667981725	NonNonUp
6899722	Anp32e	0.102379284067402	0.547314640904218	0.536889834465796	NonNonUp
6905208	Sertm1	0.185143318763208	0.963582550948675	0.61030496802039	NonNonUp
6905296	Wwtr1	0.0850938936422465	0.345926374366659	0.538463591102895	NonNonUp
6906821	Efna1	-0.191166267069144	0.238060688168776	0.536622580270952	NonNonUp
6907351	Pde4dip	-0.0763073068500199	0.631557495781696	0.605475129124543	NonNonUp
6907352	Pde4dip	-0.217978365019966	0.595437221972901	0.827811475961736	NonNonUp
6916095	Ssbp3	0.17016035067053	0.203596338441479	0.62100131021538	NonNonUp
6916127	Ndc1	0.0262428110518568	0.532737224227893	0.730609756415057	NonNonUp
6916947	Hpcal4	0.0509199895313486	0.275783513923531	0.569216298875133	NonNonUp
6919003	Tnfrsf25	-0.230121202842337	0.300524943401979	0.562324869765829	NonNonUp
6920816	Lingo2	0.0266574020555565	0.104455266214785	0.766950172883763	NonNonUp
6922471	Brinp1	0.163684994729074	0.775865435943763	0.8794211438634	NonNonUp
6924882	Ptprf	0.0646210732910964	0.172995805291356	0.597805117913475	NonNonUp
6925904	Trnp1	0.0937979953725712	0.18801070234282	0.622721104373226	NonNonUp
6925917	Zdhhc18	0.0941298511851983	0.300208081822447	0.789559587664719	NonNonUp
6926272	Cda	-0.0914885650409807	1.21754763148171	1.78952630518546	NonNonUp
6928880	Sema3e	-0.0938765463542831	0.481791257670866	0.728071086221858	NonNonUp
6931961	Lphn3	-0.0744298319487592	0.156448904684036	1.16287137137728	NonNonUp
6935370	Fscn1	0.19378939990622	0.1261849621132	0.509044055250633	NonNonUp
6937061	Slc30a3	0.108362453955602	0.0671711036979656	0.50584675520456	NonNonUp
6941186	Coro1c	0.244212740052873	0.373166876274548	0.555524542128753	NonNonUp
6941932	Ncor2	0.033741765717528	0.133469560530337	0.535028849637801	NonNonUp
6943310	Hsph1	0.112149467931803	0.56826538108367	0.907456669328713	NonNonUp
6944372	Cav1	-0.06531882537829	0.139239190142175	0.590881461090549	NonNonUp
6946558	Herc3	0.0204963126652498	0.393097800274595	0.750082401224537	NonNonUp
6948653	Ppp4r2	-0.176205590775563	0.497066225496657	0.655573782231259	NonNonUp
6949865	Tnfrsf1a	0.136679518558753	0.500978187697642	0.642977031452195	NonNonUp
6952766	Creb3l2	0.134404245026509	0.185573965828004	0.940382777784067	NonNonUp
6955025	Mthfd2	0.14193521527226	0.193558975275879	0.534889710783449	NonNonUp
6955698	Lrig1	0.0751427721478488	0.304834730527233	0.857484138247162	NonNonUp
6956045	Cntn3	-0.168517480059734	1.11089377502652	0.899741446319674	NonNonUp
6956909	Lrtm2	-0.0604596539495238	0.512333778720711	0.77194026783468	NonNonUp
6956926	Erc1	0.1373306133688	0.811958397180993	0.295193772120692	NonNonUp
6958256	Bhlhe41	-0.134624931531561	0.499458907106633	1.20100382688462	NonNonUp
6959300	Blvrb	0.266579022459694	0.421767515345894	0.802771203727227	NonNonUp
6965045	Inpp5a	0.00903710245208012	0.246918754612364	0.55738103809099	NonNonUp
6965187	Drd4	0.00244675647273815	0.481411691640271	0.73977384927597	NonNonUp
6966033	Itpkc	0.143479076177097	0.258800238590096	0.59390739347741	NonNonUp
6966164	Mrps12	-0.0190555400255943	0.35174846231296	0.545439902292411	NonNonUp
6971466	Mcmbp	-0.124908403056518	0.537673426764976	0.376900137531358	NonNonUp
6971688	Fam53b	-0.21801846254436	0.301312678949984	0.728826956990282	NonNonUp
6973477	Oscar	0.0606915302332762	0.468733888206647	0.750323031476108	NonNonUp
6974039	Arhgef7	0.109700112894297	0.462375953355269	0.649466977092322	NonNonUp
6974850	Eif4ebp1	0.163231072695778	0.215812129336659	0.586283185882322	NonNonUp
6977058	Myo9b	-0.0230771408796643	0.411611469116876	0.681731467240181	NonNonUp
6979527	6430548M08Rik	0.00897568231676713	0.2277667245643	0.596243049139578	NonNonUp
6979914	Kcnk1	0.144640821901408	0.605034951253287	0.791427077884336	NonNonUp
6979919	Slc35f3	-0.215397418700667	0.281886495143865	0.569147640122515	NonNonUp
6980016	Nrp1	0.108427057480432	0.578306183547327	0.58989721241446	NonNonUp
6980364	Col4a1	0.160950657936517	0.500573795749316	0.480328526738997	NonNonUp

6981113	Plekha2	-0.508902183168224	1.30614559664755	1.10369709440672	NonNonUp
6983162	Ncan	-0.0797454603952385	0.42941886518815	0.581056851566686	NonNonUp
6983168	Slc25a42	-0.0487096053977528	0.134495127326772	0.649438932633407	NonNonUp
6985642	Cdyl2	-0.0286825728708856	0.325465598517188	0.875785817649439	NonNonUp
6988389	Clmp	0.00237368713513674	0.296211616873207	0.648026325977731	NonNonUp
6995846	Lingo1	0.179032266507256	0.291915789918652	0.663225651873709	NonNonUp
6996223	Fem1b	0.0421502542450992	0.49153169093594	0.61002278696713	NonNonUp
6996379	Snx1	0.121925064561298	0.579456086264784	0.488438177157199	NonNonUp
6998603	Dock3	0.0179999937921601	0.246235785395506	0.658477488813018	NonNonUp
7010327	Jade3	-0.283957365239622	0.652147843132941	0.815866186581233	NonNonUp
6747871	Crispld1	-0.174084948180018	-0.181213446407553	-0.517405295499789	NonNonDown
6748011	Tmem14a	-0.0242392385049833	-0.233860711683055	-0.563850576027217	NonNonDown
6748174	B3gat2	0.239759160827296	-0.520855656708904	-0.70186450879983	NonNonDown
6748553	Arhgef4	-0.13112831702927	-0.322736416899443	-0.535855850910435	NonNonDown
6748897	Slc9a4	-0.280198997459182	-0.583833358945016	-1.27381235506253	NonNonDown
6755125	Olfml2b	0.0654250315910303	-0.60257859642242	-1.21189584262112	NonNonDown
6755713	Lefty1	-0.259473911372984	-0.407908408311041	-0.532800663955984	NonNonDown
6759391	D630023F18Rik	0.0778846838377155	-0.506180066677024	-1.04947674931891	NonNonDown
6759664	Igfbp5	-0.0145402621607338	-0.44980703219816	-0.68714025541385	NonNonDown
6760251	Sphkap	-0.248243711802952	-0.243005870125562	-0.838625545666865	NonNonDown
6768014	Gja1	0.0629795846971309	-0.275428790447197	-0.697179919506748	NonNonDown
6768204	Tbata	-0.123469452437341	-0.49192660729767	-1.02488131739321	NonNonDown
6769209	Apc2	-0.106933097822169	-0.523509612484519	-0.374916168317298	NonNonDown
6769213	Plk5	-0.100733247038468	-0.822278137477989	-1.15798815488989	NonNonDown
6770325	Slc6a15	-0.105309420733036	-0.290212907567841	-0.753232382698525	NonNonDown
6770905	Ptprr	-0.0792647813500731	-0.279645490888578	-0.519224667366889	NonNonDown
6773080	Tpd52l1	-0.260214544203969	-0.26056316936148	-0.691907397820821	NonNonDown
6773169	Fam26e	0.164604937092002	-0.424649330534685	-0.516971511156861	NonNonDown
6773504	Smpd2	-0.0184062415522852	-0.454089645310168	-0.603892136637324	NonNonDown
6775413	Diras1	-0.135774837905479	-0.464658127226654	-0.518441097463402	NonNonDown
6777917	Arhgef25	-0.228609926514811	-0.333335945270533	-0.631789067096503	NonNonDown
6781960	Slc25a35	-0.105665524030419	-0.439735794509369	-0.735940504342788	NonNonDown
6782034	Sat2	-0.153478048561239	-0.451763249592115	-0.621709091138539	NonNonDown
6782656	Flot2	-0.106569240539867	-0.303125617365138	-0.51321711438712	NonNonDown
6783182	Bcas3	0.0395535770545216	-0.212792639909256	-0.552900511976528	NonNonDown
6785549	Fn3krp	-0.247266320680743	-0.506939016374783	-0.558698704195705	NonNonDown
6785641	Mtfp1	-0.0920047442892502	-0.321829271863609	-0.641105320496406	NonNonDown
6786914	Ccdc85a	-0.2029924278724	-0.423861034201495	-0.801239421240609	NonNonDown
6786991	Acyp2	0.142562291456835	-0.291298800836422	-0.588249019279732	NonNonDown
6788283	Sowaha	-0.325976199872242	-0.360376092569506	-0.64065303965321	NonNonDown
6788388	Anxa6	-0.25539079757802	-0.414402420400049	-0.591950840091926	NonNonDown
6791418	Hap1	0.0249455087617826	-0.589958104781683	-0.871974474501064	NonNonDown
6792113	Abca8b	-0.147330011788136	-0.458942113925385	-0.598048159009829	NonNonDown
6792539	Cygb	-0.12942466990917	-0.590739854784511	-0.479374491772567	NonNonDown
6792702	Sgsh	0.0341341494165325	-0.142022652653979	-0.506689188767312	NonNonDown
6794572	Etv1	0.0521438136827828	-0.298687735515675	-0.830493662325485	NonNonDown
6795794	Atp5s	0.0455731259061827	-0.350213937961897	-0.652748615477949	NonNonDown
6796060	Dbpht2	-0.00312712706524221	-0.419319838145856	-0.676479222205941	NonNonDown
6796403	Galnt16	-0.197673726409106	-0.413495738638726	-0.608016520736376	NonNonDown
6800468	Stxbp6	-0.259470250364614	-0.482873030533032	-0.874590291675134	NonNonDown
6803102	Rps6ka5	-0.0241094727131734	-0.413593875194813	-0.624527096147428	NonNonDown
6805381	Hist1h1c	0.150419234655059	-0.584720267425847	-0.81703488994412	NonNonDown
6807192	Tspan17	0.0329095586912347	-0.29047550530681	-0.560677690209843	NonNonDown
6808339	Mctp1	-0.0627675757812224	-0.253961029039663	-0.753634259336682	NonNonDown
6809030	Arsb	-0.149929083382201	-0.178878303757422	-0.555754340796388	NonNonDown
6809032	Arsb	-0.0415224133989959	-0.169272799723622	-0.676339801476135	NonNonDown
6811724	Lrrc16a	-0.161202553184766	-0.348433001489676	-0.571243151067729	NonNonDown
6811769	Acot13	0.00841928444036472	-0.225934263874071	-0.596590235941468	NonNonDown
6814355	Nkd2	-0.019926477114289	-0.473235283965597	-0.571701447686309	NonNonDown
6814996	Atg10	-0.038349839845046	-0.263284272011269	-0.548926486446107	NonNonDown
6819244	Cpne6	0.000369092718999971	-0.369058753531055	-0.632007533088974	NonNonDown
6820083	Lgi3	-0.0248121007951151	-0.35161151840268	-0.663546864388822	NonNonDown
6822367	Itgbl1	0.0548400519932138	-0.417522526843162	-0.855890543031785	NonNonDown
6822959	Ube2e2	-0.0895042158337602	-0.226816343916424	-0.524778018975544	NonNonDown
6824507	3632451O06Rik	-0.0510485148591029	-0.223706355801112	-0.542330695805297	NonNonDown
6824800	Jph4	-0.0966259151758289	-0.119820419801671	-0.513712264306356	NonNonDown
6824942	Cryl1	0.132463835419222	-0.319764737983806	-0.751005661106663	NonNonDown
6830481	Nov	0.176002561718086	-0.287462178293704	-0.807101454641443	NonNonDown

6834025	Npr3	0.0940794701727515	-0.380035652901518	-0.710162095681154	NonNonDown
6835403	Tmem74	0.176400642267501	-0.469390275072273	-0.800595692029801	NonNonDown
6839543	Mpv17l	-0.146032588949188	-0.346388275943296	-0.565164331738483	NonNonDown
6840981	D930030D11Rik	-0.275313523487427	0.406038422179502	-0.728981761484796	NonNonDown
6842436	Rbm11	-0.0349589347157249	-0.335630832927214	-0.721988315333559	NonNonDown
6843491	B3galt5	-0.18171739497138	-0.277787246793032	-0.869512911400756	NonNonDown
6843680	Rogdi	0.0462077552519853	-0.264547562248497	-0.570156873503815	NonNonDown
6844321	Tango2	0.176935538399388	-0.505559495330821	-0.428364653630015	NonNonDown
6845933	Sidt1	0.0340140451144075	-0.348094148143025	-0.853243248240142	NonNonDown
6854462	Decr2	0.00392807277613889	-0.151379552940293	-0.529292381587741	NonNonDown
6855225	Mog	-0.00958738555421963	-0.47225522741993	-0.679751146242874	NonNonDown
6862816	Neto1	-0.00738999891963044	-0.127806977694204	-0.573247908431959	NonNonDown
6866238	Ccbe1	-0.149330954860889	-0.462795247013537	-0.933686330689062	NonNonDown
6866257	Mc4r	-0.124629720781031	-0.408619660363011	-1.10322002612628	NonNonDown
6866545	Dcc	-0.0339342492059662	-0.107666517176308	-0.625505951721961	NonNonDown
6867710	B3gnt1	0.0348544812543447	-0.358180634973713	-0.577274525922403	NonNonDown
6867947	Slc22a8	0.168760810592367	-0.819989172035517	-0.998362153250334	NonNonDown
6868884	Smarca2	-0.168926551541895	-0.235195140435653	-0.541539868767509	NonNonDown
6870424	Add3	-0.124335920003795	-0.363189215185222	-0.649253520520891	NonNonDown
6871168	Capn1	-0.0425339966181543	-0.3256232112161	-0.532768621262248	NonNonDown
6871429	Asrgl1	-0.253605511910712	-0.338934215850845	-0.507372995598495	NonNonDown
6872584	Gldc	-0.0722284828185287	-0.439529299622458	-0.521337637298947	NonNonDown
6873368	Kcnip2	-0.247342510466213	-0.496194410115433	-0.874155750902361	NonNonDown
6874080	Hspa12a	-0.0827224878484466	-0.42085822733961	-0.668017334768313	NonNonDown
6876072	Cstad	-0.208928853660499	-0.0299271198269324	-1.02049559571912	NonNonDown
6878117	Sp3os	0.110901491696064	-0.331015405936182	-0.628380693142157	NonNonDown
6880469	Ivd	-0.0787076959280298	-0.283790949344963	-0.560585578861742	NonNonDown
6882538	Acss2	-0.0359395742339101	-0.506607724289196	-0.498560095310618	NonNonDown
6885325	Hnmt	0.0047373723629132	-0.398034990559942	-0.636591505080776	NonNonDown
6885522	Fam163b	-0.118830303370563	-0.411760360256135	-0.898388428601881	NonNonDown
6887671	Mettl8	-0.102212862156132	-0.493554397162725	-0.555581870775231	NonNonDown
6888151	Frzb	-0.181564615489484	-0.918697249720036	-1.41253947737212	NonNonDown
6891025	Adra1d	-0.163609062053051	-0.400773856019042	-0.518457202529185	NonNonDown
6892032	Acss1	0.0934084228157044	-0.421523764815699	-1.04548642515584	NonNonDown
6897441	Foxo1	0.238957868099135	-0.293012109906462	-0.627077451513867	NonNonDown
6898502	Fstl5	-0.021501690918252	-0.426830373862548	-0.655028725018225	NonNonDown
6899111	Paqr6	0.224331857671364	-0.24673351336067	-0.715286761497185	NonNonDown
6902665	Erich3	-0.155461358275077	-0.280052958469789	-0.548763636173979	NonNonDown
6903454	Cyp7b1	-0.19355477715337	-0.496771825200813	-1.04955901111669	NonNonDown
6904367	Gm5148	-0.270455610261913	-0.40734813136071	-1.10091591763321	NonNonDown
6906225	Tmem144	-0.0812174322674742	-0.370982137552328	-0.548121386732193	NonNonDown
6908068	4933431E20Rik	-0.213285161353378	-0.371110727484912	-0.740728239729813	NonNonDown
6908073	Gstm7	-0.0970125220490961	-0.56378983443734	-0.80357536321668	NonNonDown
6908088	Gstm4	-0.00850573395277747	-0.200257151656047	-0.662940130283414	NonNonDown
6908219	Ntng1	0.165041747169553	-0.300616398750737	-0.707134995464943	NonNonDown
6908461	S1pr1	0.0626231449271159	-0.244811915025113	-0.612426557014152	NonNonDown
6910642	Ak5	-0.103638898037292	-0.310747772953944	-0.634665280449723	NonNonDown
6911914	Calb1	0.0227880938701161	-0.437955179919697	-0.893023888615626	NonNonDown
6913128	Grhpr	0.0190430154734917	-0.484179237294145	-0.599214849064146	NonNonDown
6913371	E130309F12Rik	0.0899947614647785	-0.523261062511209	-0.74904020363684	NonNonDown
6916023	Ppap2b	0.0482875519096093	-0.265077068070265	-0.548623592227346	NonNonDown
6916159	Echdc2	0.154459378622661	-0.474280396436766	-0.794382676379735	NonNonDown
6917125	Oscp1	-0.11585330136804	-0.401340434096926	-0.535944684345955	NonNonDown
6917277	Fndc5	-0.0926939689449218	-0.330180476848864	-0.511790898603287	NonNonDown
6924706	Cyp4x1	0.124862165994106	-0.110615526476251	-0.619683231716331	NonNonDown
6924750	Faah	-0.108864138028733	-0.491912982979189	-0.804966387398618	NonNonDown
6926376	Klhdc7a	0.0728417481452886	0.113337831463291	-0.708926098996914	NonNonDown
6929667	Atraid	0.0046013323209523	-0.260323225176034	-0.500847892722316	NonNonDown
6932704	Prdm8	0.0612343353452386	-0.38943796515181	-0.958890488936727	NonNonDown
6935275	Chst12	-0.199634142131547	-0.926635476263374	-0.939219997035464	NonNonDown
6935970	Cdk14	-0.227846166855409	-0.170504523875209	-0.588452598995054	NonNonDown
6941637	Tpcn1	-0.092471984710948	-0.31722864422222	-0.501271714230094	NonNonDown
6946412	Adcyap1r1	-0.153166252654382	-0.601713240672403	-0.517882194111892	NonNonDown
6948328	Fam19a1	-0.13144303484715	-0.231145248546388	-0.540504131958896	NonNonDown
6948878	Lrrn1	-0.203430591135696	-0.482283968774219	-0.671555546070262	NonNonDown
6955169	1700019G17Rik	-0.157301729687765	-0.651494714177118	-1.00780384165797	NonNonDown
6955778	Frmd4b	-0.198395939926568	-0.404221839347725	-0.617870983260917	NonNonDown
6957217	Ntf3	0.00513924618404164	-1.08963835133683	-1.81470858219496	NonNonDown

6957679	Hebp1	-0.17879031450983	-0.346235914593368	-0.681510314689929	NonNonDown
6957844	Lmo3	-0.161477449121692	-0.30074537128089	-0.638555784940765	NonNonDown
6960266	Syt3	0.0414833820482511	-0.380053722105537	-0.731964041422948	NonNonDown
6960834	Siglech	-0.180503127410354	-0.232898236575495	-0.503161956029936	NonNonDown
6961109	Mcee	0.0610853811152068	-0.186719293864379	-0.631616483749249	NonNonDown
6961991	Hddc3	-0.0371712739030199	-0.242840835524532	-0.59866819189968	NonNonDown
6966972	Lin7b	-0.0739635804593948	-0.441220514482604	-0.679982909329651	NonNonDown
6969007	Il16	-0.306530813608676	-0.53296691505933	-0.93365245593618	NonNonDown
6969028	Fah	0.13429160033933	-0.281944969802071	-0.564353909488115	NonNonDown
6969094	Folh1	-0.191575025993416	-0.333994119495715	-0.755459110987176	NonNonDown
6969291	Tmem126b	-0.139563896226932	-0.532276053195776	-0.44494183518852	NonNonDown
6969612	Thrsp	0.146697655890456	0.0333312465701507	-0.60144486662066	NonNonDown
6969631	Aqp11	0.0839210372631471	-0.620167729827908	-1.29321085469032	NonNonDown
6972612	Ccdc106	0.0121114613087004	-0.362619528288403	-0.515534867678688	NonNonDown
6974783	Rnf170	0.044403297900559	-0.485434151120491	-0.602185235627669	NonNonDown
6980952	Smim19	-0.0151865788633543	-0.256402281430397	-0.600117878306695	NonNonDown
6982102	Tlr3	-0.128666636105027	-0.290572252646846	-0.642319940729008	NonNonDown
6982585	Wdr17	-0.0857470907049329	-0.224940263440362	-0.628537978718333	NonNonDown
6983073	Csgalnact1	-0.084099197607119	-0.564150004275538	-0.463575844904188	NonNonDown
6983255	Abhd8	-0.0230994895696615	-0.502296252441056	-0.408797180418594	NonNonDown
6983531	Ednra	0.173252623679091	-0.329894603483316	-0.532326138873653	NonNonDown
6984684	Cdh8	-0.211442089365135	-0.325682681053704	-0.65332761098932	NonNonDown
6987352	Icam5	-0.150175957339547	-0.331947310276949	-0.503262582532733	NonNonDown
6987638	Thyn1	-0.167195237999241	-0.279056689023291	-0.522168480328499	NonNonDown
6988366	Scn3b	-0.259075841219783	-0.337455539265182	-0.659215104858847	NonNonDown
6990435	Rab27a	-0.0748752424637806	-0.451272260745405	-0.534770837271042	NonNonDown
6991090	Rwdd2a	-0.0442405665838424	-0.298251455408693	-0.805034627660673	NonNonDown
6991368	Plscr4	-0.0569536319117575	-0.387480564174028	-0.747267580063988	NonNonDown
6991760	Faim	0.0687273925666534	-0.218000714012997	-0.681731549235498	NonNonDown
6993030	Fam198a	-0.21520054655539	-0.0992963699838101	-0.538798029125826	NonNonDown
6995526	1110032A03Rik	-0.0961488459928989	-0.587879520007587	-0.703464556510197	NonNonDown
6996440	Rab8b	-0.0943566023774935	-0.333147693369984	-0.536982423488025	NonNonDown
6998706	Wdr6	0.10709194743132	-0.452240470291615	-0.796187677899596	NonNonDown
7010345	Usp11	0.147302675131134	-0.417171740100523	-0.562197959842858	NonNonDown
7010644	Dock11	-0.277468862454307	-0.0307889351951336	-0.661199455734957	NonNonDown
7012080	Fundc2	-0.0455953057674697	-0.120898922123051	-0.671207545976665	NonNonDown
7014030	Tceal1	0.150281833714487	-0.350446619863786	-0.716098165830414	NonNonDown
7014633	Rragb	0.0524958086665363	-0.472150115786039	-0.576849466862554	NonNonDown
7015006	Pir	0.0624991196940346	-0.106071171280608	-0.65499203613568	NonNonDown
7015831	Maob	-0.0698881899363035	-0.334526409128861	-0.541304385319249	NonNonDown
7017585	Pnck	0.165106508611517	-0.585865237766991	-0.638843410051564	NonNonDown
6757387	Ogfrl1	-0.180072544510549	-0.682615988279019	-0.559712203216082	NonDownDown
6759396	Idh1	0.0428673465573386	-0.509106664718593	-0.903933869487349	NonDownDown
6760232	Slc19a3	0.0581033107707658	-0.40420303451634	-0.616808853030694	NonDownDown
6778939	Vstm2a	-0.0628804841576246	-0.474759140490937	-0.65753956295871	NonDownDown
6782139	Rnf167	0.0563565008530239	-0.555919521815214	-0.48582790723887	NonDownDown
6791529	Mpp3	0.00374263365313279	-0.436251482835293	-0.557822037796482	NonDownDown
6807172	Arl10	0.0486540104101041	-0.607298507102563	-0.493294843246073	NonDownDown
6810592	Fgf10	-0.0705181385210283	-0.495577129473795	-0.743578513291305	NonDownDown
6812770	Tbc1d7	0.166077705666119	-0.584457579065724	-0.410645133520617	NonDownDown
6814827	Rasa1	-0.254553351903271	-0.53077244479587	-0.388082735976577	NonDownDown
6837143	Cbx7	0.0903067483748675	-0.52589132159651	-0.348801104751322	NonDownDown
6850062	Prrt1	0.106229304364631	-0.449076013788962	-0.539375325115968	NonDownDown
6870489	Pdcd4	0.0245868220642691	-0.823265900949448	-0.816525874027534	NonDownDown
6884750	Itga8	-0.073913620521863	-0.803712218344744	-0.881454465364385	NonDownDown
6885425	Gm996	-0.0982492833028472	-0.609511618256741	-0.728053573356085	NonDownDown
6887282	Grb14	-0.0986538058962612	-0.555264716091225	-0.84730789234079	NonDownDown
6894245	Nkain4	0.167054100426221	-0.497954280794247	-0.619630838805139	NonDownDown
6903549	Gyg	-0.0863597028068199	-0.501905389664319	-0.459732294794387	NonDownDown
6906940	Lor	-0.0210417496864067	-0.502900599759448	-0.523635177428345	NonDownDown
6933328	Tmem175	0.0473584461515263	-0.445313067163509	-0.572980906709277	NonDownDown
6942841	Mmd2	-0.060764482443825	-0.375369569116775	-0.515828438448637	NonDownDown
6947131	Lrrtm1	-0.038077374300811	-0.790069086248993	-0.606917545763211	NonDownDown
6957766	Rerg	-0.124806781961361	-0.70077139571134	-0.667804518854918	NonDownDown
6964635	Lhpp	0.212779675633573	-0.519689829460744	-0.719194588181221	NonDownDown
6976395	BC030500	-0.0162890709146636	-0.546601715943228	-0.454982388945753	NonDownDown
6988672	Ift46	-0.0616905417235757	-0.410438223302858	-0.549783087823642	NonDownDown
6992176	Pcbp4	0.131228802234123	-0.395461043706827	-0.638425407776667	NonDownDown

6992276	Camkv	-0.0720127597355068	-0.429410527980714	-0.579779388329066	NonDownDown
6778426	Rasl10a	-0.512687491932496	-0.322590846628258	1.02369747137186	DownNonUp
6799173	Trib2	-0.268287814154865	-0.0168172621117427	1.01423470235277	DownNonUp
6836558	Fam135b	-0.266595596358485	0.578727712829619	0.544016479777154	DownNonUp
6896609	Actl6a	-0.509579662824249	0.207094577853012	0.566765249560535	DownNonUp
6899875	Zfp697	-0.317382087193119	0.429900258886288	0.534808839357053	DownNonUp
6925194	LOC102633765	-0.684597926874029	0.270204440555672	0.331893585352553	DownNonUp
6926477	Ddi2	-0.206651954985569	0.226664149368821	0.59952144049914	DownNonUp
6934308	Setd8	-0.269792971756447	0.682851248800666	1.07811229842126	DownNonUp
6973132	Zfp418	-0.579610028128231	-0.0151820975749907	0.247504906107733	DownNonUp
6748503	Rab23	-0.52868728916278	-0.142891823081924	-0.017715932056336	DownNonNon
6748884	Il1r1	-0.997972031987655	0.857258506457597	0.0897853436186862	DownNonNon
6753135	Rbbp5	-0.712240984313616	-0.249715459756183	-0.0544566714969829	DownNonNon
6762016	Lct	-0.899709921414516	-0.83031214670252	-0.585983723828418	DownNonNon
6774947	Cisd1	-0.684099915583484	0.327425704691956	0.0908043123937396	DownNonNon
6775288	2610008E11Rik	-0.926449025308701	-0.202092998665781	0.0654927761586126	DownNonNon
6779790	Gpr75	-0.501032921434235	-0.390066048513791	-0.138427430821826	DownNonNon
6788632	Zfp39	-0.826077541509351	-0.205732697813426	-0.165816984574187	DownNonNon
6790734	Stxbp4	-0.37491204663648	-0.529088003771533	-0.27617502615008	DownNonNon
6791881	Limd2	-0.516034039019576	-0.422304559607009	-0.387018497271836	DownNonNon
6794274	Ccdc71l	-0.976313538460719	-0.722449033991349	0.272014856763921	DownNonNon
6798388	Zfp386	-0.570513284818577	-0.339111197568327	0.132072174174931	DownNonNon
6799852	Dus4l	-0.600583023196906	-0.058811954009447	0.18550210039618	DownNonNon
6799874	Hbp1	-0.611811008874082	-0.317727647586607	0.0530208551277423	DownNonNon
6801703	Trmt5	-0.553666520996673	-0.36682919420755	-0.209828739785751	DownNonNon
6803525	A130014H13Rik	-1.09561812782267	-0.0433050408348568	0.0672622696070633	DownNonNon
6804898	Lyst	-0.523346927381924	0.100448972893538	-0.212114550201124	DownNonNon
6807812	Zfp759	-0.697432399707504	-0.0263139348759094	-0.064446611567032	DownNonNon
6809047	Lhfpl2	-0.325042417036472	-0.538236759843134	-0.213913504762992	DownNonNon
6811512	Zkscan8	-0.522499723786415	-0.141222576800063	0.0511825830556721	DownNonNon
6814055	Zfp87	-0.614910105280996	0.0166934990564096	0.110205558578677	DownNonNon
6815708	Ppwd1	-0.605324377723	-0.0285375844856391	0.118066370989911	DownNonNon
6818950	Apex1	-0.551249115564953	-0.226278497520179	-0.0445890500876955	DownNonNon
6819628	Trim13	-0.544639128258504	-0.466797336303004	-0.0385385167320511	DownNonNon
6824550	Ttc5	-0.596826668991016	-0.675476560489483	-0.264577377671262	DownNonNon
6825273	Ints6	-0.544896845702195	0.0696767315455894	0.107423280122916	DownNonNon
6834560	Fam105a	-0.534369168388241	-0.199831682058598	-0.148362052286069	DownNonNon
6844210	Fgd4	-0.503692680440478	-0.224507095480237	-0.073737756910491	DownNonNon
6846386	Cep97	-0.579657294425955	-0.29797335967205	-0.0671099334411192	DownNonNon
6847878	Dnajc28	-0.880532304786277	-0.49779141028903	-0.232157598499101	DownNonNon
6849162	Zfp946	-0.37716010903454	-0.62615066985622	-0.195630048890408	DownNonNon
6849923	Zfp952	-0.572019098894066	-0.0181337346644108	-0.0063208237693618	DownNonNon
6850063	Fkbpl	-0.637535279494448	-0.0359602450108625	-0.166339960661356	DownNonNon
6854895	Zfp799	-0.541374964182882	-0.0646519885050643	0.00923594802179742	DownNonNon
6854914	Zfp763	-0.558189935256785	-0.164500464118276	-0.177117206249394	DownNonNon
6864895	Nr3c1	-0.40491624724416	-0.508809468397294	-0.0948230844485706	DownNonNon
6878045	Rapgef4	-0.371977980942353	0.619249210933275	0.0980717005773584	DownNonNon
6881101	Zc3h6	-0.604567730570795	-0.127929329841351	-0.149663725222133	DownNonNon
6886114	Zbtb26	-0.567923895500225	-0.334589777515947	0.0178395308410789	DownNonNon
6908224	Prmt6	-0.534468057473753	0.134506773768515	0.128792921389519	DownNonNon
6912022	Rmdn1	-0.664249585559638	-0.155353570047506	0.0439454077247663	DownNonNon
6933652	Pop5	-0.579825867450201	-0.216296306281518	-0.14843776493796	DownNonNon
6935145	A430033K04Rik	-0.717186438237647	-0.141801853816948	0.075323239585184	DownNonNon
6946759	Prdm5	-0.60355975932522	-0.343557624566892	-0.369724637506144	DownNonNon
6959101	Zfp108	-0.530614270804461	-0.07725402392438	-0.0469484038027964	DownNonNon
6959323	Zfp60	-0.67029837850164	-0.180691403374151	0.161014825858775	DownNonNon
6961108	Mtmr10	-0.512676939083662	-0.0125342376031744	0.120028449109504	DownNonNon
6961979	Gdpgp1	-0.507254966427768	-0.20064881247166	0.0345577350008806	DownNonNon
6966232	Zfp74	-0.683254535028233	-0.226541640577197	-0.123920457644978	DownNonNon
6966249	Zfp14	-0.535268898668135	-0.31807849857019	-0.17981237259132	DownNonNon
6966255	Zfp146	-0.557294302190651	0.0393966345802735	0.0807574635827669	DownNonNon
6977119	Zfp961	-0.698449955589069	-0.407817021991941	-0.0908355098277246	DownNonNon
6981932	Frg1	-0.529662498580051	-0.28108772712182	0.204613956032973	DownNonNon
6987866	Zbtb44	-0.68140078033567	-0.0541039237174093	-0.00267452675351082	DownNonNon
6988115	Pus3	-0.516668267216097	0.134948624052797	0.137422745931722	DownNonNon
6991777	Cep70	-0.640016468622018	-0.495550990527947	-0.178398313688919	DownNonNon
6993872	Zfp599	-0.604766539218005	0.147502500792448	0.159797841341406	DownNonNon
6996110	Senp8	-0.783719093908614	-0.224436396980678	0.288869253787295	DownNonNon

6997348	Phip	-0.547466756550128	-0.0869210981124719	0.135678671703838	DownNonNon
7013294	Pou3f4	-0.54756576949124	-0.74996408481704	-0.125109254676053	DownNonNon
6748546	Amer3	-0.649363435739227	-0.369835115809276	-0.306432520151858	DownNonDown
6755494	Zbtb18	-0.584439802492129	-0.580701614782313	-0.723668097639342	DownNonDown
6767402	Sesn1	-0.231727162164044	-0.438370657781001	-0.536048499597765	DownNonDown
6773147	Clvs2	-0.400244251438597	-0.345340113761706	-0.531422991040047	DownNonDown
6773802	Grik2	-0.233651060195854	-0.281810718470016	-0.609071738187846	DownNonDown
6778284	Pik3ip1	-0.373801079989802	-0.490254303092686	-0.530616454352718	DownNonDown
6800314	Lrrn3	-0.297568036107678	-0.717484007906747	-0.669053525423712	DownNonDown
6800726	Dtd2	-0.226657208387623	-0.340088530005691	-0.521373794151737	DownNonDown
6806707	Gmpr	-0.296245701232505	-0.226232933721819	-0.504159075038138	DownNonDown
6809880	Htr1a	-0.441922966257481	-0.600002067075242	-0.642650856325648	DownNonDown
6815518	Mccc2	-0.235425107762201	-0.385911111339504	-0.591806277929773	DownNonDown
6815539	Ocln	-0.639683421917026	-0.652249443709903	-0.554981811358279	DownNonDown
6819928	Pnma2	-0.350642085607862	-0.605961608287811	-0.602297138136245	DownNonDown
6826611	Pcdh20	-0.511327657047168	-0.284051449861994	-0.477934314383353	DownNonDown
6829591	Sema5a	-0.433297779086175	-0.341021468354885	-0.783331336523902	DownNonDown
6849444	Neurl1b	-0.443129546453545	-0.64449304252675	-0.652263476311834	DownNonDown
6863156	Arhgap12	-0.501082165368442	-0.573973527353272	-0.674265149673989	DownNonDown
6895532	Pkia	-0.400417337831637	-0.500115898157389	-0.446016288550416	DownNonDown
6929550	Rnf32	-0.348846879371844	-0.56283007439116	-0.489376481310247	DownNonDown
6930361	Cc2d2a	-0.317542016099405	-0.433782833748359	-0.545226549394755	DownNonDown
6931243	Wdr19	-0.405223890646921	-0.559192938385005	-0.492555972849782	DownNonDown
6943081	Gpr12	-0.253388111832791	-1.04814767373382	-1.1592855997906	DownNonDown
6947211	Lrrtm4	-0.267562184266832	-0.454189652911433	-0.875196637567513	DownNonDown
6954000	Fam13a	-0.416779039044299	-0.70020771866735	-0.898153587454309	DownNonDown
6958031	Slco1a4	-0.496922061349405	-0.728650948636924	-0.771787515963389	DownNonDown
6967091	Uevld	-0.573493266745757	-0.643644113350937	-0.57766005915874	DownNonDown
6970911	Eri2	-0.552548863039047	-0.403547399161499	-0.240302579942359	DownNonDown
7019652	Rab9b	-0.425525657171858	-0.52080408336248	-0.496386660774782	DownNonDown
6791238	Fbxl20	-0.438894011017582	-0.506440097277672	-0.0957444878894356	DownDownNon
6891690	Rbbp9	-0.351491550789141	-0.52345154380478	-0.106585571124033	DownDownNon
6936575	Napepld	-0.653622390322131	-0.607023668941247	-0.178566557611582	DownDownNon
6945548	Ttc26	-0.511788615512201	-0.631303743171443	-0.167340741083475	DownDownNon
6981314	Brf2	-0.510395687117465	-0.51821926943439	-0.167755456312431	DownDownNon
6755569	Kif26b	-0.327596117659787	-0.59501142028128	-0.478735471672221	DownDownDown
6878448	Itga4	-0.387203540906313	-0.698506602194376	-0.891286350193821	DownDownDown
6906363	Npy2r	-0.671058317368511	-1.01372150151324	-1.65168829793275	DownDownDown
6915818	Raver2	-0.305834419711161	-0.744140317004971	-0.845930019045143	DownDownDown
6947570	Tprkb	-0.284343623730267	-0.654053618857625	-0.889180493281097	DownDownDown
6964349	Orai3	-0.347079806154766	-0.643064976932225	-0.833720659468079	DownDownDown
