# Supplementary material for: Neuronal activity regulates alternative exon usage
Source: Mol Brain. 2020 Nov 10;13:148. doi: 10.1186/s13041-020-00685-3 (PMC7656758; doi:10.1186/s13041-020-00685-3)

A Immediate early genes

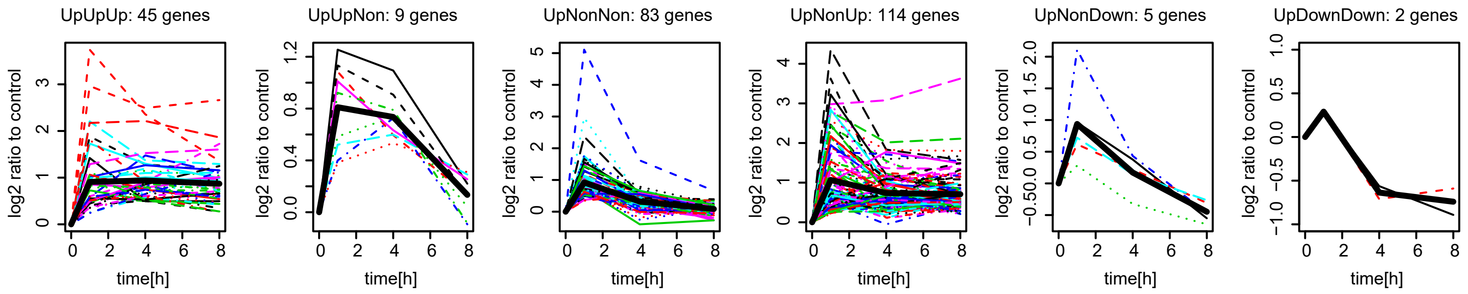

B Delayed upregulated genes

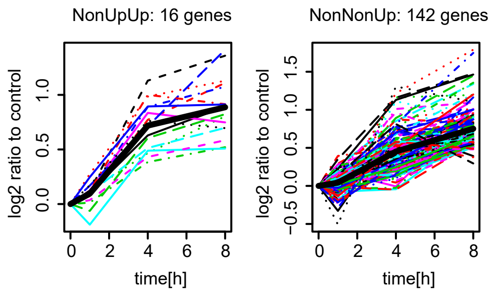

C Delayed downgulated genes

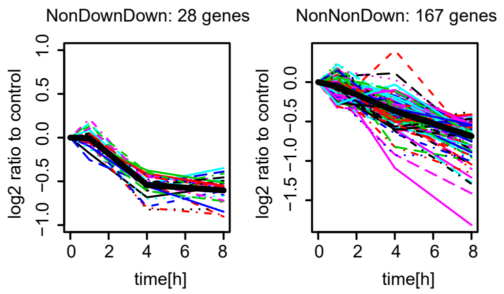

D Immediate downregulated genes

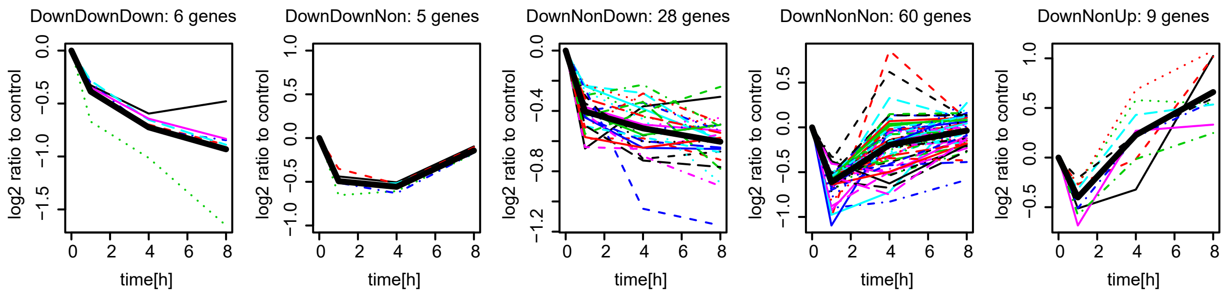

Supplement: Supplementary file 2 — Additional file 2: Temporal cluster analysis of activity-regulated genes. pdf. Activity-regulated genes identified on the whole gene level were assigned to clusters based on their differential regulation over four time points (0, 1, 4, 8 h after seizure onset). Similar expression kinetics were grouped and the thick black line represents mean expression profiles. [file 13041_2020_685_MOESM2_ESM.pdf]
