# Supplementary material for: Neuronal activity regulates alternative exon usage
Source: Mol Brain. 2020 Nov 10;13:148. doi: 10.1186/s13041-020-00685-3 (PMC7656758; doi:10.1186/s13041-020-00685-3)
Supplement: Supplementary file 3 — Additional file 3: Genes with activity-dependent splice variation identified through time-dependent ANOSVA. pdf. pValueBH, minimal adjusted p-value derived by Bonferoni and Holm method. DE, differentially expressed, indicates whether the whole gene was identified as induced by neuronal activity in our initial analysis (Additional file 1). [file 13041_2020_685_MOESM3_ESM.pdf]

Activity-dependent splice variation identified through time-dependent ANOSVA.

transcript	symbol	pValue	pValueBH	DE
6838650	Krt75	1.2e-16	1.62168e-12	-
6808997	Homer1	3.22e-11	2.175754e-07	+
6749557	Coq10b	7e-10	3.15326666666667e-06	+
6847952	Rcan1	1.14e-07	0.000385149	-
6918892	Errfi1	2.41e-07	0.0006513748	+
6879925	Bdnf	3.35e-07	0.000654463714285714	+
6765218	Atf3	3.39e-07	0.000654463714285714	-
6996448	Tpm1	4.1e-07	0.0006925925	+
6790508	Vmp1	1.01e-06	0.00151657111111111	-
6910173	Cyr61	1.56e-06	0.002108184	-
6926272	Cda	2.82e-06	0.00346449818181818	+
6766455	Sgk1	8.92e-06	0.0100454066666667	+
6833311	Nr4a1	1.16e-05	0.0120586461538462	+
6781984	Per1	1.34e-05	0.0129348285714286	+
6796691	Fos	1.55e-05	0.013682925	+
6778391	Lif	1.62e-05	0.013682925	-
6804996	Inhba	2.46e-05	0.0195555529411765	+
6799578	Sox11	5.24e-05	0.0374124421052632	+
6849626	Pim1	5.26e-05	0.0374124421052632	+
6837411	Cyp2d26	5.79e-05	0.03912303	-
6966759	C330019L16Rik	6.34e-05	0.0407994095238095	-
6834729	Ankrd33b	7.97e-05	0.0489575363636364	+
6880577	Ganc	8.51e-05	0.0500018	-
6833736	Osmr	9.17e-05	0.0516347416666667	-
6906635	Cd1d1	0.000112	0.0592536923076923	-
6967006	Sult2b1	0.000114	0.0592536923076923	+
7018819	Zcchc5	0.000119	0.0595617037037037	-
6906324	Tdo2	0.000169	0.0806978857142857	-
6974831	Prosc	0.000185	0.0806978857142857	+
6803269	Serpina9	0.000186	0.0806978857142857	-
6880322	Spred1	0.00019	0.0806978857142857	-
6919200	Fam132a	0.000192	0.0806978857142857	-
6972993	Chmp2a	0.000203	0.0806978857142857	-
6897599	Dclk1	0.000204	0.0806978857142857	-
6830770	Trib1	0.000209	0.0806978857142857	+
6782916	Ccl7	0.000226	0.0848378888888889	-
6898076	Tiparp	0.000244	0.08885455	+
6963624	Insc	0.000256	0.08885455	-
6887854	Chn1	0.000259	0.08885455	-
6989195	Gldn	0.000263	0.08885455	-
6890868	Pdyn	0.000308	0.101519804878049	-
6856367	Nudt12	0.000323	0.102769255813953	-
6964424	Bag3	0.000327	0.102769255813953	+
6768479	Egr2	0.000349	0.107190590909091	-
6960578	Nav2	0.000358	0.107511377777778	-
6998987	Arpp21	0.000425	0.123596791666667	-
6776049	Elk3	0.000435	0.123596791666667	-
6812652	Nedd9	0.000439	0.123596791666667	+

6957654	Gpr19	0.000454	0.125211346938776	+
6876181	Golga2	0.000538	0.14541064	-
6921873	Klf4	0.000577	0.15289368627451	-
6987665	Spata19	0.000609	0.158269730769231	-
6882264	Srxn1	0.000673	0.171602301886792	+
6907714	Sycp1	0.000747	0.186943666666667	-
6785224	Sphk1	0.000872	0.212236262295082	-
7010355	Timp1	0.000887	0.212236262295082	-
6787743	Cyfip2	0.000933	0.212236262295082	-
6935451	Nptx2	0.000939	0.212236262295082	+
6914006	Col27a1	0.000944	0.212236262295082	-
6855706	Srf	0.000948	0.212236262295082	+
6905208	6030405A18Rik	0.000958	0.212236262295082	-
6791960	Pecam1	0.000997	0.217313838709677	-
6965187	Drd4	0.00102	0.218798095238095	+
6911727	Gem	0.0011	0.230777538461538	-
6932559	C87414	0.00111	0.230777538461538	-
6859972	Egr1	0.00125	0.252904857142857	+
6795881	Frmd6	0.00128	0.252904857142857	+
6936759	Rheb	0.0013	0.252904857142857	+
6937106	Rbks	0.0013	0.252904857142857	-
6782451	Slc43a2	0.00131	0.252904857142857	-
6784290	Arl4d	0.00138	0.262666478873239	+
6849762	Glp1r	0.00151	0.281237297297297	-
6999449	Csrnp1	0.00152	0.281237297297297	-
6869595	Cyp2c37	0.00154	0.281237297297297	-
6867755	Fosl1	0.0016	0.286075584415584	-
6902789	Ankrd13c	0.00161	0.286075584415584	-
6890611	Shc4	0.00163	0.286075584415584	-
6880544	Tyro3	0.00167	0.289338205128205	+
6838828	Glycam1	0.00173	0.295939493670886	-
6898138	Ptx3	0.00178	0.3006865	-
6957217	Ntf3	0.00184	0.306984691358025	+
7010854	Xiap	0.00187	0.308185121951219	-
6989884	Megf11	0.00196	0.31912578313253	-
6888797	Creb3l1	0.00221	0.355546904761905	-
6762784	Rgs2	0.00226	0.356706744186046	+
6789471	Nup88	0.00227	0.356706744186046	-
6852845	Rhoq	0.00233	0.358348764044944	-
6956926	Erc1	0.00235	0.358348764044944	+
6880393	Thbs1	0.00236	0.358348764044944	+
6778426	Rasl10a	0.00266	0.399413777777778	+
6957898	Plcz1	0.00276	0.409875164835165	-
6969207	Prss23	0.00286	0.420109130434783	+
6782915	Ccl2	0.00291	0.421067789473684	-
6998750	Fbxw21	0.00295	0.421067789473684	-
6777921	Kif5a	0.00296	0.421067789473684	-
6768094	Ranbp2	0.0032	0.450466666666667	+
6838671	Krt2	0.00326	0.454181855670103	-
6889258	Cd44	0.00342	0.460525631067961	-

6960404	Dbp	0.00342	0.460525631067961	-
6979919	Slc35f3	0.00342	0.460525631067961	+
6784412	Fmnl1	0.00347	0.460525631067961	+
6924387	Prpf38a	0.00348	0.460525631067961	-
6791760	Wnt9b	0.00351	0.460525631067961	-
6800233	Ifrd1	0.00361	0.469091730769231	+
6983909	Wdr83	0.0037	0.476207619047619	-
6842940	Bach1	0.00393	0.501037924528302	+
6957434	Klra5	0.0041	0.517826168224299	-
6863210	Mkx	0.00414	0.518036666666667	-
6929632	Cenpa	0.00433	0.536840550458716	-
6994705	Spa17	0.00446	0.54542990990991	-
6879915	Kif18a	0.00448	0.54542990990991	-
6775983	Slc25a3	0.00459	0.551228947368421	-
6849921	Zfp472	0.00461	0.551228947368421	-
6847238	--	0.00465	0.551228947368421	-
6770325	Slc6a15	0.0047	0.552311304347826	+
6992011	Slco2a1	0.00476	0.55454	-
6931001	Pcdh7	0.00488	0.563660854700855	-
6912989	Dnajb5	0.00496	0.56781512605042	+
6771092	Grip1	0.005	0.56781512605042	-
6899622	Selenbp1	0.00534	0.599149386954389	-
6806435	Gcnt2	0.00545	0.599149386954389	+
6906267	Glrb	0.00571	0.599149386954389	-
7018847	Itm2a	0.00572	0.599149386954389	-
6926938	Kif1b	0.0059	0.599149386954389	-
6983226	Mast3	0.006	0.599149386954389	-
6991261	Rasgrf1	0.006	0.599149386954389	-
6806997	Spin1	0.00604	0.599149386954389	-
6807876	4930441O14Rik	0.00653	0.599149386954389	-
6922471	Dbc1	0.00673	0.599149386954389	-
6762625	Lhx9	0.00678	0.599149386954389	-
6849595	Cdkn1a	0.00689	0.599149386954389	+
6833398	Zfp740	0.00697	0.599149386954389	-
6796121	Rhoj	0.00726	0.599149386954389	+
6839960	Chrd	0.00726	0.599149386954389	-
6864680	Hbegf	0.00742	0.599149386954389	+
6989010	1600029D21Rik	0.00777	0.599149386954389	-
6924841	Rnf220	0.00792	0.599149386954389	-
6813742	Ctla2b	0.00794	0.599149386954389	-
6788054	Zfp354c	0.00797	0.599149386954389	-
6971357	Zfp764	0.00802	0.599149386954389	-
6871254	Rps6ka4	0.00819	0.599149386954389	-
6843335	Cbr1	0.00841	0.599149386954389	-
6925265	Dnali1	0.00843	0.599149386954389	-
6962950	Rab6	0.00855	0.599149386954389	-
6871771	Cep78	0.00866	0.599149386954389	-
6963456	Ampd3	0.00893	0.599149386954389	+
6942524	Serpine1	0.00905	0.599149386954389	-
6806566	Edn1	0.00917	0.599149386954389	-

6756946	Slco5a1	0.0092	0.599149386954389	-
6824835	Tinf2	0.00958	0.599149386954389	+
6925547	Hpca	0.00962	0.599149386954389	-
6983058	Psd3	0.00965	0.599149386954389	-
6824838	Tgm1	0.00992	0.599149386954389	-
6805599	Prl2a1	0.01	0.599149386954389	-
6982898	Tll1	0.0101	0.599149386954389	-
6972989	Mzf1	0.0102	0.599149386954389	-
6873111	Blnk	0.0103	0.599149386954389	-
6906830	Adam15	0.0103	0.599149386954389	-
6791641	Gfap	0.0105	0.599149386954389	+
6798180	Traf3	0.0105	0.599149386954389	-
6854895	Zfp799	0.0105	0.599149386954389	+
6978513	--	0.0106	0.599149386954389	-
6926457	Fblim1	0.0109	0.599149386954389	-
6960344	Slc17a7	0.011	0.599149386954389	-
6771334	Ppm1h	0.0112	0.599149386954389	+
6949335	Cxcl12	0.0112	0.599149386954389	-
6932368	Pf4	0.0116	0.599149386954389	-
6873066	Pdlim1	0.0117	0.599149386954389	+
6923411	Jun	0.0122	0.599149386954389	+
6830154	Trhr	0.0123	0.599149386954389	-
6875662	Zmynd19	0.0123	0.599149386954389	-
6872206	1700028P14Rik	0.0126	0.599149386954389	-
6992381	Shisa5	0.0126	0.599149386954389	-
7014017	Kir3dl1	0.0128	0.599149386954389	-
6783784	Phospho1	0.0129	0.599149386954389	+
6840223	Trp63	0.0129	0.599149386954389	-
6796574	Acot5	0.0131	0.599149386954389	-
6795889	Arid4a	0.0132	0.599149386954389	-
6908745	Rwdd3	0.0132	0.599149386954389	-
6946931	Vps24	0.0132	0.599149386954389	-
6789817	Abr	0.0135	0.599149386954389	-
6882476	Chmp4b	0.0135	0.599149386954389	-
7005797	Hmgcs1	0.0136	0.599149386954389	+
6867898	Rcor2	0.0138	0.599149386954389	-
6933037	Nudt9	0.0138	0.599149386954389	-
6885937	Zbtb43	0.014	0.599149386954389	-
6997040	Lrrc1	0.0142	0.599149386954389	-
6847748	Tiam1	0.0143	0.599149386954389	-
6951974	Wnt2	0.0145	0.599149386954389	-
6986775	Trpc6	0.0145	0.599149386954389	-
6825424	Pnoc	0.0146	0.599149386954389	-
6844850	Gm606	0.0147	0.599149386954389	-
6882730	Lbp	0.0147	0.599149386954389	-
6915818	Raver2	0.0147	0.599149386954389	+
6901347	Col25a1	0.0148	0.599149386954389	-
6905297	Commd2	0.0149	0.599149386954389	-
7015255	Mid1	0.0149	0.599149386954389	-
6788274	Uqcrq	0.015	0.599149386954389	-

6874563	Fam107b	0.015	0.599149386954389	-
6974850	Eif4ebp1	0.015	0.599149386954389	+
6752523	3110009E18Rik	0.0151	0.599149386954389	-
6813518	Trpc7	0.0152	0.599149386954389	-
6833599	Fbxo4	0.0153	0.599149386954389	-
6872878	Ppp1r3c	0.0153	0.599149386954389	+
6797747	4933433P14Rik	0.0154	0.599149386954389	-
6886908	Nr4a2	0.0154	0.599149386954389	+
6954035	Snca	0.0156	0.599149386954389	-
6966058	Pld3	0.0159	0.599149386954389	-
6972317	Igf2	0.0159	0.599149386954389	-
6844549	Igf2bp2	0.016	0.599149386954389	-
6838460	Faim2	0.0162	0.599149386954389	-
6899217	Thbs3	0.0162	0.599149386954389	-
6752884	R3hdm1	0.0163	0.599149386954389	+
6826292	Pcdh8	0.0163	0.599149386954389	+
6978948	Nip7	0.0164	0.599149386954389	-
6817952	Tmem110	0.0166	0.599149386954389	-
6802181	Gm4787	0.0167	0.599149386954389	-
6899654	Sema6c	0.0167	0.599149386954389	-
6926015	Pdik1l	0.0167	0.599149386954389	-
6987262	Mbd3l1	0.0167	0.599149386954389	-
6978774	Ces2a	0.0169	0.599149386954389	-
6766046	Plagl1	0.0171	0.599149386954389	-
6836691	Arc	0.0172	0.599149386954389	+
6913499	Smc2	0.0173	0.599149386954389	-
6915504	Ift74	0.0174	0.599149386954389	-
6931961	Lphn3	0.0174	0.599149386954389	+
6964394	Tgfb1i1	0.018	0.599149386954389	+
6841019	Zbtb20	0.0182	0.599149386954389	-
6955698	Lrig1	0.0182	0.599149386954389	+
6882615	Epb4.1l1	0.0183	0.599149386954389	-
6798153	Rcor1	0.0184	0.599149386954389	-
6781989	Alox12b	0.0186	0.599149386954389	-
6748691	Vwa3b	0.0187	0.599149386954389	-
6871568	Ms4a2	0.019	0.599149386954389	-
6885873	Lcn2	0.0191	0.599149386954389	-
6831120	Efr3a	0.0192	0.599149386954389	-
6917129	Stk40	0.0194	0.599149386954389	+
6786288	Etaa1	0.0195	0.599149386954389	-
6882435	1700058C13Rik	0.0195	0.599149386954389	-
6907534	Wdr3	0.0195	0.599149386954389	-
6972110	Tubgcp2	0.0195	0.599149386954389	-
6987244	Chordc1	0.0195	0.599149386954389	-
6871543	Ms4a5	0.0197	0.599149386954389	-
6969838	P2ry2	0.0201	0.599149386954389	-
6832078	Tnrc6b	0.0202	0.599149386954389	-
6905221	Ccna1	0.0202	0.599149386954389	-
6756625	Mrpl15	0.0204	0.599149386954389	-
6859406	Dtna	0.0208	0.599149386954389	-

6983073	Csgalnact1	0.0208	0.599149386954389	+
6889452	Dnajc24	0.021	0.599149386954389	-
6811910	Prl7b1	0.0211	0.599149386954389	-
6994688	Robo3	0.0212	0.599149386954389	-
6945704	Prss2	0.0213	0.599149386954389	-
6812250	1300014I06Rik	0.0214	0.599149386954389	-
6971899	Ebf3	0.0214	0.599149386954389	-
6841200	Slc9a10	0.0216	0.599149386954389	-
6789849	Ccdc55	0.0218	0.599149386954389	-
6769448	Rfx4	0.022	0.599149386954389	+
6848368	Tiam2	0.0221	0.599149386954389	-
6781029	Acsl6	0.0227	0.599149386954389	-
6998972	Stac	0.0229	0.599149386954389	-
6780564	BC053393	0.023	0.599149386954389	-
6749455	Hibch	0.0233	0.599149386954389	-
6923850	Jak1	0.0233	0.599149386954389	-
6854276	Flywch2	0.0234	0.599149386954389	-
6762197	Cdk18	0.0235	0.599149386954389	+
6959534	Zfp84	0.0235	0.599149386954389	-
7018195	Mageb18	0.0236	0.599149386954389	-
6749734	Cflar	0.0238	0.599149386954389	-
6778282	Patz1	0.0238	0.599149386954389	-
6837467	Efcab6	0.0238	0.599149386954389	-
6858260	Mas1	0.0238	0.599149386954389	-
6766862	6330407J23Rik	0.024	0.599149386954389	-
6817393	Vcl	0.0242	0.599149386954389	+
6831891	Maff	0.0242	0.599149386954389	-
6829277	Myo10	0.0243	0.599149386954389	-
6961912	Acan	0.0243	0.599149386954389	-
6820381	Tsc22d1	0.0246	0.599149386954389	-
6884523	Celf2	0.0248	0.599149386954389	-
6791229	Plxdc1	0.0249	0.599149386954389	-
6866635	Elac1	0.0251	0.599149386954389	-
6964798	Ptpre	0.0253	0.599149386954389	-
6781975	Tmem107	0.0254	0.599149386954389	-
6840095	Hrg	0.0254	0.599149386954389	-
6833308	Grasp	0.0255	0.599149386954389	+
6892052	Abhd12	0.0255	0.599149386954389	-
6804525	Akr1c13	0.0257	0.599149386954389	-
6993845	Rab3d	0.0257	0.599149386954389	-
6814407	Cast	0.026	0.599149386954389	-
6992994	Vipr1	0.026	0.599149386954389	-
6826883	Dach1	0.0262	0.599149386954389	-
6980364	Col4a1	0.0264	0.599149386954389	+
6935327	Foxk1	0.027	0.599149386954389	-
6997037	2310046A06Rik	0.0272	0.599149386954389	-
6764694	Enah	0.0274	0.599149386954389	-
6916497	Cyp4a31	0.0274	0.599149386954389	-
6918858	Slc2a5	0.0275	0.599149386954389	-
6777915	Slc26a10	0.0279	0.599149386954389	-

6819075	A130082M07Rik	0.0279	0.599149386954389	-
6770952	Best3	0.0281	0.599149386954389	-
6838134	Pus7l	0.0284	0.599149386954389	-
6784765	Axin2	0.0285	0.599149386954389	-
6971017	Gga2	0.0285	0.599149386954389	-
6855659	Vegfa	0.0286	0.599149386954389	+
6949860	Scnn1a	0.0287	0.599149386954389	-
6780717	Zfp62	0.029	0.599149386954389	-
6857022	Tgif1	0.029	0.599149386954389	-
6801507	Trim9	0.0291	0.599149386954389	+
6854844	Sik1	0.0292	0.599149386954389	+
6869936	Scd4	0.0292	0.599149386954389	-
6849406	0610011F06Rik	0.0293	0.599149386954389	-
6776152	4932415G12Rik	0.0294	0.599149386954389	-
6942124	Stx2	0.0294	0.599149386954389	-
6862133	Smad7	0.0295	0.599149386954389	+
6824800	Jph4	0.0296	0.599149386954389	+
6830927	Myc	0.0296	0.599149386954389	-
6815599	Pik3r1	0.0297	0.599149386954389	-
6906820	Rag1ap1	0.0297	0.599149386954389	-
6762103	Zp3r	0.0298	0.599149386954389	-
6989672	Tle3	0.0299	0.599149386954389	+
6807764	Zfp369	0.03	0.599149386954389	-
6760006	Wdfy1	0.0301	0.599149386954389	+
6819046	Rpgrip1	0.0301	0.599149386954389	-
6990216	Anxa2	0.0306	0.599149386954389	-
6997114	Slc17a5	0.0307	0.599149386954389	-
6879659	Cstf3	0.0308	0.599149386954389	-
6944372	Cav1	0.0308	0.599149386954389	+
6767351	9030224M15Rik	0.0309	0.599149386954389	-
6940535	Mapk10	0.0309	0.599149386954389	-
6959600	Tmem149	0.0309	0.599149386954389	-
6910751	Tnni3k	0.031	0.599149386954389	-
6794202	Lamb1	0.0311	0.599149386954389	-
6790478	Ints2	0.0312	0.599149386954389	-
6956985	Mical3	0.0312	0.599149386954389	-
6806948	Ippk	0.0313	0.599149386954389	-
6892144	Sox12	0.0318	0.599149386954389	-
6955292	Isy1	0.032	0.599149386954389	-
6794568	Dgkb	0.0321	0.599149386954389	-
6776029	Nedd1	0.0325	0.599149386954389	-
6981108	Adam9	0.0325	0.599149386954389	-
6992374	Slc26a6	0.0325	0.599149386954389	-
6871425	Scgb1a1	0.0327	0.599149386954389	-
7018246	Zfx	0.0327	0.599149386954389	-
6836973	Myh9	0.0328	0.599149386954389	+
6982921	Sc4mol	0.0328	0.599149386954389	-
6772550	Pde7b	0.0329	0.599149386954389	-
7013205	P2ry10	0.033	0.599149386954389	-
6916483	Stil	0.0332	0.599149386954389	-

6778719	Upp1	0.0333	0.599149386954389	-
6927362	Agrn	0.0335	0.599149386954389	-
6755569	Kif26b	0.0336	0.599149386954389	+
6890395	Ell3	0.0339	0.599149386954389	-
6966003	Nlrp9c	0.0341	0.599149386954389	-
6886200	Golga1	0.0344	0.599149386954389	-
6919163	Cdk11b	0.0344	0.599149386954389	+
6968828	Cpeb1	0.0344	0.599149386954389	-
6882880	Plcg1	0.0345	0.599149386954389	-
7014929	Rbbp7	0.0345	0.599149386954389	+
6921127	Fancg	0.0347	0.599149386954389	-
6780945	Fstl4	0.0348	0.599149386954389	+
6899252	Pmvk	0.0348	0.599149386954389	+
6946759	Prdm5	0.0349	0.599149386954389	+
7012842	Kif4	0.0349	0.599149386954389	-
6802290	Numb	0.0351	0.599149386954389	-
7015012	Asb11	0.0351	0.599149386954389	-
6890837	Il1a	0.0352	0.599149386954389	-
6897593	A730037C10Rik	0.0353	0.599149386954389	-
6857799	Thada	0.0354	0.599149386954389	-
6911412	Fam110b	0.0355	0.599149386954389	-
6972712	Leng8	0.0356	0.599149386954389	-
6764140	Kcnj9	0.0358	0.599149386954389	-
6824942	Cryl1	0.0358	0.599149386954389	+
6875850	Adamtsl2	0.0361	0.599149386954389	-
6845471	Gtf2e1	0.0362	0.599149386954389	-
6844346	Tbx1	0.0364	0.599149386954389	-
6962065	Whamm	0.0365	0.599149386954389	-
6966337	Fxyd5	0.0365	0.599149386954389	-
6970378	St5	0.0367	0.599149386954389	-
6962762	Ints4	0.0368	0.599149386954389	-
6888834	Mapk8ip1	0.0369	0.599149386954389	-
6895460	Zfhx4	0.0369	0.599149386954389	-
7015697	Med14	0.0369	0.599149386954389	-
6919003	Tnfrsf25	0.0373	0.599149386954389	+
6874085	4930506M07Rik	0.0374	0.599149386954389	-
6966145	Zfp36	0.0374	0.599149386954389	-
6977547	Gypa	0.0377	0.599149386954389	-
6966972	Lin7b	0.0378	0.599149386954389	+
6874057	Gfra1	0.0379	0.599149386954389	+
6881101	Zc3h6	0.0381	0.599149386954389	+
6983038	--	0.0381	0.599149386954389	-
6824495	Slc35f4	0.0382	0.599149386954389	-
6774384	Tspan15	0.0384	0.599149386954389	-
6769241	Izumo4	0.0386	0.599149386954389	-
6956045	Cntn3	0.0386	0.599149386954389	+
6963267	Olfml1	0.0386	0.599149386954389	-
6854420	Cacna1h	0.0389	0.599149386954389	-
6978156	Rbl2	0.0389	0.599149386954389	-
6786965	Eml6	0.039	0.599149386954389	-

6963213	Ilk	0.039	0.599149386954389	-
6793370	Smc6	0.0391	0.599149386954389	-
6882612	4921517L17Rik	0.0392	0.599149386954389	-
6929881	Adra2c	0.0394	0.599149386954389	-
6819499	Rcbtb1	0.0395	0.599149386954389	-
6838645	Krt84	0.0395	0.599149386954389	-
6979706	Def8	0.0395	0.599149386954389	-
6998213	Esyt3	0.0395	0.599149386954389	-
6762132	Mapkapk2	0.0398	0.599149386954389	+
6814666	Polr3g	0.0399	0.599149386954389	-
6902623	Slc44a5	0.0399	0.599149386954389	-
6951440	Slc25a13	0.0399	0.599149386954389	-
6785399	Baiap2	0.04	0.599149386954389	-
6899014	Etv3	0.04	0.599149386954389	+
6876543	Nek6	0.0403	0.599149386954389	-
6780844	Zfp354a	0.0407	0.599149386954389	-
6886957	Acvr1	0.0407	0.599149386954389	+
6757927	Ankrd23	0.0408	0.599149386954389	-
6788064	Zfp454	0.0409	0.599149386954389	-
6849525	Anks1	0.0409	0.599149386954389	-
6970568	Btbd10	0.0409	0.599149386954389	-
6921211	Rnf38	0.041	0.599149386954389	-
6748897	Slc9a4	0.0412	0.599149386954389	+
6998603	Dock3	0.0412	0.599149386954389	+
6773020	Rspo3	0.0417	0.599149386954389	+
6875933	Olfm1	0.0419	0.599149386954389	-
6992467	Prss44	0.0419	0.599149386954389	-
6840877	B4galt4	0.0425	0.599149386954389	-
6882622	Dlgap4	0.0425	0.599149386954389	-
6937315	Tnip2	0.0426	0.599149386954389	-
6987358	Pde4a	0.0426	0.599149386954389	-
6854256	Zfp13	0.0427	0.599149386954389	-
6861722	Psmg2	0.0427	0.599149386954389	-
6903753	Ect2	0.0427	0.599149386954389	-
6917630	Ubxn11	0.0427	0.599149386954389	-
6990418	Prtg	0.0427	0.599149386954389	-
6792787	Sirt7	0.0428	0.599149386954389	-
6860778	Dmxl1	0.0428	0.599149386954389	+
6766368	Map3k5	0.043	0.599149386954389	+
6813776	Ctsm	0.0431	0.599149386954389	-
6954529	Rpia	0.0431	0.599149386954389	-
7020675	Zrsr2	0.0431	0.599149386954389	-
6778992	Wdr92	0.0432	0.599149386954389	-
6792784	Thoc4	0.0434	0.599149386954389	-
6875720	Npdc1	0.0434	0.599149386954389	-
6929644	Dpysl5	0.0434	0.599149386954389	+
6987132	Taf1d	0.0434	0.599149386954389	-
6829659	Matn2	0.0435	0.599149386954389	-
6871077	Rab1b	0.0435	0.599149386954389	-
6929719	Fosl2	0.0435	0.599149386954389	+

6781248	Igtp	0.0436	0.599149386954389	-
6887297	Slc38a11	0.0438	0.599149386954389	-
7018531	Il2rg	0.0438	0.599149386954389	-
6828862	Rnasen	0.0441	0.599149386954389	-
6824967	Lats2	0.0443	0.599149386954389	-
6769635	Sycp3	0.0444	0.599149386954389	-
6922317	Tnfsf15	0.0444	0.599149386954389	-
6942192	Gusb	0.0444	0.599149386954389	-
6956570	Prrt3	0.0445	0.599149386954389	-
6919190	Mxra8	0.0446	0.599149386954389	-
6786674	Papolg	0.0448	0.599149386954389	-
6831164	Wisp1	0.0448	0.599149386954389	-
6890935	Gm14057	0.0448	0.599149386954389	-
6891992	Zfp442	0.0448	0.599149386954389	-
6934605	Sfswap	0.0448	0.599149386954389	-
7011910	Zfp185	0.0448	0.599149386954389	-
6845444	Slc15a2	0.0452	0.599149386954389	-
6780767	Gfpt2	0.0453	0.599149386954389	-
6899887	Wars2	0.0453	0.599149386954389	-
6948274	Kbtbd8	0.0454	0.599149386954389	-
7015941	Slc9a7	0.0454	0.599149386954389	-
7015894	4930578C19Rik	0.0456	0.599149386954389	-
6924894	Tie1	0.0457	0.599149386954389	-
6971649	Cpxm2	0.0458	0.599149386954389	-
6928695	4921511H03Rik	0.0459	0.599149386954389	-
6928939	Hgf	0.0459	0.599149386954389	-
6939960	Rchy1	0.0461	0.599149386954389	-
6755125	Olfml2b	0.0462	0.599149386954389	+
6966887	Myh14	0.0462	0.599149386954389	-
6783685	Col1a1	0.0463	0.599149386954389	-
6870193	Sorcs3	0.0463	0.599149386954389	+
6803912	Cdca4	0.0465	0.599149386954389	-
6897337	Ccrn4l	0.0465	0.599149386954389	+
6826266	Naa16	0.0468	0.599149386954389	-
6969886	Inppl1	0.0468	0.599149386954389	-
6933800	Nos1	0.0469	0.599149386954389	-
6837131	Cbx6	0.047	0.599149386954389	-
6848966	Ncrna00085	0.0472	0.599149386954389	-
6758737	Sf3b1	0.0474	0.599149386954389	-
6893279	Nfatc2	0.0474	0.599149386954389	-
6850097	Clic1	0.0475	0.599149386954389	-
6892940	Wfdc16	0.0476	0.599149386954389	-
6924872	Dph2	0.0476	0.599149386954389	-
6958939	Ceacam14	0.0476	0.599149386954389	-
7009834	Slc38a5	0.0476	0.599149386954389	-
6925654	Serinc2	0.0477	0.599149386954389	+
6763011	1200016B10Rik	0.0479	0.599149386954389	-
6977775	Dnase2a	0.0481	0.599149386954389	-
6985642	Cdyl2	0.0481	0.599149386954389	+
6816967	3830406C13Rik	0.0482	0.599149386954389	-

6975052	Rnf122	0.0482	0.599149386954389	-
6869889	Cutc	0.0483	0.599149386954389	-
6971700	Mettl10	0.0487	0.599149386954389	-
6785709	Kremen1	0.0489	0.599149386954389	-
6949613	A2m	0.0489	0.599149386954389	-
6781306	Trim17	0.0491	0.599149386954389	-
6956909	Lrtm2	0.0491	0.599149386954389	+
7012341	1700072E05Rik	0.0491	0.599149386954389	-
6920736	1700003M02Rik	0.0492	0.599149386954389	-
6887853	Chrna1	0.0494	0.599149386954389	-
6946033	Gimap1	0.0494	0.599149386954389	-
6998628	Cyb561d2	0.0494	0.599149386954389	-
6837328	Pmm1	0.0496	0.599149386954389	-
6869577	Hells	0.0496	0.599149386954389	-
6833640	Ptger4	0.0497	0.599149386954389	-
6909304	Gar1	0.0497	0.599149386954389	-
6783063	Acaca	0.05	0.599149386954389	-
6807252	B4galt7	0.05	0.599149386954389	-
6784606	Psmc5	0.0503	0.599149386954389	-
6947321	AW146020	0.0504	0.599149386954389	-
6949760	Vmn2r26	0.0504	0.599149386954389	-
6970350	Lmo1	0.0504	0.599149386954389	-
6797585	Serpina3f	0.0505	0.599149386954389	-
6832395	Trmu	0.0505	0.599149386954389	-
6879070	Arhgap1	0.0505	0.599149386954389	-
6929279	Nupl2	0.0506	0.599149386954389	-
6823891	Ldb3	0.0508	0.599149386954389	-
6899741	Mtmr11	0.0508	0.599149386954389	-
6915791	Pgm2	0.0508	0.599149386954389	-
6825192	Tnfrsf19	0.051	0.599149386954389	-
6769244	Dot1l	0.0511	0.599149386954389	+
6785936	Sun3	0.0511	0.599149386954389	-
6951899	--	0.0511	0.599149386954389	-
6818557	Styx	0.0512	0.599149386954389	-
6989752	Itga11	0.0513	0.599149386954389	-
6784526	Myl4	0.0514	0.599149386954389	-
6805206	Zscan12	0.0514	0.599149386954389	-
6978340	Coq9	0.0514	0.599149386954389	-
6785369	Slc26a11	0.0519	0.599149386954389	-
6840556	Tm4sf19	0.0519	0.599149386954389	-
6866653	Mapk4	0.0519	0.599149386954389	+
6958955	Psg22	0.0519	0.599149386954389	-
6850701	Crip3	0.0521	0.599149386954389	-
6899694	Mcl1	0.0521	0.599149386954389	+
6996444	Lactb	0.0523	0.599149386954389	-
6835933	Zhx1	0.0524	0.599149386954389	-
6867947	Slc22a8	0.0524	0.599149386954389	+
6775430	Pias4	0.0525	0.599149386954389	-
6854864	Notch3	0.0526	0.599149386954389	-
6973169	Bcl3	0.0526	0.599149386954389	-

6967028	Ush1c	0.0527	0.599149386954389	-
6760417	Ecel1	0.0528	0.599149386954389	-
6921029	Kif24	0.0528	0.599149386954389	-
6816161	Gzmk	0.0532	0.599149386954389	-
6815749	Rnf180	0.0533	0.599149386954389	-
6782288	P2rx5	0.0534	0.599149386954389	-
6870989	Ssh3	0.0534	0.599149386954389	-
6964349	Orai3	0.0535	0.599149386954389	+
6917577	Map3k6	0.0536	0.599149386954389	+
6949790	C1rb	0.0536	0.599149386954389	-
6876207	Pip5kl1	0.0537	0.599149386954389	-
7020407	Phex	0.0537	0.599149386954389	-
6847090	Robo2	0.0539	0.599149386954389	-
6830174	A930017M01Rik	0.0542	0.599149386954389	-
6775561	Aldh1l2	0.0543	0.599149386954389	-
6803070	2610021K21Rik	0.0543	0.599149386954389	-
6934962	Dtx2	0.0543	0.599149386954389	-
6795977	Lrrc9	0.0544	0.599149386954389	-
6857523	Srsf7	0.0544	0.599149386954389	+
6785261	Sept9	0.0545	0.599149386954389	-
6832532	Pim3	0.0545	0.599149386954389	+
6897441	Foxo1	0.0546	0.599149386954389	+
6987381	Qtrt1	0.0547	0.599149386954389	-
6787525	Gabrg2	0.0548	0.599149386954389	-
6849433	Mrpl28	0.0548	0.599149386954389	-
6892180	Bcl2l1	0.0549	0.599149386954389	-
6788563	Gemin5	0.055	0.599149386954389	-
6949737	Clec4a2	0.055	0.599149386954389	-
6846375	Nfkbiz	0.0552	0.599149386954389	+
6968643	Ntrk3	0.0553	0.599149386954389	-
6971281	Giyd2	0.0553	0.599149386954389	-
6971490	Fgfr2	0.0554	0.599149386954389	-
6925575	Lck	0.0555	0.599149386954389	-
6935502	Zfp655	0.0555	0.599149386954389	+
6955066	Paip2b	0.0558	0.599149386954389	-
6966954	Ccdc155	0.0559	0.599149386954389	-
6981185	4930444A02Rik	0.0559	0.599149386954389	-
6983825	Cc2d1a	0.0559	0.599149386954389	-
6894311	Rgs19	0.056	0.599149386954389	-
6946002	Sspo	0.0563	0.599149386954389	-
6988606	C1qtnf5	0.0563	0.599149386954389	-
6832540	Trabd	0.0564	0.599149386954389	-
6792813	Dcxr	0.0566	0.599149386954389	-
6812178	Serpinb1a	0.0567	0.599149386954389	-
6935970	Cdk14	0.0567	0.599149386954389	+
6952950	Dennd2a	0.0568	0.599149386954389	-
6913316	Stx17	0.0571	0.599149386954389	-
6849342	Clcn7	0.0572	0.599149386954389	-
6876423	Mrrf	0.0572	0.599149386954389	-
6781322	2310033P09Rik	0.0573	0.599149386954389	-

6861576	Nedd4l	0.0573	0.599149386954389	-
6991561	Gk5	0.0574	0.599149386954389	-
6921157	Sit1	0.0575	0.599149386954389	-
6849068	Zfp54	0.0576	0.599149386954389	-
6936690	Pus7	0.0576	0.599149386954389	-
6885326	Tbpl2	0.0577	0.599149386954389	-
6846571	Crybg3	0.0578	0.599149386954389	-
6871361	Slc3a2	0.0579	0.599149386954389	-
6784591	Taco1	0.058	0.599149386954389	-
6796303	Fam71d	0.058	0.599149386954389	-
6912113	Fbxl4	0.058	0.599149386954389	-
6880718	Sqrdl	0.0581	0.599149386954389	-
6988728	Fxyd2	0.0581	0.599149386954389	-
6942417	Vps37d	0.0582	0.599149386954389	-
6791418	Hap1	0.0584	0.599149386954389	+
6941183	Selplg	0.0585	0.599149386954389	-
6900254	Dennd2d	0.0588	0.599149386954389	-
6973238	Rdh13	0.0588	0.599149386954389	-
6959093	Zfp112	0.0591	0.599149386954389	-
6782037	Fxr2	0.0592	0.599149386954389	-
6797772	Vrk1	0.0592	0.599149386954389	-
6854425	Sox8	0.0592	0.599149386954389	-
6902618	Asb17	0.0592	0.599149386954389	-
6786469	Cep68	0.0596	0.599149386954389	-
6854314	Kctd5	0.0596	0.599149386954389	-
6854463	Nme4	0.0596	0.599149386954389	-
6803891	Tmem179	0.0597	0.599149386954389	-
6763903	Mgst3	0.0598	0.599149386954389	-
6936573	Fbxl13	0.0599	0.599149386954389	-
6933973	Slc24a6	0.06	0.599149386954389	-
6962767	Rsf1	0.0601	0.599149386954389	-
6945321	Akr1b7	0.0602	0.599149386954389	-
6866511	Poli	0.0605	0.599149386954389	-
6925988	Ccdc21	0.0607	0.599149386954389	-
6937986	Kcnip4	0.0607	0.599149386954389	-
6890926	Prosapip1	0.0608	0.599149386954389	-
6838808	Zfp385a	0.0609	0.599149386954389	-
6940366	Helq	0.0609	0.599149386954389	-
6751535	Ramp1	0.061	0.599149386954389	-
6792493	Wbp2	0.061	0.599149386954389	-
6877962	4933404M02Rik	0.061	0.599149386954389	-
6957106	Pex5	0.061	0.599149386954389	-
6959585	Nfkbid	0.0612	0.599149386954389	-
6855705	Cul9	0.0613	0.599149386954389	-
6764953	Tgfb2	0.0614	0.599149386954389	-
6807998	Nsun2	0.0614	0.599149386954389	-
6833243	Letmd1	0.0615	0.599149386954389	-
6905192	Exosc8	0.0615	0.599149386954389	-
6963004	Arap1	0.0615	0.599149386954389	-
6905296	Wwtr1	0.0616	0.599149386954389	+

6947392	Ccdc142	0.0616	0.599149386954389	-
6814039	Zfp459	0.0618	0.599149386954389	-
6976233	Glra3	0.0618	0.599149386954389	-
6892512	Ghrh	0.0619	0.599149386954389	-
6804898	Lyst	0.062	0.599149386954389	+
6987439	Cnn1	0.062	0.599149386954389	-
6992367	Prkar2a	0.062	0.599149386954389	+
6993726	S1pr2	0.0621	0.599149386954389	-
6878720	Prg3	0.0622	0.599149386954389	-
6962921	Lipt2	0.0623	0.599149386954389	-
6777917	D10Ertd610e	0.0624	0.599149386954389	-
6932164	Csn1s1	0.0624	0.599149386954389	-
6802098	Actn1	0.0625	0.599149386954389	-
6916504	Atpaf1	0.0626	0.599149386954389	-
6931844	Arl9	0.0626	0.599149386954389	-
6803120	Ccdc88c	0.0627	0.599149386954389	-
6949885	Ano2	0.0627	0.599149386954389	-
6972153	Bet1l	0.0628	0.599149386954389	-
6999097	Fbxl2	0.063	0.599149386954389	-
6820323	5031414D18Rik	0.0631	0.599149386954389	-
6988855	Cadm1	0.0634	0.599149386954389	-
6823659	Cacna1d	0.0635	0.599149386954389	-
6912022	Fam82b	0.0635	0.599149386954389	-
6818667	Samd4	0.0636	0.599149386954389	+
6822367	Itgbl1	0.0636	0.599149386954389	+
6837114	Dmc1	0.0638	0.599149386954389	-
6869597	Cyp2c50	0.0639	0.599149386954389	-
6878548	Dusp19	0.0639	0.599149386954389	-
6830026	Zfpm2	0.0641	0.599149386954389	-
6971758	Adam12	0.0641	0.599149386954389	-
6872489	4430402I18Rik	0.0643	0.599149386954389	-
6915506	Tek	0.0643	0.599149386954389	-
6961201	Snrpa1	0.0643	0.599149386954389	+
6765460	Cr2	0.0644	0.599149386954389	-
6854604	Fkbp5	0.0644	0.599149386954389	-
6891096	Gpcpd1	0.0644	0.599149386954389	-
6995068	Hinfp	0.0645	0.599149386954389	-
6905818	Ift80	0.0646	0.599149386954389	-
6848018	Setd4	0.0647	0.599149386954389	-
6775389	Mknk2	0.0648	0.599149386954389	+
6857873	Six3os1	0.0648	0.599149386954389	-
6781111	Slc36a1	0.0651	0.599149386954389	-
6959536	Zfp30	0.0651	0.599149386954389	-
6991192	Nt5e	0.0652	0.599149386954389	-
6858912	Cabyr	0.0653	0.599149386954389	-
6763537	Prdx6	0.0656	0.599149386954389	-
6775391	Mobkl2a	0.0656	0.599149386954389	-
6868180	Gm4952	0.0656	0.599149386954389	-
6869573	Tbc1d12	0.0656	0.599149386954389	-
6838287	Slc38a4	0.0657	0.599149386954389	-

6900306	Slc16a4	0.0657	0.599149386954389	-
6792079	Rgs9	0.0658	0.599149386954389	-
6888307	Ctnnd1	0.0659	0.599149386954389	+
6946009	Lrrc61	0.0659	0.599149386954389	-
6829068	Cdh10	0.066	0.599149386954389	-
6749444	Tmem194b	0.0661	0.599149386954389	-
6987384	AB124611	0.0661	0.599149386954389	-
6982100	Fam149a	0.0662	0.599149386954389	-
6941768	Kdm2b	0.0666	0.599149386954389	-
6955539	Wnt7a	0.0666	0.599149386954389	-
6952818	Atp6v0a4	0.0667	0.599149386954389	-
6798285	Pld4	0.0669	0.599149386954389	-
6869656	Ccnj	0.067	0.599149386954389	-
6806300	Snrnp48	0.0672	0.599149386954389	-
6868098	Ms4a6c	0.0672	0.599149386954389	-
6964086	Lcmt1	0.0673	0.599149386954389	-
6788025	Rufy1	0.0675	0.599149386954389	-
6749103	Bivm	0.0676	0.599149386954389	-
6877441	Tanc1	0.0676	0.599149386954389	-
6902201	Ctbs	0.0676	0.599149386954389	-
6918131	Mfap2	0.0676	0.599149386954389	-
6979836	Gnpat	0.0676	0.599149386954389	-
6968871	Hdgfrp3	0.0678	0.599149386954389	-
6896835	4932438A13Rik	0.068	0.599149386954389	-
6917551	Ahdc1	0.0681	0.599149386954389	-
6996646	Ccnb2	0.0681	0.599149386954389	-
6795794	Atp5s	0.0682	0.599149386954389	+
6947404	Bola3	0.0682	0.599149386954389	-
6844253	Sdf2l1	0.0685	0.599149386954389	-
6873061	Cyp2c68	0.0685	0.599149386954389	-
6837455	Scube1	0.0686	0.599149386954389	+
6836843	Cyhr1	0.0688	0.599149386954389	-
6764650	Ephx1	0.0689	0.599149386954389	-
6838669	Krt73	0.0689	0.599149386954389	-
6851320	Trip10	0.0689	0.599149386954389	+
7017052	Arhgef6	0.0689	0.599149386954389	-
6802139	Gm1568	0.069	0.599149386954389	-
6832018	Tnrc6b	0.069	0.599149386954389	-
6855396	Gpr115	0.0691	0.599149386954389	-
6750549	Vil1	0.0692	0.599149386954389	-
6780995	Il5	0.0692	0.599149386954389	-
6900982	Abca4	0.0693	0.599149386954389	-
6987893	Nfrkb	0.0694	0.599149386954389	-
6870925	Gal	0.0698	0.599149386954389	-
6936719	Kcnh2	0.0698	0.599149386954389	-
6884352	Hspa14	0.0699	0.599149386954389	+
6855756	1700001C19Rik	0.07	0.599149386954389	-
6980107	Cd209c	0.07	0.599149386954389	-
6819258	Irf9	0.0701	0.599149386954389	-
6797478	Rin3	0.0703	0.599149386954389	-

6851200	Mpnd	0.0703	0.599149386954389	-
6898686	Pdgfc	0.0703	0.599149386954389	-
6817970	Nt5dc2	0.0704	0.599149386954389	-
6906673	Pear1	0.0704	0.599149386954389	-
7020880	4933400A11Rik	0.0705	0.599149386954389	-
6812983	Tpmt	0.0706	0.599149386954389	-
6951253	4833442J19Rik	0.0709	0.599149386954389	-
6785642	Sec14l2	0.0712	0.599149386954389	-
6935176	Sun1	0.0712	0.599149386954389	-
6751338	Gigyf2	0.0714	0.599149386954389	-
6876023	Slc27a4	0.0714	0.599149386954389	-
6753185	Plekha6	0.0715	0.599149386954389	-
6833516	Pde1b	0.0715	0.599149386954389	-
6959124	Plaur	0.0717	0.599149386954389	-
6775136	AI646023	0.072	0.599149386954389	-
6977661	Clgn	0.072	0.599149386954389	-
6785163	2310067B10Rik	0.0721	0.599149386954389	-
6810209	Mier3	0.0721	0.599149386954389	-
6806991	Fbxw17	0.0722	0.599149386954389	-
6952500	Podxl	0.0723	0.599149386954389	-
6966339	Fxyd1	0.0723	0.599149386954389	-
6816226	Fst	0.0724	0.599149386954389	-
7019615	Tceal5	0.0724	0.599149386954389	-
6882350	BC020535	0.0729	0.599149386954389	-
6783689	Ppp1r9b	0.073	0.599149386954389	-
6838557	Slc11a2	0.073	0.599149386954389	-
6957144	Lag3	0.073	0.599149386954389	-
6844278	Thap7	0.0731	0.599149386954389	-
6852215	Slc30a6	0.0731	0.599149386954389	-
6909648	Nfkb1	0.0731	0.599149386954389	-
6946055	Gpnmb	0.0731	0.599149386954389	+
6762234	Cntn2	0.0734	0.599149386954389	-
6828697	Agxt2	0.0734	0.599149386954389	-
6899919	Spag17-ps	0.0734	0.599149386954389	-
6990327	Aldh1a2	0.0734	0.599149386954389	-
6816215	A430090L17Rik	0.0735	0.599149386954389	-
6766250	Txlnb	0.0736	0.599149386954389	-
6806203	Cdyl	0.0737	0.599149386954389	-
6966721	AW146154	0.0737	0.599149386954389	-
6932571	Cxcl13	0.0739	0.599149386954389	-
6979053	Pkd1l3	0.0739	0.599149386954389	-
6775175	Pcnt	0.074	0.599149386954389	-
6801491	Cdkl1	0.0742	0.599149386954389	-
6866856	Slc14a2	0.0744	0.599149386954389	-
6923402	4930579C15Rik	0.0744	0.599149386954389	-
6993098	Kif15	0.0744	0.599149386954389	-
6903560	Agtr1b	0.0746	0.599149386954389	-
6981823	Msr1	0.0746	0.599149386954389	-
6849536	Ppard	0.0747	0.599149386954389	-
6753937	Tpr	0.0748	0.599149386954389	-

6982585	Wdr17	0.0748	0.599149386954389	+
6910126	Clca2	0.0749	0.599149386954389	-
6924824	Urod	0.075	0.599149386954389	-
6882669	Manbal	0.0751	0.599149386954389	-
6904340	Nudt6	0.0751	0.599149386954389	-
6934885	Wbscr27	0.0751	0.599149386954389	-
6872426	D19Bwg1357e	0.0752	0.599149386954389	-
6759789	Ptprn	0.0754	0.599149386954389	-
6804670	Adarb2	0.0755	0.599149386954389	-
6835634	Eif3h	0.0755	0.599149386954389	-
6845933	Sidt1	0.0755	0.599149386954389	+
6808211	Lpcat1	0.0756	0.599149386954389	-
6791965	Ddx5	0.0757	0.599149386954389	+
6798288	BC022687	0.0758	0.599149386954389	-
6803946	Srsf10	0.0758	0.599149386954389	-
6926087	Grhl3	0.0759	0.599149386954389	-
7020676	Car5b	0.0759	0.599149386954389	-
6938891	Atp8a1	0.0762	0.599149386954389	-
6795024	G2e3	0.0763	0.599149386954389	-
6875920	Col5a1	0.0763	0.599149386954389	-
6761285	Serpinb3b	0.0764	0.599149386954389	-
6774907	Bicc1	0.0764	0.599149386954389	-
6784761	Ccdc46	0.0764	0.599149386954389	-
6875132	Vim	0.0764	0.599149386954389	+
6968408	St8sia2	0.0764	0.599149386954389	-
6762017	Mcm6	0.0765	0.599149386954389	-
6900409	Clcc1	0.0765	0.599149386954389	-
6933619	4930519G04Rik	0.0766	0.599149386954389	-
6935069	Gigyf1	0.0766	0.599149386954389	-
6749937	Fastkd2	0.0767	0.599149386954389	-
6822784	Thoc7	0.0767	0.599149386954389	-
6893558	Pmepa1	0.0767	0.599149386954389	+
7011944	Slc6a8	0.0767	0.599149386954389	+
6869370	Kif20b	0.0768	0.599149386954389	-
6942685	Micall2	0.0768	0.599149386954389	-
6765533	Syne1	0.0769	0.599149386954389	-
6936587	Slc26a5	0.0769	0.599149386954389	-
6823716	Dnahc1	0.077	0.599149386954389	-
6856756	Ptprm	0.0771	0.599149386954389	-
6927277	Mmp23	0.0771	0.599149386954389	-
6942600	Zfp113	0.0771	0.599149386954389	-
6972202	Pddc1	0.0771	0.599149386954389	-
6950762	Pyroxd1	0.0773	0.599149386954389	-
6958147	Sox5	0.0773	0.599149386954389	-
6918158	Epha2	0.0777	0.599149386954389	-
6984904	Cmtm2a	0.0778	0.599149386954389	-
6903779	Fndc3b	0.0779	0.599149386954389	-
6778284	Pik3ip1	0.078	0.599149386954389	+
6882995	Sgk2	0.0781	0.599149386954389	-
6904066	Gnb4	0.0781	0.599149386954389	-

6924952	AU022252	0.0781	0.599149386954389	-
6751684	Sned1	0.0782	0.599149386954389	-
6803223	Serpina1c	0.0782	0.599149386954389	-
6811914	Prl7a1	0.0783	0.599149386954389	-
6901962	Ccbl2	0.0783	0.599149386954389	-
6967969	Lrrk1	0.0783	0.599149386954389	-
6964517	Tacc2	0.0784	0.599149386954389	-
6779961	Lcp2	0.0785	0.599149386954389	-
6992387	Plxnb1	0.0786	0.599149386954389	-
6934861	Gtf2ird2	0.0789	0.599149386954389	-
6959099	Zfp93	0.0789	0.599149386954389	-
6963955	Vwa3a	0.0789	0.599149386954389	-
6973686	Pnpla6	0.0789	0.599149386954389	-
6952941	Mkrn1	0.0791	0.599149386954389	-
6832201	Cyp2d12	0.0792	0.599149386954389	-
6887522	Abcb11	0.0792	0.599149386954389	-
6946412	Adcyap1r1	0.0792	0.599149386954389	+
7019746	Serpina7	0.0792	0.599149386954389	-
6888334	Rtn4rl2	0.0793	0.599149386954389	+
7011039	Utp14a	0.0793	0.599149386954389	-
6789419	Pelp1	0.0795	0.599149386954389	-
6924585	Skint9	0.0796	0.599149386954389	-
6990215	Narg2	0.0796	0.599149386954389	-
7007379	Iah1	0.0797	0.599149386954389	-
6769245	Sf3a2	0.08	0.599149386954389	-
6782708	Ift20	0.08	0.599149386954389	-
6948649	Gxylt2	0.08	0.599149386954389	-
6950413	Emp1	0.0801	0.599149386954389	+
6973067	Zfp444	0.0801	0.599149386954389	-
6813934	Nlrp4f	0.0802	0.599149386954389	-
6882991	Srsf6	0.0802	0.599149386954389	-
6961917	Fanci	0.0803	0.599149386954389	-
6967140	Mrgprx1	0.0803	0.599149386954389	-
6855825	A530064D06Rik	0.0805	0.599149386954389	-
6996356	Plekho2	0.0805	0.599149386954389	-
6861747	Cep192	0.0808	0.599149386954389	-
6790500	Rps6kb1	0.081	0.599149386954389	-
7018524	Slc7a3	0.081	0.599149386954389	-
6913561	Fsd1l	0.0811	0.599149386954389	-
6816371	Mrps30	0.0812	0.599149386954389	-
6850637	Aars2	0.0812	0.599149386954389	-
6909555	Tet2	0.0813	0.599149386954389	+
6937792	Fbxl5	0.0813	0.599149386954389	-
6840400	Hes1	0.0814	0.599149386954389	-
6764246	Mnda	0.0816	0.599149386954389	-
6831469	Ptp4a3	0.0816	0.599149386954389	-
6963967	Polr3e	0.0816	0.599149386954389	-
6759773	Slc23a3	0.0817	0.599149386954389	-
6773633	Aim1	0.0817	0.599149386954389	-
6782979	Slfn5	0.0817	0.599149386954389	-

6850090	Lsm2	0.0818	0.599149386954389	-
6866289	Mppe1	0.0818	0.599149386954389	-
6899111	Paqr6	0.0819	0.599149386954389	+
6851322	Vav1	0.082	0.599149386954389	-
6755713	Lefty1	0.0823	0.599149386954389	+
6792903	B3gntl1	0.0823	0.599149386954389	-
6918720	Srm	0.0823	0.599149386954389	-
6973283	Zscan22	0.0823	0.599149386954389	-
6783359	Srsf1	0.0824	0.599149386954389	-
6941786	Hpd	0.0824	0.599149386954389	-
6977927	4933402J07Rik	0.0824	0.599149386954389	-
7014222	Tmem164	0.0824	0.599149386954389	-
6987046	Mtmr2	0.0825	0.599149386954389	-
6803028	Ptpn21	0.0826	0.599149386954389	-
6952167	Iqub	0.0827	0.599149386954389	-
6805180	Aoah	0.0829	0.599149386954389	-
6965195	Taldo1	0.083	0.599149386954389	-
6869583	Cyp2c39	0.0831	0.599149386954389	-
6890290	Pla2g4e	0.0832	0.599149386954389	-
6768089	Gcc2	0.0834	0.599149386954389	-
6788632	Zfp39	0.0834	0.599149386954389	+
6848806	1600012H06Rik	0.0835	0.599149386954389	-
6940347	Lin54	0.0835	0.599149386954389	-
6787531	Gabra6	0.0836	0.599149386954389	-
6846447	2310005G13Rik	0.0836	0.599149386954389	-
6870991	Ankrd13d	0.0837	0.599149386954389	-
6994633	D730048I06Rik	0.0837	0.599149386954389	-
6897557	Postn	0.0838	0.599149386954389	-
6993835	Dock6	0.0838	0.599149386954389	-
6869570	Plce1	0.0839	0.599149386954389	-
6833531	Ghr	0.084	0.599149386954389	-
6760917	Pam	0.0841	0.599149386954389	-
6884129	Ss18l1	0.0841	0.599149386954389	-
6959278	Cyp2a12	0.0844	0.599149386954389	-
6985877	Fbxo31	0.0844	0.599149386954389	-
6772098	9130014G24Rik	0.0845	0.599149386954389	-
6829591	Sema5a	0.0845	0.599149386954389	+
6848479	Zdhhc14	0.0845	0.599149386954389	-
6807687	Habp4	0.0846	0.599149386954389	-
6869766	Ubtd1	0.0846	0.599149386954389	+
6970005	Hbb-bh1	0.0846	0.599149386954389	-
6781960	Slc25a35	0.0847	0.599149386954389	+
6809083	Wdr41	0.0847	0.599149386954389	-
6799874	Hbp1	0.0849	0.599149386954389	+
6803136	Atxn3	0.0849	0.599149386954389	-
6987865	Zbtb44	0.0849	0.599149386954389	+
6839788	Dgcr6	0.0851	0.599149386954389	-
6871374	Taf6l	0.0851	0.599149386954389	-
6954638	Tgoln1	0.0851	0.599149386954389	-
6884184	Col9a3	0.0853	0.599149386954389	-

7009120	Obox5	0.0853	0.599149386954389	-
6822443	Dnase1l3	0.0854	0.599149386954389	-
6927238	Plch2	0.0854	0.599149386954389	-
6873893	Gucy2g	0.0855	0.599149386954389	-
6961987	Prc1	0.0857	0.599149386954389	-
6971260	Lat	0.0857	0.599149386954389	-
6997349	Hmgn3	0.0857	0.599149386954389	-
6863417	6030446N20Rik	0.086	0.599149386954389	-
6966039	Ltbp4	0.0861	0.599149386954389	-
6996440	Rab8b	0.0863	0.599149386954389	+
6799842	Slc26a4	0.0865	0.599149386954389	-
6861137	1700065I17Rik	0.0865	0.599149386954389	-
6771920	Mthfd1l	0.0866	0.599149386954389	-
6838746	Map3k12	0.0866	0.599149386954389	-
6900346	Eps8l3	0.0866	0.599149386954389	-
6792739	2410002I01Rik	0.0869	0.599149386954389	-
6831982	Syngr1	0.0869	0.599149386954389	-
6819252	Psme1	0.0871	0.599149386954389	-
6856122	Sult1c1	0.0871	0.599149386954389	-
6785468	Aspscr1	0.0872	0.599149386954389	-
6996451	Tln2	0.0873	0.599149386954389	-
6797476	Slc24a4	0.0874	0.599149386954389	+
6916493	Cyp4a12b	0.0874	0.599149386954389	-
6890648	2010106G01Rik	0.0875	0.599149386954389	-
6927291	Atad3a	0.0876	0.599149386954389	-
6783640	Spag9	0.0878	0.599149386954389	-
6996992	Hcrtr2	0.0879	0.599149386954389	-
6841882	Mina	0.088	0.599149386954389	-
6818136	Ptpn20	0.0881	0.599149386954389	-
6854735	Btbd9	0.0881	0.599149386954389	-
6977078	Gtpbp3	0.0881	0.599149386954389	-
6862744	Wdr53	0.0882	0.599149386954389	-
6968200	Nr2f2	0.0882	0.599149386954389	-
6803780	Cinp	0.0883	0.599149386954389	+
6811367	Txndc3	0.0884	0.599149386954389	-
6868176	Fam111a	0.0884	0.599149386954389	-
6957462	Magohb	0.0884	0.599149386954389	-
7019518	Btk	0.0887	0.599149386954389	-
6838580	Galnt6	0.0888	0.599149386954389	-
6793961	Cmpk2	0.0889	0.599149386954389	-
6993587	Naalad2	0.0889	0.599149386954389	-
6857483	Cyp1b1	0.0891	0.599149386954389	-
6934216	Setd1b	0.0892	0.599149386954389	-
6770905	Ptprr	0.0896	0.599149386954389	+
6992225	Nat6	0.0897	0.599149386954389	-
6842436	Rbm11	0.0899	0.599149386954389	+
6850070	Dom3z	0.0899	0.599149386954389	-
6853762	Map3k4	0.09	0.599149386954389	-
6899861	Notch2	0.09	0.599149386954389	-
6992378	Col7a1	0.0901	0.599149386954389	-

6993711	Olfm2	0.0901	0.599149386954389	-
6899039	Nes	0.0902	0.599149386954389	-
6858938	Hrh4	0.0904	0.599149386954389	-
6768853	Adora2a	0.0905	0.599149386954389	-
7015994	Zfp182	0.0905	0.599149386954389	-
6970053	Trim12a	0.0906	0.599149386954389	-
6788930	Zfp286	0.0907	0.599149386954389	-
6985818	Cotl1	0.0908	0.599149386954389	-
6788065	Zfp2	0.0909	0.599149386954389	-
6882666	Rpn2	0.0909	0.599149386954389	-
6915787	Efcab7	0.091	0.599149386954389	-
6846386	Cep97	0.0911	0.599149386954389	+
6934322	Tctn2	0.0911	0.599149386954389	-
6833414	Sp1	0.0912	0.599149386954389	-
6777902	Tspan31	0.0913	0.599149386954389	-
6816413	Nnt	0.0913	0.599149386954389	-
6966939	Flt3l	0.0913	0.599149386954389	-
6983822	Dcaf15	0.0913	0.599149386954389	-
6996438	Aph1c	0.0913	0.599149386954389	-
6998088	Zbtb38	0.0913	0.599149386954389	-
6947553	Smyd5	0.0914	0.599149386954389	-
6964030	Dctn5	0.0914	0.599149386954389	-
6817694	Il17rd	0.0915	0.599149386954389	-
6842933	Usp16	0.0915	0.599149386954389	-
6892895	Wfdc15b	0.0917	0.599149386954389	-
6848505	Synj2	0.0918	0.599149386954389	-
6916159	Echdc2	0.0918	0.599149386954389	+
6826546	Diap3	0.0919	0.599149386954389	-
6981188	Fnta	0.0919	0.599149386954389	-
6967837	Chrna7	0.092	0.599149386954389	-
6988230	Siae	0.092	0.599149386954389	-
6998192	Pik3cb	0.0921	0.599149386954389	-
6875459	Etl4	0.0922	0.599149386954389	-
6775480	Sirt6	0.0923	0.599149386954389	-
6773174	Dse	0.0924	0.599149386954389	-
6992428	Cspg5	0.0925	0.599149386954389	-
6979313	Nudt7	0.0926	0.599149386954389	-
6989368	Man2c1	0.0926	0.599149386954389	-
6918801	Lzic	0.0927	0.599149386954389	-
6921350	Tstd2	0.0927	0.599149386954389	-
6775182	Col6a2	0.0928	0.599149386954389	-
6824833	Mdp1	0.0928	0.599149386954389	-
6924873	Ipo13	0.0928	0.599149386954389	-
6794518	Ispd	0.0929	0.599149386954389	-
6759817	Obsl1	0.093	0.599149386954389	-
6763129	Lamc2	0.093	0.599149386954389	-
6772829	Akap7	0.093	0.599149386954389	-
6913315	Nr4a3	0.093	0.599149386954389	+
6856245	Vmac	0.0932	0.599149386954389	-
6959000	Rtn2	0.0932	0.599149386954389	-

6813246	Nfil3	0.0933	0.599149386954389	-
6892852	Ada	0.0933	0.599149386954389	-
6863857	Nol4	0.0935	0.599149386954389	-
6871164	Pola2	0.0935	0.599149386954389	-
6924834	Kif2c	0.0936	0.599149386954389	-
6963325	Tub	0.0936	0.599149386954389	-
6816130	Ddx4	0.0937	0.599149386954389	-
6899335	Dennd4b	0.0937	0.599149386954389	-
6838136	Twf1	0.0938	0.599149386954389	-
6847972	Runx1	0.0942	0.599149386954389	-
6933459	Chek2	0.0942	0.599149386954389	-
6902976	Pxmp3	0.0943	0.599149386954389	-
6935732	Rxfp2	0.0943	0.599149386954389	-
6979508	Wfdc1	0.0943	0.599149386954389	-
6966913	Med25	0.0944	0.599149386954389	-
6841701	Senp7	0.0946	0.599149386954389	-
6935630	Mtus2	0.0947	0.599149386954389	-
6952900	Hipk2	0.0948	0.599149386954389	-
6787967	Cnot6	0.0949	0.599149386954389	-
6929852	Grk4	0.0949	0.599149386954389	-
6907351	Pde4dip	0.0951	0.599149386954389	+
6926498	Efhd2	0.0951	0.599149386954389	+
6993096	Zfp105	0.0951	0.599149386954389	-
6876150	Exosc2	0.0952	0.599149386954389	-
6940950	Rnf212	0.0952	0.599149386954389	-
6965982	Axl	0.0952	0.599149386954389	-
6966354	Hpn	0.0953	0.599149386954389	-
6988213	Robo4	0.0953	0.599149386954389	-
6755091	Sh2d1b1	0.0954	0.599149386954389	-
6897166	Pcdh10	0.0954	0.599149386954389	-
6953145	Fam115a	0.0954	0.599149386954389	-
6782703	Sebox	0.0955	0.599149386954389	-
6824830	Tm9sf1	0.0956	0.599149386954389	-
6859273	Dsg1c	0.0956	0.599149386954389	-
6773080	Tpd52l1	0.0957	0.599149386954389	+
6836341	Sla	0.0957	0.599149386954389	-
6755451	Sdccag8	0.096	0.599149386954389	-
6836805	Plec	0.096	0.599149386954389	-
6890597	Fbn1	0.096	0.599149386954389	-
6972418	Osbpl5	0.096	0.599149386954389	-
6984413	Amfr	0.096	0.599149386954389	-
6985021	Esrp2	0.0962	0.599149386954389	-
6906083	Zbbx	0.0964	0.599149386954389	-
6827790	Tgds	0.0965	0.599149386954389	-
6884892	Nsun6	0.0965	0.599149386954389	-
6816951	Ptprg	0.0966	0.599149386954389	-
6863467	Osbpl1a	0.0966	0.599149386954389	-
6884101	Cdh4	0.0966	0.599149386954389	-
6937269	Poln	0.0967	0.599149386954389	-
6775250	Agpat3	0.0968	0.599149386954389	-

6785364	Ccdc40	0.0968	0.599149386954389	-
6775337	Sbno2	0.0969	0.599149386954389	-
6900142	Rsbn1	0.0969	0.599149386954389	-
6926377	Igsf21	0.0969	0.599149386954389	-
6945760	Sva	0.0969	0.599149386954389	-
6916148	0610037L13Rik	0.097	0.599149386954389	-
6940783	Barhl2	0.0971	0.599149386954389	-
6834025	Npr3	0.0972	0.599149386954389	+
6796744	Vash1	0.0974	0.599149386954389	-
7012561	Pcyt1b	0.0975	0.599149386954389	-
6935180	Get4	0.0976	0.599149386954389	-
6753930	Pdc	0.0977	0.599149386954389	-
6756115	Gpatch2	0.0977	0.599149386954389	-
6883533	Dok5	0.0977	0.599149386954389	+
6942634	Zfp68	0.0977	0.599149386954389	-
6800859	Egln3	0.0979	0.599149386954389	-
6899572	Rorc	0.0979	0.599149386954389	-
6973679	Arhgef18	0.098	0.599149386954389	-
6986150	Pcnxl2	0.098	0.599149386954389	-
6960382	Bcat2	0.0981	0.599149386954389	-
6972133	Olfr536	0.0981	0.599149386954389	-
6769088	Ilvbl	0.0982	0.599149386954389	-
6792494	Trim47	0.0983	0.599149386954389	-
6848146	Psmg1	0.0983	0.599149386954389	-
6751069	Col4a3	0.0984	0.599149386954389	-
6926605	Oog3	0.0985	0.599149386954389	-
6974008	Carkd	0.0986	0.599149386954389	-
6977260	Hmox1	0.0986	0.599149386954389	-
6794475	Agr3	0.0987	0.599149386954389	-
6887053	Wdsub1	0.0987	0.599149386954389	-
6908147	Fndc7	0.0987	0.599149386954389	-
6961203	H47	0.0987	0.599149386954389	-
6871117	Ctsw	0.0988	0.599149386954389	-
6960178	Klk8	0.0988	0.599149386954389	-
6984156	Tox3	0.0988	0.599149386954389	-
7012681	Msn	0.0988	0.599149386954389	-
6942550	Zan	0.0989	0.599149386954389	-
6761675	Epb4.1l5	0.0991	0.599149386954389	-
6787100	Rhbdf1	0.0992	0.599149386954389	+
6996038	Bbs4	0.0992	0.599149386954389	-
6799897	Pik3cg	0.0993	0.599149386954389	-
6975335	Dusp4	0.0993	0.599149386954389	+
6845042	Tmem44	0.0995	0.599149386954389	-
6861707	Tubb6	0.0996	0.599149386954389	-
6976609	Ddx60	0.0996	0.599149386954389	-
6881091	Tmem87b	0.0997	0.599149386954389	-
6950675	MGC7817	0.0997	0.599149386954389	-
6969429	4632434I11Rik	0.0999	0.599149386954389	-
6768621	Fam13c	0.1	0.599149386954389	-
6789229	Ndel1	0.1	0.599149386954389	+

6828741	C1qtnf3	0.1	0.599149386954389	-
6875666	Pnpla7	0.1	0.599149386954389	-
6878344	Osbpl6	0.1	0.599149386954389	-
6890802	Zc3h8	0.1	0.599149386954389	-
6892892	Wfdc12	0.1	0.599149386954389	-
6929510	Insig1	0.1	0.599149386954389	+
6934979	Alkbh4	0.1	0.599149386954389	-
6963271	Ppfibp2	0.1	0.599149386954389	-
6964553	Pstk	0.1	0.599149386954389	-
6972462	Cttn	0.1	0.599149386954389	-
6977014	Ell	0.1	0.599149386954389	-
6756238	Ptpn14	0.101	0.599149386954389	-
6772382	Ccdc28a	0.101	0.599149386954389	-
6775319	Med16	0.101	0.599149386954389	-
6831592	Zfp41	0.101	0.599149386954389	-
6851186	Emr4	0.101	0.599149386954389	-
6889510	2700007P21Rik	0.101	0.599149386954389	-
6890644	Usp50	0.101	0.599149386954389	-
6890713	Nphp1	0.101	0.599149386954389	-
6892376	Gss	0.101	0.599149386954389	-
6933599	Acacb	0.101	0.599149386954389	-
6943117	Mtif3	0.101	0.599149386954389	-
6955935	Eif4e3	0.101	0.599149386954389	-
6767384	Zbtb24	0.102	0.599149386954389	-
6785675	Nf2	0.102	0.599149386954389	-
6836797	Nrbp2	0.102	0.599149386954389	-
6843543	Bace2	0.102	0.599149386954389	-
6857512	Hnrpll	0.102	0.599149386954389	-
6869222	Pten	0.102	0.599149386954389	-
6876081	Prrx2	0.102	0.599149386954389	-
6885624	Cel	0.102	0.599149386954389	-
6924366	Scp2	0.102	0.599149386954389	-
6936406	Cd36	0.102	0.599149386954389	-
6945337	Agbl3	0.102	0.599149386954389	-
6947211	Lrrtm4	0.102	0.599149386954389	+
6947570	Tprkb	0.102	0.599149386954389	+
6960179	Klk7	0.102	0.599149386954389	-
6961108	Mtmr10	0.102	0.599149386954389	+
6765186	Rps6kc1	0.103	0.599149386954389	-
6771566	Arhgap9	0.103	0.599149386954389	-
6775201	Col18a1	0.103	0.599149386954389	-
6781927	Pik3r6	0.103	0.599149386954389	-
6791219	Cwc25	0.103	0.599149386954389	-
6811917	Prl7a2	0.103	0.599149386954389	-
6825119	Cdadc1	0.103	0.599149386954389	-
6841140	Cd200r4	0.103	0.599149386954389	-
6844553	Tra2b	0.103	0.599149386954389	-
6848127	Erg	0.103	0.599149386954389	-
6855317	Crisp1	0.103	0.599149386954389	-
6864776	Pcdh1	0.103	0.599149386954389	-

6913262	Galnt12	0.103	0.599149386954389	-
6927253	Gabrd	0.103	0.599149386954389	-
6940368	Fam175a	0.103	0.599149386954389	-
6962876	Gdpd5	0.103	0.599149386954389	+
6966788	Zfp715	0.103	0.599149386954389	-
6983890	Mast1	0.103	0.599149386954389	-
6984375	Ces1g	0.103	0.599149386954389	-
6987632	B3gat1	0.103	0.599149386954389	-
6749142	Col3a1	0.104	0.599149386954389	-
6755207	Slamf1	0.104	0.599149386954389	-
6849990	Tapbp	0.104	0.599149386954389	-
6856796	Arhgap28	0.104	0.599149386954389	-
6871062	Npas4	0.104	0.599149386954389	+
6882538	Acss2	0.104	0.599149386954389	+
6896353	Tnfsf10	0.104	0.599149386954389	-
6896850	Fgf2	0.104	0.599149386954389	+
6913371	E130309F12Rik	0.104	0.599149386954389	+
6933259	Rpap2	0.104	0.599149386954389	-
6934274	Arl6ip4	0.104	0.599149386954389	-
6977676	Dnajb1	0.104	0.599149386954389	+
6983793	Pkn1	0.104	0.599149386954389	-
6986725	Mmp3	0.104	0.599149386954389	-
6748883	Il1r2	0.105	0.599149386954389	-
6765321	Traf3ip3	0.105	0.599149386954389	-
6785609	Smtn	0.105	0.599149386954389	-
6789719	Hic1	0.105	0.599149386954389	-
6796485	Pcnx	0.105	0.599149386954389	+
6801459	Sdccag1	0.105	0.599149386954389	-
6873160	Mms19	0.105	0.599149386954389	-
6887282	Grb14	0.105	0.599149386954389	+
6905596	Vmn2r3	0.105	0.599149386954389	-
6920630	Ankrd6	0.105	0.599149386954389	-
6921130	Stoml2	0.105	0.599149386954389	-
6922026	Ptgr1	0.105	0.599149386954389	-
6933072	Spp1	0.105	0.599149386954389	-
6937711	Rab28	0.105	0.599149386954389	-
6983172	Armc6	0.105	0.599149386954389	-
6987049	Fam76b	0.105	0.599149386954389	-
7014178	Col4a5	0.105	0.599149386954389	-
6748011	Tmem14a	0.106	0.599149386954389	+
6769150	Ptbp1	0.106	0.599149386954389	+
6776761	Acss3	0.106	0.599149386954389	-
6778425	Ap1b1	0.106	0.599149386954389	-
6784291	Dhx8	0.106	0.599149386954389	-
6787174	Fgf18	0.106	0.599149386954389	-
6789743	Serpinf2	0.106	0.599149386954389	-
6791570	Itga2b	0.106	0.599149386954389	-
6809372	Ankra2	0.106	0.599149386954389	-
6815529	Naip6	0.106	0.599149386954389	-
6868524	Trpm6	0.106	0.599149386954389	-

6911682	Ccne2	0.106	0.599149386954389	-
6916775	Hivep3	0.106	0.599149386954389	+
6931856	Rest	0.106	0.599149386954389	-
6935133	Zfp157	0.106	0.599149386954389	-
6939723	Sult1d1	0.106	0.599149386954389	-
6949591	Slc6a13	0.106	0.599149386954389	-
6952659	2010107G12Rik	0.106	0.599149386954389	-
6955164	Cml5	0.106	0.599149386954389	-
6980944	Ckap2	0.106	0.599149386954389	-
6985655	2310061C15Rik	0.106	0.599149386954389	-
6998163	4930579K19Rik	0.106	0.599149386954389	-
6778358	Sec14l4	0.107	0.599149386954389	-
6781297	2810021J22Rik	0.107	0.599149386954389	-
6791332	Krt12	0.107	0.599149386954389	-
6792919	1700012B15Rik	0.107	0.599149386954389	-
6829971	Tm7sf4	0.107	0.599149386954389	-
6839420	Snx29	0.107	0.599149386954389	-
6840717	Sema5b	0.107	0.599149386954389	-
6850412	Rhag	0.107	0.599149386954389	-
6853249	Nox3	0.107	0.599149386954389	-
6899682	Ctsk	0.107	0.599149386954389	-
6900975	Arhgap29	0.107	0.599149386954389	-
6916365	Skint4	0.107	0.599149386954389	-
6932601	Fras1	0.107	0.599149386954389	-
6972200	Deaf1	0.107	0.599149386954389	-
6978314	Herpud1	0.107	0.599149386954389	-
6983639	Hhip	0.107	0.599149386954389	-
6983839	Mri1	0.107	0.599149386954389	-
6999154	Cmtm8	0.107	0.599149386954389	-
7014048	Zcchc18	0.107	0.599149386954389	-
6750868	Acsl3	0.108	0.599149386954389	-
6782430	Smyd4	0.108	0.599149386954389	-
6796902	Tshr	0.108	0.599149386954389	-
6808781	Hapln1	0.108	0.599149386954389	-
6825684	Sorbs3	0.108	0.599149386954389	-
6838717	Rarg	0.108	0.599149386954389	-
6849600	Rab44	0.108	0.599149386954389	-
6876421	Morn5	0.108	0.599149386954389	-
6883094	Rbpjl	0.108	0.599149386954389	-
6899875	Zfp697	0.108	0.599149386954389	+
6907226	Plekho1	0.108	0.599149386954389	-
6910707	Msh4	0.108	0.599149386954389	-
6926387	Arhgef10l	0.108	0.599149386954389	-
6928719	Sri	0.108	0.599149386954389	-
6939348	Spink2	0.108	0.599149386954389	-
6941150	2900026A02Rik	0.108	0.599149386954389	-
6964177	D430042O09Rik	0.108	0.599149386954389	-
6971403	Stx1b	0.108	0.599149386954389	-
6978369	Mmp15	0.108	0.599149386954389	-
6985426	Adat1	0.108	0.599149386954389	-

6993851	Ccdc151	0.108	0.599149386954389	-
6771366	Fam19a2	0.109	0.599149386954389	-
6783182	Bcas3	0.109	0.599149386954389	+
6789894	Cryba1	0.109	0.599149386954389	-
6798946	Gen1	0.109	0.599149386954389	-
6806956	Ecm2	0.109	0.599149386954389	-
6817642	Cphx	0.109	0.599149386954389	-
6835222	Lrp12	0.109	0.599149386954389	-
6849221	Pkmyt1	0.109	0.599149386954389	-
6873887	Gpam	0.109	0.599149386954389	-
6907211	Rprd2	0.109	0.599149386954389	-
6921918	D730040F13Rik	0.109	0.599149386954389	-
6965755	Qpctl	0.109	0.599149386954389	-
6991112	Prss35	0.109	0.599149386954389	-
6753592	Aspm	0.11	0.599149386954389	-
6775373	Tcf3	0.11	0.599149386954389	-
6791656	Dcakd	0.11	0.599149386954389	-
6808279	Pcsk1	0.11	0.599149386954389	+
6824871	Gzmd	0.11	0.599149386954389	-
6850646	Slc35b2	0.11	0.599149386954389	-
6870063	Nfkb2	0.11	0.599149386954389	-
6876185	Ciz1	0.11	0.599149386954389	-
6912504	Lyrm2	0.11	0.599149386954389	-
6932118	Stap1	0.11	0.599149386954389	-
6769747	Ikbip	0.111	0.599149386954389	-
6791800	Cdc27	0.111	0.599149386954389	-
6793050	2810032G03Rik	0.111	0.599149386954389	-
6811512	Zfp192	0.111	0.599149386954389	-
6901747	Adh5	0.111	0.599149386954389	-
6906433	D930015E06Rik	0.111	0.599149386954389	-
6910231	Wdr63	0.111	0.599149386954389	-
6913001	Atp8b5	0.111	0.599149386954389	-
6921397	Anks6	0.111	0.599149386954389	-
6925642	Tinagl1	0.111	0.599149386954389	-
6926950	Nmnat1	0.111	0.599149386954389	-
6928880	Sema3e	0.111	0.599149386954389	+
6933422	Pole	0.111	0.599149386954389	-
6933990	Rasal1	0.111	0.599149386954389	-
6950985	Lrmp	0.111	0.599149386954389	-
6963793	Xylt1	0.111	0.599149386954389	-
6970911	Eri2	0.111	0.599149386954389	+
7014660	4930542N07Rik	0.111	0.599149386954389	-
7017609	Irak1	0.111	0.599149386954389	-
6785608	Inpp5j	0.112	0.599149386954389	-
6797453	Catsperb	0.112	0.599149386954389	-
6815522	Naip2	0.112	0.599149386954389	-
6819722	Fam167a	0.112	0.599149386954389	-
6839681	Pkp2	0.112	0.599149386954389	-
6848102	Kcnj6	0.112	0.599149386954389	-
6876226	Fam129b	0.112	0.599149386954389	+

6901600	Nhedc1	0.112	0.599149386954389	-
6942587	Cyp3a13	0.112	0.599149386954389	-
6960846	Herc2	0.112	0.599149386954389	-
7011840	Mtm1	0.112	0.599149386954389	-
6777190	Lgr5	0.113	0.599149386954389	-
6795599	Fancm	0.113	0.599149386954389	-
6812446	Cage1	0.113	0.599149386954389	-
6813982	Zfp708	0.113	0.599149386954389	-
6824451	Otx2	0.113	0.599149386954389	-
6850763	Trerf1	0.113	0.599149386954389	-
6881438	Plcb1	0.113	0.599149386954389	-
6908079	Gstm2	0.113	0.599149386954389	-
6946021	Gimap8	0.113	0.599149386954389	-
6962502	Dlg2	0.113	0.599149386954389	-
6969690	Uvrag	0.113	0.599149386954389	-
6995453	Ttc12	0.113	0.599149386954389	-
6999438	Scn5a	0.113	0.599149386954389	-
6758970	Als2cr12	0.114	0.599149386954389	-
6766287	Nhsl1	0.114	0.599149386954389	-
6785122	Otop2	0.114	0.599149386954389	-
6793186	Hs1bp3	0.114	0.599149386954389	-
6808810	Tmem167	0.114	0.599149386954389	-
6817403	Myst4	0.114	0.599149386954389	-
6822749	Gm5087	0.114	0.599149386954389	-
6823068	Anxa7	0.114	0.599149386954389	-
6828582	2410089E03Rik	0.114	0.599149386954389	-
6831919	Kdelr3	0.114	0.599149386954389	-
6845909	Qtrtd1	0.114	0.599149386954389	-
6849986	Daxx	0.114	0.599149386954389	-
6868650	Tmem2	0.114	0.599149386954389	+
6872765	Acta2	0.114	0.599149386954389	-
6873368	Kcnip2	0.114	0.599149386954389	+
6885879	Slc25a25	0.114	0.599149386954389	+
6894182	BC066135	0.114	0.599149386954389	-
6917208	Sfpq	0.114	0.599149386954389	-
6917595	Gpn2	0.114	0.599149386954389	-
6924588	Skint10	0.114	0.599149386954389	-
6926416	Necap2	0.114	0.599149386954389	-
6941687	Acad10	0.114	0.599149386954389	-
6959292	Numbl	0.114	0.599149386954389	+
6968781	Furin	0.114	0.599149386954389	+
6977142	Rab8a	0.114	0.599149386954389	-
6980943	Nek3	0.114	0.599149386954389	-
6750302	Spag16	0.115	0.599149386954389	-
6768867	Ggt1	0.115	0.599149386954389	-
6815739	Rgs7bp	0.115	0.599149386954389	-
6828001	A2ld1	0.115	0.599149386954389	-
6831959	Apobec3	0.115	0.599149386954389	-
6832530	Creld2	0.115	0.599149386954389	-
6836794	Scrib	0.115	0.599149386954389	-

6839836	Arvcf	0.115	0.599149386954389	-
6844348	Sept5	0.115	0.599149386954389	-
6937191	C330019G07Rik	0.115	0.599149386954389	-
6937290	Zfyve28	0.115	0.599149386954389	-
6939979	Ppef2	0.115	0.599149386954389	-
6943775	Ccdc132	0.115	0.599149386954389	-
6946047	1600015I10Rik	0.115	0.599149386954389	-
6963011	Clpb	0.115	0.599149386954389	-
6992849	Eomes	0.115	0.599149386954389	-
6748191	Col9a1	0.116	0.599149386954389	-
6770465	Ppfia2	0.116	0.599149386954389	-
6782002	Kcnab3	0.116	0.599149386954389	-
6790046	Evi2b	0.116	0.599149386954389	-
6819096	A630038E17Rik	0.116	0.599149386954389	-
6847659	Grik1	0.116	0.599149386954389	-
6849432	Tmem8	0.116	0.599149386954389	-
6861441	Spink10	0.116	0.599149386954389	-
6886244	Lrp1b	0.116	0.599149386954389	-
6926078	A330049M08Rik	0.116	0.599149386954389	-
6934177	Rnf34	0.116	0.599149386954389	-
6949235	Ift122	0.116	0.599149386954389	-
6966194	Catsperg2	0.116	0.599149386954389	-
6969007	Il16	0.116	0.599149386954389	+
6969639	Myo7a	0.116	0.599149386954389	-
6971352	9130019O22Rik	0.116	0.599149386954389	-
6984926	Cdh16	0.116	0.599149386954389	-
7018979	Pof1b	0.116	0.599149386954389	-
6788851	Epn2	0.117	0.599149386954389	-
6791519	Etv4	0.117	0.599149386954389	-
6800986	Slc25a21	0.117	0.599149386954389	-
6808308	Ell2	0.117	0.599149386954389	+
6836325	Tmem71	0.117	0.599149386954389	-
6850047	BC051142	0.117	0.599149386954389	-
6864404	Gypc	0.117	0.599149386954389	-
6870630	Tdrd1	0.117	0.599149386954389	-
6873237	Dnmbp	0.117	0.599149386954389	-
6904074	Mrpl47	0.117	0.599149386954389	-
6905366	Siah2	0.117	0.599149386954389	+
6952137	Cadps2	0.117	0.599149386954389	-
6954640	Tcf7l1	0.117	0.599149386954389	-
6955259	Antxr1	0.117	0.599149386954389	-
6966158	Pak4	0.117	0.599149386954389	-
6968790	Blm	0.117	0.599149386954389	-
6994579	St3gal4	0.117	0.599149386954389	-
6747497	Sgk3	0.118	0.599149386954389	-
6769614	Nup37	0.118	0.599149386954389	-
6773504	Smpd2	0.118	0.599149386954389	+
6819744	Sox7	0.118	0.599149386954389	-
6839080	Nudt16l1	0.118	0.599149386954389	-
6854619	Slc26a8	0.118	0.599149386954389	-

6882189	Gins1	0.118	0.599149386954389	-
6898154	Rsrc1	0.118	0.599149386954389	-
6908108	Sypl2	0.118	0.599149386954389	-
6924882	Ptprf	0.118	0.599149386954389	+
6929842	Rnf4	0.118	0.599149386954389	-
6942837	Radil	0.118	0.599149386954389	-
6989217	Dnaja4	0.118	0.599149386954389	-
7020764	Ofd1	0.118	0.599149386954389	-
6762259	Mdm4	0.119	0.599149386954389	-
6775984	Tmpo	0.119	0.599149386954389	-
6781458	Smcr7	0.119	0.599149386954389	-
6782125	Arrb2	0.119	0.599149386954389	-
6815539	Ocln	0.119	0.599149386954389	+
6823887	Sncg	0.119	0.599149386954389	-
6833394	Soat2	0.119	0.599149386954389	-
6924291	Lrrc42	0.119	0.599149386954389	-
6929695	Krtcap3	0.119	0.599149386954389	-
6945032	Smo	0.119	0.599149386954389	-
6959617	Dmkn	0.119	0.599149386954389	-
6988774	Bud13	0.119	0.599149386954389	-
6992370	Ip6k2	0.119	0.599149386954389	-
6994038	Tbx20	0.119	0.599149386954389	-
6995661	AI593442	0.119	0.599149386954389	-
6999442	Scn10a	0.119	0.599149386954389	-
6766063	Adat2	0.12	0.599149386954389	-
6775316	BC005764	0.12	0.599149386954389	-
6809876	4933425L06Rik	0.12	0.599149386954389	-
6817645	Gm10394	0.12	0.599149386954389	-
6820563	Mtrf1	0.12	0.599149386954389	-
6832132	Tef	0.12	0.599149386954389	-
6868066	Prpf19	0.12	0.599149386954389	-
6900143	Phtf1	0.12	0.599149386954389	-
6905570	Plch1	0.12	0.599149386954389	-
6916125	Yipf1	0.12	0.599149386954389	-
6916127	Tmem48	0.12	0.599149386954389	-
6940611	Sparcl1	0.12	0.599149386954389	-
6983145	Gatad2a	0.12	0.599149386954389	-
6989222	Crabp1	0.12	0.599149386954389	-
6989442	Stra6	0.12	0.599149386954389	-
6749813	Cyp20a1	0.121	0.599149386954389	-
6775151	Gstt1	0.121	0.599149386954389	-
6777306	9530003J23Rik	0.121	0.599149386954389	-
6783997	Ppp1r1b	0.121	0.599149386954389	-
6785446	Slc25a10	0.121	0.599149386954389	-
6795041	Ap4s1	0.121	0.599149386954389	-
6802449	Tgfb3	0.121	0.599149386954389	-
6841363	Retnlb	0.121	0.599149386954389	-
6854341	Dnase1l2	0.121	0.599149386954389	-
6898897	Pet112l	0.121	0.599149386954389	-
6930023	Evc2	0.121	0.599149386954389	-

6953523	4921507P07Rik	0.121	0.599149386954389	-
6975059	BC019943	0.121	0.599149386954389	-
6758993	Mpp4	0.122	0.599149386954389	-
6769255	Gadd45b	0.122	0.599149386954389	+
6783737	Fam117a	0.122	0.599149386954389	-
6784005	Erbb2	0.122	0.599149386954389	-
6788580	Zfp672	0.122	0.599149386954389	-
6805259	Zfp184	0.122	0.599149386954389	-
6855079	Apom	0.122	0.599149386954389	-
6868017	Rab3il1	0.122	0.599149386954389	-
6872584	Gldc	0.122	0.599149386954389	+
6885349	Ehmt1	0.122	0.599149386954389	-
6906749	Sema4a	0.122	0.599149386954389	-
6916369	Skint3	0.122	0.599149386954389	-
6932409	Parm1	0.122	0.599149386954389	-
6984466	Fam192a	0.122	0.599149386954389	-
6985328	Il34	0.122	0.599149386954389	-
6985332	Fuk	0.122	0.599149386954389	-
7010093	Gpr82	0.122	0.599149386954389	-
6761272	Vps4b	0.123	0.599149386954389	-
6769164	Kiss1r	0.123	0.599149386954389	-
6781580	Trpv2	0.123	0.599149386954389	-
6837453	Ttll12	0.123	0.599149386954389	-
6837784	Mapk12	0.123	0.599149386954389	-
6844072	Parn	0.123	0.599149386954389	-
6858868	Rbbp8	0.123	0.599149386954389	-
6873194	Loxl4	0.123	0.599149386954389	-
6884456	Camk1d	0.123	0.599149386954389	-
6934292	2810006K23Rik	0.123	0.599149386954389	-
6942866	Rnf216	0.123	0.599149386954389	-
6965177	Lrrc56	0.123	0.599149386954389	-
6972178	Rnh1	0.123	0.599149386954389	-
6994790	AW551984	0.123	0.599149386954389	-
6997197	Col12a1	0.123	0.599149386954389	-
6749376	Stat1	0.124	0.599149386954389	-
6839871	Hira	0.124	0.599149386954389	-
6843550	Mx2	0.124	0.599149386954389	-
6871542	Ms4a1	0.124	0.599149386954389	-
6878991	Agbl2	0.124	0.599149386954389	-
6879966	Muc15	0.124	0.599149386954389	-
6891772	Crnkl1	0.124	0.599149386954389	-
6912215	Gpr63	0.124	0.599149386954389	-
6917120	Csf3r	0.124	0.599149386954389	-
6950637	Pik3c2g	0.124	0.599149386954389	-
6952231	Pot1a	0.124	0.599149386954389	-
6952872	Zc3hav1l	0.124	0.599149386954389	-
6971392	Zfp629	0.124	0.599149386954389	-
6985392	Ctrb1	0.124	0.599149386954389	-
6998649	Rbm6	0.124	0.599149386954389	-
6998763	Fbxw26	0.124	0.599149386954389	-

7018993	Chm	0.124	0.599149386954389	-
6762141	Dyrk3	0.125	0.599149386954389	-
6802262	Dpf3	0.125	0.599149386954389	-
6811714	Slc17a4	0.125	0.599149386954389	-
6818053	Oxnad1	0.125	0.599149386954389	-
6830510	Mtbp	0.125	0.599149386954389	-
6831600	Rhpn1	0.125	0.599149386954389	-
6841712	Abi3bp	0.125	0.599149386954389	-
6843178	Il10rb	0.125	0.599149386954389	-
6851103	Kat2b	0.125	0.599149386954389	-
6861776	Mc5r	0.125	0.599149386954389	-
6867885	Macrod1	0.125	0.599149386954389	-
6895420	Hnf4g	0.125	0.599149386954389	-
6915619	Nfia	0.125	0.599149386954389	-
6922808	Mpdz	0.125	0.599149386954389	-
6923497	Cyp2j11-ps	0.125	0.599149386954389	-
6925933	Pigv	0.125	0.599149386954389	-
6946103	Stk31	0.125	0.599149386954389	-
6958031	Slco1a4	0.125	0.599149386954389	+
6959448	Sars2	0.125	0.599149386954389	-
6967016	Syngr4	0.125	0.599149386954389	-
6981107	Adam32	0.125	0.599149386954389	-
6990435	Rab27a	0.125	0.599149386954389	+
6748662	Zap70	0.126	0.599149386954389	-
6749346	Tmeff2	0.126	0.599149386954389	-
6766491	Slc2a12	0.126	0.599149386954389	-
6835063	Rrm2b	0.126	0.599149386954389	-
6843177	Ifnar2	0.126	0.599149386954389	-
6873361	Fbxw4	0.126	0.599149386954389	-
6892747	Ptprt	0.126	0.599149386954389	-
6916712	Elovl1	0.126	0.599149386954389	-
6919015	Icmt	0.126	0.599149386954389	-
6926123	Lypla2	0.126	0.599149386954389	-
6934262	Hip1r	0.126	0.599149386954389	-
6941032	Fbrsl1	0.126	0.599149386954389	-
6957348	Pzp	0.126	0.599149386954389	-
6959138	Lypd3	0.126	0.599149386954389	-
6992414	Cdc25a	0.126	0.599149386954389	-
6771526	Ctdsp2	0.127	0.599149386954389	-
6772594	Myb	0.127	0.599149386954389	-
6796567	Acot3	0.127	0.599149386954389	-
6797720	Tcl1b3	0.127	0.599149386954389	-
6836838	Cpsf1	0.127	0.599149386954389	-
6849539	Fance	0.127	0.599149386954389	-
6866693	Lipg	0.127	0.599149386954389	-
6868404	Rfk	0.127	0.599149386954389	-
6913876	Ugcg	0.127	0.599149386954389	-
6918015	Pla2g2d	0.127	0.599149386954389	-
6960622	Prmt3	0.127	0.599149386954389	-
6965219	Muc2	0.127	0.599149386954389	-

6968799	Iqgap1	0.127	0.599149386954389	-
6769201	Mum1	0.128	0.599149386954389	-
6783259	Tubd1	0.128	0.599149386954389	-
6783439	Trim25	0.128	0.599149386954389	-
6786660	1700093K21Rik	0.128	0.599149386954389	-
6827801	Sox21	0.128	0.599149386954389	-
6839135	Rbfox1	0.128	0.599149386954389	-
6840101	Kng1	0.128	0.599149386954389	-
6870956	Tcirg1	0.128	0.599149386954389	-
6872530	5033414D02Rik	0.128	0.599149386954389	-
6880627	Ccndbp1	0.128	0.599149386954389	-
6887089	Rbms1	0.128	0.599149386954389	-
6917963	Rap1gap	0.128	0.599149386954389	-
6943873	Asb4	0.128	0.599149386954389	-
6949583	Pex26	0.128	0.599149386954389	-
6949617	Mug1	0.128	0.599149386954389	-
6949653	Mug2	0.128	0.599149386954389	-
6959306	Prx	0.128	0.599149386954389	-
6985263	2400003C14Rik	0.128	0.599149386954389	-
6995968	Loxl1	0.128	0.599149386954389	-
7016760	Igsf1	0.128	0.599149386954389	-
6782685	2610507B11Rik	0.129	0.599149386954389	-
6814376	Cep72	0.129	0.599149386954389	-
6822729	Fezf2	0.129	0.599149386954389	+
6824229	Ddhd1	0.129	0.599149386954389	-
6830131	Ttc35	0.129	0.599149386954389	-
6838654	Gm5478	0.129	0.599149386954389	-
6838809	Itga5	0.129	0.599149386954389	-
6880487	Rpusd2	0.129	0.599149386954389	-
6887191	Fap	0.129	0.599149386954389	-
6896038	Hltf	0.129	0.599149386954389	-
6905374	Clrn1	0.129	0.599149386954389	-
6921074	Sigmar1	0.129	0.599149386954389	-
6925054	Ctps	0.129	0.599149386954389	-
6936929	Lmbr1	0.129	0.599149386954389	-
6958951	Igfl3	0.129	0.599149386954389	-
6962745	Gab2	0.129	0.599149386954389	-
6972329	Ascl2	0.129	0.599149386954389	-
6983879	Calr	0.129	0.599149386954389	-
6760711	Hdac4	0.13	0.599149386954389	-
6775472	Gna15	0.13	0.599149386954389	-
6785481	Lrrc45	0.13	0.599149386954389	-
6798234	Zfyve21	0.13	0.599149386954389	-
6810625	4833420G17Rik	0.13	0.599149386954389	-
6814656	Gpr98	0.13	0.599149386954389	-
6837390	Cyp2d34	0.13	0.599149386954389	-
6848147	Brwd1	0.13	0.599149386954389	-
6849893	Cyp4f15	0.13	0.599149386954389	-
6852655	Pkdcc	0.13	0.599149386954389	-
6885484	Snapc4	0.13	0.599149386954389	-

6885488	Notch1	0.13	0.599149386954389	-
6896498	Mynn	0.13	0.599149386954389	-
6896655	Ttc14	0.13	0.599149386954389	-
6915096	Adamtsl1	0.13	0.599149386954389	-
6915801	Ror1	0.13	0.599149386954389	-
6930843	Anapc4	0.13	0.599149386954389	-
6934258	Ccdc62	0.13	0.599149386954389	-
6951352	Sgce	0.13	0.599149386954389	-
6955025	Mthfd2	0.13	0.599149386954389	+
6978296	Nup93	0.13	0.599149386954389	-
6987413	Ccdc159	0.13	0.599149386954389	-
6991791	Dbr1	0.13	0.599149386954389	-
6996379	Snx1	0.13	0.599149386954389	+
6773655	Prdm1	0.131	0.599149386954389	-
6791221	1700001P01Rik	0.131	0.599149386954389	-
6792814	Cbr2	0.131	0.599149386954389	-
6855166	H2-T3	0.131	0.599149386954389	-
6856480	Efna5	0.131	0.599149386954389	-
6879646	Cd59a	0.131	0.599149386954389	-
6885951	Fam125b	0.131	0.599149386954389	-
6899835	Fmo5	0.131	0.599149386954389	-
6905289	Tm4sf1	0.131	0.599149386954389	-
6913080	Melk	0.131	0.599149386954389	-
6926987	Spsb1	0.131	0.599149386954389	+
6941249	Acads	0.131	0.599149386954389	-
6950148	Clec9a	0.131	0.599149386954389	-
6956764	Zfp422	0.131	0.599149386954389	-
6983888	Gcdh	0.131	0.599149386954389	-
6993125	Lars2	0.131	0.599149386954389	-
7014115	Cldn2	0.131	0.599149386954389	-
6755241	Igsf9	0.132	0.599149386954389	-
6769366	Chst11	0.132	0.599149386954389	+
6790245	Slfn10-ps	0.132	0.599149386954389	-
6793674	Nol10	0.132	0.599149386954389	-
6833842	Nipbl	0.132	0.599149386954389	-
6846670	Epha3	0.132	0.599149386954389	-
6855737	Ubr2	0.132	0.599149386954389	-
6871297	Naa40	0.132	0.599149386954389	-
6891336	Jag1	0.132	0.599149386954389	-
6900456	Vav3	0.132	0.599149386954389	-
6908031	Slc6a17	0.132	0.599149386954389	-
6954810	Reg3g	0.132	0.599149386954389	-
6957677	Gprc5d	0.132	0.599149386954389	-
6964364	Hsd3b7	0.132	0.599149386954389	-
6983531	Ednra	0.132	0.599149386954389	+
6984093	Sall1	0.132	0.599149386954389	-
7009791	Kcnd1	0.132	0.599149386954389	-
7014855	Phka2	0.132	0.599149386954389	-
6773432	Slc16a10	0.133	0.599149386954389	-
6773546	Lace1	0.133	0.599149386954389	-

6774020	Man1a	0.133	0.599149386954389	-
6774719	Arid5b	0.133	0.599149386954389	+
6785551	Tbcd	0.133	0.599149386954389	-
6788866	Akap10	0.133	0.599149386954389	-
6790734	Stxbp4	0.133	0.599149386954389	+
6798392	Vipr2	0.133	0.599149386954389	-
6817136	Ngly1	0.133	0.599149386954389	-
6822485	4930452B06Rik	0.133	0.599149386954389	-
6849761	Dnahc8	0.133	0.599149386954389	-
6850421	Mut	0.133	0.599149386954389	-
6873993	A630007B06Rik	0.133	0.599149386954389	-
6899218	Muc1	0.133	0.599149386954389	-
6900071	Ngf	0.133	0.599149386954389	-
6923584	E130114P18Rik	0.133	0.599149386954389	-
6935082	Lrch4	0.133	0.599149386954389	-
6937334	Nop14	0.133	0.599149386954389	-
6938958	Yipf7	0.133	0.599149386954389	-
6958048	Slco1a5	0.133	0.599149386954389	-
6961232	Lins	0.133	0.599149386954389	-
6972106	Adam8	0.133	0.599149386954389	-
6973761	BC003267	0.133	0.599149386954389	-
6983222	Mpv17l2	0.133	0.599149386954389	-
7010645	Il13ra1	0.133	0.599149386954389	-
6750628	Speg	0.134	0.599149386954389	-
6751219	Sp100	0.134	0.599149386954389	-
6751345	Atg16l1	0.134	0.599149386954389	-
6788069	Zfp354b	0.134	0.599149386954389	-
6796430	Smoc1	0.134	0.599149386954389	-
6816124	Il31ra	0.134	0.599149386954389	-
6819591	Sacs	0.134	0.599149386954389	+
6837766	Alg12	0.134	0.599149386954389	-
6844301	Car15	0.134	0.599149386954389	-
6855669	Rsph9	0.134	0.599149386954389	-
6916095	Ssbp3	0.134	0.599149386954389	+
6921382	Tbc1d2	0.134	0.599149386954389	-
6942716	Snx8	0.134	0.599149386954389	-
6948015	Slc6a6	0.134	0.599149386954389	-
6973572	Isoc2b	0.134	0.599149386954389	-
6981113	Plekha2	0.134	0.599149386954389	+
6995123	Tmem25	0.134	0.599149386954389	-
6998668	Apeh	0.134	0.599149386954389	-
7013952	Gprasp1	0.134	0.599149386954389	-
6775288	2610008E11Rik	0.135	0.599149386954389	+
6811716	Scgn	0.135	0.599149386954389	-
6819247	Pck2	0.135	0.599149386954389	-
6825207	Ebpl	0.135	0.599149386954389	-
6825888	Itm2b	0.135	0.599149386954389	-
6849295	Mlst8	0.135	0.599149386954389	-
6854231	A630033E08Rik	0.135	0.599149386954389	-
6867626	Nudt8	0.135	0.599149386954389	-

6884477	Echdc3	0.135	0.599149386954389	-
6898771	Fgg	0.135	0.599149386954389	-
6910668	St6galnac3	0.135	0.599149386954389	-
6938963	Gnpda2	0.135	0.599149386954389	-
6945699	Ptpdc1	0.135	0.599149386954389	-
6957356	Klrb1a	0.135	0.599149386954389	-
6977061	Ocel1	0.135	0.599149386954389	-
6992431	Scap	0.135	0.599149386954389	-
6998301	Pccb	0.135	0.599149386954389	-
7018129	Pet2	0.135	0.599149386954389	-
6747577	A830018L16Rik	0.136	0.599149386954389	-
6764578	Adck3	0.136	0.599149386954389	-
6773067	Trmt11	0.136	0.599149386954389	-
6774947	Cisd1	0.136	0.599149386954389	+
6790972	Xylt2	0.136	0.599149386954389	-
6811724	Lrrc16a	0.136	0.599149386954389	+
6831637	Grina	0.136	0.599149386954389	-
6837081	Ankrd54	0.136	0.599149386954389	-
6845995	Tmprss7	0.136	0.599149386954389	-
6899171	5830417I10Rik	0.136	0.599149386954389	-
6928734	Slc25a40	0.136	0.599149386954389	-
6943031	Cyp3a16	0.136	0.599149386954389	-
6960848	Oca2	0.136	0.599149386954389	-
6976954	Atp13a1	0.136	0.599149386954389	-
6987862	Adamts8	0.136	0.599149386954389	-
6747986	Paqr8	0.137	0.599149386954389	-
6762195	Mfsd4	0.137	0.599149386954389	-
6768092	Lims1	0.137	0.599149386954389	-
6782414	Smg6	0.137	0.599149386954389	-
6805794	Mboat1	0.137	0.599149386954389	-
6831613	Mapk15	0.137	0.599149386954389	-
6838683	Krt77	0.137	0.599149386954389	-
6838692	Krt4	0.137	0.599149386954389	-
6852361	Vit	0.137	0.599149386954389	-
6855040	Egfl8	0.137	0.599149386954389	-
6865949	Tcof1	0.137	0.599149386954389	-
6891115	Lrrn4	0.137	0.599149386954389	-
6894180	Cables2	0.137	0.599149386954389	-
6897502	2810046L04Rik	0.137	0.599149386954389	-
6907638	Atp1a1	0.137	0.599149386954389	-
6907786	Hipk1	0.137	0.599149386954389	-
6910938	Cth	0.137	0.599149386954389	-
6914764	Tyrp1	0.137	0.599149386954389	-
6915929	Dab1	0.137	0.599149386954389	-
6918691	Nppb	0.137	0.599149386954389	-
6920990	B4galt1	0.137	0.599149386954389	-
6935312	Sdk1	0.137	0.599149386954389	-
6970134	Apbb1	0.137	0.599149386954389	-
6985886	Klhdc4	0.137	0.599149386954389	-
6990705	Mto1	0.137	0.599149386954389	-

6992475	Als2cl	0.137	0.599149386954389	-
7010086	Nyx	0.137	0.599149386954389	-
6751562	Asb1	0.138	0.599149386954389	-
6805468	Dcdc2a	0.138	0.599149386954389	-
6848813	Gm3435	0.138	0.599149386954389	-
6852471	Galm	0.138	0.599149386954389	-
6864370	Myo7b	0.138	0.599149386954389	-
6934226	Mlxip	0.138	0.599149386954389	-
6935039	Fis1	0.138	0.599149386954389	-
6942180	Phkg1	0.138	0.599149386954389	-
6946971	Tmem150a	0.138	0.599149386954389	-
6958905	Slc1a5	0.138	0.599149386954389	-
6960836	Cyfip1	0.138	0.599149386954389	-
6965192	Eps8l2	0.138	0.599149386954389	-
6974003	Col4a2	0.138	0.599149386954389	-
7014881	Scml2	0.138	0.599149386954389	-
6748828	Nms	0.139	0.599149386954389	-
6774226	Sep10	0.139	0.599149386954389	-
6784023	Zpbp2	0.139	0.599149386954389	-
6784578	Tanc2	0.139	0.599149386954389	-
6787896	Irgm1	0.139	0.599149386954389	-
6794059	Rnaseh1	0.139	0.599149386954389	-
6797551	Ppp4r4	0.139	0.599149386954389	+
6802333	Abcd4	0.139	0.599149386954389	-
6811368	A530099J19Rik	0.139	0.599149386954389	-
6835089	Klf10	0.139	0.599149386954389	+
6862094	Myo5b	0.139	0.599149386954389	-
6866002	Pcyox1l	0.139	0.599149386954389	-
6871380	Ttc9c	0.139	0.599149386954389	-
6875039	Il15ra	0.139	0.599149386954389	-
6886022	Hc	0.139	0.599149386954389	-
6892580	D630003M21Rik	0.139	0.599149386954389	-
6933652	Pop5	0.139	0.599149386954389	+
6941146	Adrbk2	0.139	0.599149386954389	-
6963422	Swap70	0.139	0.599149386954389	-
6981387	Unc5d	0.139	0.599149386954389	-
6992012	Rab6b	0.139	0.599149386954389	-
6756478	Lamb3	0.14	0.599149386954389	-
6770646	E2f7	0.14	0.599149386954389	-
6778432	Rhbdd3	0.14	0.599149386954389	-
6805399	Slc17a1	0.14	0.599149386954389	-
6819737	Pinx1	0.14	0.599149386954389	-
6825325	Mtmr9	0.14	0.599149386954389	-
6850192	Flot1	0.14	0.599149386954389	-
6851764	Ddx11	0.14	0.599149386954389	-
6856364	2610034M16Rik	0.14	0.599149386954389	-
6917232	--	0.14	0.599149386954389	-
6978233	Slc6a2	0.14	0.599149386954389	-
6979640	Trappc2l	0.14	0.599149386954389	-
7019508	Taf7l	0.14	0.599149386954389	-

6779327	Tmem17	0.141	0.599149386954389	-
6785394	Rptor	0.141	0.599149386954389	-
6806080	Bphl	0.141	0.599149386954389	-
6824633	Sall2	0.141	0.599149386954389	-
6850066	Tnxb	0.141	0.599149386954389	-
6855684	Abcc10	0.141	0.599149386954389	-
6862448	Pard6g	0.141	0.599149386954389	-
6872010	Anxa1	0.141	0.599149386954389	-
6898180	Iqcj	0.141	0.599149386954389	-
6912887	Nfx1	0.141	0.599149386954389	-
6912987	N28178	0.141	0.599149386954389	-
6931633	Cwh43	0.141	0.599149386954389	-
6931738	Gsx2	0.141	0.599149386954389	-
6932394	Areg	0.141	0.599149386954389	-
6932704	Prdm8	0.141	0.599149386954389	+
6961049	Otud7a	0.141	0.599149386954389	-
6962491	Sytl2	0.141	0.599149386954389	-
6968774	Rccd1	0.141	0.599149386954389	-
6969631	Aqp11	0.141	0.599149386954389	+
6976461	Aadat	0.141	0.599149386954389	-
6983225	Pik3r2	0.141	0.599149386954389	-
6985350	Clec18a	0.141	0.599149386954389	-
6985850	1190005I06Rik	0.141	0.599149386954389	-
6988672	Ift46	0.141	0.599149386954389	+
6989933	Spg21	0.141	0.599149386954389	-
6992022	Topbp1	0.141	0.599149386954389	-
7010434	Wdr44	0.141	0.599149386954389	-
6789684	Pafah1b1	0.142	0.599149386954389	-
6792666	D11Bwg0517e	0.142	0.599149386954389	-
6798223	Trmt61a	0.142	0.599149386954389	-
6813364	Uimc1	0.142	0.599149386954389	-
6859953	Kif20a	0.142	0.599149386954389	-
6864466	Epb4.1l4a	0.142	0.599149386954389	-
6866238	Ccbe1	0.142	0.599149386954389	+
6900821	Dpyd	0.142	0.599149386954389	-
6931182	Tbc1d1	0.142	0.599149386954389	-
6944351	Tes	0.142	0.599149386954389	-
6947987	Fbln2	0.142	0.599149386954389	-
6960226	Klk1b27	0.142	0.599149386954389	-
6966923	Bcl2l12	0.142	0.599149386954389	-
6983199	2810422J05Rik	0.142	0.599149386954389	-
6997555	4922501C03Rik	0.142	0.599149386954389	-
6749764	Gm973	0.143	0.599149386954389	-
6763235	Qsox1	0.143	0.599149386954389	-
6776173	Cradd	0.143	0.599149386954389	-
6792485	Galk1	0.143	0.599149386954389	-
6792832	Cd7	0.143	0.599149386954389	-
6824363	Atg14	0.143	0.599149386954389	-
6839665	Prkdc	0.143	0.599149386954389	-
6840766	Ildr1	0.143	0.599149386954389	-

6858575	Zeb1	0.143	0.599149386954389	-
6879020	Sfpi1	0.143	0.599149386954389	-
6910129	Clca4	0.143	0.599149386954389	-
6942692	Tmem184a	0.143	0.599149386954389	-
6950792	Cmas	0.143	0.599149386954389	-
6747871	Crispld1	0.144	0.599149386954389	+
6764580	Psen2	0.144	0.599149386954389	-
6798601	Ncoa1	0.144	0.599149386954389	-
6807266	Pcbd2	0.144	0.599149386954389	-
6813676	Zcchc6	0.144	0.599149386954389	-
6819284	Nfatc4	0.144	0.599149386954389	-
6823451	Slmap	0.144	0.599149386954389	-
6826285	Lect1	0.144	0.599149386954389	-
6829451	Dnahc5	0.144	0.599149386954389	-
6855567	Runx2	0.144	0.599149386954389	-
6861341	Cd74	0.144	0.599149386954389	-
6879637	Lmo2	0.144	0.599149386954389	-
6883770	Edn3	0.144	0.599149386954389	-
6884415	Prpf18	0.144	0.599149386954389	-
6904130	Ccdc39	0.144	0.599149386954389	-
6906858	Ubap2l	0.144	0.599149386954389	-
6908487	Gpr88	0.144	0.599149386954389	-
6910275	Spata1	0.144	0.599149386954389	-
6924295	Dio1	0.144	0.599149386954389	-
6925904	Trnp1	0.144	0.599149386954389	+
6939731	Csn2	0.144	0.599149386954389	-
6946204	Snx10	0.144	0.599149386954389	-
6958855	Tmem160	0.144	0.599149386954389	-
6959321	C030039L03Rik	0.144	0.599149386954389	-
6964392	Armc5	0.144	0.599149386954389	-
6972425	Nadsyn1	0.144	0.599149386954389	-
6973682	1700019B03Rik	0.144	0.599149386954389	-
6977058	Myo9b	0.144	0.599149386954389	+
7020425	Cnksr2	0.144	0.599149386954389	-
6784056	Rara	0.145	0.599149386954389	+
6790079	Crlf3	0.145	0.599149386954389	-
6810067	Pde4d	0.145	0.599149386954389	-
6840782	Polq	0.145	0.599149386954389	-
6854896	Zfp811	0.145	0.599149386954389	-
6885414	Ptgds	0.145	0.599149386954389	-
6905711	Shox2	0.145	0.599149386954389	-
6931243	Wdr19	0.145	0.599149386954389	+
6932335	Alb	0.145	0.599149386954389	-
6935128	Stag3	0.145	0.599149386954389	-
6962153	Tmc3	0.145	0.599149386954389	-
6971409	Zfp668	0.145	0.599149386954389	-
6977779	Hook2	0.145	0.599149386954389	-
6990526	Arpp19	0.145	0.599149386954389	+
6995503	Pts	0.145	0.599149386954389	-
6748620	Arid5a	0.146	0.599149386954389	+

6758943	Ppil3	0.146	0.599149386954389	-
6782246	Ggt6	0.146	0.599149386954389	-
6823666	Prkcd	0.146	0.599149386954389	-
6839416	Tnfrsf17	0.146	0.599149386954389	-
6874979	Prkcq	0.146	0.599149386954389	-
6876154	Abl1	0.146	0.599149386954389	-
6913619	Zfp462	0.146	0.599149386954389	-
6916492	Cyp4a12a	0.146	0.599149386954389	-
6921353	Xpa	0.146	0.599149386954389	-
6924718	Cyp4a14	0.146	0.599149386954389	-
6926988	H6pd	0.146	0.599149386954389	-
6929648	Tmem214	0.146	0.599149386954389	-
6939967	G3bp2	0.146	0.599149386954389	-
6949076	Brpf1	0.146	0.599149386954389	-
6959254	Cyp2b13	0.146	0.599149386954389	-
6971271	Atxn2l	0.146	0.599149386954389	-
6976986	Sugp2	0.146	0.599149386954389	-
6996375	Trip4	0.146	0.599149386954389	-
6751330	Chrng	0.147	0.599149386954389	-
6752222	Cdh7	0.147	0.599149386954389	-
6775743	Bpil2	0.147	0.599149386954389	-
6824893	Cenpj	0.147	0.599149386954389	-
6837783	Hdac10	0.147	0.599149386954389	-
6838098	Adamts20	0.147	0.599149386954389	-
6869407	Rpp30	0.147	0.599149386954389	-
6873663	Sorcs1	0.147	0.599149386954389	-
6942557	Mospd3	0.147	0.599149386954389	-
6953809	Crhr2	0.147	0.599149386954389	-
6985900	Slc7a5	0.147	0.599149386954389	+
6990398	Mns1	0.147	0.599149386954389	-
6992439	Kif9	0.147	0.599149386954389	-
6994954	Arhgef12	0.147	0.599149386954389	-
7019833	Col4a6	0.147	0.599149386954389	-
6749706	Aox4	0.148	0.599149386954389	-
6755687	Acbd3	0.148	0.599149386954389	-
6757809	Prss40	0.148	0.599149386954389	-
6773556	Nr2e1	0.148	0.599149386954389	-
6784238	Hsd17b1	0.148	0.599149386954389	-
6805158	Elmo1	0.148	0.599149386954389	+
6839607	Abcc1	0.148	0.599149386954389	-
6892496	Dsn1	0.148	0.599149386954389	-
6908775	Slc44a3	0.148	0.599149386954389	-
6928741	Abcb1b	0.148	0.599149386954389	-
6937466	Sorcs2	0.148	0.599149386954389	-
6940933	Slc26a1	0.148	0.599149386954389	-
6953749	9130019P16Rik	0.148	0.599149386954389	-
6956886	Bms1	0.148	0.599149386954389	-
6957352	BC048546	0.148	0.599149386954389	-
6978868	Thap11	0.148	0.599149386954389	-
7010334	Rbm10	0.148	0.599149386954389	-

7012015	Ikbkg	0.148	0.599149386954389	-
6753058	Fam72a	0.149	0.599149386954389	-
6759800	Dnpep	0.149	0.599149386954389	-
6776507	4930430F08Rik	0.149	0.599149386954389	-
6779843	Sh3pxd2b	0.149	0.599149386954389	+
6780779	Tbc1d9b	0.149	0.599149386954389	-
6782083	Neurl4	0.149	0.599149386954389	-
6796813	Nrxn3	0.149	0.599149386954389	-
6871536	1700025F22Rik	0.149	0.599149386954389	-
6882586	Spag4	0.149	0.599149386954389	-
6882670	Src	0.149	0.599149386954389	-
6888296	Calcrl	0.149	0.599149386954389	-
6918049	Aldh4a1	0.149	0.599149386954389	-
6953429	Tmem176b	0.149	0.599149386954389	-
6965084	Paox	0.149	0.599149386954389	-
6966265	Tbcb	0.149	0.599149386954389	-
6971023	Ears2	0.149	0.599149386954389	-
6992613	Clasp2	0.149	0.599149386954389	-
6998606	Mapkapk3	0.149	0.599149386954389	-
6775376	Atp8b3	0.15	0.599149386954389	-
6799054	Mycn	0.15	0.599149386954389	-
6816080	Map3k1	0.15	0.599149386954389	-
6818051	Galntl2	0.15	0.599149386954389	-
6836602	Ptk2	0.15	0.599149386954389	-
6838256	Slc38a1	0.15	0.599149386954389	-
6838346	Col2a1	0.15	0.599149386954389	-
6839864	Ufd1l	0.15	0.599149386954389	-
6844531	Ehhadh	0.15	0.599149386954389	-
6859946	Wnt8a	0.15	0.599149386954389	-
6867980	Eml3	0.15	0.599149386954389	-
6871393	AI462493	0.15	0.599149386954389	-
6878655	Itgav	0.15	0.599149386954389	+
6888752	Nr1h3	0.15	0.599149386954389	-
6900270	4933421E11Rik	0.15	0.599149386954389	-
6908919	Usp53	0.15	0.599149386954389	+
6919209	Tnfrsf4	0.15	0.599149386954389	-
6921229	Pax5	0.15	0.599149386954389	-
6925640	Hcrtr1	0.15	0.599149386954389	-
6926160	Ephb2	0.15	0.599149386954389	-
6926851	Miip	0.15	0.599149386954389	-
6947814	Mgll	0.15	0.599149386954389	-
6964274	AI467606	0.15	0.599149386954389	-
6981125	Tacc1	0.15	0.599149386954389	-
6995718	Cul5	0.15	0.599149386954389	-
7000773	Zfp131	0.15	0.599149386954389	-
6755700	BC031781	0.151	0.599149386954389	-
6763520	Dars2	0.151	0.599149386954389	-
6771655	Ankrd52	0.151	0.599149386954389	-
6781300	Butr1	0.151	0.599149386954389	-
6790774	Tom1l1	0.151	0.599149386954389	-

6843378	Ripply3	0.151	0.599149386954389	-
6854546	Spdef	0.151	0.599149386954389	-
6858563	Svil	0.151	0.599149386954389	-
6878657	Fam171b	0.151	0.599149386954389	-
6918205	Casp9	0.151	0.599149386954389	-
6922335	Tnc	0.151	0.599149386954389	-
6925590	Txlna	0.151	0.599149386954389	-
6929457	Dpp6	0.151	0.599149386954389	-
6958654	Nlrp4c	0.151	0.599149386954389	-
6962054	Pde8a	0.151	0.599149386954389	-
6965586	2810007J24Rik	0.151	0.599149386954389	-
6972410	Cars	0.151	0.599149386954389	-
6987490	Bmper	0.151	0.599149386954389	-
6773802	Grik2	0.152	0.599149386954389	+
6778077	Rdh5	0.152	0.599149386954389	-
6781257	Sh3bp5l	0.152	0.599149386954389	-
6785726	Ccdc117	0.152	0.599149386954389	-
6791384	Krt33a	0.152	0.599149386954389	-
6906441	Trim2	0.152	0.599149386954389	-
6945749	Sval2	0.152	0.599149386954389	-
6959780	Pepd	0.152	0.599149386954389	-
6963163	Dub1	0.152	0.599149386954389	-
6788789	Top3a	0.153	0.599149386954389	-
6792786	Pcyt2	0.153	0.599149386954389	-
6800675	Prkd1	0.153	0.599149386954389	-
6813797	Fbp1	0.153	0.599149386954389	-
6824492	1700011H14Rik	0.153	0.599149386954389	-
6841372	C330027C09Rik	0.153	0.599149386954389	-
6877740	Csrnp3	0.153	0.599149386954389	-
6886017	Phf19	0.153	0.599149386954389	-
6917813	Asap3	0.153	0.599149386954389	-
6926414	Crocc	0.153	0.599149386954389	-
6967914	Tjp1	0.153	0.599149386954389	-
6989175	Sln	0.153	0.599149386954389	-
7017029	Mtap7d3	0.153	0.599149386954389	-
6755174	Pfdn2	0.154	0.599149386954389	-
6804893	Nid1	0.154	0.599149386954389	-
6855615	Slc29a1	0.154	0.599149386954389	-
6861200	Slc12a2	0.154	0.599149386954389	-
6870919	Ighmbp2	0.154	0.599149386954389	-
6883640	Spo11	0.154	0.599149386954389	-
6915817	Cachd1	0.154	0.599149386954389	-
6923387	5830433M19Rik	0.154	0.599149386954389	-
6929694	Nrbp1	0.154	0.599149386954389	-
6931529	Atp10d	0.154	0.599149386954389	-
6940932	Dgkq	0.154	0.599149386954389	-
6964232	Ccdc101	0.154	0.599149386954389	-
6968735	Anpep	0.154	0.599149386954389	-
6970164	Gvin1	0.154	0.599149386954389	-
6979680	Dpep1	0.154	0.599149386954389	-

6980949	Slc25a15	0.154	0.599149386954389	-
7014702	Acot9	0.154	0.599149386954389	-
7019790	Rbm41	0.154	0.599149386954389	-
6753353	Lmod1	0.155	0.599149386954389	-
6755711	Pycr2	0.155	0.599149386954389	-
6756149	Ush2a	0.155	0.599149386954389	-
6769199	Efna2	0.155	0.599149386954389	-
6770722	Phlda1	0.155	0.599149386954389	-
6773622	1700021F05Rik	0.155	0.599149386954389	-
6782458	Crk	0.155	0.599149386954389	-
6785437	Ccdc137	0.155	0.599149386954389	-
6791989	Bptf	0.155	0.599149386954389	-
6792738	Azi1	0.155	0.599149386954389	-
6813110	Cenpp	0.155	0.599149386954389	-
6863301	Rock1	0.155	0.599149386954389	-
6882059	Cstl1	0.155	0.599149386954389	-
6901335	Casp6	0.155	0.599149386954389	-
6903542	Hps3	0.155	0.599149386954389	-
6918692	Nppa	0.155	0.599149386954389	-
6935064	Ephb4	0.155	0.599149386954389	-
6939268	Pdcl2	0.155	0.599149386954389	-
6960423	Tmem143	0.155	0.599149386954389	-
6962027	Zscan2	0.155	0.599149386954389	-
6964075	Slc5a11	0.155	0.599149386954389	-
6964371	Myst1	0.155	0.599149386954389	-
6965038	Lrrc27	0.155	0.599149386954389	-
6965598	Ehd2	0.155	0.599149386954389	-
6982690	Sap30	0.155	0.599149386954389	-
6982725	Galntl6	0.155	0.599149386954389	-
6995711	Atm	0.155	0.599149386954389	-
7010644	Dock11	0.155	0.599149386954389	+
6751339	Neu2	0.156	0.599149386954389	-
6753273	Cyb5r1	0.156	0.599149386954389	-
6768358	Ctnna3	0.156	0.599149386954389	-
6791170	Lrrc46	0.156	0.599149386954389	-
6791472	Ezh1	0.156	0.599149386954389	-
6833408	Myg1	0.156	0.599149386954389	-
6852034	Lpin2	0.156	0.599149386954389	+
6871429	Asrgl1	0.156	0.599149386954389	+
6871837	Vps13a	0.156	0.599149386954389	-
6876089	Usp20	0.156	0.599149386954389	-
6885422	Traf2	0.156	0.599149386954389	-
6903558	Cpb1	0.156	0.599149386954389	-
6916952	Heyl	0.156	0.599149386954389	-
6924832	Plk3	0.156	0.599149386954389	+
6931857	Polr2b	0.156	0.599149386954389	-
6942460	Styxl1	0.156	0.599149386954389	-
6957405	Clec1a	0.156	0.599149386954389	-
6976212	Spata4	0.156	0.599149386954389	-
6982102	Tlr3	0.156	0.599149386954389	+

6995938	Arid3b	0.156	0.599149386954389	-
6754691	F5	0.157	0.599149386954389	-
6759549	Ikzf2	0.157	0.599149386954389	-
6782680	Rab34	0.157	0.599149386954389	-
6784330	Asb16	0.157	0.599149386954389	-
6837046	Card10	0.157	0.599149386954389	-
6849581	Kctd20	0.157	0.599149386954389	-
6863934	Ino80c	0.157	0.599149386954389	-
6864761	Hdac3	0.157	0.599149386954389	-
6866118	Atp8b1	0.157	0.599149386954389	-
6876570	Arpc5l	0.157	0.599149386954389	-
6919304	Plag1	0.157	0.599149386954389	-
6925077	Zfp69	0.157	0.599149386954389	-
6956773	Tmem72	0.157	0.599149386954389	-
6992276	Camkv	0.157	0.599149386954389	+
6762671	Cfh	0.158	0.599149386954389	-
6768978	Pofut2	0.158	0.599149386954389	-
6788796	Gm16515	0.158	0.599149386954389	-
6811554	Prss16	0.158	0.599149386954389	-
6828522	Lifr	0.158	0.599149386954389	-
6844327	Ranbp1	0.158	0.599149386954389	-
6845912	Gramd1c	0.158	0.599149386954389	-
6852750	Plekhh2	0.158	0.599149386954389	+
6857167	Galnt14	0.158	0.599149386954389	+
6860903	Srfbp1	0.158	0.599149386954389	-
6881794	Banf2	0.158	0.599149386954389	-
6899025	Sh2d2a	0.158	0.599149386954389	-
6908036	Fam40a	0.158	0.599149386954389	-
6911914	Calb1	0.158	0.599149386954389	+
6919879	Slc26a7	0.158	0.599149386954389	-
6920988	Smu1	0.158	0.599149386954389	-
6927227	2810405K02Rik	0.158	0.599149386954389	-
6941031	P2rx2	0.158	0.599149386954389	-
6942487	Lrwd1	0.158	0.599149386954389	-
6959267	Nlrp4a	0.158	0.599149386954389	-
6959808	Ccdc123	0.158	0.599149386954389	-
6974610	Vps36	0.158	0.599149386954389	-
6978185	Irx3	0.158	0.599149386954389	-
6986072	Ttc13	0.158	0.599149386954389	-
6987446	Zfp809	0.158	0.599149386954389	-
6763652	Gorab	0.159	0.599149386954389	-
6766577	Vnn3	0.159	0.599149386954389	-
6780266	Nudcd2	0.159	0.599149386954389	-
6785752	Myl7	0.159	0.599149386954389	-
6791921	Ern1	0.159	0.599149386954389	-
6796339	Rdh12	0.159	0.599149386954389	-
6807074	Sykb	0.159	0.599149386954389	-
6813761	Ctsr	0.159	0.599149386954389	-
6814348	Slc6a19	0.159	0.599149386954389	-
6815046	Zfyve16	0.159	0.599149386954389	-

6855935	Tbc1d5	0.159	0.599149386954389	-
6871580	Stx3	0.159	0.599149386954389	-
6878713	Slc43a3	0.159	0.599149386954389	-
6880423	Eif2ak4	0.159	0.599149386954389	-
6882318	Cox4i2	0.159	0.599149386954389	-
6918125	Padi2	0.159	0.599149386954389	-
6933337	Idua	0.159	0.599149386954389	-
6950727	Slco1b2	0.159	0.599149386954389	-
6959101	Zfp108	0.159	0.599149386954389	+
6964172	D430042O09Rik	0.159	0.599149386954389	-
6970380	BC051019	0.159	0.599149386954389	-
6983333	Med26	0.159	0.599149386954389	-
6984989	Acd	0.159	0.599149386954389	-
6998595	Rad54l2	0.159	0.599149386954389	-
7011405	Vgll1	0.159	0.599149386954389	-
6757282	Mcm3	0.16	0.599149386954389	-
6805430	Cmah	0.16	0.599149386954389	-
6806043	Serpinb9d	0.16	0.599149386954389	-
6818944	Pnp	0.16	0.599149386954389	-
6846576	Epha6	0.16	0.599149386954389	-
6849285	Abca3	0.16	0.599149386954389	-
6871109	Drap1	0.16	0.599149386954389	-
6873897	Zdhhc6	0.16	0.599149386954389	-
6878031	Itga6	0.16	0.599149386954389	-
6882292	6820408C15Rik	0.16	0.599149386954389	-
6882377	Kif3b	0.16	0.599149386954389	-
6885514	Slc2a6	0.16	0.599149386954389	-
6889261	Pdhx	0.16	0.599149386954389	-
6910709	Acadm	0.16	0.599149386954389	-
6944988	Fam71f1	0.16	0.599149386954389	-
6949727	Clec4a3	0.16	0.599149386954389	-
6953343	Zfp786	0.16	0.599149386954389	-
6963895	Acsm3	0.16	0.599149386954389	-
6969753	Slco2b1	0.16	0.599149386954389	-
6983232	Kcnn1	0.16	0.599149386954389	-
6992900	Ttc21a	0.16	0.599149386954389	-
7017604	Renbp	0.16	0.599149386954389	-
6755163	Nr1i3	0.161	0.599149386954389	-
6767631	Hace1	0.161	0.599149386954389	-
6775329	ORF61	0.161	0.599149386954389	-
6781252	Zfp692-ps	0.161	0.599149386954389	-
6782701	Slc46a1	0.161	0.599149386954389	-
6819269	Gmpr2	0.161	0.599149386954389	-
6828642	Ranbp3l	0.161	0.599149386954389	-
6843419	Kcnj15	0.161	0.599149386954389	-
6856239	Lonp1	0.161	0.599149386954389	-
6901609	Manba	0.161	0.599149386954389	-
6901732	Rg9mtd2	0.161	0.599149386954389	-
6906220	Etfdh	0.161	0.599149386954389	-
6910611	Dnajb4	0.161	0.599149386954389	+

6917270	Ak2	0.161	0.599149386954389	-
6923525	Cyp2j9	0.161	0.599149386954389	-
6929297	Agap3	0.161	0.599149386954389	-
6943818	Col1a2	0.161	0.599149386954389	-
6950125	Clec2d	0.161	0.599149386954389	-
6954994	Htra2	0.161	0.599149386954389	-
6956241	Crbn	0.161	0.599149386954389	-
6957252	Rad51ap1	0.161	0.599149386954389	-
6958058	Gys2	0.161	0.599149386954389	-
6962736	Odz4	0.161	0.599149386954389	-
6987382	Dnm2	0.161	0.599149386954389	-
6747850	Gdap1	0.162	0.599149386954389	-
6749392	Stat4	0.162	0.599149386954389	-
6769213	Plk5	0.162	0.599149386954389	+
6796305	Mpp5	0.162	0.599149386954389	-
6815437	Tnpo1	0.162	0.599149386954389	-
6820409	Ccdc122	0.162	0.599149386954389	-
6836803	BC024139	0.162	0.599149386954389	-
6854874	Wiz	0.162	0.599149386954389	-
6855083	Bat2	0.162	0.599149386954389	-
6855144	Atat1	0.162	0.599149386954389	-
6871509	Cd5	0.162	0.599149386954389	-
6875789	Gpsm1	0.162	0.599149386954389	-
6906895	Npr1	0.162	0.599149386954389	-
6917215	Zmym6	0.162	0.599149386954389	-
6917353	Bai2	0.162	0.599149386954389	-
6931708	Fip1l1	0.162	0.599149386954389	-
6945113	Cpa5	0.162	0.599149386954389	-
6945624	Ndufb2	0.162	0.599149386954389	-
6950394	Gprc5a	0.162	0.599149386954389	-
7020683	Bmx	0.162	0.599149386954389	-
6748546	Fam123c	0.163	0.599149386954389	-
6750105	Mtap2	0.163	0.599149386954389	-
6754014	Ivns1abp	0.163	0.599149386954389	+
6757157	Jph1	0.163	0.599149386954389	-
6758920	Kctd18	0.163	0.599149386954389	-
6767066	Sult3a1	0.163	0.599149386954389	-
6769515	Fbxo7	0.163	0.599149386954389	-
6779432	Bcl11a	0.163	0.599149386954389	-
6790648	Akap1	0.163	0.599149386954389	-
6798404	Ptprn2	0.163	0.599149386954389	-
6801500	Nin	0.163	0.599149386954389	-
6836839	Slc39a4	0.163	0.599149386954389	-
6836875	Zfp647	0.163	0.599149386954389	-
6841110	Ccdc52	0.163	0.599149386954389	-
6852874	Ttc7	0.163	0.599149386954389	-
6855829	Nfya	0.163	0.599149386954389	-
6857183	Xdh	0.163	0.599149386954389	-
6858033	Kcnk12	0.163	0.599149386954389	-
6896332	Spata16	0.163	0.599149386954389	-

6925360	Adprhl2	0.163	0.599149386954389	-
6966985	Bax	0.163	0.599149386954389	-
6968845	Homer2	0.163	0.599149386954389	-
6989020	Pih1d2	0.163	0.599149386954389	-
6992215	Cacna2d2	0.163	0.599149386954389	-
6998125	Clstn2	0.163	0.599149386954389	-
7012305	Dmd	0.163	0.599149386954389	-
7016751	Enox2	0.163	0.599149386954389	-
6765977	Epm2a	0.164	0.599149386954389	-
6771884	Esr1	0.164	0.599149386954389	-
6789233	Odf4	0.164	0.599149386954389	-
6826842	Klhl1	0.164	0.599149386954389	-
6831845	Cdc42ep1	0.164	0.599149386954389	-
6832572	Miox	0.164	0.599149386954389	-
6837545	Smc1b	0.164	0.599149386954389	-
6850819	Trem3	0.164	0.599149386954389	-
6854792	Rsph1	0.164	0.599149386954389	-
6854999	Ring1	0.164	0.599149386954389	-
6872797	Pank1	0.164	0.599149386954389	-
6894231	Ythdf1	0.164	0.599149386954389	-
6909509	Npnt	0.164	0.599149386954389	-
6933405	Chfr	0.164	0.599149386954389	-
6935232	Mafk	0.164	0.599149386954389	-
6962045	Alpk3	0.164	0.599149386954389	-
6994149	Acad8	0.164	0.599149386954389	-
7013342	Apool	0.164	0.599149386954389	-
6751063	Rhbdd1	0.165	0.599149386954389	-
6758437	Gm3852	0.165	0.599149386954389	-
6758450	Slc39a10	0.165	0.599149386954389	-
6762967	Prg4	0.165	0.599149386954389	-
6764038	Fcgr2b	0.165	0.599149386954389	-
6778044	Erbb3	0.165	0.599149386954389	-
6781321	Mrpl55	0.165	0.599149386954389	-
6781440	4933439F18Rik	0.165	0.599149386954389	-
6798232	Klc1	0.165	0.599149386954389	-
6831994	Atf4	0.165	0.599149386954389	+
6836888	Rbfox2	0.165	0.599149386954389	-
6867623	Tbx10	0.165	0.599149386954389	-
6873149	Slit1	0.165	0.599149386954389	-
6887234	Fign	0.165	0.599149386954389	-
6890387	Catsper2	0.165	0.599149386954389	-
6904264	Mccc1	0.165	0.599149386954389	-
6917180	Clspn	0.165	0.599149386954389	-
6924281	Mrpl37	0.165	0.599149386954389	-
6943822	Casd1	0.165	0.599149386954389	-
6946018	AI854703	0.165	0.599149386954389	-
6960266	Syt3	0.165	0.599149386954389	+
6982852	Anxa10	0.165	0.599149386954389	-
6982936	Trim61	0.165	0.599149386954389	-
7014530	Fgd1	0.165	0.599149386954389	-

6756763	Sntg1	0.166	0.599149386954389	-
6767886	Asf1a	0.166	0.599149386954389	-
6775149	Gstt3	0.166	0.599149386954389	-
6782224	Txndc17	0.166	0.599149386954389	-
6784399	Acbd4	0.166	0.599149386954389	-
6796595	Zfp410	0.166	0.599149386954389	-
6797241	Zc3h14	0.166	0.599149386954389	-
6800968	Mbip	0.166	0.599149386954389	-
6801902	Spnb1	0.166	0.599149386954389	-
6817955	Itih4	0.166	0.599149386954389	-
6823336	Polr3a	0.166	0.599149386954389	-
6847805	2610039C10Rik	0.166	0.599149386954389	-
6880853	Dut	0.166	0.599149386954389	-
6892579	Tgm2	0.166	0.599149386954389	-
6903487	Pde7a	0.166	0.599149386954389	-
6908748	Tmem56	0.166	0.599149386954389	-
6955545	Xpc	0.166	0.599149386954389	-
6970392	Scube2	0.166	0.599149386954389	-
6985660	Pkd1l2	0.166	0.599149386954389	-
6988033	Kirrel3	0.166	0.599149386954389	-
7018304	Las1l	0.166	0.599149386954389	-
7018774	Fndc3c1	0.166	0.599149386954389	-
6754900	Rxrg	0.167	0.599149386954389	-
6757035	Eya1	0.167	0.599149386954389	-
6769263	Map2k2	0.167	0.599149386954389	-
6777974	Hsd17b6	0.167	0.599149386954389	-
6785768	Npc1l1	0.167	0.599149386954389	-
6796500	Rgs6	0.167	0.599149386954389	+
6815870	Ndufaf2	0.167	0.599149386954389	-
6843690	Nagpa	0.167	0.599149386954389	-
6852052	Spdya	0.167	0.599149386954389	-
6856278	Dennd1c	0.167	0.599149386954389	-
6864708	Dnd1	0.167	0.599149386954389	-
6881115	Polr1b	0.167	0.599149386954389	-
6881205	Oxt	0.167	0.599149386954389	-
6895832	Slc7a12	0.167	0.599149386954389	-
6925032	Foxo6	0.167	0.599149386954389	-
6929856	Htt	0.167	0.599149386954389	-
6939162	Usp46	0.167	0.599149386954389	-
6957436	Klra6	0.167	0.599149386954389	-
6966183	Ryr1	0.167	0.599149386954389	-
6978761	Car7	0.167	0.599149386954389	-
7011776	Aff2	0.167	0.599149386954389	-
7012696	Heph	0.167	0.599149386954389	-
7019573	Nxf7	0.167	0.599149386954389	-
6753917	Ptgs2	0.168	0.599149386954389	+
6782177	Mis12	0.168	0.599149386954389	-
6811260	5033411D12Rik	0.168	0.599149386954389	-
6816317	Parp8	0.168	0.599149386954389	-
6846439	Gpr128	0.168	0.599149386954389	-

6852235	Rasgrp3	0.168	0.599149386954389	-
6887520	Spc25	0.168	0.599149386954389	-
6898010	Mme	0.168	0.599149386954389	-
6963912	Abca14	0.168	0.599149386954389	-
6971359	Zfp689	0.168	0.599149386954389	-
6979096	Hydin	0.168	0.599149386954389	-
6980392	Ankrd10	0.168	0.599149386954389	-
6981573	Wrn	0.168	0.599149386954389	-
6985373	Wdr59	0.168	0.599149386954389	-
6988112	Cdon	0.168	0.599149386954389	-
6992373	Celsr3	0.168	0.599149386954389	-
6996956	Scg3	0.168	0.599149386954389	-
6751103	Ccl20	0.169	0.599149386954389	-
6752156	Serpinb2	0.169	0.599149386954389	-
6755819	Srp9	0.169	0.599149386954389	-
6757982	Mitd1	0.169	0.599149386954389	-
6769117	Vmn2r81	0.169	0.599149386954389	-
6781515	Mfap4	0.169	0.599149386954389	-
6782368	1300001I01Rik	0.169	0.599149386954389	-
6791132	Snx11	0.169	0.599149386954389	-
6791836	March10	0.169	0.599149386954389	-
6809812	Cenpk	0.169	0.599149386954389	-
6840725	Hspbap1	0.169	0.599149386954389	-
6843642	Srl	0.169	0.599149386954389	-
6847190	Samsn1	0.169	0.599149386954389	-
6856182	Zfp119a	0.169	0.599149386954389	-
6888786	Dgkz	0.169	0.599149386954389	-
6895855	Car3	0.169	0.599149386954389	-
6923058	Haus6	0.169	0.599149386954389	-
6937881	Fam184b	0.169	0.599149386954389	-
6954344	Vmn1r32	0.169	0.599149386954389	-
6992306	Gmppb	0.169	0.599149386954389	-
6785206	2310004N24Rik	0.17	0.599149386954389	-
6785799	Myo1g	0.17	0.599149386954389	-
6793225	Pum2	0.17	0.599149386954389	-
6800060	Ahr	0.17	0.599149386954389	-
6822285	Ubac2	0.17	0.599149386954389	-
6831591	Gpihbp1	0.17	0.599149386954389	-
6838652	Gm5414	0.17	0.599149386954389	-
6843953	Gspt1	0.17	0.599149386954389	-
6850831	B430306N03Rik	0.17	0.599149386954389	-
6856682	Ralbp1	0.17	0.599149386954389	-
6873150	Arhgap19	0.17	0.599149386954389	-
6888151	Frzb	0.17	0.599149386954389	+
6930361	Cc2d2a	0.17	0.599149386954389	+
6931217	Klf3	0.17	0.599149386954389	-
6933257	A830010M20Rik	0.17	0.599149386954389	-
6941685	Aldh2	0.17	0.599149386954389	-
6942558	Pcolce	0.17	0.599149386954389	-
6949073	Mtmr14	0.17	0.599149386954389	-

6955350	Kbtbd12	0.17	0.599149386954389	-
6958933	Psg29	0.17	0.599149386954389	-
6963521	Usp47	0.17	0.599149386954389	-
6981604	Rbpms	0.17	0.599149386954389	-
6983837	Zswim4	0.17	0.599149386954389	-
6985943	Galns	0.17	0.599149386954389	-
6989815	Aagab	0.17	0.599149386954389	-
6995799	Chrna3	0.17	0.599149386954389	-
7017156	Atp11c	0.17	0.599149386954389	-
6752132	Serpinb5	0.171	0.599149386954389	-
6818722	Ktn1	0.171	0.599149386954389	-
6819842	Ints9	0.171	0.599149386954389	-
6849771	Ubash3a	0.171	0.599149386954389	-
6875901	Rxra	0.171	0.599149386954389	-
6901737	Adh1	0.171	0.599149386954389	-
6917441	Srsf4	0.171	0.599149386954389	-
6933303	Ccdc18	0.171	0.599149386954389	-
6936082	Dbf4	0.171	0.599149386954389	-
6945675	Wee2	0.171	0.599149386954389	-
6945778	Casp2	0.171	0.599149386954389	-
6966249	Zfp14	0.171	0.599149386954389	+
6977713	4930432K21Rik	0.171	0.599149386954389	-
6755170	B4galt3	0.172	0.599149386954389	-
6755630	Sccpdh	0.172	0.599149386954389	-
6770743	Krr1	0.172	0.599149386954389	-
6771568	R3hdm2	0.172	0.599149386954389	-
6780570	Havcr1	0.172	0.599149386954389	-
6813752	Ctsq	0.172	0.599149386954389	-
6820085	Hr	0.172	0.599149386954389	-
6835981	Tatdn1	0.172	0.599149386954389	-
6843355	Sim2	0.172	0.599149386954389	-
6844316	Prodh	0.172	0.599149386954389	-
6846010	Cd96	0.172	0.599149386954389	-
6864696	Apbb3	0.172	0.599149386954389	-
6867785	Ltbp3	0.172	0.599149386954389	-
6871496	Tmem138	0.172	0.599149386954389	-
6880621	Wdr76	0.172	0.599149386954389	-
6916630	Tmem53	0.172	0.599149386954389	-
6916998	Rragc	0.172	0.599149386954389	-
6921375	Hemgn	0.172	0.599149386954389	-
6924465	--	0.172	0.599149386954389	-
6933058	Dmp1	0.172	0.599149386954389	-
6935777	Kl	0.172	0.599149386954389	-
6947381	Loxl3	0.172	0.599149386954389	-
6952766	Creb3l2	0.172	0.599149386954389	+
6960375	Gys1	0.172	0.599149386954389	-
6964730	2700050L05Rik	0.172	0.599149386954389	-
6970919	Zp2	0.172	0.599149386954389	-
6994586	Tirap	0.172	0.599149386954389	-
7018522	Tex11	0.172	0.599149386954389	-

6749804	Carf	0.173	0.599149386954389	-
6755227	Dcaf8	0.173	0.599149386954389	-
6786985	4930505A04Rik	0.173	0.599149386954389	-
6797969	Meg3	0.173	0.599149386954389	-
6807899	Fastkd3	0.173	0.599149386954389	-
6814385	Pdcd6	0.173	0.599149386954389	-
6825603	Adam28	0.173	0.599149386954389	-
6841149	Cd200r3	0.173	0.599149386954389	-
6845377	Dtx3l	0.173	0.599149386954389	-
6849466	A930001N09Rik	0.173	0.599149386954389	-
6854286	Prss33	0.173	0.599149386954389	-
6862062	Mro	0.173	0.599149386954389	-
6868023	1810006K21Rik	0.173	0.599149386954389	-
6873154	Rrp12	0.173	0.599149386954389	-
6876058	Dolpp1	0.173	0.599149386954389	-
6878282	Hoxd4	0.173	0.599149386954389	-
6881291	Prnd	0.173	0.599149386954389	-
6887380	Scn7a	0.173	0.599149386954389	-
6899000	Fcrl5	0.173	0.599149386954389	-
6933441	--	0.173	0.599149386954389	-
6941647	Oas2	0.173	0.599149386954389	-
6950392	Ddx47	0.173	0.599149386954389	-
6957406	Klrk1	0.173	0.599149386954389	-
6960627	Slc6a5	0.173	0.599149386954389	-
6973288	Zfp324	0.173	0.599149386954389	-
6998643	Slc38a3	0.173	0.599149386954389	-
7012843	Gdpd2	0.173	0.599149386954389	-
6765235	Dtl	0.174	0.599149386954389	-
6768227	Lrrc20	0.174	0.599149386954389	-
6779270	Vps54	0.174	0.599149386954389	-
6802352	Ltbp2	0.174	0.599149386954389	-
6806882	Id4	0.174	0.599149386954389	-
6813078	Phf2	0.174	0.599149386954389	-
6864709	Hars	0.174	0.599149386954389	-
6866545	Dcc	0.174	0.599149386954389	+
6875966	Ralgds	0.174	0.599149386954389	-
6892831	Serinc3	0.174	0.599149386954389	-
6901136	Arsj	0.174	0.599149386954389	-
6902213	Uox	0.174	0.599149386954389	-
6907202	Adamtsl4	0.174	0.599149386954389	-
6907346	Chd1l	0.174	0.599149386954389	-
6912520	Gabrr2	0.174	0.599149386954389	-
6913042	Glipr2	0.174	0.599149386954389	-
6919063	Wdr8	0.174	0.599149386954389	-
6929591	Il6	0.174	0.599149386954389	-
6935534	Wasf3	0.174	0.599149386954389	-
6955019	Wdr54	0.174	0.599149386954389	-
6977684	Ptger1	0.174	0.599149386954389	-
6979607	Banp	0.174	0.599149386954389	-
7011996	Emd	0.174	0.599149386954389	+

6755177	Usf1	0.175	0.599149386954389	-
6755216	Slamf6	0.175	0.599149386954389	-
6766372	Mtap7	0.175	0.599149386954389	-
6771703	Mmp19	0.175	0.599149386954389	-
6789789	Rph3al	0.175	0.599149386954389	-
6807650	0610007P08Rik	0.175	0.599149386954389	-
6817738	Erc2	0.175	0.599149386954389	-
6844112	Myh11	0.175	0.599149386954389	-
6870785	Slc18a2	0.175	0.599149386954389	-
6875803	Egfl7	0.175	0.599149386954389	-
6882996	Ift52	0.175	0.599149386954389	-
6883331	A530013C23Rik	0.175	0.599149386954389	-
6890985	1700037H04Rik	0.175	0.599149386954389	-
6906224	Rxfp1	0.175	0.599149386954389	-
6924869	Ccdc24	0.175	0.599149386954389	-
6937051	Cgref1	0.175	0.599149386954389	+
6939178	Scfd2	0.175	0.599149386954389	-
6941780	Rhof	0.175	0.599149386954389	-
6982246	Ing2	0.175	0.599149386954389	-
6984071	Snx20	0.175	0.599149386954389	-
6990263	Myo1e	0.175	0.599149386954389	-
6990948	Ttk	0.175	0.599149386954389	-
6995736	Elmod1	0.175	0.599149386954389	-
6751712	Atg4b	0.176	0.599149386954389	-
6804940	AW209491	0.176	0.599149386954389	-
6846468	Col8a1	0.176	0.599149386954389	-
6849950	Adamts10	0.176	0.599149386954389	-
6850077	Slc44a4	0.176	0.599149386954389	-
6851234	Kdm4b	0.176	0.599149386954389	-
6852382	Ccdc75	0.176	0.599149386954389	-
6852389	2410091C18Rik	0.176	0.599149386954389	-
6872290	Pgm5	0.176	0.599149386954389	-
6884155	Slco4a1	0.176	0.599149386954389	-
6913566	Fktn	0.176	0.599149386954389	-
6918725	Masp2	0.176	0.599149386954389	-
6933513	Tfip11	0.176	0.599149386954389	-
6935408	Cyth3	0.176	0.599149386954389	-
6941040	Noc4l	0.176	0.599149386954389	-
6941045	Ep400	0.176	0.599149386954389	-
6955514	Nup210	0.176	0.599149386954389	-
6960403	Car11	0.176	0.599149386954389	-
6964380	Itgam	0.176	0.599149386954389	-
7011933	Zfp92	0.176	0.599149386954389	-
6749115	Gulp1	0.177	0.599149386954389	-
6764841	Mark1	0.177	0.599149386954389	-
6772802	Enpp1	0.177	0.599149386954389	-
6813070	Ptpdc1	0.177	0.599149386954389	-
6822469	Acox2	0.177	0.599149386954389	-
6837785	Mapk11	0.177	0.599149386954389	-
6844306	Dgcr14	0.177	0.599149386954389	-

6857440	Cebpz	0.177	0.599149386954389	-
6861751	D18Ertd653e	0.177	0.599149386954389	-
6867728	Yif1a	0.177	0.599149386954389	-
6878448	Itga4	0.177	0.599149386954389	+
6882521	Map1lc3a	0.177	0.599149386954389	-
6926305	Pla2g5	0.177	0.599149386954389	-
6946313	Creb5	0.177	0.599149386954389	-
6951102	Stk38l	0.177	0.599149386954389	-
6987374	Slc44a2	0.177	0.599149386954389	-
6989366	Sin3a	0.177	0.599149386954389	-
7018955	Rps6ka6	0.177	0.599149386954389	-
6748525	Dst	0.178	0.599149386954389	-
6755729	Cnih4	0.178	0.599149386954389	-
6774934	Tfam	0.178	0.599149386954389	-
6777353	Nup107	0.178	0.599149386954389	-
6789693	Sgsm2	0.178	0.599149386954389	-
6819510	Cab39l	0.178	0.599149386954389	-
6861700	Impa2	0.178	0.599149386954389	-
6876029	Gle1	0.178	0.599149386954389	-
6882305	H13	0.178	0.599149386954389	-
6884262	Rtel1	0.178	0.599149386954389	-
6887660	Tlk1	0.178	0.599149386954389	-
6888011	Sestd1	0.178	0.599149386954389	-
6897864	Mbnl1	0.178	0.599149386954389	-
6924706	Cyp4x1	0.178	0.599149386954389	+
6924963	Ppcs	0.178	0.599149386954389	-
6927231	Tnfrsf14	0.178	0.599149386954389	-
6950070	Foxm1	0.178	0.599149386954389	-
6957435	Klra1	0.178	0.599149386954389	-
6972501	Tpcn2	0.178	0.599149386954389	-
6974639	Plat	0.178	0.599149386954389	+
6978866	Tsnaxip1	0.178	0.599149386954389	-
6981938	Zfp353	0.178	0.599149386954389	-
6763090	Rgl1	0.179	0.599149386954389	-
6771641	Stat2	0.179	0.599149386954389	-
6781138	G3bp1	0.179	0.599149386954389	-
6787469	Hmmr	0.179	0.599149386954389	-
6791686	Map3k14	0.179	0.599149386954389	-
6805578	Prl3c1	0.179	0.599149386954389	-
6824902	Pspc1	0.179	0.599149386954389	-
6835934	Atad2	0.179	0.599149386954389	-
6845992	BC016579	0.179	0.599149386954389	-
6855061	Cfb	0.179	0.599149386954389	-
6873408	Cuedc2	0.179	0.599149386954389	-
6888774	F2	0.179	0.599149386954389	-
6929651	Agbl5	0.179	0.599149386954389	-
6934645	Scand3	0.179	0.599149386954389	-
6942604	Mcm7	0.179	0.599149386954389	-
6945324	Bpgm	0.179	0.599149386954389	-
6962155	Stard5	0.179	0.599149386954389	-

6991383	Plod2	0.179	0.599149386954389	-
6771527	Avil	0.18	0.599149386954389	-
6782277	Camkk1	0.18	0.599149386954389	+
6785865	Igfbp3	0.18	0.599149386954389	-
6792614	Socs3	0.18	0.599149386954389	+
6815382	Rgnef	0.18	0.599149386954389	-
6821298	6720463M24Rik	0.18	0.599149386954389	-
6829667	Pop1	0.18	0.599149386954389	-
6833417	Amhr2	0.18	0.599149386954389	-
6848553	Tagap	0.18	0.599149386954389	-
6863758	Dsc2	0.18	0.599149386954389	-
6867747	Catsper1	0.18	0.599149386954389	-
6880511	Vps18	0.18	0.599149386954389	-
6884750	Itga8	0.18	0.599149386954389	+
6907216	Prpf3	0.18	0.599149386954389	-
6926882	Fbxo44	0.18	0.599149386954389	-
6957185	Cd9	0.18	0.599149386954389	-
6959096	Zfp235	0.18	0.599149386954389	-
6965206	Tspan4	0.18	0.599149386954389	-
6971275	Il27	0.18	0.599149386954389	-
6974039	Arhgef7	0.18	0.599149386954389	+
6979439	Plcg2	0.18	0.599149386954389	-
6982621	Adam29	0.18	0.599149386954389	-
6995691	Ddx10	0.18	0.599149386954389	-
6997528	Snap91	0.18	0.599149386954389	-
7011888	Fate1	0.18	0.599149386954389	-
6751226	Cab39	0.181	0.599149386954389	-
6763810	Pou2f1	0.181	0.599149386954389	-
6778375	Sf3a1	0.181	0.599149386954389	-
6780858	Agxt2l2	0.181	0.599149386954389	-
6790099	5730455P16Rik	0.181	0.599149386954389	-
6812386	F13a1	0.181	0.599149386954389	-
6823284	Kcnma1	0.181	0.599149386954389	-
6833513	Nckap1l	0.181	0.599149386954389	-
6841362	Retnla	0.181	0.599149386954389	-
6851324	Emr1	0.181	0.599149386954389	-
6867653	Clcf1	0.181	0.599149386954389	-
6885287	Mastl	0.181	0.599149386954389	-
6901353	Agxt2l1	0.181	0.599149386954389	-
6905577	E130311K13Rik	0.181	0.599149386954389	-
6906386	Fgb	0.181	0.599149386954389	-
6907289	Polr3gl	0.181	0.599149386954389	-
6916584	Ccdc163	0.181	0.599149386954389	-
6917394	Matn1	0.181	0.599149386954389	-
6922294	Kif12	0.181	0.599149386954389	-
6951401	Pon3	0.181	0.599149386954389	-
6977768	Syce2	0.181	0.599149386954389	-
7014051	Fam199x	0.181	0.599149386954389	-
7023069	Kdm5d	0.181	0.599149386954389	-
6756130	Esrrg	0.182	0.599149386954389	-

6769316	Zfp873	0.182	0.599149386954389	-
6778528	Ykt6	0.182	0.599149386954389	-
6822949	Ube2e1	0.182	0.599149386954389	-
6823690	Itih3	0.182	0.599149386954389	-
6849779	Pde9a	0.182	0.599149386954389	-
6854259	Thoc6	0.182	0.599149386954389	-
6875592	Apbb1ip	0.182	0.599149386954389	-
6884460	Cdc123	0.182	0.599149386954389	-
6889330	Hipk3	0.182	0.599149386954389	-
6920713	Spaca1	0.182	0.599149386954389	-
6930610	Pacrgl	0.182	0.599149386954389	-
6933251	Brdt	0.182	0.599149386954389	-
6939696	Ugt2b1	0.182	0.599149386954389	-
6991368	Plscr4	0.182	0.599149386954389	+
6999447	Gorasp1	0.182	0.599149386954389	-
6772217	Ltv1	0.183	0.599149386954389	-
6790015	Ksr1	0.183	0.599149386954389	-
6807328	Fbxl21	0.183	0.599149386954389	-
6809524	Smn1	0.183	0.599149386954389	-
6822167	Mbnl2	0.183	0.599149386954389	-
6832217	Serhl	0.183	0.599149386954389	-
6849307	Nthl1	0.183	0.599149386954389	-
6855801	Foxp4	0.183	0.599149386954389	-
6871929	Rorb	0.183	0.599149386954389	-
6884302	Pcmtd2	0.183	0.599149386954389	-
6897503	Stoml3	0.183	0.599149386954389	-
6944982	2310016C08Rik	0.183	0.599149386954389	-
6955605	Zfyve20	0.183	0.599149386954389	-
6965087	Mtg1	0.183	0.599149386954389	-
6971129	Zkscan2	0.183	0.599149386954389	-
6980748	Csmd1	0.183	0.599149386954389	-
6993489	Panx1	0.183	0.599149386954389	-
6998692	Klhdc8b	0.183	0.599149386954389	-
6999416	Myd88	0.183	0.599149386954389	-
6771647	Cs	0.184	0.599149386954389	-
6779288	AV249152	0.184	0.599149386954389	-
6786621	Commd1	0.184	0.599149386954389	-
6831984	Tab1	0.184	0.599149386954389	-
6832387	Ppara	0.184	0.599149386954389	-
6843624	Btbd12	0.184	0.599149386954389	-
6851204	Chaf1a	0.184	0.599149386954389	-
6874584	Frmd4a	0.184	0.599149386954389	-
6885773	Crat	0.184	0.599149386954389	-
6892401	Uqcc	0.184	0.599149386954389	-
6935145	A430033K04Rik	0.184	0.599149386954389	+
6937099	Supt7l	0.184	0.599149386954389	-
6941734	Rad9b	0.184	0.599149386954389	-
6979123	Aars	0.184	0.599149386954389	-
6998583	Acy1	0.184	0.599149386954389	-
7010856	Stag2	0.184	0.599149386954389	-

6753397	Tnni1	0.185	0.599149386954389	-
6759732	Tmbim1	0.185	0.599149386954389	-
6764040	Fcgr3	0.185	0.599149386954389	+
6782859	Tmem98	0.185	0.599149386954389	-
6840535	2310010M20Rik	0.185	0.599149386954389	-
6844218	4933404G15Rik	0.185	0.599149386954389	-
6868185	Keg1	0.185	0.599149386954389	-
6883020	Hnf4a	0.185	0.599149386954389	-
6904303	Trpc3	0.185	0.599149386954389	-
6918763	Dffa	0.185	0.599149386954389	-
6918999	Plekhg5	0.185	0.599149386954389	-
6920070	Nkain3	0.185	0.599149386954389	-
6926396	Padi1	0.185	0.599149386954389	-
6931227	Fam114a1	0.185	0.599149386954389	-
6936560	Phtf2	0.185	0.599149386954389	-
6949272	Anubl1	0.185	0.599149386954389	-
6949865	Tnfrsf1a	0.185	0.599149386954389	+
6950718	Pde3a	0.185	0.599149386954389	-
6959639	Abpe	0.185	0.599149386954389	-
6959809	Slc7a9	0.185	0.599149386954389	-
6963854	Tmc5	0.185	0.599149386954389	-
6966498	Dpy19l3	0.185	0.599149386954389	+
6992649	Cmtm6	0.185	0.599149386954389	-
7014173	Vsig1	0.185	0.599149386954389	-
6754690	Selp	0.186	0.599149386954389	-
6757745	Zfp451	0.186	0.599149386954389	+
6763591	Mettl13	0.186	0.599149386954389	-
6769553	BC030307	0.186	0.599149386954389	-
6771569	Stac3	0.186	0.599149386954389	-
6784363	Adam11	0.186	0.599149386954389	-
6789397	Bcl6b	0.186	0.599149386954389	-
6844118	0610037P05Rik	0.186	0.599149386954389	-
6863434	Npc1	0.186	0.599149386954389	-
6868845	Dmrt2	0.186	0.599149386954389	-
6884130	Gtpbp5	0.186	0.599149386954389	-
6890838	Il1b	0.186	0.599149386954389	-
6907292	6330549D23Rik	0.186	0.599149386954389	-
6907623	Ptgfrn	0.186	0.599149386954389	-
6911719	Rad54b	0.186	0.599149386954389	-
6934972	Rasa4	0.186	0.599149386954389	-
6936088	Rundc3b	0.186	0.599149386954389	-
6941887	Rilpl1	0.186	0.599149386954389	-
6951096	Med21	0.186	0.599149386954389	-
6957141	Cd4	0.186	0.599149386954389	-
6960326	Irf3	0.186	0.599149386954389	-
6963006	Pde2a	0.186	0.599149386954389	-
6963898	2610020H08Rik	0.186	0.599149386954389	-
6971602	1700007K09Rik	0.186	0.599149386954389	-
7016826	Gpc3	0.186	0.599149386954389	-
6748553	Arhgef4	0.187	0.599149386954389	+

6752165	Serpinb8	0.187	0.599149386954389	-
6775441	Atcay	0.187	0.599149386954389	-
6791465	Psmc3ip	0.187	0.599149386954389	-
6799246	Atp6v1c2	0.187	0.599149386954389	-
6818742	Peli2	0.187	0.599149386954389	-
6823059	Mrps16	0.187	0.599149386954389	-
6832343	Fam118a	0.187	0.599149386954389	-
6844955	Atp13a4	0.187	0.599149386954389	-
6852882	Epcam	0.187	0.599149386954389	-
6860128	Ik	0.187	0.599149386954389	-
6868386	Prune2	0.187	0.599149386954389	-
6873516	Col17a1	0.187	0.599149386954389	-
6887819	Ola1	0.187	0.599149386954389	-
6890714	1500011K16Rik	0.187	0.599149386954389	-
6893020	Zmynd8	0.187	0.599149386954389	-
6917045	Inpp5b	0.187	0.599149386954389	-
6929849	Sh3bp2	0.187	0.599149386954389	-
6943204	Ubl3	0.187	0.599149386954389	-
6946835	Igk-V28	0.187	0.599149386954389	-
6948906	Itpr1	0.187	0.599149386954389	-
6956902	Cacna1c	0.187	0.599149386954389	-
6963884	Acsm2	0.187	0.599149386954389	-
6754669	Scyl3	0.188	0.599149386954389	-
6757906	4632411B12Rik	0.188	0.599149386954389	-
6760208	Tm4sf20	0.188	0.599149386954389	-
6761692	Steap3	0.188	0.599149386954389	-
6771546	B4galnt1	0.188	0.599149386954389	-
6789544	Spns3	0.188	0.599149386954389	-
6792114	Abca8a	0.188	0.599149386954389	-
6798600	Cenpo	0.188	0.599149386954389	-
6803210	Ifi27l2a	0.188	0.599149386954389	-
6815031	Msh3	0.188	0.599149386954389	-
6823693	Itih1	0.188	0.599149386954389	-
6844567	Dgkg	0.188	0.599149386954389	-
6864509	Pkd2l2	0.188	0.599149386954389	-
6878016	Metap1d	0.188	0.599149386954389	-
6881126	--	0.188	0.599149386954389	-
6909278	Enpep	0.188	0.599149386954389	-
6916119	Hspb11	0.188	0.599149386954389	-
6917032	Pou3f1	0.188	0.599149386954389	-
6925984	Cd52	0.188	0.599149386954389	-
6932540	Ccng2	0.188	0.599149386954389	-
6933625	Oasl2	0.188	0.599149386954389	-
6947396	Rtkn	0.188	0.599149386954389	-
6954418	Il23r	0.188	0.599149386954389	-
6978905	Cdh3	0.188	0.599149386954389	-
6979829	Arv1	0.188	0.599149386954389	-
6981870	Mtmr7	0.188	0.599149386954389	-
6998327	Ephb1	0.188	0.599149386954389	-
6748426	Lgsn	0.189	0.599149386954389	-

6750440	Igfbp2	0.189	0.599149386954389	-
6751349	Dgkd	0.189	0.599149386954389	-
6754325	Angptl1	0.189	0.599149386954389	-
6760194	Col4a4	0.189	0.599149386954389	-
6774794	Cdk1	0.189	0.599149386954389	-
6782273	Atp2a3	0.189	0.599149386954389	-
6788928	Zfp287	0.189	0.599149386954389	-
6791240	Med1	0.189	0.599149386954389	-
6801104	Trappc6b	0.189	0.599149386954389	-
6837252	Mkl1	0.189	0.599149386954389	-
6839952	Psmd2	0.189	0.599149386954389	-
6843629	Trap1	0.189	0.599149386954389	-
6853911	Tcte3	0.189	0.599149386954389	-
6854907	Zfp870	0.189	0.599149386954389	-
6868884	Smarca2	0.189	0.599149386954389	+
6869414	Pcgf5	0.189	0.599149386954389	-
6889301	Nat10	0.189	0.599149386954389	-
6892973	Zfp335	0.189	0.599149386954389	-
6906738	Tmem79	0.189	0.599149386954389	-
6912499	Mdn1	0.189	0.599149386954389	-
6924947	Zfp691	0.189	0.599149386954389	-
6939047	Commd8	0.189	0.599149386954389	-
6964531	Dmbt1	0.189	0.599149386954389	-
6969993	Olfr69	0.189	0.599149386954389	-
6991461	Slc9a9	0.189	0.599149386954389	-
6747504	6030422M02Rik	0.19	0.599149386954389	-
6755853	Capn8	0.19	0.599149386954389	-
6762151	Ikbke	0.19	0.599149386954389	-
6769735	Uhrf1bp1l	0.19	0.599149386954389	-
6774308	Cdh23	0.19	0.599149386954389	-
6775176	A130042E20Rik	0.19	0.599149386954389	-
6788862	Ulk2	0.19	0.599149386954389	-
6833418	Prr13	0.19	0.599149386954389	-
6836782	Pycrl	0.19	0.599149386954389	-
6854378	Tsc2	0.19	0.599149386954389	-
6854537	Grm4	0.19	0.599149386954389	-
6859942	Pkd2l2	0.19	0.599149386954389	-
6871265	Gpr137	0.19	0.599149386954389	-
6871603	Olfr1426	0.19	0.599149386954389	-
6887983	Cyct	0.19	0.599149386954389	-
6890715	Bub1	0.19	0.599149386954389	-
6899213	Gba	0.19	0.599149386954389	-
6937617	Clnk	0.19	0.599149386954389	-
6964396	Slc5a2	0.19	0.599149386954389	-
6965078	Prap1	0.19	0.599149386954389	-
6968041	Synm	0.19	0.599149386954389	-
6976190	Aga	0.19	0.599149386954389	-
6976956	Lpar2	0.19	0.599149386954389	-
6986729	Mmp10	0.19	0.599149386954389	-
6988291	Vwa5a	0.19	0.599149386954389	-

6993431	Cep57	0.19	0.599149386954389	-
6997730	Tbc1d2b	0.19	0.599149386954389	-
6763585	Dnm3	0.191	0.599149386954389	-
6764435	Akt3	0.191	0.599149386954389	-
6768005	Msl3l2	0.191	0.599149386954389	-
6778293	Pla2g3	0.191	0.599149386954389	-
6781969	1500010J02Rik	0.191	0.599149386954389	-
6782092	Dvl2	0.191	0.599149386954389	-
6788375	Lyrm7	0.191	0.599149386954389	-
6806824	Kdm1b	0.191	0.599149386954389	-
6807480	Ntrk2	0.191	0.599149386954389	-
6844632	Masp1	0.191	0.599149386954389	-
6853868	4930474M22Rik	0.191	0.599149386954389	-
6854405	Telo2	0.191	0.599149386954389	-
6871259	Esrra	0.191	0.599149386954389	-
6884820	St8sia6	0.191	0.599149386954389	-
6885437	Sohlh1	0.191	0.599149386954389	-
6895790	Ralyl	0.191	0.599149386954389	-
6909792	Dapp1	0.191	0.599149386954389	-
6913824	Musk	0.191	0.599149386954389	-
6919223	9430015G10Rik	0.191	0.599149386954389	-
6929292	Nos3	0.191	0.599149386954389	-
6963888	Acsm4	0.191	0.599149386954389	-
6965141	Nlrp6	0.191	0.599149386954389	-
6969289	Tmem126a	0.191	0.599149386954389	-
6969651	Acer3	0.191	0.599149386954389	-
6977797	Man2b1	0.191	0.599149386954389	-
6983168	Slc25a42	0.191	0.599149386954389	+
7019994	Trpc5	0.191	0.599149386954389	-
6764010	Ddr2	0.192	0.599149386954389	-
6769509	Pwp1	0.192	0.599149386954389	-
6791174	Tbkbp1	0.192	0.599149386954389	-
6791569	Fam171a2	0.192	0.599149386954389	-
6792606	Tha1	0.192	0.599149386954389	-
6825273	Ints6	0.192	0.599149386954389	+
6860436	Tcerg1	0.192	0.599149386954389	-
6880629	Adal	0.192	0.599149386954389	-
6899152	Rit1	0.192	0.599149386954389	-
6900713	Sass6	0.192	0.599149386954389	-
6910592	Ifi44	0.192	0.599149386954389	-
6916615	Ptch2	0.192	0.599149386954389	-
6923284	Elavl2	0.192	0.599149386954389	-
6929152	Psmc2	0.192	0.599149386954389	-
6986031	Acta1	0.192	0.599149386954389	-
6999502	Ulk4	0.192	0.599149386954389	-
6761155	Pign	0.193	0.599149386954389	-
6768693	Ipmk	0.193	0.599149386954389	+
6778463	Xbp1	0.193	0.599149386954389	-
6781988	Aloxe3	0.193	0.599149386954389	-
6785294	Tmc8	0.193	0.599149386954389	-

6804134	Dnahc11	0.193	0.599149386954389	-
6804539	Akr1c21	0.193	0.599149386954389	-
6806073	Nqo2	0.193	0.599149386954389	-
6812313	Rpp40	0.193	0.599149386954389	-
6825132	Atp8a2	0.193	0.599149386954389	-
6833151	Spats2	0.193	0.599149386954389	-
6866004	Afap1l1	0.193	0.599149386954389	-
6867837	Naaladl1	0.193	0.599149386954389	-
6870069	Sfxn2	0.193	0.599149386954389	-
6871102	Sart1	0.193	0.599149386954389	-
6892230	8430427H17Rik	0.193	0.599149386954389	-
6911805	Gm11818	0.193	0.599149386954389	-
6939830	Gc	0.193	0.599149386954389	-
6944754	Spam1	0.193	0.599149386954389	-
6965670	Hif3a	0.193	0.599149386954389	-
6971307	Tmem219	0.193	0.599149386954389	-
6981103	Adam3	0.193	0.599149386954389	-
6989258	AI118078	0.193	0.599149386954389	-
6989914	Igdcc3	0.193	0.599149386954389	-
6750625	Des	0.194	0.599149386954389	-
6751362	Ugt1a10	0.194	0.599149386954389	-
6754143	Rnasel	0.194	0.599149386954389	-
6767845	Nus1	0.194	0.599149386954389	-
6779196	Rab1	0.194	0.599149386954389	-
6783297	Trim37	0.194	0.599149386954389	-
6807172	Arl10	0.194	0.599149386954389	+
6811664	Btn2a2	0.194	0.599149386954389	-
6825205	Sgcg	0.194	0.599149386954389	-
6832848	Irak4	0.194	0.599149386954389	-
6840694	Mylk	0.194	0.599149386954389	-
6848688	Agpat4	0.194	0.599149386954389	-
6854031	Lnpep	0.194	0.599149386954389	-
6855018	H2-Aa	0.194	0.599149386954389	-
6867642	Tmem134	0.194	0.599149386954389	-
6873360	Poll	0.194	0.599149386954389	-
6878984	Fnbp4	0.194	0.599149386954389	-
6879484	Rag2	0.194	0.599149386954389	-
6885522	Fam163b	0.194	0.599149386954389	+
6888830	Gyltl1b	0.194	0.599149386954389	-
6900450	Slc25a24	0.194	0.599149386954389	-
6902665	4922501L14Rik	0.194	0.599149386954389	-
6916708	Med8	0.194	0.599149386954389	-
6925369	Eif2c4	0.194	0.599149386954389	-
6937590	Zbtb49	0.194	0.599149386954389	-
6937813	Prom1	0.194	0.599149386954389	-
6976991	Gdf1	0.194	0.599149386954389	-
6983163	Rfxank	0.194	0.599149386954389	-
6985981	Chmp1a	0.194	0.599149386954389	-
7013375	Dach2	0.194	0.599149386954389	-
6761915	Nckap5	0.195	0.599149386954389	-

6778047	Ikzf4	0.195	0.599149386954389	-
6790231	Nle1	0.195	0.599149386954389	-
6803770	Stk30	0.195	0.599149386954389	-
6813946	Gm10324	0.195	0.599149386954389	-
6823709	Stab1	0.195	0.599149386954389	-
6836779	Eef1d	0.195	0.599149386954389	-
6837594	2210021J22Rik	0.195	0.599149386954389	-
6847517	Mrpl39	0.195	0.599149386954389	-
6873235	Cox15	0.195	0.599149386954389	-
6878035	Pdk1	0.195	0.599149386954389	-
6880084	Tmco5b	0.195	0.599149386954389	-
6889752	Nut	0.195	0.599149386954389	-
6890258	Rpap1	0.195	0.599149386954389	-
6900086	Sike1	0.195	0.599149386954389	-
6915559	Fggy	0.195	0.599149386954389	-
6918040	Akr7a5	0.195	0.599149386954389	-
6925254	Cdca8	0.195	0.599149386954389	-
6931823	Cep135	0.195	0.599149386954389	-
6932487	Art3	0.195	0.599149386954389	-
6940358	Plac8	0.195	0.599149386954389	-
6942458	Tmem120a	0.195	0.599149386954389	-
6945967	Zfp783	0.195	0.599149386954389	-
6946904	Krcc1	0.195	0.599149386954389	-
6947176	Reg1	0.195	0.599149386954389	-
6949844	Acrbp	0.195	0.599149386954389	-
6957744	Plbd1	0.195	0.599149386954389	-
6959540	Zfp420	0.195	0.599149386954389	-
6963558	Arntl	0.195	0.599149386954389	-
6974153	Tmco3	0.195	0.599149386954389	-
6983878	Rad23a	0.195	0.599149386954389	-
7020154	Tro	0.195	0.599149386954389	-
6758361	Kdelc1	0.196	0.599149386954389	-
6760754	Kif1a	0.196	0.599149386954389	-
6761701	Marco	0.196	0.599149386954389	-
6786849	Vrk2	0.196	0.599149386954389	-
6791493	Aarsd1	0.196	0.599149386954389	-
6791494	Rundc1	0.196	0.599149386954389	-
6806271	Rreb1	0.196	0.599149386954389	-
6812973	Kif13a	0.196	0.599149386954389	-
6813253	Sptlc1	0.196	0.599149386954389	-
6819032	E130112L23Rik	0.196	0.599149386954389	-
6839699	Top3b	0.196	0.599149386954389	-
6841348	Morc1	0.196	0.599149386954389	-
6849383	Lmf1	0.196	0.599149386954389	-
6851292	Alkbh7	0.196	0.599149386954389	-
6875578	Myo3a	0.196	0.599149386954389	-
6876740	Arhgap15	0.196	0.599149386954389	-
6884296	Myt1	0.196	0.599149386954389	-
6910133	Clca6	0.196	0.599149386954389	-
6919407	Nsmaf	0.196	0.599149386954389	-

6935118	Cnpy4	0.196	0.599149386954389	-
6939931	Btc	0.196	0.599149386954389	-
6950688	Aebp2	0.196	0.599149386954389	-
6957162	Ncapd2	0.196	0.599149386954389	-
6966431	Lsm14a	0.196	0.599149386954389	-
6971446	Tial1	0.196	0.599149386954389	-
6992492	Trank1	0.196	0.599149386954389	-
6995534	Sik2	0.196	0.599149386954389	+
6995619	Fdx1	0.196	0.599149386954389	-
6998930	Mlh1	0.196	0.599149386954389	-
7017672	F8	0.196	0.599149386954389	-
6770148	Poc1b	0.197	0.599149386954389	-
6774391	Hk1	0.197	0.599149386954389	-
6807609	2010111I01Rik	0.197	0.599149386954389	-
6850250	Trim26	0.197	0.599149386954389	-
6861275	Adamts19	0.197	0.599149386954389	-
6871056	Ccs	0.197	0.599149386954389	-
6884173	Ntsr1	0.197	0.599149386954389	-
6885871	Dnm1	0.197	0.599149386954389	-
6902148	Odf2l	0.197	0.599149386954389	-
6932853	Cops4	0.197	0.599149386954389	-
6942546	Slc12a9	0.197	0.599149386954389	-
6942943	Ocm	0.197	0.599149386954389	-
6944581	Wnt16	0.197	0.599149386954389	-
6949106	Irak2	0.197	0.599149386954389	-
6953069	1700074P13Rik	0.197	0.599149386954389	-
6964012	Scnn1b	0.197	0.599149386954389	-
6971332	Cd2bp2	0.197	0.599149386954389	-
6981168	Letm2	0.197	0.599149386954389	-
6983604	Mmaa	0.197	0.599149386954389	-
6990768	Senp6	0.197	0.599149386954389	-
7011937	Atp2b3	0.197	0.599149386954389	-
6759642	Mreg	0.198	0.599149386954389	-
6780696	Trim7	0.198	0.599149386954389	-
6784997	D11Wsu47e	0.198	0.599149386954389	-
6791408	Krt14	0.198	0.599149386954389	-
6803596	Slc25a29	0.198	0.599149386954389	-
6813730	BC051665	0.198	0.599149386954389	-
6826205	AU021034	0.198	0.599149386954389	-
6863571	Ss18	0.198	0.599149386954389	-
6882397	Dnmt3b	0.198	0.599149386954389	-
6916511	Mknk1	0.198	0.599149386954389	-
6920816	Lingo2	0.198	0.599149386954389	+
6926908	Ubiad1	0.198	0.599149386954389	-
6949230	Cand2	0.198	0.599149386954389	-
6957137	Gnb3	0.198	0.599149386954389	-
6974743	Fgfr1	0.198	0.599149386954389	-
6978811	D230025D16Rik	0.198	0.599149386954389	-
6983744	Il15	0.198	0.599149386954389	-
6985901	Car5a	0.198	0.599149386954389	-

6989556	Parp6	0.198	0.599149386954389	-
6993109	Clec3b	0.198	0.599149386954389	-
7019923	Ammecr1	0.198	0.599149386954389	-
6754777	Adcy10	0.199	0.599149386954389	-
6766579	Vnn1	0.199	0.599149386954389	-
6769447	Polr3b	0.199	0.599149386954389	-
6775741	D10Wsu52e	0.199	0.599149386954389	-
6777594	Tbk1	0.199	0.599149386954389	-
6778972	Egfr	0.199	0.599149386954389	-
6787585	Pttg1	0.199	0.599149386954389	-
6796728	Esrrb	0.199	0.599149386954389	-
6815305	Hmgcr	0.199	0.599149386954389	+
6817060	Il3ra	0.199	0.599149386954389	-
6820013	Stc1	0.199	0.599149386954389	-
6850017	H2-DMb2	0.199	0.599149386954389	-
6850155	H2-gs10	0.199	0.599149386954389	-
6855400	Cd2ap	0.199	0.599149386954389	-
6858694	Wac	0.199	0.599149386954389	-
6876047	Endog	0.199	0.599149386954389	-
6899029	Mrpl24	0.199	0.599149386954389	-
6917365	Col16a1	0.199	0.599149386954389	-
6924235	Bsnd	0.199	0.599149386954389	-
6925983	Lin28a	0.199	0.599149386954389	-
6926928	Pex14	0.199	0.599149386954389	-
6929550	Rnf32	0.199	0.599149386954389	+
6943142	Flt3	0.199	0.599149386954389	-
6947460	Nagk	0.199	0.599149386954389	-
6954553	Thnsl2	0.199	0.599149386954389	-
6961982	Vps33b	0.199	0.599149386954389	-
6965593	Sepw1	0.199	0.599149386954389	-
6974490	Agpat5	0.199	0.599149386954389	-
6985106	Nqo1	0.199	0.599149386954389	-
6993859	Ecsit	0.199	0.599149386954389	-
6995846	Lingo1	0.199	0.599149386954389	+
6778008	Il23a	0.2	0.599149386954389	-
6782515	Timm22	0.2	0.599149386954389	-
6796208	Churc1	0.2	0.599149386954389	-
6798312	Mta1	0.2	0.599149386954389	-
6806958	Aspn	0.2	0.599149386954389	-
6817053	Atxn7	0.2	0.599149386954389	+
6824740	Haus4	0.2	0.599149386954389	-
6837819	Chkb	0.2	0.599149386954389	-
6845459	Stxbp5l	0.2	0.599149386954389	-
6851193	Shd	0.2	0.599149386954389	-
6855076	Csnk2b	0.2	0.599149386954389	-
6869503	Kif11	0.2	0.599149386954389	-
6870128	Taf5	0.2	0.599149386954389	-
6870745	Pnliprp1	0.2	0.599149386954389	-
6883121	Pcif1	0.2	0.599149386954389	-
6885836	Fibcd1	0.2	0.599149386954389	-

6897009	Larp1b	0.2	0.599149386954389	-
6924365	Podn	0.2	0.599149386954389	-
6925878	Sytl1	0.2	0.599149386954389	-
6940872	Gfi1	0.2	0.599149386954389	-
6949509	Rad52	0.2	0.599149386954389	-
6750087	Pth2r	0.201	0.599149386954389	-
6768114	Ankrd57	0.201	0.599149386954389	-
6779787	Psme4	0.201	0.599149386954389	+
6780572	Timd4	0.201	0.599149386954389	-
6781437	Lrrc48	0.201	0.599149386954389	-
6784036	Gsdma2	0.201	0.599149386954389	-
6789597	Aspa	0.201	0.599149386954389	-
6811068	Lgals8	0.201	0.599149386954389	-
6854273	Flywch1	0.201	0.599149386954389	-
6921994	Svep1	0.201	0.599149386954389	-
6947131	Lrrtm1	0.201	0.599149386954389	+
6973688	2310057J16Rik	0.201	0.599149386954389	-
6989355	Snupn	0.201	0.599149386954389	-
6992866	Vill	0.201	0.599149386954389	-
6993878	Zfp810	0.201	0.599149386954389	-
6995898	Ppcdc	0.201	0.599149386954389	-
6999673	Slc6a20b	0.201	0.599149386954389	-
6999682	Fyco1	0.201	0.599149386954389	-
7018220	Pola1	0.201	0.599149386954389	-
6754696	Slc19a2	0.202	0.599149386954389	-
6759778	Abcb6	0.202	0.599149386954389	-
6762546	Nr5a2	0.202	0.599149386954389	-
6767797	Rfx6	0.202	0.599149386954389	-
6783007	Slfn3	0.202	0.599149386954389	-
6792296	Slc39a11	0.202	0.599149386954389	-
6812635	Mak	0.202	0.599149386954389	-
6825086	Gm5142	0.202	0.599149386954389	-
6843433	Ets2	0.202	0.599149386954389	+
6851848	Lama1	0.202	0.599149386954389	-
6857526	Dhx57	0.202	0.599149386954389	-
6867701	Ctsf	0.202	0.599149386954389	-
6871091	Sf3b2	0.202	0.599149386954389	-
6877988	Dcaf17	0.202	0.599149386954389	-
6882069	Cst13	0.202	0.599149386954389	-
6882437	Plunc	0.202	0.599149386954389	-
6890986	Spef1	0.202	0.599149386954389	-
6924784	Mast2	0.202	0.599149386954389	-
6933563	Wscd2	0.202	0.599149386954389	-
6942875	Rbak	0.202	0.599149386954389	-
6960190	Klk4	0.202	0.599149386954389	-
6963471	Ctr9	0.202	0.599149386954389	-
6965901	Atp1a3	0.202	0.599149386954389	-
6978960	Nfat5	0.202	0.599149386954389	-
6984372	Ces1f	0.202	0.599149386954389	-
6985253	Dhodh	0.202	0.599149386954389	-

6995177	Tmprss4	0.202	0.599149386954389	-
7015229	Arhgap6	0.202	0.599149386954389	-
6760847	St8sia4	0.203	0.599149386954389	-
6768891	Derl3	0.203	0.599149386954389	-
6783264	Ptrh2	0.203	0.599149386954389	-
6789406	Rnasek	0.203	0.599149386954389	-
6815433	Fcho2	0.203	0.599149386954389	-
6818017	Eaf1	0.203	0.599149386954389	-
6828403	C6	0.203	0.599149386954389	-
6843643	Tcfap4	0.203	0.599149386954389	-
6850025	Notch4	0.203	0.599149386954389	-
6852767	Ppm1b	0.203	0.599149386954389	-
6870429	Mxi1	0.203	0.599149386954389	-
6875626	Il1f8	0.203	0.599149386954389	-
6901592	Cenpe	0.203	0.599149386954389	-
6917650	Pafah2	0.203	0.599149386954389	-
6945078	Nrf1	0.203	0.599149386954389	-
6951118	Ppfibp1	0.203	0.599149386954389	-
6977975	Adcy7	0.203	0.599149386954389	-
6979903	2310079N02Rik	0.203	0.599149386954389	-
6980075	Pcp2	0.203	0.599149386954389	-
6983750	Zfp330	0.203	0.599149386954389	-
6991089	Dopey1	0.203	0.599149386954389	-
7019499	Xkrx	0.203	0.599149386954389	-
6752947	Thsd7b	0.204	0.599149386954389	-
6760642	Col6a3	0.204	0.599149386954389	-
6771538	Cdk4	0.204	0.599149386954389	-
6787019	Erlec1	0.204	0.599149386954389	-
6817381	Plau	0.204	0.599149386954389	-
6819573	Mipep	0.204	0.599149386954389	-
6823470	Appl1	0.204	0.599149386954389	-
6852031	Myom1	0.204	0.599149386954389	-
6853467	Serac1	0.204	0.599149386954389	-
6876053	Nup188	0.204	0.599149386954389	-
6876405	Dab2ip	0.204	0.599149386954389	-
6917263	Trim62	0.204	0.599149386954389	-
6918661	Gm13154	0.204	0.599149386954389	-
6930067	Stx18	0.204	0.599149386954389	-
6933616	Ankrd13a	0.204	0.599149386954389	-
6948033	4930590J08Rik	0.204	0.599149386954389	-
6965134	Psmd13	0.204	0.599149386954389	-
6751398	Trpm8	0.205	0.599149386954389	-
6757905	Lman2l	0.205	0.599149386954389	-
6757979	2010300C02Rik	0.205	0.599149386954389	-
6758036	Creg2	0.205	0.599149386954389	-
6763524	Klhl20	0.205	0.599149386954389	-
6768155	Psap	0.205	0.599149386954389	-
6781830	Tmem220	0.205	0.599149386954389	-
6784760	Apoh	0.205	0.599149386954389	-
6789359	Acap1	0.205	0.599149386954389	-

6798403	Ncapg2	0.205	0.599149386954389	-
6799651	Ttc15	0.205	0.599149386954389	-
6808609	Mef2c	0.205	0.599149386954389	-
6812679	9530008L14Rik	0.205	0.599149386954389	-
6815291	Polk	0.205	0.599149386954389	-
6832538	Panx2	0.205	0.599149386954389	-
6844329	Dgcr8	0.205	0.599149386954389	-
6852222	Birc6	0.205	0.599149386954389	-
6864330	Slc25a46	0.205	0.599149386954389	-
6872888	Cpeb3	0.205	0.599149386954389	+
6883111	Spint4	0.205	0.599149386954389	-
6906894	Ints3	0.205	0.599149386954389	-
6915064	Cntln	0.205	0.599149386954389	-
6915253	Mtap	0.205	0.599149386954389	-
6921161	Tpm2	0.205	0.599149386954389	-
6934907	Baz1b	0.205	0.599149386954389	-
6944397	Cftr	0.205	0.599149386954389	-
6958030	Slco1a1	0.205	0.599149386954389	-
6967983	Asb7	0.205	0.599149386954389	-
6969888	Folr1	0.205	0.599149386954389	-
6979559	Irf8	0.205	0.599149386954389	-
6981177	Ash2l	0.205	0.599149386954389	-
6983698	Usp38	0.205	0.599149386954389	+
6987891	Prdm10	0.205	0.599149386954389	-
6995149	Ube4a	0.205	0.599149386954389	-
7009892	Lancl3	0.205	0.599149386954389	-
7012845	Dlg3	0.205	0.599149386954389	-
6749742	Stradb	0.206	0.599149386954389	-
6754476	Mrps14	0.206	0.599149386954389	-
6765275	Traf5	0.206	0.599149386954389	-
6772111	Grm1	0.206	0.599149386954389	-
6787197	Kcnip1	0.206	0.599149386954389	-
6791063	Abi3	0.206	0.599149386954389	-
6823149	Ap3m1	0.206	0.599149386954389	-
6824346	Dlgap5	0.206	0.599149386954389	-
6829281	Fam134b	0.206	0.599149386954389	-
6840278	Ostn	0.206	0.599149386954389	-
6845957	Gtpbp8	0.206	0.599149386954389	-
6870734	1700011F14Rik	0.206	0.599149386954389	-
6887671	Mettl8	0.206	0.599149386954389	+
6913028	5430416O09Rik	0.206	0.599149386954389	-
6913194	Tdrd7	0.206	0.599149386954389	-
6918690	2510039O18Rik	0.206	0.599149386954389	-
6934132	Anapc7	0.206	0.599149386954389	-
6955169	1700019G17Rik	0.206	0.599149386954389	+
6955305	H1fx	0.206	0.599149386954389	-
6958733	Nlrp4b	0.206	0.599149386954389	-
6963623	Calcb	0.206	0.599149386954389	-
6973477	Oscar	0.206	0.599149386954389	+
6978695	Cdh5	0.206	0.599149386954389	-

6983840	Ccdc130	0.206	0.599149386954389	-
6993296	Birc3	0.206	0.599149386954389	-
6758633	Mfsd6	0.207	0.599149386954389	-
6781368	Nlrp3	0.207	0.599149386954389	-
6789270	Cntrob	0.207	0.599149386954389	-
6807564	Dapk1	0.207	0.599149386954389	-
6822191	Ipo5	0.207	0.599149386954389	-
6843601	Mefv	0.207	0.599149386954389	-
6861688	Gnal	0.207	0.599149386954389	-
6875695	Nrarp	0.207	0.599149386954389	-
6880466	Phgr1	0.207	0.599149386954389	-
6899326	Rab13	0.207	0.599149386954389	-
6899660	Cdc42se1	0.207	0.599149386954389	-
6913128	Grhpr	0.207	0.599149386954389	+
6917073	Snip1	0.207	0.599149386954389	-
6925776	Epb4.1	0.207	0.599149386954389	-
6939076	Txk	0.207	0.599149386954389	-
6947894	Txnrd3	0.207	0.599149386954389	-
6951740	Vwde	0.207	0.599149386954389	-
6958974	Pglyrp1	0.207	0.599149386954389	+
6965668	Ppp5c	0.207	0.599149386954389	-
6965853	Zfp94	0.207	0.599149386954389	-
6984973	Zdhhc1	0.207	0.599149386954389	-
6994589	Fam118b	0.207	0.599149386954389	-
7019542	Nxf2	0.207	0.599149386954389	-
7020027	Amot	0.207	0.599149386954389	-
6753091	Elk4	0.208	0.599149386954389	-
6771658	Rnf41	0.208	0.599149386954389	-
6789051	Myocd	0.208	0.599149386954389	-
6847765	Srsf15	0.208	0.599149386954389	-
6855759	Taf8	0.208	0.599149386954389	-
6870182	Gsto2	0.208	0.599149386954389	-
6899626	Rfx5	0.208	0.599149386954389	-
6907155	Prune	0.208	0.599149386954389	-
6929735	Yes1	0.208	0.599149386954389	-
6960835	Tubgcp5	0.208	0.599149386954389	-
6964011	Scnn1g	0.208	0.599149386954389	-
6969947	Trim68	0.208	0.599149386954389	-
6977177	Sin3b	0.208	0.599149386954389	-
6986602	Kbtbd3	0.208	0.599149386954389	-
7011052	Rbmx2	0.208	0.599149386954389	-
6749529	Ccdc150	0.209	0.599149386954389	-
6749701	Sgol2	0.209	0.599149386954389	-
6751525	Lrrfip1	0.209	0.599149386954389	-
6758662	Pms1	0.209	0.599149386954389	-
6780694	Gnb2l1	0.209	0.599149386954389	-
6797484	Golga5	0.209	0.599149386954389	-
6818157	Rbp3	0.209	0.599149386954389	-
6833404	Espl1	0.209	0.599149386954389	-
6840004	Vps8	0.209	0.599149386954389	-

6854680	Cpne5	0.209	0.599149386954389	-
6863274	Gjd4	0.209	0.599149386954389	-
6868018	Fads3	0.209	0.599149386954389	-
6870615	9930023K05Rik	0.209	0.599149386954389	-
6887715	Dlx2	0.209	0.599149386954389	-
6935756	Brca2	0.209	0.599149386954389	-
6942496	Cux1	0.209	0.599149386954389	-
6959538	Zfp790	0.209	0.599149386954389	-
6973221	Ppp6r1	0.209	0.599149386954389	-
6976901	Lpl	0.209	0.599149386954389	-
6977153	1700030K09Rik	0.209	0.599149386954389	-
6768207	Prf1	0.21	0.599149386954389	-
6772193	Utrn	0.21	0.599149386954389	-
6783785	Gngt2	0.21	0.599149386954389	-
6795069	Arhgap5	0.21	0.599149386954389	-
6807201	Unc5a	0.21	0.599149386954389	-
6811906	Prl8a1	0.21	0.599149386954389	-
6814044	Zfp874a	0.21	0.599149386954389	-
6815255	Aggf1	0.21	0.599149386954389	-
6817930	Tkt	0.21	0.599149386954389	-
6825441	Gulo	0.21	0.599149386954389	-
6831654	Brp16	0.21	0.599149386954389	-
6832573	Ncaph2	0.21	0.599149386954389	-
6836237	Asap1	0.21	0.599149386954389	-
6846197	Bbx	0.21	0.599149386954389	-
6870025	Pprc1	0.21	0.599149386954389	-
6873083	Tctn3	0.21	0.599149386954389	-
6890645	Trpm7	0.21	0.599149386954389	-
6898996	Fcrl1	0.21	0.599149386954389	-
6989054	Pou2af1	0.21	0.599149386954389	-
6989423	Edc3	0.21	0.599149386954389	-
6995521	Dixdc1	0.21	0.599149386954389	-
7015011	Piga	0.21	0.599149386954389	-
6747972	Il17a	0.211	0.599149386954389	-
6759617	Abca12	0.211	0.599149386954389	-
6759658	Ankar	0.211	0.599149386954389	-
6762355	Tmem183a	0.211	0.599149386954389	-
6768851	Specc1l	0.211	0.599149386954389	-
6769895	Usp44	0.211	0.599149386954389	-
6772875	L3mbtl3	0.211	0.599149386954389	+
6774238	Oit3	0.211	0.599149386954389	-
6775977	Apaf1	0.211	0.599149386954389	-
6780996	Irf1	0.211	0.599149386954389	-
6782668	BC017647	0.211	0.599149386954389	-
6788380	Tnip1	0.211	0.599149386954389	-
6796563	Acot1	0.211	0.599149386954389	-
6813744	Tpbpa	0.211	0.599149386954389	-
6823635	Cacna2d3	0.211	0.599149386954389	-
6827203	Rnf219	0.211	0.599149386954389	-
6833980	Rai14	0.211	0.599149386954389	-

6839052	Glis2	0.211	0.599149386954389	-
6839737	Serpind1	0.211	0.599149386954389	-
6843179	Ifnar1	0.211	0.599149386954389	-
6843943	Zc3h7a	0.211	0.599149386954389	-
6873167	Morn4	0.211	0.599149386954389	-
6876175	Pomt1	0.211	0.599149386954389	-
6891322	Mkks	0.211	0.599149386954389	-
6918168	Zbtb17	0.211	0.599149386954389	-
6939681	Tmprss11e	0.211	0.599149386954389	-
6945111	Cpa4	0.211	0.599149386954389	-
6964365	Stx4a	0.211	0.599149386954389	+
6966926	Prr12	0.211	0.599149386954389	-
6977523	Anapc10	0.211	0.599149386954389	-
7010330	Rgn	0.211	0.599149386954389	-
7014545	Phf8	0.211	0.599149386954389	-
6762395	Ptprv	0.212	0.599149386954389	-
6779857	Fbxw11	0.212	0.599149386954389	-
6792314	Sdk2	0.212	0.599149386954389	+
6813563	Kif27	0.212	0.599149386954389	-
6838367	Zfp641	0.212	0.599149386954389	-
6839703	Ppm1f	0.212	0.599149386954389	-
6850114	Bat5	0.212	0.599149386954389	-
6857441	Prkd3	0.212	0.599149386954389	-
6860252	0610009O20Rik	0.212	0.599149386954389	-
6884709	Pfkfb3	0.212	0.599149386954389	+
6890636	Hdc	0.212	0.599149386954389	-
6917048	Yrdc	0.212	0.599149386954389	-
6935399	Grid2ip	0.212	0.599149386954389	-
6947347	Tacr1	0.212	0.599149386954389	-
6959453	Rinl	0.212	0.599149386954389	-
6974220	Dlgap2	0.212	0.599149386954389	+
6994623	Ddx25	0.212	0.599149386954389	-
6994942	Tbcel	0.212	0.599149386954389	-
7012769	Stard8	0.212	0.599149386954389	-
6755233	Igsf8	0.213	0.599149386954389	-
6764118	Vangl2	0.213	0.599149386954389	-
6766705	Epb4.1l2	0.213	0.599149386954389	-
6770718	Nap1l1	0.213	0.599149386954389	-
6775206	Adarb1	0.213	0.599149386954389	-
6792496	Trim65	0.213	0.599149386954389	-
6813585	Slc28a3	0.213	0.599149386954389	-
6819311	Parp4	0.213	0.599149386954389	-
6825678	2610301G19Rik	0.213	0.599149386954389	-
6839552	Nde1	0.213	0.599149386954389	-
6840677	Ccdc14	0.213	0.599149386954389	-
6874947	Itih5	0.213	0.599149386954389	-
6876171	Ppapdc3	0.213	0.599149386954389	-
6880553	Mapkbp1	0.213	0.599149386954389	-
6899760	Txnip	0.213	0.599149386954389	-
6916143	Lrp8	0.213	0.599149386954389	-

6946102	D330028D13Rik	0.213	0.599149386954389	-
6947717	--	0.213	0.599149386954389	-
6955137	Sfxn5	0.213	0.599149386954389	-
6972411	Tnfrsf26	0.213	0.599149386954389	-
6974081	Gm5607	0.213	0.599149386954389	-
6978968	Wwp2	0.213	0.599149386954389	-
6996267	Zwilch	0.213	0.599149386954389	-
7019785	Morc4	0.213	0.599149386954389	-
7019818	Tsc22d3	0.213	0.599149386954389	-
6751914	Cntnap5b	0.214	0.599149386954389	-
6753110	Nuak2	0.214	0.599149386954389	-
6758367	4832428D23Rik	0.214	0.599149386954389	-
6771578	Tmem194	0.214	0.599149386954389	-
6782034	Sat2	0.214	0.599149386954389	+
6784052	Wipf2	0.214	0.599149386954389	-
6785236	1810032O08Rik	0.214	0.599149386954389	-
6792500	Acox1	0.214	0.599149386954389	-
6815523	Naip5	0.214	0.599149386954389	-
6815708	Ppwd1	0.214	0.599149386954389	+
6824880	Gzmb	0.214	0.599149386954389	-
6850058	Ager	0.214	0.599149386954389	-
6869436	Hectd2	0.214	0.599149386954389	+
6881118	Chchd5	0.214	0.599149386954389	-
6885749	D2Wsu81e	0.214	0.599149386954389	-
6919693	Esrp1	0.214	0.599149386954389	-
6941937	Dhx37	0.214	0.599149386954389	-
6944128	Phf14	0.214	0.599149386954389	-
6969255	Eed	0.214	0.599149386954389	-
6973624	Trim28	0.214	0.599149386954389	-
6979993	Pard3	0.214	0.599149386954389	-
6985946	Cbfa2t3	0.214	0.599149386954389	-
6992339	BC048562	0.214	0.599149386954389	-
6998667	Rnf123	0.214	0.599149386954389	-
7006555	Disp1	0.214	0.599149386954389	-
7011904	Gabrq	0.214	0.599149386954389	-
7012536	Arx	0.214	0.599149386954389	-
6758588	Obfc2a	0.215	0.599149386954389	-
6765519	Fbxo5	0.215	0.599149386954389	-
6767844	Nepn	0.215	0.599149386954389	-
6775185	Col6a1	0.215	0.599149386954389	-
6817940	Rft1	0.215	0.599149386954389	-
6820752	Pcdh17	0.215	0.599149386954389	-
6823513	Ccdc66	0.215	0.599149386954389	-
6840508	Mfi2	0.215	0.599149386954389	-
6843928	Txndc11	0.215	0.599149386954389	+
6844359	Cdc45	0.215	0.599149386954389	-
6855707	Ptk7	0.215	0.599149386954389	-
6856077	Efhb	0.215	0.599149386954389	-
6858892	Riok3	0.215	0.599149386954389	-
6867637	Cdk2ap2	0.215	0.599149386954389	-

6869786	Dhdpsl	0.215	0.599149386954389	-
6873407	Psd	0.215	0.599149386954389	-
6875556	Thnsl1	0.215	0.599149386954389	-
6876055	Fam73b	0.215	0.599149386954389	-
6884348	Meig1	0.215	0.599149386954389	-
6913223	Nans	0.215	0.599149386954389	-
6916382	Skint2	0.215	0.599149386954389	-
6929517	En2	0.215	0.599149386954389	-
6940878	Fam69a	0.215	0.599149386954389	-
6942636	Zfp68	0.215	0.599149386954389	-
6944013	Mios	0.215	0.599149386954389	-
6946407	Ghrhr	0.215	0.599149386954389	-
6965919	Pou2f2	0.215	0.599149386954389	-
6966918	Ap2a1	0.215	0.599149386954389	-
6977772	Klf1	0.215	0.599149386954389	-
6982924	Klhl2	0.215	0.599149386954389	-
6989015	Il18	0.215	0.599149386954389	-
6750546	Slc11a1	0.216	0.599149386954389	-
6762413	Rnpep	0.216	0.599149386954389	-
6764708	Fbxo28	0.216	0.599149386954389	-
6775160	Mmp11	0.216	0.599149386954389	-
6785558	Metrnl	0.216	0.599149386954389	-
6789930	Eral1	0.216	0.599149386954389	-
6790291	Ccl9	0.216	0.599149386954389	-
6792942	Dtnb	0.216	0.599149386954389	-
6807518	Naa35	0.216	0.599149386954389	-
6811762	Gmnn	0.216	0.599149386954389	-
6815259	Crhbp	0.216	0.599149386954389	+
6819425	Mrp63	0.216	0.599149386954389	-
6820047	Tnfrsf10b	0.216	0.599149386954389	-
6826016	Cog3	0.216	0.599149386954389	-
6833305	Acvrl1	0.216	0.599149386954389	-
6844952	Atp13a5	0.216	0.599149386954389	-
6854449	Fam195a	0.216	0.599149386954389	-
6861322	Rbm22	0.216	0.599149386954389	-
6897844	Aadac	0.216	0.599149386954389	-
6913390	Zfp189	0.216	0.599149386954389	-
6917055	Epha10	0.216	0.599149386954389	+
6917250	A3galt2	0.216	0.599149386954389	-
6925958	Rps6ka1	0.216	0.599149386954389	-
6939679	Tmprss11bnl	0.216	0.599149386954389	-
6940300	Prkg2	0.216	0.599149386954389	-
6947760	Rpn1	0.216	0.599149386954389	-
6953098	Trpv6	0.216	0.599149386954389	-
6959586	Nphs1	0.216	0.599149386954389	-
6964527	Htra1	0.216	0.599149386954389	-
6978892	Zfp90	0.216	0.599149386954389	-
6978932	Has3	0.216	0.599149386954389	-
6993713	Col5a3	0.216	0.599149386954389	-
6751253	Spata3	0.217	0.599149386954389	-

6765474	Cnksr3	0.217	0.599149386954389	-
6780443	Ebf1	0.217	0.599149386954389	-
6780725	Mgat1	0.217	0.599149386954389	-
6793498	Nbas	0.217	0.599149386954389	-
6796345	Rad51l1	0.217	0.599149386954389	-
6825872	Cysltr2	0.217	0.599149386954389	-
6832294	Pnpla3	0.217	0.599149386954389	-
6836705	D730001G18Rik	0.217	0.599149386954389	-
6873133	Pik3ap1	0.217	0.599149386954389	-
6897579	Smad9	0.217	0.599149386954389	-
6899838	Prkab2	0.217	0.599149386954389	+
6905424	P2ry12	0.217	0.599149386954389	-
6927118	Tas1r1	0.217	0.599149386954389	-
6934401	Aacs	0.217	0.599149386954389	-
6966041	Shkbp1	0.217	0.599149386954389	-
7013184	Atp7a	0.217	0.599149386954389	-
7014941	Ctps2	0.217	0.599149386954389	-
6747641	Sulf1	0.218	0.599149386954389	-
6749911	Nrp2	0.218	0.599149386954389	-
6759466	Myl1	0.218	0.599149386954389	-
6777994	Rbms2	0.218	0.599149386954389	-
6792499	Fbf1	0.218	0.599149386954389	-
6792679	Cbx8	0.218	0.599149386954389	-
6793253	Matn3	0.218	0.599149386954389	-
6802156	Slc10a1	0.218	0.599149386954389	-
6803133	Fbln5	0.218	0.599149386954389	-
6849653	Rnf8	0.218	0.599149386954389	-
6850791	Usp49	0.218	0.599149386954389	-
6852887	Msh2	0.218	0.599149386954389	-
6854366	E4f1	0.218	0.599149386954389	-
6881951	Xrn2	0.218	0.599149386954389	-
6917125	Oscp1	0.218	0.599149386954389	+
6933697	Cit	0.218	0.599149386954389	-
6951276	Bicd1	0.218	0.599149386954389	-
6953073	2210010C04Rik	0.218	0.599149386954389	-
6982094	Klkb1	0.218	0.599149386954389	-
7013389	Klhl4	0.218	0.599149386954389	-
6769030	1810043G02Rik	0.219	0.599149386954389	-
6783337	Bzrap1	0.219	0.599149386954389	-
6783673	Chad	0.219	0.599149386954389	-
6784466	Wnt3	0.219	0.599149386954389	-
6789785	Doc2b	0.219	0.599149386954389	-
6804024	Wdr60	0.219	0.599149386954389	-
6830055	Oxr1	0.219	0.599149386954389	-
6831692	Lrrc14	0.219	0.599149386954389	-
6855122	Gtf2h4	0.219	0.599149386954389	-
6867975	Ints5	0.219	0.599149386954389	-
6878117	--	0.219	0.599149386954389	-
6890658	Ncaph	0.219	0.599149386954389	-
6892103	--	0.219	0.599149386954389	-

6925828	Xkr8	0.219	0.599149386954389	-
6928457	Cdk6	0.219	0.599149386954389	-
6934076	Brap	0.219	0.599149386954389	-
6939265	Clock	0.219	0.599149386954389	-
6956891	Zfp248	0.219	0.599149386954389	-
6957766	Rerg	0.219	0.599149386954389	+
6959236	Tgfb1	0.219	0.599149386954389	-
6967058	Saal1	0.219	0.599149386954389	-
6986066	2310022B05Rik	0.219	0.599149386954389	-
6998707	P4htm	0.219	0.599149386954389	-
7014128	4930521A18Rik	0.219	0.599149386954389	-
7014200	Nxt2	0.219	0.599149386954389	-
7014854	Gpr64	0.219	0.599149386954389	-
6755757	Cnih3	0.22	0.599149386954389	-
6759781	Glb1l	0.22	0.599149386954389	-
6760051	Cul3	0.22	0.599149386954389	-
6765734	Zc3h12d	0.22	0.599149386954389	-
6785418	Bahcc1	0.22	0.599149386954389	-
6791900	Ftsj3	0.22	0.599149386954389	-
6792999	0610009D07Rik	0.22	0.599149386954389	-
6796594	Ptgr2	0.22	0.599149386954389	-
6812263	Peci	0.22	0.599149386954389	-
6815543	Rad17	0.22	0.599149386954389	-
6851605	Fert2	0.22	0.599149386954389	-
6852924	Foxn2	0.22	0.599149386954389	-
6854528	Lemd2	0.22	0.599149386954389	-
6869293	Lipf	0.22	0.599149386954389	-
6871168	Capn1	0.22	0.599149386954389	+
6873476	Calhm2	0.22	0.599149386954389	-
6876109	Ncs1	0.22	0.599149386954389	-
6881252	Mavs	0.22	0.599149386954389	-
6889413	Rcn1	0.22	0.599149386954389	-
6890700	Prom2	0.22	0.599149386954389	-
6910283	4930503B20Rik	0.22	0.599149386954389	-
6913143	Rg9mtd3	0.22	0.599149386954389	-
6919596	Asph	0.22	0.599149386954389	-
6926005	Catsper4	0.22	0.599149386954389	-
6926443	Clcnkb	0.22	0.599149386954389	-
6937438	Htra3	0.22	0.599149386954389	-
6959578	AI428936	0.22	0.599149386954389	-
6962999	Fchsd2	0.22	0.599149386954389	-
6963886	Acsm1	0.22	0.599149386954389	-
6964657	Fam175b	0.22	0.599149386954389	-
6989319	Hmg20a	0.22	0.599149386954389	-
6994958	Oaf	0.22	0.599149386954389	-
6750351	Atic	0.221	0.599149386954389	-
6756345	Nsl1	0.221	0.599149386954389	-
6771643	Pan2	0.221	0.599149386954389	-
6778057	Dgka	0.221	0.599149386954389	-
6783989	Cdk12	0.221	0.599149386954389	-

6799173	Trib2	0.221	0.599149386954389	+
6813876	Slc35d2	0.221	0.599149386954389	-
6820425	Enox1	0.221	0.599149386954389	-
6824782	Efs	0.221	0.599149386954389	-
6851107	4932415M13Rik	0.221	0.599149386954389	-
6857435	Eif2ak2	0.221	0.599149386954389	-
6869021	Rcl1	0.221	0.599149386954389	-
6871138	Ehbp1l1	0.221	0.599149386954389	-
6876568	Wdr38	0.221	0.599149386954389	-
6885485	Sdccag3	0.221	0.599149386954389	-
6906427	Tlr2	0.221	0.599149386954389	-
6906753	Lmna	0.221	0.599149386954389	-
6907374	Pde4dip	0.221	0.599149386954389	+
6907576	Man1a2	0.221	0.599149386954389	-
6909516	Gstcd	0.221	0.599149386954389	-
6912527	Rngtt	0.221	0.599149386954389	-
6916618	Tctex1d4	0.221	0.599149386954389	-
6918750	Casz1	0.221	0.599149386954389	-
6929920	Afap1	0.221	0.599149386954389	-
6946034	Gimap5	0.221	0.599149386954389	-
6958052	Recql	0.221	0.599149386954389	-
6992946	Entpd3	0.221	0.599149386954389	-
7015447	Was	0.221	0.599149386954389	-
6768827	Pcdh15	0.222	0.599149386954389	-
6769148	9130017N09Rik	0.222	0.599149386954389	-
6775192	Pcbp3	0.222	0.599149386954389	-
6777355	Rap1b	0.222	0.599149386954389	-
6782102	Mgl2	0.222	0.599149386954389	-
6782610	Myo18a	0.222	0.599149386954389	-
6788838	Mapk7	0.222	0.599149386954389	-
6799857	Gpr22	0.222	0.599149386954389	-
6804582	Klf6	0.222	0.599149386954389	+
6818792	6720456H20Rik	0.222	0.599149386954389	-
6836833	Fbxl6	0.222	0.599149386954389	-
6842503	Usp25	0.222	0.599149386954389	-
6848177	Itgb2l	0.222	0.599149386954389	-
6850847	Unc5cl	0.222	0.599149386954389	-
6885919	Lrsam1	0.222	0.599149386954389	-
6907648	BC037703	0.222	0.599149386954389	-
6910621	Nexn	0.222	0.599149386954389	-
6940863	Glmn	0.222	0.599149386954389	-
6953171	Tpk1	0.222	0.599149386954389	-
6958984	Dmpk	0.222	0.599149386954389	-
6970445	Lyve1	0.222	0.599149386954389	-
6987586	Eepd1	0.222	0.599149386954389	-
6768332	3110049J23Rik	0.223	0.599149386954389	-
6770244	Mgat4c	0.223	0.599149386954389	-
6788312	Slc22a4	0.223	0.599149386954389	-
6792476	Caskin2	0.223	0.599149386954389	-
6801855	Esr2	0.223	0.599149386954389	-

6812051	Exoc2	0.223	0.599149386954389	-
6813086	1110007C09Rik	0.223	0.599149386954389	-
6848713	Slc22a2	0.223	0.599149386954389	-
6850117	Bat4	0.223	0.599149386954389	-
6867748	Eif1ad	0.223	0.599149386954389	-
6869973	Fam178a	0.223	0.599149386954389	-
6871566	Ms4a6d	0.223	0.599149386954389	-
6871645	U05342	0.223	0.599149386954389	-
6886743	Stam2	0.223	0.599149386954389	-
6890206	Ppp1r14d	0.223	0.599149386954389	-
6892417	Cpne1	0.223	0.599149386954389	-
6900139	Ap4b1	0.223	0.599149386954389	-
6915734	Usp1	0.223	0.599149386954389	-
6915889	Mier1	0.223	0.599149386954389	-
6918991	Dnajc11	0.223	0.599149386954389	-
6922536	Cdk5rap2	0.223	0.599149386954389	-
6925236	Utp11l	0.223	0.599149386954389	-
6939677	Tmprss11f	0.223	0.599149386954389	-
6951403	Pon2	0.223	0.599149386954389	-
6966328	Usf2	0.223	0.599149386954389	-
6970060	A530023O14Rik	0.223	0.599149386954389	-
6979941	1810063B05Rik	0.223	0.599149386954389	-
6986764	1700128F08Rik	0.223	0.599149386954389	-
6993722	Dnmt1	0.223	0.599149386954389	-
7016711	Elf4	0.223	0.599149386954389	-
6751641	Rnpepl1	0.224	0.599149386954389	-
6755210	Cd84	0.224	0.599149386954389	-
6760740	Ankmy1	0.224	0.599149386954389	-
6762429	Nav1	0.224	0.599149386954389	-
6775310	Rnf126	0.224	0.599149386954389	-
6778383	Gatsl3	0.224	0.599149386954389	-
6791437	Dhx58	0.224	0.599149386954389	-
6806078	Ripk1	0.224	0.599149386954389	-
6813242	Auh	0.224	0.599149386954389	-
6847556	Adamts1	0.224	0.599149386954389	+
6848568	Ezr	0.224	0.599149386954389	-
6849391	Narfl	0.224	0.599149386954389	-
6849791	Pknox1	0.224	0.599149386954389	-
6860929	Snx24	0.224	0.599149386954389	-
6863167	Epc1	0.224	0.599149386954389	-
6877356	Galnt5	0.224	0.599149386954389	-
6908958	Ndst3	0.224	0.599149386954389	-
6909153	Larp7	0.224	0.599149386954389	+
6918717	Mtor	0.224	0.599149386954389	-
6921000	Bag1	0.224	0.599149386954389	-
6927110	Thap3	0.224	0.599149386954389	-
6932224	Rufy3	0.224	0.599149386954389	-
6942579	Pilra	0.224	0.599149386954389	-
6960140	Siglecg	0.224	0.599149386954389	-
6970673	Cyp2r1	0.224	0.599149386954389	-

6971345	Sephs2	0.224	0.599149386954389	-
6976210	Asb5	0.224	0.599149386954389	-
6978883	Pla2g15	0.224	0.599149386954389	-
6988976	Drd2	0.224	0.599149386954389	-
6999278	Rbms3	0.224	0.599149386954389	-
7010347	Araf	0.224	0.599149386954389	-
6763275	Fam20b	0.225	0.599149386954389	-
6775830	4930547N16Rik	0.225	0.599149386954389	-
6782291	Trpv1	0.225	0.599149386954389	-
6785550	Fn3k	0.225	0.599149386954389	-
6785750	Pold2	0.225	0.599149386954389	-
6789797	Vps53	0.225	0.599149386954389	-
6794550	Meox2	0.225	0.599149386954389	-
6828033	Fgf14	0.225	0.599149386954389	-
6833393	Igfbp6	0.225	0.599149386954389	-
6846506	E330017A01Rik	0.225	0.599149386954389	-
6850001	Col11a2	0.225	0.599149386954389	-
6851207	Hdgfrp2	0.225	0.599149386954389	-
6859474	Fhod3	0.225	0.599149386954389	-
6874214	Sfxn4	0.225	0.599149386954389	-
6892384	Edem2	0.225	0.599149386954389	-
6913985	Rgs3	0.225	0.599149386954389	-
6916190	Orc1	0.225	0.599149386954389	-
6945108	1700025E21Rik	0.225	0.599149386954389	-
6960139	Siglec5	0.225	0.599149386954389	-
6983230	Arrdc2	0.225	0.599149386954389	-
6984991	E130303B06Rik	0.225	0.599149386954389	-
6985108	Nob1	0.225	0.599149386954389	-
6991714	Rbp1	0.225	0.599149386954389	-
6762465	--	0.226	0.599149386954389	-
6766258	Reps1	0.226	0.599149386954389	-
6769593	Pah	0.226	0.599149386954389	-
6788303	Slc22a21	0.226	0.599149386954389	-
6791666	Plcd3	0.226	0.599149386954389	-
6800082	Bzw2	0.226	0.599149386954389	-
6818186	Anxa8	0.226	0.599149386954389	-
6837805	Sbf1	0.226	0.599149386954389	-
6851303	Crb3	0.226	0.599149386954389	-
6870010	Dpcd	0.226	0.599149386954389	-
6871004	Syt12	0.226	0.599149386954389	+
6885387	Ndor1	0.226	0.599149386954389	-
6890225	Ino80	0.226	0.599149386954389	-
6893532	Bmp7	0.226	0.599149386954389	-
6899967	Trim45	0.226	0.599149386954389	-
6907669	Vangl1	0.226	0.599149386954389	-
6909083	Ugt8a	0.226	0.599149386954389	-
6929709	Gpn1	0.226	0.599149386954389	-
6933028	Aff1	0.226	0.599149386954389	+
6936575	Napepld	0.226	0.599149386954389	+
6945548	Ttc26	0.226	0.599149386954389	+

6959250	Cyp2b10	0.226	0.599149386954389	-
6960319	Fuz	0.226	0.599149386954389	-
6972512	Suv420h2	0.226	0.599149386954389	-
6996391	Usp3	0.226	0.599149386954389	-
6749711	Aox3l1	0.227	0.599149386954389	-
6762790	Rgs13	0.227	0.599149386954389	-
6769535	Timp3	0.227	0.599149386954389	-
6775864	Spic	0.227	0.599149386954389	-
6791282	Nr1d1	0.227	0.599149386954389	-
6796024	Mnat1	0.227	0.599149386954389	-
6798197	Amn	0.227	0.599149386954389	-
6811686	Hist1h2be	0.227	0.599149386954389	-
6820040	Loxl2	0.227	0.599149386954389	-
6821514	Scel	0.227	0.599149386954389	-
6837772	Ttll8	0.227	0.599149386954389	-
6854267	1520401A03Rik	0.227	0.599149386954389	-
6856290	C3	0.227	0.599149386954389	-
6872037	Tmc1	0.227	0.599149386954389	-
6876947	Lypd6	0.227	0.599149386954389	-
6881834	Polr3f	0.227	0.599149386954389	-
6894265	Kcnq2	0.227	0.599149386954389	-
6904100	Pex5l	0.227	0.599149386954389	-
6907437	Hsd3b1	0.227	0.599149386954389	-
6907707	Tshb	0.227	0.599149386954389	-
6913924	Snx30	0.227	0.599149386954389	-
6924233	Pcsk9	0.227	0.599149386954389	-
6926182	Zbtb40	0.227	0.599149386954389	-
6939019	Gabra4	0.227	0.599149386954389	-
6952936	Slc37a3	0.227	0.599149386954389	-
6974189	Cdc16	0.227	0.599149386954389	-
6987107	Mre11a	0.227	0.599149386954389	-
6992219	Rassf1	0.227	0.599149386954389	-
6992482	Ltf	0.227	0.599149386954389	-
6994585	Dcps	0.227	0.599149386954389	-
7019538	Armcx2	0.227	0.599149386954389	-
6748893	Il18rap	0.228	0.599149386954389	-
6750642	Stk11ip	0.228	0.599149386954389	-
6780978	Shroom1	0.228	0.599149386954389	-
6782921	Tmem132e	0.228	0.599149386954389	-
6784054	Cdc6	0.228	0.599149386954389	-
6817800	Lrtm1	0.228	0.599149386954389	-
6824848	Cbln3	0.228	0.599149386954389	-
6824974	F630043A04Rik	0.228	0.599149386954389	-
6835932	9130401M01Rik	0.228	0.599149386954389	-
6836962	Apol10b	0.228	0.599149386954389	-
6843685	Ppl	0.228	0.599149386954389	-
6850196	Mdc1	0.228	0.599149386954389	-
6855121	Vars2	0.228	0.599149386954389	-
6855623	Capn11	0.228	0.599149386954389	-
6857810	Lrpprc	0.228	0.599149386954389	-

6857984	Mcfd2	0.228	0.599149386954389	-
6861627	Malt1	0.228	0.599149386954389	-
6880293	Tmco5	0.228	0.599149386954389	-
6880658	Casc4	0.228	0.599149386954389	-
6890788	Anapc1	0.228	0.599149386954389	-
6896609	Actl6a	0.228	0.599149386954389	+
6915567	Hook1	0.228	0.599149386954389	-
6917069	Gnl2	0.228	0.599149386954389	-
6919091	Mmel1	0.228	0.599149386954389	-
6919944	Ripk2	0.228	0.599149386954389	-
6926304	Pla2g2f	0.228	0.599149386954389	-
6928716	Steap4	0.228	0.599149386954389	-
6942999	Ptcd1	0.228	0.599149386954389	-
6962930	P4ha3	0.228	0.599149386954389	-
6970846	Gde1	0.228	0.599149386954389	-
6977052	Mtap1s	0.228	0.599149386954389	-
6980546	Adprhl1	0.228	0.599149386954389	-
6984997	Ranbp10	0.228	0.599149386954389	-
6985817	4632415K11Rik	0.228	0.599149386954389	-
6987343	Ppan	0.228	0.599149386954389	-
6996659	Fam63b	0.228	0.599149386954389	-
6999579	Ano10	0.228	0.599149386954389	-
6758691	Stk17b	0.229	0.599149386954389	-
6764854	Iars2	0.229	0.599149386954389	-
6772009	Sash1	0.229	0.599149386954389	-
6783873	Copz2	0.229	0.599149386954389	-
6788723	Rasd1	0.229	0.599149386954389	+
6790949	Spata20	0.229	0.599149386954389	-
6802433	0610007P14Rik	0.229	0.599149386954389	-
6821304	Klf5	0.229	0.599149386954389	-
6824325	Wdhd1	0.229	0.599149386954389	-
6840507	Dlg1	0.229	0.599149386954389	-
6845943	Boc	0.229	0.599149386954389	-
6863323	Esco1	0.229	0.599149386954389	-
6864760	Diap1	0.229	0.599149386954389	-
6870424	Add3	0.229	0.599149386954389	+
6896120	Tbl1xr1	0.229	0.599149386954389	-
6904856	Pcdh18	0.229	0.599149386954389	-
6905530	Gpr149	0.229	0.599149386954389	-
6942751	Gna12	0.229	0.599149386954389	-
6957789	Eps8	0.229	0.599149386954389	-
6965967	Bckdha	0.229	0.599149386954389	-
6970675	Calca	0.229	0.599149386954389	-
6972473	Ano1	0.229	0.599149386954389	-
6979810	Cog2	0.229	0.599149386954389	-
6988721	Tmprss13	0.229	0.599149386954389	-
6989629	Uaca	0.229	0.599149386954389	-
6993104	Exosc7	0.229	0.599149386954389	-
6993131	Sacm1l	0.229	0.599149386954389	-
7015425	Hdac6	0.229	0.599149386954389	-

7018613	Dmrtc1a	0.229	0.599149386954389	-
6761998	Zranb3	0.23	0.599149386954389	-
6802744	Gtf2a1	0.23	0.599149386954389	-
6809851	Srek1ip1	0.23	0.599149386954389	-
6810688	Net1	0.23	0.599149386954389	-
6823866	Zfp488	0.23	0.599149386954389	-
6838660	Krt71	0.23	0.599149386954389	-
6864037	5730494M16Rik	0.23	0.599149386954389	-
6871060	Peli3	0.23	0.599149386954389	-
6879489	Traf6	0.23	0.599149386954389	-
6892090	Snph	0.23	0.599149386954389	-
6916147	Magoh	0.23	0.599149386954389	-
6925434	Gjb4	0.23	0.599149386954389	-
6940841	Tgfbr3	0.23	0.599149386954389	-
6941857	Abcb9	0.23	0.599149386954389	-
6958232	Ifltd1	0.23	0.599149386954389	-
6963013	Phox2a	0.23	0.599149386954389	-
6976902	Ints10	0.23	0.599149386954389	-
6977643	Rnf150	0.23	0.599149386954389	-
6985481	Adamts18	0.23	0.599149386954389	-
6995924	Cyp1a2	0.23	0.599149386954389	-
6999530	Sec22c	0.23	0.599149386954389	-
7011124	2610018G03Rik	0.23	0.599149386954389	-
7012882	Ogt	0.23	0.599149386954389	-
6765504	Rgs17	0.231	0.599149386954389	+
6767292	Rev3l	0.231	0.599149386954389	-
6789351	Nlgn2	0.231	0.599149386954389	-
6812871	Dtnbp1	0.231	0.599149386954389	-
6815022	Ckmt2	0.231	0.599149386954389	-
6824620	Zfp219	0.231	0.599149386954389	-
6829722	Osr2	0.231	0.599149386954389	-
6835938	Fbxo32	0.231	0.599149386954389	-
6851232	Uhrf1	0.231	0.599149386954389	-
6852856	Socs5	0.231	0.599149386954389	-
6869032	Cd274	0.231	0.599149386954389	-
6870134	Neurl1a	0.231	0.599149386954389	-
6904953	Elf2	0.231	0.599149386954389	-
6916510	Mobkl2c	0.231	0.599149386954389	-
6922301	Akna	0.231	0.599149386954389	-
6931453	Guf1	0.231	0.599149386954389	-
6940431	Wdfy3	0.231	0.599149386954389	-
6941932	Ncor2	0.231	0.599149386954389	+
6952121	Aass	0.231	0.599149386954389	-
6955032	Tet3	0.231	0.599149386954389	+
6957123	Grcc10	0.231	0.599149386954389	-
6964259	Sez6l2	0.231	0.599149386954389	-
6976975	Tm6sf2	0.231	0.599149386954389	-
6978845	Ctcf	0.231	0.599149386954389	-
6980941	Nek5	0.231	0.599149386954389	-
7011640	3830417A13Rik	0.231	0.599149386954389	-

7017629	Fam3a	0.231	0.599149386954389	-
6752148	Serpinb11	0.232	0.599149386954389	-
6785123	Otop3	0.232	0.599149386954389	-
6823051	Ecd	0.232	0.599149386954389	-
6860138	Pcdha6	0.232	0.599149386954389	-
6869724	Lcor	0.232	0.599149386954389	-
6874001	Ablim1	0.232	0.599149386954389	-
6875719	Entpd2	0.232	0.599149386954389	-
6884754	Fam188a	0.232	0.599149386954389	-
6927298	Vwa1	0.232	0.599149386954389	-
6957824	Slc15a5	0.232	0.599149386954389	-
6963264	Syt9	0.232	0.599149386954389	-
6965143	Athl1	0.232	0.599149386954389	-
6965893	Lypd4	0.232	0.599149386954389	-
6975064	Fut10	0.232	0.599149386954389	-
6976520	Sh3rf1	0.232	0.599149386954389	-
6978884	Slc7a6	0.232	0.599149386954389	-
6979155	Zfp1	0.232	0.599149386954389	-
6983286	Fcho1	0.232	0.599149386954389	-
6987065	Sesn3	0.232	0.599149386954389	-
6992056	Nphp3	0.232	0.599149386954389	-
6992229	Ifrd2	0.232	0.599149386954389	-
7001220	Mucl1	0.232	0.599149386954389	-
6751318	Akp3	0.233	0.599149386954389	-
6753546	Dennd1b	0.233	0.599149386954389	-
6781012	P4ha2	0.233	0.599149386954389	-
6790226	Rffl	0.233	0.599149386954389	-
6801875	Zbtb25	0.233	0.599149386954389	-
6803364	Atg2b	0.233	0.599149386954389	-
6824844	Adcy4	0.233	0.599149386954389	-
6832588	Acr	0.233	0.599149386954389	-
6850681	Gtpbp2	0.233	0.599149386954389	-
6861892	Mbd2	0.233	0.599149386954389	-
6872980	Rbp4	0.233	0.599149386954389	-
6881826	Csrp2bp	0.233	0.599149386954389	-
6885418	Lcn12	0.233	0.599149386954389	-
6912618	Cga	0.233	0.599149386954389	-
6922573	Tle1	0.233	0.599149386954389	-
6935400	Kdelr2	0.233	0.599149386954389	-
6937253	Fam53a	0.233	0.599149386954389	-
6959982	Zfp619	0.233	0.599149386954389	-
6961261	Ttc23	0.233	0.599149386954389	-
6972660	Ncr1	0.233	0.599149386954389	-
6786561	Ugp2	0.234	0.599149386954389	-
6788993	Cox10	0.234	0.599149386954389	-
6791157	Sp2	0.234	0.599149386954389	-
6796238	Fut8	0.234	0.599149386954389	-
6806640	Cd83	0.234	0.599149386954389	-
6811923	Prl7d1	0.234	0.599149386954389	-
6819249	Dcaf11	0.234	0.599149386954389	-

6819957	Kctd9	0.234	0.599149386954389	-
6824477	Exoc5	0.234	0.599149386954389	-
6837848	Syt10	0.234	0.599149386954389	-
6850060	Agpat1	0.234	0.599149386954389	-
6850175	Cchcr1	0.234	0.599149386954389	-
6854460	Rab11fip3	0.234	0.599149386954389	-
6855088	Lta	0.234	0.599149386954389	-
6860119	Ankhd1	0.234	0.599149386954389	-
6863156	Arhgap12	0.234	0.599149386954389	+
6865222	Pggt1b	0.234	0.599149386954389	-
6869580	Cyp2c65	0.234	0.599149386954389	-
6878053	Cdca7	0.234	0.599149386954389	-
6878995	Mtch2	0.234	0.599149386954389	-
6887179	Dpp4	0.234	0.599149386954389	-
6890298	Tmem87a	0.234	0.599149386954389	-
6896507	Sec62	0.234	0.599149386954389	-
6904300	Ccna2	0.234	0.599149386954389	-
6928909	Cacna2d1	0.234	0.599149386954389	-
6929702	Gckr	0.234	0.599149386954389	-
6934089	Fam109a	0.234	0.599149386954389	-
6949202	Pparg	0.234	0.599149386954389	-
6960177	Klk9	0.234	0.599149386954389	-
6969404	Pcf11	0.234	0.599149386954389	-
6988609	Mcam	0.234	0.599149386954389	-
6989233	Agphd1	0.234	0.599149386954389	-
6998879	Pth1r	0.234	0.599149386954389	-
6749933	Adam23	0.235	0.599149386954389	-
6770693	Osbpl8	0.235	0.599149386954389	-
6775377	Rexo1	0.235	0.599149386954389	-
6791915	Scn4a	0.235	0.599149386954389	-
6818696	Lgals3	0.235	0.599149386954389	-
6824785	Myh6	0.235	0.599149386954389	-
6840527	1500031L02Rik	0.235	0.599149386954389	-
6844210	Fgd4	0.235	0.599149386954389	+
6868511	BC016495	0.235	0.599149386954389	-
6871499	Dak	0.235	0.599149386954389	-
6882775	Dhx35	0.235	0.599149386954389	-
6890308	Lrrc57	0.235	0.599149386954389	-
6890712	Mall	0.235	0.599149386954389	-
6899774	Pias3	0.235	0.599149386954389	-
6904266	Ccdc144b	0.235	0.599149386954389	-
6923694	Dock7	0.235	0.599149386954389	-
6926127	Tceb3	0.235	0.599149386954389	-
6930006	Jakmip1	0.235	0.599149386954389	-
6934657	Kctd7	0.235	0.599149386954389	-
6939675	9930032O22Rik	0.235	0.599149386954389	-
6940331	Hnrpdl	0.235	0.599149386954389	-
6941754	Ift81	0.235	0.599149386954389	-
6962111	Eftud1	0.235	0.599149386954389	-
6983513	Arhgap10	0.235	0.599149386954389	-

6983898	Asna1	0.235	0.599149386954389	-
6991942	9630041A04Rik	0.235	0.599149386954389	-
6753417	Kif21b	0.236	0.599149386954389	-
6763495	Rabgap1l	0.236	0.599149386954389	-
6777998	Spryd4	0.236	0.599149386954389	-
6785348	Engase	0.236	0.599149386954389	-
6791331	Krt10	0.236	0.599149386954389	-
6792544	St6galnac2	0.236	0.599149386954389	-
6813664	Agtpbp1	0.236	0.599149386954389	-
6825305	Gata4	0.236	0.599149386954389	-
6848608	1700010I14Rik	0.236	0.599149386954389	-
6857078	Ndc80	0.236	0.599149386954389	-
6860085	Psd2	0.236	0.599149386954389	-
6864585	Sil1	0.236	0.599149386954389	-
6867622	Acy3	0.236	0.599149386954389	-
6869545	Pde6c	0.236	0.599149386954389	-
6898162	Gfm1	0.236	0.599149386954389	-
6933671	Msi1	0.236	0.599149386954389	-
6948236	Slc25a26	0.236	0.599149386954389	-
6966912	Ptov1	0.236	0.599149386954389	-
6969837	P2ry6	0.236	0.599149386954389	+
6970349	Ric3	0.236	0.599149386954389	-
6970952	Igsf6	0.236	0.599149386954389	-
6973094	Zfp28	0.236	0.599149386954389	-
6978319	Cpne2	0.236	0.599149386954389	-
6983966	Abcc12	0.236	0.599149386954389	-
6985087	Tmed6	0.236	0.599149386954389	-
6999674	Slc6a20a	0.236	0.599149386954389	-
7017668	Mpp1	0.236	0.599149386954389	-
7020156	Maged2	0.236	0.599149386954389	-
7020677	Siah1b	0.236	0.599149386954389	-
6753247	Chi3l1	0.237	0.599149386954389	-
6782449	Scarf1	0.237	0.599149386954389	-
6788903	Ncor1	0.237	0.599149386954389	-
6791409	Krt16	0.237	0.599149386954389	-
6813396	Dbn1	0.237	0.599149386954389	-
6823886	2200001I15Rik	0.237	0.599149386954389	-
6859781	Wdr33	0.237	0.599149386954389	-
6860135	Zmat2	0.237	0.599149386954389	-
6871021	Rce1	0.237	0.599149386954389	-
6882756	Adig	0.237	0.599149386954389	-
6897002	Plk4	0.237	0.599149386954389	-
6933252	Ephx4	0.237	0.599149386954389	-
6937884	Lcorl	0.237	0.599149386954389	-
6946968	Sftpb	0.237	0.599149386954389	-
6952337	Rbm28	0.237	0.599149386954389	-
6967054	Tph1	0.237	0.599149386954389	-
6969617	1810020D17Rik	0.237	0.599149386954389	-
6989086	Arhgap20	0.237	0.599149386954389	-
6989153	4930550C14Rik	0.237	0.599149386954389	-

6992278	Traip	0.237	0.599149386954389	-
6992632	Glb1	0.237	0.599149386954389	-
7015841	Efhc2	0.237	0.599149386954389	-
6762092	Daf2	0.238	0.599149386954389	-
6768985	Itgb2	0.238	0.599149386954389	-
6770076	Epyc	0.238	0.599149386954389	-
6771476	Lrig3	0.238	0.599149386954389	-
6782274	P2rx1	0.238	0.599149386954389	-
6830163	Pkhd1l1	0.238	0.599149386954389	-
6836584	Trappc9	0.238	0.599149386954389	-
6838413	Lmbr1l	0.238	0.599149386954389	-
6838450	Fmnl3	0.238	0.599149386954389	-
6838455	Nckap5l	0.238	0.599149386954389	-
6844443	Parl	0.238	0.599149386954389	-
6867963	Zbtb3	0.238	0.599149386954389	-
6871085	Pacs1	0.238	0.599149386954389	-
6877878	4933409G03Rik	0.238	0.599149386954389	-
6882374	Pofut1	0.238	0.599149386954389	-
6883717	Stx16	0.238	0.599149386954389	-
6885811	Fnbp1	0.238	0.599149386954389	-
6892868	Rims4	0.238	0.599149386954389	-
6901331	Cfi	0.238	0.599149386954389	-
6901597	Nhedc2	0.238	0.599149386954389	-
6903711	Nlgn1	0.238	0.599149386954389	-
6907262	Fcgr1	0.238	0.599149386954389	-
6949075	Cpne9	0.238	0.599149386954389	-
6974619	Mrps31	0.238	0.599149386954389	-
6974784	Thap1	0.238	0.599149386954389	-
6976937	D130040H23Rik	0.238	0.599149386954389	-
6977781	2310036O22Rik	0.238	0.599149386954389	-
6980576	1700029H14Rik	0.238	0.599149386954389	-
6982181	Ccdc111	0.238	0.599149386954389	-
6998708	Arih2	0.238	0.599149386954389	+
6998761	Fbxw15	0.238	0.599149386954389	-
6755714	Tmem63a	0.239	0.599149386954389	-
6760680	Per2	0.239	0.599149386954389	+
6777929	Mars	0.239	0.599149386954389	-
6783204	Tbx2	0.239	0.599149386954389	-
6784280	Tmem106a	0.239	0.599149386954389	-
6788613	2210407C18Rik	0.239	0.599149386954389	-
6829551	Fam173b	0.239	0.599149386954389	-
6855221	2410137M14Rik	0.239	0.599149386954389	-
6870580	Tcf7l2	0.239	0.599149386954389	-
6882454	4833413D08Rik	0.239	0.599149386954389	-
6889197	Prr5l	0.239	0.599149386954389	-
6894273	Srms	0.239	0.599149386954389	-
6917790	Fuca1	0.239	0.599149386954389	-
6926029	Ldlrap1	0.239	0.599149386954389	-
6931168	0610040J01Rik	0.239	0.599149386954389	-
6933927	Rbm19	0.239	0.599149386954389	-

6935983	A330021E22Rik	0.239	0.599149386954389	-
6939590	Tecrl	0.239	0.599149386954389	-
6941645	Dtx1	0.239	0.599149386954389	-
6942982	Kpna7	0.239	0.599149386954389	-
6945066	Fam40b	0.239	0.599149386954389	-
6945755	Sval1	0.239	0.599149386954389	-
6949086	Ttll3	0.239	0.599149386954389	-
6955879	Foxp1	0.239	0.599149386954389	-
6960915	Atp10a	0.239	0.599149386954389	-
6963914	Abca15	0.239	0.599149386954389	-
6974607	Alg11	0.239	0.599149386954389	-
6978309	Slc12a3	0.239	0.599149386954389	-
6982000	Zfp42	0.239	0.599149386954389	-
6989101	9830163H01Rik	0.239	0.599149386954389	-
6990673	Elovl5	0.239	0.599149386954389	-
6998165	Mrps22	0.239	0.599149386954389	-
7015007	Figf	0.239	0.599149386954389	-
6760347	Htr2b	0.24	0.599149386954389	-
6779588	Fancl	0.24	0.599149386954389	-
6788172	Tcf7	0.24	0.599149386954389	-
6789484	Nlrp1c-ps	0.24	0.599149386954389	-
6795904	Dact1	0.24	0.599149386954389	-
6801703	Trmt5	0.24	0.599149386954389	+
6837935	Abcd2	0.24	0.599149386954389	-
6838469	Racgap1	0.24	0.599149386954389	-
6843222	Fam165b	0.24	0.599149386954389	-
6845980	Gm609	0.24	0.599149386954389	-
6850798	Tcfeb	0.24	0.599149386954389	-
6854653	4930539E08Rik	0.24	0.599149386954389	-
6854813	Wdr4	0.24	0.599149386954389	-
6873187	Crtac1	0.24	0.599149386954389	+
6876914	Acvr2a	0.24	0.599149386954389	-
6885627	Gfi1b	0.24	0.599149386954389	-
6890120	Gpr176	0.24	0.599149386954389	-
6894212	Dido1	0.24	0.599149386954389	-
6917437	Mecr	0.24	0.599149386954389	-
6925417	Zmym1	0.24	0.599149386954389	-
6926654	Tnfrsf1b	0.24	0.599149386954389	-
6935562	Gtf3a	0.24	0.599149386954389	-
6940592	Klhl8	0.24	0.599149386954389	-
6944293	Mdfic	0.24	0.599149386954389	-
6950115	Klrb1f	0.24	0.599149386954389	-
6953929	Vopp1	0.24	0.599149386954389	-
6978232	Lpcat2	0.24	0.599149386954389	-
6995999	Cd276	0.24	0.599149386954389	-
6782643	Phf12	0.241	0.599149386954389	-
6782811	Rnf135	0.241	0.599149386954389	-
6786572	Mdh1	0.241	0.599149386954389	-
6789395	Slc16a13	0.241	0.599149386954389	-
6791328	Krt27	0.241	0.599149386954389	-

6791427	Klhl11	0.241	0.599149386954389	-
6796158	Syne2	0.241	0.599149386954389	-
6800229	Scin	0.241	0.599149386954389	-
6806034	Serpinb6b	0.241	0.599149386954389	-
6835776	Dscc1	0.241	0.599149386954389	-
6880630	Tubgcp4	0.241	0.599149386954389	-
6881499	2210009G21Rik	0.241	0.599149386954389	-
6882611	Phf20	0.241	0.599149386954389	-
6900584	Col11a1	0.241	0.599149386954389	-
6917288	Sync	0.241	0.599149386954389	-
6924358	Cpt2	0.241	0.599149386954389	-
6932200	Smr2	0.241	0.599149386954389	-
6938704	Ugdh	0.241	0.599149386954389	-
6949850	Iffo1	0.241	0.599149386954389	-
6952658	Wdr91	0.241	0.599149386954389	-
6959589	Gm1082	0.241	0.599149386954389	-
6960205	Klk1b1	0.241	0.599149386954389	-
6963534	Mical2	0.241	0.599149386954389	-
6970446	Mrvi1	0.241	0.599149386954389	-
6971358	Zfp688	0.241	0.599149386954389	-
6990295	Adam10	0.241	0.599149386954389	-
6994947	Grik4	0.241	0.599149386954389	-
6751011	9430031J16Rik	0.242	0.599149386954389	-
6758595	Myo1b	0.242	0.599149386954389	-
6775170	Slc5a4b	0.242	0.599149386954389	-
6798640	Ubxn2a	0.242	0.599149386954389	-
6808773	Edil3	0.242	0.599149386954389	-
6815027	Rasgrf2	0.242	0.599149386954389	-
6818088	Prrxl1	0.242	0.599149386954389	-
6825553	Dock5	0.242	0.599149386954389	-
6837403	Cyp2d37-ps	0.242	0.599149386954389	-
6839956	Eif4g1	0.242	0.599149386954389	-
6843244	Clic6	0.242	0.599149386954389	-
6851879	L3mbtl4	0.242	0.599149386954389	-
6854990	H2-K1	0.242	0.599149386954389	-
6861868	Ccdc68	0.242	0.599149386954389	-
6871134	Sipa1	0.242	0.599149386954389	-
6871276	Fermt3	0.242	0.599149386954389	-
6881100	Zc3h6	0.242	0.599149386954389	+
6885494	Lcn4	0.242	0.599149386954389	-
6886026	AI182371	0.242	0.599149386954389	-
6907941	Bclp2	0.242	0.599149386954389	-
6956297	Sumf1	0.242	0.599149386954389	-
6957365	Klrb1c	0.242	0.599149386954389	-
6960270	Lrrc4b	0.242	0.599149386954389	-
6964160	Il4ra	0.242	0.599149386954389	-
6972233	Muc6	0.242	0.599149386954389	-
6982649	BC088983	0.242	0.599149386954389	-
6998217	Armc8	0.242	0.599149386954389	-
7011277	Fam122c	0.242	0.599149386954389	-

6768847	Rab36	0.243	0.599149386954389	-
6772408	Hebp2	0.243	0.599149386954389	-
6787465	Mat2b	0.243	0.599149386954389	-
6797090	Flrt2	0.243	0.599149386954389	-
6813502	Lect2	0.243	0.599149386954389	-
6814151	A530095I07Rik	0.243	0.599149386954389	-
6818107	Lrrc18	0.243	0.599149386954389	-
6831511	Bai1	0.243	0.599149386954389	+
6841132	BC027231	0.243	0.599149386954389	-
6849163	Zfp758	0.243	0.599149386954389	-
6857065	Emilin2	0.243	0.599149386954389	-
6866800	Katnal2	0.243	0.599149386954389	-
6869763	Zdhhc16	0.243	0.599149386954389	-
6880900	Fgf7	0.243	0.599149386954389	-
6890979	Gfra4	0.243	0.599149386954389	-
6897573	Fam48a	0.243	0.599149386954389	-
6917249	Phc2	0.243	0.599149386954389	-
6917697	Syf2	0.243	0.599149386954389	-
6937068	Gtf3c2	0.243	0.599149386954389	-
6953128	Epha1	0.243	0.599149386954389	-
6973132	Zfp418	0.243	0.599149386954389	+
6973735	Map2k7	0.243	0.599149386954389	-
7011382	Ncrna00086	0.243	0.599149386954389	-
7020802	Tlr7	0.243	0.599149386954389	-
6759391	D630023F18Rik	0.244	0.599149386954389	+
6789355	Tnk1	0.244	0.599149386954389	-
6790538	Prr11	0.244	0.599149386954389	-
6791765	Nsf	0.244	0.599149386954389	-
6798108	Dync1h1	0.244	0.599149386954389	-
6811776	Aldh5a1	0.244	0.599149386954389	-
6823714	Phf7	0.244	0.599149386954389	-
6825600	Adamdec1	0.244	0.599149386954389	-
6835045	Ncald	0.244	0.599149386954389	-
6837394	Cyp2d13	0.244	0.599149386954389	-
6840618	Zfp148	0.244	0.599149386954389	-
6850074	Ehmt2	0.244	0.599149386954389	-
6856024	Gm5094	0.244	0.599149386954389	-
6858110	Fshr	0.244	0.599149386954389	-
6862919	Rttn	0.244	0.599149386954389	-
6870566	Acsl5	0.244	0.599149386954389	-
6870617	Nhlrc2	0.244	0.599149386954389	-
6880623	Tmem62	0.244	0.599149386954389	-
6892285	Cdk5rap1	0.244	0.599149386954389	-
6899268	Ube2q1	0.244	0.599149386954389	-
6902764	Ptger3	0.244	0.599149386954389	-
6906764	Robld3	0.244	0.599149386954389	-
6920786	3110043O21Rik	0.244	0.599149386954389	-
6927215	Prdm16	0.244	0.599149386954389	-
6929407	Actr3b	0.244	0.599149386954389	-
6957428	Klri2	0.244	0.599149386954389	-

6970891	Umod	0.244	0.599149386954389	-
6975209	Purg	0.244	0.599149386954389	-
6977804	Orc6	0.244	0.599149386954389	-
6980158	Shcbp1	0.244	0.599149386954389	-
6980871	Angpt2	0.244	0.599149386954389	-
6986733	Mmp8	0.244	0.599149386954389	-
6999522	Cck	0.244	0.599149386954389	-
7011938	Dusp9	0.244	0.599149386954389	-
6785601	Limk2	0.245	0.599149386954389	-
6815390	Utp15	0.245	0.599149386954389	-
6839539	Ntan1	0.245	0.599149386954389	-
6844544	Liph	0.245	0.599149386954389	-
6855247	Olfr112	0.245	0.599149386954389	-
6867859	Pygm	0.245	0.599149386954389	-
6883598	2010011I20Rik	0.245	0.599149386954389	-
6885213	Enkur	0.245	0.599149386954389	-
6899033	Isg20l2	0.245	0.599149386954389	-
6900369	Amigo1	0.245	0.599149386954389	-
6925156	Ppie	0.245	0.599149386954389	-
6926329	Nbl1	0.245	0.599149386954389	-
6935210	Uncx	0.245	0.599149386954389	-
6935500	Zkscan5	0.245	0.599149386954389	-
6939358	Igfbp7	0.245	0.599149386954389	-
6940005	Nup54	0.245	0.599149386954389	-
6942673	3110082I17Rik	0.245	0.599149386954389	-
6979402	Dynlrb2	0.245	0.599149386954389	-
6981905	Mtus1	0.245	0.599149386954389	-
6985944	Pabpn1l	0.245	0.599149386954389	-
6990042	Car12	0.245	0.599149386954389	-
6992852	Golga4	0.245	0.599149386954389	-
7020162	Gnl3l	0.245	0.599149386954389	-
6750413	Xrcc5	0.246	0.599149386954389	-
6782016	Lsmd1	0.246	0.599149386954389	-
6784189	Klhl10	0.246	0.599149386954389	-
6784241	Mlx	0.246	0.599149386954389	-
6784990	Cog1	0.246	0.599149386954389	-
6785207	Cdk3-ps	0.246	0.599149386954389	-
6790947	Cacna1g	0.246	0.599149386954389	+
6792129	Abca5	0.246	0.599149386954389	+
6809655	Cd180	0.246	0.599149386954389	-
6810563	Hcn1	0.246	0.599149386954389	-
6825258	Gucy1b2	0.246	0.599149386954389	-
6825443	Ephx2	0.246	0.599149386954389	-
6849890	Cyp4f39	0.246	0.599149386954389	-
6866039	Fbxo38	0.246	0.599149386954389	-
6870993	Adrbk1	0.246	0.599149386954389	-
6872256	Fxn	0.246	0.599149386954389	-
6917933	Hspg2	0.246	0.599149386954389	-
6918129	Atp13a2	0.246	0.599149386954389	-
6925533	Adc	0.246	0.599149386954389	-

6935904	Gatad1	0.246	0.599149386954389	-
6939986	Sdad1	0.246	0.599149386954389	-
6946365	Plekha8	0.246	0.599149386954389	-
6949283	March8	0.246	0.599149386954389	-
6949972	Fgf23	0.246	0.599149386954389	-
6960274	Josd2	0.246	0.599149386954389	-
6970570	Pth	0.246	0.599149386954389	-
6980578	Rasa3	0.246	0.599149386954389	-
6984218	Rpgrip1l	0.246	0.599149386954389	-
6984999	Cenpt	0.246	0.599149386954389	-
6991267	Adamts7	0.246	0.599149386954389	-
6993744	Cdc37	0.246	0.599149386954389	-
7015549	Rpgr	0.246	0.599149386954389	-
6751106	Wdr69	0.247	0.599149386954389	-
6758754	Rftn2	0.247	0.599149386954389	-
6759718	Tns1	0.247	0.599149386954389	-
6760771	Mterfd2	0.247	0.599149386954389	-
6777305	Yeats4	0.247	0.599149386954389	-
6790549	Rad51c	0.247	0.599149386954389	-
6792935	Asxl2	0.247	0.599149386954389	-
6800228	Arl4a	0.247	0.599149386954389	-
6812534	Slc35b3	0.247	0.599149386954389	-
6817668	Fam116a	0.247	0.599149386954389	-
6836999	Ift27	0.247	0.599149386954389	-
6839985	Ephb3	0.247	0.599149386954389	-
6856221	Plin3	0.247	0.599149386954389	-
6862459	Txnl4a	0.247	0.599149386954389	-
6867846	Atg2a	0.247	0.599149386954389	-
6871332	Slc22a19	0.247	0.599149386954389	-
6871493	Tmem216	0.247	0.599149386954389	-
6881031	Bcl2l11	0.247	0.599149386954389	-
6919748	Fam92a	0.247	0.599149386954389	-
6937002	Otof	0.247	0.599149386954389	-
6951453	Shfm1	0.247	0.599149386954389	-
6954982	Hk2	0.247	0.599149386954389	-
6962379	Rab38	0.247	0.599149386954389	-
6978359	Katnb1	0.247	0.599149386954389	-
6992164	Poc1a	0.247	0.599149386954389	-
7010262	Kdm6a	0.247	0.599149386954389	-
6750616	Dnajb2	0.248	0.599149386954389	-
6755787	Dnahc14	0.248	0.599149386954389	-
6759816	Chpf	0.248	0.599149386954389	-
6771224	Gns	0.248	0.599149386954389	-
6778976	Fbxo48	0.248	0.599149386954389	-
6783917	Mllt6	0.248	0.599149386954389	-
6819153	Mmp14	0.248	0.599149386954389	-
6820216	Rcbtb2	0.248	0.599149386954389	-
6828472	Dab2	0.248	0.599149386954389	-
6829504	Dap	0.248	0.599149386954389	-
6832564	Ppp6r2	0.248	0.599149386954389	-

6833387	Tenc1	0.248	0.599149386954389	-
6835946	Anxa13	0.248	0.599149386954389	-
6844269	Pi4ka	0.248	0.599149386954389	-
6847899	Donson	0.248	0.599149386954389	-
6850198	Nrm	0.248	0.599149386954389	-
6855670	Mad2l1bp	0.248	0.599149386954389	-
6861136	Phax	0.248	0.599149386954389	-
6872731	Rnls	0.248	0.599149386954389	-
6873241	Cpn1	0.248	0.599149386954389	-
6883350	Pard6b	0.248	0.599149386954389	-
6884183	Ogfr	0.248	0.599149386954389	-
6899613	Pogz	0.248	0.599149386954389	-
6903200	Snx16	0.248	0.599149386954389	-
6942739	Iqce	0.248	0.599149386954389	-
6945782	Clcn1	0.248	0.599149386954389	-
6949849	Nop2	0.248	0.599149386954389	-
6954000	Fam13a	0.248	0.599149386954389	+
6959468	Map4k1	0.248	0.599149386954389	-
6962742	Nars2	0.248	0.599149386954389	-
6977019	Lrrc25	0.248	0.599149386954389	-
6986677	Pdgfd	0.248	0.599149386954389	-
6987513	Npsr1	0.248	0.599149386954389	-
6989270	Isl2	0.248	0.599149386954389	-
6992133	Pik3r4	0.248	0.599149386954389	-
6999137	Cnot10	0.248	0.599149386954389	-
7009782	Wdr45	0.248	0.599149386954389	-
7012827	Eda	0.248	0.599149386954389	-
7020772	Egfl6	0.248	0.599149386954389	-
6751347	Sag	0.249	0.599149386954389	-
6754567	Pigc	0.249	0.599149386954389	-
6758949	Orc2	0.249	0.599149386954389	-
6763273	Tor3a	0.249	0.599149386954389	-
6763572	Fasl	0.249	0.599149386954389	-
6769147	Palm	0.249	0.599149386954389	-
6792838	Sectm1b	0.249	0.599149386954389	-
6807790	Ptdss1	0.249	0.599149386954389	-
6809551	Ccdc125	0.249	0.599149386954389	-
6813410	Ddx41	0.249	0.599149386954389	-
6819156	Rem2	0.249	0.599149386954389	+
6839847	Gnb1l	0.249	0.599149386954389	-
6850594	Supt3h	0.249	0.599149386954389	-
6854419	Tpsab1	0.249	0.599149386954389	-
6854597	Tulp1	0.249	0.599149386954389	-
6876100	Gpr107	0.249	0.599149386954389	-
6880468	D2Ertd750e	0.249	0.599149386954389	-
6885770	Sh3glb2	0.249	0.599149386954389	-
6886110	Pdcl	0.249	0.599149386954389	-
6899589	Celf3	0.249	0.599149386954389	-
6906978	Lce1a1	0.249	0.599149386954389	-
6920056	Atp6v0d2	0.249	0.599149386954389	-

6929655	Khk	0.249	0.599149386954389	-
6939704	Ugt2b5	0.249	0.599149386954389	-
6953800	Nod1	0.249	0.599149386954389	-
6966152	Samd4b	0.249	0.599149386954389	-
6994555	--	0.249	0.599149386954389	-
6996743	Tex9	0.249	0.599149386954389	-
7014406	Htr2c	0.249	0.599149386954389	-
7020371	Prdx4	0.249	0.599149386954389	-
6750560	Ttll4	0.25	0.599149386954389	-
6754519	Gas5	0.25	0.599149386954389	-
6754893	Aldh9a1	0.25	0.599149386954389	-
6760762	2310007B03Rik	0.25	0.599149386954389	-
6768145	Dnajb12	0.25	0.599149386954389	-
6771698	Wibg	0.25	0.599149386954389	-
6775876	Ano4	0.25	0.599149386954389	-
6777505	Helb	0.25	0.599149386954389	-
6778722	Abca13	0.25	0.599149386954389	-
6779392	Pus10	0.25	0.599149386954389	-
6790027	Wsb1	0.25	0.599149386954389	-
6797880	Ccnk	0.25	0.599149386954389	+
6812209	Serpinb6c	0.25	0.599149386954389	-
6833110	Ccdc65	0.25	0.599149386954389	-
6854431	Msln	0.25	0.599149386954389	-
6868041	Cybasc3	0.25	0.599149386954389	-
6875961	Mrps2	0.25	0.599149386954389	-
6879741	Pax6	0.25	0.599149386954389	-
6916267	Dmrta2	0.25	0.599149386954389	-
6918719	Exosc10	0.25	0.599149386954389	-
6926393	Padi4	0.25	0.599149386954389	-
6927192	Dffb	0.25	0.599149386954389	-
6929674	Dnajc5g	0.25	0.599149386954389	-
6958799	Obox5	0.25	0.599149386954389	-
6966231	Zfp27	0.25	0.599149386954389	-
6974770	Kcnu1	0.25	0.599149386954389	-
6996186	Kif23	0.25	0.599149386954389	-
6998094	Acpl2	0.25	0.599149386954389	-
7018804	Cysltr1	0.25	0.599149386954389	-
6751442	Agap1	0.251	0.599149386954389	-
6755869	Tlr5	0.251	0.599149386954389	-
6756812	Lrrc67	0.251	0.599149386954389	-
6764349	Rgs7	0.251	0.599149386954389	+
6783818	Ttll6	0.251	0.599149386954389	-
6784498	Mapt	0.251	0.599149386954389	-
6791298	Top2a	0.251	0.599149386954389	-
6792113	Abca8b	0.251	0.599149386954389	+
6807214	Fgfr4	0.251	0.599149386954389	-
6810592	Fgf10	0.251	0.599149386954389	+
6819152	Mrpl52	0.251	0.599149386954389	-
6828480	Fyb	0.251	0.599149386954389	-
6854448	Wdr90	0.251	0.599149386954389	-

6876342	Hspa5	0.251	0.599149386954389	+
6888340	P2rx3	0.251	0.599149386954389	-
6888785	Mdk	0.251	0.599149386954389	-
6889357	Prrg4	0.251	0.599149386954389	-
6901950	Gbp6	0.251	0.599149386954389	-
6912093	Usp45	0.251	0.599149386954389	-
6921057	Enho	0.251	0.599149386954389	-
6952151	Slc13a1	0.251	0.599149386954389	-
6959817	Ankrd27	0.251	0.599149386954389	-
6962933	Ucp3	0.251	0.599149386954389	-
6964033	Plk1	0.251	0.599149386954389	-
6965728	Psg19	0.251	0.599149386954389	-
6975050	Dusp26	0.251	0.599149386954389	-
6977758	Lyl1	0.251	0.599149386954389	-
6999455	Xirp1	0.251	0.599149386954389	-
6751690	Ano7	0.252	0.599149386954389	-
6752529	C1ql2	0.252	0.599149386954389	-
6754403	Astn1	0.252	0.599149386954389	-
6772713	Tbpl1	0.252	0.599149386954389	-
6792122	Abca9	0.252	0.599149386954389	-
6796525	Dcaf4	0.252	0.599149386954389	-
6802026	Plek2	0.252	0.599149386954389	-
6825302	Fdft1	0.252	0.599149386954389	-
6844291	Med15	0.252	0.599149386954389	+
6885901	Fpgs	0.252	0.599149386954389	-
6908486	Vcam1	0.252	0.599149386954389	-
6912999	Unc13b	0.252	0.599149386954389	-
6918145	Fbxo42	0.252	0.599149386954389	-
6922021	AI314180	0.252	0.599149386954389	-
6926974	Pik3cd	0.252	0.599149386954389	-
6929816	Tacc3	0.252	0.599149386954389	-
6931512	Gabrb1	0.252	0.599149386954389	-
6933014	Ptpn13	0.252	0.599149386954389	-
6934221	Bcl7a	0.252	0.599149386954389	-
6934668	Tyw1	0.252	0.599149386954389	-
6944184	Tmem106b	0.252	0.599149386954389	-
6947585	Figla	0.252	0.599149386954389	-
6949696	Aicda	0.252	0.599149386954389	-
6974832	Gpr124	0.252	0.599149386954389	-
6979597	Jph3	0.252	0.599149386954389	-
6985007	Dpep3	0.252	0.599149386954389	-
6990571	Hmgcll1	0.252	0.599149386954389	-
6751399	Spp2	0.253	0.599149386954389	-
6765166	Prox1	0.253	0.599149386954389	-
6784829	Map2k6	0.253	0.599149386954389	-
6823797	Chat	0.253	0.599149386954389	-
6856187	Zfp119b	0.253	0.599149386954389	-
6864444	Stard4	0.253	0.599149386954389	-
6871700	Tle4	0.253	0.599149386954389	-
6872292	Cbwd1	0.253	0.599149386954389	-

6882237	Sdcbp2	0.253	0.599149386954389	-
6888931	Alkbh3	0.253	0.599149386954389	-
6892505	Rbl1	0.253	0.599149386954389	-
6907162	Anxa9	0.253	0.599149386954389	-
6909796	Mttp	0.253	0.599149386954389	-
6912018	Cngb3	0.253	0.599149386954389	-
6921163	Gba2	0.253	0.599149386954389	-
6922890	Frem1	0.253	0.599149386954389	-
6932603	Anxa3	0.253	0.599149386954389	-
6946001	Krba1	0.253	0.599149386954389	-
6952750	Ptn	0.253	0.599149386954389	-
6978803	Ces3b	0.253	0.599149386954389	-
6983128	Zfp866	0.253	0.599149386954389	-
6988772	Apoa5	0.253	0.599149386954389	-
7014602	Gpr143	0.253	0.599149386954389	-
6753067	Ctse	0.254	0.599149386954389	-
6756358	Tmem206	0.254	0.599149386954389	-
6766380	Bclaf1	0.254	0.599149386954389	-
6781243	Cnot8	0.254	0.599149386954389	-
6785440	Hgs	0.254	0.599149386954389	-
6789268	Alox8	0.254	0.599149386954389	-
6789344	Fgf11	0.254	0.599149386954389	-
6789401	0610010K14Rik	0.254	0.599149386954389	-
6791411	Krt42	0.254	0.599149386954389	-
6792100	Wipi1	0.254	0.599149386954389	-
6793002	BC068281	0.254	0.599149386954389	-
6798628	4930417G10Rik	0.254	0.599149386954389	-
6807722	Cdk20	0.254	0.599149386954389	-
6812187	Serpinb9c	0.254	0.599149386954389	-
6820084	Reep4	0.254	0.599149386954389	-
6825304	Neil2	0.254	0.599149386954389	-
6827135	Mycbp2	0.254	0.599149386954389	-
6833153	Kcnh3	0.254	0.599149386954389	-
6837470	Sult4a1	0.254	0.599149386954389	-
6840734	Parp9	0.254	0.599149386954389	-
6850779	Guca1b	0.254	0.599149386954389	-
6859281	Dsg4	0.254	0.599149386954389	-
6884233	Arfgap1	0.254	0.599149386954389	-
6889352	Depdc7	0.254	0.599149386954389	-
6894205	Tcfl5	0.254	0.599149386954389	-
6897075	Phf17	0.254	0.599149386954389	-
6902179	Mcoln3	0.254	0.599149386954389	-
6912477	Bach2	0.254	0.599149386954389	-
6940570	1700016H13Rik	0.254	0.599149386954389	-
6953443	Igf2bp3	0.254	0.599149386954389	-
6958078	St8sia1	0.254	0.599149386954389	-
6966135	BC089491	0.254	0.599149386954389	-
6966182	Eif3k	0.254	0.599149386954389	-
6968727	Plin1	0.254	0.599149386954389	-
6974611	Thsd1	0.254	0.599149386954389	-

6985812	Taf1c	0.254	0.599149386954389	-
6997034	Tinag	0.254	0.599149386954389	-
6997626	Snx14	0.254	0.599149386954389	-
6748884	Il1r1	0.255	0.599149386954389	+
6751326	Chrnd	0.255	0.599149386954389	-
6753014	Il10	0.255	0.599149386954389	-
6753068	5430435G22Rik	0.255	0.599149386954389	-
6777182	Thap2	0.255	0.599149386954389	-
6782172	Rpain	0.255	0.599149386954389	-
6790339	Aatf	0.255	0.599149386954389	-
6791757	Gosr2	0.255	0.599149386954389	-
6814441	Rhobtb3	0.255	0.599149386954389	-
6820282	Htr2a	0.255	0.599149386954389	-
6828742	Amacr	0.255	0.599149386954389	-
6830761	Sqle	0.255	0.599149386954389	+
6831610	Zfp623	0.255	0.599149386954389	-
6832536	Mov10l1	0.255	0.599149386954389	-
6836323	Lrrc6	0.255	0.599149386954389	-
6855177	Trim39	0.255	0.599149386954389	-
6892423	Rbm39	0.255	0.599149386954389	-
6900239	Chia	0.255	0.599149386954389	-
6931602	Slain2	0.255	0.599149386954389	-
6932177	Csn1s2b	0.255	0.599149386954389	-
6942977	Smurf1	0.255	0.599149386954389	-
6945730	Ephb6	0.255	0.599149386954389	-
6949526	Ninj2	0.255	0.599149386954389	-
6960157	Zfp658	0.255	0.599149386954389	-
6960248	Klk1b4	0.255	0.599149386954389	-
6960691	Ano5	0.255	0.599149386954389	-
6962873	Mtap6	0.255	0.599149386954389	-
6971348	Zfp747	0.255	0.599149386954389	-
6973154	Clasrp	0.255	0.599149386954389	-
6992426	Smarcc1	0.255	0.599149386954389	-
7016416	Lamp2	0.255	0.599149386954389	-
7020765	Rab9	0.255	0.599149386954389	-
6756637	Rgs20	0.256	0.599149386954389	+
6772736	Eya4	0.256	0.599149386954389	-
6777684	Usp15	0.256	0.599149386954389	-
6781933	Ccdc42	0.256	0.599149386954389	-
6785631	Slc35e4	0.256	0.599149386954389	-
6785644	Ccdc157	0.256	0.599149386954389	-
6790356	Mrm1	0.256	0.599149386954389	-
6791072	Igf2bp1	0.256	0.599149386954389	-
6791388	Krt31	0.256	0.599149386954389	-
6792819	Rfng	0.256	0.599149386954389	-
6799642	Allc	0.256	0.599149386954389	-
6805573	Prl	0.256	0.599149386954389	-
6808221	Slc12a7	0.256	0.599149386954389	-
6811061	Actn2	0.256	0.599149386954389	-
6822296	Tm9sf2	0.256	0.599149386954389	-

6831800	Csf2rb	0.256	0.599149386954389	-
6832153	Xrcc6	0.256	0.599149386954389	-
6836887	Mb	0.256	0.599149386954389	-
6847348	Tmprss15	0.256	0.599149386954389	-
6850103	Ddah2	0.256	0.599149386954389	-
6855608	Tmem151b	0.256	0.599149386954389	-
6855625	Tmem63b	0.256	0.599149386954389	-
6859937	Srp19	0.256	0.599149386954389	-
6872651	Sgms1	0.256	0.599149386954389	-
6878296	Mtx2	0.256	0.599149386954389	-
6882341	Ttll9	0.256	0.599149386954389	-
6882768	Ppp1r16b	0.256	0.599149386954389	-
6912179	F730047E07Rik	0.256	0.599149386954389	-
6917389	Sdc3	0.256	0.599149386954389	-
6926150	Kdm1a	0.256	0.599149386954389	-
6934002	Oas1d	0.256	0.599149386954389	-
6950137	Clec12a	0.256	0.599149386954389	-
6966502	Zfp507	0.256	0.599149386954389	-
6966818	Siglece	0.256	0.599149386954389	-
6988636	Slc37a4	0.256	0.599149386954389	-
7017155	Mcf2	0.256	0.599149386954389	-
6750547	Ctdsp1	0.257	0.599149386954389	-
6763489	Tnn	0.257	0.599149386954389	-
6775451	Tjp3	0.257	0.599149386954389	-
6783484	Tmem100	0.257	0.599149386954389	-
6788741	Tom1l2	0.257	0.599149386954389	-
6797573	Serpina3a	0.257	0.599149386954389	-
6833094	4930415O20Rik	0.257	0.599149386954389	-
6846457	Tbc1d23	0.257	0.599149386954389	-
6855475	Tdrd6	0.257	0.599149386954389	-
6883641	Rae1	0.257	0.599149386954389	-
6906821	Efna1	0.257	0.599149386954389	+
6925785	Gmeb1	0.257	0.599149386954389	-
6931640	Spata18	0.257	0.599149386954389	-
6955228	Anxa4	0.257	0.599149386954389	-
6959143	Cd177	0.257	0.599149386954389	-
6966322	Cd22	0.257	0.599149386954389	-
6971262	Nfatc2ip	0.257	0.599149386954389	-
6971750	Dhx32	0.257	0.599149386954389	-
6972612	Ccdc106	0.257	0.599149386954389	+
6973474	Rfpl4	0.257	0.599149386954389	-
6990715	Cd109	0.257	0.599149386954389	-
6993510	Ccdc67	0.257	0.599149386954389	-
6751344	Inpp5d	0.258	0.599149386954389	-
6753085	Nucks1	0.258	0.599149386954389	-
6762670	Cfhr1	0.258	0.599149386954389	-
6768071	Pkib	0.258	0.599149386954389	-
6789311	Wrap53	0.258	0.599149386954389	-
6792516	Prpsap1	0.258	0.599149386954389	-
6828705	Rad1	0.258	0.599149386954389	-

6837301	Rangap1	0.258	0.599149386954389	-
6852068	Clip4	0.258	0.599149386954389	-
6860254	Rnf14	0.258	0.599149386954389	-
6874683	Nudt5	0.258	0.599149386954389	-
6876238	Angptl2	0.258	0.599149386954389	-
6918011	Pla2g2c	0.258	0.599149386954389	-
6921331	Igfbpl1	0.258	0.599149386954389	-
6932708	1700007G11Rik	0.258	0.599149386954389	-
6935273	Eif3b	0.258	0.599149386954389	-
6962053	Slc28a1	0.258	0.599149386954389	-
6983953	Itfg1	0.258	0.599149386954389	-
6987414	BC018242	0.258	0.599149386954389	-
6990244	Gtf2a2	0.258	0.599149386954389	-
6994030	Dpy19l1	0.258	0.599149386954389	-
6998486	1700080E11Rik	0.258	0.599149386954389	-
7014561	Kdm5c	0.258	0.599149386954389	-
6765719	Nup43	0.259	0.599149386954389	-
6767468	Scml4	0.259	0.599149386954389	-
6780368	Slu7	0.259	0.599149386954389	-
6789283	Dnahc2	0.259	0.599149386954389	-
6805797	Agtr1a	0.259	0.599149386954389	-
6825853	Fndc3a	0.259	0.599149386954389	+
6842552	Cxadr	0.259	0.599149386954389	+
6850721	Cul7	0.259	0.599149386954389	-
6881197	Ptpra	0.259	0.599149386954389	-
6884814	Trdmt1	0.259	0.599149386954389	-
6887836	Wipf1	0.259	0.599149386954389	-
6910289	Prkacb	0.259	0.599149386954389	-
6926301	Ubxn10	0.259	0.599149386954389	-
6931229	Klhl5	0.259	0.599149386954389	-
6936600	Orc5	0.259	0.599149386954389	-
6938155	Dhx15	0.259	0.599149386954389	-
6949833	C530028O21Rik	0.259	0.599149386954389	-
6952679	Slc13a4	0.259	0.599149386954389	-
6957444	Klra3	0.259	0.599149386954389	-
6971029	Palb2	0.259	0.599149386954389	-
6971393	Bcl7c	0.259	0.599149386954389	-
6974857	Poteg	0.259	0.599149386954389	-
6998639	Sema3b	0.259	0.599149386954389	-
7020771	Tceanc	0.259	0.599149386954389	-
6768990	Ube2g2	0.26	0.599149386954389	-
6778476	Ankrd36	0.26	0.599149386954389	-
6782147	Kif1c	0.26	0.599149386954389	-
6788410	Sparc	0.26	0.599149386954389	-
6789378	Acadvl	0.26	0.599149386954389	-
6791428	Acly	0.26	0.599149386954389	-
6791942	Tex2	0.26	0.599149386954389	-
6828573	Nup155	0.26	0.599149386954389	-
6843099	Hunk	0.26	0.599149386954389	-
6865267	Sema6a	0.26	0.599149386954389	-

6887286	Cobll1	0.26	0.599149386954389	-
6892055	Ninl	0.26	0.599149386954389	-
6926447	Spen	0.26	0.599149386954389	-
6938217	Sel1l3	0.26	0.599149386954389	-
6947922	BC048671	0.26	0.599149386954389	-
6949746	Clec4d	0.26	0.599149386954389	-
6953059	Clec5a	0.26	0.599149386954389	-
6955151	Fbxo41	0.26	0.599149386954389	-
6966571	Zfp536	0.26	0.599149386954389	-
6971238	Gtf3c1	0.26	0.599149386954389	-
6750643	Slc4a3	0.261	0.599149386954389	-
6754892	Tmco1	0.261	0.599149386954389	-
6771858	Vip	0.261	0.599149386954389	-
6772906	Lama2	0.261	0.599149386954389	-
6777181	Tmem19	0.261	0.599149386954389	-
6784787	Arsg	0.261	0.599149386954389	-
6789441	Chrne	0.261	0.599149386954389	-
6792477	Recql5	0.261	0.599149386954389	-
6801636	Rtn1	0.261	0.599149386954389	-
6824648	Chd8	0.261	0.599149386954389	-
6836231	Fam49b	0.261	0.599149386954389	-
6841168	Atg3	0.261	0.599149386954389	-
6852214	Spast	0.261	0.599149386954389	-
6854444	Stub1	0.261	0.599149386954389	-
6867984	Tut1	0.261	0.599149386954389	-
6869537	Cep55	0.261	0.599149386954389	-
6880035	Slc12a6	0.261	0.599149386954389	-
6884646	Itih2	0.261	0.599149386954389	-
6899148	Arhgef2	0.261	0.599149386954389	-
6902875	Rpe65	0.261	0.599149386954389	-
6907204	Ecm1	0.261	0.599149386954389	-
6951121	Mrps35	0.261	0.599149386954389	-
6966487	Gpatch1	0.261	0.599149386954389	-
6966925	Scaf1	0.261	0.599149386954389	-
6968750	2610034B18Rik	0.261	0.599149386954389	-
6969021	Arnt2	0.261	0.599149386954389	-
6986727	Mmp1a	0.261	0.599149386954389	-
6987342	A230050P20Rik	0.261	0.599149386954389	-
7015411	Ccdc120	0.261	0.599149386954389	-
7017419	Ids	0.261	0.599149386954389	-
7020716	Mospd2	0.261	0.599149386954389	-
6753179	Ppp1r15b	0.262	0.599149386954389	+
6762603	Ptprc	0.262	0.599149386954389	-
6771794	5830405N20Rik	0.262	0.599149386954389	-
6779264	Peli1	0.262	0.599149386954389	+
6780855	Clk4	0.262	0.599149386954389	-
6782405	Mnt	0.262	0.599149386954389	-
6785385	A730011L01Rik	0.262	0.599149386954389	-
6790952	Epn3	0.262	0.599149386954389	-
6796704	Mfsd7c	0.262	0.599149386954389	-

6801030	Foxa1	0.262	0.599149386954389	-
6838747	Npff	0.262	0.599149386954389	-
6839491	Ercc4	0.262	0.599149386954389	-
6846647	Arl13b	0.262	0.599149386954389	-
6852229	Ltbp1	0.262	0.599149386954389	-
6858910	Ttc39c	0.262	0.599149386954389	-
6863822	4921528I01Rik	0.262	0.599149386954389	-
6866006	Ablim3	0.262	0.599149386954389	-
6868833	Kank1	0.262	0.599149386954389	-
6877229	Kcnj3	0.262	0.599149386954389	-
6886203	Scai	0.262	0.599149386954389	-
6889908	Aqr	0.262	0.599149386954389	-
6896587	Pik3ca	0.262	0.599149386954389	-
6900180	Slc16a1	0.262	0.599149386954389	+
6918042	Ubr4	0.262	0.599149386954389	-
6924770	Tspan1	0.262	0.599149386954389	-
6925273	Zc3h12a	0.262	0.599149386954389	-
6927060	Slc45a1	0.262	0.599149386954389	-
6941739	1500011H22Rik	0.262	0.599149386954389	-
6949841	Ing4	0.262	0.599149386954389	-
6955480	Ccdc37	0.262	0.599149386954389	-
6958271	4933424B01Rik	0.262	0.599149386954389	-
6962467	Me3	0.262	0.599149386954389	-
6970459	Eif4g2	0.262	0.599149386954389	-
6980965	Ikbkb	0.262	0.599149386954389	-
6993030	1700048O20Rik	0.262	0.599149386954389	-
6747354	St18	0.263	0.599149386954389	-
6753079	Slc26a9	0.263	0.599149386954389	-
6758003	Chst10	0.263	0.599149386954389	-
6758958	Fam126b	0.263	0.599149386954389	+
6792352	Btbd17	0.263	0.599149386954389	-
6796027	Slc38a6	0.263	0.599149386954389	-
6808231	Zdhhc11	0.263	0.599149386954389	-
6810371	Arl15	0.263	0.599149386954389	-
6815698	Nln	0.263	0.599149386954389	-
6831690	Mfsd3	0.263	0.599149386954389	-
6912371	Epha7	0.263	0.599149386954389	-
6929866	Dok7	0.263	0.599149386954389	-
6929919	Ablim2	0.263	0.599149386954389	-
6956867	Ret	0.263	0.599149386954389	-
6967539	Nipa2	0.263	0.599149386954389	-
6992093	Mrpl3	0.263	0.599149386954389	-
7014836	Sh3kbp1	0.263	0.599149386954389	-
7017784	Prkx	0.263	0.599149386954389	+
6756985	Tram1	0.264	0.599149386954389	-
6782130	Gltpd2	0.264	0.599149386954389	-
6784714	Helz	0.264	0.599149386954389	-
6785746	Polm	0.264	0.599149386954389	-
6787102	Nprl3	0.264	0.599149386954389	-
6791404	Krt13	0.264	0.599149386954389	-

6795779	Klhdc1	0.264	0.599149386954389	-
6797496	Chga	0.264	0.599149386954389	+
6800723	Heatr5a	0.264	0.599149386954389	-
6804138	Sp4	0.264	0.599149386954389	-
6814451	Arsk	0.264	0.599149386954389	-
6824799	Ap1g2	0.264	0.599149386954389	-
6837787	Plxnb2	0.264	0.599149386954389	-
6856238	2410015M20Rik	0.264	0.599149386954389	-
6864529	Cdc25c	0.264	0.599149386954389	-
6865221	Trim36	0.264	0.599149386954389	-
6869294	Lipk	0.264	0.599149386954389	-
6873503	Sh3pxd2a	0.264	0.599149386954389	-
6875865	Wdr5	0.264	0.599149386954389	-
6878012	Hat1	0.264	0.599149386954389	-
6884286	Prpf6	0.264	0.599149386954389	-
6913013	Rgp1	0.264	0.599149386954389	-
6955946	Prok2	0.264	0.599149386954389	-
6963417	Zfp143	0.264	0.599149386954389	-
6967124	Mrgpra1	0.264	0.599149386954389	-
6969887	Folr2	0.264	0.599149386954389	-
6973718	Evi5l	0.264	0.599149386954389	-
6973739	Ccl25	0.264	0.599149386954389	-
6995065	Nlrx1	0.264	0.599149386954389	-
6998645	Gnat1	0.264	0.599149386954389	-
7012591	Klhl15	0.264	0.599149386954389	-
6747308	Lypla1	0.265	0.599149386954389	-
6752869	Ccnt2	0.265	0.599149386954389	-
6755307	Spna1	0.265	0.599149386954389	-
6762123	Il24	0.265	0.599149386954389	-
6773933	Ros1	0.265	0.599149386954389	-
6791407	Krt9	0.265	0.599149386954389	-
6797538	Otub2	0.265	0.599149386954389	-
6801917	Rab15	0.265	0.599149386954389	-
6803863	Ppp1r13b	0.265	0.599149386954389	-
6804535	Akr1c6	0.265	0.599149386954389	-
6815021	Zcchc9	0.265	0.599149386954389	-
6818665	Cgrrf1	0.265	0.599149386954389	-
6819442	Fgf9	0.265	0.599149386954389	-
6831867	Eif3l	0.265	0.599149386954389	-
6840369	Opa1	0.265	0.599149386954389	-
6852934	Ston1	0.265	0.599149386954389	-
6867598	Suv420h1	0.265	0.599149386954389	-
6867615	Unc93b1	0.265	0.599149386954389	-
6871545	Ms4a7	0.265	0.599149386954389	-
6872462	Glis3	0.265	0.599149386954389	-
6878329	Agps	0.265	0.599149386954389	-
6885107	4921504E06Rik	0.265	0.599149386954389	-
6885639	Gtf3c4	0.265	0.599149386954389	-
6888311	Zdhhc5	0.265	0.599149386954389	-
6925777	Oprd1	0.265	0.599149386954389	-

6928458	Fam133b	0.265	0.599149386954389	-
6945756	Sval3	0.265	0.599149386954389	-
6948964	Grm7	0.265	0.599149386954389	-
6965940	Pafah1b3	0.265	0.599149386954389	-
6966493	Tdrd12	0.265	0.599149386954389	-
6968653	Det1	0.265	0.599149386954389	-
6977751	Cacna1a	0.265	0.599149386954389	-
7017600	L1cam	0.265	0.599149386954389	-
6747919	Tcfap2b	0.266	0.599149386954389	-
6759742	Usp37	0.266	0.599149386954389	-
6768899	Gm5134	0.266	0.599149386954389	-
6782454	Pitpna	0.266	0.599149386954389	-
6790664	Dgke	0.266	0.599149386954389	-
6791265	Ormdl3	0.266	0.599149386954389	-
6792502	Srp68	0.266	0.599149386954389	-
6794104	Myt1l	0.266	0.599149386954389	-
6798067	Ppp2r5c	0.266	0.599149386954389	-
6802732	4930534B04Rik	0.266	0.599149386954389	-
6803358	Atg2b	0.266	0.599149386954389	-
6806581	Phactr1	0.266	0.599149386954389	-
6831859	Gcat	0.266	0.599149386954389	-
6837113	Ddx17	0.266	0.599149386954389	-
6837144	Pdgfb	0.266	0.599149386954389	+
6837320	Phf5a	0.266	0.599149386954389	-
6840175	Lpp	0.266	0.599149386954389	-
6845348	Sec22a	0.266	0.599149386954389	-
6864609	Tmem173	0.266	0.599149386954389	-
6879054	Lrp4	0.266	0.599149386954389	-
6881181	Ebf4	0.266	0.599149386954389	-
6909941	Bmpr1b	0.266	0.599149386954389	-
6920033	Cpne3	0.266	0.599149386954389	-
6929653	Emilin1	0.266	0.599149386954389	-
6933451	Ddx51	0.266	0.599149386954389	-
6942439	Fkbp6	0.266	0.599149386954389	-
6943093	Usp12	0.266	0.599149386954389	-
6945509	Akr1d1	0.266	0.599149386954389	-
6945957	Zfp282	0.266	0.599149386954389	-
6988898	Fam55d	0.266	0.599149386954389	-
6995402	Nnmt	0.266	0.599149386954389	-
7012774	Efnb1	0.266	0.599149386954389	-
7013222	Tbx22	0.266	0.599149386954389	-
7018594	Phka1	0.266	0.599149386954389	-
6751763	Fam174a	0.267	0.599149386954389	-
6766835	Ptprk	0.267	0.599149386954389	-
6769918	Nr2c1	0.267	0.599149386954389	-
6772232	Aig1	0.267	0.599149386954389	-
6782201	Wscd1	0.267	0.599149386954389	-
6784343	Rundc3a	0.267	0.599149386954389	-
6807336	Tgfbi	0.267	0.599149386954389	-
6836806	Parp10	0.267	0.599149386954389	-

6837791	Fam116b	0.267	0.599149386954389	-
6844806	Leprel1	0.267	0.599149386954389	-
6848761	Smoc2	0.267	0.599149386954389	-
6856192	Stap2	0.267	0.599149386954389	-
6859304	Rnf125	0.267	0.599149386954389	-
6873469	Pcgf6	0.267	0.599149386954389	-
6879512	Commd9	0.267	0.599149386954389	-
6884642	Gata3	0.267	0.599149386954389	-
6889191	B230118H07Rik	0.267	0.599149386954389	-
6890835	Ckap2l	0.267	0.599149386954389	-
6899756	Hfe2	0.267	0.599149386954389	-
6907888	Cttnbp2nl	0.267	0.599149386954389	-
6946994	Retsat	0.267	0.599149386954389	-
6956913	Wnt5b	0.267	0.599149386954389	-
6984011	Zfp423	0.267	0.599149386954389	-
6992885	Acvr2b	0.267	0.599149386954389	-
6995121	Arcn1	0.267	0.599149386954389	-
6998405	Bfsp2	0.267	0.599149386954389	-
7020314	Kctd12b	0.267	0.599149386954389	-
6792539	Cygb	0.268	0.599149386954389	+
6798212	1200009I06Rik	0.268	0.599149386954389	-
6822891	Rarb	0.268	0.599149386954389	-
6827820	Dzip1	0.268	0.599149386954389	-
6833470	Hoxc9	0.268	0.599149386954389	-
6835005	Pabpc1	0.268	0.599149386954389	-
6850092	Vars	0.268	0.599149386954389	-
6854340	Tbc1d24	0.268	0.599149386954389	-
6855201	H2-M1	0.268	0.599149386954389	-
6866504	4930503L19Rik	0.268	0.599149386954389	-
6869579	Cyp2c55	0.268	0.599149386954389	-
6880981	Snrnp200	0.268	0.599149386954389	-
6896032	Cp	0.268	0.599149386954389	-
6909993	Pkn2	0.268	0.599149386954389	-
6936750	Smarcd3	0.268	0.599149386954389	-
6950373	Loh12cr1	0.268	0.599149386954389	-
6952415	Ube2h	0.268	0.599149386954389	-
6957427	Klri1	0.268	0.599149386954389	-
6964352	Setd1a	0.268	0.599149386954389	-
6974250	Cln8	0.268	0.599149386954389	-
6992301	Ip6k1	0.268	0.599149386954389	-
6993128	Limd1	0.268	0.599149386954389	-
6994678	Slc37a2	0.268	0.599149386954389	-
6997574	Tbx18	0.268	0.599149386954389	-
6998589	Iqcf3	0.268	0.599149386954389	-
7015463	Ftsj1	0.268	0.599149386954389	-
7018664	Slc16a2	0.268	0.599149386954389	-
6750861	Mogat1	0.269	0.599149386954389	-
6759932	Pax3	0.269	0.599149386954389	-
6768931	1700027D21Rik	0.269	0.599149386954389	-
6769877	Lta4h	0.269	0.599149386954389	-

6777784	Slc16a7	0.269	0.599149386954389	-
6777932	Gli1	0.269	0.599149386954389	-
6781560	Adora2b	0.269	0.599149386954389	-
6783137	Myo19	0.269	0.599149386954389	-
6785356	Cbx2	0.269	0.599149386954389	-
6787527	Gabra1	0.269	0.599149386954389	-
6809229	Col4a3bp	0.269	0.599149386954389	-
6825084	1700129C05Rik	0.269	0.599149386954389	-
6827912	Stk24	0.269	0.599149386954389	-
6833235	AB099516	0.269	0.599149386954389	-
6835149	Slc25a32	0.269	0.599149386954389	-
6842800	Jam2	0.269	0.599149386954389	-
6868838	Dmrt1	0.269	0.599149386954389	-
6869038	C030046E11Rik	0.269	0.599149386954389	-
6872543	Ermp1	0.269	0.599149386954389	-
6881587	Sptlc3	0.269	0.599149386954389	-
6883722	Npepl1	0.269	0.599149386954389	-
6887540	Fastkd1	0.269	0.599149386954389	-
6904618	Sclt1	0.269	0.599149386954389	-
6908095	Gnai3	0.269	0.599149386954389	-
6919095	Pank4	0.269	0.599149386954389	-
6924027	Slc35d1	0.269	0.599149386954389	-
6924863	Dmap1	0.269	0.599149386954389	-
6940955	Vmn2r9	0.269	0.599149386954389	-
6947475	Dysf	0.269	0.599149386954389	-
6949211	Mkrn2	0.269	0.599149386954389	-
6949730	Clec4a4	0.269	0.599149386954389	-
6950345	Bcl2l14	0.269	0.599149386954389	-
6961889	Mrps11	0.269	0.599149386954389	-
6977648	Tbc1d9	0.269	0.599149386954389	-
6989930	Mtfmt	0.269	0.599149386954389	-
6993471	Piwil4	0.269	0.599149386954389	-
7013011	Cdx4	0.269	0.599149386954389	-
6758694	Hecw2	0.27	0.599149386954389	-
6764093	Slamf7	0.27	0.599149386954389	-
6767762	Sim1	0.27	0.599149386954389	-
6768326	Rufy2	0.27	0.599149386954389	-
6776105	Vezt	0.27	0.599149386954389	-
6779672	Efemp1	0.27	0.599149386954389	-
6813839	Fancc	0.27	0.599149386954389	-
6837045	Mfng	0.27	0.599149386954389	-
6854270	Paqr4	0.27	0.599149386954389	-
6854386	Tbl3	0.27	0.599149386954389	-
6869795	Pi4k2a	0.27	0.599149386954389	-
6898719	Ctso	0.27	0.599149386954389	-
6935927	Cyp51	0.27	0.599149386954389	+
6964382	Itgax	0.27	0.599149386954389	-
6966422	Wtip	0.27	0.599149386954389	-
6972990	Ube2m	0.27	0.599149386954389	-
6750558	Stk36	0.271	0.599149386954389	-

6752138	Serpinb13	0.271	0.599149386954389	-
6781442	Myo15	0.271	0.599149386954389	-
6788329	Csf2	0.271	0.599149386954389	-
6794073	Tssc1	0.271	0.599149386954389	-
6855116	Tcf19	0.271	0.599149386954389	-
6855394	Opn5	0.271	0.599149386954389	-
6860259	Arhgap26	0.271	0.599149386954389	-
6870996	Kdm2a	0.271	0.599149386954389	-
6886716	Nmi	0.271	0.599149386954389	-
6886732	Cacnb4	0.271	0.599149386954389	-
6899151	2810403A07Rik	0.271	0.599149386954389	-
6912521	Gabrr1	0.271	0.599149386954389	-
6925586	Iqcc	0.271	0.599149386954389	-
6928740	Abcb1a	0.271	0.599149386954389	-
6966143	Plekhg2	0.271	0.599149386954389	-
6966910	Tbc1d17	0.271	0.599149386954389	-
6972339	R74862	0.271	0.599149386954389	-
6982550	Neil3	0.271	0.599149386954389	-
6988764	Sik3	0.271	0.599149386954389	-
6989042	Fdxacb1	0.271	0.599149386954389	-
7020412	Mbtps2	0.271	0.599149386954389	-
7020863	Amelx	0.271	0.599149386954389	-
6764526	Smyd3	0.272	0.599149386954389	-
6778560	Ogdh	0.272	0.599149386954389	-
6780349	Atp10b	0.272	0.599149386954389	-
6790367	Znhit3	0.272	0.599149386954389	-
6791729	Plekhm1	0.272	0.599149386954389	-
6794152	Tmem18	0.272	0.599149386954389	-
6835945	Klhl38	0.272	0.599149386954389	-
6836778	Naprt1	0.272	0.599149386954389	-
6854311	Prss22	0.272	0.599149386954389	-
6854923	Morc2b	0.272	0.599149386954389	-
6855006	Brd2	0.272	0.599149386954389	-
6856710	1110012J17Rik	0.272	0.599149386954389	-
6913394	Rnf20	0.272	0.599149386954389	-
6916912	9530002B09Rik	0.272	0.599149386954389	-
6925880	Tmem222	0.272	0.599149386954389	-
6932198	Smr3a	0.272	0.599149386954389	-
6946339	Chn2	0.272	0.599149386954389	-
6959008	Ercc2	0.272	0.599149386954389	-
6974082	Sox1	0.272	0.599149386954389	-
6992415	Mtap4	0.272	0.599149386954389	-
6993865	Acp5	0.272	0.599149386954389	-
7009750	Akap4	0.272	0.599149386954389	-
7019944	Chrdl1	0.272	0.599149386954389	-
7020804	Prps2	0.272	0.599149386954389	-
6764274	Ifi203	0.273	0.599149386954389	-
6775508	Gm1553	0.273	0.599149386954389	-
6795885	Actr10	0.273	0.599149386954389	-
6798399	Esyt2	0.273	0.599149386954389	-

6803895	Akt1	0.273	0.599149386954389	-
6809064	Ap3b1	0.273	0.599149386954389	-
6830506	Col14a1	0.273	0.599149386954389	-
6840111	Adipoq	0.273	0.599149386954389	-
6844480	Clcn2	0.273	0.599149386954389	-
6844937	1600021P15Rik	0.273	0.599149386954389	-
6845274	Umps	0.273	0.599149386954389	-
6850257	Trim10	0.273	0.599149386954389	-
6852056	Fam179a	0.273	0.599149386954389	-
6870929	Ppp6r3	0.273	0.599149386954389	-
6871325	Lgals12	0.273	0.599149386954389	-
6876369	Cep110	0.273	0.599149386954389	-
6887958	Nfe2l2	0.273	0.599149386954389	-
6926877	2610109H07Rik	0.273	0.599149386954389	-
6933319	Pde6b	0.273	0.599149386954389	-
6934623	Mmp17	0.273	0.599149386954389	-
6943796	Gngt1	0.273	0.599149386954389	-
6958080	5730419I09Rik	0.273	0.599149386954389	-
6959007	Ppp1r13l	0.273	0.599149386954389	-
6959291	Adck4	0.273	0.599149386954389	-
6965268	Lsp1	0.273	0.599149386954389	-
6966921	Cpt1c	0.273	0.599149386954389	-
6980052	Insr	0.273	0.599149386954389	-
6985848	Gins2	0.273	0.599149386954389	-
7012010	Plxna3	0.273	0.599149386954389	-
7020542	Gja6	0.273	0.599149386954389	-
6749108	Ercc5	0.274	0.599149386954389	-
6749691	Spats2l	0.274	0.599149386954389	-
6796773	Tmem63c	0.274	0.599149386954389	-
6807228	Rgs14	0.274	0.599149386954389	-
6809810	Trim23	0.274	0.599149386954389	-
6818707	Fbxo34	0.274	0.599149386954389	-
6853248	Tfb1m	0.274	0.599149386954389	-
6854523	Ip6k3	0.274	0.599149386954389	-
6857193	Srd5a2	0.274	0.599149386954389	-
6864762	Fchsd1	0.274	0.599149386954389	-
6866660	Ska1	0.274	0.599149386954389	-
6866960	Atp9b	0.274	0.599149386954389	-
6874610	Bend7	0.274	0.599149386954389	-
6896667	Fxr1	0.274	0.599149386954389	-
6903557	Cpa3	0.274	0.599149386954389	-
6904972	4930583H14Rik	0.274	0.599149386954389	-
6919193	Cpsf3l	0.274	0.599149386954389	-
6921172	Spag8	0.274	0.599149386954389	-
6934162	P2rx7	0.274	0.599149386954389	-
6941474	Rnft2	0.274	0.599149386954389	-
6941761	Camkk2	0.274	0.599149386954389	-
6943004	Zkscan14	0.274	0.599149386954389	-
6960343	Pih1d1	0.274	0.599149386954389	-
6973314	Zfp110	0.274	0.599149386954389	-

6984032	Gm2716	0.274	0.599149386954389	-
6988194	Tmem218	0.274	0.599149386954389	-
6988604	Usp2	0.274	0.599149386954389	+
6758987	Als2cr11	0.275	0.599149386954389	-
6762765	Cdc73	0.275	0.599149386954389	-
6770013	Eea1	0.275	0.599149386954389	-
6781609	Tekt3	0.275	0.599149386954389	-
6782572	Git1	0.275	0.599149386954389	-
6782691	Spag5	0.275	0.599149386954389	-
6784345	Grn	0.275	0.599149386954389	-
6788791	Shmt1	0.275	0.599149386954389	-
6869975	Peo1	0.275	0.599149386954389	-
6871194	Snx15	0.275	0.599149386954389	-
6873365	Mgea5	0.275	0.599149386954389	-
6880972	Blvra	0.275	0.599149386954389	-
6886016	Psmd5	0.275	0.599149386954389	-
6891125	Fermt1	0.275	0.599149386954389	-
6899578	Tdrkh	0.275	0.599149386954389	-
6910142	Clca5	0.275	0.599149386954389	-
6964245	Gdpd3	0.275	0.599149386954389	-
6971033	Ern2	0.275	0.599149386954389	-
6978332	Ccl22	0.275	0.599149386954389	-
6980519	Pcid2	0.275	0.599149386954389	-
6981865	Cnot7	0.275	0.599149386954389	-
6997348	Phip	0.275	0.599149386954389	+
6753710	Uchl5	0.276	0.599149386954389	-
6754027	Fam129a	0.276	0.599149386954389	-
6780863	Nhp2	0.276	0.599149386954389	-
6792848	1110031I02Rik	0.276	0.599149386954389	-
6800236	Zfp277	0.276	0.599149386954389	-
6805134	Sfrp4	0.276	0.599149386954389	-
6813067	Zfp169	0.276	0.599149386954389	-
6817039	Sntn	0.276	0.599149386954389	-
6819369	Ift88	0.276	0.599149386954389	-
6832200	Cyp2d9	0.276	0.599149386954389	-
6839714	Mapk1	0.276	0.599149386954389	-
6840629	Heg1	0.276	0.599149386954389	-
6860034	Matr3	0.276	0.599149386954389	-
6862088	Mbd1	0.276	0.599149386954389	-
6867566	Cpt1a	0.276	0.599149386954389	-
6875825	Surf2	0.276	0.599149386954389	-
6880451	Bub1b	0.276	0.599149386954389	-
6892256	Commd7	0.276	0.599149386954389	-
6901316	Elovl6	0.276	0.599149386954389	-
6907533	Spag17-ps	0.276	0.599149386954389	-
6907864	Fam19a3	0.276	0.599149386954389	-
6907869	Mov10	0.276	0.599149386954389	-
6917153	Col8a2	0.276	0.599149386954389	-
6917817	Zfp46	0.276	0.599149386954389	-
6924810	Toe1	0.276	0.599149386954389	-

6924949	Ermap	0.276	0.599149386954389	-
6928487	Akap9	0.276	0.599149386954389	-
6935717	4930434E21Rik	0.276	0.599149386954389	-
6942669	Adap1	0.276	0.599149386954389	-
6964570	Hmx3	0.276	0.599149386954389	-
6965045	Inpp5a	0.276	0.599149386954389	+
6969016	9930013L23Rik	0.276	0.599149386954389	-
6970166	Gm4759	0.276	0.599149386954389	-
6971678	Oat	0.276	0.599149386954389	-
6975879	Lrp2bp	0.276	0.599149386954389	-
6981530	Nrg1	0.276	0.599149386954389	-
6986651	Casp12	0.276	0.599149386954389	-
6993473	1700012B09Rik	0.276	0.599149386954389	-
7009748	Dgkk	0.276	0.599149386954389	-
6772261	Vta1	0.277	0.599149386954389	-
6774738	1700040L02Rik	0.277	0.599149386954389	-
6781970	Aurkb	0.277	0.599149386954389	-
6797952	Slc25a47	0.277	0.599149386954389	-
6803102	Rps6ka5	0.277	0.599149386954389	+
6810299	Ppap2a	0.277	0.599149386954389	-
6850204	Ppp1r10	0.277	0.599149386954389	+
6850817	Trem1	0.277	0.599149386954389	-
6854381	Slc9a3r2	0.277	0.599149386954389	-
6870680	Trub1	0.277	0.599149386954389	-
6889304	Caprin1	0.277	0.599149386954389	-
6892266	Sun5	0.277	0.599149386954389	-
6901498	Ppa2	0.277	0.599149386954389	-
6902122	Sep15	0.277	0.599149386954389	-
6944262	Foxp2	0.277	0.599149386954389	-
6949884	Vwf	0.277	0.599149386954389	-
6967882	Mphosph10	0.277	0.599149386954389	-
6973692	Retn	0.277	0.599149386954389	-
6993810	Yipf2	0.277	0.599149386954389	-
6999642	Zdhhc3	0.277	0.599149386954389	-
7014035	Plp1	0.277	0.599149386954389	-
6766353	Il20ra	0.278	0.599149386954389	-
6775369	Mex3d	0.278	0.599149386954389	-
6799205	Lpin1	0.278	0.599149386954389	-
6823105	Usp54	0.278	0.599149386954389	-
6824668	Mettl3	0.278	0.599149386954389	-
6829871	Grhl2	0.278	0.599149386954389	-
6840841	Popdc2	0.278	0.599149386954389	-
6843694	Alg1	0.278	0.599149386954389	-
6844177	Mcm4	0.278	0.599149386954389	-
6849951	Myo1f	0.278	0.599149386954389	-
6854788	Tff2	0.278	0.599149386954389	-
6856133	Slc5a7	0.278	0.599149386954389	-
6857763	Kcng3	0.278	0.599149386954389	-
6868830	Dock8	0.278	0.599149386954389	-
6879022	Mybpc3	0.278	0.599149386954389	-

6891679	Ovol2	0.278	0.599149386954389	-
6893288	Atp9a	0.278	0.599149386954389	-
6907165	Setdb1	0.278	0.599149386954389	-
6912492	Mdn1	0.278	0.599149386954389	-
6917557	Wasf2	0.278	0.599149386954389	-
6918029	Tmco4	0.278	0.599149386954389	-
6935709	6330406I15Rik	0.278	0.599149386954389	-
6953611	Hoxa7	0.278	0.599149386954389	-
6969809	Dnajb13	0.278	0.599149386954389	-
6989769	Calml4	0.278	0.599149386954389	-
6993005	Nktr	0.278	0.599149386954389	-
6748539	Prss39	0.279	0.599149386954389	-
6753316	Ube2t	0.279	0.599149386954389	-
6769139	Hcn2	0.279	0.599149386954389	-
6788784	Flii	0.279	0.599149386954389	-
6813555	Ubqln1	0.279	0.599149386954389	-
6834560	Fam105a	0.279	0.599149386954389	+
6834728	Ankrd33b	0.279	0.599149386954389	+
6834890	Stk3	0.279	0.599149386954389	-
6837827	Arsa	0.279	0.599149386954389	-
6840636	Muc13	0.279	0.599149386954389	-
6854433	Haghl	0.279	0.599149386954389	-
6866962	Sall3	0.279	0.599149386954389	-
6868654	Trpm3	0.279	0.599149386954389	-
6874212	Eif3a	0.279	0.599149386954389	-
6893486	Aurka	0.279	0.599149386954389	-
6910736	Tyw3	0.279	0.599149386954389	-
6916774	Guca2a	0.279	0.599149386954389	-
6919154	Nadk	0.279	0.599149386954389	-
6929707	Zfp512	0.279	0.599149386954389	-
6941834	Zcchc8	0.279	0.599149386954389	-
6947620	Tia1	0.279	0.599149386954389	-
6949367	Zfp239	0.279	0.599149386954389	-
6957124	Atn1	0.279	0.599149386954389	-
6964533	5430419D17Rik	0.279	0.599149386954389	-
6966203	Sipa1l3	0.279	0.599149386954389	-
6973586	Apoc1	0.279	0.599149386954389	-
6979912	BC021891	0.279	0.599149386954389	-
6991979	Amotl2	0.279	0.599149386954389	-
6995158	Cd3g	0.279	0.599149386954389	-
6998647	Rbm5	0.279	0.599149386954389	-
6998752	Fbxw13	0.279	0.599149386954389	-
6761669	Ralb	0.28	0.599149386954389	-
6782679	Tlcd1	0.28	0.599149386954389	-
6786045	Ddc	0.28	0.599149386954389	-
6802031	Tmem229b	0.28	0.599149386954389	-
6812505	Txndc5	0.28	0.599149386954389	-
6816160	Gzma	0.28	0.599149386954389	-
6838713	Csad	0.28	0.599149386954389	-
6855466	Mep1a	0.28	0.599149386954389	-

6857639	Slc8a1	0.28	0.599149386954389	-
6858936	Impact	0.28	0.599149386954389	-
6867775	Rnaseh2c	0.28	0.599149386954389	-
6885789	1700001O22Rik	0.28	0.599149386954389	-
6911610	Gdf6	0.28	0.599149386954389	-
6913011	Car9	0.28	0.599149386954389	-
6922324	Tnfsf8	0.28	0.599149386954389	-
6935696	Uspl1	0.28	0.599149386954389	-
6940363	Hpse	0.28	0.599149386954389	-
6941180	Sart3	0.28	0.599149386954389	-
6947718	Ccdc48	0.28	0.599149386954389	-
6955376	Abtb1	0.28	0.599149386954389	-
6957251	Dyrk4	0.28	0.599149386954389	-
6966720	AW146154	0.28	0.599149386954389	-
6967109	Ptpn5	0.28	0.599149386954389	-
6968803	Wdr73	0.28	0.599149386954389	-
6971121	Arhgap17	0.28	0.599149386954389	-
6980089	Fcer2a	0.28	0.599149386954389	-
6981914	Fgl1	0.28	0.599149386954389	-
6984358	Ces1d	0.28	0.599149386954389	-
6984899	Tk2	0.28	0.599149386954389	-
6985427	Kars	0.28	0.599149386954389	-
6988389	9030425E11Rik	0.28	0.599149386954389	-
6753003	Pigr	0.281	0.599149386954389	-
6769165	Arid3a	0.281	0.599149386954389	-
6787909	Tgtp1	0.281	0.599149386954389	-
6818240	Wapal	0.281	0.599149386954389	-
6834408	Magi3	0.281	0.599149386954389	-
6839015	Dnase1	0.281	0.599149386954389	-
6848677	Park2	0.281	0.599149386954389	-
6865792	Fbn2	0.281	0.599149386954389	-
6870803	Emx2	0.281	0.599149386954389	-
6871277	Stip1	0.281	0.599149386954389	-
6883132	Cd40	0.281	0.599149386954389	-
6886045	Ggta1	0.281	0.599149386954389	-
6901119	Ndst4	0.281	0.599149386954389	-
6905657	Ccnl1	0.281	0.599149386954389	+
6907912	Ddx20	0.281	0.599149386954389	-
6919685	Ints8	0.281	0.599149386954389	-
6928759	9330182L06Rik	0.281	0.599149386954389	-
6933600	Myo1h	0.281	0.599149386954389	-
6940648	Gbp4	0.281	0.599149386954389	-
6956501	Rad18	0.281	0.599149386954389	-
6958193	Bcat1	0.281	0.599149386954389	-
6970840	Tmc7	0.281	0.599149386954389	-
6971317	Prrt2	0.281	0.599149386954389	-
6974612	Tpte	0.281	0.599149386954389	-
6978229	Mmp2	0.281	0.599149386954389	-
6978822	E2f4	0.281	0.599149386954389	-
6982171	Helt	0.281	0.599149386954389	-

7020844	Msl3	0.281	0.599149386954389	-
6748888	Il1rl1	0.282	0.599149386954389	-
6755242	Ccdc19	0.282	0.599149386954389	-
6775870	Utp20	0.282	0.599149386954389	-
6785079	Ttyh2	0.282	0.599149386954389	-
6785251	Mgat5b	0.282	0.599149386954389	-
6792302	Cpsf4l	0.282	0.599149386954389	-
6792550	Mxra7	0.282	0.599149386954389	-
6815126	Bhmt2	0.282	0.599149386954389	-
6816025	Gapt	0.282	0.599149386954389	-
6844542	Tmem41a	0.282	0.599149386954389	-
6856292	Gpr108	0.282	0.599149386954389	-
6867706	Mrpl11	0.282	0.599149386954389	-
6892418	Nfs1	0.282	0.599149386954389	-
6907433	Hsd3b6	0.282	0.599149386954389	-
6908350	Amy1	0.282	0.599149386954389	-
6916128	Glis1	0.282	0.599149386954389	-
6916739	Lao1	0.282	0.599149386954389	-
6925518	Zscan20	0.282	0.599149386954389	-
6929667	0610007C21Rik	0.282	0.599149386954389	-
6932339	Afm	0.282	0.599149386954389	-
6933598	Ung	0.282	0.599149386954389	-
6934945	Srrm3	0.282	0.599149386954389	-
6937442	Sh3tc1	0.282	0.599149386954389	-
6939093	Fryl	0.282	0.599149386954389	-
6942880	Zfp316	0.282	0.599149386954389	-
6943162	Flt1	0.282	0.599149386954389	-
6962107	Sh3gl3	0.282	0.599149386954389	-
6966427	4931406P16Rik	0.282	0.599149386954389	-
6975851	Mtnr1a	0.282	0.599149386954389	-
6977042	Il12rb1	0.282	0.599149386954389	-
6990683	Ick	0.282	0.599149386954389	-
6994588	Foxred1	0.282	0.599149386954389	-
6758001	Aff3	0.283	0.599149386954389	-
6763646	Prrx1	0.283	0.599149386954389	-
6764370	Pld5	0.283	0.599149386954389	-
6765459	Cr1l	0.283	0.599149386954389	-
6767270	Traf3ip2	0.283	0.599149386954389	-
6774404	Ddx21	0.283	0.599149386954389	-
6784527	Itgb3	0.283	0.599149386954389	-
6788293	Rad50	0.283	0.599149386954389	-
6791212	Pcgf2	0.283	0.599149386954389	-
6823724	Mettl6	0.283	0.599149386954389	-
6825713	Epb4.9	0.283	0.599149386954389	-
6831868	Micall1	0.283	0.599149386954389	-
6859306	Mep1b	0.283	0.599149386954389	-
6863002	Crem	0.283	0.599149386954389	-
6873065	Cyp2c70	0.283	0.599149386954389	-
6874542	Nmt2	0.283	0.599149386954389	-
6906698	Hapln2	0.283	0.599149386954389	-

6918151	Arhgef19	0.283	0.599149386954389	-
6924247	Acot11	0.283	0.599149386954389	-
6937542	Evc	0.283	0.599149386954389	-
6951292	Calcr	0.283	0.599149386954389	-
6954603	Atoh8	0.283	0.599149386954389	-
6966964	Trpm4	0.283	0.599149386954389	-
6967022	Abcc6	0.283	0.599149386954389	-
6968314	Mctp2	0.283	0.599149386954389	-
6970744	Plekha7	0.283	0.599149386954389	-
6979098	Mtss1l	0.283	0.599149386954389	-
6980938	Atp7b	0.283	0.599149386954389	-
7019532	Armcx6	0.283	0.599149386954389	-
6759621	Fn1	0.284	0.599149386954389	-
6774309	Slc29a3	0.284	0.599149386954389	-
6783360	Vezf1	0.284	0.599149386954389	-
6783654	Ankrd40	0.284	0.599149386954389	-
6789522	4933427D14Rik	0.284	0.599149386954389	-
6790221	Cct6b	0.284	0.599149386954389	-
6802485	Angel1	0.284	0.599149386954389	-
6805591	Prl8a2	0.284	0.599149386954389	-
6819259	Rec8	0.284	0.599149386954389	-
6825371	Msra	0.284	0.599149386954389	-
6825897	Nudt15	0.284	0.599149386954389	-
6838512	1700030F18Rik	0.284	0.599149386954389	-
6861376	Pde6a	0.284	0.599149386954389	-
6885432	Fcna	0.284	0.599149386954389	-
6885734	Zer1	0.284	0.599149386954389	-
6886170	Psmb7	0.284	0.599149386954389	-
6898940	Sh3d19	0.284	0.599149386954389	-
6900994	Bcar3	0.284	0.599149386954389	-
6901634	Slc39a8	0.284	0.599149386954389	-
6929149	Pmpcb	0.284	0.599149386954389	-
6935094	Zcwpw1	0.284	0.599149386954389	-
6955035	Actg2	0.284	0.599149386954389	-
6973939	Myo16	0.284	0.599149386954389	-
6989554	Celf6	0.284	0.599149386954389	-
6993890	Anln	0.284	0.599149386954389	-
6753412	Cacna1s	0.285	0.599149386954389	-
6768928	Lss	0.285	0.599149386954389	-
6782109	Slc16a11	0.285	0.599149386954389	-
6799775	Sntg2	0.285	0.599149386954389	-
6801632	2810055F11Rik	0.285	0.599149386954389	-
6811046	Ryr2	0.285	0.599149386954389	-
6824321	Gch1	0.285	0.599149386954389	-
6862827	Cbln2	0.285	0.599149386954389	-
6865100	Dpysl3	0.285	0.599149386954389	-
6871488	4930579J09Rik	0.285	0.599149386954389	-
6896519	Skil	0.285	0.599149386954389	+
6919191	Dvl1	0.285	0.599149386954389	-
6926502	Fhad1	0.285	0.599149386954389	-

6946403	Fam188b	0.285	0.599149386954389	-
6947804	Tpra1	0.285	0.599149386954389	-
6950072	Nrip2	0.285	0.599149386954389	-
6955778	Frmd4b	0.285	0.599149386954389	+
6968829	Ap3b2	0.285	0.599149386954389	-
6987403	Ldlr	0.285	0.599149386954389	-
6988962	Zw10	0.285	0.599149386954389	-
7019504	Trmt2b	0.285	0.599149386954389	-
6762660	Crb1	0.286	0.599149386954389	-
6767402	Sesn1	0.286	0.599149386954389	+
6769461	AI597468	0.286	0.599149386954389	-
6771264	BC048403	0.286	0.599149386954389	-
6772983	2310057J18Rik	0.286	0.599149386954389	-
6774408	Ccar1	0.286	0.599149386954389	-
6784244	Tubg1	0.286	0.599149386954389	-
6784371	Ccdc103	0.286	0.599149386954389	-
6784587	Ace	0.286	0.599149386954389	-
6788651	Obscn	0.286	0.599149386954389	-
6795453	Mia2	0.286	0.599149386954389	-
6802989	Galc	0.286	0.599149386954389	-
6806673	Jarid2	0.286	0.599149386954389	-
6833919	Skp2	0.286	0.599149386954389	-
6840115	St6gal1	0.286	0.599149386954389	-
6854872	Akap8l	0.286	0.599149386954389	-
6875182	Slc39a12	0.286	0.599149386954389	-
6884986	Nebl	0.286	0.599149386954389	-
6890114	Fsip1	0.286	0.599149386954389	-
6913269	Col15a1	0.286	0.599149386954389	-
6924877	St3gal3	0.286	0.599149386954389	-
6926462	Tmem82	0.286	0.599149386954389	-
6935701	Alox5ap	0.286	0.599149386954389	-
6937270	Letm1	0.286	0.599149386954389	-
6943404	Stard13	0.286	0.599149386954389	-
6951882	Tcfec	0.286	0.599149386954389	-
6954988	Sema4f	0.286	0.599149386954389	-
6963964	Eef2k	0.286	0.599149386954389	-
6966991	Ppp1r15a	0.286	0.599149386954389	+
6972175	Ano9	0.286	0.599149386954389	-
6997255	Impg1	0.286	0.599149386954389	-
6998434	Acpp	0.286	0.599149386954389	-
6783045	Heatr6	0.287	0.599149386954389	-
6785632	Tcn2	0.287	0.599149386954389	-
6790360	Ggnbp2	0.287	0.599149386954389	-
6791017	Itga3	0.287	0.599149386954389	-
6792031	Prkca	0.287	0.599149386954389	-
6814050	Zfp58	0.287	0.599149386954389	-
6828379	Plcxd3	0.287	0.599149386954389	-
6835065	Ubr5	0.287	0.599149386954389	-
6848572	Fgfr1op	0.287	0.599149386954389	-
6849070	Zfp51	0.287	0.599149386954389	-

6859779	Polr2d	0.287	0.599149386954389	-
6860914	Gykl1	0.287	0.599149386954389	-
6863299	Usp14	0.287	0.599149386954389	-
6871128	Kat5	0.287	0.599149386954389	-
6879621	Abtb2	0.287	0.599149386954389	-
6888928	Accs	0.287	0.599149386954389	-
6892997	Zfp334	0.287	0.599149386954389	-
6900191	Ppm1j	0.287	0.599149386954389	-
6900236	Wdr77	0.287	0.599149386954389	-
6906225	Tmem144	0.287	0.599149386954389	+
6960436	Nomo1	0.287	0.599149386954389	-
6988411	Bsx	0.287	0.599149386954389	-
7016421	Cul4b	0.287	0.599149386954389	-
6763840	Mael	0.288	0.599149386954389	-
6786049	Cobl	0.288	0.599149386954389	-
6786585	Otx1	0.288	0.599149386954389	-
6798090	Dio3	0.288	0.599149386954389	-
6802035	Pigh	0.288	0.599149386954389	-
6813880	Cdc14b	0.288	0.599149386954389	-
6831687	Kifc2	0.288	0.599149386954389	-
6840094	Fetub	0.288	0.599149386954389	-
6856279	Tubb4	0.288	0.599149386954389	-
6861629	5330437I02Rik	0.288	0.599149386954389	-
6871078	Klc2	0.288	0.599149386954389	-
6873282	Ndufb8	0.288	0.599149386954389	-
6899966	Vtcn1	0.288	0.599149386954389	-
6910123	Clca1	0.288	0.599149386954389	-
6920725	Orc3	0.288	0.599149386954389	-
6926171	Epha8	0.288	0.599149386954389	-
6932883	Agpat9	0.288	0.599149386954389	-
6939664	Uba6	0.288	0.599149386954389	-
6941214	Trpv4	0.288	0.599149386954389	-
6964253	Hirip3	0.288	0.599149386954389	-
6964370	Bckdk	0.288	0.599149386954389	-
6968027	Lrrc28	0.288	0.599149386954389	-
6974040	1700018L24Rik	0.288	0.599149386954389	-
6987350	Icam1	0.288	0.599149386954389	-
6990438	2410004A20Rik	0.288	0.599149386954389	-
6992117	Aste1	0.288	0.599149386954389	-
6998020	Trpc1	0.288	0.599149386954389	-
6998676	Tcta	0.288	0.599149386954389	-
6759613	Bard1	0.289	0.599149386954389	-
6760895	Slco6b1	0.289	0.599149386954389	-
6763132	Lamc1	0.289	0.599149386954389	-
6769209	Apc2	0.289	0.599149386954389	+
6784270	Ifi35	0.289	0.599149386954389	-
6785247	Mfsd11	0.289	0.599149386954389	-
6785549	Fn3krp	0.289	0.599149386954389	+
6803132	Tc2n	0.289	0.599149386954389	-
6810260	Il6st	0.289	0.599149386954389	-

6818067	Parg	0.289	0.599149386954389	-
6824755	Acin1	0.289	0.599149386954389	-
6840491	Bdh1	0.289	0.599149386954389	-
6843811	Tmem114	0.289	0.599149386954389	-
6846006	Phldb2	0.289	0.599149386954389	-
6851763	ORF19	0.289	0.599149386954389	-
6855219	Znrd1	0.289	0.599149386954389	-
6882474	Zfp341	0.289	0.599149386954389	-
6887081	Ly75	0.289	0.599149386954389	-
6896615	Usp13	0.289	0.599149386954389	-
6902493	Fubp1	0.289	0.599149386954389	-
6913531	Nipsnap3b	0.289	0.599149386954389	-
6929252	Rint1	0.289	0.599149386954389	-
6935117	Ap4m1	0.289	0.599149386954389	-
6937069	Eif2b4	0.289	0.599149386954389	-
6940954	Tmed11	0.289	0.599149386954389	-
6955347	Sec61a1	0.289	0.599149386954389	-
6957437	Klra8	0.289	0.599149386954389	-
6960130	I1C0022H11Rik	0.289	0.599149386954389	-
6966329	Lsr	0.289	0.599149386954389	-
6974763	Star	0.289	0.599149386954389	-
6988571	Trim29	0.289	0.599149386954389	-
6992473	Prss50	0.289	0.599149386954389	-
6993838	Dock6	0.289	0.599149386954389	-
6751726	Neu4	0.29	0.599149386954389	-
6754701	Nme7	0.29	0.599149386954389	-
6760446	Ngef	0.29	0.599149386954389	-
6770445	Ccdc59	0.29	0.599149386954389	-
6774395	Supv3l1	0.29	0.599149386954389	-
6782457	Myo1c	0.29	0.599149386954389	-
6792491	Unc13d	0.29	0.599149386954389	-
6792702	Sgsh	0.29	0.599149386954389	+
6809223	Poc5	0.29	0.599149386954389	-
6813398	Pdlim7	0.29	0.599149386954389	-
6819752	Prss52	0.29	0.599149386954389	-
6824846	Ripk3	0.29	0.599149386954389	-
6832857	Tmem117	0.29	0.599149386954389	-
6836842	Nfkbil2	0.29	0.599149386954389	-
6839057	Dnaja3	0.29	0.599149386954389	-
6843343	Chaf1b	0.29	0.599149386954389	-
6843674	Anks3	0.29	0.599149386954389	-
6854654	Pxt1	0.29	0.599149386954389	-
6886039	Stom	0.29	0.599149386954389	-
6886183	Nr6a1	0.29	0.599149386954389	-
6891000	Rnf24	0.29	0.599149386954389	-
6898954	Lrba	0.29	0.599149386954389	-
6924721	Cyp4b1	0.29	0.599149386954389	-
6924763	Rad54l	0.29	0.599149386954389	-
6927203	Trp73	0.29	0.599149386954389	-
6931822	Exoc1	0.29	0.599149386954389	-

6942753	Card11	0.29	0.599149386954389	-
6950103	Clec2i	0.29	0.599149386954389	-
6953058	Prss37	0.29	0.599149386954389	-
6957263	Ccnd2	0.29	0.599149386954389	-
7015363	Ccnb3	0.29	0.599149386954389	-
6765199	Vash2	0.291	0.599149386954389	-
6765307	Sertad4	0.291	0.599149386954389	-
6766352	--	0.291	0.599149386954389	-
6769193	Midn	0.291	0.599149386954389	+
6774403	2510003E04Rik	0.291	0.599149386954389	-
6785450	Gcgr	0.291	0.599149386954389	-
6791529	Mpp3	0.291	0.599149386954389	+
6792097	Slc16a6	0.291	0.599149386954389	-
6792822	Fasn	0.291	0.599149386954389	-
6795234	Psma6	0.291	0.599149386954389	-
6796580	Dnalc1	0.291	0.599149386954389	-
6798310	Tex22	0.291	0.599149386954389	-
6823721	Sh3bp5	0.291	0.599149386954389	-
6831157	Phf20l1	0.291	0.599149386954389	-
6833286	Scn8a	0.291	0.599149386954389	-
6849775	Slc37a1	0.291	0.599149386954389	-
6856254	Nrtn	0.291	0.599149386954389	-
6906328	Gucy1a3	0.291	0.599149386954389	-
6907089	2310007A19Rik	0.291	0.599149386954389	-
6917549	Fgr	0.291	0.599149386954389	-
6928965	Sema3c	0.291	0.599149386954389	-
6946749	Tnip3	0.291	0.599149386954389	-
6975658	Slc7a2	0.291	0.599149386954389	-
6984684	Cdh8	0.291	0.599149386954389	+
6992468	Prss43	0.291	0.599149386954389	-
6997215	Tmem30a	0.291	0.599149386954389	-
7008603	Vmn1r172	0.291	0.599149386954389	-
6751699	Farp2	0.292	0.599149386954389	-
6758414	Col5a2	0.292	0.599149386954389	-
6760674	Ilkap	0.292	0.599149386954389	-
6777296	Cct2	0.292	0.599149386954389	-
6780781	3010026O09Rik	0.292	0.599149386954389	-
6806938	Bicd2	0.292	0.599149386954389	-
6817690	Hesx1	0.292	0.599149386954389	-
6840778	Hcls1	0.292	0.599149386954389	-
6856689	Twsg1	0.292	0.599149386954389	-
6873309	Pdzd7	0.292	0.599149386954389	-
6876022	Coq4	0.292	0.599149386954389	-
6929713	Mrpl33	0.292	0.599149386954389	-
6932951	Arhgap24	0.292	0.599149386954389	-
6933409	Ankle2	0.292	0.599149386954389	-
6935498	Cpsf4	0.292	0.599149386954389	-
6939077	Tec	0.292	0.599149386954389	-
6941672	Trafd1	0.292	0.599149386954389	-
6946707	Grid2	0.292	0.599149386954389	-

6950725	Slco1c1	0.292	0.599149386954389	-
6958269	Itpr2	0.292	0.599149386954389	-
6974137	Cul4a	0.292	0.599149386954389	-
6975661	Pdgfrl	0.292	0.599149386954389	-
6975930	Enpp6	0.292	0.599149386954389	-
6992477	Lrrc2	0.292	0.599149386954389	-
6995190	Il10ra	0.292	0.599149386954389	-
6749473	Osgepl1	0.293	0.599149386954389	-
6764007	Hsd17b7	0.293	0.599149386954389	+
6765454	Cd46	0.293	0.599149386954389	-
6775846	Chpt1	0.293	0.599149386954389	-
6806021	Wrnip1	0.293	0.599149386954389	-
6811904	Prl8a8	0.293	0.599149386954389	-
6817903	Chdh	0.293	0.599149386954389	-
6832117	L3mbtl2	0.293	0.599149386954389	-
6837773	Mlc1	0.293	0.599149386954389	-
6849109	Zfp760	0.293	0.599149386954389	-
6854514	Bak1	0.293	0.599149386954389	-
6858134	Nrxn1	0.293	0.599149386954389	-
6877901	Nostrin	0.293	0.599149386954389	-
6907439	Hao2	0.293	0.599149386954389	-
6915036	Cntln	0.293	0.599149386954389	-
6924287	2210012G02Rik	0.293	0.599149386954389	-
6927244	Ski	0.293	0.599149386954389	-
6929300	Accn3	0.293	0.599149386954389	-
6935041	Cldn15	0.293	0.599149386954389	-
6964428	Inpp5f	0.293	0.599149386954389	-
6966967	Ppfia3	0.293	0.599149386954389	-
6968762	Idh2	0.293	0.599149386954389	-
6983819	Il27ra	0.293	0.599149386954389	-
6985987	1300018I17Rik	0.293	0.599149386954389	-
6992942	Myrip	0.293	0.599149386954389	-
7014503	Alas2	0.293	0.599149386954389	-
6764835	Mosc2	0.294	0.599149386954389	-
6779373	Xpo1	0.294	0.599149386954389	-
6782080	Plscr3	0.294	0.599149386954389	-
6782096	Asgr1	0.294	0.599149386954389	-
6789331	Tnfsf12	0.294	0.599149386954389	-
6819885	Ccdc25	0.294	0.599149386954389	-
6824867	Ctsg	0.294	0.599149386954389	-
6829360	Ank	0.294	0.599149386954389	-
6837368	Naga	0.294	0.599149386954389	-
6844254	Ccdc116	0.294	0.599149386954389	-
6858096	Lhcgr	0.294	0.599149386954389	-
6860576	Kcnn2	0.294	0.599149386954389	-
6870958	Aldh3b1	0.294	0.599149386954389	-
6873255	Pkd2l1	0.294	0.599149386954389	-
6876735	Kynu	0.294	0.599149386954389	-
6881820	8430406I07Rik	0.294	0.599149386954389	-
6882554	Procr	0.294	0.599149386954389	-

6890981	Siglec1	0.294	0.599149386954389	-
6896997	Hspa4l	0.294	0.599149386954389	-
6915138	Acer2	0.294	0.599149386954389	+
6925361	Tekt2	0.294	0.599149386954389	-
6937081	Ift172	0.294	0.599149386954389	-
6944750	Hyal6	0.294	0.599149386954389	-
6947445	Vax2	0.294	0.599149386954389	-
6948032	C130022K22Rik	0.294	0.599149386954389	-
6963003	Stard10	0.294	0.599149386954389	-
6965592	Crx	0.294	0.599149386954389	-
6968796	Crtc3	0.294	0.599149386954389	-
6971227	Nsmce1	0.294	0.599149386954389	-
6983327	Cherp	0.294	0.599149386954389	-
6983799	Cd97	0.294	0.599149386954389	-
6989870	Snapc5	0.294	0.599149386954389	-
6992217	Nprl2	0.294	0.599149386954389	-
6999070	Pdcd6ip	0.294	0.599149386954389	-
6768618	Slc16a9	0.295	0.599149386954389	-
6775171	Prmt2	0.295	0.599149386954389	-
6787947	Btnl9	0.295	0.599149386954389	-
6789420	Cxcl16	0.295	0.599149386954389	-
6792945	Dnmt3a	0.295	0.599149386954389	-
6816288	Isl1	0.295	0.599149386954389	-
6846356	Zpld1	0.295	0.599149386954389	-
6876173	Bat2l	0.295	0.599149386954389	-
6885486	Inpp5e	0.295	0.599149386954389	-
6899315	Tpm3	0.295	0.599149386954389	-
6906902	2500003M10Rik	0.295	0.599149386954389	-
6936747	Abcf2	0.295	0.599149386954389	-
6940646	Gbp8	0.295	0.599149386954389	-
6961766	Klhl25	0.295	0.599149386954389	-
6965324	Slc22a18	0.295	0.599149386954389	-
6971266	Atp2a1	0.295	0.599149386954389	-
6975614	Vps37a	0.295	0.599149386954389	-
6986719	Mmp13	0.295	0.599149386954389	-
6991873	Stag1	0.295	0.599149386954389	-
6993086	D9Ertd402e	0.295	0.599149386954389	-
6996202	Spesp1	0.295	0.599149386954389	-
6997671	Tmed3	0.295	0.599149386954389	-
6752873	Rab3gap1	0.296	0.599149386954389	-
6767519	Bend3	0.296	0.599149386954389	-
6775146	Susd2	0.296	0.599149386954389	-
6782088	Dullard	0.296	0.599149386954389	-
6789274	Chd3	0.296	0.599149386954389	-
6791995	Pitpnc1	0.296	0.599149386954389	-
6825595	Adam7	0.296	0.599149386954389	-
6842009	Pros1	0.296	0.599149386954389	-
6849388	Rpusd1	0.296	0.599149386954389	-
6850834	Trem2	0.296	0.599149386954389	-
6851263	Tmem146	0.296	0.599149386954389	-

6860666	Commd10	0.296	0.599149386954389	-
6865926	Ndst1	0.296	0.599149386954389	-
6870027	Nolc1	0.296	0.599149386954389	-
6885751	Ccbl1	0.296	0.599149386954389	-
6917298	Bsdc1	0.296	0.599149386954389	-
6918307	Pramel1	0.296	0.599149386954389	-
6924017	Wdr78	0.296	0.599149386954389	-
6929995	Ppp2r2c	0.296	0.599149386954389	-
6953527	Npvf	0.296	0.599149386954389	-
6957107	Clstn3	0.296	0.599149386954389	-
6966490	C230052I12Rik	0.296	0.599149386954389	-
6978855	Pard6a	0.296	0.599149386954389	-
6983624	Abce1	0.296	0.599149386954389	-
6989452	Stoml1	0.296	0.599149386954389	-
6993717	Angptl6	0.296	0.599149386954389	-
6997667	AF529169	0.296	0.599149386954389	-
6998340	Cep63	0.296	0.599149386954389	-
7017595	Idh3g	0.296	0.599149386954389	-
6751332	Eif4e2	0.297	0.599149386954389	-
6767231	Lama4	0.297	0.599149386954389	-
6771558	Dctn2	0.297	0.599149386954389	-
6773421	BC021785	0.297	0.599149386954389	-
6780777	Rnf130	0.297	0.599149386954389	-
6783642	Tob1	0.297	0.599149386954389	+
6788411	Atox1	0.297	0.599149386954389	-
6811663	Btn1a1	0.297	0.599149386954389	-
6814448	Rfesd	0.297	0.599149386954389	-
6815538	Gtf2h2	0.297	0.599149386954389	-
6816148	Skiv2l2	0.297	0.599149386954389	-
6844283	Slc7a4	0.297	0.599149386954389	-
6869997	Btrc	0.297	0.599149386954389	-
6871125	Ovol1	0.297	0.599149386954389	-
6881631	Macrod2	0.297	0.599149386954389	-
6886981	Ccdc148	0.297	0.599149386954389	-
6909495	Aimp1	0.297	0.599149386954389	-
6926853	Mfn2	0.297	0.599149386954389	-
6932718	Bmp3	0.297	0.599149386954389	-
6946386	Znrf2	0.297	0.599149386954389	-
6951302	Tfpi2	0.297	0.599149386954389	-
6953505	Dfna5	0.297	0.599149386954389	-
6965233	Brsk2	0.297	0.599149386954389	-
6974114	Mcf2l	0.297	0.599149386954389	-
6979110	Cog4	0.297	0.599149386954389	-
6984971	Kctd19	0.297	0.599149386954389	-
6988958	Usp28	0.297	0.599149386954389	-
6989904	2010321M09Rik	0.297	0.599149386954389	-
6998871	Nradd	0.297	0.599149386954389	-
6750568	Wnt10a	0.298	0.599149386954389	-
6762312	Zc3h11a	0.298	0.599149386954389	-
6763748	Tbx19	0.298	0.599149386954389	-

6769262	Thop1	0.298	0.599149386954389	-
6771139	Tmbim4	0.298	0.599149386954389	-
6784485	Arf2	0.298	0.599149386954389	+
6813244	Ror2	0.298	0.599149386954389	-
6818498	Mat1a	0.298	0.599149386954389	-
6838662	Krt74	0.298	0.599149386954389	-
6838730	Aaas	0.298	0.599149386954389	-
6843680	Rogdi	0.298	0.599149386954389	+
6849363	Prss29	0.298	0.599149386954389	-
6856668	Tmem232	0.298	0.599149386954389	-
6868126	Gif	0.298	0.599149386954389	-
6890230	Exd1	0.298	0.599149386954389	-
6906086	Serpini2	0.298	0.599149386954389	-
6907306	Cd160	0.298	0.599149386954389	-
6917520	Eya3	0.298	0.599149386954389	-
6926505	9030409G11Rik	0.298	0.599149386954389	-
6932141	Ugt2b35	0.298	0.599149386954389	-
6935088	Tsc22d4	0.298	0.599149386954389	-
6935524	Cdk8	0.298	0.599149386954389	-
6936104	Crot	0.298	0.599149386954389	-
6954415	Il12rb2	0.298	0.599149386954389	-
6956572	Tmem111	0.298	0.599149386954389	-
6958993	Eml2	0.298	0.599149386954389	-
6988711	Amica1	0.298	0.599149386954389	-
6992280	Uba7	0.298	0.599149386954389	-
6993739	Tyk2	0.298	0.599149386954389	-
7013735	Diap2	0.298	0.599149386954389	-
7015002	Ace2	0.298	0.599149386954389	-
7017678	Mtcp1	0.298	0.599149386954389	-
6753033	Lgtn	0.299	0.599149386954389	-
6754666	Kifap3	0.299	0.599149386954389	-
6756383	Ints7	0.299	0.599149386954389	-
6757732	Prim2	0.299	0.599149386954389	-
6757896	Uggt1	0.299	0.599149386954389	-
6762019	Dars	0.299	0.599149386954389	-
6763631	Fmo3	0.299	0.599149386954389	-
6774400	Srgn	0.299	0.599149386954389	-
6775410	Timm13	0.299	0.599149386954389	-
6778043	Pa2g4	0.299	0.599149386954389	-
6784594	Map3k3	0.299	0.599149386954389	-
6792534	Rhbdf2	0.299	0.599149386954389	-
6802180	Slc8a3	0.299	0.599149386954389	-
6803918	Nudt14	0.299	0.599149386954389	-
6818315	Lrit2	0.299	0.599149386954389	-
6826219	Tnfsf11	0.299	0.599149386954389	-
6837279	St13	0.299	0.599149386954389	-
6840565	Pcyt1a	0.299	0.599149386954389	-
6840807	Hgd	0.299	0.599149386954389	-
6853934	Dll1	0.299	0.599149386954389	-
6859836	Wdr36	0.299	0.599149386954389	-

6868676	Klf9	0.299	0.599149386954389	-
6868899	Vldlr	0.299	0.599149386954389	-
6873944	Nrap	0.299	0.599149386954389	-
6882711	Rprd1b	0.299	0.599149386954389	-
6885360	Noxa1	0.299	0.599149386954389	-
6916562	Gpbp1l1	0.299	0.599149386954389	-
6934116	Myl2	0.299	0.599149386954389	-
6935387	Zfp12	0.299	0.599149386954389	-
6957111	C1s	0.299	0.599149386954389	-
6957584	Lrp6	0.299	0.599149386954389	-
6960203	Klk1b8	0.299	0.599149386954389	-
6962759	Kctd14	0.299	0.599149386954389	-
6965130	Ric8	0.299	0.599149386954389	-
6965225	Muc5b	0.299	0.599149386954389	-
6974647	Ank1	0.299	0.599149386954389	-
6977993	Nkd1	0.299	0.599149386954389	-
6983979	N4bp1	0.299	0.599149386954389	-
6985008	Dpep2	0.299	0.599149386954389	-
6986736	Mmp20	0.299	0.599149386954389	-
6989294	Pstpip1	0.299	0.599149386954389	-
6757049	Trpa1	0.3	0.599149386954389	-
6768082	Gcc2	0.3	0.599149386954389	-
6773158	Zufsp	0.3	0.599149386954389	-
6784239	Coasy	0.3	0.599149386954389	-
6785147	Nup85	0.3	0.599149386954389	-
6788822	Slc47a1	0.3	0.599149386954389	-
6792866	Wdr45l	0.3	0.599149386954389	-
6802386	Nek9	0.3	0.599149386954389	-
6804849	Heatr1	0.3	0.599149386954389	-
6812582	Ofcc1	0.3	0.599149386954389	-
6829687	Kcns2	0.3	0.599149386954389	-
6832923	Ano6	0.3	0.599149386954389	-
6837127	Sun2	0.3	0.599149386954389	-
6843198	Itsn1	0.3	0.599149386954389	-
6852225	Ttc27	0.3	0.599149386954389	-
6852676	Eml4	0.3	0.599149386954389	-
6854971	Angptl4	0.3	0.599149386954389	-
6855712	Rrp36	0.3	0.599149386954389	-
6895871	Ythdf3	0.3	0.599149386954389	-
6899334	Crtc2	0.3	0.599149386954389	-
6916263	Faf1	0.3	0.599149386954389	-
6927085	Per3	0.3	0.599149386954389	-
6933602	Mvk	0.3	0.599149386954389	-
6938698	Rfc1	0.3	0.599149386954389	-
6941866	Pitpnm2	0.3	0.599149386954389	-
6944371	Cav2	0.3	0.599149386954389	-
6958845	Bbc3	0.3	0.599149386954389	-
6964147	4930571K23Rik	0.3	0.599149386954389	-
6967971	Aldh1a3	0.3	0.599149386954389	-
6979124	Pdpr	0.3	0.599149386954389	-

6994133	Glb1l2	0.3	0.599149386954389	-
6996247	Map2k5	0.3	0.599149386954389	-
7015392	2010204K13Rik	0.3	0.599149386954389	-
6764049	Fcer1g	0.301	0.599149386954389	-
6775460	Fzr1	0.301	0.599149386954389	-
6779822	4930524B15Rik	0.301	0.599149386954389	-
6780430	Il12b	0.301	0.599149386954389	-
6792808	Stra13	0.301	0.599149386954389	-
6802315	Entpd5	0.301	0.599149386954389	-
6802349	Npc2	0.301	0.599149386954389	-
6826116	9030625A04Rik	0.301	0.599149386954389	-
6830638	Wdr67	0.301	0.599149386954389	-
6831640	Spatc1	0.301	0.599149386954389	-
6835692	Ext1	0.301	0.599149386954389	-
6838349	Senp1	0.301	0.599149386954389	-
6838750	Atf7	0.301	0.599149386954389	-
6852929	Klraq1	0.301	0.599149386954389	-
6854446	Rhbdl1	0.301	0.599149386954389	-
6866838	8030462N17Rik	0.301	0.599149386954389	-
6871166	Slc22a20	0.301	0.599149386954389	-
6890903	Cpxm1	0.301	0.599149386954389	-
6926896	Ptchd2	0.301	0.599149386954389	-
6929762	Depdc5	0.301	0.599149386954389	-
6937405	E130018O15Rik	0.301	0.599149386954389	-
6965614	Ccdc9	0.301	0.599149386954389	-
6966301	Upk1a	0.301	0.599149386954389	-
6966873	Mybpc2	0.301	0.599149386954389	-
6995120	Phldb1	0.301	0.599149386954389	-
6995800	Chrnb4	0.301	0.599149386954389	-
6773438	Gtf3c6	0.302	0.599149386954389	-
6784659	Ccdc45	0.302	0.599149386954389	-
6784940	Sox9	0.302	0.599149386954389	-
6806441	Pak1ip1	0.302	0.599149386954389	-
6819629	Kcnrg	0.302	0.599149386954389	-
6836596	Eif2c2	0.302	0.599149386954389	-
6838563	Csrnp2	0.302	0.599149386954389	-
6872761	Ankrd22	0.302	0.599149386954389	-
6875681	Entpd8	0.302	0.599149386954389	-
6898502	Fstl5	0.302	0.599149386954389	+
6903189	Fabp12	0.302	0.599149386954389	-
6914517	Frmd3	0.302	0.599149386954389	-
6917065	Rspo1	0.302	0.599149386954389	-
6921913	Ikbkap	0.302	0.599149386954389	-
6941199	Foxn4	0.302	0.599149386954389	-
6941637	Tpcn1	0.302	0.599149386954389	+
6942176	Psph	0.302	0.599149386954389	-
6950539	Ptpro	0.302	0.599149386954389	-
6951761	Gpr85	0.302	0.599149386954389	-
6962094	Tm6sf1	0.302	0.599149386954389	-
6966941	Aldh16a1	0.302	0.599149386954389	-

6968704	Rlbp1	0.302	0.599149386954389	-
6979675	Cpne7	0.302	0.599149386954389	-
6982695	Galnt7	0.302	0.599149386954389	-
6992487	Lrrfip2	0.302	0.599149386954389	-
6995757	Cib2	0.302	0.599149386954389	-
6748020	Gsta3	0.303	0.599149386954389	-
6755559	Efcab2	0.303	0.599149386954389	-
6756832	Cpa6	0.303	0.599149386954389	-
6774392	Hkdc1	0.303	0.599149386954389	-
6781516	B9d1	0.303	0.599149386954389	-
6782443	Tlcd2	0.303	0.599149386954389	-
6785227	Aanat	0.303	0.599149386954389	-
6788743	Atpaf2	0.303	0.599149386954389	-
6791333	Krt20	0.303	0.599149386954389	-
6795233	1110008L16Rik	0.303	0.599149386954389	-
6807265	Txndc15	0.303	0.599149386954389	-
6816714	Pxk	0.303	0.599149386954389	-
6817960	Pbrm1	0.303	0.599149386954389	-
6839650	Efcab1	0.303	0.599149386954389	-
6839685	Yars2	0.303	0.599149386954389	-
6857075	Smchd1	0.303	0.599149386954389	-
6869327	I830012O16Rik	0.303	0.599149386954389	-
6869691	Dntt	0.303	0.599149386954389	-
6877905	G6pc2	0.303	0.599149386954389	-
6879959	Slc5a12	0.303	0.599149386954389	-
6885429	B230208H17Rik	0.303	0.599149386954389	-
6885793	Asb6	0.303	0.599149386954389	-
6892286	Snta1	0.303	0.599149386954389	-
6901055	Sec24d	0.303	0.599149386954389	-
6919195	Acap3	0.303	0.599149386954389	-
6939991	Cxcl11	0.303	0.599149386954389	-
6945623	Adck2	0.303	0.599149386954389	-
6950674	Plekha5	0.303	0.599149386954389	-
6969890	Lrrc51	0.303	0.599149386954389	-
6971551	Ate1	0.303	0.599149386954389	-
6984473	Pllp	0.303	0.599149386954389	-
6994624	Hyls1	0.303	0.599149386954389	-
7012861	Med12	0.303	0.599149386954389	-
7018687	Zdhhc15	0.303	0.599149386954389	-
6755175	Pvrl4	0.304	0.599149386954389	-
6763259	Soat1	0.304	0.599149386954389	-
6772255	Gpr126	0.304	0.599149386954389	-
6778037	Esyt1	0.304	0.599149386954389	-
6784305	Cd300lg	0.304	0.599149386954389	-
6789475	Dhx33	0.304	0.599149386954389	-
6789808	Glod4	0.304	0.599149386954389	-
6791309	Tns4	0.304	0.599149386954389	-
6806162	Prpf4b	0.304	0.599149386954389	-
6808271	Erap1	0.304	0.599149386954389	-
6813474	Tifab	0.304	0.599149386954389	-

6818063	Msmb	0.304	0.599149386954389	-
6820573	Elf1	0.304	0.599149386954389	-
6840589	Muc4	0.304	0.599149386954389	-
6842273	Gbe1	0.304	0.599149386954389	-
6844411	Lamp3	0.304	0.599149386954389	-
6851273	Dus3l	0.304	0.599149386954389	-
6857431	Strn	0.304	0.599149386954389	-
6864604	Spata24	0.304	0.599149386954389	-
6867655	Pold4	0.304	0.599149386954389	-
6867978	B3gat3	0.304	0.599149386954389	-
6875702	Tprn	0.304	0.599149386954389	-
6881155	Tgm3	0.304	0.599149386954389	-
6884126	Lsm14b	0.304	0.599149386954389	-
6885525	Sardh	0.304	0.599149386954389	-
6892987	Cdh22	0.304	0.599149386954389	-
6897598	Sohlh2	0.304	0.599149386954389	-
6898037	Vmn2r1	0.304	0.599149386954389	-
6905620	Ssr3	0.304	0.599149386954389	-
6916220	Rab3b	0.304	0.599149386954389	-
6919021	Nphp4	0.304	0.599149386954389	-
6919741	Tmem67	0.304	0.599149386954389	-
6933390	Plcxd1	0.304	0.599149386954389	-
6933406	Golga3	0.304	0.599149386954389	-
6942941	AU022870	0.304	0.599149386954389	-
6957030	Phc1	0.304	0.599149386954389	-
6961099	Trpm1	0.304	0.599149386954389	-
6962043	Zfp592	0.304	0.599149386954389	-
6964244	Mapk3	0.304	0.599149386954389	-
6965379	Fgf3	0.304	0.599149386954389	-
6967014	Grin2d	0.304	0.599149386954389	-
6970442	Rnf141	0.304	0.599149386954389	-
6979421	Cenpn	0.304	0.599149386954389	-
6980565	Atp4b	0.304	0.599149386954389	-
6984536	Csnk2a2	0.304	0.599149386954389	-
6986737	Mmp7	0.304	0.599149386954389	-
6988216	BC024479	0.304	0.599149386954389	-
6992493	Dclk3	0.304	0.599149386954389	-
6750086	Pikfyve	0.305	0.599149386954389	-
6751520	Mlph	0.305	0.599149386954389	-
6752158	Serpinb10-ps	0.305	0.599149386954389	-
6759664	Igfbp5	0.305	0.599149386954389	+
6759764	Fev	0.305	0.599149386954389	-
6775762	Stab2	0.305	0.599149386954389	-
6783435	Coil	0.305	0.599149386954389	-
6786348	Meis1	0.305	0.599149386954389	-
6789445	Spag7	0.305	0.599149386954389	-
6799771	Tpo	0.305	0.599149386954389	-
6824961	N6amt2	0.305	0.599149386954389	-
6840123	Rtp1	0.305	0.599149386954389	-
6840735	Kpna1	0.305	0.599149386954389	-

6854930	Zfp101	0.305	0.599149386954389	-
6856266	Acer1	0.305	0.599149386954389	-
6860635	Eif1a	0.305	0.599149386954389	-
6861135	Gramd3	0.305	0.599149386954389	-
6879015	Rapsn	0.305	0.599149386954389	-
6879224	Alx4	0.305	0.599149386954389	-
6883737	Gnas	0.305	0.599149386954389	-
6890702	Zfp661	0.305	0.599149386954389	-
6899668	Lass2	0.305	0.599149386954389	-
6941049	Ulk1	0.305	0.599149386954389	-
6943862	Ppp1r9a	0.305	0.599149386954389	-
6956574	4931417G12Rik	0.305	0.599149386954389	-
6983193	Tmem59l	0.305	0.599149386954389	-
6984909	Cmtm4	0.305	0.599149386954389	-
6985925	Mvd	0.305	0.599149386954389	-
6989149	Exph5	0.305	0.599149386954389	-
6994956	Pou2f3	0.305	0.599149386954389	-
7012001	Taz	0.305	0.599149386954389	-
7012008	Fam50a	0.305	0.599149386954389	-
6757270	Pkhd1	0.306	0.599149386954389	-
6789329	Tnfsf13	0.306	0.599149386954389	-
6796422	Srsf5	0.306	0.599149386954389	-
6806294	Riok1	0.306	0.599149386954389	-
6807192	Tspan17	0.306	0.599149386954389	+
6812143	Gmds	0.306	0.599149386954389	-
6813326	Nop16	0.306	0.599149386954389	-
6820472	Epsti1	0.306	0.599149386954389	-
6831689	Gpt	0.306	0.599149386954389	-
6834558	Fam105b	0.306	0.599149386954389	-
6848203	Tmprss2	0.306	0.599149386954389	-
6856270	Khsrp	0.306	0.599149386954389	-
6882448	4833413D08Rik	0.306	0.599149386954389	-
6885868	Uck1	0.306	0.599149386954389	-
6891295	Pak7	0.306	0.599149386954389	+
6912030	Slc7a13	0.306	0.599149386954389	-
6918694	Mthfr	0.306	0.599149386954389	-
6921284	Exosc3	0.306	0.599149386954389	-
6930838	Pi4k2b	0.306	0.599149386954389	-
6937056	Slc5a6	0.306	0.599149386954389	-
6939338	Hopx	0.306	0.599149386954389	-
6941004	Gtpbp6	0.306	0.599149386954389	-
6942444	Ccl26	0.306	0.599149386954389	-
6942688	Ints1	0.306	0.599149386954389	-
6942709	Mad1l1	0.306	0.599149386954389	-
6957331	5930416I19Rik	0.306	0.599149386954389	-
6957432	Klra17	0.306	0.599149386954389	-
6995922	Csk	0.306	0.599149386954389	-
6996190	Paqr5	0.306	0.599149386954389	-
7015691	1810030O07Rik	0.306	0.599149386954389	-
6749471	Ormdl1	0.307	0.599149386954389	-

6771583	Zbtb39	0.307	0.599149386954389	-
6776156	Plxnc1	0.307	0.599149386954389	-
6778521	Aebp1	0.307	0.599149386954389	-
6787769	Itk	0.307	0.599149386954389	-
6792024	Cacng4	0.307	0.599149386954389	-
6794307	Sypl	0.307	0.599149386954389	-
6795279	Brms1l	0.307	0.599149386954389	-
6799206	Greb1	0.307	0.599149386954389	-
6802355	1110018G07Rik	0.307	0.599149386954389	-
6803821	Cdc42bpb	0.307	0.599149386954389	-
6828663	Capsl	0.307	0.599149386954389	-
6835429	Sybu	0.307	0.599149386954389	-
6838410	Rhebl1	0.307	0.599149386954389	-
6841136	Cd200r1	0.307	0.599149386954389	-
6843653	Coro7	0.307	0.599149386954389	-
6844000	Cpped1	0.307	0.599149386954389	-
6849386	Gng13	0.307	0.599149386954389	-
6867806	Slc25a45	0.307	0.599149386954389	-
6869305	Stambpl1	0.307	0.599149386954389	-
6873064	Cyp2c54	0.307	0.599149386954389	-
6874548	Dclre1c	0.307	0.599149386954389	-
6880852	Slc12a1	0.307	0.599149386954389	-
6881114	Ttl	0.307	0.599149386954389	-
6883098	Dbndd2	0.307	0.599149386954389	-
6887079	Cd302	0.307	0.599149386954389	-
6888751	Madd	0.307	0.599149386954389	-
6897691	Tm4sf4	0.307	0.599149386954389	-
6901957	Gbp2	0.307	0.599149386954389	-
6919159	Slc35e2	0.307	0.599149386954389	-
6920982	Aptx	0.307	0.599149386954389	-
6929858	A930005I04Rik	0.307	0.599149386954389	-
6933341	Fgfrl1	0.307	0.599149386954389	-
6937288	Mxd4	0.307	0.599149386954389	-
6941253	Unc119b	0.307	0.599149386954389	-
6947586	Add2	0.307	0.599149386954389	-
6950582	Mgst1	0.307	0.599149386954389	-
6954792	Ctnna2	0.307	0.599149386954389	-
6963120	Olfr628	0.307	0.599149386954389	-
6965944	Lipe	0.307	0.599149386954389	-
7002583	Tmem123	0.307	0.599149386954389	-
6782456	Inpp5k	0.308	0.599149386954389	-
6787293	Odz2	0.308	0.599149386954389	-
6789407	Alox12	0.308	0.599149386954389	-
6790966	Eme1	0.308	0.599149386954389	-
6801823	Ppp2r5e	0.308	0.599149386954389	-
6817763	Wnt5a	0.308	0.599149386954389	-
6820534	1300010F03Rik	0.308	0.599149386954389	-
6824166	Sftpd	0.308	0.599149386954389	-
6831852	Triobp	0.308	0.599149386954389	-
6833788	Egflam	0.308	0.599149386954389	-

6842085	Cggbp1	0.308	0.599149386954389	-
6855320	Crisp2	0.308	0.599149386954389	-
6855876	Daam2	0.308	0.599149386954389	-
6860127	Tmco6	0.308	0.599149386954389	-
6865022	Lars	0.308	0.599149386954389	-
6871490	Sdhaf2	0.308	0.599149386954389	-
6910085	Lmo4	0.308	0.599149386954389	-
6917512	Rcc1	0.308	0.599149386954389	-
6928464	Pex1	0.308	0.599149386954389	-
6937084	Fndc4	0.308	0.599149386954389	-
6945026	Tspan33	0.308	0.599149386954389	-
6947379	D6Mm5e	0.308	0.599149386954389	-
6949529	Kdm5a	0.308	0.599149386954389	-
6949838	Zfp384	0.308	0.599149386954389	-
6956864	Csgalnact2	0.308	0.599149386954389	-
6959130	Irgq	0.308	0.599149386954389	-
6961289	Fam169b	0.308	0.599149386954389	-
6969017	Fam108c	0.308	0.599149386954389	-
6970125	Fam160a2	0.308	0.599149386954389	-
6971268	Sh2b1	0.308	0.599149386954389	-
6973414	Pla2g4c	0.308	0.599149386954389	-
6976395	BC030500	0.308	0.599149386954389	+
6989622	Larp6	0.308	0.599149386954389	-
6989917	Parp16	0.308	0.599149386954389	-
6993848	Epor	0.308	0.599149386954389	-
7011848	Mtmr1	0.308	0.599149386954389	-
7017603	Naa10	0.308	0.599149386954389	-
6761139	Rnf152	0.309	0.599149386954389	-
6782294	Trpv3	0.309	0.599149386954389	-
6786586	Ehbp1	0.309	0.599149386954389	-
6788333	Il3	0.309	0.599149386954389	-
6813762	Cts6	0.309	0.599149386954389	-
6825442	Adam2	0.309	0.599149386954389	-
6825638	Slc25a37	0.309	0.599149386954389	-
6848707	Plg	0.309	0.599149386954389	-
6854658	Stk38	0.309	0.599149386954389	-
6870633	Vwa2	0.309	0.599149386954389	-
6870931	Lrp5	0.309	0.599149386954389	-
6870971	BC021614	0.309	0.599149386954389	-
6871298	Mark2	0.309	0.599149386954389	-
6876211	Ak1	0.309	0.599149386954389	-
6880587	Haus2	0.309	0.599149386954389	-
6906762	Rab25	0.309	0.599149386954389	-
6932215	Ambn	0.309	0.599149386954389	-
6943067	Rnf6	0.309	0.599149386954389	-
6957316	Tspan9	0.309	0.599149386954389	-
6963260	Nlrp14	0.309	0.599149386954389	-
6966338	Fxyd7	0.309	0.599149386954389	-
6753275	Adipor1	0.31	0.599149386954389	-
6764056	Ufc1	0.31	0.599149386954389	-

6768609	Ank3	0.31	0.599149386954389	-
6769481	Btbd11	0.31	0.599149386954389	-
6774433	Mypn	0.31	0.599149386954389	-
6792527	Ube2o	0.31	0.599149386954389	-
6792844	Tex19.2	0.31	0.599149386954389	-
6792970	Adcy3	0.31	0.599149386954389	-
6797577	Serpina3b	0.31	0.599149386954389	-
6802727	Dio2	0.31	0.599149386954389	+
6803319	Clmn	0.31	0.599149386954389	-
6835004	Snx31	0.31	0.599149386954389	-
6838564	Tcfcp2	0.31	0.599149386954389	-
6853951	Prdm9	0.31	0.599149386954389	-
6863281	Ccny	0.31	0.599149386954389	-
6869310	Fas	0.31	0.599149386954389	-
6870781	Kcnk18	0.31	0.599149386954389	-
6876567	Olfml2a	0.31	0.599149386954389	-
6878021	Dlx1	0.31	0.599149386954389	-
6929647	Mapre3	0.31	0.599149386954389	-
6933569	Ficd	0.31	0.599149386954389	-
6939721	Sult1b1	0.31	0.599149386954389	-
6948696	Gm9871	0.31	0.599149386954389	-
6958212	Casc1	0.31	0.599149386954389	-
6958983	Dmwd	0.31	0.599149386954389	-
6976645	Spock3	0.31	0.599149386954389	-
6983893	Rnaseh2a	0.31	0.599149386954389	-
6759326	Fam119a	0.311	0.599149386954389	-
6784205	Cnp	0.311	0.599149386954389	-
6785694	Gas2l1	0.311	0.599149386954389	-
6788507	Fam114a2	0.311	0.599149386954389	-
6791501	Vat1	0.311	0.599149386954389	-
6829952	Fzd6	0.311	0.599149386954389	-
6836296	Oc90	0.311	0.599149386954389	-
6849988	Zbtb22	0.311	0.599149386954389	-
6866231	Cplx4	0.311	0.599149386954389	-
6875649	Il1rn	0.311	0.599149386954389	-
6880931	Slc27a2	0.311	0.599149386954389	-
6889660	Bbox1	0.311	0.599149386954389	-
6889709	4930430A15Rik	0.311	0.599149386954389	-
6894218	Bhlhe23	0.311	0.599149386954389	-
6917970	Ece1	0.311	0.599149386954389	-
6919053	Lrrc47	0.311	0.599149386954389	-
6926872	Agtrap	0.311	0.599149386954389	-
6933474	Ttc28	0.311	0.599149386954389	-
6937585	Nsg1	0.311	0.599149386954389	-
6957119	Emg1	0.311	0.599149386954389	-
6959424	Lrfn1	0.311	0.599149386954389	-
6971287	Coro1a	0.311	0.599149386954389	-
6977766	Farsa	0.311	0.599149386954389	-
6978872	Pskh1	0.311	0.599149386954389	-
6979520	Crispld2	0.311	0.599149386954389	-

6985804	Mbtps1	0.311	0.599149386954389	-
6995063	Pdzd3	0.311	0.599149386954389	-
6997105	Ooep	0.311	0.599149386954389	-
7013015	Chic1	0.311	0.599149386954389	-
7014109	D330045A20Rik	0.311	0.599149386954389	-
6749702	Aox1	0.312	0.599149386954389	-
6777176	Tph2	0.312	0.599149386954389	-
6782959	Lig3	0.312	0.599149386954389	-
6788444	Nmur2	0.312	0.599149386954389	-
6788701	Flcn	0.312	0.599149386954389	-
6796000	Six6	0.312	0.599149386954389	-
6798795	Rhob	0.312	0.599149386954389	-
6803284	Serpina3c	0.312	0.599149386954389	-
6807258	Caml	0.312	0.599149386954389	-
6813887	Ctsl	0.312	0.599149386954389	-
6814347	Slc6a18	0.312	0.599149386954389	-
6819244	Cpne6	0.312	0.599149386954389	+
6825717	Xpo7	0.312	0.599149386954389	-
6832302	Parvg	0.312	0.599149386954389	-
6837604	Celsr1	0.312	0.599149386954389	-
6839744	Aifm3	0.312	0.599149386954389	-
6843207	Mrps6	0.312	0.599149386954389	-
6843663	5730403B10Rik	0.312	0.599149386954389	-
6845800	Gap43	0.312	0.599149386954389	-
6855327	Cenpq	0.312	0.599149386954389	-
6859832	Tslp	0.312	0.599149386954389	-
6861632	Sec11c	0.312	0.599149386954389	-
6879034	Acp2	0.312	0.599149386954389	-
6880219	BC052040	0.312	0.599149386954389	-
6885039	Dnajc1	0.312	0.599149386954389	+
6885409	Dpp7	0.312	0.599149386954389	-
6890638	Gabpb1	0.312	0.599149386954389	+
6896695	Sox2	0.312	0.599149386954389	-
6904301	Bbs7	0.312	0.599149386954389	-
6924874	Artn	0.312	0.599149386954389	-
6934506	Glt1d1	0.312	0.599149386954389	-
6937434	Acox3	0.312	0.599149386954389	-
6937548	Stk32b	0.312	0.599149386954389	-
6939667	Gnrhr	0.312	0.599149386954389	-
6941808	Vps33a	0.312	0.599149386954389	-
6957248	Kcna6	0.312	0.599149386954389	-
6957458	Klra2	0.312	0.599149386954389	-
6964345	Fbxl19	0.312	0.599149386954389	-
6965157	Pkp3	0.312	0.599149386954389	-
6966181	Actn4	0.312	0.599149386954389	-
6977151	Klf2	0.312	0.599149386954389	-
6978383	Ccdc113	0.312	0.599149386954389	-
6991090	Rwdd2a	0.312	0.599149386954389	+
6992408	Spink8	0.312	0.599149386954389	-
7009771	Foxp3	0.312	0.599149386954389	-

7015406	Magix	0.312	0.599149386954389	-
7015437	Glod5	0.312	0.599149386954389	-
6755222	Copa	0.313	0.599149386954389	-
6762304	Sox13	0.313	0.599149386954389	-
6764457	Adss	0.313	0.599149386954389	-
6768234	Sar1a	0.313	0.599149386954389	-
6769032	Dnmt3l	0.313	0.599149386954389	-
6775478	Tle6	0.313	0.599149386954389	-
6777955	Shmt2	0.313	0.599149386954389	-
6783840	Hoxb8	0.313	0.599149386954389	-
6789235	Arhgef15	0.313	0.599149386954389	-
6790359	Dhrs11	0.313	0.599149386954389	-
6790579	Lpo	0.313	0.599149386954389	-
6807812	Zfp759	0.313	0.599149386954389	+
6809047	Lhfpl2	0.313	0.599149386954389	+
6810782	Pfkp	0.313	0.599149386954389	-
6821821	Slitrk5	0.313	0.599149386954389	-
6839951	Ece2	0.313	0.599149386954389	-
6845569	Arhgap31	0.313	0.599149386954389	+
6853661	Qk	0.313	0.599149386954389	-
6855027	Btn3a3	0.313	0.599149386954389	-
6860118	Slc4a9	0.313	0.599149386954389	-
6867933	Hrasls5	0.313	0.599149386954389	-
6875832	Adamts13	0.313	0.599149386954389	-
6882894	Lpin3	0.313	0.599149386954389	-
6902684	Lrriq3	0.313	0.599149386954389	-
6906677	Prcc	0.313	0.599149386954389	-
6921929	Epb4.1l4b	0.313	0.599149386954389	-
6926017	Extl1	0.313	0.599149386954389	-
6937433	2310079F23Rik	0.313	0.599149386954389	-
6959807	Rhpn2	0.313	0.599149386954389	-
6966024	Egln2	0.313	0.599149386954389	-
6989152	Kdelc2	0.313	0.599149386954389	-
6995084	Ccdc84	0.313	0.599149386954389	-
7011033	Xpnpep2	0.313	0.599149386954389	-
7018510	Pdzd11	0.313	0.599149386954389	-
6754604	Vamp4	0.314	0.599149386954389	-
6768868	Ggt5	0.314	0.599149386954389	-
6771825	Oprm1	0.314	0.599149386954389	-
6782737	Nos2	0.314	0.599149386954389	-
6794552	Tmem195	0.314	0.599149386954389	-
6801454	Pole2	0.314	0.599149386954389	-
6813259	Msx2	0.314	0.599149386954389	-
6814157	Srd5a1	0.314	0.599149386954389	-
6839897	B3gnt5	0.314	0.599149386954389	-
6844598	Rfc4	0.314	0.599149386954389	-
6863929	Zfp191	0.314	0.599149386954389	-
6877932	Klhl23	0.314	0.599149386954389	-
6881028	Acoxl	0.314	0.599149386954389	-
6896759	Atp11b	0.314	0.599149386954389	-

6904047	Zmat3	0.314	0.599149386954389	-
6916557	Ipp	0.314	0.599149386954389	-
6924808	Mmachc	0.314	0.599149386954389	-
6941813	Clip1	0.314	0.599149386954389	-
6953810	Inmt	0.314	0.599149386954389	-
6974480	Mcph1	0.314	0.599149386954389	-
6977756	Trmt1	0.314	0.599149386954389	-
6989912	Igdcc4	0.314	0.599149386954389	-
7018329	Hsf3	0.314	0.599149386954389	-
6755179	F11r	0.315	0.599149386954389	-
6755562	Kif26b	0.315	0.599149386954389	+
6758941	Clk1	0.315	0.599149386954389	-
6772810	Enpp3	0.315	0.599149386954389	-
6780754	Scgb3a1	0.315	0.599149386954389	-
6783056	Ddx52	0.315	0.599149386954389	-
6784558	Tlk2	0.315	0.599149386954389	-
6784785	Amz2	0.315	0.599149386954389	-
6790264	Pex12	0.315	0.599149386954389	-
6799852	Dus4l	0.315	0.599149386954389	+
6800917	Ppp2r3c	0.315	0.599149386954389	-
6823116	Synpo2l	0.315	0.599149386954389	-
6827925	Dock9	0.315	0.599149386954389	-
6836874	Zfp251	0.315	0.599149386954389	-
6839624	Snai2	0.315	0.599149386954389	-
6839751	P2rx6	0.315	0.599149386954389	-
6839839	Txnrd2	0.315	0.599149386954389	-
6840612	Osbpl11	0.315	0.599149386954389	-
6854690	Ppil1	0.315	0.599149386954389	-
6857809	Abcg5	0.315	0.599149386954389	-
6867830	Cdca5	0.315	0.599149386954389	-
6875834	5930434B04Rik	0.315	0.599149386954389	-
6876430	Ptgs1	0.315	0.599149386954389	-
6899366	S100a3	0.315	0.599149386954389	-
6903157	Pag1	0.315	0.599149386954389	-
6910281	Dnase2b	0.315	0.599149386954389	-
6923391	Plaa	0.315	0.599149386954389	-
6932822	--	0.315	0.599149386954389	-
6934897	Mlxipl	0.315	0.599149386954389	-
6942960	Baiap2l1	0.315	0.599149386954389	-
6967048	Sergef	0.315	0.599149386954389	-
6969640	Capn5	0.315	0.599149386954389	-
6972118	Echs1	0.315	0.599149386954389	-
6980016	Nrp1	0.315	0.599149386954389	+
6983158	9130404D08Rik	0.315	0.599149386954389	-
6985808	Hsdl1	0.315	0.599149386954389	-
7015521	Cybb	0.315	0.599149386954389	-
6756334	Angel2	0.316	0.599149386954389	-
6764011	Uap1	0.316	0.599149386954389	-
6766422	Hbs1l	0.316	0.599149386954389	-
6790288	Ccl5	0.316	0.599149386954389	-

6792506	Exoc7	0.316	0.599149386954389	-
6794219	Slc26a3	0.316	0.599149386954389	-
6795093	Npas3	0.316	0.599149386954389	-
6796696	Jdp2	0.316	0.599149386954389	+
6811488	Pgbd1	0.316	0.599149386954389	-
6813877	Zfp367	0.316	0.599149386954389	-
6824728	Slc7a7	0.316	0.599149386954389	-
6828016	Nalcn	0.316	0.599149386954389	-
6837812	Tymp	0.316	0.599149386954389	-
6839502	Mkl2	0.316	0.599149386954389	-
6840805	Rabl3	0.316	0.599149386954389	-
6842682	Ncam2	0.316	0.599149386954389	-
6844187	2310008H04Rik	0.316	0.599149386954389	-
6847690	Krtap26-1	0.316	0.599149386954389	-
6850729	Pex6	0.316	0.599149386954389	-
6854458	Solh	0.316	0.599149386954389	-
6866631	Smad4	0.316	0.599149386954389	-
6867840	Batf2	0.316	0.599149386954389	-
6869951	Hif1an	0.316	0.599149386954389	-
6880899	Galk2	0.316	0.599149386954389	-
6905660	Veph1	0.316	0.599149386954389	-
6922241	Alad	0.316	0.599149386954389	-
6928871	Sema3a	0.316	0.599149386954389	-
6936981	Tyms	0.316	0.599149386954389	-
6949092	Il17re	0.316	0.599149386954389	-
6952294	Pax4	0.316	0.599149386954389	-
6956712	Timp4	0.316	0.599149386954389	-
6960332	Nosip	0.316	0.599149386954389	-
6963045	Pgap2	0.316	0.599149386954389	-
6984921	Ccdc79	0.316	0.599149386954389	-
6985813	Kcng4	0.316	0.599149386954389	-
6989440	Cyp11a1	0.316	0.599149386954389	-
6992274	Mst1r	0.316	0.599149386954389	-
6993102	Tgm4	0.316	0.599149386954389	-
7014815	Rps6ka3	0.316	0.599149386954389	+
6767385	Mical1	0.317	0.599149386954389	-
6785114	Rab37	0.317	0.599149386954389	-
6791069	B4galnt2	0.317	0.599149386954389	-
6796415	4933426M11Rik	0.317	0.599149386954389	-
6803219	Serpina6	0.317	0.599149386954389	-
6829647	Mtdh	0.317	0.599149386954389	-
6832005	Grap2	0.317	0.599149386954389	-
6832115	Ep300	0.317	0.599149386954389	-
6840775	Iqcb1	0.317	0.599149386954389	-
6850012	H2-DMa	0.317	0.599149386954389	-
6864520	Brd8	0.317	0.599149386954389	-
6890664	1810024B03Rik	0.317	0.599149386954389	-
6899028	Hdgf	0.317	0.599149386954389	-
6900993	Dnttip2	0.317	0.599149386954389	-
6906104	Golim4	0.317	0.599149386954389	-

6906703	Ttc24	0.317	0.599149386954389	-
6917198	AU040320	0.317	0.599149386954389	-
6944574	Ing3	0.317	0.599149386954389	-
6946558	Herc3	0.317	0.599149386954389	+
6971606	2310057M21Rik	0.317	0.599149386954389	-
6972115	1810014F10Rik	0.317	0.599149386954389	-
6998487	Nek11	0.317	0.599149386954389	-
7015006	Pir	0.317	0.599149386954389	+
6751430	Sh3bp4	0.318	0.599149386954389	-
6752409	Mki67ip	0.318	0.599149386954389	-
6754227	Acbd6	0.318	0.599149386954389	-
6754274	Nphs2	0.318	0.599149386954389	-
6758995	Als2	0.318	0.599149386954389	-
6763146	Npl	0.318	0.599149386954389	-
6789941	Nek8	0.318	0.599149386954389	-
6791406	Krt19	0.318	0.599149386954389	-
6792953	Pomc	0.318	0.599149386954389	-
6796538	Rbm25	0.318	0.599149386954389	-
6831648	Gpaa1	0.318	0.599149386954389	-
6899558	Them5	0.318	0.599149386954389	-
6906818	Trim46	0.318	0.599149386954389	-
6909629	Cisd2	0.318	0.599149386954389	-
6911537	Chd7	0.318	0.599149386954389	-
6921154	Cd72	0.318	0.599149386954389	-
6926225	Alpl	0.318	0.599149386954389	-
6931845	1700023E05Rik	0.318	0.599149386954389	-
6942655	Prkar1b	0.318	0.599149386954389	-
6944952	Snd1	0.318	0.599149386954389	-
6947985	Hdac11	0.318	0.599149386954389	-
6953395	Rarres2	0.318	0.599149386954389	-
6965153	B4galnt4	0.318	0.599149386954389	-
6966808	Cd33	0.318	0.599149386954389	-
6984338	Ces1c	0.318	0.599149386954389	-
6987422	Prkcsh	0.318	0.599149386954389	-
6992218	Zmynd10	0.318	0.599149386954389	-
6994353	St14	0.318	0.599149386954389	-
7010183	Maoa	0.318	0.599149386954389	-
7015001	Tmem27	0.318	0.599149386954389	-
6777906	Os9	0.319	0.599149386954389	-
6782411	Tsr1	0.319	0.599149386954389	-
6790294	Ccl3	0.319	0.599149386954389	-
6791204	Srcin1	0.319	0.599149386954389	-
6791504	Brca1	0.319	0.599149386954389	-
6835001	Ankrd46	0.319	0.599149386954389	-
6847174	Lipi	0.319	0.599149386954389	-
6854467	Arhgdig	0.319	0.599149386954389	-
6875191	Cacnb2	0.319	0.599149386954389	-
6907224	Car14	0.319	0.599149386954389	-
6924583	Slc5a9	0.319	0.599149386954389	-
6933675	Pxn	0.319	0.599149386954389	-

6945011	Irf5	0.319	0.599149386954389	-
6946993	Capg	0.319	0.599149386954389	-
6951282	Hepacam2	0.319	0.599149386954389	-
6951412	Pdk4	0.319	0.599149386954389	-
6951975	Asz1	0.319	0.599149386954389	-
6959273	Cyp2b19	0.319	0.599149386954389	-
6959279	Cyp2f2	0.319	0.599149386954389	-
6960166	Ctu1	0.319	0.599149386954389	-
6963916	Abca16	0.319	0.599149386954389	-
6977091	Fam129c	0.319	0.599149386954389	-
6992178	Rrp9	0.319	0.599149386954389	-
6998076	Rasa2	0.319	0.599149386954389	-
7014268	Pak3	0.319	0.599149386954389	-
6748213	Lmbrd1	0.32	0.599149386954389	-
6763014	Rnf2	0.32	0.599149386954389	-
6773629	Qrsl1	0.32	0.599149386954389	-
6785104	Gprc5c	0.32	0.599149386954389	-
6789369	Rai12	0.32	0.599149386954389	-
6789721	Dph1	0.32	0.599149386954389	-
6796043	Prkch	0.32	0.599149386954389	-
6796193	Plekhg3	0.32	0.599149386954389	-
6797978	Dlk1	0.32	0.599149386954389	-
6803862	Xrcc3	0.32	0.599149386954389	-
6815687	Erbb2ip	0.32	0.599149386954389	-
6824834	Nedd8	0.32	0.599149386954389	-
6833227	Tmprss12	0.32	0.599149386954389	-
6833302	Ankrd33	0.32	0.599149386954389	-
6833937	Il7r	0.32	0.599149386954389	-
6840637	Itgb5	0.32	0.599149386954389	-
6847824	4932438H23Rik	0.32	0.599149386954389	-
6849288	Dci	0.32	0.599149386954389	-
6855013	Psmb9	0.32	0.599149386954389	-
6856201	Plin4	0.32	0.599149386954389	-
6860133	Hars2	0.32	0.599149386954389	-
6875744	Lcn5	0.32	0.599149386954389	-
6876188	Ptges2	0.32	0.599149386954389	-
6877909	Dhrs9	0.32	0.599149386954389	-
6879682	Ccdc73	0.32	0.599149386954389	-
6900100	Trim33	0.32	0.599149386954389	-
6917217	Dlgap3	0.32	0.599149386954389	-
6947763	Gata2	0.32	0.599149386954389	-
6949094	Il17rc	0.32	0.599149386954389	-
6958982	Rsph6a	0.32	0.599149386954389	-
6964036	Prkcb	0.32	0.599149386954389	-
6965980	Hnrnpul1	0.32	0.599149386954389	-
6966448	Kctd15	0.32	0.599149386954389	-
6970393	Dennd5a	0.32	0.599149386954389	-
6970829	Syt17	0.32	0.599149386954389	-
6976982	Tmem161a	0.32	0.599149386954389	-
6979821	Capn9	0.32	0.599149386954389	-

6984336	Ces1a	0.32	0.599149386954389	-
6986781	Pgr	0.32	0.599149386954389	-
6748699	Inpp4a	0.321	0.599149386954389	-
6763787	Rcsd1	0.321	0.599149386954389	-
6764833	Mosc1	0.321	0.599149386954389	-
6764992	Spata17	0.321	0.599149386954389	-
6788649	Obscn	0.321	0.599149386954389	-
6789908	Pipox	0.321	0.599149386954389	-
6796492	Sipa1l1	0.321	0.599149386954389	-
6838327	Rpap3	0.321	0.599149386954389	-
6838682	Krt1	0.321	0.599149386954389	-
6844447	Abcc5	0.321	0.599149386954389	-
6857106	Alk	0.321	0.599149386954389	-
6860476	Stk32a	0.321	0.599149386954389	-
6875643	Il1f10	0.321	0.599149386954389	-
6883210	Ncoa3	0.321	0.599149386954389	-
6885909	Ttc16	0.321	0.599149386954389	-
6929817	Fgfr3	0.321	0.599149386954389	-
6946979	Ggcx	0.321	0.599149386954389	-
6949105	Vhl	0.321	0.599149386954389	-
6949716	Foxj2	0.321	0.599149386954389	-
6956678	Vgll4	0.321	0.599149386954389	-
6965093	Cd163l1	0.321	0.599149386954389	-
6966293	BC053749	0.321	0.599149386954389	-
6968712	Rhcg	0.321	0.599149386954389	-
6970370	Trim66	0.321	0.599149386954389	-
6971323	Qprt	0.321	0.599149386954389	-
6989861	Lctl	0.321	0.599149386954389	-
6751645	Capn10	0.322	0.599149386954389	-
6764120	Nhlh1	0.322	0.599149386954389	-
6785534	Hexdc	0.322	0.599149386954389	-
6789358	Kctd11	0.322	0.599149386954389	-
6792547	St6galnac1	0.322	0.599149386954389	-
6794766	Nrcam	0.322	0.599149386954389	-
6801681	Six1	0.322	0.599149386954389	-
6824206	Ero1l	0.322	0.599149386954389	-
6831647	Exosc4	0.322	0.599149386954389	-
6833216	Larp4	0.322	0.599149386954389	-
6839102	Alg1	0.322	0.599149386954389	-
6841739	Tomm70a	0.322	0.599149386954389	-
6845159	Fbxo45	0.322	0.599149386954389	-
6850678	Mrps18a	0.322	0.599149386954389	-
6868105	Ms4a6b	0.322	0.599149386954389	-
6870102	2010012O05Rik	0.322	0.599149386954389	-
6877139	Fmnl2	0.322	0.599149386954389	-
6877931	Phospho2	0.322	0.599149386954389	-
6880492	Casc5	0.322	0.599149386954389	-
6921036	1110017D15Rik	0.322	0.599149386954389	-
6922302	Whrn	0.322	0.599149386954389	-
6939671	Tmprss11d	0.322	0.599149386954389	-

6939868	Ankrd17	0.322	0.599149386954389	-
6950334	Etv6	0.322	0.599149386954389	-
6951180	Far2	0.322	0.599149386954389	-
6955981	Shq1	0.322	0.599149386954389	-
6959452	Sirt2	0.322	0.599149386954389	-
6960500	Ldhc	0.322	0.599149386954389	-
6964287	Zfp771	0.322	0.599149386954389	-
6966042	Spnb4	0.322	0.599149386954389	-
6976987	Homer3	0.322	0.599149386954389	-
6980964	Polb	0.322	0.599149386954389	-
6981342	Chrna6	0.322	0.599149386954389	-
6985799	Slc38a8	0.322	0.599149386954389	-
6992898	Wdr48	0.322	0.599149386954389	-
6995878	Neil1	0.322	0.599149386954389	-
6996792	Pigb	0.322	0.599149386954389	-
6763752	Sft2d2	0.323	0.599149386954389	-
6779718	Mtif2	0.323	0.599149386954389	-
6784256	Cntd1	0.323	0.599149386954389	-
6823122	Ndst2	0.323	0.599149386954389	-
6844373	Igl-C2	0.323	0.599149386954389	-
6847878	Dnajc28	0.323	0.599149386954389	+
6854400	Mapk8ip3	0.323	0.599149386954389	-
6857501	Atl2	0.323	0.599149386954389	-
6878702	Ypel4	0.323	0.599149386954389	+
6885447	Ubac1	0.323	0.599149386954389	-
6886002	Gapvd1	0.323	0.599149386954389	-
6907426	Hsd3b3	0.323	0.599149386954389	-
6919012	Acot7	0.323	0.599149386954389	-
6921162	Tln1	0.323	0.599149386954389	-
6922924	Psip1	0.323	0.599149386954389	-
6926076	Srrm1	0.323	0.599149386954389	-
6929907	Plk1	0.323	0.599149386954389	-
6934187	Orai1	0.323	0.599149386954389	-
6942494	Sh2b2	0.323	0.599149386954389	-
6945676	Ssbp1	0.323	0.599149386954389	-
6970594	Gm5600	0.323	0.599149386954389	-
6978781	Ces2c	0.323	0.599149386954389	-
6983097	Slc18a1	0.323	0.599149386954389	-
6990525	BC031353	0.323	0.599149386954389	-
6750566	Cyp27a1	0.324	0.599149386954389	-
6752571	Ccdc93	0.324	0.599149386954389	-
6753084	Rab7l1	0.324	0.599149386954389	-
6755387	Exo1	0.324	0.599149386954389	-
6760774	Pask	0.324	0.599149386954389	-
6763144	Dhx9	0.324	0.599149386954389	-
6763622	Fmo4	0.324	0.599149386954389	-
6769917	Fgd6	0.324	0.599149386954389	-
6771927	Iyd	0.324	0.599149386954389	-
6775159	Smarcb1	0.324	0.599149386954389	-
6778315	Morc2a	0.324	0.599149386954389	-

6782498	Rnmtl1	0.324	0.599149386954389	-
6788120	Hnrnpab	0.324	0.599149386954389	-
6789197	Ntn1	0.324	0.599149386954389	-
6789964	Slc13a2	0.324	0.599149386954389	-
6791883	Strada	0.324	0.599149386954389	-
6792421	Fads6	0.324	0.599149386954389	-
6802749	Sel1l	0.324	0.599149386954389	-
6803268	Serpina11	0.324	0.599149386954389	-
6807345	Smad5	0.324	0.599149386954389	-
6808638	Tmem161b	0.324	0.599149386954389	-
6823653	Il17rb	0.324	0.599149386954389	-
6824266	Bmp4	0.324	0.599149386954389	-
6827924	Slc15a1	0.324	0.599149386954389	-
6828005	Tmtc4	0.324	0.599149386954389	-
6844184	Mzt2	0.324	0.599149386954389	-
6850235	H2-M11	0.324	0.599149386954389	-
6875181	Mrc1	0.324	0.599149386954389	-
6876279	--	0.324	0.599149386954389	-
6892929	Spinlw1	0.324	0.599149386954389	-
6902661	Cryz	0.324	0.599149386954389	-
6921557	Baat	0.324	0.599149386954389	-
6929573	Ube3c	0.324	0.599149386954389	-
6949095	Creld1	0.324	0.599149386954389	-
6955272	Aplf	0.324	0.599149386954389	-
6971746	Uros	0.324	0.599149386954389	-
6972405	Cdkn1c	0.324	0.599149386954389	-
6975889	4933411K20Rik	0.324	0.599149386954389	-
6977995	Nod2	0.324	0.599149386954389	-
6994935	Sc5d	0.324	0.599149386954389	+
6751264	Psmd1	0.325	0.599149386954389	-
6777251	Cnot2	0.325	0.599149386954389	-
6788393	Ccdc69	0.325	0.599149386954389	-
6791520	Meox1	0.325	0.599149386954389	-
6813667	4921517D22Rik	0.325	0.599149386954389	-
6814128	Adcy2	0.325	0.599149386954389	-
6819257	Rnf31	0.325	0.599149386954389	-
6824507	3632451O06Rik	0.325	0.599149386954389	+
6832587	Shank3	0.325	0.599149386954389	-
6836560	Fam135b	0.325	0.599149386954389	+
6850000	Rxrb	0.325	0.599149386954389	-
6850064	Atf6b	0.325	0.599149386954389	-
6855149	Abcf1	0.325	0.599149386954389	-
6858842	Gata6	0.325	0.599149386954389	-
6861173	Megf10	0.325	0.599149386954389	-
6875582	Gad2	0.325	0.599149386954389	-
6875680	Nelf	0.325	0.599149386954389	-
6885923	Slc2a8	0.325	0.599149386954389	-
6891648	Rrbp1	0.325	0.599149386954389	-
6896557	Kcnmb2	0.325	0.599149386954389	-
6900095	Dennd2c	0.325	0.599149386954389	-

6917524	Rpa2	0.325	0.599149386954389	-
6936585	Dnajc2	0.325	0.599149386954389	-
6939185	Lnx1	0.325	0.599149386954389	-
6948759	Chl1	0.325	0.599149386954389	-
6954541	1700011F03Rik	0.325	0.599149386954389	-
6955046	Clec4f	0.325	0.599149386954389	-
6960445	Otog	0.325	0.599149386954389	-
6979670	Spg7	0.325	0.599149386954389	-
6989373	Trcg1	0.325	0.599149386954389	-
7015648	Bcor	0.325	0.599149386954389	-
7018546	Zmym3	0.325	0.599149386954389	-
7019867	Acsl4	0.325	0.599149386954389	+
6749704	Aox3	0.326	0.599149386954389	-
6756597	Rp1	0.326	0.599149386954389	-
6775576	Appl2	0.326	0.599149386954389	-
6786046	Grb10	0.326	0.599149386954389	-
6789409	Alox12e	0.326	0.599149386954389	-
6831681	Adck5	0.326	0.599149386954389	-
6832142	Aco2	0.326	0.599149386954389	-
6837096	Pla2g6	0.326	0.599149386954389	-
6848859	Tbp	0.326	0.599149386954389	-
6871505	Pga5	0.326	0.599149386954389	-
6871517	Zp1	0.326	0.599149386954389	-
6912883	Spink4	0.326	0.599149386954389	-
6913863	Dnajc25	0.326	0.599149386954389	-
6927117	Zbtb48	0.326	0.599149386954389	-
6928479	Krit1	0.326	0.599149386954389	-
6943126	Lnx2	0.326	0.599149386954389	-
6953139	Eapa2	0.326	0.599149386954389	-
6969672	2210018M11Rik	0.326	0.599149386954389	-
6979320	Wwox	0.326	0.599149386954389	-
6984484	Ciapin1	0.326	0.599149386954389	+
6988364	Zfp202	0.326	0.599149386954389	-
6992189	Vprbp	0.326	0.599149386954389	-
6992855	Itga9	0.326	0.599149386954389	-
6995076	Vps11	0.326	0.599149386954389	-
7009775	Prickle3	0.326	0.599149386954389	-
6786463	Actr2	0.327	0.599149386954389	-
6802041	Rdh11	0.327	0.599149386954389	-
6830165	Ebag9	0.327	0.599149386954389	-
6851309	Tnfsf9	0.327	0.599149386954389	-
6854335	Amdhd2	0.327	0.599149386954389	-
6871002	Rhod	0.327	0.599149386954389	-
6876052	Phyhd1	0.327	0.599149386954389	-
6921432	Alg2	0.327	0.599149386954389	-
6921493	Tex10	0.327	0.599149386954389	-
6938259	Cckar	0.327	0.599149386954389	-
6960390	Mamstr	0.327	0.599149386954389	-
6965314	Tspan32	0.327	0.599149386954389	-
6965950	Ceacam2	0.327	0.599149386954389	-

6967011	Cyth2	0.327	0.599149386954389	-
6973737	Snapc2	0.327	0.599149386954389	-
6998669	Bsn	0.327	0.599149386954389	-
7014085	Nrk	0.327	0.599149386954389	-
7016078	Klhl13	0.327	0.599149386954389	-
6760789	Stk25	0.328	0.599149386954389	-
6771912	Akap12	0.328	0.599149386954389	-
6775236	Trpm2	0.328	0.599149386954389	-
6785168	Llgl2	0.328	0.599149386954389	-
6787190	Ranbp17	0.328	0.599149386954389	-
6788301	Slc22a5	0.328	0.599149386954389	-
6792868	Rab40b	0.328	0.599149386954389	-
6839928	Eif2b5	0.328	0.599149386954389	-
6849315	Rpl3l	0.328	0.599149386954389	-
6849481	Itpr3	0.328	0.599149386954389	-
6861774	Rnmt	0.328	0.599149386954389	-
6870770	Eno4	0.328	0.599149386954389	-
6877584	Tank	0.328	0.599149386954389	-
6880983	Stard7	0.328	0.599149386954389	-
6886356	Zeb2	0.328	0.599149386954389	-
6886730	Arl5a	0.328	0.599149386954389	-
6901380	Lef1	0.328	0.599149386954389	-
6917979	Kif17	0.328	0.599149386954389	-
6924454	4930522H14Rik	0.328	0.599149386954389	-
6939966	Cdkl2	0.328	0.599149386954389	-
6940236	Antxr2	0.328	0.599149386954389	-
6957178	Plekhg6	0.328	0.599149386954389	-
6958043	Slco1a6	0.328	0.599149386954389	-
6970916	Dcun1d3	0.328	0.599149386954389	-
6972256	2700078K21Rik	0.328	0.599149386954389	-
6974663	Sfrp1	0.328	0.599149386954389	-
6986692	Dcun1d5	0.328	0.599149386954389	-
6994778	Olfr947-ps1	0.328	0.599149386954389	-
6768324	Dna2	0.329	0.599149386954389	-
6779855	Stk10	0.329	0.599149386954389	-
6782812	Rhot1	0.329	0.599149386954389	-
6783330	Rnf43	0.329	0.599149386954389	-
6789161	Glp2r	0.329	0.599149386954389	-
6789338	Polr2a	0.329	0.599149386954389	-
6789875	Taok1	0.329	0.599149386954389	-
6801560	Timm9	0.329	0.599149386954389	-
6802313	Fam161b	0.329	0.599149386954389	-
6825544	Cdca2	0.329	0.599149386954389	-
6831564	BC025446	0.329	0.599149386954389	-
6836830	Scrt1	0.329	0.599149386954389	-
6843916	Prm2	0.329	0.599149386954389	-
6848972	Fpr2	0.329	0.599149386954389	-
6854616	Srpk1	0.329	0.599149386954389	-
6855603	Cdc5l	0.329	0.599149386954389	-
6869979	Sfxn3	0.329	0.599149386954389	-

6871511	Cd6	0.329	0.599149386954389	-
6896503	Lrriq4	0.329	0.599149386954389	-
6899146	Ssr2	0.329	0.599149386954389	-
6906742	Pmf1	0.329	0.599149386954389	-
6914934	4930473A06Rik	0.329	0.599149386954389	-
6934311	Ddx55	0.329	0.599149386954389	-
6941103	E130006D01Rik	0.329	0.599149386954389	-
6951752	Tmem168	0.329	0.599149386954389	-
6968778	Man2a2	0.329	0.599149386954389	-
6969277	Ccdc83	0.329	0.599149386954389	-
6970750	Pik3c2a	0.329	0.599149386954389	-
6971303	Ino80e	0.329	0.599149386954389	-
6976955	Gmip	0.329	0.599149386954389	-
6976959	Pbx4	0.329	0.599149386954389	-
6979318	Vat1l	0.329	0.599149386954389	-
6983897	Best2	0.329	0.599149386954389	-
6992209	Cish	0.329	0.599149386954389	-
6992333	Usp4	0.329	0.599149386954389	-
6999249	Tgfbr2	0.329	0.599149386954389	-
6999412	Slc22a13	0.329	0.599149386954389	-
7014142	Frmpd3	0.329	0.599149386954389	-
6753157	Lrrn2	0.33	0.599149386954389	-
6769272	Matk	0.33	0.599149386954389	-
6783586	Car10	0.33	0.599149386954389	-
6791048	Ngfr	0.33	0.599149386954389	-
6792638	Cyth1	0.33	0.599149386954389	-
6793325	Nt5c1b	0.33	0.599149386954389	-
6813407	Dok3	0.33	0.599149386954389	-
6820896	Tdrd3	0.33	0.599149386954389	-
6827159	Ednrb	0.33	0.599149386954389	-
6840823	Gpr156	0.33	0.599149386954389	-
6847928	Atp5o	0.33	0.599149386954389	-
6854694	Mtch1	0.33	0.599149386954389	-
6855478	Slc25a27	0.33	0.599149386954389	-
6859285	Dsg2	0.33	0.599149386954389	-
6861350	Camk2a	0.33	0.599149386954389	-
6874639	Ucma	0.33	0.599149386954389	-
6876043	Tbc1d13	0.33	0.599149386954389	-
6880508	Spint1	0.33	0.599149386954389	-
6906042	Slitrk3	0.33	0.599149386954389	-
6916849	Col9a2	0.33	0.599149386954389	-
6922901	Ttc39b	0.33	0.599149386954389	-
6936589	Reln	0.33	0.599149386954389	-
6937333	Mfsd10	0.33	0.599149386954389	-
6966983	Ruvbl2	0.33	0.599149386954389	-
6968856	3110040N11Rik	0.33	0.599149386954389	-
6984966	Fhod1	0.33	0.599149386954389	-
6987954	Ets1	0.33	0.599149386954389	-
6990678	Gcm1	0.33	0.599149386954389	-
7015458	Tbc1d25	0.33	0.599149386954389	-

6753189	Golt1a	0.331	0.599149386954389	-
6775335	Polr2e	0.331	0.599149386954389	-
6775367	Adamtsl5	0.331	0.599149386954389	-
6803088	BC002230	0.331	0.599149386954389	-
6812518	Eef1e1	0.331	0.599149386954389	-
6817217	Thrb	0.331	0.599149386954389	-
6824744	4931414P19Rik	0.331	0.599149386954389	-
6833220	Dip2b	0.331	0.599149386954389	-
6852542	Tmem178	0.331	0.599149386954389	-
6876136	Hmcn2	0.331	0.599149386954389	-
6884277	Tpd52l2	0.331	0.599149386954389	-
6890355	Mfap1b	0.331	0.599149386954389	-
6897577	Alg5	0.331	0.599149386954389	-
6900116	Syt6	0.331	0.599149386954389	-
6915871	Sgip1	0.331	0.599149386954389	-
6920260	1810074P20Rik	0.331	0.599149386954389	-
6921381	Coro2a	0.331	0.599149386954389	-
6926912	Angptl7	0.331	0.599149386954389	-
6933073	Pkd2	0.331	0.599149386954389	-
6954626	Mat2a	0.331	0.599149386954389	-
6958439	Dennd5b	0.331	0.599149386954389	-
6959116	Kcnn4	0.331	0.599149386954389	-
6964241	Bola2	0.331	0.599149386954389	-
6985991	Fanca	0.331	0.599149386954389	-
6990390	Zfp280d	0.331	0.599149386954389	-
7007900	Eif4h	0.331	0.599149386954389	-
7009815	Gm6787	0.331	0.599149386954389	-
7018585	Cited1	0.331	0.599149386954389	-
6764205	Fcer1a	0.332	0.599149386954389	-
6783176	Ppm1d	0.332	0.599149386954389	-
6784034	Gsdma3	0.332	0.599149386954389	-
6792691	Tbc1d16	0.332	0.599149386954389	-
6800948	Ralgapa1	0.332	0.599149386954389	-
6805580	Prl3b1	0.332	0.599149386954389	-
6813536	Spock1	0.332	0.599149386954389	-
6837818	Cpt1b	0.332	0.599149386954389	-
6847559	Adamts5	0.332	0.599149386954389	-
6852902	Msh6	0.332	0.599149386954389	-
6866955	Nfatc1	0.332	0.599149386954389	-
6869324	Ifit2	0.332	0.599149386954389	-
6875611	Nxph2	0.332	0.599149386954389	-
6882252	Angpt4	0.332	0.599149386954389	-
6892403	Gdf5	0.332	0.599149386954389	-
6907100	Tuft1	0.332	0.599149386954389	-
6923149	C87499	0.332	0.599149386954389	-
6927112	Phf13	0.332	0.599149386954389	-
6929301	Slc4a2	0.332	0.599149386954389	-
6933157	Zfp326	0.332	0.599149386954389	-
6934119	Hvcn1	0.332	0.599149386954389	-
6934273	Ogfod2	0.332	0.599149386954389	-

6943476	Rbm47	0.332	0.599149386954389	-
6965877	Tex101	0.332	0.599149386954389	-
6969940	Trim21	0.332	0.599149386954389	-
6975900	Acsl1	0.332	0.599149386954389	-
6976978	Rfxank	0.332	0.599149386954389	-
6978784	Ces2e	0.332	0.599149386954389	-
6998753	Fbxw20	0.332	0.599149386954389	-
7012872	Itgb1bp2	0.332	0.599149386954389	-
6757322	Gm4956	0.333	0.599149386954389	-
6760251	Sphkap	0.333	0.599149386954389	+
6760410	Alppl2	0.333	0.599149386954389	-
6769246	Amh	0.333	0.599149386954389	-
6769357	Txnrd1	0.333	0.599149386954389	-
6781201	Gria1	0.333	0.599149386954389	-
6782136	Mink1	0.333	0.599149386954389	-
6789725	Rpa1	0.333	0.599149386954389	-
6797544	Ifi27l1	0.333	0.599149386954389	-
6811655	Zfp322a	0.333	0.599149386954389	-
6832300	Parvb	0.333	0.599149386954389	-
6856096	Sgol1	0.333	0.599149386954389	-
6859305	Rnf138	0.333	0.599149386954389	-
6860520	Myot	0.333	0.599149386954389	-
6878341	Rbm45	0.333	0.599149386954389	-
6883186	Eya2	0.333	0.599149386954389	-
6902183	Mcoln2	0.333	0.599149386954389	-
6905074	Cog6	0.333	0.599149386954389	-
6906676	BC023814	0.333	0.599149386954389	-
6907939	1700027A23Rik	0.333	0.599149386954389	-
6916815	Cited4	0.333	0.599149386954389	-
6926166	C1qc	0.333	0.599149386954389	-
6932336	Afp	0.333	0.599149386954389	-
6936780	Xrcc2	0.333	0.599149386954389	-
6942446	Ccl24	0.333	0.599149386954389	-
6945935	Cul1	0.333	0.599149386954389	-
6983999	Cbln1	0.333	0.599149386954389	-
6986033	Abcb10	0.333	0.599149386954389	-
6992332	Gpx1	0.333	0.599149386954389	-
6995810	Nrg4	0.333	0.599149386954389	-
6998587	Parp3	0.333	0.599149386954389	-
7016797	Rap2c	0.333	0.599149386954389	-
6747805	Rdh10	0.334	0.599149386954389	-
6756815	Cops5	0.334	0.599149386954389	-
6760777	Hdlbp	0.334	0.599149386954389	-
6763110	Smg7	0.334	0.599149386954389	-
6769175	Grin3b	0.334	0.599149386954389	-
6775674	Cry1	0.334	0.599149386954389	+
6777309	Lyz2	0.334	0.599149386954389	-
6777583	Rassf3	0.334	0.599149386954389	-
6782447	Prpf8	0.334	0.599149386954389	-
6783054	Hnf1b	0.334	0.599149386954389	-

6789167	Wdr16	0.334	0.599149386954389	-
6794483	Agr2	0.334	0.599149386954389	-
6824986	Efha1	0.334	0.599149386954389	-
6825679	9930012K11Rik	0.334	0.599149386954389	-
6838694	Krt79	0.334	0.599149386954389	-
6848579	Ccr6	0.334	0.599149386954389	-
6853905	Wdr27	0.334	0.599149386954389	-
6854596	Tead3	0.334	0.599149386954389	-
6870028	Elovl3	0.334	0.599149386954389	-
6873396	Pitx3	0.334	0.599149386954389	-
6876161	Aif1l	0.334	0.599149386954389	-
6879083	Ambra1	0.334	0.599149386954389	-
6889894	Arhgap11a	0.334	0.599149386954389	-
6892984	Ncoa5	0.334	0.599149386954389	-
6922846	Nfib	0.334	0.599149386954389	-
6927317	Gltpd1	0.334	0.599149386954389	-
6934607	Sfswap	0.334	0.599149386954389	-
6937512	Man2b2	0.334	0.599149386954389	-
6947555	Cct7	0.334	0.599149386954389	-
6958407	Tmtc1	0.334	0.599149386954389	-
6959487	Ppp1r14a	0.334	0.599149386954389	-
6960723	Gas2	0.334	0.599149386954389	-
6963856	6330503K22Rik	0.334	0.599149386954389	-
6964737	Fank1	0.334	0.599149386954389	-
6968806	Sec11a	0.334	0.599149386954389	-
6977778	Prdx2	0.334	0.599149386954389	-
6989874	Tipin	0.334	0.599149386954389	-
7014558	Smc1a	0.334	0.599149386954389	-
7015530	Dynlt3	0.334	0.599149386954389	-
6748855	D1Bwg0212e	0.335	0.599149386954389	-
6781414	Rai1	0.335	0.599149386954389	-
6781569	Pigl	0.335	0.599149386954389	-
6782801	Suz12	0.335	0.599149386954389	-
6785173	2210020M01Rik	0.335	0.599149386954389	-
6789476	Derl2	0.335	0.599149386954389	-
6795811	Atl1	0.335	0.599149386954389	-
6796401	Exd2	0.335	0.599149386954389	-
6797917	Evl	0.335	0.599149386954389	-
6814206	Adamts16	0.335	0.599149386954389	-
6819197	Bcl2l2	0.335	0.599149386954389	-
6826927	Dis3	0.335	0.599149386954389	-
6835886	BC026439	0.335	0.599149386954389	-
6840604	Lmln	0.335	0.599149386954389	-
6844302	Dgcr2	0.335	0.599149386954389	-
6845416	Stfa3	0.335	0.599149386954389	-
6849621	Pi16	0.335	0.599149386954389	-
6859971	Reep2	0.335	0.599149386954389	-
6876380	Gsn	0.335	0.599149386954389	-
6919199	Ube2j2	0.335	0.599149386954389	-
6935174	Heatr2	0.335	0.599149386954389	-

6959616	Sbsn	0.335	0.599149386954389	-
6964310	Fbrs	0.335	0.599149386954389	-
6974010	Ing1	0.335	0.599149386954389	-
6977169	Nwd1	0.335	0.599149386954389	-
6979631	Ctu2	0.335	0.599149386954389	-
6980270	Efnb2	0.335	0.599149386954389	-
6751820	Gin1	0.336	0.599149386954389	-
6772482	Pex7	0.336	0.599149386954389	-
6782776	Nf1	0.336	0.599149386954389	-
6795328	Pax9	0.336	0.599149386954389	-
6803321	4831426I19Rik	0.336	0.599149386954389	-
6805616	Prl5a1	0.336	0.599149386954389	-
6831847	Gga1	0.336	0.599149386954389	-
6837130	Dnalc4	0.336	0.599149386954389	-
6837809	Lmf2	0.336	0.599149386954389	-
6848940	Riok2	0.336	0.599149386954389	-
6849467	Bnip1	0.336	0.599149386954389	-
6850055	Gpsm3	0.336	0.599149386954389	-
6857544	Sos1	0.336	0.599149386954389	-
6874626	Sephs1	0.336	0.599149386954389	-
6890440	Shf	0.336	0.599149386954389	-
6892899	Slpi	0.336	0.599149386954389	-
6901944	Gbp5	0.336	0.599149386954389	-
6903875	Mecom	0.336	0.599149386954389	-
6908137	Gpsm2	0.336	0.599149386954389	-
6934957	Zp3	0.336	0.599149386954389	-
6952665	Cnot4	0.336	0.599149386954389	-
6966136	Dll3	0.336	0.599149386954389	-
6978817	Fbxl8	0.336	0.599149386954389	-
6987641	Ncapd3	0.336	0.599149386954389	-
6749785	Bmpr2	0.337	0.599149386954389	-
6751698	Sep 02	0.337	0.599149386954389	-
6759275	Mdh1b	0.337	0.599149386954389	-
6760896	Slco6c1	0.337	0.599149386954389	-
6781492	Kcnj12	0.337	0.599149386954389	-
6785538	Narf	0.337	0.599149386954389	-
6790228	Rad51l3	0.337	0.599149386954389	-
6790340	Lhx1	0.337	0.599149386954389	-
6791644	Kif18b	0.337	0.599149386954389	-
6797950	Yy1	0.337	0.599149386954389	-
6811366	Epdr1	0.337	0.599149386954389	-
6814996	Atg10	0.337	0.599149386954389	+
6849845	Rrp1b	0.337	0.599149386954389	-
6850750	Prph2	0.337	0.599149386954389	-
6854468	Itfg3	0.337	0.599149386954389	-
6867609	Chka	0.337	0.599149386954389	-
6869027	Jak2	0.337	0.599149386954389	-
6883614	Tcfap2c	0.337	0.599149386954389	-
6890375	Trp53bp1	0.337	0.599149386954389	-
6945914	Cntnap2	0.337	0.599149386954389	-

6952451	Copg2	0.337	0.599149386954389	-
6984312	--	0.337	0.599149386954389	-
6985924	Cyba	0.337	0.599149386954389	-
6989414	Scamp2	0.337	0.599149386954389	-
6992224	Hyal1	0.337	0.599149386954389	-
6994666	Chek1	0.337	0.599149386954389	-
6996191	Glce	0.337	0.599149386954389	-
6996370	Zfp609	0.337	0.599149386954389	-
7010647	Zcchc12	0.337	0.599149386954389	-
7015028	Fancb	0.337	0.599149386954389	-
6757928	Ankrd39	0.338	0.599149386954389	-
6764956	Rrp15	0.338	0.599149386954389	-
6769020	1700009J07Rik	0.338	0.599149386954389	-
6780551	Havcr2	0.338	0.599149386954389	-
6784187	Fkbp10	0.338	0.599149386954389	-
6805096	Vps41	0.338	0.599149386954389	-
6824553	Tep1	0.338	0.599149386954389	-
6824743	Jub	0.338	0.599149386954389	-
6845435	Cd86	0.338	0.599149386954389	-
6858520	Mtpap	0.338	0.599149386954389	-
6870982	Rad9	0.338	0.599149386954389	-
6872204	Mamdc2	0.338	0.599149386954389	-
6875038	Il2ra	0.338	0.599149386954389	-
6885489	Agpat2	0.338	0.599149386954389	-
6886917	Gpd2	0.338	0.599149386954389	-
6903454	Cyp7b1	0.338	0.599149386954389	+
6904530	Mfsd8	0.338	0.599149386954389	-
6906877	Creb3l4	0.338	0.599149386954389	-
6925558	Rbbp4	0.338	0.599149386954389	-
6934923	Nsun5	0.338	0.599149386954389	-
6967091	Uevld	0.338	0.599149386954389	+
6979055	Zfp821	0.338	0.599149386954389	-
6979433	Bcmo1	0.338	0.599149386954389	-
7003143	AU018091	0.338	0.599149386954389	-
6781032	Rapgef6	0.339	0.599149386954389	-
6781487	Map2k3	0.339	0.599149386954389	-
6810659	BC016423	0.339	0.599149386954389	-
6825223	6330409N04Rik	0.339	0.599149386954389	-
6825705	Sftpc	0.339	0.599149386954389	-
6832010	Fam83f	0.339	0.599149386954389	-
6841097	Naa50	0.339	0.599149386954389	+
6844249	Ppil2	0.339	0.599149386954389	-
6853948	Pdcd2	0.339	0.599149386954389	-
6863158	Kif5b	0.339	0.599149386954389	-
6870130	Pdcd11	0.339	0.599149386954389	-
6870746	Pnliprp2	0.339	0.599149386954389	-
6871136	Pcnxl3	0.339	0.599149386954389	-
6871467	Best1	0.339	0.599149386954389	-
6877478	March7	0.339	0.599149386954389	-
6899385	Pglyrp4	0.339	0.599149386954389	-

6912098	Coq3	0.339	0.599149386954389	-
6913321	Invs	0.339	0.599149386954389	-
6916937	Mycl1	0.339	0.599149386954389	-
6924171	C8a	0.339	0.599149386954389	-
6924887	Szt2	0.339	0.599149386954389	-
6942503	Emid2	0.339	0.599149386954389	-
6945202	1700012A03Rik	0.339	0.599149386954389	-
6955766	A130022J15Rik	0.339	0.599149386954389	-
6958897	Ceacam9	0.339	0.599149386954389	-
6960488	Gtf2h1	0.339	0.599149386954389	-
6963413	Ipo7	0.339	0.599149386954389	-
6980100	Cd209e	0.339	0.599149386954389	-
6981759	Dlc1	0.339	0.599149386954389	-
6989409	2310046O06Rik	0.339	0.599149386954389	-
6757449	Col19a1	0.34	0.599149386954389	-
6759775	1810031K17Rik	0.34	0.599149386954389	-
6763217	Xpr1	0.34	0.599149386954389	-
6765551	Syne1	0.34	0.599149386954389	-
6765723	Katna1	0.34	0.599149386954389	-
6781372	Mprip	0.34	0.599149386954389	-
6783448	--	0.34	0.599149386954389	-
6791543	Hdac5	0.34	0.599149386954389	-
6798271	Adssl1	0.34	0.599149386954389	-
6813187	Sema4d	0.34	0.599149386954389	-
6819948	Ebf2	0.34	0.599149386954389	-
6840129	Rtp4	0.34	0.599149386954389	-
6854369	Mlst8	0.34	0.599149386954389	-
6871514	Tmem132a	0.34	0.599149386954389	-
6875564	Gpr158	0.34	0.599149386954389	-
6881231	Atrn	0.34	0.599149386954389	-
6881340	Mcm8	0.34	0.599149386954389	-
6883087	Svs5	0.34	0.599149386954389	-
6883317	Slc9a8	0.34	0.599149386954389	-
6903129	Zfp704	0.34	0.599149386954389	-
6921015	Nol6	0.34	0.599149386954389	-
6929720	Plb1	0.34	0.599149386954389	-
6932175	Csn1s2a	0.34	0.599149386954389	-
6949766	Cd163	0.34	0.599149386954389	-
6951088	Fgfr1op2	0.34	0.599149386954389	-
6965771	Vasp	0.34	0.599149386954389	-
6967881	Fan1	0.34	0.599149386954389	-
6973757	Lass4	0.34	0.599149386954389	-
6974101	1700094C09Rik	0.34	0.599149386954389	-
6978890	Prmt7	0.34	0.599149386954389	-
6983214	Pgpep1	0.34	0.599149386954389	-
6985343	Ddx19b	0.34	0.599149386954389	-
6995071	C2cd2l	0.34	0.599149386954389	-
6749773	Nop58	0.341	0.599149386954389	-
6778562	Zmiz2	0.341	0.599149386954389	-
6787741	Nipal4	0.341	0.599149386954389	-

6810166	Plk2	0.341	0.599149386954389	+
6819296	Mcpt2	0.341	0.599149386954389	-
6825696	Polr3d	0.341	0.599149386954389	-
6835415	Nudcd1	0.341	0.599149386954389	-
6867685	Spnb3	0.341	0.599149386954389	-
6868013	Fth1	0.341	0.599149386954389	-
6870967	Ndufv1	0.341	0.599149386954389	-
6882582	Ergic3	0.341	0.599149386954389	-
6888754	Ddb2	0.341	0.599149386954389	-
6929676	Trim54	0.341	0.599149386954389	-
6951107	Arntl2	0.341	0.599149386954389	-
6959208	Tmem145	0.341	0.599149386954389	-
6964299	Prr14	0.341	0.599149386954389	-
6971356	E430018J23Rik	0.341	0.599149386954389	-
6973527	Lair1	0.341	0.599149386954389	-
6987324	Zfp846	0.341	0.599149386954389	-
6987838	Snx19	0.341	0.599149386954389	-
6993272	Mmp1b	0.341	0.599149386954389	-
6999147	Cmtm7	0.341	0.599149386954389	-
7011051	Slc25a14	0.341	0.599149386954389	-
6749676	9430016H08Rik	0.342	0.599149386954389	-
6752732	Gpr39	0.342	0.599149386954389	-
6752848	Mgat5	0.342	0.599149386954389	-
6768110	Sh3rf3	0.342	0.599149386954389	-
6779818	Cpeb4	0.342	0.599149386954389	-
6781612	Pmp22	0.342	0.599149386954389	+
6784552	Efcab3	0.342	0.599149386954389	-
6822139	Fam60a	0.342	0.599149386954389	-
6844321	D16H22S680E	0.342	0.599149386954389	-
6849319	Hagh	0.342	0.599149386954389	-
6849970	Kank3	0.342	0.599149386954389	-
6856231	Ptprs	0.342	0.599149386954389	-
6860198	Pcdhb16	0.342	0.599149386954389	-
6863605	Aqp4	0.342	0.599149386954389	-
6873128	Tll2	0.342	0.599149386954389	-
6880715	Pldn	0.342	0.599149386954389	-
6888276	Zswim2	0.342	0.599149386954389	-
6889432	Elp4	0.342	0.599149386954389	-
6935519	Cyp3a25	0.342	0.599149386954389	-
6949958	Akap3	0.342	0.599149386954389	-
6954385	Gadd45a	0.342	0.599149386954389	-
6954565	Rmnd5a	0.342	0.599149386954389	-
6986034	Taf5l	0.342	0.599149386954389	-
6759723	Cxcr1	0.343	0.599149386954389	-
6762448	Pkp1	0.343	0.599149386954389	-
6764562	Ahctf1	0.343	0.599149386954389	-
6766060	Fuca2	0.343	0.599149386954389	-
6771644	Cnpy2	0.343	0.599149386954389	-
6804919	Arid4b	0.343	0.599149386954389	-
6815853	Zswim6	0.343	0.599149386954389	+

6824001	Rgr	0.343	0.599149386954389	-
6824004	Cdhr1	0.343	0.599149386954389	-
6837897	Cpne8	0.343	0.599149386954389	-
6841703	Impg2	0.343	0.599149386954389	-
6841862	Cpox	0.343	0.599149386954389	-
6856208	D17Wsu104e	0.343	0.599149386954389	-
6888937	Hsd17b12	0.343	0.599149386954389	+
6909303	Rrh	0.343	0.599149386954389	-
6930383	Cd38	0.343	0.599149386954389	-
6932510	Shroom3	0.343	0.599149386954389	-
6935910	Ankib1	0.343	0.599149386954389	-
6941805	Diablo	0.343	0.599149386954389	-
6949593	Slc6a12	0.343	0.599149386954389	-
6967068	Hps5	0.343	0.599149386954389	-
6975247	Gtf2e2	0.343	0.599149386954389	-
6979451	Hsd17b2	0.343	0.599149386954389	-
6995964	Pml	0.343	0.599149386954389	-
6998858	Klhl18	0.343	0.599149386954389	-
6760915	Ppip5k2	0.344	0.599149386954389	-
6771636	Timeless	0.344	0.599149386954389	-
6782087	Cldn7	0.344	0.599149386954389	-
6783152	1700125H20Rik	0.344	0.599149386954389	-
6789343	Chrnb1	0.344	0.599149386954389	-
6802043	Zfyve26	0.344	0.599149386954389	-
6812516	Muted	0.344	0.599149386954389	-
6814355	Nkd2	0.344	0.599149386954389	+
6817944	Sfmbt1	0.344	0.599149386954389	-
6849235	Prss32	0.344	0.599149386954389	-
6850173	Pou5f1	0.344	0.599149386954389	-
6850797	Pgc	0.344	0.599149386954389	-
6861705	Cidea	0.344	0.599149386954389	-
6867883	Trpt1	0.344	0.599149386954389	-
6873127	Opalin	0.344	0.599149386954389	-
6881337	Chgb	0.344	0.599149386954389	-
6893002	Slc13a3	0.344	0.599149386954389	-
6925829	Smpdl3b	0.344	0.599149386954389	-
6928481	4932412H11Rik	0.344	0.599149386954389	-
6931362	Limch1	0.344	0.599149386954389	-
6934652	Tpst1	0.344	0.599149386954389	-
6953331	Ezh2	0.344	0.599149386954389	-
6966976	Snrnp70	0.344	0.599149386954389	-
6970147	Rrp8	0.344	0.599149386954389	-
6977660	4933434I20Rik	0.344	0.599149386954389	-
6978357	Ccdc135	0.344	0.599149386954389	-
6979701	Tcf25	0.344	0.599149386954389	-
6986030	1700054N08Rik	0.344	0.599149386954389	-
6991563	Tfdp2	0.344	0.599149386954389	-
7013857	Cenpi	0.344	0.599149386954389	-
7018262	Maged1	0.344	0.599149386954389	-
6749461	Mstn	0.345	0.599149386954389	-

6773940	Gopc	0.345	0.599149386954389	-
6779834	Mpg	0.345	0.599149386954389	-
6784251	Ramp2	0.345	0.599149386954389	-
6785188	Unk	0.345	0.599149386954389	-
6791561	Slc4a1	0.345	0.599149386954389	-
6791793	1700081L11Rik	0.345	0.599149386954389	-
6799064	Ddx1	0.345	0.599149386954389	-
6815391	Btf3	0.345	0.599149386954389	-
6832086	Sgsm3	0.345	0.599149386954389	-
6837008	Csf2rb2	0.345	0.599149386954389	-
6844227	Spag6	0.345	0.599149386954389	-
6846455	Nit2	0.345	0.599149386954389	-
6850507	Gpr110	0.345	0.599149386954389	-
6858819	Mib1	0.345	0.599149386954389	-
6860068	Cxxc5	0.345	0.599149386954389	-
6875722	Abca2	0.345	0.599149386954389	-
6875988	Ttf1	0.345	0.599149386954389	-
6883115	Snx21	0.345	0.599149386954389	-
6892896	Semg1	0.345	0.599149386954389	-
6900734	Frrs1	0.345	0.599149386954389	-
6907958	Cept1	0.345	0.599149386954389	-
6913774	Palm2	0.345	0.599149386954389	-
6921020	Ubap2	0.345	0.599149386954389	-
6932234	Dck	0.345	0.599149386954389	-
6932930	Cds1	0.345	0.599149386954389	-
6933997	Oas1b	0.345	0.599149386954389	-
6956537	Lhfpl4	0.345	0.599149386954389	-
6972030	Nkx6-2	0.345	0.599149386954389	-
7019970	Dcx	0.345	0.599149386954389	-
6760289	Pid1	0.346	0.599149386954389	-
6783327	Mtmr4	0.346	0.599149386954389	-
6797719	Tcl1b5	0.346	0.599149386954389	-
6802382	Mlh3	0.346	0.599149386954389	-
6806444	Tmem14c	0.346	0.599149386954389	-
6808977	Gm4814	0.346	0.599149386954389	-
6821301	Pibf1	0.346	0.599149386954389	-
6833183	Accn2	0.346	0.599149386954389	-
6849534	Def6	0.346	0.599149386954389	-
6850062	Prrt1	0.346	0.599149386954389	+
6851897	Epb4.1l3	0.346	0.599149386954389	-
6860912	1700034E13Rik	0.346	0.599149386954389	-
6860920	Sncaip	0.346	0.599149386954389	-
6866068	Txnl1	0.346	0.599149386954389	-
6869068	Il33	0.346	0.599149386954389	-
6870107	Cnnm2	0.346	0.599149386954389	-
6892725	Emilin3	0.346	0.599149386954389	-
6899262	Adar	0.346	0.599149386954389	-
6908088	Gstm4	0.346	0.599149386954389	+
6916784	Edn2	0.346	0.599149386954389	-
6939987	Cxcl9	0.346	0.599149386954389	-

6957632	Mansc1	0.346	0.599149386954389	-
6958995	Opa3	0.346	0.599149386954389	-
6963323	Eif3f	0.346	0.599149386954389	-
6966934	Rcn3	0.346	0.599149386954389	-
6971274	Cln3	0.346	0.599149386954389	-
6971466	1110007A13Rik	0.346	0.599149386954389	-
6995507	Tex12	0.346	0.599149386954389	-
6995743	Cyp19a1	0.346	0.599149386954389	-
7013137	2610029G23Rik	0.346	0.599149386954389	-
6754526	Ankrd45	0.347	0.599149386954389	-
6768951	Slc19a1	0.347	0.599149386954389	-
6785441	Mrpl12	0.347	0.599149386954389	-
6788141	Phf15	0.347	0.599149386954389	-
6806038	Serpinb9b	0.347	0.599149386954389	-
6833176	Aqp6	0.347	0.599149386954389	-
6838999	Nat15	0.347	0.599149386954389	-
6842587	Chodl	0.347	0.599149386954389	-
6849552	4930511I11Rik	0.347	0.599149386954389	-
6854271	Kremen2	0.347	0.599149386954389	-
6867976	Ganab	0.347	0.599149386954389	-
6871058	Bbs1	0.347	0.599149386954389	-
6890332	Ubr1	0.347	0.599149386954389	-
6899789	Pdzk1	0.347	0.599149386954389	-
6916640	Eri3	0.347	0.599149386954389	-
6933154	D830014E11Rik	0.347	0.599149386954389	-
6947854	4933427D06Rik	0.347	0.599149386954389	-
6947939	Slc41a3	0.347	0.599149386954389	-
6953804	Ggct	0.347	0.599149386954389	-
6954269	Hpgds	0.347	0.599149386954389	-
6964068	Tnrc6a	0.347	0.599149386954389	-
6966928	Prrg2	0.347	0.599149386954389	-
6987366	Atg4d	0.347	0.599149386954389	-
6993850	Rgl3	0.347	0.599149386954389	-
6996246	Skor1	0.347	0.599149386954389	-
7001691	Hhipl2	0.347	0.599149386954389	-
6754047	Glt25d2	0.348	0.599149386954389	-
6756346	Batf3	0.348	0.599149386954389	-
6757433	Fam135a	0.348	0.599149386954389	-
6763208	Mr1	0.348	0.599149386954389	-
6773417	AI317395	0.348	0.599149386954389	-
6775521	Nfyb	0.348	0.599149386954389	-
6781840	Myh8	0.348	0.599149386954389	-
6782286	Itgae	0.348	0.599149386954389	-
6791439	Kat2a	0.348	0.599149386954389	-
6792418	Grin2c	0.348	0.599149386954389	-
6792991	Itsn2	0.348	0.599149386954389	-
6803922	Brf1	0.348	0.599149386954389	-
6804900	Gng4	0.348	0.599149386954389	-
6810620	Paip1	0.348	0.599149386954389	-
6829760	Spag1	0.348	0.599149386954389	-

6848594	Pde10a	0.348	0.599149386954389	-
6849435	Rgs11	0.348	0.599149386954389	-
6861260	Isoc1	0.348	0.599149386954389	+
6865137	9530002K18Rik	0.348	0.599149386954389	-
6871479	Dagla	0.348	0.599149386954389	-
6875804	Fam69b	0.348	0.599149386954389	-
6877080	Rif1	0.348	0.599149386954389	-
6889350	Tcp11l1	0.348	0.599149386954389	-
6936746	4931409K22Rik	0.348	0.599149386954389	-
6949706	Nanog	0.348	0.599149386954389	-
6964293	Itgal	0.348	0.599149386954389	-
6968772	Cib1	0.348	0.599149386954389	-
6970009	Hbb-y	0.348	0.599149386954389	-
6973546	Cabp5	0.348	0.599149386954389	-
6977861	Lonp2	0.348	0.599149386954389	-
6989909	Dpp8	0.348	0.599149386954389	-
6990527	Myo5a	0.348	0.599149386954389	-
6991538	Pcolce2	0.348	0.599149386954389	-
6754799	Cd247	0.349	0.599149386954389	-
6768323	Slc25a16	0.349	0.599149386954389	-
6775246	Pwp2	0.349	0.599149386954389	-
6778068	Ormdl2	0.349	0.599149386954389	-
6788314	Pdlim4	0.349	0.599149386954389	+
6809030	Arsb	0.349	0.599149386954389	+
6813096	Fgd3	0.349	0.599149386954389	-
6817954	Mustn1	0.349	0.599149386954389	-
6818647	Cdkn3	0.349	0.599149386954389	-
6822731	Cadps	0.349	0.599149386954389	-
6823084	Ppp3cb	0.349	0.599149386954389	-
6828651	Lmbrd2	0.349	0.599149386954389	-
6832713	Lrrk2	0.349	0.599149386954389	-
6850711	Mrpl2	0.349	0.599149386954389	-
6857434	Heatr5b	0.349	0.599149386954389	-
6860424	Rbm27	0.349	0.599149386954389	-
6877888	4932414N04Rik	0.349	0.599149386954389	-
6915844	Dnajc6	0.349	0.599149386954389	-
6919064	Megf6	0.349	0.599149386954389	-
6941681	Adam1a	0.349	0.599149386954389	-
6945170	Mkln1	0.349	0.599149386954389	-
6952523	Plxna4	0.349	0.599149386954389	-
6957327	Tead4	0.349	0.599149386954389	-
6957756	Wbp11	0.349	0.599149386954389	-
6962922	Kcne3	0.349	0.599149386954389	-
6971316	Mvp	0.349	0.599149386954389	-
6996311	Ptplad1	0.349	0.599149386954389	-
6999443	Scn11a	0.349	0.599149386954389	-
7009773	Cacna1f	0.349	0.599149386954389	-
7011663	Ctag2	0.349	0.599149386954389	-
6753792	Fam5c	0.35	0.599149386954389	-
6765281	Rcor3	0.35	0.599149386954389	-

6767155	Hdac2	0.35	0.599149386954389	-
6778055	Cdk2	0.35	0.599149386954389	-
6778998	C1d	0.35	0.599149386954389	-
6782141	Eno3	0.35	0.599149386954389	-
6784605	Ddx42	0.35	0.599149386954389	-
6801499	Sav1	0.35	0.599149386954389	-
6805103	Amph	0.35	0.599149386954389	-
6805452	Tdp2	0.35	0.599149386954389	-
6815535	Naip1	0.35	0.599149386954389	-
6825429	Elp3	0.35	0.599149386954389	-
6828793	Mtmr12	0.35	0.599149386954389	-
6840249	Cldn16	0.35	0.599149386954389	-
6848854	Fam120b	0.35	0.599149386954389	-
6856215	Ticam1	0.35	0.599149386954389	-
6865551	Ppic	0.35	0.599149386954389	-
6867860	Rasgrp2	0.35	0.599149386954389	-
6902440	Eltd1	0.35	0.599149386954389	+
6906826	Efna4	0.35	0.599149386954389	-
6918814	Clstn1	0.35	0.599149386954389	-
6949237	Rho	0.35	0.599149386954389	-
6955268	Arhgap25	0.35	0.599149386954389	-
6959632	Lgi4	0.35	0.599149386954389	-
6964519	Btbd16	0.35	0.599149386954389	-
6965932	Dedd2	0.35	0.599149386954389	-
6969014	Mesdc1	0.35	0.599149386954389	-
6976498	Nek1	0.35	0.599149386954389	-
6984513	Cngb1	0.35	0.599149386954389	-
6995879	Commd4	0.35	0.599149386954389	-
7011413	Cd40lg	0.35	0.599149386954389	-
7015399	Ccdc22	0.35	0.599149386954389	-
7016397	Rhox11	0.35	0.599149386954389	-
6752730	Slc35f5	0.351	0.599149386954389	-
6754252	Tor1aip2	0.351	0.599149386954389	-
6765119	Kctd3	0.351	0.599149386954389	-
6771620	Prim1	0.351	0.599149386954389	-
6781526	Fam83g	0.351	0.599149386954389	-
6802507	Pomt2	0.351	0.599149386954389	-
6841068	Drd3	0.351	0.599149386954389	-
6849216	Cldn6	0.351	0.599149386954389	-
6890296	Vps39	0.351	0.599149386954389	-
6925345	1700029G01Rik	0.351	0.599149386954389	-
6934650	Crcp	0.351	0.599149386954389	-
6939661	Cenpc1	0.351	0.599149386954389	-
6939888	Rassf6	0.351	0.599149386954389	-
6945034	Ahcyl2	0.351	0.599149386954389	-
6948418	Mitf	0.351	0.599149386954389	-
6956932	Wnk1	0.351	0.599149386954389	-
6962935	Ucp2	0.351	0.599149386954389	-
6966033	Itpkc	0.351	0.599149386954389	+
6966799	4931406B18Rik	0.351	0.599149386954389	-

6977093	Glt25d1	0.351	0.599149386954389	-
6978794	Ces2g	0.351	0.599149386954389	-
6980167	Slc10a2	0.351	0.599149386954389	-
7017699	Pls3	0.351	0.599149386954389	-
6750848	Sgpp2	0.352	0.599149386954389	-
6751655	Agxt	0.352	0.599149386954389	-
6768151	Spock2	0.352	0.599149386954389	-
6775887	Slc17a8	0.352	0.599149386954389	-
6811300	Pou6f2	0.352	0.599149386954389	-
6815518	Mccc2	0.352	0.599149386954389	+
6820325	Lcp1	0.352	0.599149386954389	-
6822320	Pcca	0.352	0.599149386954389	-
6849515	Pacsin1	0.352	0.599149386954389	-
6849994	Vps52	0.352	0.599149386954389	-
6885421	C8g	0.352	0.599149386954389	-
6885462	Lhx3	0.352	0.599149386954389	-
6888002	Prkra	0.352	0.599149386954389	-
6906747	Slc25a44	0.352	0.599149386954389	-
6924243	BC055111	0.352	0.599149386954389	-
6933591	Dao	0.352	0.599149386954389	-
6935200	Gpr30	0.352	0.599149386954389	-
6956550	Camk1	0.352	0.599149386954389	-
6970944	Cdr2	0.352	0.599149386954389	-
6975904	Mlf1ip	0.352	0.599149386954389	-
6980990	Agpat6	0.352	0.599149386954389	-
6985006	Slc12a4	0.352	0.599149386954389	-
6757981	Tsga10	0.353	0.599149386954389	-
6760232	Slc19a3	0.353	0.599149386954389	+
6762094	Cd55	0.353	0.599149386954389	-
6764050	Ndufs2	0.353	0.599149386954389	-
6775299	Mier2	0.353	0.599149386954389	-
6781885	Gas7	0.353	0.599149386954389	-
6782389	Mett10d	0.353	0.599149386954389	-
6795595	Prpf39	0.353	0.599149386954389	-
6802197	Map3k9	0.353	0.599149386954389	-
6824634	Supt16h	0.353	0.599149386954389	-
6834561	Trio	0.353	0.599149386954389	-
6837105	Csnk1e	0.353	0.599149386954389	-
6848581	Sft2d1	0.353	0.599149386954389	-
6849325	Spsb3	0.353	0.599149386954389	-
6864837	Fgf1	0.353	0.599149386954389	-
6879935	Lin7c	0.353	0.599149386954389	-
6880776	Sema6d	0.353	0.599149386954389	-
6888720	Ptpmt1	0.353	0.599149386954389	-
6888925	Ext2	0.353	0.599149386954389	-
6888968	Api5	0.353	0.599149386954389	-
6899496	Rptn	0.353	0.599149386954389	-
6900431	4921515J06Rik	0.353	0.599149386954389	-
6920960	Topors	0.353	0.599149386954389	-
6921120	BC049635	0.353	0.599149386954389	-

6923477	Cyp2j13	0.353	0.599149386954389	-
6924754	Nsun4	0.353	0.599149386954389	-
6933812	Tesc	0.353	0.599149386954389	-
6942379	Limk1	0.353	0.599149386954389	-
6954991	Dok1	0.353	0.599149386954389	-
6958833	Kptn	0.353	0.599149386954389	-
6960197	Klk15	0.353	0.599149386954389	-
6967803	Gm9801	0.353	0.599149386954389	-
6972415	Tnfrsf23	0.353	0.599149386954389	-
6987348	Mrpl4	0.353	0.599149386954389	-
6989100	Rdx	0.353	0.599149386954389	-
7014077	Il1rapl2	0.353	0.599149386954389	-
7016804	Mbnl3	0.353	0.599149386954389	-
6761914	Lypd1	0.354	0.599149386954389	-
6769282	Tbxa2r	0.354	0.599149386954389	-
6769305	Tle2	0.354	0.599149386954389	-
6786002	Zpbp	0.354	0.599149386954389	-
6792825	Ccdc57	0.354	0.599149386954389	-
6796666	Eif2b2	0.354	0.599149386954389	-
6802532	Sptlc2	0.354	0.599149386954389	-
6811136	Hecw1	0.354	0.599149386954389	-
6815797	Kif2a	0.354	0.599149386954389	-
6817337	Fam149b	0.354	0.599149386954389	-
6836260	Adcy8	0.354	0.599149386954389	-
6845154	Lrrc33	0.354	0.599149386954389	-
6849339	Ift140	0.354	0.599149386954389	-
6854371	Traf7	0.354	0.599149386954389	-
6867197	Cndp2	0.354	0.599149386954389	-
6867782	Kcnk7	0.354	0.599149386954389	-
6868181	Glyat	0.354	0.599149386954389	-
6870029	Gbf1	0.354	0.599149386954389	-
6872250	Tjp2	0.354	0.599149386954389	-
6882071	Cst9	0.354	0.599149386954389	-
6883677	1700021F07Rik	0.354	0.599149386954389	-
6898241	Smc4	0.354	0.599149386954389	-
6899889	Tbx15	0.354	0.599149386954389	-
6924750	Faah	0.354	0.599149386954389	+
6931181	Pgm1	0.354	0.599149386954389	-
6935150	Fam20c	0.354	0.599149386954389	-
6939109	Fryl	0.354	0.599149386954389	-
6966198	Spint2	0.354	0.599149386954389	-
6966986	Dhdh	0.354	0.599149386954389	-
6972174	Sigirr	0.354	0.599149386954389	-
6976765	March1	0.354	0.599149386954389	-
6983250	Ushbp1	0.354	0.599149386954389	-
6983558	Pou4f2	0.354	0.599149386954389	-
6993300	Yap1	0.354	0.599149386954389	-
6997367	Lca5	0.354	0.599149386954389	-
7012151	Tbl1x	0.354	0.599149386954389	-
6758435	Slc40a1	0.355	0.599149386954389	-

6776667	Tmtc2	0.355	0.599149386954389	-
6782139	Rnf167	0.355	0.599149386954389	+
6784254	Wnk4	0.355	0.599149386954389	-
6787099	Il9r	0.355	0.599149386954389	-
6789813	Nxn	0.355	0.599149386954389	-
6790275	Mmp28	0.355	0.599149386954389	-
6791405	Krt15	0.355	0.599149386954389	-
6802316	Aldh6a1	0.355	0.599149386954389	-
6804486	Gdi2	0.355	0.599149386954389	-
6808215	Tert	0.355	0.599149386954389	-
6808230	Brd9	0.355	0.599149386954389	-
6809550	Taf9	0.355	0.599149386954389	-
6809880	Htr1a	0.355	0.599149386954389	+
6813844	Ptch1	0.355	0.599149386954389	-
6815074	Cmya5	0.355	0.599149386954389	-
6838338	Vdr	0.355	0.599149386954389	-
6845551	Pla1a	0.355	0.599149386954389	-
6849098	BC049807	0.355	0.599149386954389	-
6854438	Metrn	0.355	0.599149386954389	-
6855062	C2	0.355	0.599149386954389	-
6871264	Kcnk4	0.355	0.599149386954389	-
6881652	Macrod2	0.355	0.599149386954389	-
6881895	Rin2	0.355	0.599149386954389	-
6885345	Cacna1b	0.355	0.599149386954389	-
6888744	Slc39a13	0.355	0.599149386954389	-
6896406	Pld1	0.355	0.599149386954389	-
6901952	Gbp1	0.355	0.599149386954389	-
6905529	Dhx36	0.355	0.599149386954389	-
6906840	Cks1b	0.355	0.599149386954389	-
6907277	Terc	0.355	0.599149386954389	-
6907652	Slc22a15	0.355	0.599149386954389	-
6908510	Slc35a3	0.355	0.599149386954389	-
6920954	Ddx58	0.355	0.599149386954389	-
6936116	Dmtf1	0.355	0.599149386954389	-
6939270	Nmu	0.355	0.599149386954389	-
6940674	Gbp11	0.355	0.599149386954389	-
6945004	Ccdc136	0.355	0.599149386954389	-
6946554	Abcg2	0.355	0.599149386954389	-
6948815	Trnt1	0.355	0.599149386954389	-
6949264	D6Wsu116e	0.355	0.599149386954389	-
6965608	Dhx34	0.355	0.599149386954389	-
6967004	Sphk2	0.355	0.599149386954389	-
6968804	Nmb	0.355	0.599149386954389	-
7010345	Usp11	0.355	0.599149386954389	+
7014560	Iqsec2	0.355	0.599149386954389	-
7016666	Smarca1	0.355	0.599149386954389	-
6755679	Lin9	0.356	0.599149386954389	-
6768351	Dnajc12	0.356	0.599149386954389	-
6771052	Ifng	0.356	0.599149386954389	-
6779972	Fam196b	0.356	0.599149386954389	-

6792965	Dnajc27	0.356	0.599149386954389	-
6813931	Hiatl1	0.356	0.599149386954389	-
6817416	Vdac2	0.356	0.599149386954389	-
6822852	Lrrc3b	0.356	0.599149386954389	-
6832775	Pphln1	0.356	0.599149386954389	-
6838794	Smug1	0.356	0.599149386954389	-
6840261	Il1rap	0.356	0.599149386954389	-
6844558	Etv5	0.356	0.599149386954389	-
6861358	Csf1r	0.356	0.599149386954389	-
6869864	Cnnm1	0.356	0.599149386954389	-
6879120	Pex16	0.356	0.599149386954389	-
6881625	2310003L22Rik	0.356	0.599149386954389	-
6884019	Phactr3	0.356	0.599149386954389	-
6894268	Eef1a2	0.356	0.599149386954389	-
6899667	Fam63a	0.356	0.599149386954389	-
6906694	Bcan	0.356	0.599149386954389	-
6907128	Pip5k1a	0.356	0.599149386954389	-
6911925	Nbn	0.356	0.599149386954389	-
6915650	Inadl	0.356	0.599149386954389	-
6917909	Wnt4	0.356	0.599149386954389	-
6926369	Pax7	0.356	0.599149386954389	-
6926495	Ctrc	0.356	0.599149386954389	-
6938178	Sepsecs	0.356	0.599149386954389	-
6960182	Klk6	0.356	0.599149386954389	-
6960931	Ube3a	0.356	0.599149386954389	-
6961913	Abhd2	0.356	0.599149386954389	-
6964478	Wdr11	0.356	0.599149386954389	-
6967013	Grwd1	0.356	0.599149386954389	-
6976560	Cbr4	0.356	0.599149386954389	-
6979504	Lrrc50	0.356	0.599149386954389	-
6984420	Bbs2	0.356	0.599149386954389	-
6991760	Faim	0.356	0.599149386954389	+
7018897	Hmgn5	0.356	0.599149386954389	-
6771693	Si	0.357	0.599149386954389	-
6784608	Tcam1	0.357	0.599149386954389	-
6797434	9030617O03Rik	0.357	0.599149386954389	-
6810548	Emb	0.357	0.599149386954389	-
6814018	Zfp456	0.357	0.599149386954389	-
6819907	Stmn4	0.357	0.599149386954389	+
6823114	Myoz1	0.357	0.599149386954389	-
6850120	Bat3	0.357	0.599149386954389	-
6855891	Rftn1	0.357	0.599149386954389	-
6856276	Slc25a23	0.357	0.599149386954389	-
6857415	Fez2	0.357	0.599149386954389	-
6868371	Gna14	0.357	0.599149386954389	-
6876310	Mapkap1	0.357	0.599149386954389	-
6883643	Rbm38	0.357	0.599149386954389	-
6890386	Strc	0.357	0.599149386954389	-
6899312	1700094D03Rik	0.357	0.599149386954389	-
6910597	Ptgfr	0.357	0.599149386954389	-

6917124	Mrps15	0.357	0.599149386954389	-
6935292	Lfng	0.357	0.599149386954389	-
6941051	Hscb	0.357	0.599149386954389	-
6941187	Ssh1	0.357	0.599149386954389	-
6999196	Stt3b	0.357	0.599149386954389	-
7010835	Gria3	0.357	0.599149386954389	-
6772104	Rab32	0.358	0.599149386954389	-
6779688	Pnpt1	0.358	0.599149386954389	-
6781727	Elac2	0.358	0.599149386954389	-
6786666	0610010F05Rik	0.358	0.599149386954389	-
6794939	Foxg1	0.358	0.599149386954389	-
6812261	Peci	0.358	0.599149386954389	-
6825137	Nupl1	0.358	0.599149386954389	-
6846029	Pvrl3	0.358	0.599149386954389	-
6850071	Rdbp	0.358	0.599149386954389	-
6859850	Camk4	0.358	0.599149386954389	-
6867627	Doc2g	0.358	0.599149386954389	-
6874722	Usp6nl	0.358	0.599149386954389	-
6878028	Gm1631	0.358	0.599149386954389	-
6880513	Dll4	0.358	0.599149386954389	-
6882333	Tpx2	0.358	0.599149386954389	-
6883740	Th1l	0.358	0.599149386954389	-
6885733	Zdhhc12	0.358	0.599149386954389	-
6907615	Ttf2	0.358	0.599149386954389	-
6922004	Lpar1	0.358	0.599149386954389	-
6929298	Abcb8	0.358	0.599149386954389	-
6945776	Tmem139	0.358	0.599149386954389	-
6957343	Itfg2	0.358	0.599149386954389	-
6963124	Olfr631	0.358	0.599149386954389	-
6968682	Hapln3	0.358	0.599149386954389	-
6970650	Psma1	0.358	0.599149386954389	-
6973206	Il11	0.358	0.599149386954389	-
6978263	Gnao1	0.358	0.599149386954389	-
6987325	5730577I03Rik	0.358	0.599149386954389	-
6993153	Ccr2	0.358	0.599149386954389	-
6995273	Apoc3	0.358	0.599149386954389	-
6995393	Rexo2	0.358	0.599149386954389	-
6755845	Trp53bp2	0.359	0.599149386954389	-
6759248	Ndufs1	0.359	0.599149386954389	-
6764910	Lyplal1	0.359	0.599149386954389	-
6768843	Gnaz	0.359	0.599149386954389	-
6769033	Icosl	0.359	0.599149386954389	-
6785148	Mrps7	0.359	0.599149386954389	-
6795522	Lrfn5	0.359	0.599149386954389	-
6803122	Smek1	0.359	0.599149386954389	-
6818181	Antxrl	0.359	0.599149386954389	-
6849438	Luc7l	0.359	0.599149386954389	-
6869086	Mbl2	0.359	0.599149386954389	-
6885195	Arhgap21	0.359	0.599149386954389	-
6888029	Cwc22	0.359	0.599149386954389	-

6907088	Oaz3	0.359	0.599149386954389	-
6908146	Stxbp3a	0.359	0.599149386954389	-
6917312	2510006D16Rik	0.359	0.599149386954389	-
6921670	Abca1	0.359	0.599149386954389	-
6949814	Cdca3	0.359	0.599149386954389	-
6952419	Zc3hc1	0.359	0.599149386954389	-
6961279	Igf1r	0.359	0.599149386954389	-
6977261	Mcm5	0.359	0.599149386954389	-
6979111	St3gal2	0.359	0.599149386954389	-
6991777	Cep70	0.359	0.599149386954389	+
6759378	4921521F21Rik	0.36	0.599149386954389	-
6762236	Nfasc	0.36	0.599149386954389	-
6763240	Tor1aip1	0.36	0.599149386954389	-
6773582	Sobp	0.36	0.599149386954389	-
6783913	Socs7	0.36	0.599149386954389	-
6788921	BC046404	0.36	0.599149386954389	-
6790475	Brip1	0.36	0.599149386954389	-
6790513	Cltc	0.36	0.599149386954389	-
6791637	Gjc1	0.36	0.599149386954389	-
6825436	Esco2	0.36	0.599149386954389	-
6830481	Nov	0.36	0.599149386954389	+
6835353	Angpt1	0.36	0.599149386954389	-
6839340	Ciita	0.36	0.599149386954389	-
6844485	Thpo	0.36	0.599149386954389	-
6845419	Casr	0.36	0.599149386954389	-
6849474	Phf1	0.36	0.599149386954389	-
6871267	Plcb3	0.36	0.599149386954389	-
6877157	Arl6ip6	0.36	0.599149386954389	-
6899249	Shc1	0.36	0.599149386954389	-
6913193	1300002K09Rik	0.36	0.599149386954389	-
6926577	Oog4	0.36	0.599149386954389	-
6935043	Plod3	0.36	0.599149386954389	-
6951783	Ppp1r3a	0.36	0.599149386954389	-
6956965	Cecr5	0.36	0.599149386954389	-
6957640	Dusp16	0.36	0.599149386954389	-
6959477	Ggn	0.36	0.599149386954389	-
6963881	Acsm5	0.36	0.599149386954389	-
6965317	Tssc4	0.36	0.599149386954389	-
6988102	Rpusd4	0.36	0.599149386954389	-
6988147	Acrv1	0.36	0.599149386954389	-
7004010	Retnlg	0.36	0.599149386954389	-
7015445	Suv39h1	0.36	0.599149386954389	-
6757301	Tram2	0.361	0.599149386954389	-
6778595	Ramp3	0.361	0.599149386954389	-
6792501	Evpl	0.361	0.599149386954389	-
6792839	Sectm1a	0.361	0.599149386954389	-
6793283	Osr1	0.361	0.599149386954389	-
6803192	Prima1	0.361	0.599149386954389	-
6804033	Wdr60	0.361	0.599149386954389	-
6837582	Wnt7b	0.361	0.599149386954389	-

6838666	Krt72-ps	0.361	0.599149386954389	-
6855051	C4b	0.361	0.599149386954389	-
6855060	Skiv2l	0.361	0.599149386954389	-
6861628	Zfp532	0.361	0.599149386954389	-
6869522	Cyp26a1	0.361	0.599149386954389	-
6894303	Znf512b	0.361	0.599149386954389	-
6895835	Lrrcc1	0.361	0.599149386954389	-
6912517	Rragd	0.361	0.599149386954389	-
6919013	Gpr153	0.361	0.599149386954389	-
6925936	Arid1a	0.361	0.599149386954389	-
6926975	Tmem201	0.361	0.599149386954389	-
6941897	Eif2b1	0.361	0.599149386954389	-
6961980	Ttll13	0.361	0.599149386954389	-
6963384	D930014E17Rik	0.361	0.599149386954389	-
6964285	Mylpf	0.361	0.599149386954389	-
6974830	Erlin2	0.361	0.599149386954389	-
6983183	Upf1	0.361	0.599149386954389	-
6984572	Slc38a7	0.361	0.599149386954389	-
6985086	Cog8	0.361	0.599149386954389	-
6996275	Dis3l	0.361	0.599149386954389	-
7014889	Rai2	0.361	0.599149386954389	-
7016555	Odz1	0.361	0.599149386954389	-
7019492	Sytl4	0.361	0.599149386954389	-
6781456	Llgl1	0.362	0.599149386954389	-
6787035	Stc2	0.362	0.599149386954389	-
6791902	Smarcd2	0.362	0.599149386954389	-
6792368	Cd300c	0.362	0.599149386954389	-
6796196	Plekhg3	0.362	0.599149386954389	-
6796299	Gphn	0.362	0.599149386954389	-
6800974	Nkx2-1	0.362	0.599149386954389	-
6820330	Cpb2	0.362	0.599149386954389	-
6823041	Camk2g	0.362	0.599149386954389	-
6824864	Mcpt8	0.362	0.599149386954389	-
6850268	Zfp57	0.362	0.599149386954389	-
6856087	4921523A10Rik	0.362	0.599149386954389	-
6885708	Trub2	0.362	0.599149386954389	-
6900360	Gnat2	0.362	0.599149386954389	-
6904320	Il21	0.362	0.599149386954389	-
6909794	Gm5105	0.362	0.599149386954389	-
6916611	Hectd3	0.362	0.599149386954389	-
6919208	Sdf4	0.362	0.599149386954389	-
6932190	Csn3	0.362	0.599149386954389	-
6933998	Oas1f	0.362	0.599149386954389	-
6942892	E130309D02Rik	0.362	0.599149386954389	-
6942956	Tecpr1	0.362	0.599149386954389	-
6956497	Oxtr	0.362	0.599149386954389	-
6956950	Ccdc77	0.362	0.599149386954389	-
6978736	Cmtm3	0.362	0.599149386954389	-
6992228	Hyal3	0.362	0.599149386954389	-
6760911	D1Ertd622e	0.363	0.599149386954389	-

6767345	Ddo	0.363	0.599149386954389	-
6771718	Itga7	0.363	0.599149386954389	-
6772231	Pex3	0.363	0.599149386954389	-
6782553	Blmh	0.363	0.599149386954389	-
6783338	Mpo	0.363	0.599149386954389	-
6796184	Hspa2	0.363	0.599149386954389	-
6798258	A530016L24Rik	0.363	0.599149386954389	-
6798388	Zfp386	0.363	0.599149386954389	+
6803161	Lgmn	0.363	0.599149386954389	-
6810255	Ankrd55	0.363	0.599149386954389	-
6818858	Mudeng	0.363	0.599149386954389	-
6819866	Zfp395	0.363	0.599149386954389	-
6831538	Ly6e	0.363	0.599149386954389	-
6838331	Endou	0.363	0.599149386954389	-
6866936	Ctdp1	0.363	0.599149386954389	-
6870740	1810018F18Rik	0.363	0.599149386954389	-
6879643	Fbxo3	0.363	0.599149386954389	-
6883344	Ptpn1	0.363	0.599149386954389	-
6883526	Pfdn4	0.363	0.599149386954389	-
6884295	Oprl1	0.363	0.599149386954389	-
6888133	Pde1a	0.363	0.599149386954389	-
6890257	Ltk	0.363	0.599149386954389	-
6890397	Serinc4	0.363	0.599149386954389	-
6898171	Mfsd1	0.363	0.599149386954389	-
6899144	Ubqln4	0.363	0.599149386954389	-
6901023	Fabp2	0.363	0.599149386954389	-
6915905	Oma1	0.363	0.599149386954389	-
6919396	Cyp7a1	0.363	0.599149386954389	-
6921131	B230312A22Rik	0.363	0.599149386954389	-
6931377	Slc30a9	0.363	0.599149386954389	-
6932509	Stbd1	0.363	0.599149386954389	-
6939725	Sult1e1	0.363	0.599149386954389	-
6955431	Plxna1	0.363	0.599149386954389	-
6966292	Arhgap33	0.363	0.599149386954389	-
6966303	Etv2	0.363	0.599149386954389	-
6966922	Prmt1	0.363	0.599149386954389	-
6985249	Dhx38	0.363	0.599149386954389	-
6996667	Lipc	0.363	0.599149386954389	-
6998231	Cldn18	0.363	0.599149386954389	-
7014308	Alg13	0.363	0.599149386954389	-
7020020	Lhfpl1	0.363	0.599149386954389	-
6749044	AI597479	0.364	0.599149386954389	-
6754608	Myoc	0.364	0.599149386954389	-
6780856	Col23a1	0.364	0.599149386954389	-
6782248	Mybbp1a	0.364	0.599149386954389	-
6783674	Lrrc59	0.364	0.599149386954389	-
6788401	Slc36a2	0.364	0.599149386954389	-
6792708	Nptx1	0.364	0.599149386954389	-
6794430	Snx13	0.364	0.599149386954389	-
6824825	Fam158a	0.364	0.599149386954389	-

6833146	Prph	0.364	0.599149386954389	-
6844100	--	0.364	0.599149386954389	-
6870980	Tbc1d10c	0.364	0.599149386954389	-
6878511	Dnajc10	0.364	0.599149386954389	-
6880337	Fam98b	0.364	0.599149386954389	-
6884446	Optn	0.364	0.599149386954389	-
6886953	Cytip	0.364	0.599149386954389	-
6891454	Tasp1	0.364	0.599149386954389	-
6921916	Ctnnal1	0.364	0.599149386954389	-
6929366	Galnt11	0.364	0.599149386954389	-
6941029	Pxmp2	0.364	0.599149386954389	-
6949535	Il17ra	0.364	0.599149386954389	-
6953591	Hoxa1	0.364	0.599149386954389	-
6970735	Sox6	0.364	0.599149386954389	-
6970904	Thumpd1	0.364	0.599149386954389	-
6971949	Tcerg1l	0.364	0.599149386954389	-
6757432	1110058L19Rik	0.365	0.599149386954389	-
6763623	Fmo1	0.365	0.599149386954389	-
6764401	Cep170	0.365	0.599149386954389	-
6767760	Ascc3	0.365	0.599149386954389	-
6770908	Ptprb	0.365	0.599149386954389	-
6777073	Glipr1	0.365	0.599149386954389	-
6782697	Unc119	0.365	0.599149386954389	-
6788019	Maml1	0.365	0.599149386954389	-
6806544	Hivep1	0.365	0.599149386954389	-
6806770	Gm1574	0.365	0.599149386954389	-
6813094	Susd3	0.365	0.599149386954389	-
6815792	Ipo11	0.365	0.599149386954389	-
6826029	Gtf2f2	0.365	0.599149386954389	-
6831527	Psca	0.365	0.599149386954389	-
6835806	Mrpl13	0.365	0.599149386954389	-
6837095	Baiap2l2	0.365	0.599149386954389	-
6838695	Krt78	0.365	0.599149386954389	-
6839320	Rpl39l	0.365	0.599149386954389	-
6916154	Slc1a7	0.365	0.599149386954389	-
6949586	Tuba8	0.365	0.599149386954389	-
6977707	Rfx1	0.365	0.599149386954389	-
6979064	Ap1g1	0.365	0.599149386954389	-
6988624	Dpagt1	0.365	0.599149386954389	-
6993481	Folr4	0.365	0.599149386954389	-
7020664	Grpr	0.365	0.599149386954389	-
6748645	Cnnm4	0.366	0.599149386954389	-
6769137	Gzmm	0.366	0.599149386954389	-
6780797	Hnrnph1	0.366	0.599149386954389	-
6786914	Ccdc85a	0.366	0.599149386954389	+
6791171	Tbx21	0.366	0.599149386954389	-
6798629	A830093I24Rik	0.366	0.599149386954389	-
6805582	Prl3a1	0.366	0.599149386954389	-
6823223	Zfp503	0.366	0.599149386954389	-
6836366	St3gal1	0.366	0.599149386954389	-

6850537	Cyp39a1	0.366	0.599149386954389	-
6856204	Sema6b	0.366	0.599149386954389	-
6864763	Arap3	0.366	0.599149386954389	-
6870978	Rps6kb2	0.366	0.599149386954389	-
6871273	Vegfb	0.366	0.599149386954389	-
6890119	--	0.366	0.599149386954389	-
6899413	Pglyrp3	0.366	0.599149386954389	-
6900226	Adora3	0.366	0.599149386954389	-
6901671	Emcn	0.366	0.599149386954389	-
6909871	Rap1gds1	0.366	0.599149386954389	-
6911729	Cdh17	0.366	0.599149386954389	-
6916090	Ttc22	0.366	0.599149386954389	-
6921158	E130306D19Rik	0.366	0.599149386954389	-
6924878	Kdm4a	0.366	0.599149386954389	-
6948003	Tmem43	0.366	0.599149386954389	-
6959270	Nlrp9a	0.366	0.599149386954389	-
6967584	Gabrg3	0.366	0.599149386954389	-
6977761	Gadd45gip1	0.366	0.599149386954389	-
6983873	Nfix	0.366	0.599149386954389	-
6994356	Aplp2	0.366	0.599149386954389	-
6760620	Asb18	0.367	0.599149386954389	-
6768065	Hsf2	0.367	0.599149386954389	-
6782256	Ankfy1	0.367	0.599149386954389	-
6788388	Anxa6	0.367	0.599149386954389	+
6791236	Stac2	0.367	0.599149386954389	-
6806610	Sirt5	0.367	0.599149386954389	-
6831651	Cyc1	0.367	0.599149386954389	-
6836815	Sharpin	0.367	0.599149386954389	-
6840828	Gsk3b	0.367	0.599149386954389	-
6840837	Cox17	0.367	0.599149386954389	-
6848214	C2cd2	0.367	0.599149386954389	-
6855754	Guca1a	0.367	0.599149386954389	-
6867956	Stx5a	0.367	0.599149386954389	-
6873243	Cyp2c44	0.367	0.599149386954389	-
6881341	Crls1	0.367	0.599149386954389	-
6891689	6330439K17Rik	0.367	0.599149386954389	-
6892493	Ndrg3	0.367	0.599149386954389	-
6899221	Krtcap2	0.367	0.599149386954389	-
6928742	Abcb4	0.367	0.599149386954389	-
6934634	Gbas	0.367	0.599149386954389	-
6956740	Mbd4	0.367	0.599149386954389	-
6965906	Grik5	0.367	0.599149386954389	-
6980090	Clec4g	0.367	0.599149386954389	-
6994937	Tecta	0.367	0.599149386954389	-
7013201	Lpar4	0.367	0.599149386954389	-
6781795	Zkscan6	0.368	0.599149386954389	-
6786504	Aftph	0.368	0.599149386954389	-
6790012	Lgals9	0.368	0.599149386954389	-
6791748	Lyzl6	0.368	0.599149386954389	-
6791992	Nol11	0.368	0.599149386954389	-

6796784	Ahsa1	0.368	0.599149386954389	-
6802364	Prox2	0.368	0.599149386954389	-
6825657	Rhobtb2	0.368	0.599149386954389	-
6834870	Hrsp12	0.368	0.599149386954389	-
6839919	Klhl24	0.368	0.599149386954389	-
6859428	Galnt1	0.368	0.599149386954389	-
6864593	Slc23a1	0.368	0.599149386954389	-
6867917	2700081O15Rik	0.368	0.599149386954389	-
6869798	Zfyve27	0.368	0.599149386954389	-
6875638	Il1f6	0.368	0.599149386954389	-
6889273	Ehf	0.368	0.599149386954389	-
6890363	Tgm5	0.368	0.599149386954389	-
6891493	Flrt3	0.368	0.599149386954389	-
6903183	Pmp2	0.368	0.599149386954389	-
6922966	Bnc2	0.368	0.599149386954389	-
6932197	4931407G18Rik	0.368	0.599149386954389	-
6933987	Ddx54	0.368	0.599149386954389	-
6945114	Cpa1	0.368	0.599149386954389	-
6949962	D6Wsu163e	0.368	0.599149386954389	-
6960175	Klk10	0.368	0.599149386954389	-
6972193	Cdhr5	0.368	0.599149386954389	-
6972194	Sct	0.368	0.599149386954389	-
6976980	Mef2b	0.368	0.599149386954389	-
6981314	Brf2	0.368	0.599149386954389	+
6998900	Tdgf1	0.368	0.599149386954389	-
7011403	Htatsf1	0.368	0.599149386954389	-
6756536	Plxna2	0.369	0.599149386954389	-
6760306	Slc16a14	0.369	0.599149386954389	-
6773918	Gprc6a	0.369	0.599149386954389	-
6780746	Flt4	0.369	0.599149386954389	-
6781090	Gpx3	0.369	0.599149386954389	-
6789525	Med31	0.369	0.599149386954389	-
6805606	Prl4a1	0.369	0.599149386954389	-
6830401	Med30	0.369	0.599149386954389	-
6830477	Colec10	0.369	0.599149386954389	-
6838332	Rapgef3	0.369	0.599149386954389	-
6845540	Nr1i2	0.369	0.599149386954389	-
6850564	Clic5	0.369	0.599149386954389	-
6856203	Lrg1	0.369	0.599149386954389	-
6858637	Rab18	0.369	0.599149386954389	-
6867983	Mta2	0.369	0.599149386954389	-
6877297	Gpd2	0.369	0.599149386954389	-
6895959	Mtfr1	0.369	0.599149386954389	-
6921151	Fam166b	0.369	0.599149386954389	-
6925080	Smap2	0.369	0.599149386954389	-
6959584	Tyrobp	0.369	0.599149386954389	-
6963211	Fxc1	0.369	0.599149386954389	-
6963540	Micalcl	0.369	0.599149386954389	-
6977031	Pde4c	0.369	0.599149386954389	-
6979693	Zfp276	0.369	0.599149386954389	-

6998554	Glyctk	0.369	0.599149386954389	-
6998818	Camp	0.369	0.599149386954389	-
6749720	Nif3l1	0.37	0.599149386954389	-
6752994	Fcamr	0.37	0.599149386954389	-
6775300	Theg	0.37	0.599149386954389	-
6782918	Ccl12	0.37	0.599149386954389	-
6790580	Epx	0.37	0.599149386954389	-
6845147	Pigx	0.37	0.599149386954389	-
6853780	Igf2r	0.37	0.599149386954389	-
6855084	Aif1	0.37	0.599149386954389	-
6855708	Klc4	0.37	0.599149386954389	-
6865983	Arhgef37	0.37	0.599149386954389	-
6871095	Cst6	0.37	0.599149386954389	-
6922100	Slc46a2	0.37	0.599149386954389	-
6939315	Aasdh	0.37	0.599149386954389	-
6955010	Wbp1	0.37	0.599149386954389	-
6966600	Ccne1	0.37	0.599149386954389	-
6993498	4931406C07Rik	0.37	0.599149386954389	-
6775411	Lmnb2	0.371	0.599149386954389	-
6775414	Slc39a3	0.371	0.599149386954389	-
6780811	Adamts2	0.371	0.599149386954389	-
6783694	Samd14	0.371	0.599149386954389	-
6791918	Icam2	0.371	0.599149386954389	-
6822946	Nr1d2	0.371	0.599149386954389	-
6824641	Chd8	0.371	0.599149386954389	-
6843130	Mrap	0.371	0.599149386954389	-
6843880	Emp2	0.371	0.599149386954389	-
6849303	Pkd1	0.371	0.599149386954389	-
6849476	Ggnbp1	0.371	0.599149386954389	-
6850786	Ccnd3	0.371	0.599149386954389	-
6853910	Phf10	0.371	0.599149386954389	-
6854239	Zfp40	0.371	0.599149386954389	-
6859814	Bin1	0.371	0.599149386954389	-
6866919	1110032A13Rik	0.371	0.599149386954389	-
6882510	Itch	0.371	0.599149386954389	-
6883693	Vapb	0.371	0.599149386954389	-
6887337	Galnt3	0.371	0.599149386954389	-
6890424	Patl2	0.371	0.599149386954389	-
6897486	Lhfp	0.371	0.599149386954389	+
6898630	Fam198b	0.371	0.599149386954389	-
6916836	Rims3	0.371	0.599149386954389	-
6924900	Tmem125	0.371	0.599149386954389	-
6955604	Mrps25	0.371	0.599149386954389	-
6971272	Eif3c	0.371	0.599149386954389	-
6975613	Zdhhc2	0.371	0.599149386954389	-
6979543	Gse1	0.371	0.599149386954389	-
6979856	Disc1	0.371	0.599149386954389	-
6980091	Cd209a	0.371	0.599149386954389	-
6983925	Mylk3	0.371	0.599149386954389	-
6988656	Treh	0.371	0.599149386954389	-

6994887	Ubash3b	0.371	0.599149386954389	-
6996124	Thsd4	0.371	0.599149386954389	-
6998214	Mras	0.371	0.599149386954389	-
7015988	Syn1	0.371	0.599149386954389	-
7016678	Apln	0.371	0.599149386954389	-
6748877	Map4k4	0.372	0.599149386954389	-
6763238	Cep350	0.372	0.599149386954389	-
6789851	Efcab5	0.372	0.599149386954389	-
6793652	Rock2	0.372	0.599149386954389	-
6808326	Spata9	0.372	0.599149386954389	-
6817229	Nkiras1	0.372	0.599149386954389	-
6827978	Zic5	0.372	0.599149386954389	-
6835728	Tnfrsf11b	0.372	0.599149386954389	-
6849766	Abcg1	0.372	0.599149386954389	-
6860516	Spink12	0.372	0.599149386954389	-
6861662	Pmaip1	0.372	0.599149386954389	-
6869932	Scd2	0.372	0.599149386954389	-
6881267	Smox	0.372	0.599149386954389	-
6894187	Gata5	0.372	0.599149386954389	-
6902799	Lrrc40	0.372	0.599149386954389	-
6907787	Dclre1b	0.372	0.599149386954389	-
6919052	BC046331	0.372	0.599149386954389	-
6919089	Ttc34	0.372	0.599149386954389	-
6919932	Osgin2	0.372	0.599149386954389	+
6934660	Rabgef1	0.372	0.599149386954389	-
6939714	Ugt2a3	0.372	0.599149386954389	-
6939719	Ugt2a1	0.372	0.599149386954389	-
6946912	Cd8b1	0.372	0.599149386954389	-
6947929	Zxdc	0.372	0.599149386954389	-
6950170	Klrd1	0.372	0.599149386954389	-
6957687	Gsg1	0.372	0.599149386954389	-
6971291	Ppp4c	0.372	0.599149386954389	-
6975913	Casp3	0.372	0.599149386954389	-
6977444	Ttc29	0.372	0.599149386954389	-
6985363	Fa2h	0.372	0.599149386954389	-
6998398	1300017J02Rik	0.372	0.599149386954389	-
7010871	Sh2d1a	0.372	0.599149386954389	-
6754842	Tada1	0.373	0.599149386954389	-
6757830	Fam168b	0.373	0.599149386954389	-
6774346	Eif4ebp2	0.373	0.599149386954389	-
6785183	Itgb4	0.373	0.599149386954389	-
6791440	Rab5c	0.373	0.599149386954389	-
6806707	Gmpr	0.373	0.599149386954389	+
6824878	Gzmc	0.373	0.599149386954389	-
6832342	Upk3a	0.373	0.599149386954389	-
6832394	Gtse1	0.373	0.599149386954389	-
6839930	Dvl3	0.373	0.599149386954389	-
6868070	Gpr44	0.373	0.599149386954389	-
6887088	Itgb6	0.373	0.599149386954389	-
6893147	B4galt5	0.373	0.599149386954389	-

6907205	Tars2	0.373	0.599149386954389	-
6915856	Pde4b	0.373	0.599149386954389	-
6932217	Enam	0.373	0.599149386954389	-
6933326	Pcgf3	0.373	0.599149386954389	-
6936584	Dnajc2	0.373	0.599149386954389	-
6940792	Zfp644	0.373	0.599149386954389	-
6956738	BC060267	0.373	0.599149386954389	-
6962343	Nox4	0.373	0.599149386954389	-
6963972	Mettl9	0.373	0.599149386954389	-
6964887	Mgmt	0.373	0.599149386954389	-
6968126	Arrdc4	0.373	0.599149386954389	-
6977068	Ankle1	0.373	0.599149386954389	-
6978366	Tepp	0.373	0.599149386954389	-
7011757	Fmr1	0.373	0.599149386954389	-
6748889	Il18r1	0.374	0.599149386954389	-
6754735	Dpt	0.374	0.599149386954389	-
6757357	Rims1	0.374	0.599149386954389	-
6768157	4632428N05Rik	0.374	0.599149386954389	-
6769445	Tcp11l2	0.374	0.599149386954389	-
6769883	Ccdc38	0.374	0.599149386954389	-
6771628	Baz2a	0.374	0.599149386954389	-
6775854	Mybpc1	0.374	0.599149386954389	-
6783057	Synrg	0.374	0.599149386954389	-
6804526	Akr1c19	0.374	0.599149386954389	-
6811074	Gpr137b-ps	0.374	0.599149386954389	-
6824980	Zdhhc20	0.374	0.599149386954389	-
6832351	Fbln1	0.374	0.599149386954389	-
6836767	Top1mt	0.374	0.599149386954389	-
6843834	Usp7	0.374	0.599149386954389	-
6850200	Dhx16	0.374	0.599149386954389	-
6856108	Sult1c2	0.374	0.599149386954389	-
6866112	Fech	0.374	0.599149386954389	-
6885530	Vav2	0.374	0.599149386954389	-
6908800	Abcd3	0.374	0.599149386954389	-
6911833	Runx1t1	0.374	0.599149386954389	-
6916773	Foxj3	0.374	0.599149386954389	-
6917594	Gpatch3	0.374	0.599149386954389	-
6927145	Kcnab2	0.374	0.599149386954389	-
6932615	Bmp2k	0.374	0.599149386954389	-
6939241	Kdr	0.374	0.599149386954389	-
6972882	Prpf31	0.374	0.599149386954389	-
6982289	Odz3	0.374	0.599149386954389	-
6984499	Kifc3	0.374	0.599149386954389	-
6984977	Agrp	0.374	0.599149386954389	-
6989948	Rbpms2	0.374	0.599149386954389	-
6990019	Dapk2	0.374	0.599149386954389	-
6991969	Ky	0.374	0.599149386954389	-
6994710	Tbrg1	0.374	0.599149386954389	-
6999545	Hhatl	0.374	0.599149386954389	-
6747786	Terf1	0.375	0.599149386954389	-

6753280	Rabif	0.375	0.599149386954389	-
6777879	Xrcc6bp1	0.375	0.599149386954389	-
6780771	Mapk9	0.375	0.599149386954389	-
6781607	Fam18b	0.375	0.599149386954389	-
6782814	Rhbdl3	0.375	0.599149386954389	-
6792419	Fdxr	0.375	0.599149386954389	-
6800934	Nfkbia	0.375	0.599149386954389	-
6819893	Chrna2	0.375	0.599149386954389	-
6831162	Tg	0.375	0.599149386954389	-
6855150	Prr3	0.375	0.599149386954389	-
6863973	Slc39a6	0.375	0.599149386954389	-
6867707	Slc29a2	0.375	0.599149386954389	-
6870104	As3mt	0.375	0.599149386954389	-
6871189	Zfpl1	0.375	0.599149386954389	-
6871874	Gcnt1	0.375	0.599149386954389	-
6882462	Cbfa2t2	0.375	0.599149386954389	-
6892190	Dusp15	0.375	0.599149386954389	-
6901757	Eif4e	0.375	0.599149386954389	-
6906564	Fam160a1	0.375	0.599149386954389	-
6907217	Vps45	0.375	0.599149386954389	-
6928846	Sema3d	0.375	0.599149386954389	-
6933514	Hps4	0.375	0.599149386954389	-
6955140	Rab11fip5	0.375	0.599149386954389	-
6957122	Ptpn6	0.375	0.599149386954389	-
6957429	Gm156	0.375	0.599149386954389	-
6957758	Art4	0.375	0.599149386954389	-
6985389	Ldhd	0.375	0.599149386954389	-
6990167	Rora	0.375	0.599149386954389	-
7011949	Abcd1	0.375	0.599149386954389	-
7015746	Cask	0.375	0.599149386954389	-
7018041	Gyk	0.375	0.599149386954389	-
6755896	Dusp10	0.376	0.599149386954389	+
6762804	Rgs18	0.376	0.599149386954389	-
6765443	C030002c11rik	0.376	0.599149386954389	-
6789166	Usp43	0.376	0.599149386954389	-
6789939	Traf4	0.376	0.599149386954389	-
6830354	Utp23	0.376	0.599149386954389	-
6830766	Nsmce2	0.376	0.599149386954389	-
6834745	March6	0.376	0.599149386954389	-
6840613	Snx4	0.376	0.599149386954389	-
6849317	Hs3st6	0.376	0.599149386954389	-
6855176	Rpp21	0.376	0.599149386954389	-
6868055	A430093F15Rik	0.376	0.599149386954389	-
6871295	Otub1	0.376	0.599149386954389	-
6873025	Noc3l	0.376	0.599149386954389	-
6875716	2010317E24Rik	0.376	0.599149386954389	-
6886021	Traf1	0.376	0.599149386954389	-
6900141	Ptpn22	0.376	0.599149386954389	-
6918998	Nol9	0.376	0.599149386954389	-
6987396	Smarca4	0.376	0.599149386954389	-

6995820	Rfpl3s	0.376	0.599149386954389	-
6997513	Pgm3	0.376	0.599149386954389	-
6999645	Cdcp1	0.376	0.599149386954389	-
6760678	Hes6	0.377	0.599149386954389	-
6769887	Ntn4	0.377	0.599149386954389	-
6777164	Trhde	0.377	0.599149386954389	-
6783225	Tbx4	0.377	0.599149386954389	-
6789119	2310004I24Rik	0.377	0.599149386954389	-
6790537	1200011M11Rik	0.377	0.599149386954389	-
6792455	Mif4gd	0.377	0.599149386954389	-
6799841	Cbll1	0.377	0.599149386954389	-
6801677	4930447C04Rik	0.377	0.599149386954389	-
6805462	Gpld1	0.377	0.599149386954389	-
6811473	Gpx5	0.377	0.599149386954389	-
6840357	Hrasls	0.377	0.599149386954389	-
6840623	Slc12a8	0.377	0.599149386954389	-
6840849	Cd80	0.377	0.599149386954389	-
6873204	Hps1	0.377	0.599149386954389	-
6877588	Tbr1	0.377	0.599149386954389	-
6880842	Slc24a5	0.377	0.599149386954389	-
6881191	Vps16	0.377	0.599149386954389	-
6900214	Kcnd3	0.377	0.599149386954389	-
6935490	Arpc1b	0.377	0.599149386954389	-
6942894	Zdhhc4	0.377	0.599149386954389	-
6952175	Wasl	0.377	0.599149386954389	-
6952873	Zc3hav1	0.377	0.599149386954389	-
6957168	Tapbpl	0.377	0.599149386954389	-
6970420	Sbf2	0.377	0.599149386954389	-
6975871	Pdlim3	0.377	0.599149386954389	-
6983692	Gab1	0.377	0.599149386954389	-
6991362	Plscr2	0.377	0.599149386954389	-
6996092	Tmem202	0.377	0.599149386954389	-
6996952	Tmod2	0.377	0.599149386954389	-
7015835	Ndp	0.377	0.599149386954389	-
7016369	Rhox8	0.377	0.599149386954389	-
6755672	Parp1	0.378	0.599149386954389	-
6773494	Fig4	0.378	0.599149386954389	-
6774006	Mcm9	0.378	0.599149386954389	-
6789588	Ctns	0.378	0.599149386954389	-
6791338	Krt23	0.378	0.599149386954389	-
6817926	Dcp1a	0.378	0.599149386954389	-
6826042	1200011I18Rik	0.378	0.599149386954389	-
6831688	Ppp1r16a	0.378	0.599149386954389	-
6843384	Ttc3	0.378	0.599149386954389	-
6844196	Dnm1l	0.378	0.599149386954389	-
6846003	Abhd10	0.378	0.599149386954389	-
6855672	Polh	0.378	0.599149386954389	-
6869893	Abcc2	0.378	0.599149386954389	-
6903185	Fabp4	0.378	0.599149386954389	-
6908361	Rnpc3	0.378	0.599149386954389	-

6924749	Dmbx1	0.378	0.599149386954389	-
6929109	Pion	0.378	0.599149386954389	-
6930845	Slc34a2	0.378	0.599149386954389	-
6931257	Klb	0.378	0.599149386954389	-
6933678	Gcn1l1	0.378	0.599149386954389	-
6940146	Cnot6l	0.378	0.599149386954389	-
6942563	Agfg2	0.378	0.599149386954389	-
6947446	Atp6v1b1	0.378	0.599149386954389	-
6949064	Setd5	0.378	0.599149386954389	-
6950030	Tspan11	0.378	0.599149386954389	-
6958326	Pthlh	0.378	0.599149386954389	-
6959442	Fbxo27	0.378	0.599149386954389	-
6979527	6430548M08Rik	0.378	0.599149386954389	+
6979914	Kcnk1	0.378	0.599149386954389	+
6980606	2610019F03Rik	0.378	0.599149386954389	-
6987153	Slc36a4	0.378	0.599149386954389	-
6988635	Hyou1	0.378	0.599149386954389	-
6996254	Smad3	0.378	0.599149386954389	-
7023072	Eif2s3y	0.378	0.599149386954389	-
6750608	Ankzf1	0.379	0.599149386954389	-
6775240	Aire	0.379	0.599149386954389	-
6790944	Abcc3	0.379	0.599149386954389	-
6791340	Krt39	0.379	0.599149386954389	-
6797274	Ttc8	0.379	0.599149386954389	-
6801335	C79407	0.379	0.599149386954389	-
6819683	Wdfy2	0.379	0.599149386954389	-
6835215	Dpys	0.379	0.599149386954389	-
6851191	Ebi3	0.379	0.599149386954389	-
6854345	Ntn3	0.379	0.599149386954389	-
6857100	BC027072	0.379	0.599149386954389	-
6868149	Patl1	0.379	0.599149386954389	-
6870976	Cabp4	0.379	0.599149386954389	-
6891111	Trmt6	0.379	0.599149386954389	-
6913777	Akap2	0.379	0.599149386954389	-
6917696	D4Wsu53e	0.379	0.599149386954389	-
6940876	Evi5	0.379	0.599149386954389	-
6942455	Rhbdd2	0.379	0.599149386954389	-
6948034	Fgd5	0.379	0.599149386954389	-
6980526	Grtp1	0.379	0.599149386954389	-
6983235	Slc5a5	0.379	0.599149386954389	-
6985859	Mthfsd	0.379	0.599149386954389	-
6993754	Keap1	0.379	0.599149386954389	-
7009872	B630019K06Rik	0.379	0.599149386954389	-
6760009	Serpine2	0.38	0.599149386954389	-
6762314	Lax1	0.38	0.599149386954389	-
6763864	Fmo9	0.38	0.599149386954389	-
6781895	Dhrs7c	0.38	0.599149386954389	-
6782255	Ube2g1	0.38	0.599149386954389	-
6819307	Atp12a	0.38	0.599149386954389	-
6824195	Txndc16	0.38	0.599149386954389	-

6825639	D930020E02Rik	0.38	0.599149386954389	-
6836305	Kcnq3	0.38	0.599149386954389	-
6867854	Sf1	0.38	0.599149386954389	-
6868049	Vwce	0.38	0.599149386954389	-
6871386	Gng3	0.38	0.599149386954389	-
6890699	Kcnip3	0.38	0.599149386954389	+
6900348	Gstm5	0.38	0.599149386954389	-
6923526	Cyp2j5	0.38	0.599149386954389	-
6936987	Hadha	0.38	0.599149386954389	-
6941026	Pgam5	0.38	0.599149386954389	-
6941751	Atp2a2	0.38	0.599149386954389	-
6958948	Ceacam12	0.38	0.599149386954389	-
6961932	5730590G19Rik	0.38	0.599149386954389	-
6973539	Fiz1	0.38	0.599149386954389	-
6988963	Tmprss5	0.38	0.599149386954389	-
6990532	Gnb5	0.38	0.599149386954389	-
6765317	AA408296	0.381	0.599149386954389	-
6774659	Reep3	0.381	0.599149386954389	-
6783005	Slfn4	0.381	0.599149386954389	-
6785368	Card14	0.381	0.599149386954389	-
6787819	Timd2	0.381	0.599149386954389	-
6789360	2810408A11Rik	0.381	0.599149386954389	-
6803314	Dicer1	0.381	0.599149386954389	-
6812603	Tcfap2a	0.381	0.599149386954389	-
6845914	Atp6v1a	0.381	0.599149386954389	-
6855772	Bysl	0.381	0.599149386954389	-
6866054	Fam38b	0.381	0.599149386954389	-
6873369	9130011E15Rik	0.381	0.599149386954389	-
6877952	Ubr3	0.381	0.599149386954389	-
6882727	Bpi	0.381	0.599149386954389	-
6884341	Olah	0.381	0.599149386954389	-
6896591	Zfp639	0.381	0.599149386954389	-
6899016	Arhgef11	0.381	0.599149386954389	-
6923164	Klhl9	0.381	0.599149386954389	-
6925346	Thrap3	0.381	0.599149386954389	-
6935073	Actl6b	0.381	0.599149386954389	-
6935110	Zkscan1	0.381	0.599149386954389	-
6946953	Polr1a	0.381	0.599149386954389	-
6955175	Dusp11	0.381	0.599149386954389	-
6956012	Pdzrn3	0.381	0.599149386954389	-
6965204	Cd151	0.381	0.599149386954389	-
6979652	Acsf3	0.381	0.599149386954389	-
6988971	Gm4894	0.381	0.599149386954389	-
6989422	Cyp1a1	0.381	0.599149386954389	-
6991552	Xrn1	0.381	0.599149386954389	-
6996704	Tcf12	0.381	0.599149386954389	-
6760802	Dtymk	0.382	0.599149386954389	-
6763626	Fmo2	0.382	0.599149386954389	-
6765313	Syt14	0.382	0.599149386954389	-
6767057	Trdn	0.382	0.599149386954389	-

6779714	Ccdc88a	0.382	0.599149386954389	-
6783626	Mbtd1	0.382	0.599149386954389	-
6792658	Cant1	0.382	0.599149386954389	-
6801488	L2hgdh	0.382	0.599149386954389	-
6823710	Nisch	0.382	0.599149386954389	-
6824286	Cnih	0.382	0.599149386954389	-
6824779	Slc22a17	0.382	0.599149386954389	-
6838648	Krt82	0.382	0.599149386954389	-
6841160	Ccdc80	0.382	0.599149386954389	-
6848939	BC018101	0.382	0.599149386954389	-
6849968	H2-Eb1	0.382	0.599149386954389	-
6855210	Trim15	0.382	0.599149386954389	-
6871206	Ppp2r5b	0.382	0.599149386954389	-
6887082	Pla2r1	0.382	0.599149386954389	-
6887187	Gcg	0.382	0.599149386954389	-
6924246	Ttc4	0.382	0.599149386954389	-
6930606	Slit2	0.382	0.599149386954389	-
6939320	Ppat	0.382	0.599149386954389	-
6953615	Hoxa10	0.382	0.599149386954389	-
6959459	Ech1	0.382	0.599149386954389	-
6966324	Mag	0.382	0.599149386954389	-
6966895	Zfp473	0.382	0.599149386954389	-
6968780	Fes	0.382	0.599149386954389	-
6969094	Folh1	0.382	0.599149386954389	+
6982267	Wwc2	0.382	0.599149386954389	-
6994672	Pknox2	0.382	0.599149386954389	-
6996678	Aqp9	0.382	0.599149386954389	-
7012840	Arr3	0.382	0.599149386954389	-
6763937	Pbx1	0.383	0.599149386954389	-
6775279	Casp14	0.383	0.599149386954389	-
6780707	Olfr56	0.383	0.599149386954389	-
6785664	Gm11961	0.383	0.599149386954389	-
6791558	Ubtf	0.383	0.599149386954389	-
6802745	Ston2	0.383	0.599149386954389	-
6808199	Irx4	0.383	0.599149386954389	-
6815111	Jmy	0.383	0.599149386954389	-
6825875	Rb1	0.383	0.599149386954389	-
6848877	Chd1	0.383	0.599149386954389	-
6850056	Pbx2	0.383	0.599149386954389	-
6854387	Rnf151	0.383	0.599149386954389	-
6877428	Pkp4	0.383	0.599149386954389	-
6879039	Pacsin3	0.383	0.599149386954389	-
6899106	0610031J06Rik	0.383	0.599149386954389	-
6903190	Impa1	0.383	0.599149386954389	-
6915847	Lepr	0.383	0.599149386954389	-
6932372	Cxcl15	0.383	0.599149386954389	-
6957138	Leprel2	0.383	0.599149386954389	-
6969121	Tyr	0.383	0.599149386954389	-
6985361	Mlkl	0.383	0.599149386954389	-
6754137	Rgs8	0.384	0.599149386954389	+

6759758	Prkag3	0.384	0.599149386954389	-
6766381	Fam54a	0.384	0.599149386954389	-
6766863	Echdc1	0.384	0.599149386954389	-
6778609	Igfbp1	0.384	0.599149386954389	-
6779832	Snrnp25	0.384	0.599149386954389	-
6788423	Glra1	0.384	0.599149386954389	-
6828492	Rictor	0.384	0.599149386954389	-
6837018	Tmprss6	0.384	0.599149386954389	-
6854914	Zfp763	0.384	0.599149386954389	+
6864678	Pfdn1	0.384	0.599149386954389	-
6880540	Itpka	0.384	0.599149386954389	-
6911212	Rusc1	0.384	0.599149386954389	-
6926341	Pqlc2	0.384	0.599149386954389	-
6934321	Gtf2h3	0.384	0.599149386954389	-
6936564	Ptpn12	0.384	0.599149386954389	+
6946744	C130060K24Rik	0.384	0.599149386954389	-
6953587	Skap2	0.384	0.599149386954389	-
6956488	D630042P16Rik	0.384	0.599149386954389	-
6960562	Zdhhc13	0.384	0.599149386954389	-
6981182	Hgsnat	0.384	0.599149386954389	-
6987470	Bbs9	0.384	0.599149386954389	-
6762425	Ipo9	0.385	0.599149386954389	-
6764289	Ifi202b	0.385	0.599149386954389	-
6769235	Csnk1g2	0.385	0.599149386954389	-
6769934	Ccdc41	0.385	0.599149386954389	-
6777510	Irak3	0.385	0.599149386954389	-
6786571	4932414J04Rik	0.385	0.599149386954389	-
6789450	Inca1	0.385	0.599149386954389	-
6789517	Aipl1	0.385	0.599149386954389	-
6789830	Cpd	0.385	0.599149386954389	-
6798218	Mark3	0.385	0.599149386954389	-
6833157	Prpf40b	0.385	0.599149386954389	-
6838376	Lalba	0.385	0.599149386954389	-
6848504	Snx9	0.385	0.599149386954389	-
6856210	Dpp9	0.385	0.599149386954389	-
6877940	Ubr3	0.385	0.599149386954389	-
6890151	A430105I19Rik	0.385	0.599149386954389	-
6890288	Ehd4	0.385	0.599149386954389	-
6900193	Rhoc	0.385	0.599149386954389	-
6929688	Snx17	0.385	0.599149386954389	-
6946277	Tax1bp1	0.385	0.599149386954389	-
6953101	Kel	0.385	0.599149386954389	-
6970851	2310008H09Rik	0.385	0.599149386954389	-
6978386	Gins3	0.385	0.599149386954389	-
6981190	Hook3	0.385	0.599149386954389	-
6990774	Myo6	0.385	0.599149386954389	-
6995536	4833427G06Rik	0.385	0.599149386954389	-
6996018	Neo1	0.385	0.599149386954389	-
6998286	Nck1	0.385	0.599149386954389	-
6755323	Fmn2	0.386	0.599149386954389	-

6765332	Camk1g	0.386	0.599149386954389	-
6767537	Cd24a	0.386	0.599149386954389	-
6771575	Stat6	0.386	0.599149386954389	-
6779955	Kcnmb1	0.386	0.599149386954389	-
6783886	Osbpl7	0.386	0.599149386954389	-
6788017	Ltc4s	0.386	0.599149386954389	-
6791150	Cdk5rap3	0.386	0.599149386954389	-
6791275	Med24	0.386	0.599149386954389	-
6796707	Ttll5	0.386	0.599149386954389	-
6833326	Krt7	0.386	0.599149386954389	-
6840676	Ropn1	0.386	0.599149386954389	-
6866299	Afg3l2	0.386	0.599149386954389	-
6870697	Atrnl1	0.386	0.599149386954389	-
6891064	Rassf2	0.386	0.599149386954389	-
6901595	Bdh2	0.386	0.599149386954389	-
6908048	Csf1	0.386	0.599149386954389	-
6920290	Manea	0.386	0.599149386954389	-
6936990	Gpr113	0.386	0.599149386954389	-
6940333	Tmem150c	0.386	0.599149386954389	-
6941157	Sgsm1	0.386	0.599149386954389	-
6941704	Sh2b3	0.386	0.599149386954389	-
6945786	Zyx	0.386	0.599149386954389	-
6958945	Ceacam13	0.386	0.599149386954389	-
6966028	Mia1	0.386	0.599149386954389	-
6974261	Myom2	0.386	0.599149386954389	-
6979073	Tat	0.386	0.599149386954389	-
6979519	Usp10	0.386	0.599149386954389	-
6985355	Glg1	0.386	0.599149386954389	-
6988713	Scn2b	0.386	0.599149386954389	-
7009780	Gpkow	0.386	0.599149386954389	-
6748980	Pou3f3	0.387	0.599149386954389	-
6757278	Il17f	0.387	0.599149386954389	-
6766605	Moxd1	0.387	0.599149386954389	-
6767581	Atg5	0.387	0.599149386954389	-
6767850	Slc35f1	0.387	0.599149386954389	-
6778420	Thoc5	0.387	0.599149386954389	-
6791470	Plekhh3	0.387	0.599149386954389	-
6800912	2700097O09Rik	0.387	0.599149386954389	-
6817287	Nid2	0.387	0.599149386954389	-
6845141	Senp5	0.387	0.599149386954389	-
6849416	Solh	0.387	0.599149386954389	-
6850645	Nfkbie	0.387	0.599149386954389	-
6855722	Cnpy3	0.387	0.599149386954389	-
6866116	Nars	0.387	0.599149386954389	-
6867761	Efemp2	0.387	0.599149386954389	-
6869582	Cyp2c29	0.387	0.599149386954389	-
6872615	Dkk1	0.387	0.599149386954389	-
6875602	Acbd5	0.387	0.599149386954389	-
6885351	Arrdc1	0.387	0.599149386954389	-
6890908	Fam113a	0.387	0.599149386954389	-

6902192	Ssx2ip	0.387	0.599149386954389	-
6933852	Med13l	0.387	0.599149386954389	-
6934053	Naa25	0.387	0.599149386954389	-
6937255	Tmem129	0.387	0.599149386954389	-
6942847	Tnrc18	0.387	0.599149386954389	-
6943889	Dync1i1	0.387	0.599149386954389	-
6959274	Cyp2g1	0.387	0.599149386954389	-
6976997	Comp	0.387	0.599149386954389	-
6998757	Fbxw19	0.387	0.599149386954389	-
7016693	Zdhhc9	0.387	0.599149386954389	-
6750557	Bcs1l	0.388	0.599149386954389	-
6786673	Rel	0.388	0.599149386954389	-
6789696	Srr	0.388	0.599149386954389	-
6792931	Kif3c	0.388	0.599149386954389	-
6798429	Cdca7l	0.388	0.599149386954389	-
6835706	Samd12	0.388	0.599149386954389	-
6839934	Abcf3	0.388	0.599149386954389	-
6847948	Kcne1	0.388	0.599149386954389	-
6870675	Fam160b1	0.388	0.599149386954389	-
6880517	1500003O03Rik	0.388	0.599149386954389	-
6880906	Dtwd1	0.388	0.599149386954389	-
6885325	Hnmt	0.388	0.599149386954389	+
6888763	1110051M20Rik	0.388	0.599149386954389	-
6901813	B930007M17Rik	0.388	0.599149386954389	-
6907222	BC028528	0.388	0.599149386954389	-
6917506	Med18	0.388	0.599149386954389	-
6923394	Lrrc19	0.388	0.599149386954389	-
6926084	Nipal3	0.388	0.599149386954389	-
6927086	Vamp3	0.388	0.599149386954389	-
6943387	N4bp2l1	0.388	0.599149386954389	-
6961795	Agbl1	0.388	0.599149386954389	-
6976976	Hapln4	0.388	0.599149386954389	-
6989052	Btg4	0.388	0.599149386954389	-
6998121	Trim42	0.388	0.599149386954389	-
7018500	Awat2	0.388	0.599149386954389	-
6750433	Smarcal1	0.389	0.599149386954389	-
6756599	Sox17	0.389	0.599149386954389	-
6763894	Uck2	0.389	0.599149386954389	-
6778027	Slc39a5	0.389	0.599149386954389	-
6788134	Sec24a	0.389	0.599149386954389	-
6801332	Fkbp3	0.389	0.599149386954389	-
6803524	Bcl11b	0.389	0.599149386954389	-
6836810	Oplah	0.389	0.599149386954389	-
6847823	Gcfc1	0.389	0.599149386954389	-
6849645	Tbc1d22b	0.389	0.599149386954389	-
6876205	Fam102a	0.389	0.599149386954389	-
6880556	Pla2g4b	0.389	0.599149386954389	-
6890918	Fastkd5	0.389	0.599149386954389	-
6917936	Usp48	0.389	0.599149386954389	-
6920732	Slc35a1	0.389	0.599149386954389	-

6929714	Bre	0.389	0.599149386954389	-
6940008	Ccdc158	0.389	0.599149386954389	-
6941650	Oas1c	0.389	0.599149386954389	-
6979023	Zfhx3	0.389	0.599149386954389	-
6980119	BC068157	0.389	0.599149386954389	-
6993708	Fbxl12	0.389	0.599149386954389	-
7019641	Glra4	0.389	0.599149386954389	-
6763682	Scyl3	0.39	0.599149386954389	-
6778410	Ascc2	0.39	0.599149386954389	-
6779694	Smek2	0.39	0.599149386954389	-
6785530	Tex19.1	0.39	0.599149386954389	-
6797474	Cpsf2	0.39	0.599149386954389	-
6805106	Tcrg-V5	0.39	0.599149386954389	-
6823460	Pde12	0.39	0.599149386954389	-
6833972	Brix1	0.39	0.599149386954389	-
6838578	Cela1	0.39	0.599149386954389	-
6849297	Caskin1	0.39	0.599149386954389	-
6853779	Slc22a1	0.39	0.599149386954389	-
6854347	Ccnf	0.39	0.599149386954389	-
6854430	Chtf18	0.39	0.599149386954389	-
6855123	Ddr1	0.39	0.599149386954389	-
6858633	4921524L21Rik	0.39	0.599149386954389	-
6865643	Zfp608	0.39	0.599149386954389	-
6867776	Rela	0.39	0.599149386954389	+
6867925	Atl3	0.39	0.599149386954389	-
6867955	Wdr74	0.39	0.599149386954389	-
6884441	Mcm10	0.39	0.599149386954389	-
6900089	Csde1	0.39	0.599149386954389	-
6925885	Wdtc1	0.39	0.599149386954389	-
6945275	Exoc4	0.39	0.599149386954389	-
6947936	Aldh1l1	0.39	0.599149386954389	-
6961396	Zic4	0.39	0.599149386954389	-
6965781	Klc3	0.39	0.599149386954389	-
6969009	1700026D08Rik	0.39	0.599149386954389	-
6970890	Gp2	0.39	0.599149386954389	-
6971261	Spns1	0.39	0.599149386954389	-
6978727	Cklf	0.39	0.599149386954389	-
6981683	Eri1	0.39	0.599149386954389	-
7012831	Igbp1	0.39	0.599149386954389	-
7017601	Arhgap4	0.39	0.599149386954389	-
6749736	Casp8	0.391	0.599149386954389	-
6768910	S100b	0.391	0.599149386954389	-
6769080	Cstb	0.391	0.599149386954389	+
6769159	Cfd	0.391	0.599149386954389	-
6775838	Dram1	0.391	0.599149386954389	-
6801838	Sgpp1	0.391	0.599149386954389	-
6813394	F12	0.391	0.599149386954389	-
6820372	Nufip1	0.391	0.599149386954389	-
6822317	Zic2	0.391	0.599149386954389	-
6849923	C920016K16Rik	0.391	0.599149386954389	-

6862922	Cd226	0.391	0.599149386954389	-
6868728	Apba1	0.391	0.599149386954389	-
6880529	Nusap1	0.391	0.599149386954389	-
6890210	Rhov	0.391	0.599149386954389	-
6892138	Rbck1	0.391	0.599149386954389	-
6894159	Hrh3	0.391	0.599149386954389	-
6906634	Dclk2	0.391	0.599149386954389	-
6908528	Palmd	0.391	0.599149386954389	-
6918808	Ctnnbip1	0.391	0.599149386954389	-
6924181	Prkaa2	0.391	0.599149386954389	-
6932821	Enoph1	0.391	0.599149386954389	-
6935402	Daglb	0.391	0.599149386954389	-
6941398	Suds3	0.391	0.599149386954389	-
6947390	Lbx2	0.391	0.599149386954389	-
6952470	AB041803	0.391	0.599149386954389	-
6965163	Ptdss2	0.391	0.599149386954389	-
6967096	Spty2d1	0.391	0.599149386954389	-
6989922	Clpx	0.391	0.599149386954389	-
6998829	Dhx30	0.391	0.599149386954389	-
6753250	Mybph	0.392	0.599149386954389	-
6755158	Mpz	0.392	0.599149386954389	-
6759748	Zfp142	0.392	0.599149386954389	-
6766158	Nmbr	0.392	0.599149386954389	-
6779359	Cct4	0.392	0.599149386954389	-
6791233	Cacnb1	0.392	0.599149386954389	-
6792768	Pde6g	0.392	0.599149386954389	-
6794648	Immp2l	0.392	0.599149386954389	-
6799879	Prkar2b	0.392	0.599149386954389	-
6803020	Kcnk10	0.392	0.599149386954389	-
6820057	Bin3	0.392	0.599149386954389	-
6832259	Tspo	0.392	0.599149386954389	-
6849364	Tpsb2	0.392	0.599149386954389	-
6850661	Mrpl14	0.392	0.599149386954389	-
6854310	Prss30	0.392	0.599149386954389	-
6854983	H2-Ke2	0.392	0.599149386954389	-
6861175	Prrc1	0.392	0.599149386954389	-
6877077	Tnfaip6	0.392	0.599149386954389	-
6882068	Cst8	0.392	0.599149386954389	-
6890402	Frmd5	0.392	0.599149386954389	-
6912092	Ccnc	0.392	0.599149386954389	-
6912931	Dnaic1	0.392	0.599149386954389	-
6917755	4930555I21Rik	0.392	0.599149386954389	-
6942087	Tmem132d	0.392	0.599149386954389	-
6942413	Wbscr22	0.392	0.599149386954389	-
6946900	Fabp1	0.392	0.599149386954389	-
6953100	Trpv5	0.392	0.599149386954389	-
6953639	Jazf1	0.392	0.599149386954389	-
6965786	Mark4	0.392	0.599149386954389	-
6978843	Fam65a	0.392	0.599149386954389	-
6983325	Eps15l1	0.392	0.599149386954389	-

7020797	Tmsb4x	0.392	0.599149386954389	-
6771593	Rdh19	0.393	0.599149386954389	-
6775309	Polrmt	0.393	0.599149386954389	-
6788674	Zfp867	0.393	0.599149386954389	-
6801821	Gphb5	0.393	0.599149386954389	-
6806036	Serpinb9	0.393	0.599149386954389	-
6806963	Iars	0.393	0.599149386954389	-
6813411	Fam193b	0.393	0.599149386954389	-
6814956	Vcan	0.393	0.599149386954389	-
6815560	Slc30a5	0.393	0.599149386954389	-
6856196	Sh3gl1	0.393	0.599149386954389	-
6871119	Mus81	0.393	0.599149386954389	-
6871156	Frmd8	0.393	0.599149386954389	-
6882556	Mmp24	0.393	0.599149386954389	-
6892193	Pdrg1	0.393	0.599149386954389	-
6910122	Sh3glb1	0.393	0.599149386954389	-
6916722	Ebna1bp2	0.393	0.599149386954389	-
6929604	Ept1	0.393	0.599149386954389	-
6929865	Hgfac	0.393	0.599149386954389	-
6932385	Epgn	0.393	0.599149386954389	-
6936874	Cnpy1	0.393	0.599149386954389	-
6941189	Svop	0.393	0.599149386954389	-
6942441	Hip1	0.393	0.599149386954389	-
6942492	Prkrip1	0.393	0.599149386954389	-
6953561	Hnrnpa2b1	0.393	0.599149386954389	-
6957304	Prmt8	0.393	0.599149386954389	-
6960061	Vstm2b	0.393	0.599149386954389	-
6962780	Gdpd4	0.393	0.599149386954389	-
6964522	Plekha1	0.393	0.599149386954389	-
6985879	Zcchc14	0.393	0.599149386954389	-
6991718	Copb2	0.393	0.599149386954389	-
7020206	Ribc1	0.393	0.599149386954389	-
6750532	Pnkd	0.394	0.599149386954389	-
6780331	Gabrb2	0.394	0.599149386954389	-
6785935	Hus1	0.394	0.599149386954389	-
6803757	1700001K19Rik	0.394	0.599149386954389	-
6818029	Btd	0.394	0.599149386954389	-
6819274	2610027L16Rik	0.394	0.599149386954389	-
6832719	Cntn1	0.394	0.599149386954389	-
6850265	Znrd1as	0.394	0.599149386954389	-
6860787	Hsd17b4	0.394	0.599149386954389	-
6871057	Actn3	0.394	0.599149386954389	-
6883120	Ctsa	0.394	0.599149386954389	-
6885381	Slc34a3	0.394	0.599149386954389	-
6892306	Necab3	0.394	0.599149386954389	-
6895838	Car13	0.394	0.599149386954389	-
6900082	Tspan2	0.394	0.599149386954389	-
6901898	Unc5c	0.394	0.599149386954389	-
6925762	Ptpru	0.394	0.599149386954389	-
6929125	Fam185a	0.394	0.599149386954389	-

6959597	U2af1l4	0.394	0.599149386954389	-
6966138	Supt5h	0.394	0.599149386954389	-
6970137	Hpx	0.394	0.599149386954389	-
6971714	Ctbp2	0.394	0.599149386954389	-
6972505	Clcn4-2	0.394	0.599149386954389	-
6996122	Nr2e3	0.394	0.599149386954389	-
6998907	Rtp3	0.394	0.599149386954389	-
6999527	Lyzl4	0.394	0.599149386954389	-
6749788	Fam117b	0.395	0.599149386954389	-
6750412	Tmem169	0.395	0.599149386954389	-
6773545	Foxo3	0.395	0.599149386954389	-
6778034	Myl6b	0.395	0.599149386954389	-
6782278	1200014J11Rik	0.395	0.599149386954389	-
6789741	Serpinf1	0.395	0.599149386954389	-
6812770	Tbc1d7	0.395	0.599149386954389	+
6852769	Slc3a1	0.395	0.599149386954389	-
6867569	Mtl5	0.395	0.599149386954389	-
6880690	Duoxa2	0.395	0.599149386954389	-
6906645	Kirrel	0.395	0.599149386954389	-
6907624	Cd2	0.395	0.599149386954389	-
6919497	Car8	0.395	0.599149386954389	-
6955381	Mcm2	0.395	0.599149386954389	-
6956558	Cidec	0.395	0.599149386954389	-
6961245	Lysmd4	0.395	0.599149386954389	-
6978223	Irx6	0.395	0.599149386954389	-
7015831	Maob	0.395	0.599149386954389	+
6747696	Xkr9	0.396	0.599149386954389	-
6752068	2310035C23Rik	0.396	0.599149386954389	-
6755154	1700009P17Rik	0.396	0.599149386954389	-
6769180	Abca7	0.396	0.599149386954389	-
6769381	D10Wsu102e	0.396	0.599149386954389	-
6773179	Nt5dc1	0.396	0.599149386954389	-
6775466	Nfic	0.396	0.599149386954389	-
6776872	Syt1	0.396	0.599149386954389	-
6780544	Med7	0.396	0.599149386954389	+
6788653	A230051G13Rik	0.396	0.599149386954389	-
6814257	Irx1	0.396	0.599149386954389	-
6847525	Atp5j	0.396	0.599149386954389	-
6850033	H2-Ab1	0.396	0.599149386954389	-
6869984	Tlx1	0.396	0.599149386954389	-
6887816	Sp3	0.396	0.599149386954389	-
6900197	St7l	0.396	0.599149386954389	-
6905897	B3galnt1	0.396	0.599149386954389	-
6908488	Cdc14a	0.396	0.599149386954389	-
6929671	Cad	0.396	0.599149386954389	-
6935386	Spdyb	0.396	0.599149386954389	-
6941649	Oas3	0.396	0.599149386954389	-
6941713	Ccdc63	0.396	0.599149386954389	-
6942380	Eln	0.396	0.599149386954389	-
6943310	Hsph1	0.396	0.599149386954389	+

6945664	Gm5567	0.396	0.599149386954389	-
6952298	Lrrc4	0.396	0.599149386954389	-
6959131	Xrcc1	0.396	0.599149386954389	-
6961977	Sema4b	0.396	0.599149386954389	-
6968647	Mrpl46	0.396	0.599149386954389	-
6977692	Lphn1	0.396	0.599149386954389	-
6977699	1700067K01Rik	0.396	0.599149386954389	-
6984061	Brd7	0.396	0.599149386954389	-
6986722	Mmp12	0.396	0.599149386954389	-
6992385	Ccdc51	0.396	0.599149386954389	-
6992878	Acaa1a	0.396	0.599149386954389	-
6993762	Ap1m2	0.396	0.599149386954389	-
6995950	Ccdc33	0.396	0.599149386954389	-
6749073	1500015O10Rik	0.397	0.599149386954389	-
6753400	Lad1	0.397	0.599149386954389	-
6768926	Mcm3ap	0.397	0.599149386954389	-
6778083	Mettl7b	0.397	0.599149386954389	-
6781986	Hes7	0.397	0.599149386954389	-
6797742	Bdkrb2	0.397	0.599149386954389	-
6822478	Oit1	0.397	0.599149386954389	-
6823011	Kcnk5	0.397	0.599149386954389	-
6827239	Rbm26	0.397	0.599149386954389	-
6840092	Ahsg	0.397	0.599149386954389	-
6857885	Srbd1	0.397	0.599149386954389	-
6891675	Snx5	0.397	0.599149386954389	-
6909375	Ostc	0.397	0.599149386954389	-
6912099	6230409E13Rik	0.397	0.599149386954389	-
6913982	4933430I17Rik	0.397	0.599149386954389	-
6931321	Nsun7	0.397	0.599149386954389	-
6933965	Lhx5	0.397	0.599149386954389	-
6940820	Hfm1	0.397	0.599149386954389	-
6967908	Fam189a1	0.397	0.599149386954389	-
6991799	Dzip1l	0.397	0.599149386954389	-
6750632	Accn4	0.398	0.599149386954389	-
6753289	Kdm5b	0.398	0.599149386954389	-
6759779	Atg9a	0.398	0.599149386954389	-
6787196	Gabrp	0.398	0.599149386954389	-
6797958	Wdr25	0.398	0.599149386954389	-
6808173	Irx2	0.398	0.599149386954389	-
6823221	Comtd1	0.398	0.599149386954389	-
6823742	Hacl1	0.398	0.599149386954389	-
6836729	Ly6c1	0.398	0.599149386954389	-
6843657	Nmral1	0.398	0.599149386954389	-
6847901	Cryzl1	0.398	0.599149386954389	-
6869974	Sema4g	0.398	0.599149386954389	-
6885506	Surf4	0.398	0.599149386954389	-
6891640	Bfsp1	0.398	0.599149386954389	-
6908077	Gstm3	0.398	0.599149386954389	-
6928889	Pclo	0.398	0.599149386954389	-
6941710	Cux2	0.398	0.599149386954389	-

6942554	Pop7	0.398	0.599149386954389	-
6946138	Mpp6	0.398	0.599149386954389	-
6953887	Pde1c	0.398	0.599149386954389	-
6956552	Tada3	0.398	0.599149386954389	-
6956765	Rassf4	0.398	0.599149386954389	-
6960239	Klk1b22	0.398	0.599149386954389	-
6965947	Cxcl17	0.398	0.599149386954389	-
6966990	Nucb1	0.398	0.599149386954389	-
6985929	Snai3	0.398	0.599149386954389	-
6994383	Barx2	0.398	0.599149386954389	-
7014110	Rnf128	0.398	0.599149386954389	-
7017585	Pnck	0.398	0.599149386954389	+
6762147	Rassf5	0.399	0.599149386954389	-
6763853	Pogk	0.399	0.599149386954389	-
6780895	Cdkl3	0.399	0.599149386954389	-
6780961	Zcchc10	0.399	0.599149386954389	-
6782919	Ccl8	0.399	0.599149386954389	-
6785743	Urgcp	0.399	0.599149386954389	-
6795936	Daam1	0.399	0.599149386954389	-
6831993	Smcr7l	0.399	0.599149386954389	-
6833314	6030408B16Rik	0.399	0.599149386954389	-
6836959	Apol7c	0.399	0.599149386954389	-
6839746	Lztr1	0.399	0.599149386954389	-
6881259	Pank2	0.399	0.599149386954389	-
6883078	Stk4	0.399	0.599149386954389	-
6892209	Plagl2	0.399	0.599149386954389	-
6892371	Ncoa6	0.399	0.599149386954389	-
6893382	Bcas1	0.399	0.599149386954389	-
6909434	Sgms2	0.399	0.599149386954389	-
6932707	Fgf5	0.399	0.599149386954389	-
6941048	Pus1	0.399	0.599149386954389	-
6941345	Prkab1	0.399	0.599149386954389	-
6946044	Abp1	0.399	0.599149386954389	-
6950992	Lyrm5	0.399	0.599149386954389	-
6966935	Fcgrt	0.399	0.599149386954389	-
6971320	Kif22	0.399	0.599149386954389	-
6972328	Th	0.399	0.599149386954389	-
6989349	Cspg4	0.399	0.599149386954389	-
6992062	Acad11	0.399	0.599149386954389	-
6998561	Ppm1m	0.399	0.599149386954389	-
7012730	Ar	0.399	0.599149386954389	-
6774336	Sgpl1	0.4	0.599149386954389	-
6784564	Mrc2	0.4	0.599149386954389	-
6785111	Cd300a	0.4	0.599149386954389	-
6785758	Camk2b	0.4	0.599149386954389	-
6806701	Mylip	0.4	0.599149386954389	-
6808948	Dhfr	0.4	0.599149386954389	-
6819209	Ngdn	0.4	0.599149386954389	-
6836827	Bop1	0.4	0.599149386954389	-
6844093	Pdxdc1	0.4	0.599149386954389	-

6845587	Upk1b	0.4	0.599149386954389	-
6883000	Mybl2	0.4	0.599149386954389	-
6884281	Dnajc5	0.4	0.599149386954389	-
6909310	Ccdc109b	0.4	0.599149386954389	-
6917810	E2f2	0.4	0.599149386954389	-
6926477	Ddi2	0.4	0.599149386954389	+
6935343	Wipi2	0.4	0.599149386954389	-
6945584	Tbxas1	0.4	0.599149386954389	-
6953144	Fam115c	0.4	0.599149386954389	-
6957557	Kap	0.4	0.599149386954389	-
6959386	9530053A07Rik	0.4	0.599149386954389	-
6962773	Clns1a	0.4	0.599149386954389	-
6974254	Arhgef10	0.4	0.599149386954389	-
6980032	Itgb1	0.4	0.599149386954389	-
6988592	Pvrl1	0.4	0.599149386954389	-
6990427	Ccpg1	0.4	0.599149386954389	-
7009786	Tcfe3	0.4	0.599149386954389	-
6767258	Fyn	0.401	0.599149386954389	-
6771533	Mettl1	0.401	0.599149386954389	-
6781925	Pik3r5	0.401	0.599149386954389	-
6796597	Coq6	0.401	0.599149386954389	-
6845044	Lsg1	0.401	0.599149386954389	-
6849665	Ftsjd2	0.401	0.599149386954389	-
6850638	Tcte1	0.401	0.599149386954389	-
6859411	Mapre2	0.401	0.599149386954389	-
6875827	Gm711	0.401	0.599149386954389	-
6879016	Psmc3	0.401	0.599149386954389	-
6933054	Dspp	0.401	0.599149386954389	-
6934001	Oas1h	0.401	0.599149386954389	-
6942276	Wbscr17	0.401	0.599149386954389	-
6954619	Vamp8	0.401	0.599149386954389	-
6958832	Napa	0.401	0.599149386954389	-
6966309	Gapdhs	0.401	0.599149386954389	-
7018756	Magt1	0.401	0.599149386954389	-
7019494	Nox1	0.401	0.599149386954389	-
6783845	Hoxb4	0.402	0.599149386954389	-
6787730	Lsm11	0.402	0.599149386954389	-
6789269	Gucy2e	0.402	0.599149386954389	-
6800711	Strn3	0.402	0.599149386954389	-
6822526	Fhit	0.402	0.599149386954389	-
6825691	Piwil2	0.402	0.599149386954389	-
6834031	Sub1	0.402	0.599149386954389	-
6845971	Slc35a5	0.402	0.599149386954389	-
6862126	BC031181	0.402	0.599149386954389	-
6864895	Nr3c1	0.402	0.599149386954389	+
6870093	Trim8	0.402	0.599149386954389	-
6872063	1110059E24Rik	0.402	0.599149386954389	-
6872827	A830019P07Rik	0.402	0.599149386954389	-
6877608	Gca	0.402	0.599149386954389	-
6885487	Sec16a	0.402	0.599149386954389	-

6913975	Bspry	0.402	0.599149386954389	-
6942376	Ncf1	0.402	0.599149386954389	-
6953339	Pdia4	0.402	0.599149386954389	-
6981592	Ubxn8	0.402	0.599149386954389	-
6994667	Stt3a	0.402	0.599149386954389	-
6998494	Col6a6	0.402	0.599149386954389	-
7010092	Gpr34	0.402	0.599149386954389	-
7020626	Reps2	0.402	0.599149386954389	-
6747912	Tcfap2d	0.403	0.599149386954389	-
6768150	Ascc1	0.403	0.599149386954389	-
6769156	Elane	0.403	0.599149386954389	-
6775235	Lrrc3	0.403	0.599149386954389	-
6796658	Dlst	0.403	0.599149386954389	-
6799524	Rnf144a	0.403	0.599149386954389	-
6820113	Gfra2	0.403	0.599149386954389	-
6824142	Tspan14	0.403	0.599149386954389	-
6833306	Acvr1b	0.403	0.599149386954389	-
6849312	Noxo1	0.403	0.599149386954389	-
6849532	Zfp523	0.403	0.599149386954389	-
6850688	Dlk2	0.403	0.599149386954389	-
6875792	Pmpca	0.403	0.599149386954389	-
6892903	Matn4	0.403	0.599149386954389	-
6915745	Atg4c	0.403	0.599149386954389	-
6921559	Mrpl50	0.403	0.599149386954389	-
6929622	Kcnk3	0.403	0.599149386954389	-
6959968	1600014C10Rik	0.403	0.599149386954389	-
6966189	Spred3	0.403	0.599149386954389	-
6970635	Rras2	0.403	0.599149386954389	-
6986339	Ccdc7	0.403	0.599149386954389	-
6988773	Zfp259	0.403	0.599149386954389	-
6997764	Zic1	0.403	0.599149386954389	-
6752152	Serpinb7	0.404	0.599149386954389	-
6755966	Rab3gap2	0.404	0.599149386954389	-
6768450	Jmjd1c	0.404	0.599149386954389	+
6781909	Stx8	0.404	0.599149386954389	-
6786516	1110067D22Rik	0.404	0.599149386954389	-
6787285	Wwc1	0.404	0.599149386954389	-
6792416	Nat9	0.404	0.599149386954389	-
6796699	Batf	0.404	0.599149386954389	-
6798801	Slc7a15	0.404	0.599149386954389	-
6807544	Gm5084	0.404	0.599149386954389	-
6809272	Enc1	0.404	0.599149386954389	-
6816060	Gpbp1	0.404	0.599149386954389	-
6817611	Zmiz1	0.404	0.599149386954389	+
6819143	Abhd4	0.404	0.599149386954389	-
6847540	App	0.404	0.599149386954389	-
6847553	Cyyr1	0.404	0.599149386954389	-
6850094	D17H6S56E-3	0.404	0.599149386954389	-
6866305	Spire1	0.404	0.599149386954389	-
6867930	Pla2g16	0.404	0.599149386954389	-

6871097	Banf1	0.404	0.599149386954389	-
6871181	Tm7sf2	0.404	0.599149386954389	-
6888891	Tspan18	0.404	0.599149386954389	-
6905588	Slc33a1	0.404	0.599149386954389	-
6929260	Klhl7	0.404	0.599149386954389	-
6932387	Ereg	0.404	0.599149386954389	-
6940912	Mfsd7a	0.404	0.599149386954389	-
6949007	Lmcd1	0.404	0.599149386954389	-
6963237	Olfr701	0.404	0.599149386954389	-
6986086	2810004N23Rik	0.404	0.599149386954389	-
6987460	9530077C05Rik	0.404	0.599149386954389	-
7010390	Slc6a14	0.404	0.599149386954389	-
7014542	Fam120c	0.404	0.599149386954389	-
7016816	Usp26	0.404	0.599149386954389	-
7017533	Cetn2	0.404	0.599149386954389	-
6764721	Capn2	0.405	0.599149386954389	-
6774371	Col13a1	0.405	0.599149386954389	-
6780868	D930048N14Rik	0.405	0.599149386954389	-
6785133	Kctd2	0.405	0.599149386954389	-
6792760	Nploc4	0.405	0.599149386954389	-
6836829	Dgat1	0.405	0.599149386954389	-
6875197	Arl5b	0.405	0.599149386954389	+
6876212	Eng	0.405	0.599149386954389	-
6876218	Tor2a	0.405	0.599149386954389	-
6886180	Nr5a1	0.405	0.599149386954389	-
6889661	Fibin	0.405	0.599149386954389	-
6911651	2610301B20Rik	0.405	0.599149386954389	-
6916221	Nrd1	0.405	0.599149386954389	-
6922293	Ambp	0.405	0.599149386954389	-
6989983	Csnk1g1	0.405	0.599149386954389	-
7009896	Xk	0.405	0.599149386954389	-
7011928	Zfp275	0.405	0.599149386954389	+
6759648	Pecr	0.406	0.599149386954389	-
6775364	Pcsk4	0.406	0.599149386954389	-
6784259	Aoc2	0.406	0.599149386954389	-
6798281	AW555464	0.406	0.599149386954389	-
6836723	Ly6i	0.406	0.599149386954389	-
6838807	Gpr84	0.406	0.599149386954389	-
6854462	Decr2	0.406	0.599149386954389	+
6865573	Cep120	0.406	0.599149386954389	-
6894286	Zbtb46	0.406	0.599149386954389	-
6916322	Bend5	0.406	0.599149386954389	-
6926490	Dnajc16	0.406	0.599149386954389	-
6928470	4930511M11Rik	0.406	0.599149386954389	-
6934922	Trim50	0.406	0.599149386954389	-
6942617	BC037034	0.406	0.599149386954389	-
6950092	Clec2h	0.406	0.599149386954389	-
6959588	Prodh2	0.406	0.599149386954389	-
6970155	Mrpl17	0.406	0.599149386954389	-
6973689	Stxbp2	0.406	0.599149386954389	-

6974762	Lsm1	0.406	0.599149386954389	-
6990416	Nedd4	0.406	0.599149386954389	-
7013165	Fgf16	0.406	0.599149386954389	-
6778373	Rnf215	0.407	0.599149386954389	-
6782533	Tusc5	0.407	0.599149386954389	-
6819308	Rnf17	0.407	0.599149386954389	-
6843393	Dyrk1a	0.407	0.599149386954389	-
6848199	Mx1	0.407	0.599149386954389	-
6858456	Cul2	0.407	0.599149386954389	-
6874080	Hspa12a	0.407	0.599149386954389	+
6876037	Pkn3	0.407	0.599149386954389	-
6876162	Nup214	0.407	0.599149386954389	-
6878406	Gm14461	0.407	0.599149386954389	-
6916192	Cc2d1b	0.407	0.599149386954389	-
6942562	Irs3	0.407	0.599149386954389	-
6958256	Bhlhe41	0.407	0.599149386954389	+
6968683	Mfge8	0.407	0.599149386954389	-
6982014	Adam34	0.407	0.599149386954389	-
6987924	Arhgap32	0.407	0.599149386954389	-
6993696	Zfp426	0.407	0.599149386954389	-
7002728	Itgb1bp1	0.407	0.599149386954389	-
7012860	Foxo4	0.407	0.599149386954389	-
7017592	Bcap31	0.407	0.599149386954389	-
6775625	Nuak1	0.408	0.599149386954389	-
6776603	Alx1	0.408	0.599149386954389	-
6785508	Slc16a3	0.408	0.599149386954389	-
6815726	Cwc27	0.408	0.599149386954389	-
6819901	Trim35	0.408	0.599149386954389	-
6851177	St6gal2	0.408	0.599149386954389	-
6863048	Map3k8	0.408	0.599149386954389	-
6865967	Hmgxb3	0.408	0.599149386954389	-
6868091	Ms4a4c	0.408	0.599149386954389	-
6869964	Pax2	0.408	0.599149386954389	-
6880497	Rad51	0.408	0.599149386954389	-
6882491	Raly	0.408	0.599149386954389	-
6899650	Tmod4	0.408	0.599149386954389	-
6909300	Egf	0.408	0.599149386954389	-
6916597	Mutyh	0.408	0.599149386954389	-
6929908	Acox3	0.408	0.599149386954389	-
6933613	Tchp	0.408	0.599149386954389	-
6937228	Ctbp1	0.408	0.599149386954389	-
6941661	Rph3a	0.408	0.599149386954389	-
6944019	Glcci1	0.408	0.599149386954389	-
6947470	Zfml	0.408	0.599149386954389	-
6949992	Parp11	0.408	0.599149386954389	-
6956852	Fxyd4	0.408	0.599149386954389	-
6979699	Spire2	0.408	0.599149386954389	-
6979716	Gas8	0.408	0.599149386954389	-
6981757	Dlc1	0.408	0.599149386954389	-
6992350	Dalrd3	0.408	0.599149386954389	-

6995259	Sidt2	0.408	0.599149386954389	-
7011402	Brs3	0.408	0.599149386954389	-
6749081	Tpp2	0.409	0.599149386954389	-
6769597	Igf1	0.409	0.599149386954389	-
6785130	Ict1	0.409	0.599149386954389	-
6785745	Pgam2	0.409	0.599149386954389	-
6792260	BC006965	0.409	0.599149386954389	-
6792427	C630004H02Rik	0.409	0.599149386954389	-
6798213	Tnfaip2	0.409	0.599149386954389	-
6813733	Ctsll3	0.409	0.599149386954389	-
6819287	Khnyn	0.409	0.599149386954389	-
6836716	2010109I03Rik	0.409	0.599149386954389	-
6858895	3110002H16Rik	0.409	0.599149386954389	-
6875856	Dbh	0.409	0.599149386954389	-
6890193	Ccdc32	0.409	0.599149386954389	-
6916530	Lrrc41	0.409	0.599149386954389	-
6917981	Ddost	0.409	0.599149386954389	-
6946750	A930038C07Rik	0.409	0.599149386954389	-
6947558	Alms1	0.409	0.599149386954389	-
6947738	8430410A17Rik	0.409	0.599149386954389	-
6959800	Slc7a10	0.409	0.599149386954389	-
6965847	Zfp109	0.409	0.599149386954389	-
6971341	Sep 01	0.409	0.599149386954389	-
6973247	Nup98	0.409	0.599149386954389	+
6977096	Jak3	0.409	0.599149386954389	-
6989925	Pdcd7	0.409	0.599149386954389	-
6759342	Plekhm3	0.41	0.599149386954389	-
6771535	Cyp27b1	0.41	0.599149386954389	-
6771627	Atp5b	0.41	0.599149386954389	-
6782496	Dbil5	0.41	0.599149386954389	-
6795610	Wdr20b	0.41	0.599149386954389	-
6809447	Mrps27	0.41	0.599149386954389	-
6830370	Slc30a8	0.41	0.599149386954389	-
6845366	Dirc2	0.41	0.599149386954389	-
6851198	Fsd1	0.41	0.599149386954389	-
6867742	Gal3st3	0.41	0.599149386954389	-
6871274	Dnajc4	0.41	0.599149386954389	-
6872029	Aldh1a7	0.41	0.599149386954389	-
6878718	Prg2	0.41	0.599149386954389	-
6882249	Rspo4	0.41	0.599149386954389	-
6884196	2310003C23Rik	0.41	0.599149386954389	-
6886678	Rnd3	0.41	0.599149386954389	+
6886711	Rbm43	0.41	0.599149386954389	-
6908149	Prpf38b	0.41	0.599149386954389	-
6913638	Rad23b	0.41	0.599149386954389	-
6952070	Tspan12	0.41	0.599149386954389	-
6958063	Abcc9	0.41	0.599149386954389	-
6963137	Trim34	0.41	0.599149386954389	-
6770160	Dusp6	0.411	0.599149386954389	+
6784263	G6pc	0.411	0.599149386954389	-

6792733	Aatk	0.411	0.599149386954389	-
6807000	Nxnl2	0.411	0.599149386954389	-
6811901	Prl8a6	0.411	0.599149386954389	-
6813768	Cts7	0.411	0.599149386954389	-
6823429	D14Ertd449e	0.411	0.599149386954389	-
6824915	Zmym5	0.411	0.599149386954389	-
6826018	Slc25a30	0.411	0.599149386954389	-
6831821	Cyth4	0.411	0.599149386954389	-
6837322	Polr3h	0.411	0.599149386954389	-
6843194	Son	0.411	0.599149386954389	-
6849571	Mapk13	0.411	0.599149386954389	-
6875664	Wdr85	0.411	0.599149386954389	-
6883127	Slc12a5	0.411	0.599149386954389	-
6900970	F3	0.411	0.599149386954389	-
6908219	Ntng1	0.411	0.599149386954389	+
6917791	Hmgcl	0.411	0.599149386954389	-
6925559	Zbtb8a	0.411	0.599149386954389	-
6936855	Paxip1	0.411	0.599149386954389	-
6940301	A430057O09	0.411	0.599149386954389	-
6946026	Gimap9	0.411	0.599149386954389	-
6946894	Eif2ak3	0.411	0.599149386954389	-
6966302	Cox6b1	0.411	0.599149386954389	-
6969693	Dgat2	0.411	0.599149386954389	-
6996213	Coro2b	0.411	0.599149386954389	-
7009796	Slc35a2	0.411	0.599149386954389	-
6760314	A630001G21Rik	0.412	0.599149386954389	-
6765971	Shprh	0.412	0.599149386954389	-
6775518	Glt8d2	0.412	0.599149386954389	-
6782085	Ybx2	0.412	0.599149386954389	-
6784253	Vps25	0.412	0.599149386954389	-
6788083	BC049762	0.412	0.599149386954389	-
6792685	Cbx4	0.412	0.599149386954389	-
6797230	Spata7	0.412	0.599149386954389	-
6814557	Nr2f1	0.412	0.599149386954389	-
6817143	Top2b	0.412	0.599149386954389	-
6822127	Dnajc3	0.412	0.599149386954389	-
6828326	Sepp1	0.412	0.599149386954389	-
6831606	Gsdmd	0.412	0.599149386954389	-
6839943	Vwa5b2	0.412	0.599149386954389	-
6840579	Tfrc	0.412	0.599149386954389	-
6854562	Taf11	0.412	0.599149386954389	-
6861354	Pdgfrb	0.412	0.599149386954389	-
6867638	Pitpnm1	0.412	0.599149386954389	-
6868128	Mrpl16	0.412	0.599149386954389	-
6880469	Ivd	0.412	0.599149386954389	+
6907239	Bola1	0.412	0.599149386954389	-
6919050	A430005L14Rik	0.412	0.599149386954389	-
6932374	Cxcl1	0.412	0.599149386954389	-
6955265	Gkn1	0.412	0.599149386954389	-
6977139	Tpm4	0.412	0.599149386954389	+

6988643	Bcl9l	0.412	0.599149386954389	-
6995420	Htr3b	0.412	0.599149386954389	-
6759396	Idh1	0.413	0.599149386954389	+
6763991	Rgs4	0.413	0.599149386954389	+
6771587	Rdh9	0.413	0.599149386954389	-
6776577	Nts	0.413	0.599149386954389	-
6790075	Utp6	0.413	0.599149386954389	-
6791000	Sgca	0.413	0.599149386954389	-
6791565	Slc25a39	0.413	0.599149386954389	-
6795025	Scfd1	0.413	0.599149386954389	-
6800020	Hdac9	0.413	0.599149386954389	-
6817368	2310021P13Rik	0.413	0.599149386954389	-
6837445	Pacsin2	0.413	0.599149386954389	-
6853766	Slc22a3	0.413	0.599149386954389	-
6859707	Pik3c3	0.413	0.599149386954389	-
6860183	Pcdhb11	0.413	0.599149386954389	-
6862586	Mbp	0.413	0.599149386954389	-
6885498	Abo	0.413	0.599149386954389	-
6887903	Evx2	0.413	0.599149386954389	-
6925839	Stx12	0.413	0.599149386954389	-
6934061	Tmem116	0.413	0.599149386954389	-
6937481	Tbc1d14	0.413	0.599149386954389	-
6963911	Anks4b	0.413	0.599149386954389	-
6972205	Lrdd	0.413	0.599149386954389	-
6980568	Gas6	0.413	0.599149386954389	-
6990842	Mei4	0.413	0.599149386954389	-
6995450	Ankk1	0.413	0.599149386954389	-
7010327	Phf16	0.413	0.599149386954389	-
6751337	Efhd1	0.414	0.599149386954389	-
6767613	Popdc3	0.414	0.599149386954389	-
6778352	Pes1	0.414	0.599149386954389	-
6786249	Plek	0.414	0.599149386954389	-
6788676	Zkscan17	0.414	0.599149386954389	-
6792742	Slc38a10	0.414	0.599149386954389	-
6822772	Psmd6	0.414	0.599149386954389	-
6826007	Spert	0.414	0.599149386954389	-
6844080	Pla2g10	0.414	0.599149386954389	-
6867853	Map4k2	0.414	0.599149386954389	-
6869013	Slc1a1	0.414	0.599149386954389	-
6869216	Papss2	0.414	0.599149386954389	-
6869300	Lipm	0.414	0.599149386954389	-
6870614	Casp7	0.414	0.599149386954389	-
6891903	9230104L09Rik	0.414	0.599149386954389	-
6907715	Nr1h5	0.414	0.599149386954389	-
6924793	Tmem69	0.414	0.599149386954389	-
6935486	Arpc1a	0.414	0.599149386954389	-
6939196	Chic2	0.414	0.599149386954389	-
6945308	Akr1b8	0.414	0.599149386954389	-
6959167	Arhgef1	0.414	0.599149386954389	-
6963202	Smpd1	0.414	0.599149386954389	-

6964635	Lhpp	0.414	0.599149386954389	+
6966187	Rasgrp4	0.414	0.599149386954389	-
6971656	Chst15	0.414	0.599149386954389	-
6977085	Slc27a1	0.414	0.599149386954389	-
6980061	Pex11c	0.414	0.599149386954389	-
6992430	2610002I17Rik	0.414	0.599149386954389	-
6992472	Prss46	0.414	0.599149386954389	-
6762452	Igfn1	0.415	0.599149386954389	-
6784368	Higd1b	0.415	0.599149386954389	-
6796645	Ylpm1	0.415	0.599149386954389	-
6818213	Mmrn2	0.415	0.599149386954389	-
6823768	Dph3	0.415	0.599149386954389	-
6840923	Lsamp	0.415	0.599149386954389	-
6845185	Osta	0.415	0.599149386954389	-
6852820	Prkce	0.415	0.599149386954389	-
6875960	Gm347	0.415	0.599149386954389	-
6881556	Btbd3	0.415	0.599149386954389	-
6893263	Dpm1	0.415	0.599149386954389	+
6897008	3110057O12Rik	0.415	0.599149386954389	-
6916535	Pomgnt1	0.415	0.599149386954389	-
6966588	C80913	0.415	0.599149386954389	-
6968836	Fsd2	0.415	0.599149386954389	-
7009793	Otud5	0.415	0.599149386954389	-
7014551	Huwe1	0.415	0.599149386954389	-
7017277	Slitrk4	0.415	0.599149386954389	-
6748545	Cfc1	0.416	0.599149386954389	-
6750555	Plcd4	0.416	0.599149386954389	-
6758325	Uxs1	0.416	0.599149386954389	-
6762016	Lct	0.416	0.599149386954389	+
6762411	Elf3	0.416	0.599149386954389	-
6775750	Syn3	0.416	0.599149386954389	-
6790684	Pctp	0.416	0.599149386954389	-
6825653	Chmp7	0.416	0.599149386954389	-
6873363	Fgf8	0.416	0.599149386954389	-
6887327	Scn1a	0.416	0.599149386954389	-
6892990	Slc35c2	0.416	0.599149386954389	-
6901953	Gbp3	0.416	0.599149386954389	-
6908075	Gstm6	0.416	0.599149386954389	-
6915292	Zfp352	0.416	0.599149386954389	-
6922883	Zdhhc21	0.416	0.599149386954389	-
6953624	Hibadh	0.416	0.599149386954389	-
6959198	Cic	0.416	0.599149386954389	-
6970035	E030002O03Rik	0.416	0.599149386954389	-
6974113	Atp11a	0.416	0.599149386954389	-
6977145	Ap1m1	0.416	0.599149386954389	-
6979424	Atmin	0.416	0.599149386954389	-
6980124	Timm44	0.416	0.599149386954389	-
6983869	Nacc1	0.416	0.599149386954389	-
6985942	Aprt	0.416	0.599149386954389	-
7011014	Ocrl	0.416	0.599149386954389	-

6749009	Mrps9	0.417	0.599149386954389	-
6758625	Nab1	0.417	0.599149386954389	-
6762104	C4bp	0.417	0.599149386954389	-
6769105	Slc1a6	0.417	0.599149386954389	-
6769267	Eef2	0.417	0.599149386954389	-
6784048	Msl1	0.417	0.599149386954389	-
6784313	Nags	0.417	0.599149386954389	-
6787610	Ttc1	0.417	0.599149386954389	-
6788657	Guk1	0.417	0.599149386954389	-
6804901	B3galnt2	0.417	0.599149386954389	-
6806164	4933417A18Rik	0.417	0.599149386954389	-
6813671	4932411G14Rik	0.417	0.599149386954389	-
6814028	Zfp595	0.417	0.599149386954389	-
6815702	2410002O22Rik	0.417	0.599149386954389	-
6819662	Rnaseh2b	0.417	0.599149386954389	-
6827787	Dct	0.417	0.599149386954389	-
6850695	Zfp318	0.417	0.599149386954389	-
6858773	Colec12	0.417	0.599149386954389	-
6906261	Gria2	0.417	0.599149386954389	-
6910088	Hs2st1	0.417	0.599149386954389	-
6913149	Dcaf10	0.417	0.599149386954389	-
6924968	Rimkla	0.417	0.599149386954389	-
6938797	Apbb2	0.417	0.599149386954389	-
6939771	Grsf1	0.417	0.599149386954389	-
6949160	Atg7	0.417	0.599149386954389	-
6960163	Zfp819	0.417	0.599149386954389	-
6966424	Pdcd2l	0.417	0.599149386954389	-
6971437	Rgs10	0.417	0.599149386954389	-
6992309	Mst1	0.417	0.599149386954389	-
7011263	Phf6	0.417	0.599149386954389	-
6769138	Bsg	0.418	0.599149386954389	-
6788290	Il4	0.418	0.599149386954389	-
6790655	Scpep1	0.418	0.599149386954389	-
6791326	Krt25	0.418	0.599149386954389	-
6791443	Ghdc	0.418	0.599149386954389	-
6819860	Fbxo16	0.418	0.599149386954389	-
6834504	Fbxl7	0.418	0.599149386954389	-
6835759	Enpp2	0.418	0.599149386954389	-
6838036	Zcrb1	0.418	0.599149386954389	-
6854287	Prss41	0.418	0.599149386954389	-
6867643	Gpr152	0.418	0.599149386954389	-
6873252	Cwf19l1	0.418	0.599149386954389	-
6882101	Cst10	0.418	0.599149386954389	-
6882416	Bpil1	0.418	0.599149386954389	-
6882444	BC018465	0.418	0.599149386954389	-
6883084	Svs6	0.418	0.599149386954389	-
6883125	Mmp9	0.418	0.599149386954389	-
6890698	Fahd2a	0.418	0.599149386954389	-
6909311	Sec24b	0.418	0.599149386954389	-
6935463	Trrap	0.418	0.599149386954389	-

6942193	Asl	0.418	0.599149386954389	-
6959300	Blvrb	0.418	0.599149386954389	+
6959417	Paf1	0.418	0.599149386954389	-
6960692	Slc17a6	0.418	0.599149386954389	-
6965178	Rassf7	0.418	0.599149386954389	-
6968708	Polg	0.418	0.599149386954389	-
6969196	Tmem135	0.418	0.599149386954389	-
6971432	BC017158	0.418	0.599149386954389	-
6978354	Gpr56	0.418	0.599149386954389	-
6982155	Snx25	0.418	0.599149386954389	-
6983339	9130011J15Rik	0.418	0.599149386954389	-
6992338	1700102P08Rik	0.418	0.599149386954389	-
6993204	Gria4	0.418	0.599149386954389	-
7010696	Nkap	0.418	0.599149386954389	-
6748688	Vwa3b	0.419	0.599149386954389	-
6748886	Il1rl2	0.419	0.599149386954389	-
6759333	Fzd5	0.419	0.599149386954389	-
6775457	Hmg20b	0.419	0.599149386954389	-
6790374	Appbp2	0.419	0.599149386954389	-
6810874	Wdr37	0.419	0.599149386954389	-
6825216	Kpna3	0.419	0.599149386954389	-
6837409	Cyp2d40	0.419	0.599149386954389	-
6848513	Tulp4	0.419	0.599149386954389	-
6849765	Umodl1	0.419	0.599149386954389	-
6871570	Ms4a3	0.419	0.599149386954389	-
6872203	Smc5	0.419	0.599149386954389	-
6875325	Mllt10	0.419	0.599149386954389	-
6875637	Il1f9	0.419	0.599149386954389	-
6881139	Sirpa	0.419	0.599149386954389	-
6881488	Snap25	0.419	0.599149386954389	-
6893173	Spata2	0.419	0.599149386954389	-
6897363	Rab33b	0.419	0.599149386954389	-
6907124	Zfp687	0.419	0.599149386954389	-
6908092	Ampd2	0.419	0.599149386954389	-
6923676	Kank4	0.419	0.599149386954389	-
6957346	Fkbp4	0.419	0.599149386954389	-
6964368	Zfp646	0.419	0.599149386954389	-
6965319	Kcnq1	0.419	0.599149386954389	-
6977589	Inpp4b	0.419	0.599149386954389	-
6977656	Ucp1	0.419	0.599149386954389	-
6985919	Trhr2	0.419	0.599149386954389	-
6986648	Casp1	0.419	0.599149386954389	-
6989360	Ptpn9	0.419	0.599149386954389	-
6990530	Myo5c	0.419	0.599149386954389	-
6994371	Tmem45b	0.419	0.599149386954389	-
7013867	Drp2	0.419	0.599149386954389	-
7016887	Fam122b	0.419	0.599149386954389	-
6747472	Adhfe1	0.42	0.599149386954389	-
6768270	Tacr2	0.42	0.599149386954389	-
6775891	Actr6	0.42	0.599149386954389	-

6777937	Inhbc	0.42	0.599149386954389	-
6785306	Afmid	0.42	0.599149386954389	-
6792553	Jmjd6	0.42	0.599149386954389	-
6818939	Parp2	0.42	0.599149386954389	-
6833420	Pcbp2	0.42	0.599149386954389	-
6836854	Arhgap39	0.42	0.599149386954389	-
6839957	Fam131a	0.42	0.599149386954389	-
6840631	Heg1	0.42	0.599149386954389	-
6882515	Dynlrb1	0.42	0.599149386954389	-
6912491	Casp8ap2	0.42	0.599149386954389	-
6912565	Cnr1	0.42	0.599149386954389	-
6947927	Uroc1	0.42	0.599149386954389	-
6950558	Dera	0.42	0.599149386954389	-
6955047	Cd207	0.42	0.599149386954389	-
6956238	Il5ra	0.42	0.599149386954389	-
6958404	Ergic2	0.42	0.599149386954389	-
6967269	Dbx1	0.42	0.599149386954389	-
6970839	Coq7	0.42	0.599149386954389	-
6972471	Fadd	0.42	0.599149386954389	-
6973685	Mcoln1	0.42	0.599149386954389	-
6974682	Zmat4	0.42	0.599149386954389	-
6984485	Dok4	0.42	0.599149386954389	-
6988701	Cd3d	0.42	0.599149386954389	-
7020644	S100g	0.42	0.599149386954389	-
6749923	Eef1b2	0.421	0.599149386954389	-
6757423	Smap1	0.421	0.599149386954389	-
6789066	Map2k4	0.421	0.599149386954389	-
6792125	Abca6	0.421	0.599149386954389	-
6792595	Tmc6	0.421	0.599149386954389	-
6796544	Papln	0.421	0.599149386954389	-
6801324	Klhl28	0.421	0.599149386954389	-
6803178	Btbd7	0.421	0.599149386954389	-
6810333	Esm1	0.421	0.599149386954389	-
6812642	Elovl2	0.421	0.599149386954389	-
6823892	Opn4	0.421	0.599149386954389	-
6835454	Kcnv1	0.421	0.599149386954389	-
6836700	Ly6d	0.421	0.599149386954389	-
6838657	Krt6a	0.421	0.599149386954389	-
6875994	Setx	0.421	0.599149386954389	-
6884466	Dhtkd1	0.421	0.599149386954389	-
6885855	Fam78a	0.421	0.599149386954389	-
6914029	6330416G13Rik	0.421	0.599149386954389	-
6916101	Cdcp2	0.421	0.599149386954389	-
6917277	Fndc5	0.421	0.599149386954389	+
6923928	C130073F10Rik	0.421	0.599149386954389	-
6937072	Zfp513	0.421	0.599149386954389	-
6948797	Cntn4	0.421	0.599149386954389	-
6953613	Hoxa9	0.421	0.599149386954389	-
6954585	Ptcd3	0.421	0.599149386954389	-
6963907	Tmem159	0.421	0.599149386954389	-

6965202	Pnpla2	0.421	0.599149386954389	-
6972292	6330512M04Rik	0.421	0.599149386954389	-
6990509	Onecut1	0.421	0.599149386954389	-
6754138	Rgs16	0.422	0.599149386954389	-
6754263	Tor1aip2	0.422	0.599149386954389	-
6755240	Slamf9	0.422	0.599149386954389	-
6757646	Lgsn	0.422	0.599149386954389	-
6758223	2610017I09Rik	0.422	0.599149386954389	-
6769179	Cnn2	0.422	0.599149386954389	-
6774295	Chst3	0.422	0.599149386954389	-
6781441	Drg2	0.422	0.599149386954389	-
6789538	Tekt1	0.422	0.599149386954389	-
6790124	Myo1d	0.422	0.599149386954389	-
6791533	Ppy	0.422	0.599149386954389	-
6792030	Cacng5	0.422	0.599149386954389	-
6806219	Fars2	0.422	0.599149386954389	-
6814382	Exoc3	0.422	0.599149386954389	-
6832256	Bik	0.422	0.599149386954389	-
6844575	Tbccd1	0.422	0.599149386954389	-
6872834	Ankrd1	0.422	0.599149386954389	-
6883487	Tshz2	0.422	0.599149386954389	-
6892504	Samhd1	0.422	0.599149386954389	-
6892811	0610008F07Rik	0.422	0.599149386954389	-
6945526	Trim24	0.422	0.599149386954389	-
6969765	Neu3	0.422	0.599149386954389	-
6977025	Lsm4	0.422	0.599149386954389	-
6979483	Cdh13	0.422	0.599149386954389	-
6983780	Elmod2	0.422	0.599149386954389	-
6984927	Rrad	0.422	0.599149386954389	-
6985413	Tmem170	0.422	0.599149386954389	-
6995717	Acat1	0.422	0.599149386954389	-
7011952	Srpk3	0.422	0.599149386954389	-
6764036	Fcrla	0.423	0.599149386954389	-
6783701	Dlx3	0.423	0.599149386954389	-
6784237	Naglu	0.423	0.599149386954389	-
6792406	Cd300lf	0.423	0.599149386954389	-
6792756	2310003H01Rik	0.423	0.599149386954389	-
6809266	Gfm2	0.423	0.599149386954389	-
6820033	Entpd4	0.423	0.599149386954389	-
6849073	Zfp53	0.423	0.599149386954389	-
6850683	Xpo5	0.423	0.599149386954389	-
6892032	Acss1	0.423	0.599149386954389	+
6924515	Elavl4	0.423	0.599149386954389	-
6926934	Apitd1	0.423	0.599149386954389	-
6929663	Tcf23	0.423	0.599149386954389	-
6932209	Prol1	0.423	0.599149386954389	-
6940412	Nkx6-1	0.423	0.599149386954389	-
6943036	Cyp3a41b	0.423	0.599149386954389	-
6974714	Tm2d2	0.423	0.599149386954389	-
6977251	Hmgxb4	0.423	0.599149386954389	-

6977696	Asf1b	0.423	0.599149386954389	-
6977814	Gpt2	0.423	0.599149386954389	-
6979040	Pmfbp1	0.423	0.599149386954389	-
6989238	Chrna5	0.423	0.599149386954389	-
6992974	Trak1	0.423	0.599149386954389	-
6998397	Trf	0.423	0.599149386954389	-
7010764	Mcts1	0.423	0.599149386954389	-
7015461	Ebp	0.423	0.599149386954389	-
7016823	Gpc4	0.423	0.599149386954389	-
6753191	Ren1	0.424	0.599149386954389	-
6759949	Farsb	0.424	0.599149386954389	-
6760643	Rab17	0.424	0.599149386954389	-
6781383	Nt5m	0.424	0.599149386954389	-
6783367	Mrps23	0.424	0.599149386954389	-
6785367	Gaa	0.424	0.599149386954389	-
6789365	Eif5a	0.424	0.599149386954389	-
6791442	Hcrt	0.424	0.599149386954389	-
6801493	Map4k5	0.424	0.599149386954389	-
6802185	Adam21	0.424	0.599149386954389	-
6810063	Elovl7	0.424	0.599149386954389	-
6810421	Mocs2	0.424	0.599149386954389	-
6813874	Hsd17b3	0.424	0.599149386954389	-
6822073	Gpr180	0.424	0.599149386954389	-
6845446	Eaf2	0.424	0.599149386954389	-
6848215	Zfp295	0.424	0.599149386954389	-
6849110	3110052M02Rik	0.424	0.599149386954389	-
6881237	Hspa12b	0.424	0.599149386954389	-
6885502	Med22	0.424	0.599149386954389	-
6890253	Ndufaf1	0.424	0.599149386954389	-
6908073	Gstm7	0.424	0.599149386954389	+
6908474	Slc30a7	0.424	0.599149386954389	-
6955111	Cyp26b1	0.424	0.599149386954389	-
6962751	Alg8	0.424	0.599149386954389	-
6972156	Sirt3	0.424	0.599149386954389	-
6992346	Usp19	0.424	0.599149386954389	-
7013345	2010106E10Rik	0.424	0.599149386954389	-
6755176	Arhgap30	0.425	0.599149386954389	-
6761268	Kdsr	0.425	0.599149386954389	-
6765716	Lrp11	0.425	0.599149386954389	-
6768143	Cbara1	0.425	0.599149386954389	-
6768232	Ppa1	0.425	0.599149386954389	-
6781605	Trim16	0.425	0.599149386954389	-
6784260	Aoc3	0.425	0.599149386954389	-
6787830	Sgcd	0.425	0.599149386954389	-
6790239	Slfn9	0.425	0.599149386954389	-
6837189	Rps19bp1	0.425	0.599149386954389	-
6839504	Mkl2	0.425	0.599149386954389	-
6855246	H2-M2	0.425	0.599149386954389	-
6865231	Fem1c	0.425	0.599149386954389	-
6869885	Entpd7	0.425	0.599149386954389	+

6885924	Garnl3	0.425	0.599149386954389	-
6892374	Ggt7	0.425	0.599149386954389	-
6893393	Cyp24a1	0.425	0.599149386954389	-
6896518	Prkci	0.425	0.599149386954389	-
6898775	Plrg1	0.425	0.599149386954389	-
6907971	Cd53	0.425	0.599149386954389	-
6916239	Ttc39a	0.425	0.599149386954389	-
6916767	Zmynd12	0.425	0.599149386954389	-
6930484	Lap3	0.425	0.599149386954389	-
6937190	Pisd	0.425	0.599149386954389	-
6961895	Aen	0.425	0.599149386954389	-
6970287	Cyb5r2	0.425	0.599149386954389	-
6978323	Rspry1	0.425	0.599149386954389	-
6981664	Tnks	0.425	0.599149386954389	-
6987392	1810026J23Rik	0.425	0.599149386954389	-
6989045	Ppp2r1b	0.425	0.599149386954389	-
7009939	Otc	0.425	0.599149386954389	-
7020084	Lrch2	0.425	0.599149386954389	-
6761286	Serpinb3c	0.426	0.599149386954389	-
6783029	Taf15	0.426	0.599149386954389	-
6788739	Srebf1	0.426	0.599149386954389	-
6798513	Rab10	0.426	0.599149386954389	-
6802525	Vipar	0.426	0.599149386954389	-
6819883	Pbk	0.426	0.599149386954389	-
6829283	Zfp622	0.426	0.599149386954389	-
6836006	E430025E21Rik	0.426	0.599149386954389	-
6850789	Med20	0.426	0.599149386954389	-
6859935	Apc	0.426	0.599149386954389	-
6866307	Ptpn2	0.426	0.599149386954389	-
6878469	Ssfa2	0.426	0.599149386954389	-
6879938	Ccdc34	0.426	0.599149386954389	-
6882700	Ctnnbl1	0.426	0.599149386954389	-
6884267	Lime1	0.426	0.599149386954389	-
6899097	Mef2d	0.426	0.599149386954389	-
6903946	Phc3	0.426	0.599149386954389	-
6912594	Rars2	0.426	0.599149386954389	-
6946927	Rnf103	0.426	0.599149386954389	-
6957126	Lrrc23	0.426	0.599149386954389	-
6960421	Kdelr1	0.426	0.599149386954389	-
6963578	Far1	0.426	0.599149386954389	-
6975224	Tex15	0.426	0.599149386954389	-
6980271	Arglu1	0.426	0.599149386954389	-
6755282	Aim2	0.427	0.599149386954389	-
6764048	Tomm40l	0.427	0.599149386954389	-
6786991	Acyp2	0.427	0.599149386954389	+
6825688	Slc39a14	0.427	0.599149386954389	-
6835918	Derl1	0.427	0.599149386954389	-
6838337	Hdac7	0.427	0.599149386954389	-
6839826	Trmt2a	0.427	0.599149386954389	-
6845209	Iqcg	0.427	0.599149386954389	-

6873960	Dclre1a	0.427	0.599149386954389	-
6876219	Ptrh1	0.427	0.599149386954389	-
6898210	Il12a	0.427	0.599149386954389	-
6899052	Gpatch4	0.427	0.599149386954389	-
6899053	Iqgap3	0.427	0.599149386954389	-
6916171	2010305A19Rik	0.427	0.599149386954389	-
6923520	Cyp2j6	0.427	0.599149386954389	-
6924466	Cdkn2c	0.427	0.599149386954389	-
6929861	Rgs12	0.427	0.599149386954389	-
6934662	0610007L01Rik	0.427	0.599149386954389	-
6941635	Plbd2	0.427	0.599149386954389	-
6946196	Nfe2l3	0.427	0.599149386954389	-
6947932	Klf15	0.427	0.599149386954389	-
6959615	Atp4a	0.427	0.599149386954389	-
6976971	Sugp1	0.427	0.599149386954389	-
6758663	Asnsd1	0.428	0.599149386954389	-
6775888	Scyl2	0.428	0.599149386954389	-
6792779	P4hb	0.428	0.599149386954389	-
6796331	Arg2	0.428	0.599149386954389	-
6825311	Blk	0.428	0.599149386954389	-
6833171	Aqp2	0.428	0.599149386954389	-
6853121	Adcyap1	0.428	0.599149386954389	-
6879105	Phf21a	0.428	0.599149386954389	-
6900708	Lrrc39	0.428	0.599149386954389	-
6911884	Tmem64	0.428	0.599149386954389	-
6917273	Tmem54	0.428	0.599149386954389	-
6919185	Ccnl2	0.428	0.599149386954389	-
6924801	Nasp	0.428	0.599149386954389	-
6935295	AA881470	0.428	0.599149386954389	-
6956912	Adipor2	0.428	0.599149386954389	-
6959557	Zfp260	0.428	0.599149386954389	-
6978336	Ccl17	0.428	0.599149386954389	-
6983190	Klhl26	0.428	0.599149386954389	-
6993099	Tmem42	0.428	0.599149386954389	-
6996263	Smad6	0.428	0.599149386954389	-
6998614	Hemk1	0.428	0.599149386954389	-
7015462	Porcn	0.428	0.599149386954389	-
6771954	Pcmt1	0.429	0.599149386954389	-
6777518	Hmga2	0.429	0.599149386954389	-
6810717	Akr1e1	0.429	0.599149386954389	-
6817365	Sec24c	0.429	0.599149386954389	-
6818153	Gdf10	0.429	0.599149386954389	-
6856282	Cd70	0.429	0.599149386954389	-
6859471	Elp2	0.429	0.599149386954389	-
6862751	Cyb5	0.429	0.599149386954389	-
6885930	Ralgps1	0.429	0.599149386954389	-
6961182	Tarsl2	0.429	0.599149386954389	-
6962133	A530021J07Rik	0.429	0.599149386954389	-
6965629	Npas1	0.429	0.599149386954389	-
6966297	Wbp7	0.429	0.599149386954389	-

6977016	Isyna1	0.429	0.599149386954389	-
6978923	Cdh1	0.429	0.599149386954389	-
6994711	Panx3	0.429	0.599149386954389	-
7012082	Brcc3	0.429	0.599149386954389	-
7020636	Txlng	0.429	0.599149386954389	-
6751634	Gpc1	0.43	0.599149386954389	-
6755385	Wdr64	0.43	0.599149386954389	-
6764054	Usp21	0.43	0.599149386954389	-
6766470	Raet1e	0.43	0.599149386954389	-
6808363	2210408I21Rik	0.43	0.599149386954389	-
6815490	Mtap1b	0.43	0.599149386954389	-
6829967	Rims2	0.43	0.599149386954389	-
6833005	Fam113b	0.43	0.599149386954389	-
6845375	Parp14	0.43	0.599149386954389	-
6849567	Mapk14	0.43	0.599149386954389	-
6866486	Rab27b	0.43	0.599149386954389	-
6867702	Zdhhc24	0.43	0.599149386954389	-
6871051	Rbm14	0.43	0.599149386954389	-
6884644	Taf3	0.43	0.599149386954389	-
6885395	Ssna1	0.43	0.599149386954389	-
6917489	Taf12	0.43	0.599149386954389	-
6935427	Pms2	0.43	0.599149386954389	-
6936745	Asb10	0.43	0.599149386954389	-
6983115	Zfp868	0.43	0.599149386954389	-
6751231	Itm2c	0.431	0.599149386954389	-
6760794	Thap4	0.431	0.599149386954389	-
6763196	Cacna1e	0.431	0.599149386954389	-
6769878	Hal	0.431	0.599149386954389	-
6774379	2010107G23Rik	0.431	0.599149386954389	-
6784329	BC030867	0.431	0.599149386954389	-
6789969	Sarm1	0.431	0.599149386954389	-
6792792	Pycr1	0.431	0.599149386954389	-
6797707	Glrx5	0.431	0.599149386954389	-
6842112	Pou1f1	0.431	0.599149386954389	-
6846105	Trat1	0.431	0.599149386954389	-
6849991	Rgl2	0.431	0.599149386954389	-
6854403	Cramp1l	0.431	0.599149386954389	-
6867872	Trmt112	0.431	0.599149386954389	-
6867957	Nxf1	0.431	0.599149386954389	-
6873249	Chuk	0.431	0.599149386954389	-
6885475	4932418E24Rik	0.431	0.599149386954389	-
6886078	Rbm18	0.431	0.599149386954389	-
6906838	Flad1	0.431	0.599149386954389	-
6920256	Fhl5	0.431	0.599149386954389	-
6962376	Ctsc	0.431	0.599149386954389	-
6978390	Ndrg4	0.431	0.599149386954389	-
6992347	Qars	0.431	0.599149386954389	-
7017663	Gab3	0.431	0.599149386954389	-
6769249	3110056O03Rik	0.432	0.599149386954389	-
6778065	Mmp19	0.432	0.599149386954389	-

6786945	Ccdc104	0.432	0.599149386954389	-
6786978	Spnb2	0.432	0.599149386954389	-
6797579	Serpina3n	0.432	0.599149386954389	+
6814150	Papd7	0.432	0.599149386954389	-
6822879	Oxsm	0.432	0.599149386954389	-
6855183	H2-M10.1	0.432	0.599149386954389	-
6881146	Stk35	0.432	0.599149386954389	-
6882302	Rem1	0.432	0.599149386954389	-
6898276	Ppm1l	0.432	0.599149386954389	-
6904283	Qrfpr	0.432	0.599149386954389	-
6925350	Mtap7d1	0.432	0.599149386954389	-
6938171	Lgi2	0.432	0.599149386954389	-
6942440	Pom121	0.432	0.599149386954389	-
6942532	Trim56	0.432	0.599149386954389	-
6943168	Slc46a3	0.432	0.599149386954389	-
6947332	Fam176a	0.432	0.599149386954389	-
6952426	Tmem209	0.432	0.599149386954389	-
6956727	Raf1	0.432	0.599149386954389	-
6957423	Klrc1	0.432	0.599149386954389	-
6960273	Aspdh	0.432	0.599149386954389	-
6975443	6430573F11Rik	0.432	0.599149386954389	-
6978823	Elmo3	0.432	0.599149386954389	-
7013998	Ngfrap1	0.432	0.599149386954389	-
6755621	Cnst	0.433	0.599149386954389	-
6775470	Ncln	0.433	0.599149386954389	-
6775559	Slc41a2	0.433	0.599149386954389	+
6776688	BC067068	0.433	0.599149386954389	-
6783321	Sep4	0.433	0.599149386954389	-
6785698	Emid1	0.433	0.599149386954389	-
6788264	Hspa4	0.433	0.599149386954389	-
6792371	Cd300ld	0.433	0.599149386954389	-
6807229	Grk6	0.433	0.599149386954389	-
6813965	Mterfd1	0.433	0.599149386954389	-
6836405	Zfat	0.433	0.599149386954389	-
6839231	Abat	0.433	0.599149386954389	-
6850836	Treml1	0.433	0.599149386954389	-
6860001	Ctnna1	0.433	0.599149386954389	-
6867823	Syvn1	0.433	0.599149386954389	-
6869070	Uhrf2	0.433	0.599149386954389	-
6894245	Nkain4	0.433	0.599149386954389	+
6896770	Acad9	0.433	0.599149386954389	-
6899742	Ankrd34a	0.433	0.599149386954389	-
6904333	--	0.433	0.599149386954389	-
6905006	Maml3	0.433	0.599149386954389	-
6926855	Plod1	0.433	0.599149386954389	-
6926941	Ube4b	0.433	0.599149386954389	-
6933226	Cdc7	0.433	0.599149386954389	-
6948038	Nr2c2	0.433	0.599149386954389	-
6963608	Pde3b	0.433	0.599149386954389	-
6977997	Cyld	0.433	0.599149386954389	-

6986032	Nup133	0.433	0.599149386954389	-
6999114	Crtap	0.433	0.599149386954389	-
6756473	Irf6	0.434	0.599149386954389	-
6767094	Frk	0.434	0.599149386954389	-
6767235	Tube1	0.434	0.599149386954389	-
6771050	Mdm1	0.434	0.599149386954389	-
6796053	Syt16	0.434	0.599149386954389	-
6807007	S1pr3	0.434	0.599149386954389	-
6818499	Sftpa1	0.434	0.599149386954389	-
6824829	Ipo4	0.434	0.599149386954389	-
6834877	Nipal2	0.434	0.599149386954389	-
6844310	Slc25a1	0.434	0.599149386954389	-
6872616	Prkg1	0.434	0.599149386954389	-
6877215	Galnt13	0.434	0.599149386954389	-
6880034	Nop10	0.434	0.599149386954389	-
6884199	Slc17a9	0.434	0.599149386954389	-
6885376	Cobra1	0.434	0.599149386954389	-
6888016	Zfp385b	0.434	0.599149386954389	-
6892893	Wfdc15a	0.434	0.599149386954389	-
6894925	Arrdc3	0.434	0.599149386954389	-
6904309	Il2	0.434	0.599149386954389	-
6960378	Tulp2	0.434	0.599149386954389	-
6975420	D8Ertd82e	0.434	0.599149386954389	-
6976472	Mfap3l	0.434	0.599149386954389	-
6986211	Rbm34	0.434	0.599149386954389	-
6995074	Hmbs	0.434	0.599149386954389	-
7013502	Pabpc5	0.434	0.599149386954389	-
6748736	Eif5b	0.435	0.599149386954389	-
6763777	Mpzl1	0.435	0.599149386954389	-
6766839	Themis	0.435	0.599149386954389	-
6773069	Ncoa7	0.435	0.599149386954389	-
6774343	X99384	0.435	0.599149386954389	-
6783838	Hoxb9	0.435	0.599149386954389	-
6804379	Klf11	0.435	0.599149386954389	-
6827977	Gm5089	0.435	0.599149386954389	-
6836983	Eif3d	0.435	0.599149386954389	-
6840086	Dnajb11	0.435	0.599149386954389	-
6849048	Zfp160	0.435	0.599149386954389	-
6855000	H2-Ke6	0.435	0.599149386954389	-
6872646	Asah2	0.435	0.599149386954389	-
6872791	Slc16a12	0.435	0.599149386954389	-
6879569	Slc1a2	0.435	0.599149386954389	-
6879610	Elf5	0.435	0.599149386954389	-
6881087	Mertk	0.435	0.599149386954389	-
6896804	Exosc9	0.435	0.599149386954389	-
6922202	Zfp37	0.435	0.599149386954389	-
6922229	Wdr31	0.435	0.599149386954389	-
6932367	Ppbp	0.435	0.599149386954389	-
6933491	C130026L21Rik	0.435	0.599149386954389	-
6946475	Fkbp9	0.435	0.599149386954389	-

6949744	Clec4n	0.435	0.599149386954389	-
6953170	Nobox	0.435	0.599149386954389	-
6957465	Csda	0.435	0.599149386954389	-
6960358	Hrc	0.435	0.599149386954389	-
6962787	--	0.435	0.599149386954389	-
6964573	Bub3	0.435	0.599149386954389	-
6977776	Rtbdn	0.435	0.599149386954389	-
6979493	Hsbp1	0.435	0.599149386954389	-
6983935	Dnaja2	0.435	0.599149386954389	-
7010326	Rp2h	0.435	0.599149386954389	-
6783255	Rnft1	0.436	0.599149386954389	+
6788057	Zfp879	0.436	0.599149386954389	-
6803211	Ifi27l2b	0.436	0.599149386954389	-
6805108	Otud6b	0.436	0.599149386954389	-
6813763	Cts8	0.436	0.599149386954389	-
6817902	Actr8	0.436	0.599149386954389	-
6837415	Nfam1	0.436	0.599149386954389	-
6845195	1700021K19Rik	0.436	0.599149386954389	-
6849530	Scube3	0.436	0.599149386954389	-
6871537	Ms4a13	0.436	0.599149386954389	-
6875650	Psd4	0.436	0.599149386954389	-
6881169	Tmc2	0.436	0.599149386954389	-
6883055	Wisp2	0.436	0.599149386954389	-
6887057	Baz2b	0.436	0.599149386954389	-
6888329	Smtnl1	0.436	0.599149386954389	-
6890127	Bmf	0.436	0.599149386954389	-
6907810	Magi3	0.436	0.599149386954389	-
6933627	Oasl1	0.436	0.599149386954389	-
6933913	Tbx3	0.436	0.599149386954389	-
6941205	Mmab	0.436	0.599149386954389	-
6949555	Bcl2l13	0.436	0.599149386954389	-
6956748	Plxnd1	0.436	0.599149386954389	-
6979269	Syce1l	0.436	0.599149386954389	-
6998670	Dag1	0.436	0.599149386954389	-
6748551	--	0.437	0.599149386954389	-
6762326	Optc	0.437	0.599149386954389	-
6766409	Ahi1	0.437	0.599149386954389	-
6768933	Ftcd	0.437	0.599149386954389	-
6792456	Slc25a19	0.437	0.599149386954389	-
6813332	Rnf44	0.437	0.599149386954389	-
6829123	Cdh12	0.437	0.599149386954389	-
6840586	Tnk2	0.437	0.599149386954389	-
6846712	Htr1f	0.437	0.599149386954389	-
6846782	Cadm2	0.437	0.599149386954389	-
6849389	Mslnl	0.437	0.599149386954389	-
6849458	Atp6v0e	0.437	0.599149386954389	-
6861468	Napg	0.437	0.599149386954389	-
6878709	Ube2l6	0.437	0.599149386954389	-
6892890	Wfdc5	0.437	0.599149386954389	-
6913039	Reck	0.437	0.599149386954389	-

6931790	Srd5a3	0.437	0.599149386954389	-
6933672	Pla2g1b	0.437	0.599149386954389	-
6948773	Cntn6	0.437	0.599149386954389	-
6953594	Hoxa3	0.437	0.599149386954389	-
6965069	Gpr123	0.437	0.599149386954389	-
6967593	Gabra5	0.437	0.599149386954389	-
6979636	Cdt1	0.437	0.599149386954389	-
6982786	Clcn3	0.437	0.599149386954389	-
6990423	Dyx1c1	0.437	0.599149386954389	-
7010079	Ddx3x	0.437	0.599149386954389	-
7013909	Armcx3	0.437	0.599149386954389	-
7017621	Dnase1l1	0.437	0.599149386954389	-
6754776	Brp44	0.438	0.599149386954389	-
6771207	Wif1	0.438	0.599149386954389	-
6782134	Pld2	0.438	0.599149386954389	-
6789411	Alox15	0.438	0.599149386954389	-
6795869	Tmx1	0.438	0.599149386954389	-
6797758	Papola	0.438	0.599149386954389	-
6845559	4930455C21Rik	0.438	0.599149386954389	-
6848947	Lix1	0.438	0.599149386954389	-
6849955	Pram1	0.438	0.599149386954389	-
6876944	Lypd6b	0.438	0.599149386954389	-
6880033	Lpcat4	0.438	0.599149386954389	-
6888779	Atg13	0.438	0.599149386954389	-
6900287	Kcna2	0.438	0.599149386954389	-
6913569	Tmem38b	0.438	0.599149386954389	-
6916797	Scmh1	0.438	0.599149386954389	-
6926881	Fbxo6	0.438	0.599149386954389	-
6943389	N4bp2l2	0.438	0.599149386954389	-
6959004	Ercc1	0.438	0.599149386954389	-
6959568	Polr2i	0.438	0.599149386954389	-
6965848	Zfp111	0.438	0.599149386954389	-
6983223	Ifi30	0.438	0.599149386954389	-
7009788	Gripap1	0.438	0.599149386954389	-
6751215	Fbxo36	0.439	0.599149386954389	-
6775380	Fam108a	0.439	0.599149386954389	-
6777177	Tbc1d15	0.439	0.599149386954389	-
6790318	Tada2a	0.439	0.599149386954389	-
6802265	Zfyve1	0.439	0.599149386954389	-
6805825	Irf4	0.439	0.599149386954389	-
6825390	Hmbox1	0.439	0.599149386954389	-
6848361	Rbm16	0.439	0.599149386954389	-
6849622	Fgd2	0.439	0.599149386954389	-
6864565	Hspa9	0.439	0.599149386954389	-
6882774	Fam83d	0.439	0.599149386954389	-
6884027	Cdh26	0.439	0.599149386954389	-
6884100	4930591A17Rik	0.439	0.599149386954389	-
6906652	Fcrls	0.439	0.599149386954389	-
6907313	Gpr89	0.439	0.599149386954389	-
6918699	Mad2l2	0.439	0.599149386954389	-

6934646	4930579G22Rik	0.439	0.599149386954389	-
6946729	Smarcad1	0.439	0.599149386954389	-
6948918	Edem1	0.439	0.599149386954389	-
6951164	Ccdc91	0.439	0.599149386954389	-
6966267	Wdr62	0.439	0.599149386954389	-
6979709	Afg3l1	0.439	0.599149386954389	-
6983326	Calr3	0.439	0.599149386954389	-
6989483	Nptn	0.439	0.599149386954389	-
6752136	Serpinb12	0.44	0.599149386954389	-
6780816	Grm6	0.44	0.599149386954389	-
6810051	Ercc8	0.44	0.599149386954389	-
6815511	Cartpt	0.44	0.599149386954389	-
6819511	Shisa2	0.44	0.599149386954389	-
6828747	Adamts12	0.44	0.599149386954389	-
6879003	Kbtbd4	0.44	0.599149386954389	-
6880982	Tmem127	0.44	0.599149386954389	-
6882066	Cst12	0.44	0.599149386954389	-
6890710	Mal	0.44	0.599149386954389	-
6896857	Spata5	0.44	0.599149386954389	-
6913132	Polr1e	0.44	0.599149386954389	-
6917771	Il28ra	0.44	0.599149386954389	-
6919927	Decr1	0.44	0.599149386954389	-
6925247	Maneal	0.44	0.599149386954389	-
6926392	Padi6	0.44	0.599149386954389	-
6942654	Pdgfa	0.44	0.599149386954389	-
6974138	Lamp1	0.44	0.599149386954389	-
6976251	Fbxo8	0.44	0.599149386954389	-
6978825	Tmem208	0.44	0.599149386954389	-
6982153	Ankrd37	0.44	0.599149386954389	-
6992465	Prss42	0.44	0.599149386954389	-
6994830	Gramd1b	0.44	0.599149386954389	-
6994927	Sorl1	0.44	0.599149386954389	-
7011759	Fmr1nb	0.44	0.599149386954389	-
7011990	Tktl1	0.44	0.599149386954389	-
6754700	4930455F23Rik	0.441	0.599149386954389	-
6801102	Sec23a	0.441	0.599149386954389	-
6806791	Cap2	0.441	0.599149386954389	-
6811496	Zfp187	0.441	0.599149386954389	-
6813740	4930486L24Rik	0.441	0.599149386954389	-
6825704	Bmp1	0.441	0.599149386954389	-
6832420	Tbc1d22a	0.441	0.599149386954389	-
6839743	Crkl	0.441	0.599149386954389	-
6871372	Tmem179b	0.441	0.599149386954389	-
6871627	Dtx4	0.441	0.599149386954389	-
6876217	Sh2d3c	0.441	0.599149386954389	-
6880703	AA467197	0.441	0.599149386954389	-
6898995	Cd5l	0.441	0.599149386954389	-
6899019	Insrr	0.441	0.599149386954389	-
6935579	Gsx1	0.441	0.599149386954389	-
6937052	Preb	0.441	0.599149386954389	-

6945963	Zfp212	0.441	0.599149386954389	-
6966298	Zbtb32	0.441	0.599149386954389	-
6978870	Edc4	0.441	0.599149386954389	-
6979613	Zfpm1	0.441	0.599149386954389	-
6981932	Frg1	0.441	0.599149386954389	+
6992864	Ctdspl	0.441	0.599149386954389	-
7015375	Clcn5	0.441	0.599149386954389	-
6751304	Dis3l2	0.442	0.599149386954389	-
6757479	Bai3	0.442	0.599149386954389	-
6775419	Creb3l3	0.442	0.599149386954389	-
6784039	Psmd3	0.442	0.599149386954389	-
6788128	N4bp3	0.442	0.599149386954389	-
6789531	Slc13a5	0.442	0.599149386954389	-
6796403	Galntl1	0.442	0.599149386954389	-
6796719	1700020O03Rik	0.442	0.599149386954389	-
6807209	Zfp346	0.442	0.599149386954389	-
6822297	Clybl	0.442	0.599149386954389	-
6844601	St6gal1	0.442	0.599149386954389	-
6855202	Ppp1r11	0.442	0.599149386954389	-
6868193	Lpxn	0.442	0.599149386954389	-
6882057	Gzf1	0.442	0.599149386954389	-
6885431	Tmem141	0.442	0.599149386954389	-
6895856	Car2	0.442	0.599149386954389	-
6917039	Sf3a3	0.442	0.599149386954389	-
6918852	Gpr157	0.442	0.599149386954389	-
6921008	Aqp7	0.442	0.599149386954389	-
6921479	Erp44	0.442	0.599149386954389	-
6935112	Zscan21	0.442	0.599149386954389	-
6950168	Klre1	0.442	0.599149386954389	-
6957140	Gpr162	0.442	0.599149386954389	-
6959461	Lgals7	0.442	0.599149386954389	-
6964329	Phkg2	0.442	0.599149386954389	-
6969399	Ankrd42	0.442	0.599149386954389	-
6992377	Uqcrc1	0.442	0.599149386954389	-
7018857	2610002M06Rik	0.442	0.599149386954389	-
6783309	Tex14	0.443	0.599149386954389	-
6784049	Casc3	0.443	0.599149386954389	-
6788271	Leap2	0.443	0.599149386954389	-
6789444	Pfn1	0.443	0.599149386954389	-
6789948	Supt6h	0.443	0.599149386954389	-
6796711	1700019E19Rik	0.443	0.599149386954389	-
6825683	Pdlim2	0.443	0.599149386954389	-
6842326	Robo1	0.443	0.599149386954389	-
6873410	Actr1a	0.443	0.599149386954389	-
6880987	Dusp2	0.443	0.599149386954389	-
6896524	Cldn11	0.443	0.599149386954389	-
6905408	P2ry14	0.443	0.599149386954389	-
6920754	Mobkl2b	0.443	0.599149386954389	-
6936679	Srpk2	0.443	0.599149386954389	-
6936939	Mnx1	0.443	0.599149386954389	-

6979515	Klhl36	0.443	0.599149386954389	-
6752073	Tnfrsf11a	0.444	0.599149386954389	-
6754444	Tnr	0.444	0.599149386954389	-
6762106	Pfkfb2	0.444	0.599149386954389	-
6764043	Sdhc	0.444	0.599149386954389	-
6768907	Slc5a4a	0.444	0.599149386954389	-
6785684	Nefh	0.444	0.599149386954389	-
6796612	Vsx2	0.444	0.599149386954389	-
6800726	6530401N04Rik	0.444	0.599149386954389	-
6825410	Fzd3	0.444	0.599149386954389	-
6830505	Depdc6	0.444	0.599149386954389	-
6840207	Tprg	0.444	0.599149386954389	-
6849891	Cyp4f40	0.444	0.599149386954389	-
6864607	Dnajc18	0.444	0.599149386954389	-
6869976	Lzts2	0.444	0.599149386954389	-
6873246	Erlin1	0.444	0.599149386954389	-
6875421	Msrb2	0.444	0.599149386954389	-
6885671	6530402F18Rik	0.444	0.599149386954389	-
6888696	Ptprj	0.444	0.599149386954389	-
6916502	4732418C07Rik	0.444	0.599149386954389	-
6941934	Scarb1	0.444	0.599149386954389	-
6942395	Wbscr28	0.444	0.599149386954389	-
6944734	Asb15	0.444	0.599149386954389	-
6954557	Smyd1	0.444	0.599149386954389	-
6955770	Uba3	0.444	0.599149386954389	-
6966958	Dkkl1	0.444	0.599149386954389	-
6976200	Vegfc	0.444	0.599149386954389	-
6979196	Terf2ip	0.444	0.599149386954389	-
6990543	Leo1	0.444	0.599149386954389	-
6995258	Tagln	0.444	0.599149386954389	-
6764138	Atp1a2	0.445	0.599149386954389	-
6771603	BC089597	0.445	0.599149386954389	-
6780438	Rnf145	0.445	0.599149386954389	-
6790087	1110002N22Rik	0.445	0.599149386954389	-
6794181	Sh3yl1	0.445	0.599149386954389	-
6803917	Jag2	0.445	0.599149386954389	-
6806960	Ogn	0.445	0.599149386954389	-
6813387	Lman2	0.445	0.599149386954389	-
6839754	Smpd4	0.445	0.599149386954389	-
6860800	Fam170a	0.445	0.599149386954389	-
6875697	Fam166a	0.445	0.599149386954389	-
6886639	Mmadhc	0.445	0.599149386954389	-
6897349	Naa15	0.445	0.599149386954389	-
6899277	She	0.445	0.599149386954389	-
6900044	Nhlh2	0.445	0.599149386954389	-
6907126	Psmd4	0.445	0.599149386954389	-
6912820	Aco1	0.445	0.599149386954389	-
6953063	Moxd2	0.445	0.599149386954389	-
6957463	Styk1	0.445	0.599149386954389	-
6962961	Fam168a	0.445	0.599149386954389	-

6964261	Cdipt	0.445	0.599149386954389	-
6971219	4933440M02Rik	0.445	0.599149386954389	-
6987685	Opcml	0.445	0.599149386954389	-
6996228	Pias1	0.445	0.599149386954389	-
6754517	Serpinc1	0.446	0.599149386954389	-
6769144	Fstl3	0.446	0.599149386954389	-
6783489	Mmd	0.446	0.599149386954389	-
6818500	Mbl1	0.446	0.599149386954389	-
6838716	Itgb7	0.446	0.599149386954389	-
6846463	2610528E23Rik	0.446	0.599149386954389	-
6847819	1110004E09Rik	0.446	0.599149386954389	-
6871903	Ostf1	0.446	0.599149386954389	-
6885645	Barhl1	0.446	0.599149386954389	-
6892993	Zfp663	0.446	0.599149386954389	-
6919309	Sdr16c5	0.446	0.599149386954389	-
6939057	Corin	0.446	0.599149386954389	-
6966252	Zfp82	0.446	0.599149386954389	-
6970484	Galntl4	0.446	0.599149386954389	-
6989534	Adpgk	0.446	0.599149386954389	+
7010338	Uba1	0.446	0.599149386954389	-
6750639	Inha	0.447	0.599149386954389	-
6775298	Ppap2c	0.447	0.599149386954389	-
6778784	Ikzf1	0.447	0.599149386954389	-
6788594	4930504O13Rik	0.447	0.599149386954389	-
6795448	Sip1	0.447	0.599149386954389	-
6807154	Sfxn1	0.447	0.599149386954389	-
6817958	Glt8d1	0.447	0.599149386954389	-
6822173	Rap2a	0.447	0.599149386954389	-
6826279	Wbp4	0.447	0.599149386954389	-
6834975	Rnf19a	0.447	0.599149386954389	-
6870975	Aip	0.447	0.599149386954389	-
6882442	U46068	0.447	0.599149386954389	-
6913901	Hsdl2	0.447	0.599149386954389	-
6926504	Tmem51	0.447	0.599149386954389	-
6941364	Ccdc60	0.447	0.599149386954389	-
6941374	Hspb8	0.447	0.599149386954389	-
6949789	C1rl	0.447	0.599149386954389	-
6950770	Golt1b	0.447	0.599149386954389	-
6955653	Magi1	0.447	0.599149386954389	-
6961896	Isg20	0.447	0.599149386954389	-
6964662	Zranb1	0.447	0.599149386954389	-
6978928	Tmco7	0.447	0.599149386954389	-
6979267	Mon1b	0.447	0.599149386954389	-
6988222	Vsig2	0.447	0.599149386954389	-
6989249	Fbxo22	0.447	0.599149386954389	-
6992348	Qrich1	0.447	0.599149386954389	-
6993760	Kri1	0.447	0.599149386954389	-
7017788	Pbsn	0.447	0.599149386954389	-
6778506	Dbnl	0.448	0.599149386954389	-
6781259	2210415F13Rik	0.448	0.599149386954389	-

6788725	Pemt	0.448	0.599149386954389	-
6797510	Ubr7	0.448	0.599149386954389	-
6807169	4732471D19Rik	0.448	0.599149386954389	-
6813771	Cts3	0.448	0.599149386954389	-
6823302	Dlg5	0.448	0.599149386954389	-
6823889	Bmpr1a	0.448	0.599149386954389	-
6825445	Ptk2b	0.448	0.599149386954389	-
6842803	Gabpa	0.448	0.599149386954389	-
6852130	Ypel5	0.448	0.599149386954389	-
6854151	3110048L19Rik	0.448	0.599149386954389	-
6855898	Dazl	0.448	0.599149386954389	-
6859775	Sap130	0.448	0.599149386954389	-
6860111	0610010O12Rik	0.448	0.599149386954389	-
6867711	Brms1	0.448	0.599149386954389	-
6885803	Tor1a	0.448	0.599149386954389	-
6909972	Pdlim5	0.448	0.599149386954389	-
6910279	Rpf1	0.448	0.599149386954389	-
6921561	Aldob	0.448	0.599149386954389	-
6923142	Ptplad2	0.448	0.599149386954389	-
6929710	Slc4a1ap	0.448	0.599149386954389	-
6930371	Bst1	0.448	0.599149386954389	-
6934127	Gpn3	0.448	0.599149386954389	-
6945950	Zfp398	0.448	0.599149386954389	-
6955320	Eefsec	0.448	0.599149386954389	-
6958922	Ptgir	0.448	0.599149386954389	-
6966159	C330005M16Rik	0.448	0.599149386954389	-
6977787	Fbxw9	0.448	0.599149386954389	-
6980476	Tubgcp3	0.448	0.599149386954389	-
6987444	Pigyl	0.448	0.599149386954389	-
6755054	Rgs5	0.449	0.599149386954389	-
6758027	Tbc1d8	0.449	0.599149386954389	-
6762964	BC003331	0.449	0.599149386954389	-
6763731	Xcl1	0.449	0.599149386954389	-
6767828	Dcbld1	0.449	0.599149386954389	-
6769198	1600002K03Rik	0.449	0.599149386954389	-
6775242	D10Jhu81e	0.449	0.599149386954389	-
6775456	Gipc3	0.449	0.599149386954389	-
6788076	Prop1	0.449	0.599149386954389	-
6807230	Slc34a1	0.449	0.599149386954389	-
6817672	Arf4	0.449	0.599149386954389	-
6837435	A4galt	0.449	0.599149386954389	-
6838565	Pou6f1	0.449	0.599149386954389	-
6843886	Nubp1	0.449	0.599149386954389	-
6844362	Mrpl40	0.449	0.599149386954389	-
6846646	Nsun3	0.449	0.599149386954389	-
6859290	Ttr	0.449	0.599149386954389	-
6872783	Lipa	0.449	0.599149386954389	-
6886204	Ppp6c	0.449	0.599149386954389	-
6886768	Prpf40a	0.449	0.599149386954389	-
6893292	Sall4	0.449	0.599149386954389	-

6913348	Tmeff1	0.449	0.599149386954389	-
6915997	C8b	0.449	0.599149386954389	-
6925165	Macf1	0.449	0.599149386954389	-
6929132	Lrrc17	0.449	0.599149386954389	-
6944380	Met	0.449	0.599149386954389	-
6946370	2410066E13Rik	0.449	0.599149386954389	-
6965984	Cyp2s1	0.449	0.599149386954389	-
6966875	Pold1	0.449	0.599149386954389	-
6972114	Caly	0.449	0.599149386954389	-
6972413	Tnfrsf22	0.449	0.599149386954389	-
6978836	Hsd11b2	0.449	0.599149386954389	-
6985004	Psmb10	0.449	0.599149386954389	-
6988749	Pcsk7	0.449	0.599149386954389	-
6990685	Gsta4	0.449	0.599149386954389	-
6995526	1110032A03Rik	0.449	0.599149386954389	+
7002998	Tpbpb	0.449	0.599149386954389	-
6747326	Oprk1	0.45	0.599149386954389	-
6751647	Aqp12	0.45	0.599149386954389	-
6759042	Ica1l	0.45	0.599149386954389	-
6760177	Irs1	0.45	0.599149386954389	-
6778052	Suox	0.45	0.599149386954389	-
6781329	Wnt9a	0.45	0.599149386954389	-
6783744	Spop	0.45	0.599149386954389	-
6785434	Tspan10	0.45	0.599149386954389	-
6814957	Xrcc4	0.45	0.599149386954389	-
6828743	Slc45a2	0.45	0.599149386954389	-
6838823	Ppp1r1a	0.45	0.599149386954389	+
6843494	Igsf5	0.45	0.599149386954389	-
6859800	Iws1	0.45	0.599149386954389	-
6864597	2010001M09Rik	0.45	0.599149386954389	-
6874653	Ccdc3	0.45	0.599149386954389	-
6882441	2310021H06Rik	0.45	0.599149386954389	-
6888153	Nckap1	0.45	0.599149386954389	-
6918318	Gm13103	0.45	0.599149386954389	-
6922649	3110001D03Rik	0.45	0.599149386954389	-
6925917	Zdhhc18	0.45	0.599149386954389	+
6931759	Kit	0.45	0.599149386954389	-
6949206	Tsen2	0.45	0.599149386954389	-
6953461	Tra2a	0.45	0.599149386954389	-
6953507	Osbpl3	0.45	0.599149386954389	-
6962577	Rab30	0.45	0.599149386954389	-
6964207	Rabep2	0.45	0.599149386954389	-
6981497	Mak16	0.45	0.599149386954389	-
6991264	Ctsh	0.45	0.599149386954389	-
6747314	Atp6v1h	0.451	0.599149386954389	-
6748174	B3gat2	0.451	0.599149386954389	+
6762796	Rgs1	0.451	0.599149386954389	-
6764662	Wdr26	0.451	0.599149386954389	-
6778028	Obfc2b	0.451	0.599149386954389	-
6782656	Flot2	0.451	0.599149386954389	+

6788020	Canx	0.451	0.599149386954389	-
6795947	Jkamp	0.451	0.599149386954389	-
6811121	Ggps1	0.451	0.599149386954389	-
6811924	Prl7c1	0.451	0.599149386954389	-
6848062	Hlcs	0.451	0.599149386954389	-
6862195	Smad2	0.451	0.599149386954389	-
6919417	Tox	0.451	0.599149386954389	-
6929306	Chpf2	0.451	0.599149386954389	-
6929660	Preb	0.451	0.599149386954389	-
6929728	Ppp1cb	0.451	0.599149386954389	-
6942589	Gjc3	0.451	0.599149386954389	-
6963418	Wee1	0.451	0.599149386954389	-
6965123	1190003J15Rik	0.451	0.599149386954389	-
6965970	Tmem91	0.451	0.599149386954389	-
6966358	Gramd1a	0.451	0.599149386954389	-
6990952	Bckdhb	0.451	0.599149386954389	-
7019854	Gucy2f	0.451	0.599149386954389	-
7023079	Ddx3y	0.451	0.599149386954389	+
6762397	Gpr37l1	0.452	0.599149386954389	-
6771623	Ptges3	0.452	0.599149386954389	-
6777898	Tsfm	0.452	0.599149386954389	-
6781443	Alkbh5	0.452	0.599149386954389	-
6784283	Rdm1	0.452	0.599149386954389	-
6789367	Slc2a4	0.452	0.599149386954389	-
6816247	Itga2	0.452	0.599149386954389	-
6831927	Cby1	0.452	0.599149386954389	-
6837263	Slc25a17	0.452	0.599149386954389	-
6838257	Slc38a2	0.452	0.599149386954389	+
6839771	Scarf2	0.452	0.599149386954389	-
6845562	Poglut1	0.452	0.599149386954389	-
6851734	Ppp4r1	0.452	0.599149386954389	-
6854346	1600002H07Rik	0.452	0.599149386954389	-
6854871	Akap8	0.452	0.599149386954389	-
6871199	Arl2	0.452	0.599149386954389	-
6890980	Adam33	0.452	0.599149386954389	-
6899510	Tchhl1	0.452	0.599149386954389	-
6907424	Hsd3b2	0.452	0.599149386954389	-
6910610	Gipc2	0.452	0.599149386954389	-
6917496	Sesn2	0.452	0.599149386954389	-
6918705	Fbxo2	0.452	0.599149386954389	-
6934854	Gatsl2	0.452	0.599149386954389	-
6934892	Stx1a	0.452	0.599149386954389	-
6953051	E330009J07Rik	0.452	0.599149386954389	-
6960834	Siglech	0.452	0.599149386954389	+
6963590	Spon1	0.452	0.599149386954389	-
6968913	Fam154b	0.452	0.599149386954389	-
6984975	Atp6v0d1	0.452	0.599149386954389	-
6748535	Ptpn18	0.453	0.599299265785609	-
6754681	Sell	0.453	0.599299265785609	-
6757120	Stau2	0.453	0.599299265785609	-

6757994	Rev1	0.453	0.599299265785609	-
6768572	Tmem26	0.453	0.599299265785609	-
6789979	Nlk	0.453	0.599299265785609	-
6818994	AY358078	0.453	0.599299265785609	-
6819228	Dhrs2	0.453	0.599299265785609	-
6832332	Nup50	0.453	0.599299265785609	-
6837375	Cyp2d22	0.453	0.599299265785609	-
6861850	Tcf4	0.453	0.599299265785609	-
6882181	Pygb	0.453	0.599299265785609	-
6901573	Tacr3	0.453	0.599299265785609	-
6926936	Pgd	0.453	0.599299265785609	-
6932517	Sep11	0.453	0.599299265785609	-
6953920	Ppm1k	0.453	0.599299265785609	-
6979097	Vac14	0.453	0.599299265785609	-
6983255	Abhd8	0.453	0.599299265785609	+
6992091	Cpne4	0.453	0.599299265785609	-
6995233	Cep164	0.453	0.599299265785609	-
6765730	Ppil4	0.454	0.599331444759207	-
6774146	Serinc1	0.454	0.599331444759207	-
6775257	Rrp1	0.454	0.599331444759207	-
6781307	Trim11	0.454	0.599331444759207	-
6791410	Krt17	0.454	0.599331444759207	-
6824962	Xpo4	0.454	0.599331444759207	-
6833029	Slc48a1	0.454	0.599331444759207	-
6850149	H2-Q10	0.454	0.599331444759207	-
6858247	Acat3	0.454	0.599331444759207	-
6870070	Sufu	0.454	0.599331444759207	-
6891898	Napb	0.454	0.599331444759207	-
6916586	Tesk2	0.454	0.599331444759207	-
6917931	1700013G24Rik	0.454	0.599331444759207	-
6921012	Aqp3	0.454	0.599331444759207	-
6925587	Ccdc28b	0.454	0.599331444759207	-
6946396	Gars	0.454	0.599331444759207	-
6956487	5031434C07Rik	0.454	0.599331444759207	-
6959463	Capn12	0.454	0.599331444759207	-
6960295	Vrk3	0.454	0.599331444759207	-
6965191	Tmem80	0.454	0.599331444759207	-
6978215	Irx5	0.454	0.599331444759207	-
6993797	Tmed1	0.454	0.599331444759207	-
6768237	Aifm2	0.455	0.599831235976978	-
6771608	Sdr9c7	0.455	0.599831235976978	-
6783909	Mrpl45	0.455	0.599831235976978	-
6810416	4930544M13Rik	0.455	0.599831235976978	-
6813570	Hnrnpk	0.455	0.599831235976978	-
6819547	Mtmr6	0.455	0.599831235976978	-
6871471	Fads2	0.455	0.599831235976978	-
6880549	Mga	0.455	0.599831235976978	-
6897556	Trpc4	0.455	0.599831235976978	-
6916023	Ppap2b	0.455	0.599831235976978	+
6935109	Azgp1	0.455	0.599831235976978	-

6940303	Rasgef1b	0.455	0.599831235976978	-
6942606	Taf6	0.455	0.599831235976978	-
6959180	Zfp574	0.455	0.599831235976978	-
6764053	Ppox	0.456	0.599862162951426	-
6783020	Ap2b1	0.456	0.599862162951426	-
6794293	Nampt	0.456	0.599862162951426	-
6813084	Wnk2	0.456	0.599862162951426	-
6829945	Atp6v1c1	0.456	0.599862162951426	-
6849952	Zfp414	0.456	0.599862162951426	-
6852483	Morn2	0.456	0.599862162951426	-
6852939	Gtf2a1l	0.456	0.599862162951426	-
6855717	Ppp2r5d	0.456	0.599862162951426	-
6867715	Rin1	0.456	0.599862162951426	-
6871879	Pcsk5	0.456	0.599862162951426	-
6873068	Sorbs1	0.456	0.599862162951426	-
6881306	Cds2	0.456	0.599862162951426	-
6882499	a	0.456	0.599862162951426	-
6885399	Grin1	0.456	0.599862162951426	-
6892991	Elmo2	0.456	0.599862162951426	-
6901735	Adh7	0.456	0.599862162951426	-
6916762	Lepre1	0.456	0.599862162951426	-
6934942	Mdh2	0.456	0.599862162951426	-
6935594	Pan3	0.456	0.599862162951426	-
6963123	Olfr65	0.456	0.599862162951426	-
7018634	Nap1l2	0.456	0.599862162951426	-
6765301	Hhat	0.457	0.60009811906147	-
6774451	Sirt1	0.457	0.60009811906147	-
6785626	4921536K21Rik	0.457	0.60009811906147	-
6813666	Golm1	0.457	0.60009811906147	-
6817396	Adk	0.457	0.60009811906147	-
6842938	ORF63	0.457	0.60009811906147	-
6870489	Pdcd4	0.457	0.60009811906147	+
6884265	Zgpat	0.457	0.60009811906147	-
6890851	F830045P16Rik	0.457	0.60009811906147	-
6949797	Lpcat3	0.457	0.60009811906147	-
6954572	Kdm3a	0.457	0.60009811906147	-
6959460	Lgals4	0.457	0.60009811906147	-
6966960	Cd37	0.457	0.60009811906147	-
6981991	Triml1	0.457	0.60009811906147	-
6983950	Neto2	0.457	0.60009811906147	-
6754830	Gpa33	0.458	0.60009811906147	-
6777677	Mon2	0.458	0.60009811906147	-
6784246	Cntnap1	0.458	0.60009811906147	-
6784331	Tmub2	0.458	0.60009811906147	-
6793181	1110057K04Rik	0.458	0.60009811906147	-
6832542	1300018J18Rik	0.458	0.60009811906147	-
6835984	Mtss1	0.458	0.60009811906147	-
6848209	Ripk4	0.458	0.60009811906147	-
6850132	Bat1a	0.458	0.60009811906147	-
6854944	Hnrnpm	0.458	0.60009811906147	-

6884351	Suv39h2	0.458	0.60009811906147	-
6892145	Zcchc3	0.458	0.60009811906147	-
6894274	BC006779	0.458	0.60009811906147	-
6912885	Chmp5	0.458	0.60009811906147	-
6914521	Kdm4c	0.458	0.60009811906147	-
6923405	Mysm1	0.458	0.60009811906147	-
6926655	Tnfrsf8	0.458	0.60009811906147	-
6926916	Tardbp	0.458	0.60009811906147	-
6936760	Prkag2	0.458	0.60009811906147	-
6952362	Opn1sw	0.458	0.60009811906147	-
6952812	Svopl	0.458	0.60009811906147	-
6957389	4922502D21Rik	0.458	0.60009811906147	-
6962752	Ndufc2	0.458	0.60009811906147	-
7011936	Bgn	0.458	0.60009811906147	-
7015061	Gpm6b	0.458	0.60009811906147	-
7018750	Atrx	0.458	0.60009811906147	-
6789325	Cd68	0.459	0.600130430594355	+
6795033	Coch	0.459	0.600130430594355	-
6802102	Dcaf5	0.459	0.600130430594355	-
6806039	Serpinb1b	0.459	0.600130430594355	-
6824842	2610027L16Rik	0.459	0.600130430594355	-
6841187	Btla	0.459	0.600130430594355	-
6849434	Axin1	0.459	0.600130430594355	-
6853899	Thbs2	0.459	0.600130430594355	-
6854404	Tmem204	0.459	0.600130430594355	-
6854909	Cyp4f14	0.459	0.600130430594355	-
6857834	Prepl	0.459	0.600130430594355	-
6862318	Pstpip2	0.459	0.600130430594355	-
6864522	Cdc23	0.459	0.600130430594355	-
6872496	Ak3	0.459	0.600130430594355	-
6878000	Dync1i2	0.459	0.600130430594355	-
6925362	Eif2c3	0.459	0.600130430594355	-
6926033	Tmem57	0.459	0.600130430594355	-
6942544	Srrt	0.459	0.600130430594355	-
6964734	Bccip	0.459	0.600130430594355	-
6965527	Zik1	0.459	0.600130430594355	-
7013837	Srpx2	0.459	0.600130430594355	-
6792507	Foxj1	0.46	0.600130430594355	-
6812320	Lyrm4	0.46	0.600130430594355	-
6866354	4933403F05Rik	0.46	0.600130430594355	-
6887901	Lnp	0.46	0.600130430594355	-
6897777	2810407C02Rik	0.46	0.600130430594355	-
6908886	Fnbp1l	0.46	0.600130430594355	-
6909520	Arhgef38	0.46	0.600130430594355	-
6926591	Pramel5	0.46	0.600130430594355	-
6941148	Crybb3	0.46	0.600130430594355	-
6950750	Iapp	0.46	0.600130430594355	-
6957083	Clec4e	0.46	0.600130430594355	-
6959221	Atp5sl	0.46	0.600130430594355	-
6959309	2310022A10Rik	0.46	0.600130430594355	-

6963857	9030624J02Rik	0.46	0.600130430594355	-
6966193	Psmd8	0.46	0.600130430594355	-
6975861	Sorbs2	0.46	0.600130430594355	-
6985408	Cfdp1	0.46	0.600130430594355	-
6989019	Timm8b	0.46	0.600130430594355	-
6993833	Spc24	0.46	0.600130430594355	-
6997109	E330016A19Rik	0.46	0.600130430594355	-
6997994	2610101N10Rik	0.46	0.600130430594355	-
6998401	Cdv3	0.46	0.600130430594355	-
6998584	Abhd14a	0.46	0.600130430594355	-
6752980	Yod1	0.461	0.600130430594355	-
6760296	Trip12	0.461	0.600130430594355	-
6762370	4931440L10Rik	0.461	0.600130430594355	-
6784038	Gsdma	0.461	0.600130430594355	-
6788836	Rnf112	0.461	0.600130430594355	-
6791482	Becn1	0.461	0.600130430594355	-
6797580	Serpina3m	0.461	0.600130430594355	-
6822477	Fam107a	0.461	0.600130430594355	-
6836979	Txn2	0.461	0.600130430594355	-
6838483	Lass5	0.461	0.600130430594355	-
6862102	Acaa2	0.461	0.600130430594355	-
6863096	Zfp438	0.461	0.600130430594355	-
6865709	Aldh7a1	0.461	0.600130430594355	-
6875742	Lcn8	0.461	0.600130430594355	-
6876010	Med27	0.461	0.600130430594355	-
6877593	Slc4a10	0.461	0.600130430594355	-
6893109	Stau1	0.461	0.600130430594355	-
6935074	Trfr2	0.461	0.600130430594355	-
6936076	Adam22	0.461	0.600130430594355	-
6942553	Gnb2	0.461	0.600130430594355	-
6965885	Cd177	0.461	0.600130430594355	-
6977454	Slc10a7	0.461	0.600130430594355	-
6978355	Gpr97	0.461	0.600130430594355	-
6755146	Fcgr4	0.462	0.600333461538462	-
6758862	Satb2	0.462	0.600333461538462	-
6772417	Tnfaip3	0.462	0.600333461538462	-
6780983	Sep8	0.462	0.600333461538462	-
6781561	Ttc19	0.462	0.600333461538462	-
6791156	Pnpo	0.462	0.600333461538462	-
6867966	Hnrnpul2	0.462	0.600333461538462	-
6899679	Arnt	0.462	0.600333461538462	-
6902174	2410004B18Rik	0.462	0.600333461538462	-
6917690	Rhd	0.462	0.600333461538462	-
6925562	Zbtb8b	0.462	0.600333461538462	-
6941883	Rilpl2	0.462	0.600333461538462	-
6943448	Rfc3	0.462	0.600333461538462	-
6963197	Cckbr	0.462	0.600333461538462	-
6969028	Fah	0.462	0.600333461538462	+
6974630	Dkk4	0.462	0.600333461538462	-
6984993	Gfod2	0.462	0.600333461538462	-

6995256	Rnf214	0.462	0.600333461538462	-
7015995	Zfp300	0.462	0.600333461538462	-
6750600	Ankzf1	0.463	0.600852433882714	-
6765153	Smyd2	0.463	0.600852433882714	-
6791215	Pip4k2b	0.463	0.600852433882714	-
6823213	Dusp13	0.463	0.600852433882714	-
6828657	Ugt3a2	0.463	0.600852433882714	-
6832321	Prr5	0.463	0.600852433882714	-
6876209	St6galnac4	0.463	0.600852433882714	+
6937426	Cpz	0.463	0.600852433882714	-
6942936	Ankrd61	0.463	0.600852433882714	-
6942973	Tmem130	0.463	0.600852433882714	-
6957763	Arhgdib	0.463	0.600852433882714	-
6966869	2310044H10Rik	0.463	0.600852433882714	-
6968776	Unc45a	0.463	0.600852433882714	-
6761964	Tmem163	0.464	0.600852433882714	-
6762944	Pla2g4a	0.464	0.600852433882714	-
6768866	Snrpd3	0.464	0.600852433882714	-
6775155	Gstt2	0.464	0.600852433882714	-
6789319	Shbg	0.464	0.600852433882714	-
6791639	Eftud2	0.464	0.600852433882714	-
6793226	Sdc1	0.464	0.600852433882714	-
6807268	Catsper3	0.464	0.600852433882714	-
6824873	Gzmg	0.464	0.600852433882714	-
6868902	Kcnv2	0.464	0.600852433882714	-
6882616	0610011L14Rik	0.464	0.600852433882714	-
6890302	Zfp106	0.464	0.600852433882714	-
6913009	Tesk1	0.464	0.600852433882714	-
6922244	Pole3	0.464	0.600852433882714	-
6924684	Foxd2	0.464	0.600852433882714	-
6933632	Sppl3	0.464	0.600852433882714	-
6937821	Tapt1	0.464	0.600852433882714	-
6945335	Cald1	0.464	0.600852433882714	-
6952436	Tsga14	0.464	0.600852433882714	-
6965773	Ppm1n	0.464	0.600852433882714	-
6965777	Cd3eap	0.464	0.600852433882714	-
6966754	Vmn2r60	0.464	0.600852433882714	-
6975235	Gsr	0.464	0.600852433882714	-
6749852	Icos	0.465	0.600938127570049	-
6752497	Tmem185b	0.465	0.600938127570049	-
6769617	Ccdc53	0.465	0.600938127570049	-
6773525	2410017P07Rik	0.465	0.600938127570049	-
6782099	Asgr2	0.465	0.600938127570049	-
6785641	1700020C11Rik	0.465	0.600938127570049	-
6791970	Smurf2	0.465	0.600938127570049	-
6814491	Ankrd32	0.465	0.600938127570049	-
6838386	Adcy6	0.465	0.600938127570049	-
6838405	Prkag1	0.465	0.600938127570049	-
6844099	Ifitm7	0.465	0.600938127570049	-
6849347	0610007P22Rik	0.465	0.600938127570049	-

6871139	Mtvr2	0.465	0.600938127570049	-
6876357	D730039F16Rik	0.465	0.600938127570049	-
6900014	Igsf3	0.465	0.600938127570049	-
6906834	Zbtb7b	0.465	0.600938127570049	-
6911716	1110037F02Rik	0.465	0.600938127570049	-
6937282	Whsc2	0.465	0.600938127570049	-
6940361	Coq2	0.465	0.600938127570049	-
6977971	Heatr3	0.465	0.600938127570049	-
6997632	Syncrip	0.465	0.600938127570049	-
6759459	Acadl	0.466	0.601681571169797	-
6782554	Slc6a4	0.466	0.601681571169797	-
6782626	Sez6	0.466	0.601681571169797	-
6785307	Birc5	0.466	0.601681571169797	-
6785460	Anapc11	0.466	0.601681571169797	-
6822123	Cldn10	0.466	0.601681571169797	-
6833175	Aqp5	0.466	0.601681571169797	-
6852438	Fam82a1	0.466	0.601681571169797	-
6963047	Stim1	0.466	0.601681571169797	-
6819694	Ctsb	0.467	0.601681571169797	-
6862258	Pias2	0.467	0.601681571169797	-
6868021	Fads1	0.467	0.601681571169797	-
6869783	Ankrd2	0.467	0.601681571169797	-
6878712	Slc43a1	0.467	0.601681571169797	-
6881837	Sec23b	0.467	0.601681571169797	-
6883273	Ddx27	0.467	0.601681571169797	-
6886142	Dennd1a	0.467	0.601681571169797	-
6886525	Orc4	0.467	0.601681571169797	-
6893303	Zfp64	0.467	0.601681571169797	-
6896611	Ndufb5	0.467	0.601681571169797	-
6899585	Mrpl9	0.467	0.601681571169797	-
6912518	Ube2j1	0.467	0.601681571169797	-
6945090	Klhdc10	0.467	0.601681571169797	-
6947391	Slc4a5	0.467	0.601681571169797	-
6950111	Clec2g	0.467	0.601681571169797	-
6957125	Eno2	0.467	0.601681571169797	-
6965076	Zfp511	0.467	0.601681571169797	-
6972342	Trpm5	0.467	0.601681571169797	-
6980547	Dcun1d2	0.467	0.601681571169797	-
6996269	Map2k1	0.467	0.601681571169797	-
7011581	C230004F18Rik	0.467	0.601681571169797	-
7013332	Tex16	0.467	0.601681571169797	-
6766350	Ifngr1	0.468	0.601936994384696	-
6771662	Smarcc2	0.468	0.601936994384696	-
6782091	Phf23	0.468	0.601936994384696	-
6785753	Gck	0.468	0.601936994384696	-
6791399	Krt32	0.468	0.601936994384696	-
6813795	Fbp2	0.468	0.601936994384696	-
6813881	1110018J18Rik	0.468	0.601936994384696	-
6838979	Zfp263	0.468	0.601936994384696	-
6849162	Zfp758	0.468	0.601936994384696	-

6858784	Thoc1	0.468	0.601936994384696	-
6871457	Incenp	0.468	0.601936994384696	-
6878267	Hoxd9	0.468	0.601936994384696	-
6892799	Jph2	0.468	0.601936994384696	-
6907304	Polr3c	0.468	0.601936994384696	-
6933306	Dr1	0.468	0.601936994384696	-
6948328	Fam19a1	0.468	0.601936994384696	+
6953064	BC048599	0.468	0.601936994384696	-
6971245	Xpo6	0.468	0.601936994384696	-
6747861	Pi15	0.469	0.602027515208236	-
6759767	Ccdc108	0.469	0.602027515208236	-
6774399	Vps26a	0.469	0.602027515208236	-
6800890	Eapp	0.469	0.602027515208236	-
6838383	Ccnt1	0.469	0.602027515208236	-
6850258	Trim31	0.469	0.602027515208236	-
6865723	March3	0.469	0.602027515208236	-
6867593	1810055G02Rik	0.469	0.602027515208236	-
6906900	Snapin	0.469	0.602027515208236	-
6935328	C330006K01Rik	0.469	0.602027515208236	-
6937073	Ppm1g	0.469	0.602027515208236	-
6953424	Gimap3	0.469	0.602027515208236	-
6957261	9630033F20Rik	0.469	0.602027515208236	-
6988653	Ddx6	0.469	0.602027515208236	-
6991716	Rbp2	0.469	0.602027515208236	-
6992372	Nckipsd	0.469	0.602027515208236	-
6995282	4931429L15Rik	0.469	0.602027515208236	-
6751717	Ing5	0.47	0.602027515208236	-
6753402	Tnnt2	0.47	0.602027515208236	-
6766649	Med23	0.47	0.602027515208236	-
6769197	Cirbp	0.47	0.602027515208236	-
6785347	C1qtnf1	0.47	0.602027515208236	-
6785774	Ddx56	0.47	0.602027515208236	-
6792424	Ush1g	0.47	0.602027515208236	-
6835428	Sybu	0.47	0.602027515208236	-
6835892	--	0.47	0.602027515208236	-
6868058	Slc15a3	0.47	0.602027515208236	-
6879567	Pamr1	0.47	0.602027515208236	-
6892723	Zhx3	0.47	0.602027515208236	-
6929797	Maea	0.47	0.602027515208236	-
6932469	Uso1	0.47	0.602027515208236	-
6936729	Fastk	0.47	0.602027515208236	-
6939604	Epha5	0.47	0.602027515208236	-
6958304	1700034J05Rik	0.47	0.602027515208236	-
6960351	Tead2	0.47	0.602027515208236	-
6963542	Parva	0.47	0.602027515208236	-
6755173	Dedd	0.471	0.602027515208236	-
6759729	Aamp	0.471	0.602027515208236	-
6790674	4932411E22Rik	0.471	0.602027515208236	-
6791151	Kpnb1	0.471	0.602027515208236	-
6813470	H2afy	0.471	0.602027515208236	-

6818203	Syt15	0.471	0.602027515208236	-
6838811	Marcksl1	0.471	0.602027515208236	-
6843923	Litaf	0.471	0.602027515208236	-
6852836	Epas1	0.471	0.602027515208236	-
6882429	Psp	0.471	0.602027515208236	-
6896860	Spry1	0.471	0.602027515208236	-
6966611	Pop4	0.471	0.602027515208236	-
6968426	Slco3a1	0.471	0.602027515208236	-
6978780	Ces2b	0.471	0.602027515208236	-
6980598	2410022L05Rik	0.471	0.602027515208236	-
6998429	Ccrl1	0.471	0.602027515208236	-
7006286	Vamp7	0.471	0.602027515208236	-
6763695	Blzf1	0.472	0.602027515208236	-
6764088	Itln1	0.472	0.602027515208236	-
6782105	Clec10a	0.472	0.602027515208236	-
6784218	Stat5a	0.472	0.602027515208236	-
6785762	Nudcd3	0.472	0.602027515208236	-
6837437	Arfgap3	0.472	0.602027515208236	-
6844444	Cyp2ab1	0.472	0.602027515208236	-
6847184	Hspa13	0.472	0.602027515208236	-
6861551	Onecut2	0.472	0.602027515208236	-
6899360	S100a14	0.472	0.602027515208236	-
6917046	Mtf1	0.472	0.602027515208236	-
6917816	Tcea3	0.472	0.602027515208236	-
6955432	Chchd6	0.472	0.602027515208236	-
6959323	Zfp60	0.472	0.602027515208236	+
6965024	Dpysl4	0.472	0.602027515208236	-
6968728	Pex11a	0.472	0.602027515208236	-
6972227	Chid1	0.472	0.602027515208236	-
6976237	Hpgd	0.472	0.602027515208236	-
6989229	Ireb2	0.472	0.602027515208236	-
6993834	Kank2	0.472	0.602027515208236	-
7011040	Bcorl1	0.472	0.602027515208236	-
7013856	Tmem35	0.472	0.602027515208236	-
6747837	Tmem70	0.473	0.602027515208236	-
6764089	Ly9	0.473	0.602027515208236	-
6764134	Casq1	0.473	0.602027515208236	-
6764657	Nvl	0.473	0.602027515208236	-
6784004	Pnmt	0.473	0.602027515208236	-
6785299	Syngr2	0.473	0.602027515208236	-
6791964	Polg2	0.473	0.602027515208236	-
6798269	Inf2	0.473	0.602027515208236	-
6804551	Gm5444	0.473	0.602027515208236	-
6812212	Serpinb6a	0.473	0.602027515208236	-
6813495	Il9	0.473	0.602027515208236	-
6824662	Rab2b	0.473	0.602027515208236	-
6831652	Maf1	0.473	0.602027515208236	-
6832324	Arhgap8	0.473	0.602027515208236	-
6845193	Muc20	0.473	0.602027515208236	-
6850019	Tap1	0.473	0.602027515208236	-

6854893	Zfp871	0.473	0.602027515208236	-
6856257	Rfx2	0.473	0.602027515208236	-
6869543	Gpr120	0.473	0.602027515208236	-
6871177	Mrpl49	0.473	0.602027515208236	-
6876028	Odf2	0.473	0.602027515208236	-
6885453	Nacc2	0.473	0.602027515208236	-
6903480	Armc1	0.473	0.602027515208236	-
6919173	Ssu72	0.473	0.602027515208236	-
6941639	1110008J03Rik	0.473	0.602027515208236	-
6949152	Slc6a11	0.473	0.602027515208236	-
6959612	2200002J24Rik	0.473	0.602027515208236	-
6749851	Ctla4	0.474	0.602027515208236	-
6751549	Fam132b	0.474	0.602027515208236	-
6755378	Kmo	0.474	0.602027515208236	-
6763972	Nuf2	0.474	0.602027515208236	-
6769131	Madcam1	0.474	0.602027515208236	-
6799836	Dld	0.474	0.602027515208236	-
6817417	Comtd1	0.474	0.602027515208236	-
6849572	Brpf3	0.474	0.602027515208236	-
6858503	Lyzl1	0.474	0.602027515208236	-
6880694	Slc28a2	0.474	0.602027515208236	-
6882256	2310046K01Rik	0.474	0.602027515208236	-
6885516	Tmem8c	0.474	0.602027515208236	-
6885532	Brd3	0.474	0.602027515208236	-
6892957	Acot8	0.474	0.602027515208236	-
6893918	Gm14288	0.474	0.602027515208236	-
6894272	Ptk6	0.474	0.602027515208236	-
6900052	Casq2	0.474	0.602027515208236	-
6901059	Prss12	0.474	0.602027515208236	-
6940658	Abcg3	0.474	0.602027515208236	-
6947383	Dqx1	0.474	0.602027515208236	-
6949694	Mfap5	0.474	0.602027515208236	-
6973561	Lilra6	0.474	0.602027515208236	-
6975966	Rwdd4a	0.474	0.602027515208236	-
6987288	Zfp317	0.474	0.602027515208236	-
6996308	Slc24a1	0.474	0.602027515208236	-
6769264	Zbtb7a	0.475	0.602027515208236	-
6771995	Ust	0.475	0.602027515208236	-
6779725	Rtn4	0.475	0.602027515208236	-
6783736	Tac4	0.475	0.602027515208236	-
6786668	Pex13	0.475	0.602027515208236	-
6791451	Stat3	0.475	0.602027515208236	+
6795078	Akap6	0.475	0.602027515208236	-
6803208	Ddx24	0.475	0.602027515208236	-
6845553	Adprh	0.475	0.602027515208236	-
6851669	Man2a1	0.475	0.602027515208236	-
6871161	Dpf2	0.475	0.602027515208236	-
6896431	Eif5a2	0.475	0.602027515208236	-
6907341	Bcl9	0.475	0.602027515208236	-
6912912	Ubap1	0.475	0.602027515208236	-

6921174	Serf2	0.475	0.602027515208236	-
6924951	4930538K18Rik	0.475	0.602027515208236	-
6925435	Gjb5	0.475	0.602027515208236	-
6934217	Psmd9	0.475	0.602027515208236	-
6946406	Aqp1	0.475	0.602027515208236	-
6951042	Rassf8	0.475	0.602027515208236	-
6977846	Phkb	0.475	0.602027515208236	-
6993465	Endod1	0.475	0.602027515208236	-
6996043	Arih1	0.475	0.602027515208236	-
7017610	Mecp2	0.475	0.602027515208236	-
6748695	Cnga3	0.476	0.602027515208236	-
6753439	Zfp281	0.476	0.602027515208236	+
6796807	Adck1	0.476	0.602027515208236	-
6824760	Cebpe	0.476	0.602027515208236	-
6825715	Npm2	0.476	0.602027515208236	-
6826265	1190002H23Rik	0.476	0.602027515208236	-
6828478	C9	0.476	0.602027515208236	-
6831869	Polr2f	0.476	0.602027515208236	-
6836699	Lynx1	0.476	0.602027515208236	-
6840705	Adcy5	0.476	0.602027515208236	-
6854868	Brd4	0.476	0.602027515208236	-
6873069	Aldh18a1	0.476	0.602027515208236	-
6873510	Obfc1	0.476	0.602027515208236	-
6880988	Astl	0.476	0.602027515208236	-
6892888	Kcns1	0.476	0.602027515208236	-
6901280	Pitx2	0.476	0.602027515208236	-
6914045	--	0.476	0.602027515208236	-
6915873	Tctex1d1	0.476	0.602027515208236	-
6917366	Pef1	0.476	0.602027515208236	-
6942377	Gtf2i	0.476	0.602027515208236	-
6949239	H1foo	0.476	0.602027515208236	-
6952293	Gcc1	0.476	0.602027515208236	-
6956581	Ghrl	0.476	0.602027515208236	-
6976214	Gpm6a	0.476	0.602027515208236	-
6977687	Ddx39	0.476	0.602027515208236	-
6981175	Bag4	0.476	0.602027515208236	-
7007402	Zfp71-rs1	0.476	0.602027515208236	-
6748534	Imp4	0.477	0.602052675819557	-
6784008	Grb7	0.477	0.602052675819557	-
6789540	Smtnl2	0.477	0.602052675819557	-
6810961	Chrm3	0.477	0.602052675819557	-
6818987	Vmn2r89	0.477	0.602052675819557	-
6857769	Haao	0.477	0.602052675819557	-
6874173	D19Ertd737e	0.477	0.602052675819557	-
6875376	Bmi1	0.477	0.602052675819557	-
6881975	Pax1	0.477	0.602052675819557	-
6885508	Rexo4	0.477	0.602052675819557	-
6911988	Mmp16	0.477	0.602052675819557	-
6924893	Mpl	0.477	0.602052675819557	-
6933917	Tbx5	0.477	0.602052675819557	-

6945550	Ubn2	0.477	0.602052675819557	-
6945830	Olfr446	0.477	0.602052675819557	-
6957134	Usp5	0.477	0.602052675819557	-
6966232	Zfp74	0.477	0.602052675819557	+
6971344	Dctpp1	0.477	0.602052675819557	-
6974752	Whsc1l1	0.477	0.602052675819557	-
6982091	F11	0.477	0.602052675819557	-
6989044	Alg9	0.477	0.602052675819557	-
7011378	Zfp449	0.477	0.602052675819557	-
6750589	Fam134a	0.478	0.602077733246342	-
6776782	Myf5	0.478	0.602077733246342	-
6783828	Hoxb13	0.478	0.602077733246342	-
6786473	Slc1a4	0.478	0.602077733246342	-
6789328	Senp3	0.478	0.602077733246342	-
6809082	Otp	0.478	0.602077733246342	-
6811997	Cdkal1	0.478	0.602077733246342	-
6813385	Rab24	0.478	0.602077733246342	-
6816018	Rab3c	0.478	0.602077733246342	-
6836849	Recql4	0.478	0.602077733246342	-
6854384	Gfer	0.478	0.602077733246342	-
6855820	9830107B12Rik	0.478	0.602077733246342	-
6876026	Cercam	0.478	0.602077733246342	-
6885463	Qsox2	0.478	0.602077733246342	-
6901458	Tbck	0.478	0.602077733246342	-
6905129	Nhlrc3	0.478	0.602077733246342	-
6918050	Tas1r2	0.478	0.602077733246342	-
6924871	Atp6v0b	0.478	0.602077733246342	-
6927001	Car6	0.478	0.602077733246342	-
6942551	Epo	0.478	0.602077733246342	-
6957421	Klrc2	0.478	0.602077733246342	-
6994431	Fli1	0.478	0.602077733246342	-
6762124	Il19	0.479	0.602270748046148	-
6769277	Apba3	0.479	0.602270748046148	-
6770745	Glipr1l2	0.479	0.602270748046148	-
6800906	Cfl2	0.479	0.602270748046148	-
6818497	Dydc1	0.479	0.602270748046148	-
6854260	Tnfrsf12a	0.479	0.602270748046148	-
6865229	Tmed7	0.479	0.602270748046148	-
6867852	Men1	0.479	0.602270748046148	-
6883607	2410001C21Rik	0.479	0.602270748046148	-
6900414	Aknad1	0.479	0.602270748046148	-
6901745	Adh4	0.479	0.602270748046148	-
6903088	Hey1	0.479	0.602270748046148	-
6926395	Padi3	0.479	0.602270748046148	-
6941675	Erp29	0.479	0.602270748046148	-
6942545	Trip6	0.479	0.602270748046148	-
6944951	Fscn3	0.479	0.602270748046148	-
6959447	Fbxo17	0.479	0.602270748046148	-
6960164	Ceacam18	0.479	0.602270748046148	-
6996297	Rab11a	0.479	0.602270748046148	-

6754680	Sele	0.48	0.602575011611705	-
6757932	Fam178b	0.48	0.602575011611705	-
6785663	Mtmr3	0.48	0.602575011611705	-
6801506	Pygl	0.48	0.602575011611705	-
6802557	Snw1	0.48	0.602575011611705	-
6810317	Dhx29	0.48	0.602575011611705	-
6837088	Micall1	0.48	0.602575011611705	-
6911089	Pklr	0.48	0.602575011611705	-
6918098	Rcc2	0.48	0.602575011611705	-
6918310	Pramef8	0.48	0.602575011611705	-
6927316	Tas1r3	0.48	0.602575011611705	-
6934164	P2rx4	0.48	0.602575011611705	-
6936723	Atg9b	0.48	0.602575011611705	-
6947617	C87436	0.48	0.602575011611705	-
6953906	Nt5c3	0.48	0.602575011611705	-
6958850	Zc3h4	0.48	0.602575011611705	-
6993872	Zfp599	0.48	0.602575011611705	+
6758984	Trak2	0.481	0.602822405638505	-
6792452	Gga3	0.481	0.602822405638505	-
6816248	Itga1	0.481	0.602822405638505	-
6850552	Enpp5	0.481	0.602822405638505	-
6860129	Wdr55	0.481	0.602822405638505	-
6886114	Zbtb26	0.481	0.602822405638505	+
6908935	Mettl14	0.481	0.602822405638505	-
6913020	Npr2	0.481	0.602822405638505	-
6929609	1700001C02Rik	0.481	0.602822405638505	-
6938770	9130230L23Rik	0.481	0.602822405638505	-
6940930	Gak	0.481	0.602822405638505	-
6962580	Prcp	0.481	0.602822405638505	-
6966314	Ffar2	0.481	0.602822405638505	-
6981099	Ido1	0.481	0.602822405638505	-
6981924	Asah1	0.481	0.602822405638505	-
6998646	Sema3f	0.481	0.602822405638505	-
7011377	Ddx26b	0.481	0.602822405638505	-
7018388	Ophn1	0.481	0.602822405638505	-
6757975	Mgat4a	0.482	0.602845719574271	-
6769304	Aes	0.482	0.602845719574271	-
6785603	Rnf185	0.482	0.602845719574271	-
6791467	Fam134c	0.482	0.602845719574271	-
6801451	1110034A24Rik	0.482	0.602845719574271	-
6802023	Atp6v1d	0.482	0.602845719574271	-
6819328	Zmym2	0.482	0.602845719574271	-
6829661	BC030476	0.482	0.602845719574271	-
6848556	Gm9992	0.482	0.602845719574271	-
6854447	Rhot2	0.482	0.602845719574271	-
6871121	Snx32	0.482	0.602845719574271	-
6881164	Tgm6	0.482	0.602845719574271	-
6891466	Esf1	0.482	0.602845719574271	-
6908493	Rtcd1	0.482	0.602845719574271	-
6909774	Gm4861	0.482	0.602845719574271	-

6913902	E130308A19Rik	0.482	0.602845719574271	-
6933628	Hnf1a	0.482	0.602845719574271	-
6934969	Upk3b	0.482	0.602845719574271	-
6939695	Ugt2b34	0.482	0.602845719574271	-
6942575	Mepce	0.482	0.602845719574271	-
6964250	Fam57b	0.482	0.602845719574271	-
6985088	Terf2	0.482	0.602845719574271	-
6760385	Pde6d	0.483	0.603258964879852	-
6764693	Lbr	0.483	0.603258964879852	-
6784553	Mettl2	0.483	0.603258964879852	-
6791387	Krt34	0.483	0.603258964879852	-
6811903	Prl8a9	0.483	0.603258964879852	-
6836848	Foxh1	0.483	0.603258964879852	-
6856202	Plin5	0.483	0.603258964879852	-
6871519	Ccdc86	0.483	0.603258964879852	-
6878978	Nup160	0.483	0.603258964879852	-
6898772	Fga	0.483	0.603258964879852	-
6934568	Piwil1	0.483	0.603258964879852	-
6942523	Ap1s1	0.483	0.603258964879852	-
6942573	6430598A04Rik	0.483	0.603258964879852	-
6951304	Bet1	0.483	0.603258964879852	-
6996649	Rnf111	0.483	0.603258964879852	-
6761825	Dpp10	0.484	0.603581361082973	-
6810280	Slc38a9	0.484	0.603581361082973	-
6834013	Tars	0.484	0.603581361082973	-
6882581	Cep250	0.484	0.603581361082973	-
6885336	Pax8	0.484	0.603581361082973	-
6890617	Cops2	0.484	0.603581361082973	-
6902543	Pigk	0.484	0.603581361082973	-
6913045	Clta	0.484	0.603581361082973	-
6916488	Pdzk1ip1	0.484	0.603581361082973	-
6916802	Slfnl1	0.484	0.603581361082973	-
6921068	Dctn3	0.484	0.603581361082973	-
6946778	Mad2l1	0.484	0.603581361082973	-
6971687	Nkx1-2	0.484	0.603581361082973	-
6971688	Fam53b	0.484	0.603581361082973	+
6978368	AA960436	0.484	0.603581361082973	-
7017134	Fgf13	0.484	0.603581361082973	-
6766333	Olig3	0.485	0.603581361082973	-
6772815	Arg1	0.485	0.603581361082973	-
6785321	Pgs1	0.485	0.603581361082973	-
6785808	Tbrg4	0.485	0.603581361082973	-
6813668	1700013B16Rik	0.485	0.603581361082973	-
6824556	A930018M24Rik	0.485	0.603581361082973	-
6838637	Krt86	0.485	0.603581361082973	-
6854320	Pdpk1	0.485	0.603581361082973	-
6861314	Gm4841	0.485	0.603581361082973	-
6890382	Ppip5k1	0.485	0.603581361082973	-
6899685	Golph3l	0.485	0.603581361082973	-
6939130	Ociad2	0.485	0.603581361082973	-

6946954	St3gal5	0.485	0.603581361082973	-
6953369	Zfp746	0.485	0.603581361082973	-
6964600	Gpr26	0.485	0.603581361082973	-
6966137	Timm50	0.485	0.603581361082973	-
6966343	Fxyd3	0.485	0.603581361082973	-
6971335	Tbc1d10b	0.485	0.603581361082973	-
6984955	4931428F04Rik	0.485	0.603581361082973	-
6986565	Cwf19l2	0.485	0.603581361082973	-
6987378	Ilf3	0.485	0.603581361082973	-
6990281	Sltm	0.485	0.603581361082973	-
6993900	rp9	0.485	0.603581361082973	-
6748837	Npas2	0.486	0.603663622389005	-
6764175	Slamf8	0.486	0.603663622389005	-
6775758	Hsp90b1	0.486	0.603663622389005	-
6777297	Frs2	0.486	0.603663622389005	-
6780986	Kif3a	0.486	0.603663622389005	-
6781498	Tnfrsf13b	0.486	0.603663622389005	-
6807251	Tmed9	0.486	0.603663622389005	-
6833600	AW549877	0.486	0.603663622389005	-
6837925	Kif21a	0.486	0.603663622389005	-
6849797	Cryaa	0.486	0.603663622389005	-
6873466	Nt5c2	0.486	0.603663622389005	-
6879695	0610012H03Rik	0.486	0.603663622389005	-
6888619	Olfr1232	0.486	0.603663622389005	-
6934670	Caln1	0.486	0.603663622389005	-
6942712	Ftsj2	0.486	0.603663622389005	-
6949847	Chd4	0.486	0.603663622389005	-
6957330	Tulp3	0.486	0.603663622389005	-
6968453	Sv2b	0.486	0.603663622389005	-
6984958	Exoc3l	0.486	0.603663622389005	-
6995504	Bco2	0.486	0.603663622389005	-
6769280	Pip5k1c	0.487	0.603663622389005	-
6806618	Nol7	0.487	0.603663622389005	-
6807259	Ddx46	0.487	0.603663622389005	-
6815555	Cenph	0.487	0.603663622389005	-
6819154	Lrp10	0.487	0.603663622389005	-
6828547	Gdnf	0.487	0.603663622389005	-
6836852	C030006K11Rik	0.487	0.603663622389005	-
6862816	Neto1	0.487	0.603663622389005	+
6885796	Ptges	0.487	0.603663622389005	-
6891065	Slc23a2	0.487	0.603663622389005	-
6892033	Vsx1	0.487	0.603663622389005	-
6896593	Mfn1	0.487	0.603663622389005	-
6903099	Tpd52	0.487	0.603663622389005	-
6903360	Sirpb1b	0.487	0.603663622389005	-
6921379	Trim14	0.487	0.603663622389005	-
6935765	Pds5b	0.487	0.603663622389005	-
6958910	Strn4	0.487	0.603663622389005	-
6989901	Dennd4a	0.487	0.603663622389005	-
6992363	Slc25a20	0.487	0.603663622389005	-

6993501	Fat3	0.487	0.603663622389005	-
6994157	Jam3	0.487	0.603663622389005	-
7017598	Pdzd4	0.487	0.603663622389005	-
6751070	Mff	0.488	0.603663622389005	-
6768898	Vpreb3	0.488	0.603663622389005	-
6787122	Efcab9	0.488	0.603663622389005	-
6823849	Mapk8	0.488	0.603663622389005	-
6837022	C1qtnf6	0.488	0.603663622389005	-
6838754	Calcoco1	0.488	0.603663622389005	-
6867780	Map3k11	0.488	0.603663622389005	-
6872916	Ide	0.488	0.603663622389005	-
6873441	Cyp17a1	0.488	0.603663622389005	-
6883119	Zswim1	0.488	0.603663622389005	-
6885501	Surf6	0.488	0.603663622389005	-
6887196	Ifih1	0.488	0.603663622389005	-
6938999	Gabrg1	0.488	0.603663622389005	-
6960370	Kcna7	0.488	0.603663622389005	-
6966903	Atf5	0.488	0.603663622389005	-
6979224	Cntnap4	0.488	0.603663622389005	-
6998919	Ccrl2	0.488	0.603663622389005	-
6753083	Slc41a1	0.489	0.603663622389005	-
6755247	Vsig8	0.489	0.603663622389005	-
6792019	Cacng1	0.489	0.603663622389005	-
6803216	Serpina10	0.489	0.603663622389005	-
6803274	Serpina12	0.489	0.603663622389005	-
6806959	Omd	0.489	0.603663622389005	-
6815058	Thbs4	0.489	0.603663622389005	-
6817700	Arhgef3	0.489	0.603663622389005	+
6823717	Dnahc1	0.489	0.603663622389005	-
6825439	Scara3	0.489	0.603663622389005	-
6835579	Trps1	0.489	0.603663622389005	-
6843216	Kcne2	0.489	0.603663622389005	-
6853960	Rgmb	0.489	0.603663622389005	-
6868110	Ms4a4d	0.489	0.603663622389005	-
6912945	Galt	0.489	0.603663622389005	-
6915739	Angptl3	0.489	0.603663622389005	-
6917794	Gale	0.489	0.603663622389005	-
6952284	Grm8	0.489	0.603663622389005	-
6955764	Fam19a4	0.489	0.603663622389005	-
6995090	Upk2	0.489	0.603663622389005	-
6998396	Srprb	0.489	0.603663622389005	-
7018328	Vsig4	0.489	0.603663622389005	-
6753594	F13b	0.49	0.603663622389005	-
6755189	Cd244	0.49	0.603663622389005	-
6818556	Psmc6	0.49	0.603663622389005	-
6831932	Gtpbp1	0.49	0.603663622389005	-
6840532	Wdr53	0.49	0.603663622389005	-
6848201	Fam3b	0.49	0.603663622389005	-
6854401	Hn1l	0.49	0.603663622389005	-
6875173	Stam	0.49	0.603663622389005	-

6875964	Gbgt1	0.49	0.603663622389005	-
6875969	Tsc1	0.49	0.603663622389005	-
6876943	Kif5c	0.49	0.603663622389005	-
6890436	Duoxa1	0.49	0.603663622389005	-
6892964	Pltp	0.49	0.603663622389005	-
6894304	Samd10	0.49	0.603663622389005	-
6901413	Papss1	0.49	0.603663622389005	-
6910727	Lhx8	0.49	0.603663622389005	-
6922063	Rod1	0.49	0.603663622389005	-
6941762	Anapc5	0.49	0.603663622389005	-
6958981	Sympk	0.49	0.603663622389005	-
6969788	Pold3	0.49	0.603663622389005	-
6971843	Clrn3	0.49	0.603663622389005	-
6972260	Dusp8	0.49	0.603663622389005	-
6984365	Ces1e	0.49	0.603663622389005	-
6986649	Casp4	0.49	0.603663622389005	-
6992380	Pfkfb4	0.49	0.603663622389005	-
6751546	Klhl30	0.491	0.603663622389005	-
6764705	Degs1	0.491	0.603663622389005	-
6767463	Ostm1	0.491	0.603663622389005	-
6792554	Srsf2	0.491	0.603663622389005	-
6796532	Rbm25	0.491	0.603663622389005	-
6824738	Prmt5	0.491	0.603663622389005	-
6835856	Has2	0.491	0.603663622389005	-
6867650	Ppp1ca	0.491	0.603663622389005	-
6877398	Upp2	0.491	0.603663622389005	-
6877954	Myo3b	0.491	0.603663622389005	-
6892879	Tomm34	0.491	0.603663622389005	-
6893556	Zbp1	0.491	0.603663622389005	-
6900644	Dph5	0.491	0.603663622389005	-
6916748	Slc2a1	0.491	0.603663622389005	+
6941193	Alkbh2	0.491	0.603663622389005	-
6964557	Acadsb	0.491	0.603663622389005	-
6993055	Snrk	0.491	0.603663622389005	-
7014637	Klf8	0.491	0.603663622389005	-
6756541	Cd34	0.492	0.603663622389005	-
6782777	Rab11fip4	0.492	0.603663622389005	-
6784279	Nbr1	0.492	0.603663622389005	-
6792994	Pfn4	0.492	0.603663622389005	-
6813560	Gkap1	0.492	0.603663622389005	-
6824841	Dhrs1	0.492	0.603663622389005	-
6836948	Apol7a	0.492	0.603663622389005	-
6843951	Rsl1d1	0.492	0.603663622389005	-
6845911	Zdhhc23	0.492	0.603663622389005	-
6851095	Rab5a	0.492	0.603663622389005	-
6857963	Pigf	0.492	0.603663622389005	-
6859809	Map3k2	0.492	0.603663622389005	-
6870497	Shoc2	0.492	0.603663622389005	-
6871144	Scyl1	0.492	0.603663622389005	-
6875747	Lcn13	0.492	0.603663622389005	-

6876159	Lamc3	0.492	0.603663622389005	-
6888299	Tfpi	0.492	0.603663622389005	-
6910708	Rabggtb	0.492	0.603663622389005	-
6932140	Ythdc1	0.492	0.603663622389005	-
6972192	Irf7	0.492	0.603663622389005	-
6973156	Clptm1	0.492	0.603663622389005	-
6977970	Tmem188	0.492	0.603663622389005	-
6979772	Galnt2	0.492	0.603663622389005	-
6985030	Slc7a6os	0.492	0.603663622389005	-
6990922	Sh3bgrl2	0.492	0.603663622389005	-
6996432	Aph1b	0.492	0.603663622389005	-
7011035	Sash3	0.492	0.603663622389005	-
7017930	4930595M18Rik	0.492	0.603663622389005	-
7019669	Esx1	0.492	0.603663622389005	-
6752425	Tcfcp2l1	0.493	0.603663622389005	-
6757129	Tceb1	0.493	0.603663622389005	-
6780882	Cdkn2aipnl	0.493	0.603663622389005	-
6787230	Foxi1	0.493	0.603663622389005	-
6819147	Oxa1l	0.493	0.603663622389005	-
6829910	Odf1	0.493	0.603663622389005	-
6831709	1110038F14Rik	0.493	0.603663622389005	-
6833382	Krt18	0.493	0.603663622389005	-
6843675	Sep 12	0.493	0.603663622389005	-
6863755	Dsc3	0.493	0.603663622389005	-
6865980	Ppargc1b	0.493	0.603663622389005	-
6870166	Slk	0.493	0.603663622389005	-
6885412	BC029214	0.493	0.603663622389005	-
6912524	Srsf13b	0.493	0.603663622389005	-
6913195	Tmod1	0.493	0.603663622389005	-
6921988	Txndc8	0.493	0.603663622389005	-
6931261	Lias	0.493	0.603663622389005	-
6955226	Gmcl1	0.493	0.603663622389005	-
6964058	Rbbp6	0.493	0.603663622389005	-
6969832	Relt	0.493	0.603663622389005	-
6975919	Irf2	0.493	0.603663622389005	-
6980072	Xab2	0.493	0.603663622389005	-
7015390	Usp27x	0.493	0.603663622389005	-
6764033	Dusp12	0.494	0.603663622389005	-
6764352	Opn3	0.494	0.603663622389005	-
6769154	Prtn3	0.494	0.603663622389005	-
6769928	Tmcc3	0.494	0.603663622389005	+
6781472	Dhrs7b	0.494	0.603663622389005	-
6783363	Cuedc1	0.494	0.603663622389005	-
6790621	Msi2	0.494	0.603663622389005	-
6801395	Mdga2	0.494	0.603663622389005	-
6802040	Vti1b	0.494	0.603663622389005	-
6812637	Gcm2	0.494	0.603663622389005	-
6842926	N6amt1	0.494	0.603663622389005	-
6847880	Gart	0.494	0.603663622389005	-
6871520	Ms4a10	0.494	0.603663622389005	-

6889314	Fbxo3	0.494	0.603663622389005	-
6911575	Clvs1	0.494	0.603663622389005	-
6925507	Hmgb4	0.494	0.603663622389005	-
6926976	Slc25a33	0.494	0.603663622389005	-
6944385	St7	0.494	0.603663622389005	-
6952097	Fam3c	0.494	0.603663622389005	-
6968018	Mef2a	0.494	0.603663622389005	-
6984214	Aktip	0.494	0.603663622389005	-
6998407	Tmem108	0.494	0.603663622389005	-
6999631	1110059G10Rik	0.494	0.603663622389005	-
7018225	Pdk3	0.494	0.603663622389005	-
6763295	Ralgps2	0.495	0.603957204767064	-
6767464	Sec63	0.495	0.603957204767064	-
6779162	Spred2	0.495	0.603957204767064	+
6783842	Hoxb6	0.495	0.603957204767064	-
6784202	Ttc25	0.495	0.603957204767064	-
6798309	Pacs2	0.495	0.603957204767064	-
6841849	Dcbld2	0.495	0.603957204767064	-
6855003	Slc39a7	0.495	0.603957204767064	-
6865112	Spink3	0.495	0.603957204767064	-
6869177	A1cf	0.495	0.603957204767064	-
6884294	Tcea2	0.495	0.603957204767064	-
6892576	Tti1	0.495	0.603957204767064	-
6902172	Bcl10	0.495	0.603957204767064	-
6929312	Nub1	0.495	0.603957204767064	-
6936744	Gbx1	0.495	0.603957204767064	-
6992887	Exog	0.495	0.603957204767064	-
7011050	Rab33a	0.495	0.603957204767064	+
6763389	Fam5b	0.496	0.604304363505229	-
6775392	Ap3d1	0.496	0.604304363505229	-
6784236	Atp6v0a1	0.496	0.604304363505229	-
6796564	Acot4	0.496	0.604304363505229	-
6817989	Capn7	0.496	0.604304363505229	-
6824610	Ndrg2	0.496	0.604304363505229	-
6835640	Rad21	0.496	0.604304363505229	-
6837373	Ndufa6	0.496	0.604304363505229	-
6860411	Sh3rf2	0.496	0.604304363505229	-
6871240	Slc22a12	0.496	0.604304363505229	-
6895537	Fam164a	0.496	0.604304363505229	-
6899805	Acp6	0.496	0.604304363505229	-
6957150	Cops7a	0.496	0.604304363505229	-
6977669	Ndufb7	0.496	0.604304363505229	-
6988220	Esam	0.496	0.604304363505229	-
7018297	Zc4h2	0.496	0.604304363505229	-
6748646	Cnnm3	0.497	0.604541674167417	-
6751640	Dusp28	0.497	0.604541674167417	-
6785139	Armc7	0.497	0.604541674167417	-
6791436	Zfp385c	0.497	0.604541674167417	-
6806896	Barx1	0.497	0.604541674167417	-
6828420	Prkaa1	0.497	0.604541674167417	-

6839380	Snn	0.497	0.604541674167417	-
6849974	Cd320	0.497	0.604541674167417	-
6850080	Neu1	0.497	0.604541674167417	-
6856269	Gtf2f1	0.497	0.604541674167417	-
6866747	Gm672	0.497	0.604541674167417	-
6916943	Bmp8b	0.497	0.604541674167417	-
6918021	Pla2g2e	0.497	0.604541674167417	-
6940565	Slc10a6	0.497	0.604541674167417	-
6952926	Parp12	0.497	0.604541674167417	-
6965896	Rabac1	0.497	0.604541674167417	-
6984922	Nae1	0.497	0.604541674167417	-
6987128	2200002K05Rik	0.497	0.604541674167417	-
6760364	Ncl	0.498	0.604560905497664	-
6766443	1700020N01Rik	0.498	0.604560905497664	-
6773368	1700025K23Rik	0.498	0.604560905497664	-
6784494	Crhr1	0.498	0.604560905497664	-
6808537	Lysmd3	0.498	0.604560905497664	-
6810697	Akr1c18	0.498	0.604560905497664	-
6833481	Hoxc4	0.498	0.604560905497664	-
6834063	6030458C11Rik	0.498	0.604560905497664	-
6837614	Cerk	0.498	0.604560905497664	-
6849612	BC004004	0.498	0.604560905497664	-
6861547	St8sia3	0.498	0.604560905497664	-
6867193	Cndp1	0.498	0.604560905497664	-
6879006	Celf1	0.498	0.604560905497664	-
6899628	Pi4kb	0.498	0.604560905497664	-
6899867	Reg4	0.498	0.604560905497664	-
6912040	Ttpa	0.498	0.604560905497664	-
6942737	Ttyh3	0.498	0.604560905497664	-
6947034	Suclg1	0.498	0.604560905497664	-
6966304	Haus5	0.498	0.604560905497664	-
6968875	Bnc1	0.498	0.604560905497664	-
6969030	Zfand6	0.498	0.604560905497664	-
6978935	Cirh1a	0.498	0.604560905497664	-
6781525	Grap	0.499	0.604599141016464	-
6784317	G6pc3	0.499	0.604599141016464	-
6796632	Isca2	0.499	0.604599141016464	-
6801807	Kcnh5	0.499	0.604599141016464	-
6804405	Grhl1	0.499	0.604599141016464	-
6834108	Cdh6	0.499	0.604599141016464	-
6838171	Nell2	0.499	0.604599141016464	-
6851927	Dlgap1	0.499	0.604599141016464	-
6866839	Haus1	0.499	0.604599141016464	-
6872051	Gda	0.499	0.604599141016464	-
6885483	Card9	0.499	0.604599141016464	-
6897836	C130079G13Rik	0.499	0.604599141016464	-
6901196	Tifa	0.499	0.604599141016464	-
6926202	Cela3b	0.499	0.604599141016464	-
6927124	Espn	0.499	0.604599141016464	-
6936758	Crygn	0.499	0.604599141016464	-

6956937	B4galnt3	0.499	0.604599141016464	-
6989945	Pif1	0.499	0.604599141016464	-
6751082	Agfg1	0.5	0.604599141016464	-
6765325	Hsd11b1	0.5	0.604599141016464	-
6771602	Rdh19	0.5	0.604599141016464	-
6784396	Nmt1	0.5	0.604599141016464	-
6788399	Slc36a3	0.5	0.604599141016464	-
6795455	Ctage5	0.5	0.604599141016464	-
6796561	Acot2	0.5	0.604599141016464	-
6831567	Ly6f	0.5	0.604599141016464	-
6847702	Krtap14	0.5	0.604599141016464	-
6850841	AI314976	0.5	0.604599141016464	-
6885969	Pbx3	0.5	0.604599141016464	-
6891210	Hao1	0.5	0.604599141016464	-
6892307	E2f1	0.5	0.604599141016464	-
6940201	Paqr3	0.5	0.604599141016464	-
6942939	Aimp2	0.5	0.604599141016464	-
6946028	Gimap4	0.5	0.604599141016464	-
6948107	Adamts9	0.5	0.604599141016464	-
6955377	Podxl2	0.5	0.604599141016464	-
6958944	Ceacam11	0.5	0.604599141016464	-
6967211	E2f8	0.5	0.604599141016464	-
6971996	Stk32c	0.5	0.604599141016464	-
6972181	Hras1	0.5	0.604599141016464	-
6977084	Fam125a	0.5	0.604599141016464	-
6983265	Bst2	0.5	0.604599141016464	-
6984526	Gtl3	0.5	0.604599141016464	-
7012768	Yipf6	0.5	0.604599141016464	-
6759905	Epha4	0.501	0.604942280200143	-
6787809	Dppa1	0.501	0.604942280200143	-
6796777	Gstz1	0.501	0.604942280200143	-
6811073	Edaradd	0.501	0.604942280200143	-
6819200	Pabpn1	0.501	0.604942280200143	-
6820081	Phyhip	0.501	0.604942280200143	-
6876937	Epc2	0.501	0.604942280200143	-
6880087	Fmn1	0.501	0.604942280200143	-
6894152	Psma7	0.501	0.604942280200143	-
6904297	Anxa5	0.501	0.604942280200143	-
6926419	D4Ertd22e	0.501	0.604942280200143	-
6959127	Cadm4	0.501	0.604942280200143	-
6962880	Arrb1	0.501	0.604942280200143	-
6963049	Rrm1	0.501	0.604942280200143	-
6964252	Doc2a	0.501	0.604942280200143	-
6968744	Ap3s2	0.501	0.604942280200143	-
6753245	Chit1	0.502	0.605446497099509	-
6792650	Ddc8	0.502	0.605446497099509	-
6817978	Tnnc1	0.502	0.605446497099509	-
6824820	Nrl	0.502	0.605446497099509	-
6850540	Rcan2	0.502	0.605446497099509	-
6854790	Tff1	0.502	0.605446497099509	-

6873271	Scd1	0.502	0.605446497099509	-
6910622	Fam73a	0.502	0.605446497099509	-
6929784	Slc5a1	0.502	0.605446497099509	-
6931413	Gm5108	0.502	0.605446497099509	-
6938631	Rell1	0.502	0.605446497099509	-
6980394	Gm6524	0.502	0.605446497099509	-
6995059	Cbl	0.502	0.605446497099509	-
6782093	Dlg4	0.503	0.605841532976827	-
6782214	Fam64a	0.503	0.605841532976827	-
6792820	Dus1l	0.503	0.605841532976827	-
6808680	Ccnh	0.503	0.605841532976827	-
6857876	Six2	0.503	0.605841532976827	-
6935991	Steap1	0.503	0.605841532976827	-
6959474	Rasgrp4	0.503	0.605841532976827	-
6960309	Pnkp	0.503	0.605841532976827	-
6965702	Psg18	0.503	0.605841532976827	-
6971264	Cd19	0.503	0.605841532976827	-
6972592	Exoc3l2	0.503	0.605841532976827	-
6983922	Vps35	0.503	0.605841532976827	-
6998706	Wdr6	0.503	0.605841532976827	+
6999622	Zfp445	0.503	0.605841532976827	-
7013412	Ube2dnl	0.503	0.605841532976827	-
6758646	Inpp1	0.504	0.60601432867504	-
6781982	Vamp2	0.504	0.60601432867504	-
6791444	Stat5b	0.504	0.60601432867504	-
6832079	Adsl	0.504	0.60601432867504	-
6857212	Dpy30	0.504	0.60601432867504	-
6859284	Dsg3	0.504	0.60601432867504	-
6869981	Kazald1	0.504	0.60601432867504	-
6891998	3300002I08Rik	0.504	0.60601432867504	-
6918047	Iffo2	0.504	0.60601432867504	-
6933065	Ibsp	0.504	0.60601432867504	-
6933328	Tmem175	0.504	0.60601432867504	+
6933662	Srsf9	0.504	0.60601432867504	-
6956981	Bid	0.504	0.60601432867504	-
6959507	4930432E11Rik	0.504	0.60601432867504	-
6960824	Luzp2	0.504	0.60601432867504	-
6983249	Nr2f6	0.504	0.60601432867504	-
6985288	Marveld3	0.504	0.60601432867504	-
6990662	Klhl31	0.504	0.60601432867504	-
7012862	Nlgn3	0.504	0.60601432867504	-
6753268	Myog	0.505	0.60601432867504	-
6764133	Pea15a	0.505	0.60601432867504	-
6764211	Cadm3	0.505	0.60601432867504	-
6772410	Gm4922	0.505	0.60601432867504	-
6779845	Ubtd2	0.505	0.60601432867504	+
6782552	Tmigd1	0.505	0.60601432867504	-
6788815	Aldh3a2	0.505	0.60601432867504	-
6800468	Stxbp6	0.505	0.60601432867504	+
6807467	Rmi1	0.505	0.60601432867504	-

6808544	Cetn3	0.505	0.60601432867504	-
6813327	Cltb	0.505	0.60601432867504	+
6843338	Cbr3	0.505	0.60601432867504	-
6849214	Ccdc64b	0.505	0.60601432867504	-
6849992	Wdr46	0.505	0.60601432867504	-
6863540	Zfp521	0.505	0.60601432867504	-
6901551	Cxxc4	0.505	0.60601432867504	-
6941261	Mlec	0.505	0.60601432867504	-
6945555	Luc7l2	0.505	0.60601432867504	-
6955131	Spr	0.505	0.60601432867504	-
6961110	Apba2	0.505	0.60601432867504	-
6973064	Galp	0.505	0.60601432867504	-
6973201	Shisa7	0.505	0.60601432867504	-
6760292	Dner	0.506	0.60601432867504	-
6760614	Gbx2	0.506	0.60601432867504	-
6770201	Kitl	0.506	0.60601432867504	+
6773018	Rnf146	0.506	0.60601432867504	-
6808892	Acot12	0.506	0.60601432867504	-
6810066	Depdc1b	0.506	0.60601432867504	-
6818950	Apex1	0.506	0.60601432867504	+
6829964	Dcaf13	0.506	0.60601432867504	-
6871270	Fkbp2	0.506	0.60601432867504	-
6872704	Atad1	0.506	0.60601432867504	-
6901187	4930422G04Rik	0.506	0.60601432867504	-
6913270	Tgfbr1	0.506	0.60601432867504	-
6947679	Gfpt1	0.506	0.60601432867504	-
6954981	Pole4	0.506	0.60601432867504	-
6959159	Dmrtc2	0.506	0.60601432867504	-
6959595	Hspb6	0.506	0.60601432867504	-
6974621	Slc20a2	0.506	0.60601432867504	-
6990564	Lysmd2	0.506	0.60601432867504	-
6998640	Gnai2	0.506	0.60601432867504	-
6750007	Creb1	0.507	0.60601432867504	-
6753409	Tmem9	0.507	0.60601432867504	-
6756572	Xkr4	0.507	0.60601432867504	-
6770923	5330438D12Rik	0.507	0.60601432867504	-
6780527	Adam19	0.507	0.60601432867504	-
6783040	Gm11428	0.507	0.60601432867504	-
6798479	Sp8	0.507	0.60601432867504	-
6803577	Degs2	0.507	0.60601432867504	-
6813456	Pitx1	0.507	0.60601432867504	-
6823696	Gnl3	0.507	0.60601432867504	-
6836981	Foxred2	0.507	0.60601432867504	-
6858871	Cables1	0.507	0.60601432867504	-
6868356	Gnaq	0.507	0.60601432867504	-
6900768	4833424O15Rik	0.507	0.60601432867504	-
6916938	Trit1	0.507	0.60601432867504	-
6926440	Clcnka	0.507	0.60601432867504	-
6936140	Grm3	0.507	0.60601432867504	-
6939717	Ugt2b38	0.507	0.60601432867504	-

6947737	Copg	0.507	0.60601432867504	-
6953763	Fkbp14	0.507	0.60601432867504	-
6963548	Tead1	0.507	0.60601432867504	-
6966229	BC027344	0.507	0.60601432867504	-
6970066	Trim30d	0.507	0.60601432867504	-
6972149	BC066028	0.507	0.60601432867504	-
6992925	Mobp	0.507	0.60601432867504	-
7009800	Pcsk1n	0.507	0.60601432867504	-
6769212	Reep6	0.508	0.606405087889762	-
6805450	BC005537	0.508	0.606405087889762	-
6811140	Mrpl32	0.508	0.606405087889762	-
6832571	Adm2	0.508	0.606405087889762	-
6837835	Rabl2	0.508	0.606405087889762	-
6893139	Ptgis	0.508	0.606405087889762	-
6911337	Lyn	0.508	0.606405087889762	-
6913560	Slc44a1	0.508	0.606405087889762	-
6927278	Mib2	0.508	0.606405087889762	-
6939005	Gabra2	0.508	0.606405087889762	-
6941667	Ptpn11	0.508	0.606405087889762	-
6965262	Syt8	0.508	0.606405087889762	-
6974126	F7	0.508	0.606405087889762	-
6974851	Chrnb3	0.508	0.606405087889762	-
6975701	Pcm1	0.508	0.606405087889762	-
6763754	Tiprl	0.509	0.606542286368037	-
6791881	Limd2	0.509	0.606542286368037	+
6794757	Pnpla8	0.509	0.606542286368037	-
6802505	Ngb	0.509	0.606542286368037	-
6818249	Grid1	0.509	0.606542286368037	-
6831798	Ncf4	0.509	0.606542286368037	-
6838390	Ddx23	0.509	0.606542286368037	-
6839769	Klhl22	0.509	0.606542286368037	-
6883320	Rnf114	0.509	0.606542286368037	-
6884473	5430407P10Rik	0.509	0.606542286368037	-
6899574	Lingo4	0.509	0.606542286368037	-
6941217	Git2	0.509	0.606542286368037	-
6945217	--	0.509	0.606542286368037	-
6966215	Catsperg2	0.509	0.606542286368037	-
6978818	Hsf4	0.509	0.606542286368037	-
6985032	Smpd3	0.509	0.606542286368037	-
7011893	Cnga2	0.509	0.606542286368037	-
6747839	Ly96	0.51	0.606542286368037	-
6756394	Nek2	0.51	0.606542286368037	-
6756790	Mybl1	0.51	0.606542286368037	-
6769309	Ankrd24	0.51	0.606542286368037	-
6778376	Tbc1d10a	0.51	0.606542286368037	-
6791532	Mpp2	0.51	0.606542286368037	-
6803038	Foxn3	0.51	0.606542286368037	-
6804696	Larp4b	0.51	0.606542286368037	-
6828374	Oxct1	0.51	0.606542286368037	-
6844862	Uts2d	0.51	0.606542286368037	-

6848584	T2	0.51	0.606542286368037	-
6852751	Dync2li1	0.51	0.606542286368037	-
6885616	1700007K13Rik	0.51	0.606542286368037	-
6918036	Capzb	0.51	0.606542286368037	-
6921129	Pigo	0.51	0.606542286368037	-
6922382	Astn2	0.51	0.606542286368037	-
6929883	Hmx1	0.51	0.606542286368037	-
6934323	Atp6v0a2	0.51	0.606542286368037	-
6937227	Spon2	0.51	0.606542286368037	-
6941856	Vps37b	0.51	0.606542286368037	+
6947382	Aup1	0.51	0.606542286368037	-
6947400	Mobkl1b	0.51	0.606542286368037	-
6983331	Slc35e1	0.51	0.606542286368037	-
6988771	Apoa4	0.51	0.606542286368037	-
7016722	Zfp280c	0.51	0.606542286368037	-
6789279	Tmem88	0.511	0.607250615546957	-
6813748	Ctsj	0.511	0.607250615546957	-
6840860	Tmem39a	0.511	0.607250615546957	-
6890422	Spg11	0.511	0.607250615546957	-
6925380	Tcfap2e	0.511	0.607250615546957	-
6970139	Arfip2	0.511	0.607250615546957	-
6972949	Trappc6a	0.511	0.607250615546957	-
6977712	Podnl1	0.511	0.607250615546957	-
6989038	Cryab	0.511	0.607250615546957	-
6753338	Arl8a	0.512	0.607584123638918	-
6817966	Pbrm1	0.512	0.607584123638918	-
6848179	Dscam	0.512	0.607584123638918	-
6849361	Prss34	0.512	0.607584123638918	-
6850271	Gabbr1	0.512	0.607584123638918	-
6860507	Scgb3a2	0.512	0.607584123638918	-
6861321	Dctn4	0.512	0.607584123638918	-
6883601	Cstf1	0.512	0.607584123638918	-
6916947	Hpcal4	0.512	0.607584123638918	+
6946802	Serbp1	0.512	0.607584123638918	-
6966274	Lrfn3	0.512	0.607584123638918	-
6978808	Ces4a	0.512	0.607584123638918	-
6983141	Ndufa13	0.512	0.607584123638918	-
6993735	Raver1	0.512	0.607584123638918	-
6995762	Acsbg1	0.512	0.607584123638918	-
6997077	Fbxo9	0.512	0.607584123638918	-
6769607	Pmch	0.513	0.60807665994211	-
6815479	Ptcd2	0.513	0.60807665994211	-
6838249	Srsf2ip	0.513	0.60807665994211	-
6855071	Msh5	0.513	0.60807665994211	-
6874000	Afap1l2	0.513	0.60807665994211	-
6925594	Tmem39b	0.513	0.60807665994211	-
6934348	Fam101a	0.513	0.60807665994211	-
6937482	D5Ertd579e	0.513	0.60807665994211	-
6941130	Sez6l	0.513	0.60807665994211	-
6946119	Npy	0.513	0.60807665994211	+

6960328	Rras	0.513	0.60807665994211	-
6965942	Cnfn	0.513	0.60807665994211	-
6974130	Proz	0.513	0.60807665994211	-
6764243	Ifi205	0.514	0.608461457603364	-
6791230	Arl5c	0.514	0.608461457603364	-
6792497	Mrpl38	0.514	0.608461457603364	-
6805397	Slc17a3	0.514	0.608461457603364	-
6830710	Ndufb9	0.514	0.608461457603364	-
6860911	Zfp474	0.514	0.608461457603364	-
6868032	Syt7	0.514	0.608461457603364	-
6869517	Exoc6	0.514	0.608461457603364	-
6876014	Rapgef1	0.514	0.608461457603364	-
6892380	Trpc4ap	0.514	0.608461457603364	-
6900237	Ovgp1	0.514	0.608461457603364	-
6919184	Mrpl20	0.514	0.608461457603364	-
6963901	Lyrm1	0.514	0.608461457603364	-
7011283	Etd	0.514	0.608461457603364	-
7019821	Tex13	0.514	0.608461457603364	-
6769181	Hmha1	0.515	0.608898512685914	-
6769189	Stk11	0.515	0.608898512685914	-
6791422	Jup	0.515	0.608898512685914	-
6792367	Cd300lb	0.515	0.608898512685914	-
6808213	Slc6a3	0.515	0.608898512685914	-
6836784	Tsta3	0.515	0.608898512685914	-
6854910	Cyp4f13	0.515	0.608898512685914	-
6864695	Sra1	0.515	0.608898512685914	-
6886947	Ermn	0.515	0.608898512685914	-
6889777	Ryr3	0.515	0.608898512685914	-
6902162	Ddah1	0.515	0.608898512685914	-
6916996	Mycbp	0.515	0.608898512685914	-
6980991	Gins4	0.515	0.608898512685914	-
6993846	Tmem205	0.515	0.608898512685914	-
6753591	Zbtb41	0.516	0.60944100681699	-
6766351	Il22ra2	0.516	0.60944100681699	-
6783998	Stard3	0.516	0.60944100681699	-
6869813	D19Ertd386e	0.516	0.60944100681699	-
6881008	Mrps5	0.516	0.60944100681699	-
6883691	Rab22a	0.516	0.60944100681699	-
6891690	Rbbp9	0.516	0.60944100681699	+
6906572	Prss48	0.516	0.60944100681699	-
6931626	Ociad1	0.516	0.60944100681699	-
6946945	Reep1	0.516	0.60944100681699	-
6963744	Nucb2	0.516	0.60944100681699	-
6971321	Zg16	0.516	0.60944100681699	-
6751300	Cops7b	0.517	0.609892695652174	-
6791528	Dusp3	0.517	0.609892695652174	-
6796211	Fntb	0.517	0.609892695652174	-
6832585	Mapk8ip2	0.517	0.609892695652174	-
6856288	Tnfsf14	0.517	0.609892695652174	-
6860780	Tnfaip8	0.517	0.609892695652174	-

6869944	Wnt8b	0.517	0.609892695652174	-
6889232	Trim44	0.517	0.609892695652174	-
6890356	Epb4.2	0.517	0.609892695652174	-
6916663	Slc6a9	0.517	0.609892695652174	-
6964256	Kctd13	0.517	0.609892695652174	-
6991701	Nmnat3	0.517	0.609892695652174	-
6998893	Tmie	0.517	0.609892695652174	-
6752026	Cdh20	0.518	0.609892695652174	-
6757935	Actr1b	0.518	0.609892695652174	-
6770214	Cep290	0.518	0.609892695652174	-
6770901	Tspan8	0.518	0.609892695652174	-
6784042	Thra	0.518	0.609892695652174	-
6784245	Tubg2	0.518	0.609892695652174	-
6790536	Gdpd1	0.518	0.609892695652174	-
6790902	Nme1	0.518	0.609892695652174	-
6823058	Dnajc9	0.518	0.609892695652174	-
6832146	Csdc2	0.518	0.609892695652174	-
6846440	Tmem45a	0.518	0.609892695652174	-
6854826	Cbs	0.518	0.609892695652174	-
6855145	Mrps18b	0.518	0.609892695652174	-
6875723	Clic3	0.518	0.609892695652174	-
6878473	Ppp1r1c	0.518	0.609892695652174	-
6886117	Strbp	0.518	0.609892695652174	-
6922625	2310002L09Rik	0.518	0.609892695652174	-
6939353	2610024G14Rik	0.518	0.609892695652174	-
6941128	Asphd2	0.518	0.609892695652174	-
6955062	Tex261	0.518	0.609892695652174	-
6960621	Htatip2	0.518	0.609892695652174	-
6979851	Tsnax	0.518	0.609892695652174	-
6760804	Pdcd1	0.519	0.609892695652174	-
6764209	Darc	0.519	0.609892695652174	-
6768333	Pbld	0.519	0.609892695652174	-
6769449	Ric8b	0.519	0.609892695652174	-
6774197	Edar	0.519	0.609892695652174	-
6779360	Fam161a	0.519	0.609892695652174	-
6779795	Asb3	0.519	0.609892695652174	-
6791139	Nfe2l1	0.519	0.609892695652174	-
6804608	Pitrm1	0.519	0.609892695652174	-
6868053	Vps37c	0.519	0.609892695652174	-
6898063	Kcnab1	0.519	0.609892695652174	-
6908569	Snx7	0.519	0.609892695652174	-
6919895	Necab1	0.519	0.609892695652174	-
6934024	Gm15800	0.519	0.609892695652174	-
6938844	Phox2b	0.519	0.609892695652174	-
6951483	Dlx5	0.519	0.609892695652174	-
6971600	Cuzd1	0.519	0.609892695652174	-
6977782	Tnpo2	0.519	0.609892695652174	-
6987297	Olfr77	0.519	0.609892695652174	-
6988741	Bace1	0.519	0.609892695652174	-
6990859	Irak1bp1	0.519	0.609892695652174	-

6995384	Fam55b	0.519	0.609892695652174	-
7011394	Fhl1	0.519	0.609892695652174	-
6765202	Mfsd7b	0.52	0.61016584179908	-
6791626	Ccdc43	0.52	0.61016584179908	-
6804974	Gli3	0.52	0.61016584179908	-
6832947	Arid2	0.52	0.61016584179908	-
6843133	4931408A02Rik	0.52	0.61016584179908	-
6859967	Fam53c	0.52	0.61016584179908	-
6866862	Setbp1	0.52	0.61016584179908	-
6876532	Lhx2	0.52	0.61016584179908	-
6925149	Mfsd2a	0.52	0.61016584179908	+
6926267	Pink1	0.52	0.61016584179908	-
6935406	2810453I06Rik	0.52	0.61016584179908	-
6943195	Slc7a1	0.52	0.61016584179908	-
6957412	Olr1	0.52	0.61016584179908	-
6972750	Peg3	0.52	0.61016584179908	-
6979655	Cdh15	0.52	0.61016584179908	-
6994049	Herpud2	0.52	0.61016584179908	-
7015449	Wdr13	0.52	0.61016584179908	-
6748605	Hs6st1	0.521	0.610226555728896	-
6759792	Resp18	0.521	0.610226555728896	-
6775322	C030046I01Rik	0.521	0.610226555728896	-
6782818	Zfp207	0.521	0.610226555728896	-
6784709	Psmd12	0.521	0.610226555728896	-
6809586	--	0.521	0.610226555728896	-
6859441	9530053H22	0.521	0.610226555728896	-
6870569	Vti1a	0.521	0.610226555728896	-
6873171	Avpi1	0.521	0.610226555728896	-
6877924	Bbs5	0.521	0.610226555728896	-
6883097	Sys1	0.521	0.610226555728896	-
6894253	Chrna4	0.521	0.610226555728896	-
6916089	Dhcr24	0.521	0.610226555728896	-
6937844	Ldb2	0.521	0.610226555728896	-
6954633	Elmod3	0.521	0.610226555728896	-
6983262	Plvap	0.521	0.610226555728896	-
6984266	Irx3	0.521	0.610226555728896	-
6985252	Hp	0.521	0.610226555728896	-
6989732	Anp32a	0.521	0.610226555728896	-
7013997	Wbp5	0.521	0.610226555728896	-
7014769	Smpx	0.521	0.610226555728896	-
6749301	C230029F24Rik	0.522	0.610498312418866	-
6775886	Nr1h4	0.522	0.610498312418866	-
6778414	Zmat5	0.522	0.610498312418866	-
6778419	Nipsnap1	0.522	0.610498312418866	-
6792781	Arhgdia	0.522	0.610498312418866	-
6849397	Wdr24	0.522	0.610498312418866	-
6851291	Clpp	0.522	0.610498312418866	-
6852753	Abcg8	0.522	0.610498312418866	-
6870897	Grk5	0.522	0.610498312418866	-
6892393	Eif6	0.522	0.610498312418866	-

6899856	Sec22b	0.522	0.610498312418866	-
6902518	Usp33	0.522	0.610498312418866	-
6935120	BC055004	0.522	0.610498312418866	-
6941240	Hnf1a	0.522	0.610498312418866	-
6941682	Mapkapk5	0.522	0.610498312418866	-
6969950	Olfr78	0.522	0.610498312418866	-
6977682	Gipc1	0.522	0.610498312418866	-
6757945	Tmem131	0.523	0.610716495290763	-
6777346	Mdm2	0.523	0.610716495290763	-
6782129	Tm4sf5	0.523	0.610716495290763	-
6832714	Smgc	0.523	0.610716495290763	-
6838797	Cbx5	0.523	0.610716495290763	-
6839334	Nubp1	0.523	0.610716495290763	-
6875703	Anapc2	0.523	0.610716495290763	-
6878430	Ube2e3	0.523	0.610716495290763	-
6898873	Fbxw7	0.523	0.610716495290763	+
6900707	Dbt	0.523	0.610716495290763	-
6908041	Ahcyl1	0.523	0.610716495290763	-
6917383	Pum1	0.523	0.610716495290763	-
6925974	Dhdds	0.523	0.610716495290763	-
6959577	Alkbh6	0.523	0.610716495290763	-
6960142	Lim2	0.523	0.610716495290763	-
6965979	Ccdc97	0.523	0.610716495290763	-
6995412	Htr3a	0.523	0.610716495290763	-
7016808	Hs6st2	0.523	0.610716495290763	-
6754537	Tnfsf18	0.524	0.61119765233903	-
6798933	Kcns3	0.524	0.61119765233903	-
6805601	Prl2c1	0.524	0.61119765233903	-
6817956	Nek4	0.524	0.61119765233903	-
6830671	D15Ertd621e	0.524	0.61119765233903	-
6873744	Xpnpep1	0.524	0.61119765233903	-
6885504	Surf1	0.524	0.61119765233903	-
6900228	Adora3	0.524	0.61119765233903	-
6925076	Dem1	0.524	0.61119765233903	-
6955011	Ino80b	0.524	0.61119765233903	-
6957044	Apobec1	0.524	0.61119765233903	-
6959011	Ckm	0.524	0.61119765233903	-
6966453	Chst8	0.524	0.61119765233903	-
6759043	Wdr12	0.525	0.611308805790109	-
6790940	Luc7l3	0.525	0.611308805790109	-
6792324	4932435O22Rik	0.525	0.611308805790109	-
6797572	Serpina5	0.525	0.611308805790109	-
6798229	2810002N01Rik	0.525	0.611308805790109	-
6808339	Mctp1	0.525	0.611308805790109	+
6826179	Dnajc15	0.525	0.611308805790109	-
6833276	Slc4a8	0.525	0.611308805790109	-
6850793	Frs3	0.525	0.611308805790109	-
6913010	Ccdc107	0.525	0.611308805790109	-
6923212	Cdkn2a	0.525	0.611308805790109	-
6925148	Cap1	0.525	0.611308805790109	-

6939976	U90926	0.525	0.611308805790109	-
6939985	Naaa	0.525	0.611308805790109	-
6946309	9430076C15Rik	0.525	0.611308805790109	-
6946920	Cd8a	0.525	0.611308805790109	-
6949063	Thumpd3	0.525	0.611308805790109	-
6950147	Clec1b	0.525	0.611308805790109	-
6983162	Ncan	0.525	0.611308805790109	+
6988706	Mpzl2	0.525	0.611308805790109	-
6757744	Bag2	0.526	0.611735283993115	-
6774704	Zfp365	0.526	0.611735283993115	-
6784257	Psme3	0.526	0.611735283993115	-
6847627	Rwdd2b	0.526	0.611735283993115	-
6850046	Btnl3	0.526	0.611735283993115	-
6864747	Taf7	0.526	0.611735283993115	-
6865958	Cdx1	0.526	0.611735283993115	-
6881473	Ankrd5	0.526	0.611735283993115	-
6894283	Arfrp1	0.526	0.611735283993115	-
6907887	Wnt2b	0.526	0.611735283993115	-
6918900	Tnfrsf9	0.526	0.611735283993115	-
6926615	Pramef12	0.526	0.611735283993115	-
6940595	Hsd17b11	0.526	0.611735283993115	-
6977144	Hsh2d	0.526	0.611735283993115	-
6770661	Csrp2	0.527	0.612160735774454	-
6774311	Unc5b	0.527	0.612160735774454	-
6864527	Gfra3	0.527	0.612160735774454	-
6874190	2700078E11Rik	0.527	0.612160735774454	-
6890898	Idh3b	0.527	0.612160735774454	-
6892955	Tnnc2	0.527	0.612160735774454	-
6933512	Tpst2	0.527	0.612160735774454	-
6951756	B630005N14Rik	0.527	0.612160735774454	-
6960243	Klk1b24	0.527	0.612160735774454	-
6962913	Chrdl2	0.527	0.612160735774454	-
6968830	BC048679	0.527	0.612160735774454	-
6972314	H19	0.527	0.612160735774454	-
6979319	Clec3a	0.527	0.612160735774454	-
7014098	Trap1a	0.527	0.612160735774454	-
6778984	Cnrip1	0.528	0.612742979819665	-
6780977	Gdf9	0.528	0.612742979819665	-
6783500	Cox11	0.528	0.612742979819665	-
6792107	Fam20a	0.528	0.612742979819665	-
6802371	Rps6kl1	0.528	0.612742979819665	-
6818956	Rnase4	0.528	0.612742979819665	-
6841142	Cd200r2	0.528	0.612742979819665	-
6878838	Pramel6	0.528	0.612742979819665	-
6889219	Ldlrad3	0.528	0.612742979819665	-
6969253	l7Rn6	0.528	0.612742979819665	-
6976307	Hand2	0.528	0.612742979819665	-
6782808	Adap2	0.529	0.613008574858515	-
6811117	Tbce	0.529	0.613008574858515	-
6815345	Hexb	0.529	0.613008574858515	-

6824875	Gzmn	0.529	0.613008574858515	-
6839078	Mgrn1	0.529	0.613008574858515	-
6845060	AI480653	0.529	0.613008574858515	-
6855610	Hsp90ab1	0.529	0.613008574858515	-
6881771	Pcsk2	0.529	0.613008574858515	-
6894313	4930526D03Rik	0.529	0.613008574858515	-
6897592	Spg20	0.529	0.613008574858515	-
6907935	Atp5f1	0.529	0.613008574858515	-
6931740	Pdgfra	0.529	0.613008574858515	-
6938679	Tlr6	0.529	0.613008574858515	-
6972201	B230206H07Rik	0.529	0.613008574858515	-
6974129	F10	0.529	0.613008574858515	-
6985851	Cox4nb	0.529	0.613008574858515	-
6987352	Icam5	0.529	0.613008574858515	+
6767563	Rtn4ip1	0.53	0.613220890410959	-
6773068	Hint3	0.53	0.613220890410959	-
6785591	Drg1	0.53	0.613220890410959	-
6791423	1110036O03Rik	0.53	0.613220890410959	-
6820268	--	0.53	0.613220890410959	-
6830619	Zhx2	0.53	0.613220890410959	-
6843171	Olig1	0.53	0.613220890410959	-
6855719	Gnmt	0.53	0.613220890410959	-
6899767	Itga10	0.53	0.613220890410959	-
6912582	Akirin2	0.53	0.613220890410959	-
6925577	Fam167b	0.53	0.613220890410959	-
6925910	Nudc	0.53	0.613220890410959	-
6926564	Pdpn	0.53	0.613220890410959	-
6935018	Myl10	0.53	0.613220890410959	-
6935979	Cldn12	0.53	0.613220890410959	-
6949153	Slc6a1	0.53	0.613220890410959	-
6957025	Klrg1	0.53	0.613220890410959	-
7020169	Tsr2	0.53	0.613220890410959	-
6753108	Klhdc8a	0.531	0.613327692307692	-
6753430	Ddx59	0.531	0.613327692307692	-
6755224	Pex19	0.531	0.613327692307692	-
6758139	Mfsd9	0.531	0.613327692307692	-
6759251	Gpr1	0.531	0.613327692307692	-
6759297	Klf7	0.531	0.613327692307692	-
6775147	Cabin1	0.531	0.613327692307692	-
6788122	Rmnd5b	0.531	0.613327692307692	-
6793672	Pdia6	0.531	0.613327692307692	-
6836358	Ndrg1	0.531	0.613327692307692	-
6916109	Tmem59	0.531	0.613327692307692	-
6916705	Hyi	0.531	0.613327692307692	-
6925381	Ncdn	0.531	0.613327692307692	-
6938729	Pds5a	0.531	0.613327692307692	-
6952376	Tnpo3	0.531	0.613327692307692	-
6964247	Tbx6	0.531	0.613327692307692	-
6977083	Fam32a	0.531	0.613327692307692	-
6977697	Prkaca	0.531	0.613327692307692	-

6990664	Gclc	0.531	0.613327692307692	-
6996703	Cgnl1	0.531	0.613327692307692	-
6756969	Ncoa2	0.532	0.613853142076503	-
6785178	Sap30bp	0.532	0.613853142076503	-
6789448	Camta2	0.532	0.613853142076503	-
6790953	Mycbpap	0.532	0.613853142076503	-
6791856	Cyb561	0.532	0.613853142076503	-
6803297	Gsc	0.532	0.613853142076503	-
6811276	Rala	0.532	0.613853142076503	-
6867677	Pcx	0.532	0.613853142076503	-
6895702	Chmp4c	0.532	0.613853142076503	-
6965115	Cyp2e1	0.532	0.613853142076503	-
6976988	Cope	0.532	0.613853142076503	-
6978880	Nfatc3	0.532	0.613853142076503	-
6761680	Ptpn4	0.533	0.614325117270789	-
6778355	Gal3st1	0.533	0.614325117270789	-
6789443	Slc25a11	0.533	0.614325117270789	-
6791400	Krt35	0.533	0.614325117270789	-
6820015	Nkx2-6	0.533	0.614325117270789	-
6837090	Sox10	0.533	0.614325117270789	-
6849398	Jmjd8	0.533	0.614325117270789	-
6874090	Vax1	0.533	0.614325117270789	-
6876087	Tor1b	0.533	0.614325117270789	-
6881092	Fbln7	0.533	0.614325117270789	-
6901435	Dkk2	0.533	0.614325117270789	-
6958952	Mill1	0.533	0.614325117270789	-
7013835	Tnmd	0.533	0.614325117270789	-
6774274	Anapc16	0.534	0.614586612161472	-
6807652	0610007P08Rik	0.534	0.614586612161472	-
6814827	Rasa1	0.534	0.614586612161472	+
6840818	Fstl1	0.534	0.614586612161472	-
6844653	Bcl6	0.534	0.614586612161472	-
6861437	Htr4	0.534	0.614586612161472	-
6868839	Dmrt3	0.534	0.614586612161472	-
6878038	Rapgef4	0.534	0.614586612161472	+
6900097	Bcas2	0.534	0.614586612161472	-
6900659	Extl2	0.534	0.614586612161472	-
6919267	Tmem68	0.534	0.614586612161472	-
6932240	Slc4a4	0.534	0.614586612161472	-
6955037	Stambp	0.534	0.614586612161472	-
6963977	Otoa	0.534	0.614586612161472	-
6977067	5430437P03Rik	0.534	0.614586612161472	-
6994628	Pate4	0.534	0.614586612161472	-
7010055	Atp6ap2	0.534	0.614586612161472	-
6782682	Sdf2	0.535	0.615055107412754	-
6810893	Gtpbp4	0.535	0.615055107412754	-
6819203	Il25	0.535	0.615055107412754	-
6840019	Senp2	0.535	0.615055107412754	-
6843340	Dopey2	0.535	0.615055107412754	-
6846729	Chmp2b	0.535	0.615055107412754	-

6848040	Cldn14	0.535	0.615055107412754	-
6859778	Ammecr1l	0.535	0.615055107412754	-
6885424	Mamdc4	0.535	0.615055107412754	-
6967059	Saa3	0.535	0.615055107412754	-
6973098	Olfr1346	0.535	0.615055107412754	-
6977795	Dhps	0.535	0.615055107412754	-
6755975	Bpnt1	0.536	0.615055107412754	-
6791084	Ube2z	0.536	0.615055107412754	-
6791727	Sh3d20	0.536	0.615055107412754	-
6817637	Anxa11	0.536	0.615055107412754	-
6818118	Arhgap22	0.536	0.615055107412754	-
6819292	Mcpt1	0.536	0.615055107412754	-
6840539	Rnf168	0.536	0.615055107412754	-
6870841	E330013P04Rik	0.536	0.615055107412754	-
6873158	Exosc1	0.536	0.615055107412754	-
6879691	Wt1	0.536	0.615055107412754	-
6882409	Efcab8	0.536	0.615055107412754	-
6896506	Samd7	0.536	0.615055107412754	-
6899034	Crabp2	0.536	0.615055107412754	-
6899330	Jtb	0.536	0.615055107412754	-
6918138	Spata21	0.536	0.615055107412754	-
6941186	Coro1c	0.536	0.615055107412754	+
6941633	Sdsl	0.536	0.615055107412754	-
6957033	Rimklb	0.536	0.615055107412754	-
6970292	Ovch2	0.536	0.615055107412754	-
6973150	Lrrc68	0.536	0.615055107412754	-
6987638	Vps26b	0.536	0.615055107412754	-
6996127	Lrrc49	0.536	0.615055107412754	-
6998596	Manf	0.536	0.615055107412754	-
6761733	Insig2	0.537	0.615470952421338	-
6771974	BC013529	0.537	0.615470952421338	-
6819214	Thtpa	0.537	0.615470952421338	-
6825402	Extl3	0.537	0.615470952421338	-
6849362	Prss28	0.537	0.615470952421338	-
6860925	Snx2	0.537	0.615470952421338	-
6881455	Plcb4	0.537	0.615470952421338	-
6899722	Anp32e	0.537	0.615470952421338	+
6902519	Zzz3	0.537	0.615470952421338	-
6941273	Rnf10	0.537	0.615470952421338	-
6949191	Syn2	0.537	0.615470952421338	-
6972319	Ins2	0.537	0.615470952421338	-
6972408	Nap1l4	0.537	0.615470952421338	-
6987391	Carm1	0.537	0.615470952421338	-
6752885	Ubxn4	0.538	0.615885811096993	-
6817334	Nudt13	0.538	0.615885811096993	-
6823414	Zcchc24	0.538	0.615885811096993	-
6838394	Fkbp11	0.538	0.615885811096993	-
6847632	Cct8	0.538	0.615885811096993	-
6849456	Ergic1	0.538	0.615885811096993	-
6851907	Zfp161	0.538	0.615885811096993	-

6864698	E230025N22Rik	0.538	0.615885811096993	-
6882279	Tbc1d20	0.538	0.615885811096993	-
6888003	Fkbp7	0.538	0.615885811096993	-
6911421	Ubxn2b	0.538	0.615885811096993	-
6960287	Napsa	0.538	0.615885811096993	-
6975876	Ufsp2	0.538	0.615885811096993	-
6998564	Alas1	0.538	0.615885811096993	-
6753135	Rbbp5	0.539	0.616351836182095	+
6759468	Lancl1	0.539	0.616351836182095	-
6766772	Arhgap18	0.539	0.616351836182095	-
6777925	Mbd6	0.539	0.616351836182095	-
6785427	Fscn2	0.539	0.616351836182095	-
6790517	Dhx40	0.539	0.616351836182095	-
6796048	Hif1a	0.539	0.616351836182095	-
6818314	Lrit1	0.539	0.616351836182095	-
6819877	Scara5	0.539	0.616351836182095	-
6892486	Sla2	0.539	0.616351836182095	-
6926011	Zfp593	0.539	0.616351836182095	-
6960099	Zfp788	0.539	0.616351836182095	-
6988153	Fez1	0.539	0.616351836182095	-
6753089	Slc45a3	0.54	0.616712583453055	-
6769232	Scamp4	0.54	0.616712583453055	-
6799362	9030624G23Rik	0.54	0.616712583453055	-
6811357	Stard3nl	0.54	0.616712583453055	-
6853805	Tcte2	0.54	0.616712583453055	-
6873777	Smndc1	0.54	0.616712583453055	-
6875214	Plxdc2	0.54	0.616712583453055	-
6912896	Ube2r2	0.54	0.616712583453055	-
6933478	Pitpnb	0.54	0.616712583453055	-
6936889	Shh	0.54	0.616712583453055	-
6944966	Lep	0.54	0.616712583453055	-
6962444	Fzd4	0.54	0.616712583453055	-
6970138	Trim3	0.54	0.616712583453055	-
7011393	Slc9a6	0.54	0.616712583453055	-
7014171	Mid2	0.54	0.616712583453055	-
6748437	Khdrbs2	0.541	0.6171765996961	-
6764832	Hlx	0.541	0.6171765996961	-
6789309	Efnb3	0.541	0.6171765996961	-
6801656	Dhrs7	0.541	0.6171765996961	-
6857310	Fam98a	0.541	0.6171765996961	-
6880516	Chac1	0.541	0.6171765996961	-
6917992	Mul1	0.541	0.6171765996961	-
6931355	Uchl1	0.541	0.6171765996961	-
6932263	Npffr2	0.541	0.6171765996961	-
6958216	Kras	0.541	0.6171765996961	-
6985697	Sdr42e1	0.541	0.6171765996961	-
6995825	Tspan3	0.541	0.6171765996961	-
6996834	Unc13c	0.541	0.6171765996961	-
6763059	1700025G04Rik	0.542	0.617587521079258	-
6773147	Clvs2	0.542	0.617587521079258	+

6792794	Notum	0.542	0.617587521079258	-
6805194	Trim27	0.542	0.617587521079258	-
6834743	Ropn1l	0.542	0.617587521079258	-
6839543	Mpv17l	0.542	0.617587521079258	+
6870068	Tmem180	0.542	0.617587521079258	-
6878049	B230120H23Rik	0.542	0.617587521079258	-
6891880	Cd93	0.542	0.617587521079258	-
6900938	Cnn3	0.542	0.617587521079258	-
6940594	Hsd17b13	0.542	0.617587521079258	-
6945344	Stra8	0.542	0.617587521079258	-
6966423	Uba2	0.542	0.617587521079258	-
7017628	Slc10a3	0.542	0.617587521079258	-
6759769	Ihh	0.543	0.61804952413038	-
6775213	1810008A18Rik	0.543	0.61804952413038	-
6782694	Aldoc	0.543	0.61804952413038	-
6875377	BC061194	0.543	0.61804952413038	-
6882180	Entpd6	0.543	0.61804952413038	-
6882878	Top1	0.543	0.61804952413038	-
6892031	2310001A20Rik	0.543	0.61804952413038	-
6898721	Accn5	0.543	0.61804952413038	-
6901138	Camk2d	0.543	0.61804952413038	-
6916099	Cyb5rl	0.543	0.61804952413038	-
6937717	Nkx3-2	0.543	0.61804952413038	-
6981326	Got1l1	0.543	0.61804952413038	-
6999164	Gpd1l	0.543	0.61804952413038	-
6754205	Stx6	0.544	0.618198452741339	-
6789047	AU040829	0.544	0.618198452741339	-
6837143	Cbx7	0.544	0.618198452741339	+
6873129	Tm9sf3	0.544	0.618198452741339	-
6878730	Ssrp1	0.544	0.618198452741339	-
6885728	Wdr34	0.544	0.618198452741339	-
6887703	Slc25a12	0.544	0.618198452741339	-
6890453	Slc30a4	0.544	0.618198452741339	-
6893630	Ctsz	0.544	0.618198452741339	-
6924420	Osbpl9	0.544	0.618198452741339	-
6933983	Iqcd	0.544	0.618198452741339	-
6936725	Cdk5	0.544	0.618198452741339	-
6950391	Cdkn1b	0.544	0.618198452741339	-
6957410	Clec7a	0.544	0.618198452741339	-
6972242	Tollip	0.544	0.618198452741339	-
6972294	Ctsd	0.544	0.618198452741339	-
6979556	Cox4i1	0.544	0.618198452741339	-
6980101	Cd209d	0.544	0.618198452741339	-
6987137	BC017612	0.544	0.618198452741339	-
6750611	Stk16	0.545	0.618710517473118	-
6775473	Gna11	0.545	0.618710517473118	-
6783740	Slc35b1	0.545	0.618710517473118	-
6790135	Accn1	0.545	0.618710517473118	-
6795990	Ppm1a	0.545	0.618710517473118	-
6807323	Slc25a48	0.545	0.618710517473118	-

6814383	Ahrr	0.545	0.618710517473118	-
6818282	4930474N05Rik	0.545	0.618710517473118	-
6933601	Ube3b	0.545	0.618710517473118	-
6942220	Sbds	0.545	0.618710517473118	-
6961963	Zfp710	0.545	0.618710517473118	-
6986090	Egln1	0.545	0.618710517473118	-
6750595	Zfand2b	0.546	0.619013758389262	-
6755540	Pppde1	0.546	0.619013758389262	-
6760490	Hjurp	0.546	0.619013758389262	-
6773485	Cdc40	0.546	0.619013758389262	-
6777960	Nab2	0.546	0.619013758389262	-
6782563	Ssh2	0.546	0.619013758389262	-
6828474	Dab2	0.546	0.619013758389262	-
6855724	Ptcra	0.546	0.619013758389262	-
6870470	Smc3	0.546	0.619013758389262	-
6874946	Kin	0.546	0.619013758389262	-
6897762	Eif2a	0.546	0.619013758389262	-
6899353	Ilf2	0.546	0.619013758389262	-
6952529	Chchd3	0.546	0.619013758389262	-
6952680	Fam180a	0.546	0.619013758389262	-
6953126	Fam131b	0.546	0.619013758389262	-
6978802	Ces3a	0.546	0.619013758389262	-
6769012	C330046G03Rik	0.547	0.619575727097477	-
6780933	Vdac1	0.547	0.619575727097477	-
6789347	4933402P03Rik	0.547	0.619575727097477	-
6843634	Adcy9	0.547	0.619575727097477	-
6850863	Lrfn2	0.547	0.619575727097477	-
6933296	Mtf2	0.547	0.619575727097477	-
6954584	Mrpl35	0.547	0.619575727097477	-
6970857	Gprc5b	0.547	0.619575727097477	-
6978335	Cx3cl1	0.547	0.619575727097477	-
6993067	Abhd5	0.547	0.619575727097477	-
6999404	Plcd1	0.547	0.619575727097477	-
6750143	Unc80	0.548	0.619980912515697	-
6785742	Mrps24	0.548	0.619980912515697	-
6786010	--	0.548	0.619980912515697	-
6787602	Fabp6	0.548	0.619980912515697	-
6791432	Dnajc7	0.548	0.619980912515697	-
6796164	Mthfd1	0.548	0.619980912515697	-
6819735	Xkr6	0.548	0.619980912515697	-
6851269	Ndufa11	0.548	0.619980912515697	-
6861389	Csnk1a1	0.548	0.619980912515697	-
6864062	Celf4	0.548	0.619980912515697	-
6867948	Slc22a6	0.548	0.619980912515697	-
6914007	Orm1	0.548	0.619980912515697	-
6964163	Il21r	0.548	0.619980912515697	-
6984998	Ctrl	0.548	0.619980912515697	-
6753387	Nav1	0.549	0.620385149259972	-
6763231	Lhx4	0.549	0.620385149259972	-
6785259	Sec14l1	0.549	0.620385149259972	-

6791684	4933400C05Rik	0.549	0.620385149259972	-
6805395	Slc17a2	0.549	0.620385149259972	-
6824007	Ghitm	0.549	0.620385149259972	-
6863783	B4galt6	0.549	0.620385149259972	-
6866003	Grpel2	0.549	0.620385149259972	-
6934397	Bri3bp	0.549	0.620385149259972	-
6940006	Scarb2	0.549	0.620385149259972	-
6947915	Vmn1r51	0.549	0.620385149259972	-
6950397	8430419L09Rik	0.549	0.620385149259972	-
6964337	Ctf1	0.549	0.620385149259972	-
6992220	Tusc2	0.549	0.620385149259972	-
6786694	A830031A19Rik	0.55	0.621047794117647	-
6799525	Rsad2	0.55	0.621047794117647	-
6906325	Gucy1b3	0.55	0.621047794117647	-
6914190	Tlr4	0.55	0.621047794117647	-
6935370	Fscn1	0.55	0.621047794117647	+
6944384	Capza2	0.55	0.621047794117647	-
6946250	Evx1	0.55	0.621047794117647	-
6955034	Dguok	0.55	0.621047794117647	-
6959120	1500002O20Rik	0.55	0.621047794117647	-
6769192	Atp5d	0.551	0.621605643208949	-
6795228	Ppp2r3c	0.551	0.621605643208949	-
6850241	H2-M10.5	0.551	0.621605643208949	-
6874079	1700019N19Rik	0.551	0.621605643208949	-
6874954	Sfmbt2	0.551	0.621605643208949	-
6948913	Bhlhe40	0.551	0.621605643208949	-
6955295	Cnbp	0.551	0.621605643208949	-
6988209	Hepacam	0.551	0.621605643208949	-
6993305	9230110C19Rik	0.551	0.621605643208949	-
6995515	Dlat	0.551	0.621605643208949	-
7019158	Nap1l3	0.551	0.621605643208949	-
6773072	Hey2	0.552	0.622110582937203	-
6826975	Klf12	0.552	0.622110582937203	-
6867644	Coro1b	0.552	0.622110582937203	-
6880670	B2m	0.552	0.622110582937203	-
6901657	Ppp3ca	0.552	0.622110582937203	-
6904892	Slc7a11	0.552	0.622110582937203	-
6913094	Zcchc7	0.552	0.622110582937203	-
6917774	Il22ra1	0.552	0.622110582937203	-
6918019	Pla2g2a	0.552	0.622110582937203	-
6947596	Tgfa	0.552	0.622110582937203	-
6962179	Mesdc2	0.552	0.622110582937203	-
6981854	Fgf20	0.552	0.622110582937203	-
6759750	Rnf25	0.553	0.622770166666667	-
6788291	Il13	0.553	0.622770166666667	-
6812444	Ssr1	0.553	0.622770166666667	-
6819750	Rp1l1	0.553	0.622770166666667	-
6906814	Mtx1	0.553	0.622770166666667	-
6917037	Fhl3	0.553	0.622770166666667	-
6917585	Slc9a1	0.553	0.622770166666667	-

6943991	C1galt1	0.553	0.622770166666667	-
6981609	Dctn6	0.553	0.622770166666667	-
6762612	Nek7	0.554	0.623065579227696	-
6769224	Onecut3	0.554	0.623065579227696	-
6777957	Lrp1	0.554	0.623065579227696	-
6781264	1810065E05Rik	0.554	0.623065579227696	-
6781846	Myh4	0.554	0.623065579227696	-
6783242	4632419I22Rik	0.554	0.623065579227696	-
6824223	Fermt2	0.554	0.623065579227696	-
6828790	Zfr	0.554	0.623065579227696	-
6833185	Gpd1	0.554	0.623065579227696	+
6879161	Syt13	0.554	0.623065579227696	-
6903056	Il7	0.554	0.623065579227696	-
6931792	Tmem165	0.554	0.623065579227696	-
6946431	Gsbs	0.554	0.623065579227696	-
6973472	Zfp128	0.554	0.623065579227696	-
6987580	Sep7	0.554	0.623065579227696	-
6992273	Mon1a	0.554	0.623065579227696	-
6769634	Gnptab	0.555	0.623619356447992	-
6812894	Atxn1	0.555	0.623619356447992	-
6870613	Habp2	0.555	0.623619356447992	-
6880675	Trim69	0.555	0.623619356447992	-
6925367	Eif2c1	0.555	0.623619356447992	-
6933399	Zfp605	0.555	0.623619356447992	-
6934938	Por	0.555	0.623619356447992	-
6949011	Cav3	0.555	0.623619356447992	-
6957249	Ndufa9	0.555	0.623619356447992	-
6975566	Tusc3	0.555	0.623619356447992	-
6981447	Tex24	0.555	0.623619356447992	-
6755165	Apoa2	0.556	0.624068438538206	-
6782702	Vtn	0.556	0.624068438538206	-
6783883	Scrn2	0.556	0.624068438538206	-
6798315	4930427A07Rik	0.556	0.624068438538206	-
6825686	Ppp3cc	0.556	0.624068438538206	-
6826081	Serp2	0.556	0.624068438538206	-
6853388	5730437N04Rik	0.556	0.624068438538206	-
6855833	Apobec2	0.556	0.624068438538206	-
6881370	Bmp2	0.556	0.624068438538206	-
6899743	Sv2a	0.556	0.624068438538206	-
6906846	Chrnb2	0.556	0.624068438538206	-
6970065	Trim30a	0.556	0.624068438538206	-
6996990	Gfral	0.556	0.624068438538206	-
6782861	Spaca3	0.557	0.624516551895794	-
6790244	Slfn8	0.557	0.624516551895794	-
6837447	Ttll1	0.557	0.624516551895794	-
6855089	Nfkbil1	0.557	0.624516551895794	-
6859811	Ercc3	0.557	0.624516551895794	-
6868048	Ddb1	0.557	0.624516551895794	-
6868618	Zfand5	0.557	0.624516551895794	+
6876062	Ppp2r4	0.557	0.624516551895794	-

6949077	Ogg1	0.557	0.624516551895794	-
6957762	Erp27	0.557	0.624516551895794	-
6964246	Ypel3	0.557	0.624516551895794	-
6998069	BC043934	0.557	0.624516551895794	-
7018291	Asb12	0.557	0.624516551895794	-
6776978	Zdhhc17	0.558	0.625170950091195	-
6785384	Rnf213	0.558	0.625170950091195	-
6824181	Ptgdr	0.558	0.625170950091195	-
6862085	Cxxc1	0.558	0.625170950091195	-
6872444	Rfx3	0.558	0.625170950091195	-
6896836	Adad1	0.558	0.625170950091195	-
6961109	Mcee	0.558	0.625170950091195	+
6970920	Crym	0.558	0.625170950091195	-
6997398	Elovl4	0.558	0.625170950091195	-
6810702	Akr1c12	0.559	0.625565253395164	-
6841341	Dppa2	0.559	0.625565253395164	-
6848080	Pigp	0.559	0.625565253395164	-
6866643	Me2	0.559	0.625565253395164	-
6917782	Srsf10	0.559	0.625565253395164	-
6930022	Crmp1	0.559	0.625565253395164	-
6938093	Ppargc1a	0.559	0.625565253395164	-
6951396	Pon1	0.559	0.625565253395164	-
6956893	Zfp9	0.559	0.625565253395164	-
6965128	Odf3	0.559	0.625565253395164	-
6982580	Spcs3	0.559	0.625565253395164	-
6997672	Ankrd34c	0.559	0.625565253395164	-
7010073	Usp9x	0.559	0.625565253395164	-
7014034	BC065397	0.559	0.625565253395164	-
6782164	Rabep1	0.56	0.625958643507031	-
6785624	Osbp2	0.56	0.625958643507031	-
6816183	Snx18	0.56	0.625958643507031	-
6818863	Naa30	0.56	0.625958643507031	-
6839733	Hic2	0.56	0.625958643507031	-
6849091	Zfp52	0.56	0.625958643507031	-
6849215	Hcfc1r1	0.56	0.625958643507031	-
6880043	Aven	0.56	0.625958643507031	-
6899649	Vps72	0.56	0.625958643507031	-
6921023	Dcaf12	0.56	0.625958643507031	-
6931274	Ube2k	0.56	0.625958643507031	-
6960246	Klk1b3	0.56	0.625958643507031	-
6965658	Calm3	0.56	0.625958643507031	-
6994668	Ei24	0.56	0.625958643507031	-
6781502	Aldh3a1	0.561	0.626299380421314	-
6794063	Adi1	0.561	0.626299380421314	-
6807437	5133401N09Rik	0.561	0.626299380421314	-
6816708	Rpp14	0.561	0.626299380421314	-
6836795	Puf60	0.561	0.626299380421314	-
6855185	H2-M10.3	0.561	0.626299380421314	-
6864783	Pcdh12	0.561	0.626299380421314	-
6867632	Cabp2	0.561	0.626299380421314	-

6894281	Stmn3	0.561	0.626299380421314	-
6911857	Tmem55a	0.561	0.626299380421314	-
6952168	Ndufa5	0.561	0.626299380421314	-
6965316	Cd81	0.561	0.626299380421314	-
6979721	Rhou	0.561	0.626299380421314	-
6994883	Crtam	0.561	0.626299380421314	-
6996638	Fam81a	0.561	0.626299380421314	-
6751687	Ppp1r7	0.562	0.626535885167464	-
6783914	Arhgap23	0.562	0.626535885167464	-
6811694	Hfe	0.562	0.626535885167464	-
6812035	Uqcrfs1	0.562	0.626535885167464	-
6835104	Azin1	0.562	0.626535885167464	-
6917190	Psmb2	0.562	0.626535885167464	-
6926126	1110049F12Rik	0.562	0.626535885167464	-
6936702	Fam126a	0.562	0.626535885167464	-
6940280	1700010H22Rik	0.562	0.626535885167464	-
6948008	Lsm3	0.562	0.626535885167464	-
6955223	Snrnp27	0.562	0.626535885167464	-
6959235	B9d2	0.562	0.626535885167464	-
6970822	Arl6ip1	0.562	0.626535885167464	-
6990569	Bmp5	0.562	0.626535885167464	-
6992330	Rhoa	0.562	0.626535885167464	-
6995927	Clk3	0.562	0.626535885167464	-
7012007	Gdi1	0.562	0.626535885167464	-
6753126	Dstyk	0.563	0.627029998351739	-
6753391	Csrp1	0.563	0.627029998351739	-
6764123	Ncstn	0.563	0.627029998351739	-
6793416	Fam49a	0.563	0.627029998351739	-
6794339	Twistnb	0.563	0.627029998351739	-
6850784	1700001C19Rik	0.563	0.627029998351739	-
6867845	Gpha2	0.563	0.627029998351739	-
6906912	S100a9	0.563	0.627029998351739	-
6923059	Plin2	0.563	0.627029998351739	-
6941837	Rsrc2	0.563	0.627029998351739	-
6947692	Gkn2	0.563	0.627029998351739	-
6984497	Cnot1	0.563	0.627029998351739	-
6762345	Btg2	0.564	0.627368178450901	+
6769845	Cdk17	0.564	0.627368178450901	-
6798216	Eif5	0.564	0.627368178450901	-
6873393	Ldb1	0.564	0.627368178450901	-
6900608	Olfm3	0.564	0.627368178450901	-
6929364	Galntl5	0.564	0.627368178450901	-
6931835	Paics	0.564	0.627368178450901	-
6952657	3110062M04Rik	0.564	0.627368178450901	-
6960388	Rasip1	0.564	0.627368178450901	-
6968857	Btbd1	0.564	0.627368178450901	-
6972204	Slc25a22	0.564	0.627368178450901	-
6978286	Mt3	0.564	0.627368178450901	-
7015991	Elk1	0.564	0.627368178450901	-
7016713	Aifm1	0.564	0.627368178450901	-

7018590	Hdac8	0.564	0.627368178450901	-
6754102	Nmnat2	0.565	0.62770552449852	-
6755168	Adamts4	0.565	0.62770552449852	-
6763706	Atp1b1	0.565	0.62770552449852	-
6780963	Aff4	0.565	0.62770552449852	-
6809022	Dmgdh	0.565	0.62770552449852	-
6864327	Syt4	0.565	0.62770552449852	-
6864564	Etf1	0.565	0.62770552449852	+
6899251	Pbxip1	0.565	0.62770552449852	-
6909139	Ank2	0.565	0.62770552449852	-
6939703	Ugt2b36	0.565	0.62770552449852	-
6954648	Kcmf1	0.565	0.62770552449852	-
6995083	Trappc4	0.565	0.62770552449852	-
7014863	Rs1	0.565	0.62770552449852	-
7016409	Fam70a	0.565	0.62770552449852	-
7017607	Hcfc1	0.565	0.62770552449852	-
6768339	Herc4	0.566	0.628248377823409	-
6782290	Shpk	0.566	0.628248377823409	-
6796540	Psen1	0.566	0.628248377823409	-
6807263	B230219D22Rik	0.566	0.628248377823409	-
6838655	Krt5	0.566	0.628248377823409	-
6839718	Ypel1	0.566	0.628248377823409	-
6855041	Ppt2	0.566	0.628248377823409	-
6873217	Got1	0.566	0.628248377823409	-
6883267	Cse1l	0.566	0.628248377823409	-
6886009	Rabepk	0.566	0.628248377823409	-
6973282	Rps5	0.566	0.628248377823409	-
6772864	Tmem200a	0.567	0.62863549101649	-
6777919	Pip4k2c	0.567	0.62863549101649	-
6783945	Lasp1	0.567	0.62863549101649	-
6784783	Gna13	0.567	0.62863549101649	-
6786262	Pno1	0.567	0.62863549101649	-
6790699	Hlf	0.567	0.62863549101649	-
6837428	Cyb5r3	0.567	0.62863549101649	-
6838492	Lima1	0.567	0.62863549101649	-
6841201	Gcet2	0.567	0.62863549101649	-
6854453	Rab40c	0.567	0.62863549101649	-
6861711	Slmo1	0.567	0.62863549101649	-
6889893	Scg5	0.567	0.62863549101649	-
6910948	Srsf11	0.567	0.62863549101649	-
6955205	Pcyox1	0.567	0.62863549101649	-
6768925	2610028H24Rik	0.568	0.629227969505697	-
6844110	4921513D23Rik	0.568	0.629227969505697	-
6862130	Dym	0.568	0.629227969505697	-
6919212	Tnfrsf18	0.568	0.629227969505697	-
6924892	Cdc20	0.568	0.629227969505697	-
6965211	Ap2a2	0.568	0.629227969505697	-
6967017	Emp3	0.568	0.629227969505697	-
6985876	1700018B08Rik	0.568	0.629227969505697	-
6988603	Thy1	0.568	0.629227969505697	-

7017520	Gabra3	0.568	0.629227969505697	-
6781941	Myh10	0.569	0.629767895167895	-
6782569	Coro6	0.569	0.629767895167895	-
6812968	Nup153	0.569	0.629767895167895	-
6886355	Gtdc1	0.569	0.629767895167895	-
6908551	D3Bwg0562e	0.569	0.629767895167895	-
6959092	Zfp180	0.569	0.629767895167895	-
6963364	Rpl27a	0.569	0.629767895167895	-
6983351	Large	0.569	0.629767895167895	-
6995066	Abcg4	0.569	0.629767895167895	-
6995817	Etfa	0.569	0.629767895167895	-
7016321	Sep6	0.569	0.629767895167895	-
6748503	Rab23	0.57	0.630152159685864	+
6770164	B530045E10Rik	0.57	0.630152159685864	-
6777973	Rdh7	0.57	0.630152159685864	-
6778278	Eif4enif1	0.57	0.630152159685864	-
6807178	Faf2	0.57	0.630152159685864	-
6812778	Ranbp9	0.57	0.630152159685864	-
6836224	Gsdmc	0.57	0.630152159685864	-
6871314	Rtn3	0.57	0.630152159685864	-
6901780	Tspan5	0.57	0.630152159685864	+
6929850	Add1	0.57	0.630152159685864	-
6940330	Hnrnpd	0.57	0.630152159685864	-
6964330	Rnf40	0.57	0.630152159685864	-
6992674	Osbpl10	0.57	0.630152159685864	-
7013843	Cstf2	0.57	0.630152159685864	-
6753706	Glrx2	0.571	0.630381014622988	-
6754437	Rfwd2	0.571	0.630381014622988	+
6769204	Ndufs7	0.571	0.630381014622988	-
6788368	Cdc42se2	0.571	0.630381014622988	-
6796052	Snapc1	0.571	0.630381014622988	-
6798115	Wdr20a	0.571	0.630381014622988	-
6829612	Pgcp	0.571	0.630381014622988	-
6847198	Nrip1	0.571	0.630381014622988	-
6849589	Srsf3	0.571	0.630381014622988	-
6859790	Lims2	0.571	0.630381014622988	-
6871275	Nudt22	0.571	0.630381014622988	-
6882767	Actr5	0.571	0.630381014622988	-
6895997	Dnajc5b	0.571	0.630381014622988	-
6905145	Ufm1	0.571	0.630381014622988	-
6949732	Clec4b1	0.571	0.630381014622988	-
6959228	Exosc5	0.571	0.630381014622988	-
6989985	Ppib	0.571	0.630381014622988	-
6752083	Phlpp1	0.572	0.630712140992167	-
6778298	Selm	0.572	0.630712140992167	-
6788635	Rnf187	0.572	0.630712140992167	-
6792789	Mafg	0.572	0.630712140992167	-
6855189	H2-M10.4	0.572	0.630712140992167	-
6868649	Fam108b	0.572	0.630712140992167	-
6899104	Cct3	0.572	0.630712140992167	-

6924866	Klf17	0.572	0.630712140992167	-
6925549	S100pbp	0.572	0.630712140992167	-
6935895	C030048B08Rik	0.572	0.630712140992167	-
6948878	Lrrn1	0.572	0.630712140992167	+
6949717	Necap1	0.572	0.630712140992167	-
6959133	Ethe1	0.572	0.630712140992167	-
6990243	Bnip2	0.572	0.630712140992167	-
6995454	Ncam1	0.572	0.630712140992167	-
6750149	Rpe	0.573	0.63119677208999	-
6755237	Kcnj10	0.573	0.63119677208999	-
6768198	Pcbd1	0.573	0.63119677208999	-
6792301	Cpsf4l	0.573	0.63119677208999	-
6829549	Cmbl	0.573	0.63119677208999	-
6856197	Ubxn6	0.573	0.63119677208999	-
6867849	Ehd1	0.573	0.63119677208999	-
6925595	Khdrbs1	0.573	0.63119677208999	-
6934130	Arpc3	0.573	0.63119677208999	-
6937654	Hs3st1	0.573	0.63119677208999	-
6957679	Hebp1	0.573	0.63119677208999	+
6992918	Slc25a38	0.573	0.63119677208999	-
6760714	Ndufa10	0.574	0.631546651495449	-
6790547	Ppm1e	0.574	0.631546651495449	-
6841399	Ift57	0.574	0.631546651495449	-
6842941	2810407A14Rik	0.574	0.631546651495449	-
6845146	Pak2	0.574	0.631546651495449	-
6852220	Yipf4	0.574	0.631546651495449	-
6862316	Atp5a1	0.574	0.631546651495449	-
6868582	Aldh1a1	0.574	0.631546651495449	-
6877964	Gorasp2	0.574	0.631546651495449	-
6882352	Hck	0.574	0.631546651495449	-
6900990	Gclm	0.574	0.631546651495449	-
6918335	9430007A20Rik	0.574	0.631546651495449	-
6981621	Leprotl1	0.574	0.631546651495449	-
7010772	Atp1b4	0.574	0.631546651495449	-
6762125	Il20	0.575	0.631546651495449	-
6775355	Gamt	0.575	0.631546651495449	-
6777614	Tmem5	0.575	0.631546651495449	-
6781214	Galnt10	0.575	0.631546651495449	-
6791175	Npepps	0.575	0.631546651495449	-
6798634	Fkbp1b	0.575	0.631546651495449	-
6810954	Zmynd11	0.575	0.631546651495449	-
6818104	E130203B14Rik	0.575	0.631546651495449	-
6849237	Prss21	0.575	0.631546651495449	-
6850502	Tnfrsf21	0.575	0.631546651495449	-
6883113	Dnttip1	0.575	0.631546651495449	-
6892308	Pxmp4	0.575	0.631546651495449	-
6901334	Pla2g12a	0.575	0.631546651495449	-
6903095	Mrps28	0.575	0.631546651495449	-
6908103	Atxn7l2	0.575	0.631546651495449	-
6917393	Laptm5	0.575	0.631546651495449	-

6926023	Man1c1	0.575	0.631546651495449	-
6956587	Sec13	0.575	0.631546651495449	-
6965015	Ppp2r2d	0.575	0.631546651495449	-
6974601	Ccdc70	0.575	0.631546651495449	-
6983103	Lzts1	0.575	0.631546651495449	-
7020870	Hccs	0.575	0.631546651495449	-
6749817	Abi2	0.576	0.631823376623377	-
6775258	Pdxk	0.576	0.631823376623377	-
6778764	Vwc2	0.576	0.631823376623377	-
6782704	Poldip2	0.576	0.631823376623377	-
6791540	Tmem101	0.576	0.631823376623377	-
6793249	Laptm4a	0.576	0.631823376623377	-
6825511	Ppp2r2a	0.576	0.631823376623377	-
6849310	Zfp598	0.576	0.631823376623377	-
6850877	Kif6	0.576	0.631823376623377	-
6888502	Pramel7	0.576	0.631823376623377	-
6916238	Eps15	0.576	0.631823376623377	-
6926020	Fam54b	0.576	0.631823376623377	-
6935116	Cops6	0.576	0.631823376623377	-
6957174	Tuba3a	0.576	0.631823376623377	-
6973435	Usp29	0.576	0.631823376623377	-
6979144	Znrf1	0.576	0.631823376623377	-
6890312	Ttbk2	0.577	0.632663529411765	-
6921550	Acnat2	0.577	0.632663529411765	-
6935269	Nudt1	0.577	0.632663529411765	-
6942694	Psmg3	0.577	0.632663529411765	-
6988366	Scn3b	0.577	0.632663529411765	+
6750567	Wnt6	0.578	0.633246209971626	-
6771635	Mip	0.578	0.633246209971626	-
6815086	Papd4	0.578	0.633246209971626	-
6818212	Glud1	0.578	0.633246209971626	-
6833503	Copz1	0.578	0.633246209971626	-
6899254	Kcnn3	0.578	0.633246209971626	-
6900928	Alg14	0.578	0.633246209971626	-
6909430	Hadh	0.578	0.633246209971626	-
6945775	Gstk1	0.578	0.633246209971626	-
6967862	Klf13	0.578	0.633246209971626	-
6766595	Stx7	0.579	0.633725277395319	-
6768860	Upb1	0.579	0.633725277395319	-
6783033	E230016K23Rik	0.579	0.633725277395319	-
6789541	Spns2	0.579	0.633725277395319	-
6824763	Slc7a8	0.579	0.633725277395319	-
6855057	Stk19	0.579	0.633725277395319	-
6870738	Pnlip	0.579	0.633725277395319	-
6921058	Cntfr	0.579	0.633725277395319	-
6967542	Nipa1	0.579	0.633725277395319	-
6969698	Mogat2	0.579	0.633725277395319	-
6985062	Chtf8	0.579	0.633725277395319	-
6999405	Acaa1b	0.579	0.633725277395319	-
6769286	Dohh	0.58	0.63430606134175	-

6778583	Ccm2	0.58	0.63430606134175	-
6812984	Dek	0.58	0.63430606134175	-
6839008	Cluap1	0.58	0.63430606134175	-
6905200	Rfxap	0.58	0.63430606134175	-
6935296	Amz1	0.58	0.63430606134175	-
6970648	Copb1	0.58	0.63430606134175	-
6971280	Sult1a1	0.58	0.63430606134175	-
6978810	Cbfb	0.58	0.63430606134175	-
7019829	Psmd10	0.58	0.63430606134175	-
6791914	Cd79b	0.581	0.634988596845936	-
6836792	Fam83h	0.581	0.634988596845936	-
6867861	Nrxn2	0.581	0.634988596845936	-
6924971	AA415398	0.581	0.634988596845936	-
6937597	Wdr1	0.581	0.634988596845936	-
6952340	Impdh1	0.581	0.634988596845936	-
6960301	Il4i1	0.581	0.634988596845936	-
7010675	Ube2a	0.581	0.634988596845936	-
6766301	Perp	0.582	0.635362145569109	-
6780521	Sox30	0.582	0.635362145569109	-
6815248	Pde8b	0.582	0.635362145569109	-
6838800	Nfe2	0.582	0.635362145569109	-
6839738	Snap29	0.582	0.635362145569109	-
6850011	H2-Oa	0.582	0.635362145569109	-
6865041	Ppp2r2b	0.582	0.635362145569109	-
6865957	Slc6a7	0.582	0.635362145569109	-
6871044	Rbm4	0.582	0.635362145569109	-
6883100	Pigt	0.582	0.635362145569109	-
6913012	Creb3	0.582	0.635362145569109	-
6942240	A330070K13Rik	0.582	0.635362145569109	-
6983234	Ccdc124	0.582	0.635362145569109	-
6983299	Cyp4f18	0.582	0.635362145569109	-
6754536	Tnfsf4	0.583	0.635991443332257	-
6827132	Fbxl3	0.583	0.635991443332257	-
6828422	Ttc33	0.583	0.635991443332257	-
6837028	Rac2	0.583	0.635991443332257	-
6837412	Tcf20	0.583	0.635991443332257	-
6905829	Kpna4	0.583	0.635991443332257	-
6917078	Meaf6	0.583	0.635991443332257	-
6978773	--	0.583	0.635991443332257	-
6998594	Tex264	0.583	0.635991443332257	-
6748832	Pdcl3	0.584	0.636363167231092	-
6749572	Mobkl3	0.584	0.636363167231092	-
6771158	1700006J14Rik	0.584	0.636363167231092	-
6775238	Pfkl	0.584	0.636363167231092	-
6776198	Nudt4	0.584	0.636363167231092	-
6815542	Marveld2	0.584	0.636363167231092	-
6845382	Fam162a	0.584	0.636363167231092	-
6867972	Ubxn1	0.584	0.636363167231092	-
6892139	Trib3	0.584	0.636363167231092	-
6905356	Serp1	0.584	0.636363167231092	-

6922201	Mup21	0.584	0.636363167231092	-
6948916	Arl8b	0.584	0.636363167231092	-
6969992	Olfr630	0.584	0.636363167231092	-
6971301	4930451I11Rik	0.584	0.636363167231092	-
6765129	Kcnk2	0.585	0.636836636056066	-
6783868	Cbx1	0.585	0.636836636056066	-
6792373	AF251705	0.585	0.636836636056066	-
6844324	Zdhhc8	0.585	0.636836636056066	-
6855087	Tnf	0.585	0.636836636056066	-
6868096	Ms4a4b	0.585	0.636836636056066	-
6883030	Ttpal	0.585	0.636836636056066	-
6887859	Atf2	0.585	0.636836636056066	-
6903549	Gyg	0.585	0.636836636056066	+
6956731	Tmem40	0.585	0.636836636056066	-
6982158	Slc25a4	0.585	0.636836636056066	-
7013252	Sh3bgrl	0.585	0.636836636056066	-
6767387	Cd164	0.586	0.637411783644559	-
6839240	Pmm2	0.586	0.637411783644559	-
6892364	Pigu	0.586	0.637411783644559	-
6907153	Bnipl	0.586	0.637411783644559	-
6908684	Ptbp2	0.586	0.637411783644559	-
6947175	Reg2	0.586	0.637411783644559	-
6956509	Srgap3	0.586	0.637411783644559	-
6961184	Tm2d3	0.586	0.637411783644559	-
6962483	Picalm	0.586	0.637411783644559	-
6983184	Crtc1	0.586	0.637411783644559	-
6767354	Wasf1	0.587	0.6379347004423	-
6768897	Chchd10	0.587	0.6379347004423	-
6775370	Mbd3	0.587	0.6379347004423	-
6777956	Nxph4	0.587	0.6379347004423	-
6847338	D16Ertd472e	0.587	0.6379347004423	-
6913118	Zcchc7	0.587	0.6379347004423	-
6929960	Grpel1	0.587	0.6379347004423	-
6942675	Zfand2a	0.587	0.6379347004423	-
6998428	Uba5	0.587	0.6379347004423	-
7009795	Pim2	0.587	0.6379347004423	-
7012265	Tmem47	0.587	0.6379347004423	-
6747343	Rb1cc1	0.588	0.638302835569122	-
6753271	4933406M09Rik	0.588	0.638302835569122	-
6782655	Dhrs13	0.588	0.638302835569122	-
6784041	Csf3	0.588	0.638302835569122	-
6784266	Rundc1	0.588	0.638302835569122	-
6811656	Abt1	0.588	0.638302835569122	-
6817033	Synpr	0.588	0.638302835569122	-
6829598	Sdc2	0.588	0.638302835569122	-
6836558	Fam135b	0.588	0.638302835569122	+
6917593	Nr0b2	0.588	0.638302835569122	-
6931375	Tmem33	0.588	0.638302835569122	-
6988714	Scn4b	0.588	0.638302835569122	-
6992469	Prss45	0.588	0.638302835569122	-

6996343	Ostb	0.588	0.638302835569122	-
6756386	Lpgat1	0.589	0.639029062299293	-
6789324	Mpdu1	0.589	0.639029062299293	-
6805200	Gpx6	0.589	0.639029062299293	-
6911091	Clk2	0.589	0.639029062299293	-
6924448	Rnf11	0.589	0.639029062299293	-
6943974	Tac1	0.589	0.639029062299293	-
6964090	Aqp8	0.589	0.639029062299293	-
6758261	Fhl2	0.59	0.639395348837209	-
6769268	Dapk3	0.59	0.639395348837209	-
6806245	Ly86	0.59	0.639395348837209	-
6806514	Gm5082	0.59	0.639395348837209	-
6817617	Ppif	0.59	0.639395348837209	-
6829495	Ctnnd2	0.59	0.639395348837209	-
6846497	St3gal6	0.59	0.639395348837209	-
6849294	Pgp	0.59	0.639395348837209	-
6855981	Satb1	0.59	0.639395348837209	-
6929628	4930471M23Rik	0.59	0.639395348837209	-
6932598	Mrpl1	0.59	0.639395348837209	-
6957051	Slc2a3	0.59	0.639395348837209	+
6959265	Cyp2a4	0.59	0.639395348837209	-
7017513	Gabre	0.59	0.639395348837209	-
6819241	Dhrs4	0.591	0.639812064407594	-
6832412	Gramd4	0.591	0.639812064407594	-
6835972	Tmem65	0.591	0.639812064407594	-
6865238	Cdo1	0.591	0.639812064407594	-
6868038	Cpsf7	0.591	0.639812064407594	-
6877963	Gad1	0.591	0.639812064407594	-
6887559	Mettl5	0.591	0.639812064407594	-
6895589	Stmn2	0.591	0.639812064407594	-
6897707	Rnf13	0.591	0.639812064407594	-
6924983	Guca2b	0.591	0.639812064407594	-
6971410	Prss53	0.591	0.639812064407594	-
6983608	Smad1	0.591	0.639812064407594	-
7017578	Haus7	0.591	0.639812064407594	-
6768123	P4ha1	0.592	0.640279151660664	-
6784844	Kcnj16	0.592	0.640279151660664	-
6791023	Myst2	0.592	0.640279151660664	-
6807158	Cplx2	0.592	0.640279151660664	-
6824840	Rabggta	0.592	0.640279151660664	-
6849251	Prss27	0.592	0.640279151660664	-
6882346	Xkr7	0.592	0.640279151660664	-
6899339	Gatad2b	0.592	0.640279151660664	-
6970496	Dkk3	0.592	0.640279151660664	-
6971424	Cox6a2	0.592	0.640279151660664	-
6977060	Use1	0.592	0.640279151660664	-
6995124	Ttc36	0.592	0.640279151660664	-
6793649	E2f6	0.593	0.640847820871651	-
6843614	1700037C18Rik	0.593	0.640847820871651	-
6864518	Nme5	0.593	0.640847820871651	-

6892905	Sdc4	0.593	0.640847820871651	-
6923123	Mllt3	0.593	0.640847820871651	-
6965648	Fkrp	0.593	0.640847820871651	-
6966166	Nfkbib	0.593	0.640847820871651	-
6973287	Zfp446	0.593	0.640847820871651	-
6974583	Defb50	0.593	0.640847820871651	-
6985330	Sf3b3	0.593	0.640847820871651	-
6751544	Scly	0.594	0.641261862917399	-
6780373	C1qtnf2	0.594	0.641261862917399	-
6815682	Srsf12	0.594	0.641261862917399	-
6817680	Asb14	0.594	0.641261862917399	-
6833332	1700011A15Rik	0.594	0.641261862917399	-
6834865	Rpl30	0.594	0.641261862917399	-
6852181	Ehd3	0.594	0.641261862917399	-
6871261	1700019N12Rik	0.594	0.641261862917399	-
6906056	Bche	0.594	0.641261862917399	-
6908029	Kcnc4	0.594	0.641261862917399	-
6925579	Eif3i	0.594	0.641261862917399	-
6925592	Kpna6	0.594	0.641261862917399	-
6944434	Ankrd7	0.594	0.641261862917399	-
6755197	Cd48	0.595	0.641879939331045	-
6785115	Slc9a3r1	0.595	0.641879939331045	-
6788672	Snap47	0.595	0.641879939331045	-
6849064	Zfp677	0.595	0.641879939331045	-
6850138	H2-Q1	0.595	0.641879939331045	-
6874558	Cdnf	0.595	0.641879939331045	-
6989209	Idh3a	0.595	0.641879939331045	-
6995899	Scamp5	0.595	0.641879939331045	-
7018366	Eda2r	0.595	0.641879939331045	-
6762328	Prelp	0.596	0.642292185007974	-
6771704	Dnajc14	0.596	0.642292185007974	-
6775402	Jsrp1	0.596	0.642292185007974	-
6836851	Lrrc24	0.596	0.642292185007974	-
6837094	Slc16a8	0.596	0.642292185007974	-
6883013	Gdap1l1	0.596	0.642292185007974	-
6898181	Schip1	0.596	0.642292185007974	-
6926072	Clic4	0.596	0.642292185007974	-
6937364	Lrpap1	0.596	0.642292185007974	-
6958834	Slc8a2	0.596	0.642292185007974	-
6978735	Cmtm2b	0.596	0.642292185007974	-
6996935	Mapk6	0.596	0.642292185007974	+
6998488	Atp2c1	0.596	0.642292185007974	-
6778939	Vstm2a	0.597	0.642857211155378	+
6839230	BC024814	0.597	0.642857211155378	-
6853849	Dact2	0.597	0.642857211155378	-
6871009	2010003K11Rik	0.597	0.642857211155378	-
6889585	Mett5d1	0.597	0.642857211155378	-
6910137	Clca3	0.597	0.642857211155378	-
6925837	Ppp1r8	0.597	0.642857211155378	-
6926041	Tmem50a	0.597	0.642857211155378	-

6943038	Cyp3a11	0.597	0.642857211155378	-
6983788	Tecr	0.597	0.642857211155378	-
6764068	Nit1	0.598	0.643114117459812	-
6777315	Cpsf6	0.598	0.643114117459812	-
6806831	Rnf144b	0.598	0.643114117459812	-
6815188	Scamp1	0.598	0.643114117459812	-
6848511	Gtf2h5	0.598	0.643114117459812	-
6850116	Ly6g5c	0.598	0.643114117459812	-
6854954	March2	0.598	0.643114117459812	-
6889281	Cat	0.598	0.643114117459812	-
6895532	Pkia	0.598	0.643114117459812	+
6902880	Wls	0.598	0.643114117459812	-
6912461	Map3k7	0.598	0.643114117459812	-
6918167	Hspb7	0.598	0.643114117459812	-
6934905	Bcl7b	0.598	0.643114117459812	-
6945611	Rab19	0.598	0.643114117459812	-
6988098	Srpr	0.598	0.643114117459812	-
6993496	Med17	0.598	0.643114117459812	-
6791257	1810046J19Rik	0.599	0.643882118994591	-
6898309	Nmd3	0.599	0.643882118994591	-
6912042	Ggh	0.599	0.643882118994591	-
6964199	Sbk1	0.599	0.643882118994591	-
6987331	Pin1	0.599	0.643882118994591	-
7015013	Asb9	0.599	0.643882118994591	-
6775415	Sgta	0.6	0.644598139756737	-
6832295	Samm50	0.6	0.644598139756737	-
6839557	Ifitm7	0.6	0.644598139756737	-
6884135	Osbpl2	0.6	0.644598139756737	-
6886013	Fbxw2	0.6	0.644598139756737	-
6917586	Fam46b	0.6	0.644598139756737	-
6960240	Klk1b16	0.6	0.644598139756737	-
6766444	Gm5420	0.601	0.644852242953553	-
6771894	1700052N19Rik	0.601	0.644852242953553	-
6799224	Pqlc3	0.601	0.644852242953553	-
6811487	Zkscan3	0.601	0.644852242953553	-
6824000	Gcap14	0.601	0.644852242953553	-
6830161	Eny2	0.601	0.644852242953553	-
6875642	Il1f5	0.601	0.644852242953553	-
6907148	Mllt11	0.601	0.644852242953553	-
6926948	Rbp7	0.601	0.644852242953553	-
6929018	Magi2	0.601	0.644852242953553	-
6942373	Wbscr16	0.601	0.644852242953553	-
6950485	Atf7ip	0.601	0.644852242953553	-
6984824	Cdh11	0.601	0.644852242953553	-
6985117	Psmd7	0.601	0.644852242953553	-
6989207	Sh2d7	0.601	0.644852242953553	-
7018671	Rlim	0.601	0.644852242953553	-
6761691	Dbi	0.602	0.645310383120489	-
6781954	Rnf222	0.602	0.645310383120489	-
6820574	Sugt1	0.602	0.645310383120489	-

6821985	Gpc6	0.602	0.645310383120489	-
6846518	Olfr172	0.602	0.645310383120489	-
6849374	Tpsg1	0.602	0.645310383120489	-
6870181	Gsto1	0.602	0.645310383120489	-
6875746	Lcn10	0.602	0.645310383120489	-
6885939	Lmx1b	0.602	0.645310383120489	-
6941147	Crybb2	0.602	0.645310383120489	-
6970384	Nrip3	0.602	0.645310383120489	-
7009774	Syp	0.602	0.645310383120489	-
6754909	Lmx1a	0.603	0.645972413793103	-
6756408	Rd3	0.603	0.645972413793103	-
6870125	Ina	0.603	0.645972413793103	+
6893057	Sulf2	0.603	0.645972413793103	-
6911354	Chchd7	0.603	0.645972413793103	-
6916913	Ppt1	0.603	0.645972413793103	-
6988819	2900052N01Rik	0.603	0.645972413793103	-
6999415	Oxsr1	0.603	0.645972413793103	-
6747515	Cspp1	0.604	0.646428763760196	-
6790290	Ccl6	0.604	0.646428763760196	-
6802379	Pgf	0.604	0.646428763760196	-
6852399	Qpct	0.604	0.646428763760196	-
6867879	Bad	0.604	0.646428763760196	-
6925162	Bmp8a	0.604	0.646428763760196	-
6946785	Gng12	0.604	0.646428763760196	-
6955219	Mxd1	0.604	0.646428763760196	-
6978937	Sntb2	0.604	0.646428763760196	-
6992158	Twf2	0.604	0.646428763760196	-
6995918	Lman1l	0.604	0.646428763760196	-
7016312	C330007P06Rik	0.604	0.646428763760196	-
6765222	Nenf	0.605	0.646781900166126	-
6792458	Grb2	0.605	0.646781900166126	+
6812375	Nrn1	0.605	0.646781900166126	+
6840598	Fyttd1	0.605	0.646781900166126	-
6864926	Yipf5	0.605	0.646781900166126	-
6889760	Tmem85	0.605	0.646781900166126	-
6907141	Gabpb2	0.605	0.646781900166126	-
6924905	Wdr65	0.605	0.646781900166126	-
6930883	Tbc1d19	0.605	0.646781900166126	-
6950384	Crebl2	0.605	0.646781900166126	-
6954611	Usp39	0.605	0.646781900166126	-
6978777	4932416K20Rik	0.605	0.646781900166126	-
6985292	Chst4	0.605	0.646781900166126	-
7015432	Gata1	0.605	0.646781900166126	-
6764034	Fcrlb	0.606	0.647185395922238	-
6765513	Mtrf1l	0.606	0.647185395922238	-
6777545	Msrb3	0.606	0.647185395922238	-
6800089	1700108M19Rik	0.606	0.647185395922238	-
6820083	Lgi3	0.606	0.647185395922238	+
6844819	Cldn1	0.606	0.647185395922238	-
6916487	Tal1	0.606	0.647185395922238	-

6930335	Cpeb2	0.606	0.647185395922238	-
6937867	Qdpr	0.606	0.647185395922238	-
6956974	Atp6v1e1	0.606	0.647185395922238	-
6971412	Prss8	0.606	0.647185395922238	-
6992176	Pcbp4	0.606	0.647185395922238	+
7020153	Apex2	0.606	0.647185395922238	-
6787474	Ccng1	0.607	0.647741471888819	-
6823007	Gng2	0.607	0.647741471888819	-
6839528	Bfar	0.607	0.647741471888819	-
6882315	Id1	0.607	0.647741471888819	-
6901489	Ints12	0.607	0.647741471888819	-
6904979	Setd7	0.607	0.647741471888819	-
6946038	Tmem176a	0.607	0.647741471888819	-
6962779	Pak1	0.607	0.647741471888819	-
6965620	Sae1	0.607	0.647741471888819	-
6992436	Ngp	0.607	0.647741471888819	-
6780782	Mgat4b	0.608	0.648450161786757	-
6789825	Gosr1	0.608	0.648450161786757	-
6813386	Mxd3	0.608	0.648450161786757	-
6815733	Fam159b	0.608	0.648450161786757	-
6830651	Wdyhv1	0.608	0.648450161786757	-
6937066	Mpv17	0.608	0.648450161786757	-
7017630	G6pdx	0.608	0.648450161786757	-
6798313	Crip2	0.609	0.649055678233438	-
6816158	Gpx8	0.609	0.649055678233438	-
6843487	Wrb	0.609	0.649055678233438	-
6860188	Pcdhb14	0.609	0.649055678233438	-
6863761	Dsc1	0.609	0.649055678233438	-
6926082	Rcan3	0.609	0.649055678233438	-
6965269	Tnnt3	0.609	0.649055678233438	-
6966359	Apbh	0.609	0.649055678233438	-
6995522	2310030G06Rik	0.609	0.649055678233438	-
6774495	Lrrtm3	0.61	0.649404443043958	-
6775154	Gstt4	0.61	0.649404443043958	-
6787185	Tlx3	0.61	0.649404443043958	-
6791020	Dlx4	0.61	0.649404443043958	-
6792827	Csnk1d	0.61	0.649404443043958	-
6836708	Ly6k	0.61	0.649404443043958	-
6838392	Rnd1	0.61	0.649404443043958	-
6844468	Alg3	0.61	0.649404443043958	-
6862251	Hdhd2	0.61	0.649404443043958	-
6883184	Slc2a10	0.61	0.649404443043958	-
6892897	Svs4	0.61	0.649404443043958	-
6900327	Alx3	0.61	0.649404443043958	-
6919152	Gnb1	0.61	0.649404443043958	-
6957715	Grin2b	0.61	0.649404443043958	-
6783144	Car4	0.611	0.649905863833137	-
6793861	Mboat2	0.611	0.649905863833137	-
6801857	Tex21	0.611	0.649905863833137	-
6803172	Itpk1	0.611	0.649905863833137	-

6835372	Rspo2	0.611	0.649905863833137	-
6850021	Psmb8	0.611	0.649905863833137	-
6887450	Stk39	0.611	0.649905863833137	-
6927075	Park7	0.611	0.649905863833137	-
6942841	Mmd2	0.611	0.649905863833137	+
6998741	Trex1	0.611	0.649905863833137	-
7011953	Ssr4	0.611	0.649905863833137	-
6782090	Gabarap	0.612	0.650559899315661	-
6784810	Prkar1a	0.612	0.650559899315661	-
6785483	Rac3	0.612	0.650559899315661	-
6804497	Asb13	0.612	0.650559899315661	-
6832462	Fam19a5	0.612	0.650559899315661	-
6864326	Rit2	0.612	0.650559899315661	-
6917495	Atpif1	0.612	0.650559899315661	-
6942078	Slc15a4	0.612	0.650559899315661	-
6762471	5730559C18Rik	0.613	0.651315512225804	-
6790199	Ccl1	0.613	0.651315512225804	-
6830480	Mal2	0.613	0.651315512225804	-
6900265	Dram2	0.613	0.651315512225804	-
6925782	Ythdf2	0.613	0.651315512225804	-
6964226	Apob48r	0.613	0.651315512225804	-
6786659	Ahsa2	0.614	0.651711907005969	-
6789316	Atp1b2	0.614	0.651711907005969	-
6797391	Calm1	0.614	0.651711907005969	-
6849785	Ndufv3	0.614	0.651711907005969	-
6856272	Slc25a41	0.614	0.651711907005969	-
6860892	Ftmt	0.614	0.651711907005969	-
6875761	Lcn9	0.614	0.651711907005969	-
6944997	Calu	0.614	0.651711907005969	-
6946235	Hoxa3	0.614	0.651711907005969	-
6951589	Ica1	0.614	0.651711907005969	-
6989553	Hexa	0.614	0.651711907005969	-
6993719	Eif3g	0.614	0.651711907005969	-
7011987	Opn1mw	0.614	0.651711907005969	-
6780007	Pank3	0.615	0.652107493134563	-
6832105	Xpnpep3	0.615	0.652107493134563	-
6832276	Mpped1	0.615	0.652107493134563	-
6833057	Pfkm	0.615	0.652107493134563	-
6848212	Prdm15	0.615	0.652107493134563	-
6850022	Tap2	0.615	0.652107493134563	-
6862188	Zbtb7c	0.615	0.652107493134563	-
6869635	Entpd1	0.615	0.652107493134563	-
6885425	Gm996	0.615	0.652107493134563	+
6888324	Serping1	0.615	0.652107493134563	-
6946511	Lancl2	0.615	0.652107493134563	-
6957690	Pbp2	0.615	0.652107493134563	-
7002886	Hmgcs2	0.615	0.652107493134563	-
6768126	Pla2g12b	0.616	0.652604578237692	-
6821935	Gpc5	0.616	0.652604578237692	-
6824564	Rnase9	0.616	0.652604578237692	-

6840819	Lrrc58	0.616	0.652604578237692	-
6856695	Ndufv2	0.616	0.652604578237692	-
6862462	Pqlc1	0.616	0.652604578237692	-
6873230	Slc25a28	0.616	0.652604578237692	-
6875608	Yme1l1	0.616	0.652604578237692	-
6878694	Med19	0.616	0.652604578237692	-
6888898	Cd82	0.616	0.652604578237692	-
6937522	Wfs1	0.616	0.652604578237692	-
6749835	Cd28	0.617	0.653151966160113	-
6834752	Cct5	0.617	0.653151966160113	-
6864456	D0H4S114	0.617	0.653151966160113	-
6870097	D19Wsu162e	0.617	0.653151966160113	-
6889921	Atpbd4	0.617	0.653151966160113	-
6921208	Gne	0.617	0.653151966160113	-
6956762	Alox5	0.617	0.653151966160113	-
6964374	Fus	0.617	0.653151966160113	-
6967135	Mrgpra2a	0.617	0.653151966160113	-
6973587	Apoe	0.617	0.653151966160113	-
6864378	Proc	0.618	0.653749667318982	-
6872262	Pip5k1b	0.618	0.653749667318982	-
6879763	Dcdc5	0.618	0.653749667318982	-
6885903	Cdk9	0.618	0.653749667318982	-
6926324	Htr6	0.618	0.653749667318982	-
6934126	Vps29	0.618	0.653749667318982	-
6983267	Nxnl1	0.618	0.653749667318982	-
6989290	Rcn2	0.618	0.653749667318982	-
7019652	Rab9b	0.618	0.653749667318982	+
6759765	Cryba2	0.619	0.654551330203443	-
6833184	Smarcd1	0.619	0.654551330203443	-
6873421	Arl3	0.619	0.654551330203443	-
6917713	Runx3	0.619	0.654551330203443	-
6943138	Cdx2	0.619	0.654551330203443	-
6791310	Ccr7	0.62	0.655249863142254	-
6855785	Tomm6	0.62	0.655249863142254	-
6861746	Seh1l	0.62	0.655249863142254	-
6862973	Tmx3	0.62	0.655249863142254	-
6886034	Rab14	0.62	0.655249863142254	-
6899372	S100a4	0.62	0.655249863142254	-
6960347	Pth2	0.62	0.655249863142254	-
6757796	Ccdc115	0.621	0.656050187617261	-
6801929	Max	0.621	0.656050187617261	-
6825494	Dpysl2	0.621	0.656050187617261	-
6876079	Mettl11a	0.621	0.656050187617261	-
6957112	Gm5077	0.621	0.656050187617261	-
6787020	Chac2	0.622	0.656798562275356	-
6863965	Rprd1a	0.622	0.656798562275356	-
6875751	Lcn3	0.622	0.656798562275356	-
6957272	D130058E03	0.622	0.656798562275356	-
6973730	Lrrc8e	0.622	0.656798562275356	-
6985975	Sult5a1	0.622	0.656798562275356	-

6763247	Fam163a	0.623	0.657392207386585	-
6791317	Krt222	0.623	0.657392207386585	-
6881839	Dtd1	0.623	0.657392207386585	-
6883105	Wfdc2	0.623	0.657392207386585	-
6899605	Selenbp2	0.623	0.657392207386585	-
6957844	Lmo3	0.623	0.657392207386585	+
6985005	Lcat	0.623	0.657392207386585	-
6992614	Ubp1	0.623	0.657392207386585	-
6998097	Spsb4	0.623	0.657392207386585	-
6808957	Ankrd34b	0.624	0.658139077499415	-
6847876	Tmem50b	0.624	0.658139077499415	-
6854572	Tcp11	0.624	0.658139077499415	-
6862281	St8sia5	0.624	0.658139077499415	-
6863326	Abhd3	0.624	0.658139077499415	-
6949856	Vamp1	0.624	0.658139077499415	-
6782977	Unc45b	0.625	0.659039481897628	-
6898477	Serpini1	0.625	0.659039481897628	-
6911092	Scamp3	0.625	0.659039481897628	-
6789958	Foxn1	0.626	0.659527870897326	-
6790990	Tmem92-ps	0.626	0.659527870897326	-
6813480	Cxcl14	0.626	0.659527870897326	-
6838621	4732456N10Rik	0.626	0.659527870897326	-
6841410	Cd47	0.626	0.659527870897326	-
6880639	Ckmt1	0.626	0.659527870897326	-
6892102	Psmf1	0.626	0.659527870897326	-
6961991	Hddc3	0.626	0.659527870897326	+
6966308	Tmem147	0.626	0.659527870897326	-
6983526	Tmem184c	0.626	0.659527870897326	-
6995508	Sdhd	0.626	0.659527870897326	-
6811605	Vmn1r211	0.627	0.660324033665835	-
6917152	Trappc3	0.627	0.660324033665835	-
6920742	1810030N24Rik	0.627	0.660324033665835	-
6932364	Cxcl5	0.627	0.660324033665835	-
6985399	Bcar1	0.627	0.660324033665835	-
6784178	Gast	0.628	0.660862171001402	-
6840516	Pigz	0.628	0.660862171001402	-
6856674	Vapa	0.628	0.660862171001402	-
6881204	Mrps26	0.628	0.660862171001402	-
6934902	Tbl2	0.628	0.660862171001402	-
6935496	Bud31	0.628	0.660862171001402	-
6940883	Tmed5	0.628	0.660862171001402	-
6995161	Cd3e	0.628	0.660862171001402	-
7014982	Ap1s2	0.628	0.660862171001402	-
7020724	Glra2	0.628	0.660862171001402	-
6824850	Sdr39u1	0.629	0.66145093767022	-
6831706	Rpl8	0.629	0.66145093767022	-
6833259	Dazap2	0.629	0.66145093767022	-
6860224	Rell2	0.629	0.66145093767022	-
6865240	Atg12	0.629	0.66145093767022	-
6871076	Cnih2	0.629	0.66145093767022	-

6877990	Cybrd1	0.629	0.66145093767022	-
6922672	Ptprd	0.629	0.66145093767022	-
6960144	Nkg7	0.629	0.66145093767022	-
6777286	Rab3ip	0.63	0.662090364725095	-
6783879	Prr15l	0.63	0.662090364725095	-
6791424	Nt5c3l	0.63	0.662090364725095	-
6809522	Serf1	0.63	0.662090364725095	-
6845978	Cd200	0.63	0.662090364725095	-
6907949	BC051070	0.63	0.662090364725095	-
6918901	Uts2	0.63	0.662090364725095	-
6935035	Rabl5	0.63	0.662090364725095	-
6820241	Sucla2	0.631	0.662883550995025	-
6889978	Meis2	0.631	0.662883550995025	-
6971848	Mki67	0.631	0.662883550995025	-
6980126	Elavl1	0.631	0.662883550995025	-
7001591	Tarm1	0.631	0.662883550995025	-
6771931	Ppp1r14c	0.632	0.663263803680982	-
6781352	Jmjd4	0.632	0.663263803680982	-
6785270	Gm11733	0.632	0.663263803680982	-
6839944	Vwa5b2	0.632	0.663263803680982	-
6861408	Sh3tc2	0.632	0.663263803680982	-
6868184	Olfr1443	0.632	0.663263803680982	-
6871574	Oosp1	0.632	0.663263803680982	-
6889517	Fshb	0.632	0.663263803680982	-
6898806	Sfrp2	0.632	0.663263803680982	-
6899683	Ctss	0.632	0.663263803680982	-
6907136	Scnm1	0.632	0.663263803680982	-
6967210	Csrp3	0.632	0.663263803680982	-
7017063	Rbmx	0.632	0.663263803680982	-
6776784	Myf6	0.633	0.663952343992549	-
6777180	Rab21	0.633	0.663952343992549	-
6873307	Mrpl43	0.633	0.663952343992549	-
6882762	Slc32a1	0.633	0.663952343992549	-
6906721	Rhbg	0.633	0.663952343992549	-
6923083	Slc24a2	0.633	0.663952343992549	-
6993854	Elavl3	0.633	0.663952343992549	-
6752539	En1	0.634	0.664588582066398	-
6766110	Hivep2	0.634	0.664588582066398	-
6767460	Snx3	0.634	0.664588582066398	-
6775449	Mrpl54	0.634	0.664588582066398	-
6795768	Ppil5	0.634	0.664588582066398	-
6829656	Laptm4b	0.634	0.664588582066398	-
6871377	Polr2g	0.634	0.664588582066398	-
6901967	Gtf2b	0.634	0.664588582066398	-
6836991	Cacng2	0.635	0.665224031007752	-
6838411	Dhh	0.635	0.665224031007752	-
6852695	Mta3	0.635	0.665224031007752	-
6864702	Ndufa2	0.635	0.665224031007752	-
6896428	Slc2a2	0.635	0.665224031007752	-
6933138	Lrrc8c	0.635	0.665224031007752	-

6949722	Clec4a1	0.635	0.665224031007752	-
6992962	Ctnnb1	0.635	0.665224031007752	-
6798302	Btbd6	0.636	0.666065096094234	-
6820109	Dok2	0.636	0.666065096094234	-
6966494	Nudt19	0.636	0.666065096094234	-
6983280	B3gnt3	0.636	0.666065096094234	-
6785102	Gpr142	0.637	0.666699039653036	-
6787283	Rars	0.637	0.666699039653036	-
6832356	Atxn10	0.637	0.666699039653036	-
6836714	Cyp11b2	0.637	0.666699039653036	-
6949084	Arpc4	0.637	0.666699039653036	-
6974582	Defb1	0.637	0.666699039653036	-
6980968	Ap3m2	0.637	0.666699039653036	-
7019519	Gla	0.637	0.666699039653036	+
6762423	Shisa4	0.638	0.667383853239415	-
6825573	Nefm	0.638	0.667383853239415	-
6846193	Gm4827	0.638	0.667383853239415	-
6882530	Trp53inp2	0.638	0.667383853239415	-
6908788	A730020M07Rik	0.638	0.667383853239415	-
6971610	Ikzf5	0.638	0.667383853239415	-
6980889	Xkr5	0.638	0.667383853239415	-
6896031	4632415L05Rik	0.639	0.66832644532157	-
6967060	Saa4	0.639	0.66832644532157	-
6774875	Phyhipl	0.64	0.669061653902684	-
6797714	Tcl1b2	0.64	0.669061653902684	-
6843184	Ifngr2	0.64	0.669061653902684	-
6854787	Tff3	0.64	0.669061653902684	-
6870562	Tectb	0.64	0.669061653902684	-
6882630	1110008F13Rik	0.64	0.669061653902684	-
6757750	Bend6	0.641	0.669744394618834	-
6833117	Wnt1	0.641	0.669744394618834	-
6855716	Klhdc3	0.641	0.669744394618834	-
6876486	Rabgap1	0.641	0.669744394618834	-
6880945	Usp8	0.641	0.669744394618834	-
6960308	Akt1s1	0.641	0.669744394618834	-
6982214	Stox2	0.641	0.669744394618834	-
6761284	Serpinb3d	0.642	0.670322799969095	-
6769662	Slc5a8	0.642	0.670322799969095	-
6831660	Hsf1	0.642	0.670322799969095	-
6867758	Fibp	0.642	0.670322799969095	-
6869035	Pdcd1lg2	0.642	0.670322799969095	-
6879599	Apip	0.642	0.670322799969095	-
6927240	Rer1	0.642	0.670322799969095	-
6966108	Psmc4	0.642	0.670322799969095	-
7013031	Tsx	0.642	0.670322799969095	-
6756403	Slc30a1	0.643	0.671211339409856	+
6760502	Glrp1	0.643	0.671211339409856	-
6854398	Nubp2	0.643	0.671211339409856	-
6797893	Cyp46a1	0.644	0.671788189888074	-
6882064	8030411F24Rik	0.644	0.671788189888074	-

6884305	Polr3k	0.644	0.671788189888074	-
6891905	Cst3	0.644	0.671788189888074	-
6904806	1700018B24Rik	0.644	0.671788189888074	-
6933805	Fbxo21	0.644	0.671788189888074	-
6934976	Polr2j	0.644	0.671788189888074	-
6941472	Fbxw8	0.644	0.671788189888074	-
6962495	Crebzf	0.644	0.671788189888074	-
6768204	Tbata	0.645	0.672623659232966	+
6803598	Wars	0.645	0.672623659232966	-
6933447	Galnt9	0.645	0.672623659232966	-
6933511	Crybb1	0.645	0.672623659232966	-
6776128	Gm4792	0.646	0.673302791917322	-
6838399	Wnt10b	0.646	0.673302791917322	-
6910648	St6galnac5	0.646	0.673302791917322	-
6959317	Ttc9b	0.646	0.673302791917322	-
6989438	Sema7a	0.646	0.673302791917322	-
7010342	Cdk16	0.646	0.673302791917322	-
7015398	Ppp1r3f	0.646	0.673302791917322	-
6762321	Atp2b4	0.647	0.67408511294426	-
6849698	Zfand3	0.647	0.67408511294426	-
6915070	Sh3gl2	0.647	0.67408511294426	-
6937061	Slc30a3	0.647	0.67408511294426	+
6959144	Ceacam10	0.647	0.67408511294426	-
6759782	Tuba4a	0.648	0.67486683107275	-
6837335	Pppde2	0.648	0.67486683107275	-
6870172	6330577E15Rik	0.648	0.67486683107275	-
6870891	Fam45a	0.648	0.67486683107275	-
6957380	Clec2e	0.648	0.67486683107275	-
6849063	4930432O21Rik	0.649	0.675595902018179	-
6905746	Lxn	0.649	0.675595902018179	-
6914916	Snapc3	0.649	0.675595902018179	-
6922541	Megf9	0.649	0.675595902018179	-
6926842	2810408P10Rik	0.649	0.675595902018179	-
6946948	Immt	0.649	0.675595902018179	-
6753552	Atp6v1g3	0.65	0.676220169361047	-
6761615	Tsn	0.65	0.676220169361047	-
6785669	Cabp7	0.65	0.676220169361047	-
6803890	A730018C14Rik	0.65	0.676220169361047	-
6874631	Phyh	0.65	0.676220169361047	-
6882738	Snhg11	0.65	0.676220169361047	-
6991991	Ryk	0.65	0.676220169361047	-
6992409	Nme6	0.65	0.676220169361047	-
6794331	Atxn7l1	0.651	0.67694783010157	-
6795593	Fam179b	0.651	0.67694783010157	-
6808214	Clptm1l	0.651	0.67694783010157	-
6808314	Glrx	0.651	0.67694783010157	-
6813229	Diras2	0.651	0.67694783010157	-
6894278	Gmeb2	0.651	0.67694783010157	+
6753295	Syt2	0.652	0.677414315368648	-
6762217	Tmcc2	0.652	0.677414315368648	-

6762773	Trove2	0.652	0.677414315368648	-
6767610	Prep	0.652	0.677414315368648	-
6768217	Nodal	0.652	0.677414315368648	-
6788702	Cops3	0.652	0.677414315368648	-
6795780	Klhdc2	0.652	0.677414315368648	-
6848586	T	0.652	0.677414315368648	-
6853944	Psmb1	0.652	0.677414315368648	-
6983123	Zfp869	0.652	0.677414315368648	-
6989921	Cilp	0.652	0.677414315368648	-
6785793	H2afv	0.653	0.677880012290675	-
6837426	Poldip3	0.653	0.677880012290675	-
6854509	Cuta	0.653	0.677880012290675	-
6855056	Cyp21a1	0.653	0.677880012290675	-
6883114	Ube2c	0.653	0.677880012290675	-
6898972	Cd1d2	0.653	0.677880012290675	-
6916092	Acot11	0.653	0.677880012290675	-
6926864	Clcn6	0.653	0.677880012290675	-
6933656	Coq5	0.653	0.677880012290675	-
6934631	Mrps17	0.653	0.677880012290675	-
6992490	Epm2aip1	0.653	0.677880012290675	-
6753192	Etnk2	0.654	0.678553243761996	-
6835973	Tmem65	0.654	0.678553243761996	-
6848087	Dscr3	0.654	0.678553243761996	-
6854506	Nkx2-5	0.654	0.678553243761996	-
6900407	Wdr47	0.654	0.678553243761996	-
6917063	9930104L06Rik	0.654	0.678553243761996	-
6996695	Gcom1	0.654	0.678553243761996	-
6956592	Atp2b2	0.655	0.679486451216704	-
6966405	Abpz	0.655	0.679486451216704	-
6774937	Ube2d1	0.656	0.680262737875998	-
6781463	Smcr8	0.656	0.680262737875998	-
6860038	Paip2	0.656	0.680262737875998	-
6960147	Etfb	0.656	0.680262737875998	-
6971744	Mmp21	0.656	0.680262737875998	-
6792657	Lgals3bp	0.657	0.681090671985272	-
6870957	Ndufs8	0.657	0.681090671985272	-
6885912	Stxbp1	0.657	0.681090671985272	-
6970382	Tmem9b	0.657	0.681090671985272	-
6767825	Vgll2	0.658	0.681656726715217	-
6768075	Fabp7	0.658	0.681656726715217	-
6783884	Mrpl10	0.658	0.681656726715217	-
6876206	Dpm2	0.658	0.681656726715217	-
6883654	Pck1	0.658	0.681656726715217	-
6890068	Rasgrp1	0.658	0.681656726715217	-
6890670	Ciao1	0.658	0.681656726715217	-
6908508	Hiat1	0.658	0.681656726715217	-
6978171	Fto	0.658	0.681656726715217	-
6750552	Rqcd1	0.659	0.682222000919258	-
6768987	Pttg1ip	0.659	0.682222000919258	-
6808964	Serinc5	0.659	0.682222000919258	-

6822459	Pdhb	0.659	0.682222000919258	-
6832182	Sep3	0.659	0.682222000919258	-
6929828	Nat8l	0.659	0.682222000919258	-
6949740	Clec4b2	0.659	0.682222000919258	-
6965264	Tnni2	0.659	0.682222000919258	-
6985340	Ddx19a	0.659	0.682222000919258	-
6771560	Ddit3	0.66	0.682891049689917	-
6782465	Ywhae	0.66	0.682891049689917	-
6808237	Tppp	0.66	0.682891049689917	-
6850821	Treml4	0.66	0.682891049689917	-
6877587	Psmd14	0.66	0.682891049689917	-
6997211	Cox7a2	0.66	0.682891049689917	-
7009947	Mid1ip1	0.66	0.682891049689917	-
6825714	Fgf17	0.661	0.683402494070844	-
6831254	Khdrbs3	0.661	0.683402494070844	-
6854383	Syngr3	0.661	0.683402494070844	-
6899250	Pygo2	0.661	0.683402494070844	-
6906702	Apoa1bp	0.661	0.683402494070844	-
6917972	Eif4g3	0.661	0.683402494070844	-
6923760	Itgb3bp	0.661	0.683402494070844	-
6941293	Gatc	0.661	0.683402494070844	-
6954617	Vamp5	0.661	0.683402494070844	-
6991111	A330041J22Rik	0.661	0.683402494070844	-
6757898	Neurl3	0.662	0.684174671153258	-
6806044	Serpinb9e	0.662	0.684174671153258	-
6949826	Mlf2	0.662	0.684174671153258	-
6951054	Sspn	0.662	0.684174671153258	-
6978945	Vps4a	0.662	0.684174671153258	-
6825364	4930578I06Rik	0.663	0.684893900015288	-
6833100	Cacnb3	0.663	0.684893900015288	-
6838032	Yaf2	0.663	0.684893900015288	-
6931682	Rasl11b	0.663	0.684893900015288	+
6965631	Grlf1	0.663	0.684893900015288	-
6978819	Nol3	0.663	0.684893900015288	-
6837102	Tmem184b	0.664	0.685717255081767	-
6899921	Gdap2	0.664	0.685717255081767	-
6983243	Haus8	0.664	0.685717255081767	-
6986006	Dbndd1	0.664	0.685717255081767	-
6856676	Rab31	0.665	0.686487663280116	-
6884788	Rsu1	0.665	0.686487663280116	-
6899777	Rnf115	0.665	0.686487663280116	-
6940928	Cplx1	0.665	0.686487663280116	-
6961650	Fam174b	0.665	0.686487663280116	-
6755856	Susd4	0.666	0.687205008780637	-
6784590	Dcaf7	0.666	0.687205008780637	-
6817412	Samd8	0.666	0.687205008780637	-
6887205	Kcnh7	0.666	0.687205008780637	-
6945309	Akr1b10	0.666	0.687205008780637	-
6965880	Tex101	0.666	0.687205008780637	-
6763493	Cacybp	0.667	0.688079236641222	-

6814200	BC018507	0.667	0.688079236641222	-
6963946	Uqcrc2	0.667	0.688079236641222	-
6813315	Thoc3	0.668	0.689005647992673	-
6952700	Mtpn	0.668	0.689005647992673	-
6747478	3110035E14Rik	0.669	0.689353107129241	-
6783809	Gip	0.669	0.689353107129241	-
6791913	Gh	0.669	0.689353107129241	-
6792649	Timp2	0.669	0.689353107129241	-
6798418	Rapgef5	0.669	0.689353107129241	-
6820237	Med4	0.669	0.689353107129241	-
6839059	Hmox2	0.669	0.689353107129241	-
6890448	Gatm	0.669	0.689353107129241	-
6925081	Zmpste24	0.669	0.689353107129241	-
6949348	--	0.669	0.689353107129241	-
6955291	Rab43	0.669	0.689353107129241	-
6979043	Txnl4b	0.669	0.689353107129241	-
6983210	Ssbp4	0.669	0.689353107129241	-
6854389	Ndufb10	0.67	0.690278264847145	-
6969291	Tmem126b	0.67	0.690278264847145	+
6771637	Apon	0.671	0.690992455993294	-
6878347	Plekha3	0.671	0.690992455993294	-
6885070	Pip4k2a	0.671	0.690992455993294	-
6887861	Atp5g3	0.671	0.690992455993294	-
6916540	Pik3r3	0.671	0.690992455993294	-
6944514	Kcnd2	0.671	0.690992455993294	-
6903983	Slc7a14	0.672	0.691811381122877	-
6941173	Cmklr1	0.672	0.691811381122877	-
6941804	Il31	0.672	0.691811381122877	-
6962925	Pgm2l1	0.672	0.691811381122877	-
6771985	Tab2	0.673	0.692629807326175	-
6792849	BC017643	0.673	0.692629807326175	-
6854414	Gnptg	0.673	0.692629807326175	-
6978871	Nrn1l	0.673	0.692629807326175	-
6764615	Mixl1	0.674	0.693342163355408	-
6828799	Golph3	0.674	0.693342163355408	-
6914009	Orm2	0.674	0.693342163355408	-
6941266	Cabp1	0.674	0.693342163355408	-
6958904	Ap2s1	0.674	0.693342163355408	-
6992172	Dusp7	0.674	0.693342163355408	-
6757127	Ube2w	0.675	0.694106680870492	-
6957145	Ptms	0.675	0.694106680870492	-
6965863	Irgc1	0.675	0.694106680870492	-
6969818	Plekhb1	0.675	0.694106680870492	-
6992830	Azi2	0.675	0.694106680870492	-
6774405	Ddx50	0.676	0.694553637953319	-
6788812	Usp22	0.676	0.694553637953319	-
6813348	Sncb	0.676	0.694553637953319	-
6863582	Kctd1	0.676	0.694553637953319	-
6881459	6330527O06Rik	0.676	0.694553637953319	-
6933970	Sds	0.676	0.694553637953319	-

6935583	Pdx1	0.676	0.694553637953319	-
6943230	Katnal1	0.676	0.694553637953319	-
6946054	Svs1	0.676	0.694553637953319	-
6973937	Tnfsf13b	0.676	0.694553637953319	-
7020507	Pdha1	0.676	0.694553637953319	-
6863645	Cdh2	0.677	0.695211094224924	-
6869016	Cdc37l1	0.677	0.695211094224924	-
6960454	Myod1	0.677	0.695211094224924	-
6960885	Gabrb3	0.677	0.695211094224924	-
6966284	Aplp1	0.677	0.695211094224924	-
6985828	Zdhhc7	0.677	0.695211094224924	-
6993857	Zfp653	0.677	0.695211094224924	-
6768076	Smpdl3a	0.678	0.69602643573382	-
6838355	Asb8	0.678	0.69602643573382	-
6958938	Ceacam5	0.678	0.69602643573382	-
6993864	Elof1	0.678	0.69602643573382	-
6771581	Tac2	0.679	0.696894205209995	-
6833161	Tmbim6	0.679	0.696894205209995	-
6986064	Agt	0.679	0.696894205209995	-
6803787	Ankrd9	0.68	0.697549719143768	-
6893636	Slmo2	0.68	0.697549719143768	-
6905321	Pfn2	0.68	0.697549719143768	-
6922312	Gm11213	0.68	0.697549719143768	-
6934122	Pptc7	0.68	0.697549719143768	-
6976821	Nat2	0.68	0.697549719143768	-
6995771	Wdr61	0.68	0.697549719143768	-
6748557	Plekhb2	0.681	0.698204536833321	-
6891902	Cst11	0.681	0.698204536833321	-
6902746	Negr1	0.681	0.698204536833321	-
6972710	Ttyh1	0.681	0.698204536833321	-
6988722	Fxyd6	0.681	0.698204536833321	-
6992945	Eif1b	0.681	0.698204536833321	-
7015856	Fundc1	0.681	0.698204536833321	-
6945741	1700034O15Rik	0.682	0.699176756182673	-
6802184	Med6	0.683	0.699936452566922	-
6824291	Gmfb	0.683	0.699936452566922	-
6938710	1110003E01Rik	0.683	0.699936452566922	-
6974158	Grk1	0.683	0.699936452566922	-
7017014	Mmgt1	0.683	0.699936452566922	-
6823100	Usp54	0.684	0.700854954886648	-
6886073	Ndufa8	0.684	0.700854954886648	-
6800079	Tspan13	0.685	0.701666792996286	-
6861278	A730017C20Rik	0.685	0.701666792996286	-
6911332	Tgs1	0.685	0.701666792996286	-
6992328	Nicn1	0.685	0.701666792996286	-
6867987	Ahnak	0.686	0.702478138970978	-
6993198	Aasdhppt	0.686	0.702478138970978	-
6995828	C230081A13Rik	0.686	0.702478138970978	-
7019547	Zmat1	0.686	0.702478138970978	-
6809436	Zfp366	0.687	0.703182458532152	-

6819887	Clu	0.687	0.703182458532152	-
6837006	Pvalb	0.687	0.703182458532152	-
6888309	2700094K13Rik	0.687	0.703182458532152	-
6906903	S100a1	0.687	0.703182458532152	-
6957759	Mgp	0.687	0.703182458532152	-
6892998	4833422F24Rik	0.688	0.703939430648092	-
6942404	Wbscr25	0.688	0.703939430648092	-
6980289	Fam155a	0.688	0.703939430648092	-
6986560	Alkbh8	0.688	0.703939430648092	-
7012085	Vbp1	0.688	0.703939430648092	-
6751505	Cops8	0.689	0.704589178963299	-
6790531	Ypel2	0.689	0.704589178963299	-
6822959	Ube2e2	0.689	0.704589178963299	+
6869879	Nkx2-3	0.689	0.704589178963299	-
6951525	Asns	0.689	0.704589178963299	-
6959457	Hnrnpl	0.689	0.704589178963299	-
6998743	Atrip	0.689	0.704589178963299	-
6782695	Pigs	0.69	0.705291581574767	-
6797383	Kcnk13	0.69	0.705291581574767	-
6851192	Ccdc94	0.69	0.705291581574767	-
6859837	Wdr36	0.69	0.705291581574767	-
6969735	Rps3	0.69	0.705291581574767	-
6970149	Tpp1	0.69	0.705291581574767	-
6798318	Tmem121	0.691	0.70615350877193	-
6820050	Pebp4	0.691	0.70615350877193	-
7014633	Rragb	0.691	0.70615350877193	+
6869918	Scd3	0.692	0.706961596613245	-
6942876	Spdyb	0.692	0.706961596613245	-
6962829	Prkrir	0.692	0.706961596613245	-
6964023	Ubfd1	0.692	0.706961596613245	-
6782031	Trp53	0.693	0.707822689139143	-
6856584	Pja2	0.693	0.707822689139143	-
6964053	Rbbp6	0.693	0.707822689139143	-
6788662	Wnt3a	0.694	0.708576307041402	-
6810716	Akr1c20	0.694	0.708576307041402	-
6849316	Sepx1	0.694	0.708576307041402	-
6871258	Prdx5	0.694	0.708576307041402	-
6976735	Tmem192	0.694	0.708576307041402	-
6764481	Hnrnpu	0.695	0.709222230612399	-
6800894	Snx6	0.695	0.709222230612399	-
6838397	Arf3	0.695	0.709222230612399	-
6849231	Srrm2	0.695	0.709222230612399	-
6880640	Pdia3	0.695	0.709222230612399	-
6881123	Slc20a1	0.695	0.709222230612399	+
6935059	Ache	0.695	0.709222230612399	-
6843861	Grin2a	0.697	0.711048388314335	-
6881870	Slc24a3	0.697	0.711048388314335	-
6949800	Phb2	0.697	0.711048388314335	-
6999348	Cmc1	0.697	0.711048388314335	-
6926330	2310028O11Rik	0.698	0.711907320754717	-

6995912	Mpi	0.698	0.711907320754717	-
7020641	Syap1	0.698	0.711907320754717	-
6798951	Vsnl1	0.699	0.712604556427278	-
6843681	Glyr1	0.699	0.712604556427278	-
6861639	Grp	0.699	0.712604556427278	-
6865971	Slc26a2	0.699	0.712604556427278	-
6884237	Col20a1	0.699	0.712604556427278	-
6978953	Cyb5b	0.699	0.712604556427278	-
6748701	Unc50	0.7	0.713301161212487	-
6756989	Lactb2	0.7	0.713301161212487	-
6776104	Metap2	0.7	0.713301161212487	-
6869509	Hhex	0.7	0.713301161212487	-
6899359	S100a13	0.7	0.713301161212487	-
6910642	Ak5	0.7	0.713301161212487	+
6951247	3010003L21Rik	0.701	0.714212454764777	-
7011264	Hprt	0.701	0.714212454764777	-
6789474	C1qbp	0.702	0.7150156768164	-
6809806	Sgtb	0.702	0.7150156768164	-
6989437	Ubl7	0.702	0.7150156768164	-
6991326	Zic4	0.702	0.7150156768164	-
6824146	5730469M10Rik	0.703	0.71587235325145	-
6868577	1500015L24Rik	0.703	0.71587235325145	-
6996942	Tmod3	0.703	0.71587235325145	-
6794496	Ankmy2	0.704	0.716674651600753	-
6855680	Tjap1	0.704	0.716674651600753	-
6924382	Gpx7	0.704	0.716674651600753	-
6984972	Tppp3	0.704	0.716674651600753	-
6775412	Gng7	0.705	0.717584544701363	-
6918127	Sdhb	0.705	0.717584544701363	-
6761256	Bcl2	0.706	0.718385964912281	-
6954613	Rnf181	0.706	0.718385964912281	-
6972203	Cend1	0.706	0.718385964912281	-
7018507	P2ry4	0.706	0.718385964912281	-
6833902	Slc1a3	0.707	0.719132771338251	-
6850534	Pla2g7	0.707	0.719132771338251	-
6917336	Ptp4a2	0.707	0.719132771338251	-
6933679	Rab35	0.707	0.719132771338251	-
7009783	Praf2	0.707	0.719132771338251	-
6756792	Vcpip1	0.708	0.719824857056876	-
6780907	Ppp2ca	0.708	0.719824857056876	-
6832094	4930483J18Rik	0.708	0.719824857056876	-
6866233	Lman1	0.708	0.719824857056876	-
6883073	Ywhab	0.708	0.719824857056876	-
6895836	E2f5	0.708	0.719824857056876	-
6797387	Psmc1	0.709	0.720787331678327	-
6779827	Nsg2	0.71	0.721532561287412	-
6780871	Sar1b	0.71	0.721532561287412	-
6788014	Sqstm1	0.71	0.721532561287412	-
6941215	Gltp	0.71	0.721532561287412	-
6984919	Dync1li2	0.71	0.721532561287412	-

6760387	Nppc	0.711	0.722277230699842	-
6819974	Nefl	0.711	0.722277230699842	-
6882706	Vstm2l	0.711	0.722277230699842	-
6934933	Rhbdd2	0.711	0.722277230699842	-
6988769	Apoa1	0.711	0.722277230699842	-
6782917	Ccl11	0.712	0.723130016533894	-
6917283	Yars	0.712	0.723130016533894	-
6973228	Syt5	0.712	0.723130016533894	-
6791015	Pdk2	0.713	0.724091230179605	-
6751709	Bok	0.714	0.724725552050473	-
6801691	Six4	0.714	0.724725552050473	-
6869547	Lgi1	0.714	0.724725552050473	-
6875727	Fbxw5	0.714	0.724725552050473	-
6893131	Kcnb1	0.714	0.724725552050473	-
6976923	Atp6v1b2	0.714	0.724725552050473	-
6992647	Dync1li1	0.714	0.724725552050473	-
6749069	Nck2	0.715	0.725631571042355	-
6911439	Sdcbp	0.715	0.725631571042355	-
6784062	Igfbp4	0.716	0.72648276897665	-
6803335	Tcl1	0.716	0.72648276897665	-
6880467	Disp2	0.716	0.72648276897665	-
6769141	Fgf22	0.717	0.727333583546014	-
6817984	Bap1	0.717	0.727333583546014	-
6854541	Nudt3	0.717	0.727333583546014	-
6978327	Arl2bp	0.718	0.72829332732868	-
6795451	Pnn	0.719	0.729198198874296	-
6886075	Lhx6	0.719	0.729198198874296	-
6789974	Tnfaip1	0.72	0.729993247805537	-
6790585	Dynll2	0.72	0.729993247805537	-
6796799	1810035L17Rik	0.72	0.729993247805537	-
6816222	Ndufs4	0.72	0.729993247805537	-
6789483	6330403K07Rik	0.721	0.730842634263426	-
6982918	Cpe	0.721	0.730842634263426	-
6994151	Vps26b	0.721	0.730842634263426	-
6876210	St6galnac6	0.722	0.731746512674366	-
6954935	Mrpl19	0.722	0.731746512674366	-
6911679	Trp53inp1	0.723	0.732650119976005	-
6962841	Wnt11	0.723	0.732650119976005	-
6846437	Tfg	0.724	0.733553456290298	-
6867565	Mrpl21	0.724	0.733553456290298	-
6804523	Akr1c14	0.725	0.73445652173913	-
6966996	Fgf21	0.725	0.73445652173913	-
6814387	Sdha	0.726	0.735414436698898	-
6764351	Fh1	0.727	0.736151506069234	-
6779838	Hba-x	0.727	0.736151506069234	-
6850937	Plcl2	0.727	0.736151506069234	-
6941202	Kctd10	0.727	0.736151506069234	-
6974200	Fbxo25	0.727	0.736151506069234	-
6944432	Naa38	0.728	0.737108863415	-
6977012	Fkbp8	0.729	0.738010787324893	-

7017489	Cd99l2	0.729	0.738010787324893	-
6764016	Uhmk1	0.73	0.738857100059916	-
6900090	Nras	0.73	0.738857100059916	-
6907115	Psmb4	0.73	0.738857100059916	-
6965381	Fgf4	0.731	0.739813824608702	-
6860256	Ndfip1	0.732	0.740714938225384	-
6908924	Myoz2	0.732	0.740714938225384	-
6770064	Dcn	0.733	0.741615781986973	-
6813322	4833439L19Rik	0.733	0.741615781986973	-
6841865	Cldnd1	0.734	0.742460778443114	-
6994266	Ntm	0.734	0.742460778443114	-
7018526	Snx12	0.734	0.742460778443114	-
6781104	Gm2a	0.735	0.743194163860831	-
6881787	Dstn	0.735	0.743194163860831	-
6945748	Pip	0.735	0.743194163860831	-
6946004	Atp6v0e2	0.735	0.743194163860831	-
6987084	Cwc15	0.735	0.743194163860831	-
6775148	Ddt	0.736	0.743927000747943	-
6777351	Slc35e3	0.736	0.743927000747943	-
6808876	Ssbp2	0.736	0.743927000747943	-
6813172	Shc3	0.736	0.743927000747943	-
6866852	Slc14a1	0.736	0.743927000747943	+
6799462	Id2	0.737	0.744714969343502	+
6914010	--	0.737	0.744714969343502	-
6936730	Tmub1	0.737	0.744714969343502	-
6978918	6030452D12Rik	0.737	0.744714969343502	-
6773096	Nkain2	0.738	0.745613935406699	-
6874215	Prdx3	0.738	0.745613935406699	-
6780940	9530068E07Rik	0.739	0.746401046337818	-
6856262	Mllt1	0.739	0.746401046337818	-
6976002	Dctd	0.739	0.746401046337818	-
6994704	Nrgn	0.739	0.746401046337818	-
6880583	Snap23	0.74	0.747299357345688	-
6965348	Dhcr7	0.74	0.747299357345688	-
6754867	Fam78b	0.741	0.748085611833259	-
6911703	1110037F02Rik	0.741	0.748085611833259	-
6950555	Strap	0.741	0.748085611833259	-
7012080	Fundc2	0.741	0.748085611833259	+
6902185	Lpar3	0.742	0.748983268598745	-
6999677	Lztfl1	0.742	0.748983268598745	-
6780920	Skp1a	0.743	0.749880657206871	-
6833430	Tarbp2	0.743	0.749880657206871	-
6763517	Zbtb37	0.744	0.750609630459127	-
6767336	Cdk19	0.744	0.750609630459127	-
6952601	Slc35b4	0.744	0.750609630459127	-
6954032	A530053G22Rik	0.744	0.750609630459127	-
6972491	Ccnd1	0.744	0.750609630459127	-
6855134	Tubb5	0.746	0.752515040680749	-
6855225	Mog	0.746	0.752515040680749	+
6840887	Igsf11	0.748	0.754419882080752	-

6998049	Atp1b3	0.748	0.754419882080752	-
6948378	Arl6ip5	0.749	0.755372089552239	-
6789977	Tmem97	0.75	0.755985679122846	-
6819250	Fitm1	0.75	0.755985679122846	-
6845079	Apod	0.75	0.755985679122846	-
6846000	Tagln3	0.75	0.755985679122846	-
6849044	Ppp2r1a	0.75	0.755985679122846	-
6917835	Luzp1	0.75	0.755985679122846	-
6941772	Morn3	0.75	0.755985679122846	-
6844904	Fgf12	0.751	0.756711452430659	-
6882406	Mapre1	0.751	0.756711452430659	-
6916218	Txndc12	0.751	0.756711452430659	-
6934308	Setd8	0.751	0.756711452430659	+
6953762	Scrn1	0.751	0.756711452430659	-
6798770	Gdf7	0.752	0.757549608647037	-
6806933	Ninj1	0.752	0.757549608647037	-
6896351	Nceh1	0.752	0.757549608647037	-
6777249	Kcnmb4	0.754	0.759337953647813	-
6811769	Acot13	0.754	0.759337953647813	+
6978274	Ogfod1	0.754	0.759337953647813	-
6995263	Pafah1b2	0.754	0.759337953647813	-
6768783	Zwint	0.755	0.760175085680227	-
6880040	2900064A13Rik	0.755	0.760175085680227	-
6880501	Gchfr	0.755	0.760175085680227	-
6750314	Vwc2l	0.756	0.761011843575419	-
6969800	Ppme1	0.756	0.761011843575419	-
6986056	Pgbd5	0.756	0.761011843575419	-
6747364	Pcmtd1	0.758	0.762854632112005	-
6855022	H2-Ea-ps	0.758	0.762854632112005	-
6890916	Avp	0.758	0.762854632112005	-
6846575	Arl6	0.759	0.763804155186537	-
6854159	Gm4769	0.761	0.765702777157323	-
6970381	Ascl3	0.761	0.765702777157323	-
6855725	Rpl7l1	0.762	0.766537740062528	-
6914765	D4Bwg0951e	0.762	0.766537740062528	-
6917591	1810019J16Rik	0.762	0.766537740062528	-
6949098	6720456B07Rik	0.763	0.767429443286693	-
7011907	Nsdhl	0.763	0.767429443286693	-
6827123	Kctd12	0.765	0.769269290869856	+
6878643	Zc3h15	0.765	0.769269290869856	-
6975307	Tmem66	0.765	0.769269290869856	-
6990017	Fam96a	0.766	0.77021755952381	-
6899308	4933434E20Rik	0.767	0.7711083172147	-
6942474	Srcrb4d	0.767	0.7711083172147	-
6814070	A530054K11Rik	0.768	0.771998809878012	-
7002924	Hsd17b14	0.768	0.771998809878012	-
6849287	Rnps1	0.769	0.772946522870956	-
6924691	Cmpk1	0.77	0.773836543466944	-
7018249	Eif2s3x	0.77	0.773836543466944	-
6771586	Rdh1	0.771	0.774611106980894	-

6844334	Comt1	0.771	0.774611106980894	-
6921286	Mcart1	0.771	0.774611106980894	-
7018773	Taf9b	0.771	0.774611106980894	-
6877895	Lass6	0.772	0.775558132619685	-
6836694	Slurp1	0.774	0.777451761557901	-
6963950	BC030336	0.774	0.777451761557901	-
6754798	Creg1	0.775	0.778224847674246	-
6850723	Mea1	0.775	0.778224847674246	-
6911530	Rab2a	0.775	0.778224847674246	-
6996603	Foxb1	0.775	0.778224847674246	-
6788147	Ube2b	0.776	0.779055345070946	-
6816703	Abhd6	0.776	0.779055345070946	-
6925659	Zcchc17	0.776	0.779055345070946	-
6850705	BC048355	0.777	0.77994340043081	-
6904262	Dcun1d1	0.777	0.77994340043081	-
6881172	Nop56	0.778	0.780889185977421	-
6753394	Phlda3	0.779	0.781776771127283	-
6924802	Akr1a4	0.779	0.781776771127283	-
6764046	Pcp4l1	0.782	0.784612666122207	-
6838382	2310037I24Rik	0.782	0.784612666122207	-
6869212	Minpp1	0.782	0.784612666122207	-
6799849	Bcap29	0.783	0.785557683741648	-
6796768	2310044G17Rik	0.785	0.78750575309925	-
6860967	Csnk1g3	0.786	0.78845041567696	-
6935613	Pomp	0.787	0.789336351491762	-
6964571	Hmx2	0.787	0.789336351491762	-
6792392	Cd300e	0.789	0.79116613489649	-
6890584	Myef2	0.789	0.79116613489649	-
7015419	Pqbp1	0.789	0.79116613489649	-
6793639	Ntsr2	0.79	0.792110105356878	-
6892143	Nrsn2	0.791	0.79305393575191	-
6855526	Enpp4	0.793	0.795000148367953	-
6756419	Kcnh1	0.794	0.79588458685655	-
6864497	Reep5	0.794	0.79588458685655	-
6869801	Golga7b	0.795	0.796768762978345	-
6985984	Spata2L	0.795	0.796768762978345	-
6830641	Fam83a	0.796	0.797711827956989	-
6873179	Sfrp5	0.797	0.798654753077265	-
6785777	Tmed4	0.798	0.799597538370283	-
6987109	Gpr83	0.799	0.800540183867141	-
6980952	AI316807	0.801	0.802484542960931	-
6840103	Eif4a2	0.803	0.804368986731895	-
6890125	Srp14	0.803	0.804368986731895	-
6926165	C1qb	0.804	0.805251315496924	-
7012006	Atp6ap1	0.804	0.805251315496924	-
6852849	Cript	0.81	0.811200533570476	-
6983686	Smarca5	0.812	0.813143238236384	-
6803527	Setd3	0.814	0.815085655008892	-
6792486	H3f3b	0.819	0.82003156256946	+
6995853	Odf3l1	0.824	0.824976737294414	-

7020242	Tspyl2	0.826	0.826917845766353	-
6950160	Gabarapl1	0.83	0.830860740740741	-
7014155	Prps1	0.832	0.83280112584253	-
7010657	Pgrmc1	0.834	0.834741223522441	-
6762420	Timm17a	0.842	0.842623518957346	-
6775502	B230315N10Rik	0.842	0.842623518957346	-
6933791	Wsb2	0.843	0.843561791928915	-
6934186	A930024E05Rik	0.845	0.845500518288168	-
6774784	A930033H14Rik	0.853	0.853442067076331	-
6832191	1500032L24Rik	0.857	0.857380663310631	-
6757974	6330578E17Rik	0.858	0.858317566067066	-
6785617	Tug1	0.861	0.861254922279793	-
6930485	Med28	0.866	0.866192287765524	-
7014557	Hsd17b10	0.887	0.887131290704559	-
6843500	Pcp4	0.905	0.905066972544957	-
6852144	Lbh	0.916	0.916	+
