# Supplementary material for: Neuronal activity regulates alternative exon usage
Source: Mol Brain. 2020 Nov 10;13:148. doi: 10.1186/s13041-020-00685-3 (PMC7656758; doi:10.1186/s13041-020-00685-3)
Supplement: Supplementary file 4 — Additional file 4: Genes with activity-dependent exon usage identified through the FIRMA method. pdf. FIRMA (F) scores represent the discrepancy of one probeset from the other probesets in the same gene and were calculated for every probeset and sample. fSscore, corresponds to samples collected after seizure compared to controls and samples. f0 score, corresponds to samples collected after vehicle treatment compared to controls. FDR, False discovery Rate calculated from F scores for the alternative and the null model. DE, differentially expressed, indicates whether the whole gene was identified as induced by neuronal activity in our initial analysis (Additional file 1). [file 13041_2020_685_MOESM4_ESM.pdf]

Genes with activity-dependent exon usage identified through the FIRMA method.

geneName	probesetID	fSscore	f0score	FDR	DE
Rcan1	4567413	444.582.794.189.453	0.344759613275528	0	-
Dclk1	5144145	968.378.067.016.602	0.52497011423111	0	-
Mt1	5480425	709.025.955.200.195	0.621032059192657	0	+
Vmp1	4780502	501.396.141.052.246	0.54198831319809	0	-
Ntf3	5239597	148.107.719.421.387	0.370917677879333	0.0666666666666667	+
Rheb	5489435	151.304.006.576.538	0.404361933469772	0.0714285714285714	+
Grik2	5235346	15.519.868.850.708	121.967.780.590.057	0.0769230769230769	+
Car2	4363942	16.081.901.550.293	0.346997320652008	0.0833333333333333	-
Homer1	5397705	161.636.638.641.357	0.581226289272308	0.0909090909090909	+
Nav2	4655500	169.553.165.435.791	0.84081643819809	0.1	-
Cyfip2	5021796	134.137.477.874.756	0.140903875231743	0.105263157894737	-
Hmgcs1	5180606	19.122.501.373.291	0.893704950809479	0.111111111111111	+
Kcnip4	5157280	139.248.809.814.453	0.671719372272491	0.111111111111111	-
Kif1b	5125179	144.353.475.570.679	0.483153700828552	0.117647058823529	-
Lats2	5106561	197.682.247.161.865	0.256945759057999	0.125	-
Chn1	4855558	146.951.456.069.946	0.109870821237564	0.125	-
Sertm1	5573324	204.109.687.805.176	0.747392535209656	0.142857142857143	+
Hcn1	5022633	124.642.887.115.479	364.745.879.173.279	0.142857142857143	-
Spred1	5596841	129.366.073.608.398	0.323668390512466	0.15	-
Mpv17l	4759141	113.677.825.927.734	0.328563868999481	0.153846153846154	+
Epb4.1l4b	4830765	941.250.801.086.426	0.257142871618271	0.15625	-
Homer1	5099689	115.591.611.862.183	0.586897134780884	0.16	+
Abr	5471089	957.511.806.488.037	0.305998474359512	0.161290322580645	-
Dclk1	4895553	343.155.784.606.934	0.723257184028625	0.166666666666667	-
Rbl2	5447102	116.679.000.854.492	0.44045552611351	0.166666666666667	-
Rasgrf1	4535438	959.745.693.206.787	0.307280302047729	0.166666666666667	-
Col27a1	4828451	971.152.400.970.459	10.505.176.782.608	0.172413793103448	-
Lats2	5283052	116.751.956.939.697	0.94549959897995	0.173913043478261	-
Golga2	5189440	103.900.804.519.653	0.914161443710327	0.178571428571429	-
Adam15	5437318	121.897.230.148.315	0.164070054888725	0.181818181818182	-
R3hdm1	4953035	107.233.848.571.777	199.737.441.539.764	0.185185185185185	+
Raver2	4749916	742.785.978.317.261	0.308557778596878	0.195652173913043	+
Homer1	5049858	347.550.849.914.551	0.491658627986908	0.2	+
Prkcb	5164882	7.503.005.027.771	0.946676075458527	0.2	-
Map7	5295094	75.383.186.340.332	159.370.636.940.002	0.204545454545455	-
Slc36a1	4451683	755.315.065.383.911	0.782684862613678	0.209302325581395	-
Errfi1	4605188	888.826.656.341.553	100.439.953.804.016	0.212121212121212	+
Pde1a	4644192	727.731.657.028.198	0.429972976446152	0.212765957446808	-
Dnm1l	5481824	598.207.855.224.609	0.180213034152985	0.213114754098361	-
Kif1b	4738866	762.454.509.735.107	0.314740478992462	0.214285714285714	-
Arid5a	5454723	648.310.804.367.065	0.622977614402771	0.214285714285714	+
Prrc2a	4832191	691.376.972.198.486	0.581727564334869	0.215686274509804	-
Homer1	5231202	836.506.271.362.305	0.863182902336121	0.216216216216216	+
Jak1	5055286	598.247.337.341.309	227.634.310.722.351	0.216666666666667	-
Gna12	5412254	666.432.523.727.417	0.824824154376984	0.218181818181818	-
Erc1	4991499	77.222.695.350.647	0.543177962303162	0.219512195121951	+
Ankrd13c	4737063	691.872.549.057.007	0.766865611076355	0.22	-
Ints8	4373869	610.299.396.514.893	0.701506912708282	0.220338983050847	-

Ptgds	4408859	851.396.465.301.514	0.346206158399582	0.222222222222222	-
Errfi1	4558221	669.861.793.518.066	114.717.817.306.519	0.222222222222222	+
Msmo1	5104399	633.639.574.050.903	0.671354174613953	0.224137931034483	+
Plcg1	5491235	713.136.863.708.496	0.390589207410812	0.224489795918367	-
Vmp1	4782304	796.803.760.528.564	0.643729090690613	0.225	-
Egr1	4633715	597.258.806.228.638	0.727976143360138	0.225806451612903	+
Prosc	5415115	674.414.014.816.284	0.714691877365112	0.226415094339623	+
Dtna	4919815	634.728.813.171.387	0.545904517173767	0.228070175438597	-
Rab6a	5590127	85.945.463.180.542	0.371126651763916	0.228571428571429	-
Homer1	4889194	724.265.718.460.083	0.527361512184143	0.229166666666667	+
Mbnl2	4849027	677.890.968.322.754	0.887720346450806	0.230769230769231	-
Nfat5	4979992	801.226.997.375.488	0.798625409603119	0.230769230769231	-
Erc1	5195361	870.497.417.449.951	0.830735862255096	0.235294117647059	+
Col27a1	5159640	812.429.428.100.586	0.569792568683624	0.236842105263158	-
Sep9	4348550	592.576.694.488.525	0.594299077987671	0.238095238095238	-
Kif1a	5323399	581.170.797.348.022	0.24662820994854	0.238805970149254	-
Kif5a	4525678	581.981.372.833.252	30.936.758.518.219	0.242424242424242	-
Sep5	5200839	584.917.831.420.898	0.971312403678894	0.246153846153846	-
Uhrf2	4384547	588.790.464.401.245	121.725.928.783.417	0.25	-
Btf3	4579726	575.825.357.437.134	0.771148264408112	0.25	-
Zfp318	5593701	567.770.481.109.619	0.510933876037598	0.257142857142857	-
Slc24a4	4778380	56.961.464.881.897	0.81304520368576	0.260869565217391	+
Ddx5	4323262	555.592.060.089.111	0.332314521074295	0.267605633802817	+
Capza2	4902630	547.597.551.345.825	0.793530464172363	0.277777777777778	-
Dgkz	5165507	532.782.745.361.328	0.302279770374298	0.283783783783784	-
Ranbp2	4634020	521.471.786.499.023	0.310932904481888	0.2875	+
Tesc	5371879	537.940.454.483.032	0.223368436098099	0.287671232876712	-
Fgfr1	5228863	522.677.516.937.256	11.907.787.322.998	0.291139240506329	-
Fth1	4414186	51.222.448.348.999	0.566493093967438	0.292682926829268	-
Nt5c	4970708	531.786.346.435.547	0.489939600229263	0.293333333333333	-
Grik2	4412758	524.970.245.361.328	107.270.109.653.473	0.294871794871795	+
Mast3	5047905	513.801.383.972.168	0.568446576595306	0.296296296296296	-
Psd3	5060935	509.692.907.333.374	0.784289300441742	0.297619047619048	-
Runx2	5290904	526.287.698.745.728	0.634175002574921	0.298701298701299	-
Mpc2	4704910	508.220.052.719.116	0.43385249376297	0.298850574712644	-
Tnfrsf12a	5104152	509.967.565.536.499	0.54179322719574	0.301204819277108	-
Nudt9	5099848	508.297.872.543.335	0.829665064811707	0.302325581395349	-
Dnajb5	5601267	528.030.395.507.812	0.414490103721619	0.302631578947368	+
Cyfip2	4834997	509.381.198.883.057	0.283698052167892	0.305882352941176	-
Parp6	4643370	504.007.863.998.413	504.007.863.998.413	0.329545454545455	-
Egr1	5344741	471.339.654.922.485	0.873186409473419	0.356435643564356	+
Nr4a1	5597794	492.761.659.622.192	0.550353467464447	0.359550561797753	+
Map2	4785961	47.138.934.135.437	0.262425810098648	0.36	-
R3hdm1	5156487	484.483.051.300.049	0.449324607849121	0.361702127659574	+
Kat6b	4652241	48.800.687.789.917	0.712228536605835	0.362637362637363	-
Syn2	4321117	471.826.171.875	0.437313288450241	0.363636363636364	-
Baiap2	4625866	485.954.141.616.821	0.824180722236633	0.365591397849462	-
Arpp21	4819053	488.416.385.650.635	0.46011084318161	0.366666666666667	-
Ivns1abp	4783731	476.064.157.485.962	0.5750892162323	0.36734693877551	+
Cdk14	4916982	487.121.868.133.545	0.532153189182281	0.369565217391304	+

Brinp1	4608671	430.780.363.082.886	0.562350392341614	0.370689655172414	+
Cacna2d1	4589487	476.104.402.542.114	0.718649089336395	0.371134020618557	-
Csnk2a2	5485429	431.308.078.765.869	0.700520217418671	0.373913043478261	-
Mical2	5486707	477.188.444.137.573	104.855.036.735.535	0.375	-
Arl14ep	4792483	429.749.202.728.271	0.999771773815155	0.376068376068376	-
Fezf2	5090183	431.515.216.827.393	0.418081402778625	0.37719298245614	+
Poli	4518491	480.732.297.897.339	0.460026562213898	0.378947368421053	-
Serinc5	4739371	432.925.939.559.937	131.651.282.310.486	0.380530973451327	-
Klc1	5367115	452.605.152.130.127	0.756637871265411	0.380952380952381	-
Arpp21	4780785	462.135.219.573.975	0.507557094097137	0.382352941176471	-
Trappc13	4757210	446.702.098.846.436	0.172503501176834	0.383177570093458	-
Pde1b	4448691	424.253.845.214.844	0.853988409042358	0.383333333333333	-
Arhgef12	4635406	433.928.966.522.217	0.695422947406769	0.383928571428571	-
Psd3	5147321	453.851.890.563.965	0.759846270084381	0.384615384615385	-
Drd4	5128943	440.777.063.369.751	0.681343734264374	0.385321100917431	+
Olfml2b	4414832	425.536.060.333.252	0.741023778915405	0.386554621848739	+
Acadm	5283374	451.009.845.733.643	0.89716625213623	0.386792452830189	-
Gspt1	4313074	436.560.487.747.192	0.780334949493408	0.387387387387387	-
Tsc22d1	5551896	454.256.868.362.427	0.385905206203461	0.388349514563107	-
Sh3glb2	4682439	44.232.029.914.856	0.48603543639183	0.388888888888889	-
Xiap	4556038	428.335.332.870.483	0.539761960506439	0.389830508474576	-
Papolg	4688963	437.589.311.599.731	10.430.029.630.661	0.390909090909091	-
Mkl2	5475988	417.629.098.892.212	0.764750361442566	0.40495867768595	-
Mapk1	4891855	412.194.299.697.876	0.211011856794357	0.422764227642276	-
Col27a1	5242268	412.468.194.961.548	0.558513820171356	0.426229508196721	-
Zfp318	5069922	411.067.676.544.189	0.575087428092957	0.42741935483871	-
Myl12b	4339917	381.223.344.802.856	137.813.007.831.573	0.428571428571429	-
Rasgrf1	4626770	378.076.171.875	0.136711224913597	0.431137724550898	-
Abhd17c	5114789	381.303.906.440.735	0.662821590900421	0.43125	-
Cygb	5484185	377.306.175.231.934	112.671.661.376.953	0.431952662721893	+
Crbn	5099556	375.335.359.573.364	0.93275386095047	0.433526011560694	-
Nr4a2	4719027	378.142.428.398.132	110.800.099.372.864	0.433734939759036	+
Nedd4l	4836449	381.380.891.799.927	0.516821384429932	0.433962264150943	-
Nfia	4593139	377.692.317.962.646	0.619429707527161	0.43452380952381	-
Arhgap12	4926509	375.500.559.806.824	0.535576403141022	0.436046511627907	+
Slc50a1	5361942	378.261.256.217.957	0.992694020271301	0.436363636363636	-
Cdk14	5339288	382.124.423.980.713	0.65151172876358	0.436708860759494	+
Gigyf2	4668976	374.148.392.677.307	0.655776143074036	0.436781609195402	-
Shisa5	4368572	378.755.187.988.281	0.535433948040009	0.438271604938272	-
Olfm1	5533311	375.644.063.949.585	0.822599649429321	0.43859649122807	-
Ints6	4361337	378.279.852.867.126	0.857430696487427	0.439024390243902	+
Pdcd4	5375380	383.753.848.075.867	0.613311290740967	0.439490445859873	+
Tcf25	4474575	40.786.075.592.041	0.91341632604599	0.44	-
Bdnf	5325572	375.645.089.149.475	0.652423202991486	0.441176470588235	+
Slc35b1	4912253	378.346.920.013.428	0.265401631593704	0.441717791411043	-
Abhd12	4449101	384.531.474.113.464	0.494602262973785	0.442307692307692	-
Crtac1	4336183	373.528.170.585.632	0.670258462429047	0.443181818181818	+
Ankrd13c	5010119	38.644.323.348.999	0.750117182731628	0.444444444444444	-
Nudt9	4383390	384.767.007.827.759	0.688529491424561	0.445161290322581	-
Reps2	4598343	373.685.097.694.397	117.249.250.411.987	0.445714285714286	-

Ap3m1	4385199	387.350.654.602.051	0.646839678287506	0.447368421052632	-
Mkrn1	4749056	385.059.356.689.453	0.864354729652405	0.448051948051948	-
Phf11a	5327416	372.305.512.428.284	0.807568311691284	0.449438202247191	-
Gpr12	5201634	388.482.236.862.183	0.797658741474152	0.449664429530201	+
Slc6a11	5015234	387.460.136.413.574	0.538369655609131	0.450331125827815	-
Rasgrf1	4640486	367.052.841.186.523	0.34768995642662	0.450549450549451	-
Nufip1	4967144	365.181.994.438.171	0.765884935855865	0.451086956521739	-
Htra1	5370968	372.431.087.493.896	372.431.087.493.896	0.451977401129943	-
Syne1	5212348	371.443.605.422.974	103.719.115.257.263	0.452513966480447	-
Lta4h	4499930	389.852.929.115.295	0.447315633296967	0.452702702702703	-
Mfap3	4342030	367.819.237.709.045	0.556497097015381	0.453038674033149	-
Ptov1	4683762	387.593.603.134.155	0.271024614572525	0.453333333333333	-
Atp1a3	5385869	366.925.072.669.983	0.874972224235535	0.453551912568306	-
Smyd3	4605639	39.317.262.172.699	0.650407552719116	0.453900709219858	-
Flywch1	4905464	342.537.045.478.821	0.386619985103607	0.455357142857143	-
Ndel1	4611908	369.829.273.223.877	0.472517997026443	0.455555555555556	+
Cds2	5537685	390.294.122.695.923	0.250863671302795	0.45578231292517	-
Tmx2	4400468	3.936.518.907.547	0.64613151550293	0.457142857142857	-
Tmod2	4341567	402.539.491.653.442	0.527269005775452	0.457364341085271	-
Col4a1	5125333	34.292.266.368.866	118.974.208.831.787	0.457399103139013	+
Slc25a11	5118117	341.781.258.583.069	136.104.226.112.366	0.457777777777778	-
Mapre3	5135577	401.011.991.500.854	0.917632818222046	0.458015267175573	-
Cda	4989372	391.379.523.277.283	0.782387733459473	0.458333333333333	+
Pak3	4600790	390.616.774.559.021	0.161671072244644	0.458904109589041	-
Calb1	4998914	342.927.551.269.531	0.687267899513245	0.459459459459459	+
Rbfox1	5373397	397.861.123.085.022	0.73223751783371	0.45985401459854	-
Atp13a1	5237776	394.617.366.790.771	0.791016221046448	0.460431654676259	-
Ndel1	5264936	403.267.240.524.292	0.820062696933746	0.4609375	+
Ptprn	4578524	401.310.539.245.605	0.808985233306885	0.461538461538462	-
Faf1	5070777	391.796.827.316.284	111.833.715.438.843	0.461538461538462	-
Dock3	5391390	342.977.142.333.984	0.636410117149353	0.461538461538462	+
Egr1	4902102	390.855.145.454.407	0.661017894744873	0.462068965517241	+
Rsbn1	4644086	363.474.202.156.067	0.680656790733337	0.462365591397849	-
Col27a1	4961871	399.865.984.916.687	0.664934515953064	0.462686567164179	-
Bola1	5161234	361.389.803.886.414	0.622151970863342	0.463157894736842	-
Mgll	5292727	398.310.279.846.191	0.646293342113495	0.463235294117647	-
Tsc22d1	5306568	360.636.281.967.163	0.544821858406067	0.463541666666667	-
Rasgrf1	5038873	343.193.411.827.087	0.322417438030243	0.463636363636364	-
Prdm8	4775568	394.944.643.974.304	0.807953476905823	0.463768115942029	+
Arpp21	4757464	404.177.570.343.018	0.496552526950836	0.464566929133858	-
Setd8	4443806	39.201.226.234.436	250.906.658.172.607	0.464788732394366	+
Ppargc1a	5420393	363.593.816.757.202	0.827559173107147	0.464864864864865	-
Dgcr2	5015151	362.012.577.056.885	0.908524751663208	0.465608465608466	-
Eif3a	5062418	34.334.704.875.946	0.670114874839783	0.465753424657534	-
Sf3b1	5163733	360.858.511.924.744	360.858.511.924.744	0.465968586387435	-
Csnk2b	5610861	400.082.921.981.812	0.29217529296875	0.466165413533835	-
Elk3	4511354	358.354.210.853.577	0.68811696767807	0.466321243523316	-
Syn1	5150087	357.049.250.602.722	0.716859936714172	0.466666666666667	-
Usp2	5333440	398.530.793.190.002	0.520991444587708	0.466666666666667	+
Acsl3	5568851	343.352.723.121.643	0.829928755760193	0.467889908256881	-

Homer1	4725497	362.321.066.856.384	0.40749853849411	0.468085106382979	+
Morf4l1	4321486	404.224.252.700.806	125.675.976.276.398	0.468253968253968	-
Lphn3	5301240	341.227.054.595.947	10.117.654.800.415	0.469026548672566	+
Fam107a	4667975	357.318.735.122.681	0.573965072631836	0.469072164948454	-
Rph3a	5598023	400.394.296.646.118	0.329240798950195	0.46969696969697	-
Ankrd13c	5385942	339.312.672.615.051	0.656331777572632	0.469827586206897	-
Prelp	5198699	353.058.576.583.862	0.825815498828888	0.47	-
Cdk11b	4904061	344.116.568.565.369	0.691078782081604	0.470046082949309	+
Erc1	5339426	352.710.866.928.101	0.607132315635681	0.47029702970297	+
Tro	4638069	362.501.239.776.611	0.637295365333557	0.470588235294118	-
Pcdhac1	5076863	340.864.872.932.434	0.287958115339279	0.47136563876652	-
Glp1r	4901893	339.702.320.098.877	0.471303522586823	0.471861471861472	-
Dgkb	4727679	345.090.079.307.556	0.497616142034531	0.472222222222222	-
Ccnk	5236496	353.084.683.418.274	0.478174924850464	0.472361809045226	+
Fam220a	5146409	352.897.071.838.379	0.569362282752991	0.472636815920398	-
Tmem160	5024430	336.654.758.453.369	0.985447764396667	0.473029045643154	-
Top3b	4371036	336.189.937.591.553	0.684044003486633	0.473251028806584	-
Tub	5053018	349.909.639.358.521	0.774954855442047	0.473429951690821	-
Nbn	4985548	339.726.972.579.956	0.551075220108032	0.473913043478261	-
Zmym2	5416364	337.687.110.900.879	0.843580961227417	0.474358974358974	-
Kcnab2	4814113	345.207.524.299.622	0.445600897073746	0.474418604651163	-
Nf1	5339528	353.428.483.009.338	0.364236265420914	0.474747474747475	-
Rab36	4885679	336.667.513.847.351	0.560925543308258	0.475	-
Frmd6	5220411	336.197.948.455.811	0.909384727478027	0.475206611570248	+
Mbd2	5192205	350.401.592.254.639	151.151.669.025.421	0.475728155339806	-
Gabrg2	5608111	339.934.134.483.337	0.826101958751678	0.475982532751092	-
Rgl1	4919569	337.745.404.243.469	0.829007148742676	0.476394849785408	-
Zbtb3	4361528	346.026.730.537.415	0.778062880039215	0.476635514018692	-
Cntn2	4373088	336.697.554.588.318	0.767853975296021	0.476987447698745	-
Erc1	4913667	353.651.356.697.083	0.956008911132812	0.477157360406091	+
Lrrc59	5165530	350.523.567.199.707	0.61638468503952	0.478048780487805	-
Nfkbiz	5125302	340.444.660.186.768	0.695339262485504	0.478070175438597	+
Apbb1	5391818	329.228.687.286.377	0.559107661247253	0.47843137254902	-
Gabra2	5335661	347.117.972.373.962	0.701231479644775	0.478468899521531	-
Arcn1	5602508	346.158.337.593.079	0.816297769546509	0.47887323943662	-
Cecr6	5157526	336.803.579.330.444	11.737.722.158.432	0.478991596638655	-
Epha10	4367159	353.992.033.004.761	0.474608451128006	0.479591836734694	+
Pim3	4822432	329.228.973.388.672	0.633493602275848	0.480314960629921	+
Ptprn	4429051	35.094.428.062.439	0.502884685993195	0.480392156862745	-
Polr2a	4978159	329.144.239.425.659	329.144.239.425.659	0.48046875	-
Hbegf	5346601	347.463.297.843.933	0.787609100341797	0.480769230769231	+
C1ql2	4555494	337.405.562.400.818	0.700283646583557	0.480851063829787	-
4932438A13Rik	5532936	347.033.023.834.229	0.702878594398499	0.480952380952381	-
Timp3	4526081	337.093.615.531.921	0.804566860198975	0.481012658227848	-
Bmper	4733381	346.413.946.151.733	145.904.588.699.341	0.481132075471698	-
Tpp2	4471432	329.598.832.130.432	0.685061156749725	0.482213438735178	-
Wdr3	4671213	351.852.607.727.051	120.687.294.006.348	0.482758620689655	-
Abr	5613928	337.140.655.517.578	287.385.296.821.594	0.483050847457627	-
Ranbp2	4541074	346.467.471.122.742	0.70392382144928	0.483412322274882	+
Vmp1	5460685	329.767.751.693.726	0.656900763511658	0.484126984126984	-
