# Supplementary figures and images for: Neuronal activity regulates alternative exon usage
Source: Mol Brain. 2020 Nov 10;13:148. doi: 10.1186/s13041-020-00685-3 (PMC7656758; doi:10.1186/s13041-020-00685-3)

# Krt75

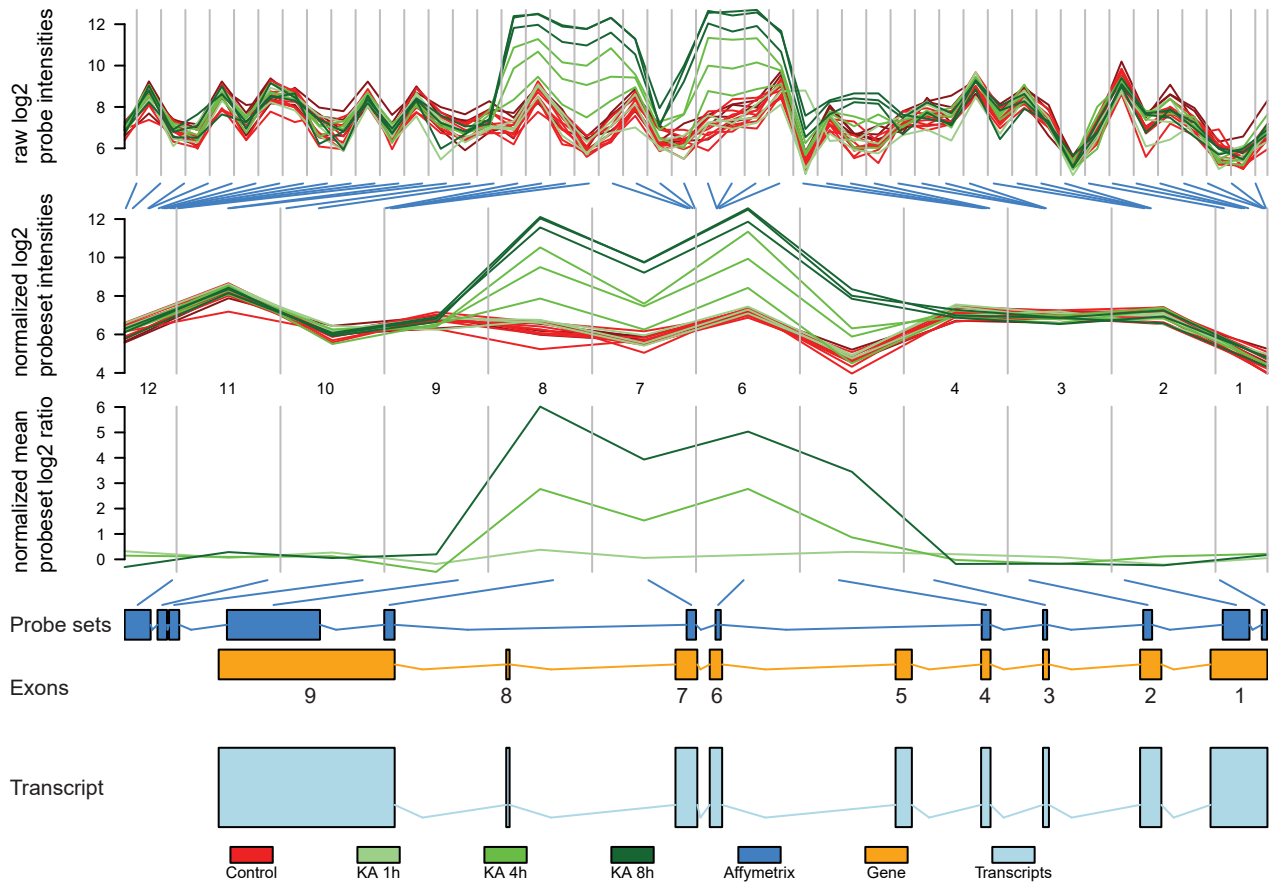

Supplement: Supplementary file 5 — Additional file 5: Gene expression profile of Krt75. pdf. Analysis and visualization of the microarray data obtained for Krt75. The upper 3 plots show the log2 transformed expression values of Krt75 over the course of its probes and probesets per exon. While the red lines show the intensity values of the control samples (vehicle and untreated), the 3 shades of green represent the expression intensities in the samples at the three different time points after seizures. The upper plot shows raw probe level intensities and grey bins stand for the individual probes. The middle plot shows the normalized intensities summarized to probeset level (probes divided by grey bins) for every sample. The lower plot shows the log2 ratio calculated from the mean of the intensities seen in the second plot, each line represents one of the 3 time points after seizure. At the bottom, 3 types of exon models are depicted. The dark blue boxes represent the probesets per exons, which were included in the microarrays. The yellow boxes and track correspond to genomic sequence and each box indicates an exon. The light blue tracks and boxes represent the only so far annotated transcript present in the Ensembl database. Note that exons 5 and 8 are not represented by probesets. [file 13041_2020_685_MOESM5_ESM.pdf]

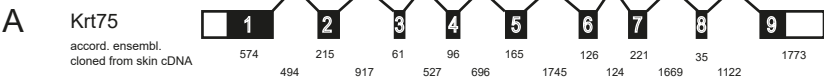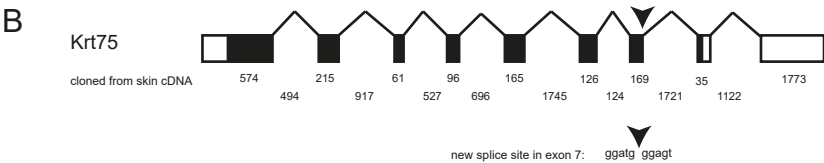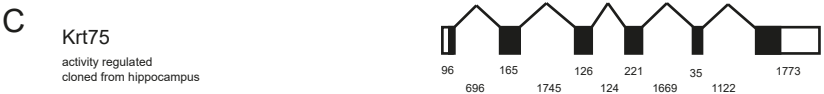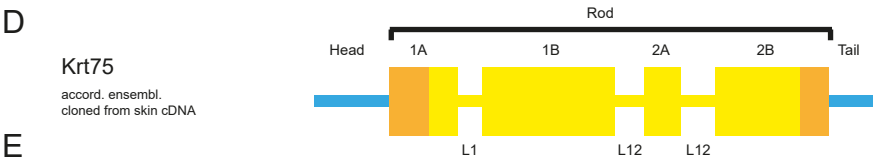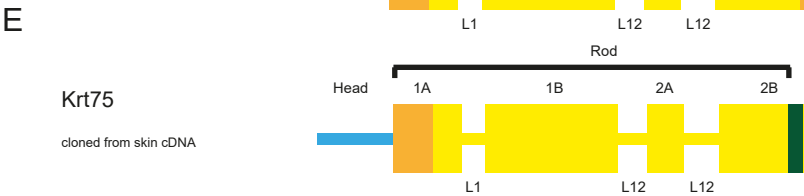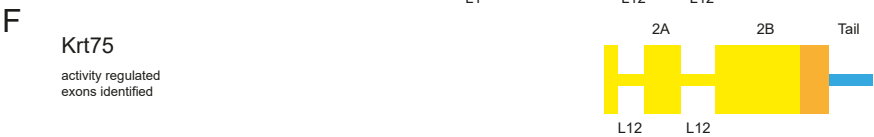

Supplement: Supplementary file 8 — Additional file 8: Alternative splice variants of Krt75. pdf. a Exons of murine Krt75 according to the Ensembl database. Boxes represent exons (not to scale), black boxes correspond to coding sequence, white boxes to untranslated regions. Numbers indicate exon size (upper row) and intron size (lower row). A corresponding cDNA was cloned from skin in this study (Genebank accession number NM_133357). b An alternatively spliced and previously undescribed cDNA was cloned in this study from skin (Genebank accession number MN037882). The internal alternative splice site (arrowhead) in exon 7 is indicated. The usage of this alternative splice site results in a frameshift and premature stop in the sequence corresponding to exon 8. c A truncated splice variant was cloned in this study from hippocampus of mice 4 h after seizure onset (Genebank accession number MN124092). It starts with exon 4. d Schematic of the protein domain structure of Krt75 corresponding to the canonical variant depicted in a. 1A, 1B, 2A and 2B are coiled-coil subdomains and L1, L12 and L2 are intervening linkers. The orange box indicates 10–20 amino acid segments that are highly conserved among keratin and other interfilament proteins. e Schematic of the protein domain structure of Krt75 corresponding to the new splice variant from skin depicted in b. f Schematic of the protein domain structure of Krt75 corresponding to the activity-regulated splice variant expressed in the hippocampus depicted in c. [file 13041_2020_685_MOESM8_ESM.pdf]

# Rcan1

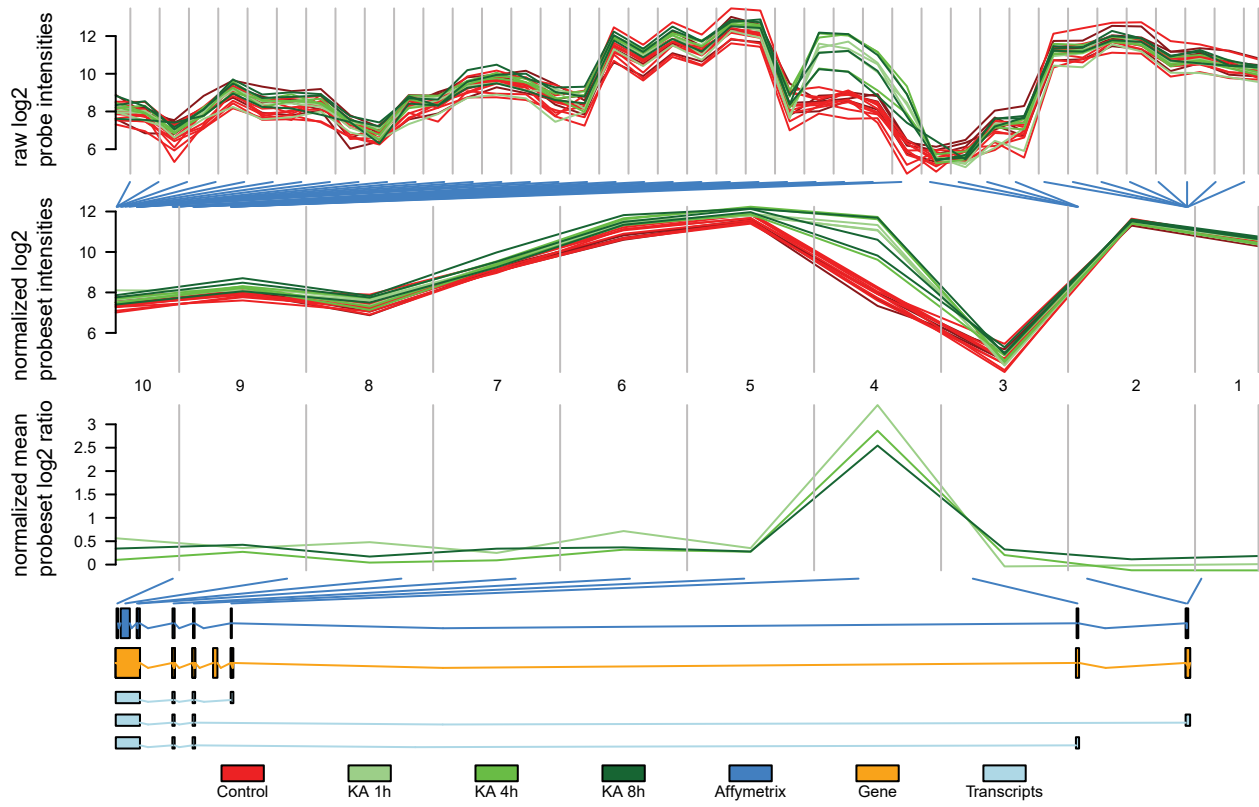

Supplement: Supplementary file 9 — Additional file 9: Gene expression profile of Rcan1. pdf. Analysis and visualization of the microarray data obtained for Rcan1. The upper 3 plots show the log2 transformed expression values of Rcan1 over the course of its probes and probesets per exon. While the red lines show the intensity values of the control samples (vehicle and untreated), the 3 shades of green represent the expression intensities in the samples at the three different time points after seizures. The upper plot shows raw probe level intensities and grey bins stand for the individual probes. The middle plot shows the normalized intensities summarized to probeset level (probes divided by grey bins) for every sample. The lower plot shows the log2 ratio calculated from the mean of the intensities seen in the second plot, each line represents one of the 3 time points after seizure. At the bottom 3 types of exon models are depicted. The dark blue boxes represent the probesets per exons, which were included in the microarray analysis. The yellow boxes and track correspond to genomic sequence and each box indicates an exon. The light blue tracks and boxes represent the annotated transcripts present in the Ensembl database. [file 13041_2020_685_MOESM9_ESM.pdf]

A

Rcan1

[0 - 1436]

Cntrl

3h

B

Cntrl

3h

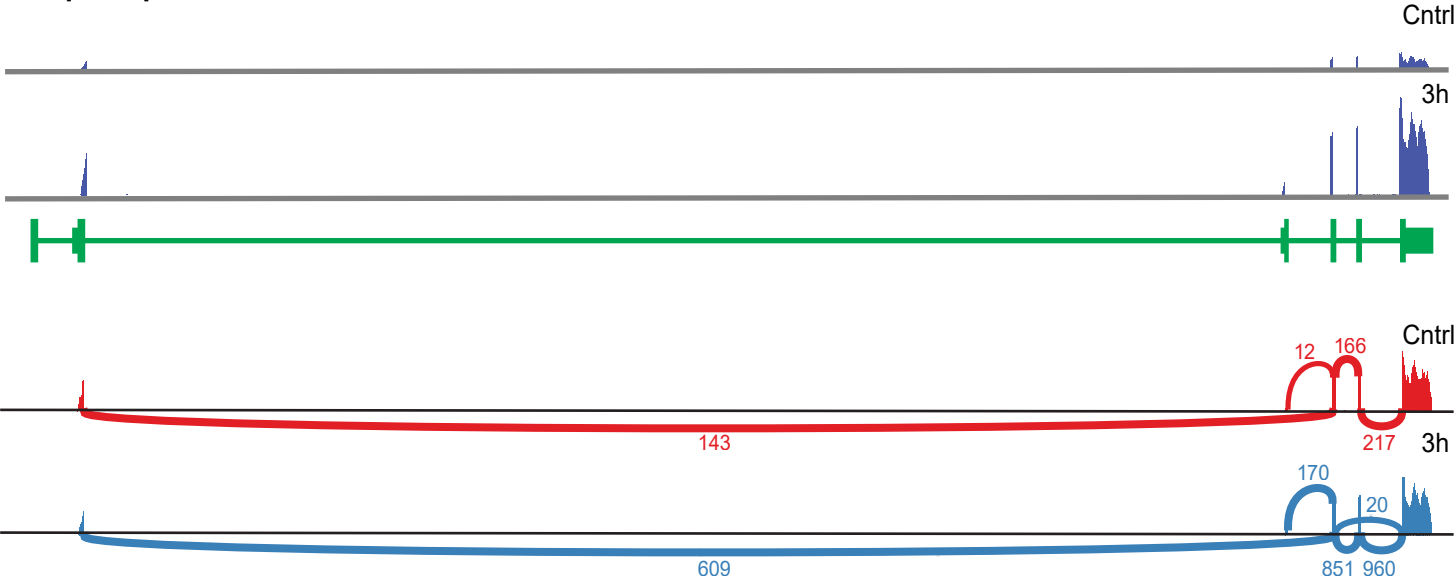

Supplement: Supplementary file 10 — Additional file 10: Activity-dependent splicing of Rcan1 in primary cultured hippocampal neurons. pdf. a Coverage plots (blue) from paired-end reads of RNA from untreated primary hippocampal neurons (upper panel) and 3 h after KCl induced depolarization (lower panel). The reference gene track is depicted below (green). b Sashimi plot of RNA sequencing data from untreated primary neurons (upper panel) or 3 h after KCl treatment (lower panel). Numbers indicate the counts of RNA sequencing reads that span the respective exon junctions. RNA-seq data was generated by Quesnel et al. and deposited in the GEO database under ID code GEO: GSE89984. [file 13041_2020_685_MOESM10_ESM.pdf]

A

Cda

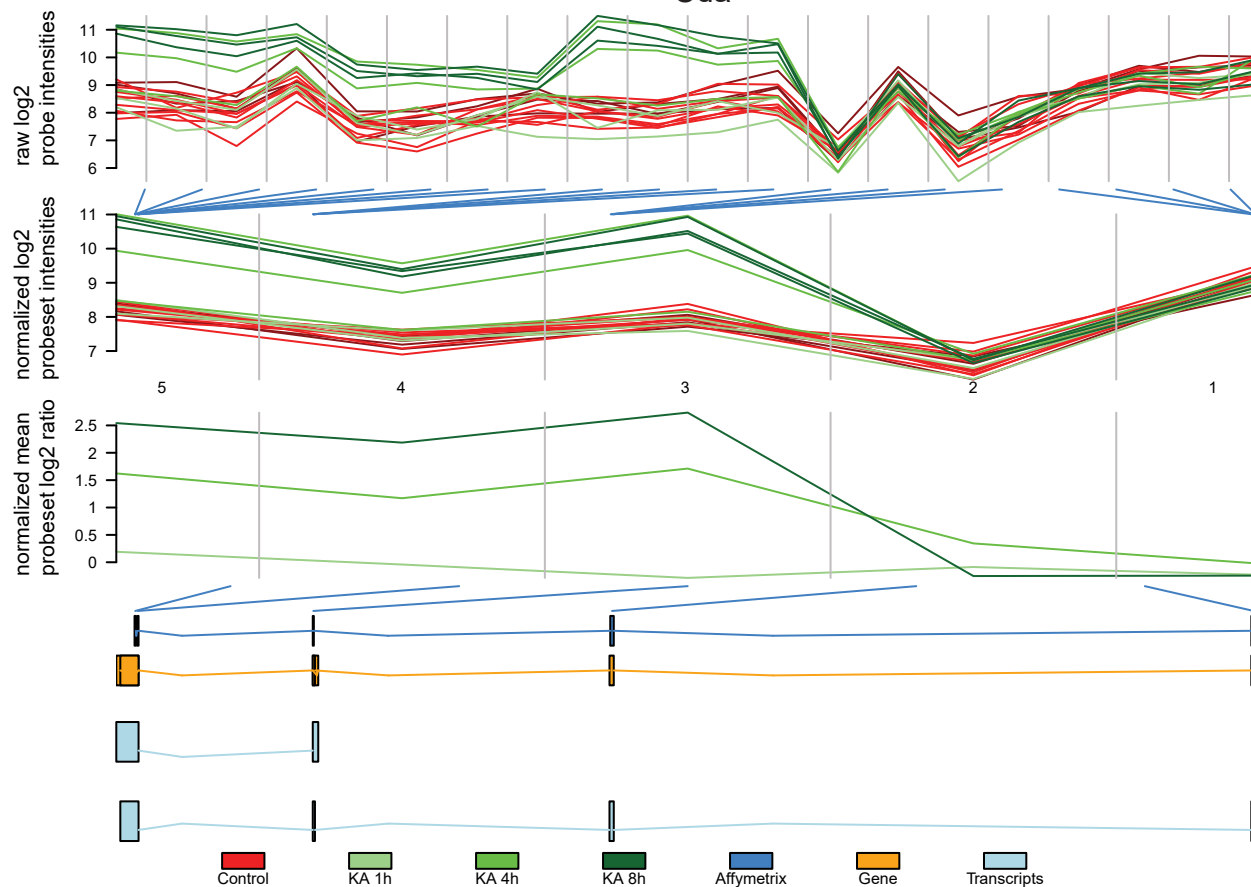

B

Errfi1

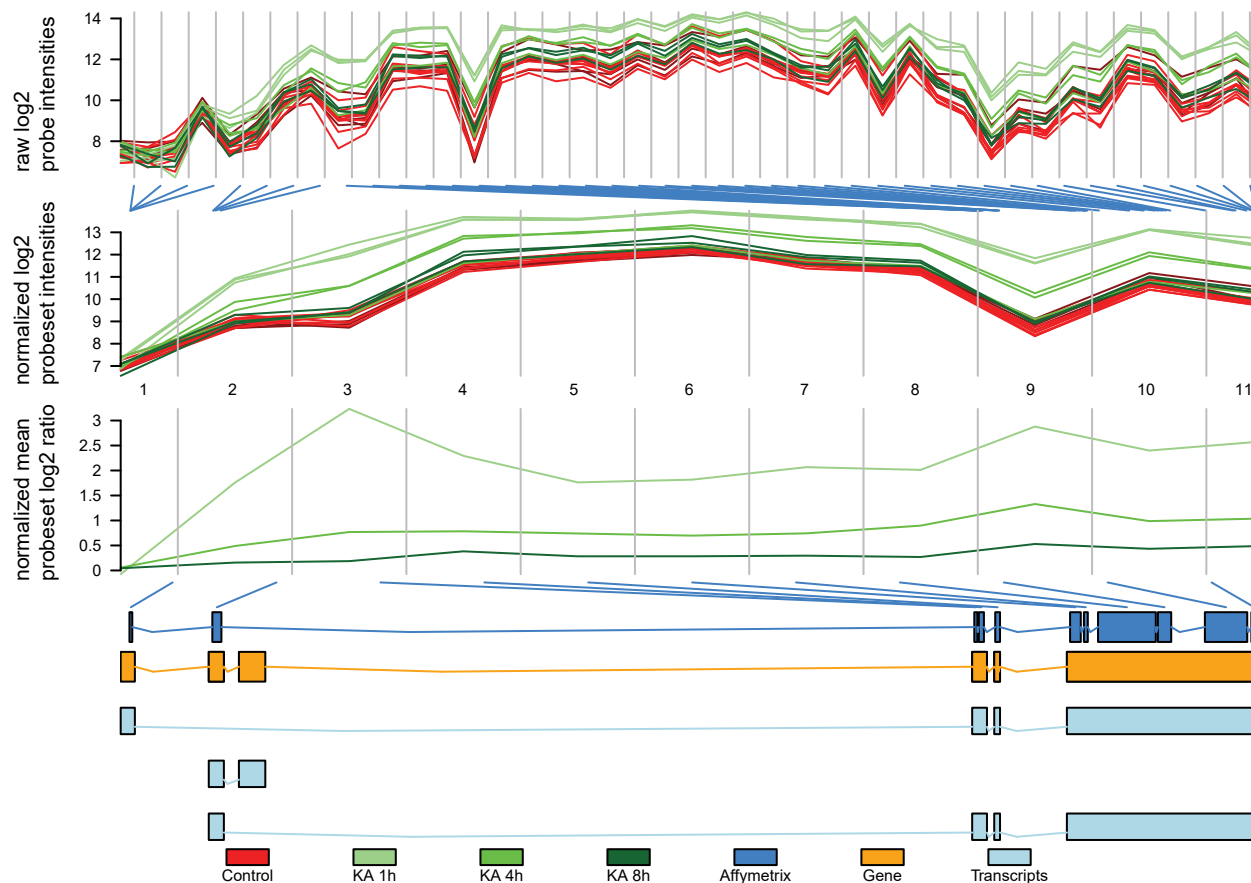

Supplement: Supplementary file 11 — Additional file 11: Gene expression profile of Cda and Errfi1. pdf. Analysis and visualization of the microarray data obtained for a Cda and b Errfi1. The upper 3 plots show the log2 transformed expression values over the course of its probes and probesets per exon. While the red lines show the intensity values of the control samples (vehicle and untreated), the 3 shades of green represent the expression intensities in the samples at the three different time points after seizures. The upper plot shows raw probe level intensities and grey bins stand for the individual probes. The middle plot shows the normalized intensities summarized to probeset level (probes divided by grey bins) for every sample. The lower plot shows the log2 ratio calculated from the mean of the intensities seen in the second plot, each line represents one of the 3 time points after seizure. At the bottom 3 types of exon models are depicted. The dark blue boxes represent the probesets per exons, which were included in the microarray analysis. The yellow boxes and track correspond to genomic sequence and each box indicates an exon. The light blue tracks and boxes represent the annotated transcripts present in the Ensembl database. [file 13041_2020_685_MOESM11_ESM.pdf]

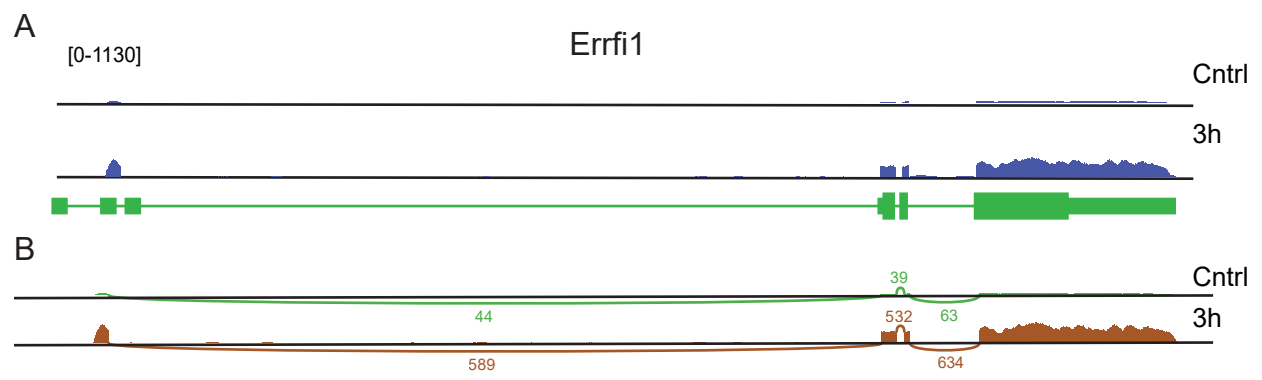

Supplement: Supplementary file 12 — Additional file 12: Activity-dependent splicing of Errfi1 in primary cultured hippocampal neurons. pdf. a Coverage plots (blue) from paired-end reads of RNA from untreated primary hippocampal neurons (upper panel) and 3 h after KCl induced depolarization (lower panel). The reference gene track of Errfi1 is depicted below (green). c Sashimi plot of RNA sequencing data from the untreated primary neurons (upper panel) or 3 h after KCl treatment (lower panel). Numbers indicate the counts of RNA sequencing reads that span the respective exon junctions. RNA-seq data was generated by Quesnel et al. and deposited in the GEO database under ID code GEO: GSE89984. [file 13041_2020_685_MOESM12_ESM.pdf]

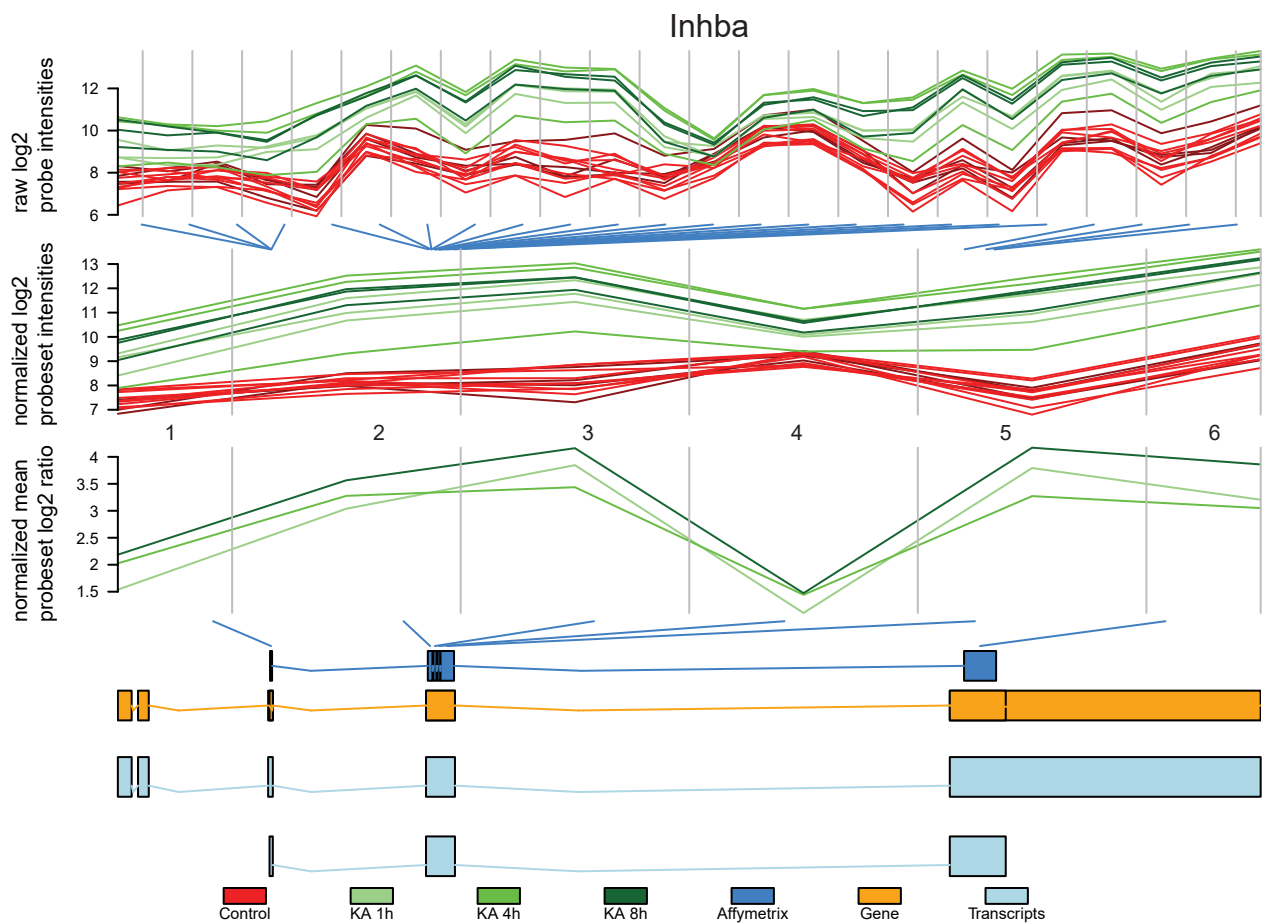

Supplement: Supplementary file 13 — Additional file 13: Gene expression profile of Inhba. pdf. Analysis and visualization of the microarray data obtained for Inhba. The upper 3 plots show the log2 transformed expression values of Inhba over the course of its probes and probesets per exons. While the red lines show the intensity values of the control samples (vehicle and untreated), the 3 shades of green represent the expression intensities in the samples at the three different time points after seizures. The upper plot shows raw probe level intensities and grey bins stand for the individual probes. The middle plot shows the normalized intensities summarized to probeset level (probes divided by grey bins) for every sample. The lower plot shows the log2 ratio calculated from the mean of the intensities seen in the second plot, each line represents one of the 3 time points after seizure. At the bottom 3 types of exon models are depicted. The dark blue boxes represent the probesets per exons, which were included in the microarray analysis. The yellow boxes and track correspond to genomic sequence and each box indicates an exon. The light blue tracks and boxes represent the only so far annotated transcript present in the Ensembl database. [file 13041_2020_685_MOESM13_ESM.pdf]

# Homer1

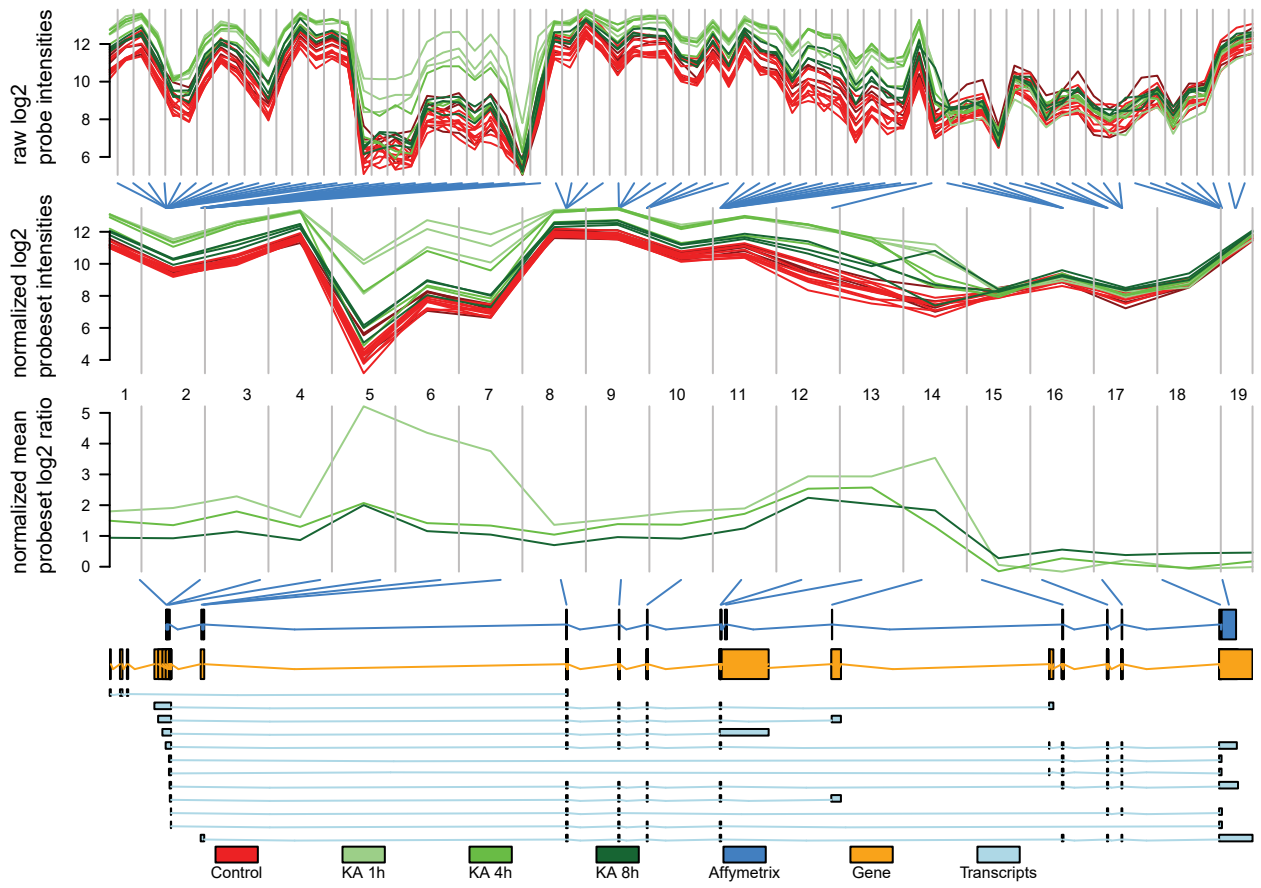

Supplement: Supplementary file 14 — Additional file 14: Gene expression profile of Homer1. pdf. Analysis and visualization of the microarray data obtained for Homer1. The upper 3 plots show the log2 transformed expression values of Homer1 over the course of its probes and probesets per exon. While the red lines show the intensity values of the control samples (vehicle and untreated), the 3 shades of green represent the expression intensities in the samples at the three different time points after seizures (compare color code at the bottom). The upper plot shows raw probe level intensities and grey bins stand for the individual probes. The middle plot shows the normalized intensities summarized to probeset level (probes divided by grey bins) for every sample. The lower plot shows the log2 ratio calculated from the mean of the intensities seen in the second plot, each line represents one of the 3 time points after seizure. At the bottom 3 types of exon models are depicted. The blue boxes represent the probesets per exons, which were included in the microarray experiments. The yellow boxes and track correspond to genomic sequence and each box indicates an exon. The light blue tracks and boxes represent transcripts as annotated in the Ensembl database. The blue lines below the first and third intensity plot show which probesets map to which exon. [file 13041_2020_685_MOESM14_ESM.pdf]

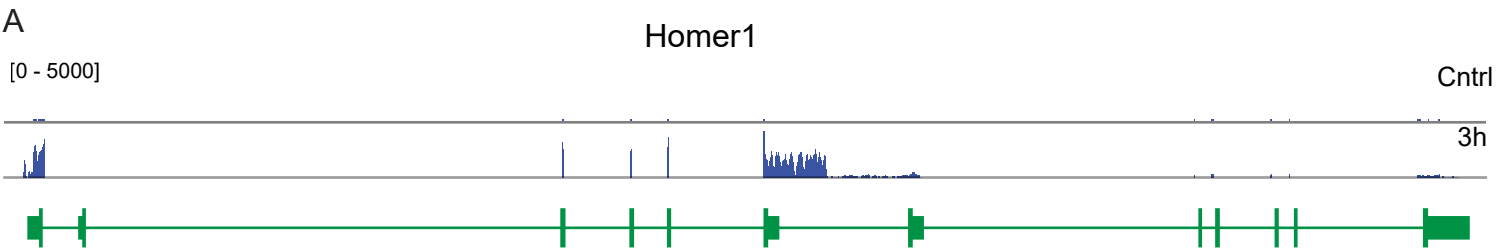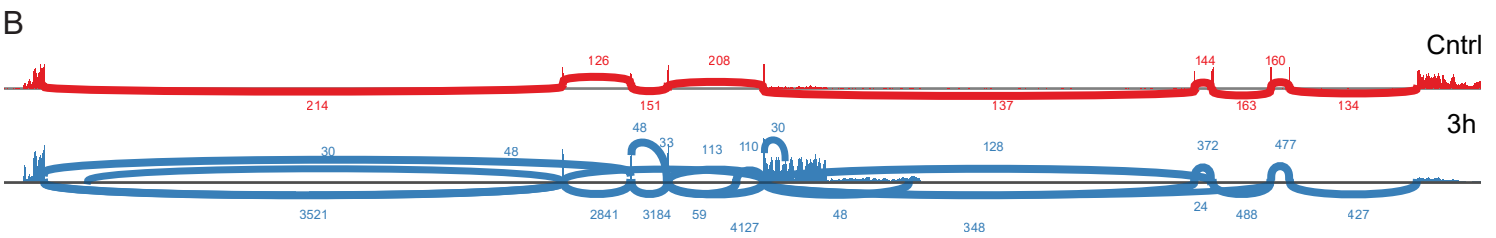

Supplement: Supplementary file 15 — Additional file 15: Activity-dependent splicing of Homer1 in primary cultured hippocampal neurons. pdf. a Homer1 expression in primary cultured hippocampal neurons of control mice or 3 h after KCl treatment detected by RNA sequencing. Shown are coverage plots (blue) from paired-end reads for the two sample groups. The reference gene track is depicted below (green). b Sashimi plot of RNA sequencing data for Homer1. Numbers indicate the counts of RNA sequencing reads that span the respective exon junctions. RNA-seq data was generated by Quesnel et al. and deposited in the GEO database under ID code GEO: GSE89984. [file 13041_2020_685_MOESM15_ESM.pdf]

Tpm1

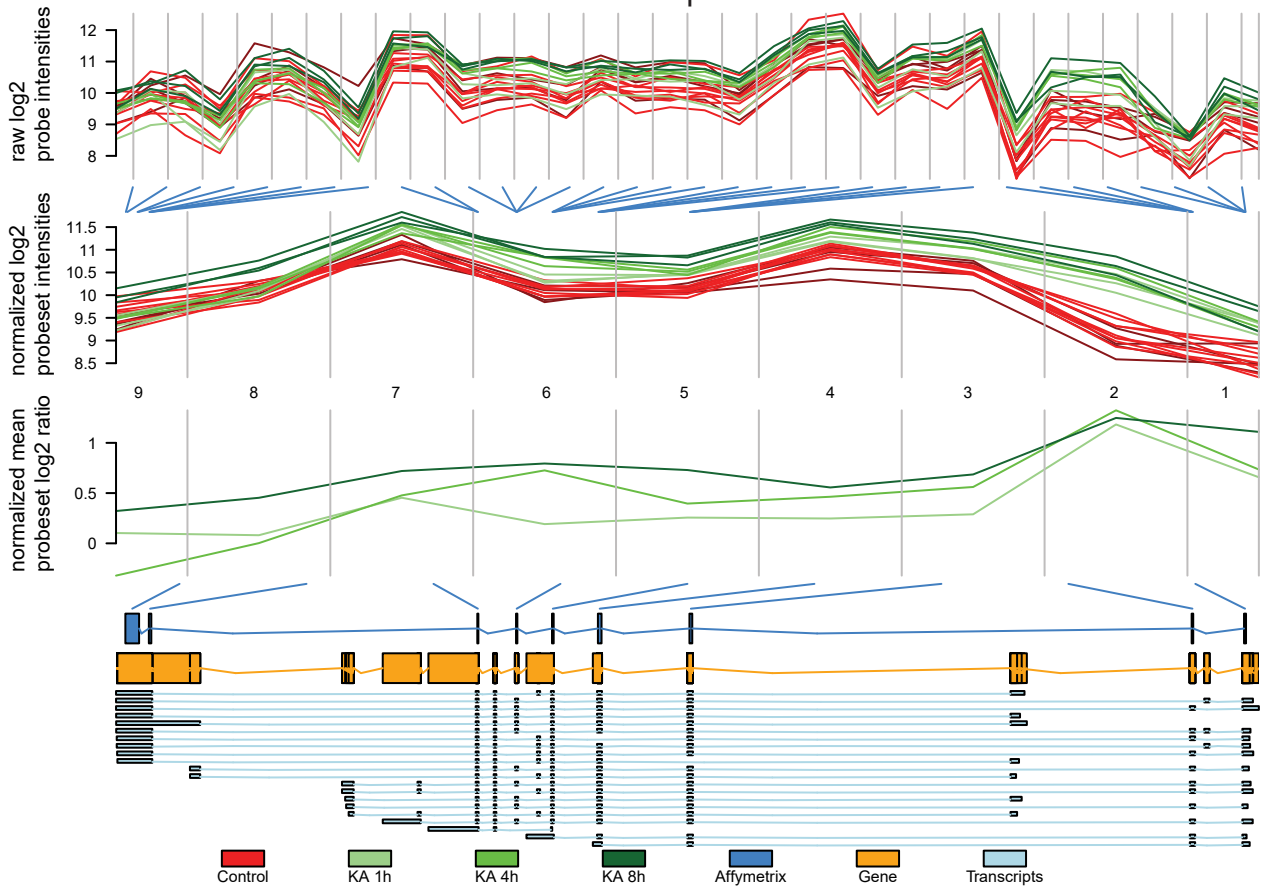

Supplement: Supplementary file 16 — Additional file 16: Gene expression profile of Tpm1. pdf. Analysis and visualization of the microarray data obtained for Tpm1. The upper 3 plots show the log2 transformed expression values of Tpm1 over the course of its probes and probesets per exon. While the red lines show the intensity values of the control samples (vehicle and untreated), the 3 shades of green represent the expression intensities in the samples at the three different time points after seizures. The upper plot shows raw probe level intensities and grey bins stand for the individual probes. The middle plot shows the normalized intensities summarized to probeset level (probes divided by grey bins) for every sample. The lower plot shows the log2 ratio calculated from the mean of the intensities seen in the second plot, each line represents one of the 3 time points after seizure. At the bottom 3 types of exon models are depicted. The dark blue boxes represent the probesets per exons, which were included in the microarray analysis. The yellow boxes and track correspond to genomic sequence and each box indicates an exon. The light blue tracks and boxes represent the only so far annotated transcript present in the Ensembl database. [file 13041_2020_685_MOESM16_ESM.pdf]

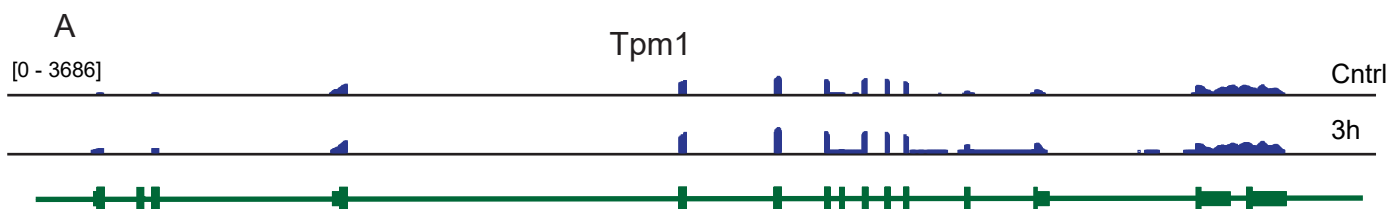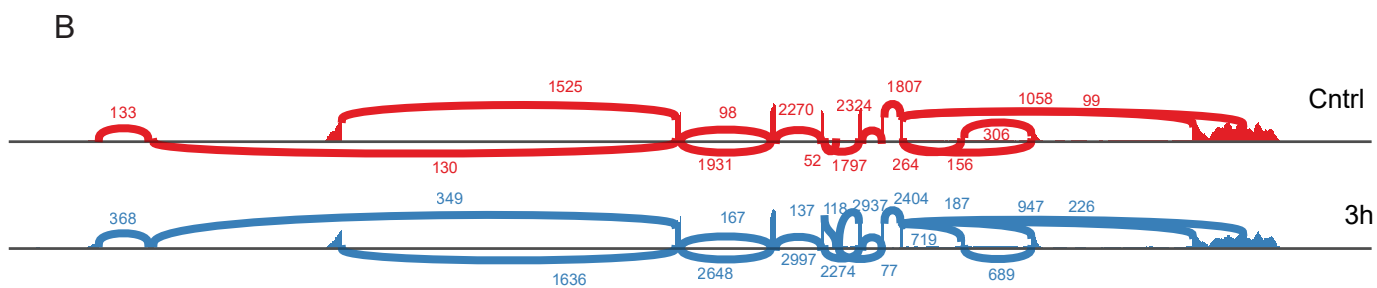

Supplement: Supplementary file 17 — Additional file 17: Activity-dependent splicing of Tpm1 in primary cultured hippocampal neurons. pdf. a Coverage plots (blue) from paired-end reads of RNA from untreated primary hippocampal neurons (upper panel) and 3 h after KCl induced depolarization (lower panel). The reference gene track is depicted below (green). b Sashimi plot of RNA sequencing data from untreated primary neurons (upper panel) or 3 h after KCl treatment (lower panel). Numbers indicate the counts of RNA sequencing reads that span the respective exon junctions. RNA-seq data was generated by Quesnel et al. and deposited in the GEO database under ID code GEO: GSE89984. [file 13041_2020_685_MOESM17_ESM.pdf]

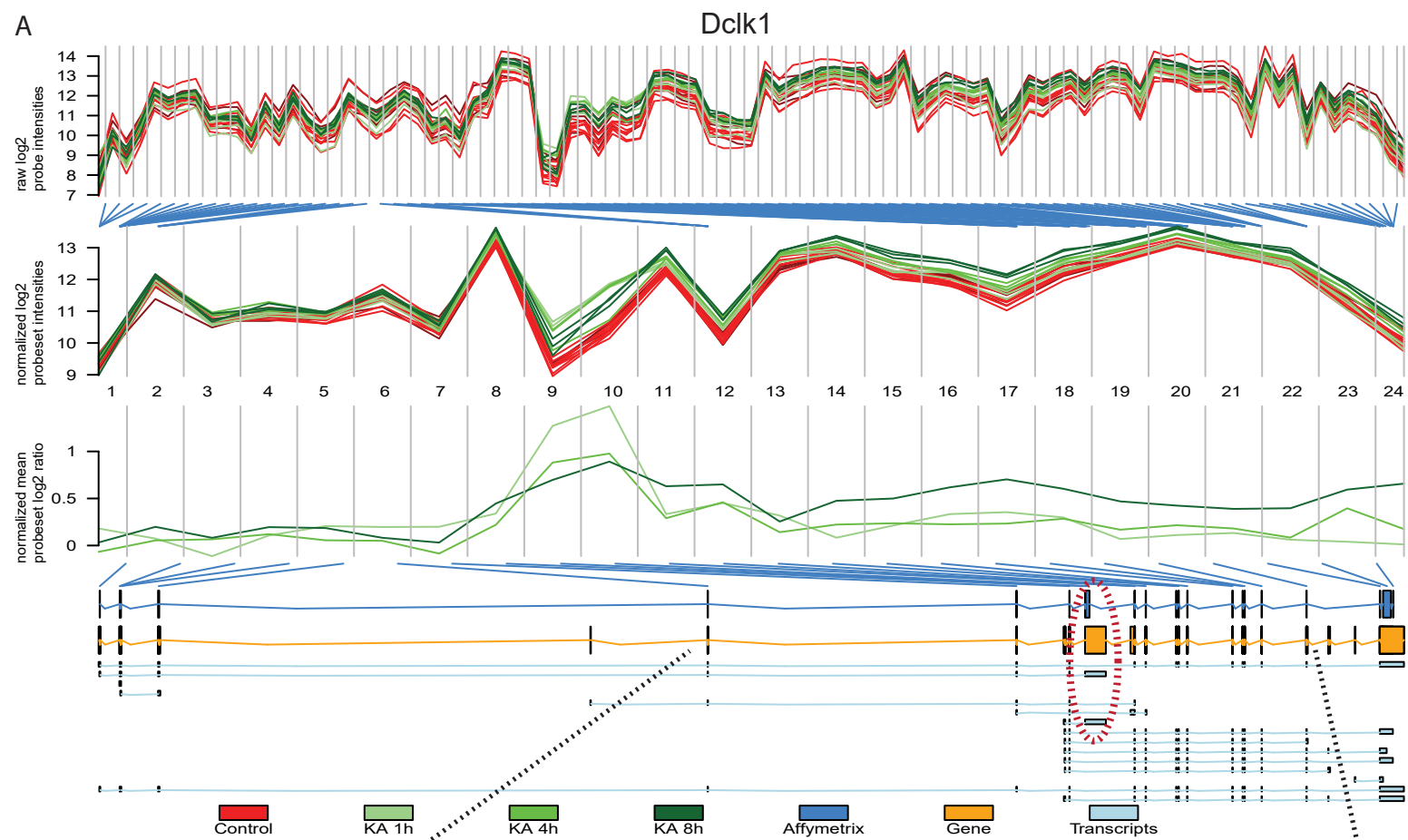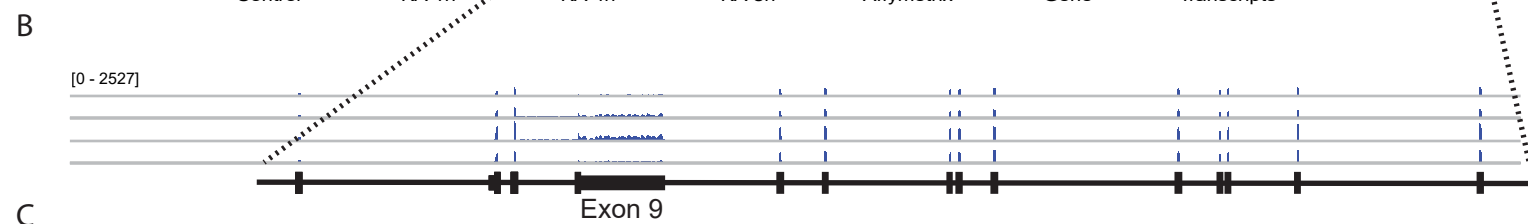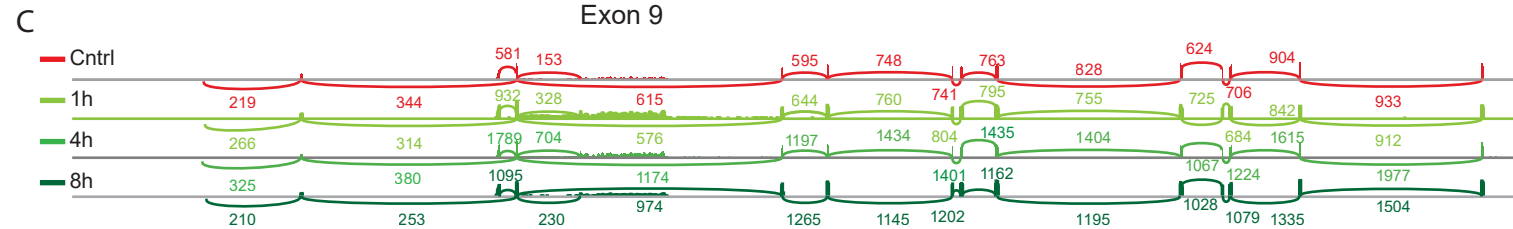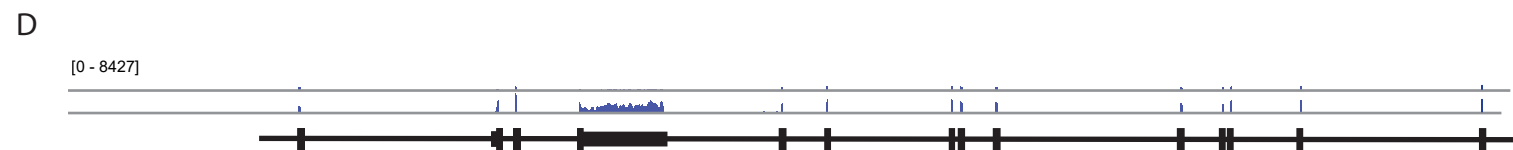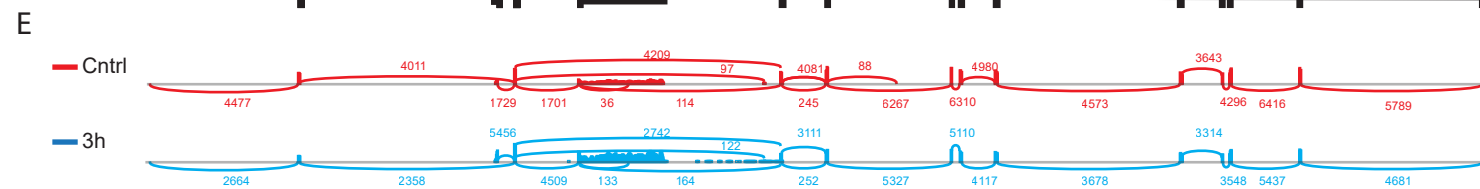

Supplement: Supplementary file 18 — Additional file 18: Gene expression profile of Dclk1. pdf. a Analysis and visualization of the microarray data obtained for Dclk1. The plots and color code are in correspondence to previous figures. The red dotted circle marks the activity-regulated exon 9. Black dotted lines indicate area between exon 6 and 19 that is magnified in B-D. b Validation of Dclk1 variant expression in hippocampus of control mice or 1, 4 or 8 h after seizure onset by RNA sequencing. Shown are coverage plots (blue) from paired-end reads for the four sample groups. The reference gene track of Dclk1 is depicted below (black). c Sashimi plot of RNA sequencing data for Dclk1. Numbers indicate the counts of RNA sequencing reads that span the respective exon junctions. d Coverage plots (blue) from paired-end reads of RNA from untreated primary hippocampal neurons (upper panel) and 3 h after KCl induced depolarization (lower panel). The reference gene track is depicted below (black). e Sashimi plot of RNA sequencing data from the untreated primary neurons (upper panel) or 3 h after KCl treatment (lower panel). Numbers indicate the counts of RNA sequencing reads that span the respective exon junctions. RNA-seq data was generated by Quesnel et al. and deposited in the GEO database under ID code GEO: GSE89984. [file 13041_2020_685_MOESM18_ESM.pdf]
