# Supplementary material for: Neuronal activity regulates alternative exon usage
Source: Mol Brain. 2020 Nov 10;13:148. doi: 10.1186/s13041-020-00685-3 (PMC7656758; doi:10.1186/s13041-020-00685-3)
Supplement: Supplementary file 6 — Additional file 6: Exon spanning primers. pdf [file 13041_2020_685_MOESM6_ESM.pdf]

Exon spanning primers

Gene	Exon	Orientation	Primer	Primer Name
Krt75	4	Forward	GCGAAGGTCGACTCTCTGAC	Krt75-4F
Krt75	5	Reverse	TGGCAATGTCCTCGTACTGG	Krt75-5R
Krt75	5	Forward	GCAGAACCAGGTGAGTGACA	Krt75-5F
Krt75	6	Reverse	CTTGTTTGGTGTTGCGGAGG	Krt75-6R
Krt75	9	Reverse	GCTGGGAAGGGTGCTATTGT	Krt75-9R
Rcan1	2	Forward	ACTGGAGCTTCATCGACTGC	Rcan1-2F
Rcan1	3	Forward	GTCGTTCGTTAAGCGTCTGC	Rcan1-3F
Rcan1	4	Reverse	CCCAGGAACTCGGTCTTGTG	Rcan1-4R
Cda	3	Forward	TTGTGAATTTCAGCCGTCGG	Cda-3F
Cda	4	Reverse	TCTTCAGGTCCAAACGAGGC	Cda-4Ra
Cda	4	Reverse	TGAGTGGCACCTTCCATCAC	Cda-4Rb
Errfi1	1	Forward	CGAACCCTCCTCCCTGTAGA	Errfi1-1F
Errfi1	2	Forward	AGCGAGCAGAGAGAAAGAGC	Errfi1-2F
Errfi1	4	Reverse	CCTGCTGTTGACATTGTGCC	Errfi1-4R
Inhba	2	Forward	GCGCTTTTAAACGAAGTTGC	Inhba-2F
Inhba	3	Forward	AAACAGAAGGGACCCGAAAG	Inhba-3F
Inhba	4	Reverse	CGGGTCTCTTCTTCAAGTGC	Inhba-4R
Inhba (lncRNA)	1	Reverse	CATTGACTCTCCCCCACACT	lncRNA-1R
Inhba (lncRNA)	2	Forward	GACAGGGGGAGGAGAGAATC	lncRNA-1F
Homer1	1-UTR	Forward	GTGTCAGCGCGAGTGAAATC	Homer1-1UF
Homer1	2-UTR	Forward	ATGGAACTGGGTGCTGCTAC	Homer1-2UF
Homer1	3	Reverse	AGACATGAGCTCGAGTGCTG	Homer1-3R
Homer1	5	Forward	GCTGACCAGTACCCCTTCAC	Homer1-5F
Homer1	6-UTR	Reverse	GGCCTGTGGTAAAGCTTTCC	Homer1-6UR
Homer1	6	Reverse	GCCCTTGGCTCTGAGTTCTG	Homer1-6R
Tpm1	1-UTR	Forward	CATATCAGGGAGCAGCAGGC	Tpm1-1UF
Tpm1	1	Forward	CCTTGGATCGAGCTGAGCAA	Tpm1-1F
Tpm1	3	Reverse	GCATCTTTGAGAGCCTCGGA	Tpm1-3R
Tpm1	3	Forward	TCCGAGGCTCTCAAAGATGC	Tpm1-3F
Tpm1	4	Forward	GGAGAAAGATCCGGAGCCTG	Tpm1-4F
Tpm1	4-UTR	Forward	ACTTCCGGCCTGCTTCTTAG	Tpm1-4UF
Tpm1	5	Reverse	CTCCTCCAGCTTCTGCAGAG	Tpm1-5R
Tpm1	11	Forward	TCGGGCTGAGTTTGCAGAG	Tpm1-11F
Tpm1	13-UTR	Reverse	CACTGGGCGAATTGCTTCTG	Tpm1-13UR
Tpm1	14	Reverse	AATCCTCATTCAGGGCCAGC	Tpm1-14R
Tpm1	14-UTR	Reverse	CCGCAGCTAAGGAGGGTTTC	Tpm1-14UR
