# Supplementary material for: Neuronal activity regulates alternative exon usage
Source: Mol Brain. 2020 Nov 10;13:148. doi: 10.1186/s13041-020-00685-3 (PMC7656758; doi:10.1186/s13041-020-00685-3)
Supplement: Supplementary file 7 — Additional file 7: Products of RT-PCR. pdf [file 13041_2020_685_MOESM7_ESM.pdf]

Products of RT-PCR

Gene	Exons	Forward Primer	Reverse Primer	Expected Product size [bp]	Shown in Figure*
Krt75	4-5	Krt75-4F	Krt75-5R	182	3E
Krt75	5-6	Krt75-5F	Krt75-6R	191	3E
Krt75	4-6 (incl. 5)	Krt75-4F	Krt75-6R	284	3E
Krt75	4-6 (excl. 5)	Krt75-4F	Krt75-6R	119	n.d. 3E
Krt75	4-9 (incl. 5, 6, 7, 8)	Krt75-4F	Krt75-6R	612	3E
Rcan1	2-4 (incl. 3)	Rcan1-2F	Rcan1-4R	438	n.d. 4E
Rcan1	2-4 (excl. 3)	Rcan1-2F	Rcan1-4R	256	4E
Rcan1	3-4	Rcan1-3F	Rcan1-4R	268	4E
Cda	3-4	Cda-3F	Cda-4Ra	225	5E
Cda	3-4	Cda-3F	Cda-4Rb	434	5E
Errfi1	1-4 (incl. 2)	Errfi1-1F	Errfi1-4R	380	n.d. 5J
Errfi1	1-4 (excl. 2)	Errfi1-1F	Errfi1-4R	183	n.d. 5J
Errfi1	2-4	Errfi1-2F	Errfi1-4R	222	5J
Inhba	2-4 (incl. 3)	Inhba-2F	Inhba-4R	571	6E
Inhba	2-4 (excl. 3)	Inhba-2F	Inhba-4R	489	6E
Inhba	3-4	Inhba-3F	Inhba-4R	351	6E
Inhba (lncRNA)	2-1	lncRNA-1F	lncRNA-1R	575	6F
Homer1	1-3 (incl. 2)	Homer1-1UF	Homer1-3R	279	n.d. 7E
Homer1	1-3 (excl. 2)	Homer1-1UF	Homer1-3R	226	7E
Homer1	2-3	Homer1-2UF	Homer1-3R	164	7E
Homer1	5-6	Homer1-5F	Homer1-6R	126	7E
Homer1	5-6UTR	Homer1-5F	Homer1-6UR	528	7E
Tpm1	1UTR-3 (incl. 2)	Tpm1-1UF	Tpm1-3R	435	n.d. 8E
Tpm1	1UTR-3 (excl. 2)	Tpm1-1UF	Tpm1-3R	309	8E
Tpm1	1-3 (incl. 2)	Tpm1-1F	Tpm1-3R	274	n.d. 8E
Tpm1	1-3 (excl. 2)	Tpm1-1F	Tpm1-3R	148	8E
Tpm1	3-5 (incl. 4)	Tpm1-3F	Tpm1-5R	297	n.d. 8E
Tpm1	3-5 (excl. 4)	Tpm1-3F	Tpm1-5R	165	8E
Tpm1	4-5	Tpm1-4F	Tpm1-5R	206	8E
Tpm1	4UTR-5	Tpm1-4UF	Tpm1-5R	384	8E
Tpm1	11-13UTR (incl. 12)	Tpm1-11F	Tpm1-13UR	235	8E
Tpm1	11-13UTR (excl. 12)	Tpm1-11F	Tpm1-13UR	314	8E
Tpm1	11-14 (incl. 12, 13)	Tpm1-11F	Tpm1-14R	269	n.d. 8E
Tpm1	11-14 (excl. 12, 13)	Tpm1-11F	Tpm1-14R	190	8E
Tpm1	11-14UTR (incl. 12)	Tpm1-11F	Tpm1-14UR	298	n.d. 8E
Tpm1	11-14UTR (excl. 12)	Tpm1-11F	Tpm1-14UR	219	8E

*n.d., not detected
