# Supplementary material for: Neuronal activity regulates alternative exon usage
Source: Mol Brain. 2020 Nov 10;13:148. doi: 10.1186/s13041-020-00685-3 (PMC7656758; doi:10.1186/s13041-020-00685-3)
Supplement: Supplementary file 19 — Additional file 19: Modified Racine scale (behavioral scoring in response to kainic acid). pdf [file 13041_2020_685_MOESM19_ESM.pdf]

**Modified Racine scale (behavioral scoring in response to kainic acid)**

| **Classification** | **Modified Racine scale** |
|---|---|
| Stage 1 | Immobility / Flattening |
| Stage 2 | Forelimb and / or tail extension, rigid posture |
| Stage 3 | Repetitive movements, including head bobs |
| Stage 4 | Rearing and falling |
| Stage 5 | Continuos rearing and falling, barrel rolling |
| Stage 6 | Severe tonic-clonic seizures |
